# Diretriz de Síndrome Coronariana Crônica – 2025

**DOI:** 10.36660/abc.20250619

**Published:** 2025-09-16

**Authors:** Luiz A. Machado Cesar, Luis Henrique W. Gowdak, Ricardo Pavanello, João Fernando M. Ferreira, Bruno M. Mioto, Nilson T. Poppi, Caio Menezes M. de Mendonça, Adenalva Lima de Souza Beck, Adriana Soares Xavier de Brito, Alexandre Abizaid, Andréa Araujo Brandão, Andrea Maria Gomes Marinho Falcão, Andrei Sposito, Antônio Carlos Sobral Sousa, Antonio de Padua Mansur, Ariane Vieira Scarlatelli Macedo, Áurea Jacob Chaves, Breno de Alencar Araripe Falcão, Brivaldo Markman, Bruno Ramos Nascimento, Camila Paixão Jordão, Carlos Augusto Homem de Magalhães Campos, Carlos Eduardo Lucena Montenegro, Carlos Eduardo Rochitte, Carlos Vicente Serrano, Celia Maria Cassaro Strunz, Daniel Medeiros Moreira, Danielle Misumi Watanabe, Eduardo Gomes Lima, Eduardo Nagib Gaui, Elizabeth Regina Giunco Alexandre, Fabiana Hanna Rached, Fabio Biscegli Jatene, Gentil Barreira de Aguiar, Gilson Soares Feitosa-Filho, Henrique Patrus Mundim Pena, Ibraim Masciarelli Francisco Pinto, Iran Castro, Jaqueline R. Scholz, João Carlos Ferreira Leal, José Armando Mangione, José Jayme Galvão de Lima, Jose Rocha Faria, José Soares, Kleisson Antônio Pontes Maia, Lara Cristiane Terra Ferreira Carreira, Luciana Diniz Nagem Janot de Matos, Luciana Oliveira Cascaes Dourado, Luhanda Leonora Cardoso Monti Sousa, Luis Alberto Oliveira Dallan, Luiz Eduardo Mastrocola, Marcia Maria Godoy Gowdak, Olimpio Ribeiro França, Otavio Rizzi Coelho, Otávio R. Coelho-Filho, Paulo Eduardo Ballvé Behr, Paulo Ricardo Avancini Caramori, Pedro Alves Lemos, Pedro Silvio Farsky, Raul Dias dos Santos, Renato D. Lopes, Salvador Manoel Serra, Sarah Fagundes Grobe, Sérgio Tavares Montenegro, Silvio Henrique Barberato, Tania Mara Varejão Strabelli, Ursula Maria Moreira Costa Burgos, Vinicius José da Silva Nina, Walter Jose Gomes, William Azem Chalela, Wilson Mathias

**Affiliations:** 1 Hospital das Clínicas da Faculdade de Medicina da Universidade de São Paulo Instituto do Coração São Paulo SP Brasil Instituto do Coração (Incor) do Hospital das Clínicas da Faculdade de Medicina da Universidade de São Paulo (HCFMUSP), São Paulo, SP – Brasil; 2 Instituto Dante Pazzanese de Cardiologia São Paulo SP Brasil Instituto Dante Pazzanese de Cardiologia, São Paulo, SP – Brasil; 3 Instituto de Cardiologia e Transplantes do Distrito Federal Brasília DF Brasil Instituto de Cardiologia e Transplantes do Distrito Federal, Brasília, DF – Brasil; 4 Hospital Sírio Libanês Brasília Brasília DF Brasil Hospital Sírio Libanês Brasília, Brasília, DF – Brasil; 5 Instituto Nacional de Cardiologia Rio de Janeiro RJ Brasil Instituto Nacional de Cardiologia, Rio de Janeiro, RJ – Brasil; 6 Rede D’Or Rio de Janeiro RJ Brasil Rede D’Or, Rio de Janeiro, RJ – Brasil; 7 DASA Rio de Janeiro RJ Brasil DASA, Rio de Janeiro, RJ – Brasil; 8 Universidade do Estado do Rio de Janeiro Rio de Janeiro RJ Brasil Universidade do Estado do Rio de Janeiro (UERJ), Rio de Janeiro, RJ – Brasil; 9 Universidade Estadual de Campinas Campinas SP Brasil Universidade Estadual de Campinas (UNICAMP), Campinas, SP – Brasil; 10 Universidade Federal de Sergipe Aracaju SE Brasil Universidade Federal de Sergipe (UFS), Aracaju, SE – Brasil; 11 Hospital São Lucas, Rede D'Or de Aracaju Aracaju SE Brasil Hospital São Lucas, Rede D'Or de Aracaju, Aracaju, SE – Brasil; 12 Faculdade de Ciências Médicas da Santa Casa de São Paulo São Paulo SP Brasil Faculdade de Ciências Médicas da Santa Casa de São Paulo, São Paulo, SP – Brasil; 13 Hospital de Messejana Fortaleza CE Brasil Hospital de Messejana, Fortaleza, CE – Brasil; 14 Universidade Federal do Ceará Hospital Universitário Walter Cantídio Fortaleza CE Brasil Hospital Universitário Walter Cantídio, Universidade Federal do Ceará, Fortaleza, CE – Brasil; 15 Hospital Monte Klinikum Fortaleza CE Brasil Hospital Monte Klinikum, Fortaleza, CE – Brasil; 16 Universidade Federal de Pernambuco Recife PE Brasil Universidade Federal de Pernambuco, Recife, PE – Brasil; 17 Universidade Federal de Minas Gerais Belo Horizonte MG Brasil Universidade Federal de Minas Gerais, Belo Horizonte, MG – Brasil; 18 Hospital do Coração de PE Recife PE Brasil Hospital do Coração de PE (PROCAPE), Recife, PE – Brasil; 19 Universidade de Pernambuco Recife PE Brasil Universidade de Pernambuco (UPE), Recife, PE – Brasil; 20 Hospital Esperança Recife, Rede D'or Recife PE Brasil Hospital Esperança Recife, Rede D'or, Recife, PE – Brasil; 21 Instituto de Cardiologia de Santa Catarina São José SC Brasil Instituto de Cardiologia de Santa Catarina, São José, SC – Brasil; 22 Hospital Municipal Miguel Couto Rio de Janeiro RJ Brasil Hospital Municipal Miguel Couto, Rio de Janeiro, RJ – Brasil; 23 Hospital do Coração São Paulo SP Brasil Hospital do Coração (HCor), São Paulo, SP – Brasil; 24 Escola Bahiana de Medicina e Saúde Pública Salvador BA Brasil Escola Bahiana de Medicina e Saúde Pública, Salvador, BA – Brasil; 25 Hospital Santa Izabel Salvador BA Brasil Hospital Santa Izabel, Salvador, BA – Brasil; 26 Santa Casa de Misericórdia da Bahia Salvador BA Brasil Santa Casa de Misericórdia da Bahia, Salvador, BA – Brasil; 27 Rede Mater Dei de Saúde Belo Horizonte MG Brasil Rede Mater Dei de Saúde, Belo Horizonte, MG – Brasil; 28 Grupo Fleury São Paulo SP Brasil Grupo Fleury, São Paulo, SP – Brasil; 29 Instituto de Cardiologia do Rio Grande do Sul Porto Alegre RS Brasil Instituto de Cardiologia do Rio Grande do Sul, Porto Alegre, RS – Brasil; 30 Faculdade de Medicina de São José do Rio Preto São José do Rio Preto SP Brasil Faculdade de Medicina de São José do Rio Preto (FAMERP), São José do Rio Preto, SP – Brasil; 31 Hospital Beneficência Portuguesa de São José do Rio Preto São José do Rio Preto SP Brasil Hospital Beneficência Portuguesa de São José do Rio Preto, São José do Rio Preto, SP – Brasil; 32 Hospital Beneficência Portuguesa de São Paulo São Paulo SP Brasil Hospital Beneficência Portuguesa de São Paulo, São Paulo, SP – Brasil; 33 Pontifícia Universidade Católica do Paraná Curitiba PR Brasil Pontifícia Universidade Católica do Paraná, Curitiba, PR – Brasil; 34 Faculdade Ciências Médicas de Minas Gerais Belo Horizonte MG Brasil Faculdade Ciências Médicas de Minas Gerais, Belo Horizonte, MG – Brasil; 35 Cardiologia Nuclear de Curitiba Curitiba PR Brasil Cardiologia Nuclear de Curitiba (CNC), Curitiba, PR – Brasil; 36 Hospital Israelita Albert Einstein São Paulo SP Brasil Hospital Israelita Albert Einstein, São Paulo, SP – Brasil; 37 Escola Vera Cruz São Paulo SP Brasil Escola Vera Cruz, São Paulo, SP – Brasil; 38 Quanta Diagnóstico Curitiba PR Brasil Quanta Diagnóstico, Curitiba, PR – Brasil; 39 Santa Casa de Porto Alegre Porto Alegre RS Brasil Santa Casa de Porto Alegre, Porto Alegre, RS – Brasil; 40 Hospital São Lucas da Pontifícia Universidade Católica do Rio Grande do Sul Porto Alegre RS Brasil Hospital São Lucas da Pontifícia Universidade Católica do Rio Grande do Sul (PUCRS), Porto Alegre, RS – Brasil; 41 Universidade de São Paulo São Paulo SP Brasil Universidade de São Paulo (USP), São Paulo, SP – Brasil; 42 Duke University Medical Center Durham EUA Duke University Medical Center, Durham – EUA; 43 Instituto Estadual de Cardiologia Aloysio de Castro Rio de Janeiro RJ Brasil Instituto Estadual de Cardiologia Aloysio de Castro, Rio de Janeiro, RJ – Brasil; 44 Centro de Diagnóstico Cardiovascular Curitiba PR Brasil Centro de Diagnóstico Cardiovascular (CardioEco), Curitiba, PR – Brasil; 45 Universidade Tiradentes Aracaju SE Brasil Universidade Tiradentes, Aracaju, SE – Brasil; 46 Universidade Federal do Maranhão São Luís MA Brasil Universidade Federal do Maranhão, São Luís, MA – Brasil; 47 Universidade Federal de São Paulo São Paulo SP Brasil Universidade Federal de São Paulo, São Paulo, SP – Brasil

**Table:** 

Diretriz de Síndrome Coronariana Crônica – 2025	
O relatório abaixo lista as declarações de interesse conforme relatadas à SBC pelos especialistas durante o período de desenvolvimento deste posicionamento, 2024/2025.	
Especialista	Tipo de relacionamento com a indústria
Adenalva Lima de Souza Beck	Nada a ser declarado
Adriana Soares Xavier de Brito	Nada a ser declarado
Alexandre Abizaid	Outros relacionamentos Financiamento de atividades de educação médica continuada, incluindo viagens, hospedagens e inscrições para congressos e cursos, provenientes da indústria farmacêutica, de órteses, próteses, equipamentos e implantes, brasileiras ou estrangeiras: - CRF, Polares, Shiffamed.
Andréa Araujo Brandão	Declaração financeira A - Pagamento de qualquer espécie e desde que economicamente apreciáveis, feitos a (i) você, (ii) ao seu cônjuge/ companheiro ou a qualquer outro membro que resida com você, (iii) a qualquer pessoa jurídica em que qualquer destes seja controlador, sócio, acionista ou participante, de forma direta ou indireta, recebimento por palestras, aulas, atuação como proctor de treinamentos, remunerações, honorários pagos por participações em conselhos consultivos, de investigadores, ou outros comitês, etc. Provenientes da indústria farmacêutica, de órteses, próteses, equipamentos e implantes, brasileiras ou estrangeiras: - Biolab: Nebivolol; AstraZeneca: Metoprolol; Servier: Perindopril, Hypera; Mantecorp: Olmesartana e combinações; Torrent: Olmesartana e combinações; Daiichi Sankyo: Olmesartana e combinações. B - Financiamento de pesquisas sob sua responsabilidade direta/pessoal (direcionado ao departamento ou instituição) provenientes da indústria farmacêutica, de órteses, próteses, equipamentos e implantes, brasileiras ou estrangeiras: - Daiichi Sankyo: Olmesartana; Servier: Elfie; Brainfarma: Olmesartana. Outros relacionamentos Financiamento de atividades de educação médica continuada, incluindo viagens, hospedagens e inscrições para congressos e cursos, provenientes da indústria farmacêutica, de órteses, próteses, equipamentos e implantes, brasileiras ou estrangeiras: - Servier: Perindopril; Novo Nordisk: Semaglutida.
Andrea Maria Gomes Marinho Falcão	Nada a ser declarado
Andrei Sposito	Declaração financeira A - Pagamento de qualquer espécie e desde que economicamente apreciáveis, feitos a (i) você, (ii) ao seu cônjuge/ companheiro ou a qualquer outro membro que resida com você, (iii) a qualquer pessoa jurídica em que qualquer destes seja controlador, sócio, acionista ou participante, de forma direta ou indireta, recebimento por palestras, aulas, atuação como proctor de treinamentos, remunerações, honorários pagos por participações em conselhos consultivos, de investigadores, ou outros comitês, etc. Provenientes da indústria farmacêutica, de órteses, próteses, equipamentos e implantes, brasileiras ou estrangeiras: - Lilly, Novo Nordisk, Daiichi Sankyo. B - Financiamento de pesquisas sob sua responsabilidade direta/pessoal (direcionado ao departamento ou instituição) provenientes da indústria farmacêutica, de órteses, próteses, equipamentos e implantes, brasileiras ou estrangeiras: - AstraZeneca. Outros relacionamentos Financiamento de atividades de educação médica continuada, incluindo viagens, hospedagens e inscrições para congressos e cursos, provenientes da indústria farmacêutica, de órteses, próteses, equipamentos e implantes, brasileiras ou estrangeiras: - Daiichi, Novo Nordisk.
Antônio Carlos Sobral Sousa	Nada a ser declarado
Antonio de Padua Mansur	Nada a ser declarado
Ariane Vieira Scarlatelli Macedo	Declaração financeira A - Pagamento de qualquer espécie e desde que economicamente apreciáveis, feitos a (i) você, (ii) ao seu cônjuge/ companheiro ou a qualquer outro membro que resida com você, (iii) a qualquer pessoa jurídica em que qualquer destes seja controlador, sócio, acionista ou participante, de forma direta ou indireta, recebimento por palestras, aulas, atuação como proctor de treinamentos, remunerações, honorários pagos por participações em conselhos consultivos, de investigadores, ou outros comitês, etc. Provenientes da indústria farmacêutica, de órteses, próteses, equipamentos e implantes, brasileiras ou estrangeiras: - Adium: câncer de mama; Amgen: Mieloma; Astellas: câncer de próstata; AstraZeneca: Forxiga; BeOne: Zanubrutinibe; BMS: Mielodisplasia e Mavacanteno; Bayer:câncer de próstata; Edwards: estenose aórtica; Ferring: câncer de próstata; Jonhson: câncer de próstata e LLC; Novo Nordisk: Diabetes; Novartis: Sybrava, Entresto; Pfizer: amiloidose e LMC. B - Financiamento de pesquisas sob sua responsabilidade direta/pessoal (direcionado ao departamento ou instituição) provenientes da indústria farmacêutica, de órteses, próteses, equipamentos e implantes, brasileiras ou estrangeiras: - Ferring: câncer de próstata. Outros relacionamentos Financiamento de atividades de educação médica continuada, incluindo viagens, hospedagens e inscrições para congressos e cursos, provenientes da indústria farmacêutica, de órteses, próteses, equipamentos e implantes, brasileiras ou estrangeiras: - Novo Nordisk: diabetes; Novartis: Entresto, Sybrava.
Áurea Jacob Chaves	Nada a ser declarado
Breno de Alencar Araripe Falcão	Nada a ser declarado
Brivaldo Markman Filho	Nada a ser declarado
Bruno Mahler Mioto	Declaração financeira A - Pagamento de qualquer espécie e desde que economicamente apreciáveis, feitos a (i) você, (ii) ao seu cônjuge/ companheiro ou a qualquer outro membro que resida com você, (iii) a qualquer pessoa jurídica em que qualquer destes seja controlador, sócio, acionista ou participante, de forma direta ou indireta, recebimento por palestras, aulas, atuação como proctor de treinamentos, remunerações, honorários pagos por participações em conselhos consultivos, de investigadores, ou outros comitês, etc. Provenientes da indústria farmacêutica, de órteses, próteses, equipamentos e implantes, brasileiras ou estrangeiras: - Servier: doença coronária; Novo Nordisk: diabetes e obesidade; Novartis: dislipidemia; Daiichi Sankyo: Dislipidemia; Boehringer Ingelheim: diabetes. Outros relacionamentos Financiamento de atividades de educação médica continuada, incluindo viagens, hospedagens e inscrições para congressos e cursos, provenientes da indústria farmacêutica, de órteses, próteses, equipamentos e implantes, brasileiras ou estrangeiras: - Servier: doença coronária; Novo Nordisk: diabetes e obesidade.
Bruno Ramos Nascimento	Nada a ser declarado
Caio Menezes Machado de Mendonça	Declaração financeira A - Pagamento de qualquer espécie e desde que economicamente apreciáveis, feitos a (i) você, (ii) ao seu cônjuge/ companheiro ou a qualquer outro membro que resida com você, (iii) a qualquer pessoa jurídica em que qualquer destes seja controlador, sócio, acionista ou participante, de forma direta ou indireta, recebimento por palestras, aulas, atuação como proctor de treinamentos, remunerações, honorários pagos por participações em conselhos consultivos, de investigadores, ou outros comitês, etc. Provenientes da indústria farmacêutica, de órteses, próteses, equipamentos e implantes, brasileiras ou estrangeiras: - Servier: Vastarel, síndrome coronariana crônica; Novartis: Sybrava, dislipidemia; Chiesi: Trimbow, DPOC; Libbs: Plenance, dislipidemia; Novo Nordisk: Rybelsus. Outros relacionamentos Financiamento de atividades de educação médica continuada, incluindo viagens, hospedagens e inscrições para congressos e cursos, provenientes da indústria farmacêutica, de órteses, próteses, equipamentos e implantes, brasileiras ou estrangeiras: - Servier: Congresso Brasileiro de Cardiologia 2024; Daiichi-Sankyo: Congresso da SOCESP 2024; Novo Nordisk: Congresso da SOCESP 2025; Novartis: Congresso.
Camila Paixão Jordão	Nada a ser declarado
Carlos Augusto Homem de Magalhães Campos	Nada a ser declarado
Carlos Eduardo Lucena Montenegro	Declaração financeira A - Pagamento de qualquer espécie e desde que economicamente apreciáveis, feitos a (i) você, (ii) ao seu cônjuge/ companheiro ou a qualquer outro membro que resida com você, (iii) a qualquer pessoa jurídica em que qualquer destes seja controlador, sócio, acionista ou participante, de forma direta ou indireta, recebimento por palestras, aulas, atuação como proctor de treinamentos, remunerações, honorários pagos por participações em conselhos consultivos, de investigadores, ou outros comitês, etc. Provenientes da indústria farmacêutica, de órteses, próteses, equipamentos e implantes, brasileiras ou estrangeiras: - Bayer: Firialta (IC); Pfizer: Vyndaquel/ Vynkella (Amiloidose); Alnylan: Amvuttra (Amiloidose) Servier: Vastarel (DAC); Novartis: Entresto, Sybrava (IC, DLP); AstraZeneca: Forxiga, Lokelma, Breztri (IC, DRC, DPOC); Viatris: Inspra (IC); Merck: Concor (IC, DAC); Novo Nordisk: Wegovy (Obesidade, IC); EMS: Vynaxa (DAC). Outros relacionamentos Financiamento de atividades de educação médica continuada, incluindo viagens, hospedagens e inscrições para congressos e cursos, provenientes da indústria farmacêutica, de órteses, próteses, equipamentos e implantes, brasileiras ou estrangeiras: - Novo Nordisk: Wegovy (Obesidade, IC).
Carlos Eduardo Rochitte	Declaração financeira A - Pagamento de qualquer espécie e desde que economicamente apreciáveis, feitos a (i) você, (ii) ao seu cônjuge/ companheiro ou a qualquer outro membro que resida com você, (iii) a qualquer pessoa jurídica em que qualquer destes seja controlador, sócio, acionista ou participante, de forma direta ou indireta, recebimento por palestras, aulas, atuação como proctor de treinamentos, remunerações, honorários pagos por participações em conselhos consultivos, de investigadores, ou outros comitês, etc. Provenientes da indústria farmacêutica, de órteses, próteses, equipamentos e implantes, brasileiras ou estrangeiras: - GE Healthcare, Canon Medical Systems, Novartis, Cleerly.
Carlos Vicente Serrano Jr.	Nada a ser declarado
Celia Maria Cassaro Strunz	Nada a ser declarado
Daniel Medeiros Moreira	Nada a ser declarado
Danielle Misumi Watanabe	Nada a ser declarado
Eduardo Gomes Lima	Declaração financeira A - Pagamento de qualquer espécie e desde que economicamente apreciáveis, feitos a (i) você, (ii) ao seu cônjuge/ companheiro ou a qualquer outro membro que resida com você, (iii) a qualquer pessoa jurídica em que qualquer destes seja controlador, sócio, acionista ou participante, de forma direta ou indireta, recebimento por palestras, aulas, atuação como proctor de treinamentos, remunerações, honorários pagos por participações em conselhos consultivos, de investigadores, ou outros comitês, etc. Provenientes da indústria farmacêutica, de órteses, próteses, equipamentos e implantes, brasileiras ou estrangeiras: - Novo Nordisk: obesidade e diabetes; Daiichi Sankyo: dislipidemia e antiagregação; Novartis: Inclisirana; Bayer: finerenona; AstraZeneca: forxiga; Lilly: Mounjaro. B - Financiamento de pesquisas sob sua responsabilidade direta/pessoal (direcionado ao departamento ou instituição) provenientes da indústria farmacêutica, de órteses, próteses, equipamentos e implantes, brasileiras ou estrangeiras: - Lilly: Mounjaro; Novo Nordisk: Ziltivekimab; Novartis: Inclisirana. Outros relacionamentos Financiamento de atividades de educação médica continuada, incluindo viagens, hospedagens e inscrições para congressos e cursos, provenientes da indústria farmacêutica, de órteses, próteses, equipamentos e implantes, brasileiras ou estrangeiras: - Novo Nordisk: obesidade e diabetes; Lilly: obesidade.
Eduardo Nagib Gaui	Nada a ser declarado
Elizabeth Regina Giunco Alexandre	Declaração financeira A - Pagamento de qualquer espécie e desde que economicamente apreciáveis, feitos a (i) você, (ii) ao seu cônjuge/ companheiro ou a qualquer outro membro que resida com você, (iii) a qualquer pessoa jurídica em que qualquer destes seja controlador, sócio, acionista ou participante, de forma direta ou indireta, recebimento por palestras, aulas, atuação como proctor de treinamentos, remunerações, honorários pagos por participações em conselhos consultivos, de investigadores, ou outros comitês, etc. Provenientes da indústria farmacêutica, de órteses, próteses, equipamentos e implantes, brasileiras ou estrangeiras: - Servier: Vastarel MR; Lilly: Mounjaro; Libbs: Ebatz e Stanglitz, Novo Nordisk: Ozempic; AstraZeneca: Breztri; Boehringer-Ingelhein: Glyxambi; Mantecorpp: Nesina/Addera. Outros relacionamentos Financiamento de atividades de educação médica continuada, incluindo viagens, hospedagens e inscrições para congressos e cursos, provenientes da indústria farmacêutica, de órteses, próteses, equipamentos e implantes, brasileiras ou estrangeiras: - Lilly.
Fabiana Hanna Rached	Declaração financeira A - Pagamento de qualquer espécie e desde que economicamente apreciáveis, feitos a (i) você, (ii) ao seu cônjuge/ companheiro ou a qualquer outro membro que resida com você, (iii) a qualquer pessoa jurídica em que qualquer destes seja controlador, sócio, acionista ou participante, de forma direta ou indireta, recebimento por palestras, aulas, atuação como proctor de treinamentos, remunerações, honorários pagos por participações em conselhos consultivos, de investigadores, ou outros comitês, etc. Provenientes da indústria farmacêutica, de órteses, próteses, equipamentos e implantes, brasileiras ou estrangeiras: - Novo Nordisk: semaglutida; Novartis: inclisirana; Daiichi Sankyo: ácido bempedoico. Outros relacionamentos Financiamento de atividades de educação médica continuada, incluindo viagens, hospedagens e inscrições para congressos e cursos, provenientes da indústria farmacêutica, de órteses, próteses, equipamentos e implantes, brasileiras ou estrangeiras: - Novo Nordisk: semaglutida; Novartis: inclisirana; Daiichi Sankyo: ácido bempedoico.
Fabio Biscegli Jatene	Nada a ser declarado
Gentil Barreira de Aguiar Filho	Nada a ser declarado
Gilson Soares Feitosa-Filho	Declaração financeira B - Financiamento de pesquisas sob sua responsabilidade direta/pessoal (direcionado ao departamento ou instituição) provenientes da indústria farmacêutica, de órteses, próteses, equipamentos e implantes, brasileiras ou estrangeiras: - Amgen: Olpasiran; Idorsia: Selatogrel; Anthos: Abelacimabe; Jansen e Bayer: Milvexiana. Outros relacionamentos Financiamento de atividades de educação médica continuada, incluindo viagens, hospedagens e inscrições para congressos e cursos, provenientes da indústria farmacêutica, de órteses, próteses, equipamentos e implantes, brasileiras ou estrangeiras: - Servier/Trimetazidina: Inscrição em Congresso.
Henrique Patrus Mundim Pena	Declaração financeira A - Pagamento de qualquer espécie e desde que economicamente apreciáveis, feitos a (i) você, (ii) ao seu cônjuge/ companheiro ou a qualquer outro membro que resida com você, (iii) a qualquer pessoa jurídica em que qualquer destes seja controlador, sócio, acionista ou participante, de forma direta ou indireta, recebimento por palestras, aulas, atuação como proctor de treinamentos, remunerações, honorários pagos por participações em conselhos consultivos, de investigadores, ou outros comitês, etc. Provenientes da indústria farmacêutica, de órteses, próteses, equipamentos e implantes, brasileiras ou estrangeiras: - Astrazeneca: insuficiência cardíaca/ dapaglizina/ hipercalemia; Viatris: insuficiência cardíaca/ eplerenone. Outros relacionamentos Participação societária de qualquer natureza e qualquer valor economicamente apreciável de empresas na área de saúde, de ensino ou em empresas concorrentes ou fornecedoras da SBC: - Novo Nordisk: obesidade, cardiometabolismo.
Ibraim Masciarelli Francisco Pinto	Nada a ser declarado
Iran Castro	Nada a ser declarado
Jaqueline R Scholz	Nada a ser declarado
João Carlos Ferreira Leal	Declaração financeira A - Pagamento de qualquer espécie e desde que economicamente apreciáveis, feitos a (i) você, (ii) ao seu cônjuge/ companheiro ou a qualquer outro membro que resida com você, (iii) a qualquer pessoa jurídica em que qualquer destes seja controlador, sócio, acionista ou participante, de forma direta ou indireta, recebimento por palestras, aulas, atuação como proctor de treinamentos, remunerações, honorários pagos por participações em conselhos consultivos, de investigadores, ou outros comitês, etc. Provenientes da indústria farmacêutica, de órteses, próteses, equipamentos e implantes, brasileiras ou estrangeiras: - Braile Biomédica: proctor e palestras; Neomex: proctor e palestras. B - Financiamento de pesquisas sob sua responsabilidade direta/pessoal (direcionado ao departamento ou instituição) provenientes da indústria farmacêutica, de órteses, próteses, equipamentos e implantes, brasileiras ou estrangeiras: - Braile Biomédica: prótese biológica. Outros relacionamentos Financiamento de atividades de educação médica continuada, incluindo viagens, hospedagens e inscrições para congressos e cursos, provenientes da indústria farmacêutica, de órteses, próteses, equipamentos e implantes, brasileiras ou estrangeiras: - Braile Biomédica: palestras.
João Fernando Monteiro Ferreira	Nada a ser declarado
José Armando Mangione	Declaração financeira A - Pagamento de qualquer espécie e desde que economicamente apreciáveis, feitos a (i) você, (ii) ao seu cônjuge/ companheiro ou a qualquer outro membro que resida com você, (iii) a qualquer pessoa jurídica em que qualquer destes seja controlador, sócio, acionista ou participante, de forma direta ou indireta, recebimento por palestras, aulas, atuação como proctor de treinamentos, remunerações, honorários pagos por participações em conselhos consultivos, de investigadores, ou outros comitês, etc. Provenientes da indústria farmacêutica, de órteses, próteses, equipamentos e implantes, brasileiras ou estrangeiras: - Edwards Lifecience: proctor TAVI; Medtronic: proctor TAVI.
José Jayme Galvão de Lima	Nada a ser declarado
José Rocha Faria Neto	Declaração financeira A - Pagamento de qualquer espécie e desde que economicamente apreciáveis, feitos a (i) você, (ii) ao seu cônjuge/ companheiro ou a qualquer outro membro que resida com você, (iii) a qualquer pessoa jurídica em que qualquer destes seja controlador, sócio, acionista ou participante, de forma direta ou indireta, recebimento por palestras, aulas, atuação como proctor de treinamentos, remunerações, honorários pagos por participações em conselhos consultivos, de investigadores, ou outros comitês, etc. Provenientes da indústria farmacêutica, de órteses, próteses, equipamentos e implantes, brasileiras ou estrangeiras: - Aché: DAC e dislipidemia; Daiichi Sankyo: DAC e dislipidemia; Libbs: DAC e dislipidemia; Novartis: dislipidemia; AstraZeneca: diabetes; Lilly: diabetes e obesidade; Novo Nordisk: diabetes e obesidade; Sanofi e Medley: dislipidemia; Bayer: risco cardiovascular. Outros relacionamentos Financiamento de atividades de educação médica continuada, incluindo viagens, hospedagens e inscrições para congressos e cursos, provenientes da indústria farmacêutica, de órteses, próteses, equipamentos e implantes, brasileiras ou estrangeiras: - Novo Nordisk; Bayer; Daiichi Sankyo; AstraZeneca.
José Soares Junior	Nada a ser declarado
Kleisson Antônio Pontes Maia	Declaração financeira A - Pagamento de qualquer espécie e desde que economicamente apreciáveis, feitos a (i) você, (ii) ao seu cônjuge/ companheiro ou a qualquer outro membro que resida com você, (iii) a qualquer pessoa jurídica em que qualquer destes seja controlador, sócio, acionista ou participante, de forma direta ou indireta, recebimento por palestras, aulas, atuação como proctor de treinamentos, remunerações, honorários pagos por participações em conselhos consultivos, de investigadores, ou outros comitês, etc. Provenientes da indústria farmacêutica, de órteses, próteses, equipamentos e implantes, brasileiras ou estrangeiras: - Novartis: hipercolesterolemia; GSK: vacinas; Biolab: hipertensão, Lilly: diabetes, obesidade; Novo Nordisk: diabetes, obesidade; Servier: doença coronária. B - Financiamento de pesquisas sob sua responsabilidade direta/pessoal (direcionado ao departamento ou instituição) provenientes da indústria farmacêutica, de órteses, próteses, equipamentos e implantes, brasileiras ou estrangeiras: - Lilly: Lp(a). Outros relacionamentos Financiamento de atividades de educação médica continuada, incluindo viagens, hospedagens e inscrições para congressos e cursos, provenientes da indústria farmacêutica, de órteses, próteses, equipamentos e implantes, brasileiras ou estrangeiras: - Servier: doença coronária; Lilly: diabetes; Novo Nordisk: diabetes, obesidade; Viatris: hipercolesterolemia.
Lara Cristiane Terra Ferreira Carreira	Nada a ser declarado
Luciana Diniz Nagem Janot de Matos	Nada a ser declarado
Luciana Oliveira Cascaes Dourado	Nada a ser declarado
Luhanda Leonora Cardoso Monti Sousa	Outros relacionamentos Financiamento de atividades de educação médica continuada, incluindo viagens, hospedagens e inscrições para congressos e cursos, provenientes da indústria farmacêutica, de órteses, próteses, equipamentos e implantes, brasileiras ou estrangeiras: - Novo Nordisk: Semaglutida.
Luis Alberto Oliveira Dallan	Nada a ser declarado
Luís Henrique Wolff Gowdak	Declaração financeira A - Pagamento de qualquer espécie e desde que economicamente apreciáveis, feitos a (i) você, (ii) ao seu cônjuge/ companheiro ou a qualquer outro membro que resida com você, (iii) a qualquer pessoa jurídica em que qualquer destes seja controlador, sócio, acionista ou participante, de forma direta ou indireta, recebimento por palestras, aulas, atuação como proctor de treinamentos, remunerações, honorários pagos por participações em conselhos consultivos, de investigadores, ou outros comitês, etc. Provenientes da indústria farmacêutica, de órteses, próteses, equipamentos e implantes, brasileiras ou estrangeiras: - Servier: Angina; Novartis: hipercolesterolemia; GSK: vacinação. B - Financiamento de pesquisas sob sua responsabilidade direta/pessoal (direcionado ao departamento ou instituição) provenientes da indústria farmacêutica, de órteses, próteses, equipamentos e implantes, brasileiras ou estrangeiras: - Servier: Angina. Outros relacionamentos Financiamento de atividades de educação médica continuada, incluindo viagens, hospedagens e inscrições para congressos e cursos, provenientes da indústria farmacêutica, de órteses, próteses, equipamentos e implantes, brasileiras ou estrangeiras: - Novo Nordisk: obesidade e diabetes; Lilly: obesidade.
Luiz Antonio Machado Cesar	Declaração financeira A - Pagamento de qualquer espécie e desde que economicamente apreciáveis, feitos a (i) você, (ii) ao seu cônjuge/ companheiro ou a qualquer outro membro que resida com você, (iii) a qualquer pessoa jurídica em que qualquer destes seja controlador, sócio, acionista ou participante, de forma direta ou indireta, recebimento por palestras, aulas, atuação como proctor de treinamentos, remunerações, honorários pagos por participações em conselhos consultivos, de investigadores, ou outros comitês, etc. Provenientes da indústria farmacêutica, de órteses, próteses, equipamentos e implantes, brasileiras ou estrangeiras: - SERVIER: Vastarel/ angina. Outros relacionamentos Financiamento de atividades de educação médica continuada, incluindo viagens, hospedagens e inscrições para congressos e cursos, provenientes da indústria farmacêutica, de órteses, próteses, equipamentos e implantes, brasileiras ou estrangeiras: - EMS: Vynaxa/ anticoagulação.
Luiz Eduardo Mastrocola	Nada a ser declarado
Marcia Maria Godoy Gowdak	Nada a ser declarado
Nilson Tavares Poppi	Declaração financeira A - Pagamento de qualquer espécie e desde que economicamente apreciáveis, feitos a (i) você, (ii) ao seu cônjuge/ companheiro ou a qualquer outro membro que resida com você, (iii) a qualquer pessoa jurídica em que qualquer destes seja controlador, sócio, acionista ou participante, de forma direta ou indireta, recebimento por palestras, aulas, atuação como proctor de treinamentos, remunerações, honorários pagos por participações em conselhos consultivos, de investigadores, ou outros comitês, etc. Provenientes da indústria farmacêutica, de órteses, próteses, equipamentos e implantes, brasileiras ou estrangeiras: - EMS: doença coronária; Boehringer-Ingelheim: anticoagulação; Servier: doença coronária. Outros relacionamentos Financiamento de atividades de educação médica continuada, incluindo viagens, hospedagens e inscrições para congressos e cursos, provenientes da indústria farmacêutica, de órteses, próteses, equipamentos e implantes, brasileiras ou estrangeiras: - Servier: doença coronária.
Olimpio Ribeiro França Neto	Declaração financeira A - Pagamento de qualquer espécie e desde que economicamente apreciáveis, feitos a (i) você, (ii) ao seu cônjuge/ companheiro ou a qualquer outro membro que resida com você, (iii) a qualquer pessoa jurídica em que qualquer destes seja controlador, sócio, acionista ou participante, de forma direta ou indireta, recebimento por palestras, aulas, atuação como proctor de treinamentos, remunerações, honorários pagos por participações em conselhos consultivos, de investigadores, ou outros comitês, etc. Provenientes da indústria farmacêutica, de órteses, próteses, equipamentos e implantes, brasileiras ou estrangeiras: - Servier: angina; Biolab: hipertensão. B - Financiamento de pesquisas sob sua responsabilidade direta/pessoal (direcionado ao departamento ou instituição) provenientes da indústria farmacêutica, de órteses, próteses, equipamentos e implantes, brasileiras ou estrangeiras: - Servier: angina. Outros relacionamentos Financiamento de atividades de educação médica continuada, incluindo viagens, hospedagens e inscrições para congressos e cursos, provenientes da indústria farmacêutica, de órteses, próteses, equipamentos e implantes, brasileiras ou estrangeiras: - Servier: angina; Biolab: hipertensão..
Otavio Rizzi Coelho	Declaração financeira A - Pagamento de qualquer espécie e desde que economicamente apreciáveis, feitos a (i) você, (ii) ao seu cônjuge/ companheiro ou a qualquer outro membro que resida com você, (iii) a qualquer pessoa jurídica em que qualquer destes seja controlador, sócio, acionista ou participante, de forma direta ou indireta, recebimento por palestras, aulas, atuação como proctor de treinamentos, remunerações, honorários pagos por participações em conselhos consultivos, de investigadores, ou outros comitês, etc. Provenientes da indústria farmacêutica, de órteses, próteses, equipamentos e implantes, brasileiras ou estrangeiras: - Novartis: Inclisiran; Novo Nordisk: Semaglutida; AstraZeneca: Dapaglifozina; Daiichi Sankyo: Ticagrelor; Bayer: Fenirinone. Outros relacionamentos Financiamento de atividades de educação médica continuada, incluindo viagens, hospedagens e inscrições para congressos e cursos, provenientes da indústria farmacêutica, de órteses, próteses, equipamentos e implantes, brasileiras ou estrangeiras: - AstraZeneca: Dapaglifozina; Novo Nordisk: Semaglutida.
Otávio R. Coelho-Filho	Declaração financeira A - Pagamento de qualquer espécie e desde que economicamente apreciáveis, feitos a (i) você, (ii) ao seu cônjuge/companheiro ou a qualquer outro membro que resida com você, (iii) a qualquer pessoa jurídica em que qualquer destes seja controlador, sócio, acionista ou participante, de forma direta ou indireta, recebimento por palestras, aulas, atuação como proctor de treinamentos, remunerações, honorários pagos por participações em conselhos consultivos, de investigadores, ou outros comitês, etc. Provenientes da indústria farmacêutica, de órteses, próteses, equipamentos e implantes, brasileiras ou estrangeiras: - Pfizer: amiloidose/Tafamides; AstraZeneca: insuficiência cardíaca/Forxiga; Bayer: Firialta; Norvartis: insuficiência cardíaca/dislipidemia; EMS: insuficiência cardíaca. Outros relacionamentos Financiamento de atividades de educação médica continuada, incluindo viagens, hospedagens e inscrições para congressos e cursos, provenientes da indústria farmacêutica, de órteses, próteses, equipamentos e implantes, brasileiras ou estrangeiras: - Bayer; Pfizer; AstraZeneca.
Paulo Eduardo Ballvé Behr	Declaração financeira A - Pagamento de qualquer espécie e desde que economicamente apreciáveis, feitos a (i) você, (ii) ao seu cônjuge/ companheiro ou a qualquer outro membro que resida com você, (iii) a qualquer pessoa jurídica em que qualquer destes seja controlador, sócio, acionista ou participante, de forma direta ou indireta, recebimento por palestras, aulas, atuação como proctor de treinamentos, remunerações, honorários pagos por participações em conselhos consultivos, de investigadores, ou outros comitês, etc. Provenientes da indústria farmacêutica, de órteses, próteses, equipamentos e implantes, brasileiras ou estrangeiras: - Novartis: Sybrava; Novartis: Cosentyx; Biolab: Livalo; Aché: Trezete; PTC: Volanesorsena; Libbs: Zinpass; Novo Nordisk: Ozempic; Daiichi Sankyo: Nustendi; Amgen: Olpasiran. Outros relacionamentos Financiamento de atividades de educação médica continuada, incluindo viagens, hospedagens e inscrições para congressos e cursos, provenientes da indústria farmacêutica, de órteses, próteses, equipamentos e implantes, brasileiras ou estrangeiras: - Novartis: Sybrava; Daiichi: Nustendi; Novo Nordisk: Ozempic.
Paulo Ricardo Avancini Caramori	Declaração financeira B - Financiamento de pesquisas sob sua responsabilidade direta/pessoal (direcionado ao departamento ou instituição) provenientes da indústria farmacêutica, de órteses, próteses, equipamentos e implantes, brasileiras ou estrangeiras: - Palestrante: Novo Nordisk, Viatris, Abbott, Boston Scientific, Meril Life Sciences.
Pedro Alves Lemos Neto	Declaração financeira A - Pagamento de qualquer espécie e desde que economicamente apreciáveis, feitos a (i) você, (ii) ao seu cônjuge/ companheiro ou a qualquer outro membro que resida com você, (iii) a qualquer pessoa jurídica em que qualquer destes seja controlador, sócio, acionista ou participante, de forma direta ou indireta, recebimento por palestras, aulas, atuação como proctor de treinamentos, remunerações, honorários pagos por participações em conselhos consultivos, de investigadores, ou outros comitês, etc. Provenientes da indústria farmacêutica, de órteses, próteses, equipamentos e implantes, brasileiras ou estrangeiras: - Nordisk: inflamação; Abbott Vascular: stent; Daiichi Sankyo: antiplaquetário; Edwards Lifesciences: TAVI; B. Braun Melsungen AG: balão farmacológico; Boston Scientific: oclusão de apêndice atrial esquerdo. B - Financiamento de pesquisas sob sua responsabilidade direta/pessoal (direcionado ao departamento ou instituição) provenientes da indústria farmacêutica, de órteses, próteses, equipamentos e implantes, brasileiras ou estrangeiras: - Nordisk: inflamação.
Pedro Silvio Farsky	Outros relacionamentos Financiamento de atividades de educação médica continuada, incluindo viagens, hospedagens e inscrições para congressos e cursos, provenientes da indústria farmacêutica, de órteses, próteses, equipamentos e implantes, brasileiras ou estrangeiras: - Novo Nordisk: Semaglutida; Lilly: Tirzepatida; Novartis: Inclisirana.
Raul Dias dos Santos Filho	Declaração financeira A - Pagamento de qualquer espécie e desde que economicamente apreciáveis, feitos a (i) você, (ii) ao seu cônjuge/ companheiro ou a qualquer outro membro que resida com você, (iii) a qualquer pessoa jurídica em que qualquer destes seja controlador, sócio, acionista ou participante, de forma direta ou indireta, recebimento por palestras, aulas, atuação como proctor de treinamentos, remunerações, honorários pagos por participações em conselhos consultivos, de investigadores, ou outros comitês, etc. Provenientes da indústria farmacêutica, de órteses, próteses, equipamentos e implantes, brasileiras ou estrangeiras: - Amgen; Novartis;, Arrowhead; Ionis; Torrent, Sanofi; Daiichi Sankyo;Aché: hipolipemiantes; Novo Nordisk, Eli-Lilly: hipoglicemiantes. B - Financiamento de pesquisas sob sua responsabilidade direta/pessoal (direcionado ao departamento ou instituição) provenientes da indústria farmacêutica, de órteses, próteses, equipamentos e implantes, brasileiras ou estrangeiras: - Amgen; Arrowhead, Ionis; Eli-Lilly: hipolipemiantes.
Renato D. Lopes	Declaração financeira A - Pagamento de qualquer espécie e desde que economicamente apreciáveis, feitos a (i) você, (ii) ao seu cônjuge/ companheiro ou a qualquer outro membro que resida com você, (iii) a qualquer pessoa jurídica em que qualquer destes seja controlador, sócio, acionista ou participante, de forma direta ou indireta, recebimento por palestras, aulas, atuação como proctor de treinamentos, remunerações, honorários pagos por participações em conselhos consultivos, de investigadores, ou outros comitês, etc. Provenientes da indústria farmacêutica, de órteses, próteses, equipamentos e implantes, brasileiras ou estrangeiras: - Pfizer, Daiichi Sankyo, Novo Nordisk, Bayer, Boehringer Ingelheim, Bristol-Myers Squibb. B - Financiamento de pesquisas sob sua responsabilidade direta/pessoal (direcionado ao departamento ou instituição) provenientes da indústria farmacêutica, de órteses, próteses, equipamentos e implantes, brasileiras ou estrangeiras: - Amgen, Bristol-Myers Squibb, GlaxoSmithKline, Medtronic, Pfizer, Sanofi-Aventis. Outros relacionamentos Financiamento de atividades de educação médica continuada, incluindo viagens, hospedagens e inscrições para congressos e cursos, provenientes da indústria farmacêutica, de órteses, próteses, equipamentos e implantes, brasileiras ou estrangeiras: - Pfizer, Daiichi Sankyo, Novo Nordisk, Novartis.
Ricardo Pavanello	Nada a ser declarado
Salvador Manoel Serra	Nada a ser declarado
Sarah Fagundes Grobe	Declaração financeira A - Pagamento de qualquer espécie e desde que economicamente apreciáveis, feitos a (i) você, (ii) ao seu cônjuge/ companheiro ou a qualquer outro membro que resida com você, (iii) a qualquer pessoa jurídica em que qualquer destes seja controlador, sócio, acionista ou participante, de forma direta ou indireta, recebimento por palestras, aulas, atuação como proctor de treinamentos, remunerações, honorários pagos por participações em conselhos consultivos, de investigadores, ou outros comitês, etc. Provenientes da indústria farmacêutica, de órteses, próteses, equipamentos e implantes, brasileiras ou estrangeiras: - Biolab: Doble; Novartis: Sybrava; Ache: dislipidemia; Servier: Vastarel; Mantecorp: Saúde da mulher; Daiichi Sankyo: Nustenti. B - Financiamento de pesquisas sob sua responsabilidade direta/pessoal (direcionado ao departamento ou instituição) provenientes da indústria farmacêutica, de órteses, próteses, equipamentos e implantes, brasileiras ou estrangeiras: - Servier: Vastarel; Daiichi Sankyo: Nustendi. Outros relacionamentos Financiamento de atividades de educação médica continuada, incluindo viagens, hospedagens e inscrições para congressos e cursos, provenientes da indústria farmacêutica, de órteses, próteses, equipamentos e implantes, brasileiras ou estrangeiras: - Servier; Novartis.
Sérgio Tavares Montenegro	Declaração financeira A - Pagamento de qualquer espécie e desde que economicamente apreciáveis, feitos a (i) você, (ii) ao seu cônjuge/ companheiro ou a qualquer outro membro que resida com você, (iii) a qualquer pessoa jurídica em que qualquer destes seja controlador, sócio, acionista ou participante, de forma direta ou indireta, recebimento por palestras, aulas, atuação como proctor de treinamentos, remunerações, honorários pagos por participações em conselhos consultivos, de investigadores, ou outros comitês, etc. Provenientes da indústria farmacêutica, de órteses, próteses, equipamentos e implantes, brasileiras ou estrangeiras: - Servier: Ticagrelor; Merck: Bisoprolol. Outros relacionamentos Financiamento de atividades de educação médica continuada, incluindo viagens, hospedagens e inscrições para congressos e cursos, provenientes da indústria farmacêutica, de órteses, próteses, equipamentos e implantes, brasileiras ou estrangeiras: - Servier.
Silvio Henrique Barberato	Declaração financeira A - Pagamento de qualquer espécie e desde que economicamente apreciáveis, feitos a (i) você, (ii) ao seu cônjuge/ companheiro ou a qualquer outro membro que resida com você, (iii) a qualquer pessoa jurídica em que qualquer destes seja controlador, sócio, acionista ou participante, de forma direta ou indireta, recebimento por palestras, aulas, atuação como proctor de treinamentos, remunerações, honorários pagos por participações em conselhos consultivos, de investigadores, ou outros comitês, etc. Provenientes da indústria farmacêutica, de órteses, próteses, equipamentos e implantes, brasileiras ou estrangeiras: - Pfizer: amiloidose; Bristol: Camzyos. Outros relacionamentos Financiamento de atividades de educação médica continuada, incluindo viagens, hospedagens e inscrições para congressos e cursos, provenientes da indústria farmacêutica, de órteses, próteses, equipamentos e implantes, brasileiras ou estrangeiras: - Pfizer: amiloidose.
Tania Mara Varejão Strabelli	Nada a ser declarado
Ursula Maria Moreira Costa Burgos	Nada a ser declarado
Vinicius José da Silva Nina	Nada a ser declarado
Walter Jose Gomes	Nada a ser declarado
William Azem Chalela	Nada a ser declarado
Wilson Mathias Junior	Nada a ser declarado

**Sumário**
**1. Introdução** 14
**2. Diagnóstico** 16
  **2.1. Avaliação Clínica** 16
    **2.1.1. Definição de Angina** 16
    **2.1.2. Avaliação Clínica dos Pacientes com Dor Torácica** 17
      ***2.1.2.1. História Clínica*** 17
      ***2.1.2.2. Exame Físico*** 18
      ***2.1.2.3. Diagnóstico Diferencial da Dor Torácica*** 18
    **2.1.3. Abordagem Laboratorial** 19
  **2.2. Testes Não Invasivos** 21
    **2.2.1. Teste Ergométrico** 21
    **2.2.2. Ecocardiografia** 22
      ***2.2.2.1. Ecocardiografia Transtorácica*** 22
      ***2.2.2.2. Ecocardiografia sob Estresse*** 23
    **2.2.3. Medicina Nuclear** 23
      ***2.2.3.1. Decisão Clínica após Estudos de Perfusão com Radionuclídeos – Impacto da Quantidade de Isquemia nos Desfechos Cardiovasculares*** 25
      ***2.2.3.2. Tomografia por Emissão de Pósitrons (PET)*** 26
      ***2.2.3.3. Reserva Coronariana com SPECT CZT*** 30
    **2.2.4. Abordagem Laboratorial** 30
    **2.2.5. Tomografia de Coronárias** 30
      ***2.2.5.1. Tomografia de Coronárias para Determinar o Escore de Cálcio Coronário*** 30
      ***2.2.5.2. Angiotomografia Coronária*** 31
    **2.2.6. Ressonância Magnética Cardiovascular** 32
      ***2.2.6.1. Avaliação da Isquemia Miocárdica pela RMC*** 32
      ***2.2.6.2. Avaliação da Contratilidade Segmentar/Reserva Contrátil pela RMC*** 33
      ***2.2.6.3. Pesquisa de Doença Arterial Coronariana pela Ressonância Magnética – Viabilidade Miocárdica*** 33
      ***2.2.6.4. Aplicando a RMC para Pesquisa de Viabilidade na DAC*** 34
      ***2.2.6.5. Grau de Recomendação e Nível de Evidência*** 34
    **2.2.7. Cateterismo Cardíaco** 34
**3. Estratégias Clínicas para Avaliar o Risco Cardiovascular e a Estratificação da Doença Aterosclerótica Coronariana** 36
  **3.1. Avaliação Inicial do Risco** 36
    **3.1.1. Marcadores Laboratoriais de Risco na Doença Coronariana Crônica** 36
    **3.1.2. TE para Avaliação do Prognóstico da DAC Estável** 36
    **3.1.3. Ecocardiografia Transtorácica (Repouso e sob Estresse)** 36
    **3.1.4. Cintilografia de Perfusão Miocárdica** 37
    **3.1.5. Ressonância Magnética Cardíaca** 37
    **3.1.6. Angiotomografia de Coronárias** 37
    **3.1.7. Resumo com Sugestão de como Investigar com Métodos Diagnósticos** 38
**4. Tratamento Clínico-Farmacológico para Redução do Risco de Eventos** 39
  **4.1. Terapia Antitrombótica nas Síndromes Coronarianas Crônicas** 39
    **4.1.1. Aspirina** 39
    **4.1.2. Inibidores do Receptor P2Y12** 39
    **4.1.3. Dupla Terapia Antiplaquetária** 39
    **4.1.4. Anticoagulação em Dose Baixa** 40
    **4.1.5. Anticoagulação a Longo Prazo** 41
    **4.1.6. Antiplaquetários no Contexto da Intervenção Coronariana Percutânea (ICP)** 41
    **4.1.7. Anticoagulantes no Contexto da Intervenção Coronariana Percutânea (ICP)** 41
  **4.2. Manejo Lipídico na Síndrome Coronária Crônica** 41
    **4.2.1. Estatinas e Outras Drogas que Atuam no LDL-C** 41
    **4.2.2. Lipoproteína(a)** 42
    **4.2.3. Lipoproteínas Ricas em Triglicerídeos** 44
  **4.3. Terapia de Reposição Hormonal** 44
  **4.4. Bloqueio do Sistema Renina-Angiotensina-Aldosterona** 44
    **4.4.1. Inibidores da Neprilisina e do Receptor da Angiotensina** 45
    **4.4.2. Bloqueadores da Aldosterona** 46
  **4.5. Colchicina** 46
  **4.6. Terapia Antidiabética** 47
**5. Tratamento Clínico-Farmacológico para Controle Ótimo de Sintomas** 48
  **5.1. Betabloqueadores** 48
  **5.2. Trimetazidina** 49
  **5.3. Antagonistas dos Canais de Cálcio** 49
  **5.4. Verapamil** 50
  **5.5. Diltiazem** 50
  **5.6. Di-hidropiridínicos** 50
  **5.7. Ivabradina** 50
  **5.8. Ranolazina** 50
  **5.9. Nitratos de Ação Curta e Prolongada** 51
  **5.10. Alopurinol** 51
  **5.11. Individualização do Tratamento Antianginoso** 52
**6. Tratamento com Medidas Invasivas** 54
  **6.1. Tratamento Percutâneo para Controle de Sintomas e Redução do Risco de Eventos** 54
    **6.1.1. Escores de Complexidade Angiográfica na Tomada de Decisão** 54
    **6.1.2. Recomendação** 56
  **6.2. Avaliação Funcional Invasiva** 56
    **6.2.1. Avaliação de Estenoses Coronárias Epicárdicas** 56
    **6.2.2. Recomendação** 56
    **6.2.3. Angina Microvascular** 57
    **6.2.4. Recomendação** 57
  **6.3. Métodos de Imagem Intravascular** 57
    **6.3.1. Uso de Imagens Intravasculares no Diagnóstico e na Avaliação de Estenoses Coronárias** 57
    **6.3.2. Recomendação** 57
    **6.3.3. Uso de Imagens Intravasculares para Guiar Intervenção Coronária Percutânea** 58
    **6.3.4. Recomendação** 58
  **6.4. Intervenção Coronariana Percutânea como Estratégia para Melhora Prognóstica** 59
    **6.4.1. Intervenção Coronariana Percutânea como Estratégia para Alívio de Sintomas** 59
  **6.5. Escores de Risco de Sangramento na Tomada de Decisão** 60
    **6.5.1. Recomendação** 61
  **6.6. Preparo em Situações Específicas (Pacientes com Disfunção Renal ou Alérgicos ao Contraste)** 62
    **6.6.1. Disfunção Renal** 62
    **6.6.2. Medidas de Proteção Renal** 62
    **6.6.3. Medidas Gerais** 62
    **6.6.4. Administração de Fluídos** 63
    **6.6.5. Prevenção Farmacológica** 63
    **6.6.6. Meios de Contraste** 63
    **6.6.7. Alergia aos Meios de Contraste** 63
    **6.6.8. Conclusões** 63
  **6.7. Tratamento Cirúrgico para Controle de Sintomas e Redução do Risco de Eventos** 63
    **6.7.1. Pacientes com Doença de Múltiplos Vasos Coronarianos e Escore SYNTAX > 23** 64
    **6.7.2. Pacientes com DAC Avançada E Diabetes Mellitus** 65
    **6.7.3. Pacientes com Disfunção Ventricular Esquerda** 66
    **6.7.4. Pacientes com Lesão de Tronco de Artéria Coronária Esquerda** 66
    **6.7.5. Alívio do Sintoma Anginoso e Qualidade de Vida** 67
**7. Vacinação Contra Pneumonia, Influenza e COVID-19** 68
  **7.1. Vacina para Influenza** 68
  **7.2. Efeitos e Eventos Adversos** 68
  **7.3. Vacina para Pneumococo** 70
  **7.4. Observações para Esquema Sequencial VPC13 e VPP23** 70
  **7.5. Vacina para COVID-19** 70
  **7.6. Vacinas Recomendadas** 70
  **7.7. Risco de Miocardite/Pericardite após Vacinação da COVID-19** 70
**8. Dieta e Álcool/Controle de Peso** 71
  **8.1. Controle de Peso** 71
**9. Atividade Física, Reabilitação Cardíaca e Atividade Sexual** 72
  **9.1 Mudanças no Estilo de Vida e Outros Fatores** 72
    **9.1.1. Sedentarismo, Atividade Física e Exercício Físico** 72
    **9.1.2. Prevenção Secundária (Reabilitação Cardíaca Baseada em Exercício)** 72
    **9.1.3. Reabilitação Domiciliar** 75
    **9.1.4. Atividade Sexual** 75
**10. Tabagismo e Poluição Ambiental** 76
**11. Fatores Psicossociais e Adesão ao Tratamento** 78
  **11.1. Fatores Psicossociais** 78
  **11.2. Adesão ao Tratamento** 78
**12. Angina e Isquemia sem Obstrução das Artérias Coronárias (INOCA, ANOCA)** 78
  **12.1. Características Gerais da Doença Isquêmica do Coração Feminino** 79
    **12.1.1. Doença Aterosclerótica Coronária** 79
    **12.1.2. ANOCA e INOCA (isquemia sem Obstrução das Artérias Coronárias)** 79
    **12.1.3. MINOCA (Infarto do Miocárdio sem Obstrução das Artérias Coronárias)** 79
    **12.1.4. Doença Microvascular (DMV)** 79
    **12.1.5. Dissecção Espontânea de Artéria Coronária (DEAC)** 79
    **12.1.6. Vasoespasmo Coronariano (VPC)** 80
    **12.1.7. Trombose/Embolia Coronária** 80
**13. Doença Isquêmica na Mulher – Aspectos Específicos do Sexo Feminino** 80
  **13.1. Apresentação Clínica e Diagnóstico** 80
  **13.2. Tratamento e Manejo** 80
  **13.3. Fatores Prognósticos e Desfechos** 81
  **13.4. Influência Hormonal e Fatores Genéticos** 81
**14. Angina Refratária** 81
**15. Viabilidade Miocárdica** 81
  **15.1. Viabilidade Miocárdica** 81
  **15.2. Estudos Observacionais de Viabilidade** 83
  **15.3. Viabilidade e Melhora da Função Ventricular** 83
  **15.4. Estudos Contemporâneos: STICH e REVIVED–BCIS2** 83
  **15.5. Terapia Farmacológica na DAC e ICfer** 84
  **15.6. Resumo** 84
**16. Doença Renal Crônica na SCC** 85
  **16.1. Estratificação por Testes Não Invasivos** 85
  **16.2. Estratificação por Testes Invasivos** 86
  **16.3. Crítica à Estratégia Invasiva de Investigação da DAC** 86
  **16.4. Medicação e Ajuste de Dose em Pacientes com DCV e DRC** 87
**17. Aspectos Cardio-Oncológicos na SCC** 88
**18. Tratamento da Hipertensão Arterial no Portador de Doença Arterial Coronariana Crônica** 90
**19. Aspectos do Tratamento na Presença da Fibrilação Atrial** 90
  **19.1. Novos Anticoagulantes Orais Associados ao Ácido Acetilsalicílico** 90
  **19.2. Anticoagulação em Indivíduos com Síndromes Coronarianas Crônicas e Fibrilação Atrial** 91
**20. Prevenção Secundária PÓS-SCA** 91
  **20.1. Prioridades no Período Pós-Alta por SCA** 92
  **20.2. Estratificação de Risco Pós-SCA para Planejamento da Prevenção Secundária** 92
  **20.3. Variáveis de Risco para Insuficiência Cardíaca e Eventos Trombóticos** 92
  **20.4. Estratégias para Pacientes de Baixo Risco** 92
  **20.5. Seguimento Clínico** 93
**21. Cuidados Pós-Intervenção Coronária Percutânea na SCC** 93
  **21.1. Antiagregação Plaquetária após ICP com Stent em Paciente sem Indicação de Uso de Anticoagulante** 93
  **21.2. Antiagregação Plaquetária após ICP com Stent em Paciente com Indicação de Uso de Anticoagulação** 93
**22. Pós-Operatório de Cirurgia de Revascularização Miocárdica – Cuidados e Controles De Rotina** 96
**Referências** 98

## 1. Introdução

Uma das principais atividades da Sociedade Brasileira de Cardiologia (SBC) consiste na elaboração de diretrizes para diversas doenças cardíacas, utilizando a produção científica mais recente e baseando-se em evidências clínicas robustas. O objetivo é orientar os profissionais de saúde quanto à prevenção, diagnóstico e tratamento das doenças cardíacas. Esta diretriz para a síndrome coronária crônica (SCC) foi desenvolvida por membros voluntários da SBC, sem apoio comercial, priorizando aspectos éticos na orientação clínico-cardiológica dos diversos elementos e participantes envolvidos na diretriz.

As doenças cardíacas são as principais causas de morte na população brasileira, e a SCC destaca-se como a mais significativa em termos de morbidade e mortalidade das doenças não transmissíveis. Portanto, esta diretriz é de extrema importância para auxiliar os profissionais de saúde, especialmente os cardiologistas, nas recomendações para as diversas situações clínicas encontradas nos pacientes com SCC.

A SCC abrange uma variedade de perfis de pacientes, analisados em detalhes nesta diretriz. Esse conceito surgiu em 2019, durante o Congresso da Sociedade Europeia de Cardiologia (European Society of Cardiology [ESC]), para que pudéssemos entender a doença coronariana como um contínuo, como evolução, na maioria das vezes, silenciosa por anos e com momentos de agudização. Em cada momento desses, com estratégias diferentes de condutas tanto do uso de métodos diagnósticos quanto das terapêuticas de orientação geral de estilo de vida, os reforços ao longo dessa jornada, as necessidades de mudanças na terapêutica medicamentosa e as indicações de procedimentos de revascularização. Bem como para relembrar o quanto a ocorrência de um evento agudo muda a curva de eventos futuros para esses indivíduos (Figura Central). Ao mesmo tempo, o conceito de SCC traz, de forma importante, o fato de muitos pacientes, especialmente mulheres, terem quadros clínicos de angina e isquemia na ausência de obstruções coronárias, muito embora, na maioria das vezes, na presença de aterosclerose.

**Figure:**
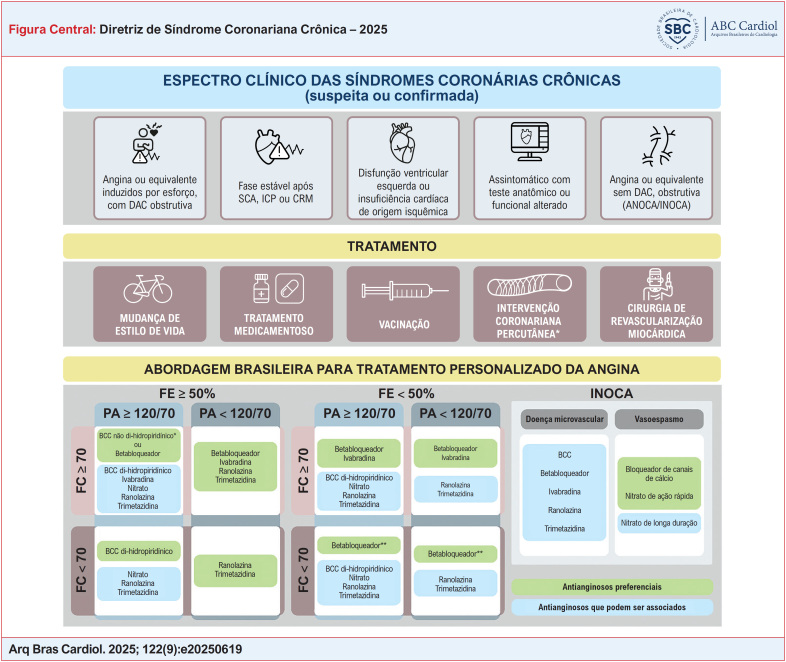


Assim, para resumir, a Tabela 1 ressalta os pacientes por situações clínicas com ou sem aterosclerose, podendo, quando presente, ter ou não obstruções na árvore coronariana.

**Tabela 1:** Características de apresentação clínica da síndrome coronária crônica

1. Pacientes que receberam alta após internação por um evento de síndrome coronariana aguda ou após procedimento de revascularização coronariana.
2. Pacientes com disfunção sistólica do ventrículo esquerdo e doença arterial coronariana conhecida ou suspeita, ou aqueles com cardiomiopatia estabelecida considerada de origem isquêmica.
3. Pacientes com sintomas de angina estável (ou equivalentes isquêmicos, como dispneia ou dor no braço durante o esforço) tratados clinicamente, com ou sem resultados positivos de um teste de imagem.
4. Pacientes com sintomas de angina e evidências de vasoespasmo coronariano ou angina microvascular, com ou sem a presença de aterosclerose.
5. Pacientes diagnosticados com base exclusivamente nos resultados de um estudo de triagem (teste de estresse, angiografia coronária por tomografia computadorizada), em que o médico responsável conclui que o paciente possui doença coronariana.

Uma boa revisão pode ser avaliada na diretriz da ESC de 2024,^1^ mas há algumas informações que precisam ser vistas com cautela, como as avaliações feitas em estudos epidemiológicos em população europeia com angiotomografia (angioTC), demonstrando que homens acima de 70 anos com "angina típica" têm chances abaixo de 30% de terem de obstruções coronárias.^2^ Ressaltamos, aqui, uma mudança drástica em relação à classificação de Diamond-Forrester et al.^3^ A cautela está em uma história clínica realmente precisa ou senão, vejamos. No estudo DISCHARGE (*Diagnostic Imaging Strategies for Patients with Stable Chest Pain and Intermediate Risk of Coronary Artery Disease*),^4^ realizado em países europeus, pacientes com suspeita de doença arterial coronariana (DAC) encaminhados por generalistas para centros de cardiologia foram randomizados para angioTC de coronária ou cinecoronariografia, objetivando determinar a acurácia e os desfechos após os exames. A população foi randomizada e, só então, foi submetida a uma anamnese nos centros de cardiologia, com 38% desses pacientes tendo dor NÃO anginosa, e 36% angina atípica. Portanto, estudos populacionais podem realmente revelar uma verdade imprecisa quando avaliamos sintomas, gerando informação inadequada. E os grandes centros de cardiologia veem muito mais DAC obstrutiva em homens na presença de angina típica.

A prevenção primária e secundária, o diagnóstico e o tratamento da SCC estão evoluindo intensamente, com uma melhor compreensão da etiologia, fisiopatologia e a introdução de novos ou atualizados procedimentos diagnósticos. Além disso, o tratamento clínico sofreu considerável desenvolvimento desde a diretriz anterior.

Desejamos a todos uma leitura proveitosa desta diretriz, esperando que ela se revele um recurso valioso para os profissionais de saúde na tomada de decisões relativas à SCC.

## 2. Diagnóstico

### 2.1. Avaliação Clínica

A avaliação inicial de pacientes com suspeita de SCC fundamenta-se na história clínica detalhada e no exame físico, com análise de comorbidades, fatores de risco e impacto na qualidade de vida. Esses dados guiam a estratificação de risco e indicam a necessidade de exames complementares.^5^

A anamnese minuciosa é essencial, pois a angina é o sintoma inicial em cerca de 50% dos pacientes com SCC.^6^ Sua presença dobra a incidência de eventos cardiovasculares e os custos relacionados.^7^

Devem ser investigadas comorbidades como doença renal crônica (DRC), acidente vascular cerebral (AVC) e doença vascular periférica, além de fatores clássicos como hipertensão arterial, tabagismo, dislipidemia, diabetes, obesidade, sedentarismo, estresse psicossocial e história familiar de DAC precoce.^8^

O exame físico raramente fornece achados diagnósticos específicos, mas pode revelar sinais indiretos de risco cardiovascular e manifestações de comorbidades. Ele é fundamental na exclusão de causas não cardíacas de dor torácica.

A estimativa da probabilidade pré-teste (PPT) de DAC é uma etapa essencial e deve utilizar algoritmos clínicos.

**Figura 1:**
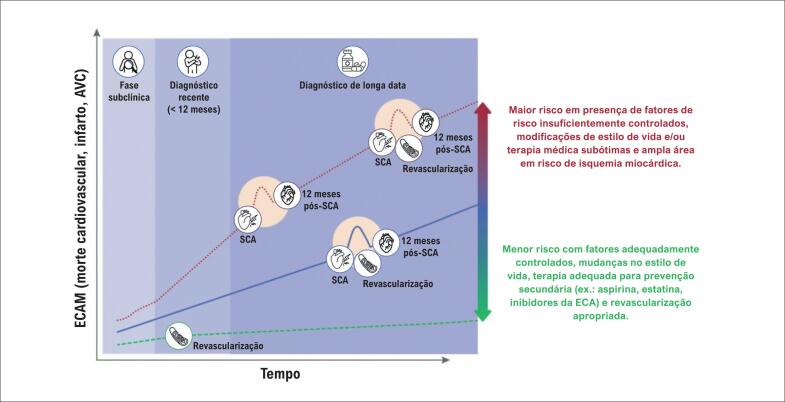
Ilustração esquemática da história natural das síndromes coronarianas crônicas. AVC: acidente vascular cerebral; ECA: enzima conversora de angiotensina; ECAM: eventos cardíacos adversos maiores; SCA: síndrome coronariana aguda.

Na Figura 1, é apresentada uma ilustração esquemática da história natural das SCCs.^1^

#### 2.1.1. Definição de Angina

A *angina pectoris* estável foi considerada uma manifestação relacionada à doença coronariana em situações estáveis e considerada, quase sempre, associada a obstruções da árvore coronária. Já há alguns anos, observa-se que, mesmo na angina típica, há muitas pessoas que não apresentam obstruções à cinecoronariografia ou, mais recentemente, pela angioTC de coronárias, como descrito na introdução desta seção.

A descrição clássica da angina foi feita por William Heberden, em 1772, como um desconforto torácico desencadeado pelo esforço, com melhora ao repouso.^9^ Hoje, reconhece-se a importância de uma anamnese criteriosa para o diagnóstico, considerando sintomas típicos e atípicos. O desconforto anginoso é, usualmente, retroesternal ou precordial, com irradiação para braços, mandíbula ou dorso, e pode ser descrito como aperto, opressão ou queimação, além de estar geralmente associado a esforço físico, durar até 20 minutos e melhora com repouso ou nitratos.

Outros sintomas, como dispneia e fadiga, denominados "equivalentes isquêmicos", ocorrem com frequência, mas têm valor diagnóstico limitado quando isolados. Em pacientes acima de 50 anos, a probabilidade de DAC obstrutiva associada à dispneia isolada é de 20 a 32% em homens e de 9 a 14% em mulheres.^4^ Por sua inespecificidade, recomenda-se mpac-los de "sintomas relacionados à isquemia miocárdica" na ausência de dor torácica.^10^

A angina pode decorrer de mecanismos além da obstrução epicárdica: disfunção endotelial, vasoespasmo coronariano (angina vasoespástica), alterações microvasculares ou disfunções metabólicas miocárdicas.^11,12^ Em estudo com quase 400 mil pacientes submetidos à angiografia coronária eletiva, apenas 41% dos que apresentavam isquemia funcional tinham DAC obstrutiva.^13^

Esses mecanismos podem coexistir. A presença de aterosclerose, mesmo suboclusiva, pode desencadear isquemia por disfunção endotelial, contribuindo para a fisiopatologia da angina.^14^

Angina é um sintoma; isquemia é um achado objetivo em exames funcionais. Pode haver angina sem isquemia detectável, e isquemia silenciosa também é comum. Nesses casos, devem-se considerar outras causas para dor torácica e dispneia. O Quadro 1 apresenta as principais etiologias de dor em tórax, epigástrio, mandíbula e pescoço.

**Quadro 1:** Causas de dor torácica, epigástrica, na mandíbula ou pescoço

Sistema	Causas possíveis
Cardíaco	Angina estável, angina instável, infarto, pericardite, miocardite
Vascular	Dissecção de aorta, tromboembolismo pulmonar
Pulmonar	Pneumotórax, pneumonia, pleurite, hipertensão pulmonar
Gastrointestinal	Doença do refluxo gastroesofágico, úlcera péptica, pancreatite, colecistite
Musculoesquelético	Costocondrite, contraturas musculares, lesões cervicais
Outros	Ansiedade, herpes-zóster (fase pré-vesicular), anemia grave

#### 2.1.2. Avaliação Clínica dos Pacientes com Dor Torácica

##### 2.1.2.1. História Clínica

A anamnese detalhada é o principal instrumento para o diagnóstico da angina.^15^ A dor torácica é uma queixa frequente, com etiologias cardíacas, pulmonares, gastrointestinais e musculoesqueléticas.^16^ A caracterização dos sintomas permite diferenciar entre dor de origem isquêmica e causas não cardíacas.

A avaliação deve considerar localização, irradiação, tipo de dor, duração, fatores desencadeantes e de alívio, além da presença de sintomas associados.^1^ Os termos mais usados pelos pacientes para descrever angina são "aperto", "opressão", "queimação" ou "peso". Nem sempre o paciente refere dor, mas apenas "desconforto". Dor em pontada ou relacionada à respiração ou posição raramente é isquêmica.^16^

A dor típica de angina localiza-se, geralmente, na região retroesternal ou precordial, com irradiação para braços, pescoço, mandíbula ou dorso, mais frequentemente à esquerda. A duração habitual é de minutos, e o alívio ocorre com repouso ou nitratos sublinguais. Desconforto súbito e fugaz ou contínuo por horas tem baixa probabilidade de origem coronariana.

Sintomas como sudorese, náusea, pré-síncope e dispneia podem acompanhar a dor, sendo denominados equivalentes anginosos. A dispneia isolada pode representar manifestação de DAC, mas deve ser interpretada com cautela.^1^ Sintomas após refeições ou nas primeiras horas da manhã são comuns na angina.

A classificação da dor torácica em típica, atípica ou não cardíaca baseia-se em três critérios: localização e tipo de dor, relação com esforço e alívio com repouso ou nitrato. A presença de todos os critérios define angina típica; de dois deles, angina atípica; de um ou nenhum, dor não anginosa.^17^ Essa classificação, embora subjetiva, é útil na estimativa da probabilidade de DAC.^18^

O Quadro 2 apresenta essa classificação. O Quadro 3 descreve a gravidade da angina conforme a Canadian Cardiovascular Society. O Quadro 4 distingue os subtipos clínicos de angina instável, condição com alto risco de evolução para infarto agudo.

**Quadro 2:** Classificação da dor torácica com base nos sintomas apresentados

Critérios:	
1. Desconforto retroesternal com qualidade e duração características	
2. Desencadeado pelo esforço físico ou estresse emocional	
3. Alívio com repouso e/ou nitrato sublingual em até 10 minutos	
**Tipo de dor**	**Características apresentadas**
Angina típica	Presença dos três critérios acima
Angina atípica	Presença de dois dos três critérios
Dor não anginosa	Presença de apenas um ou nenhum dos critérios

**Quadro 3:** Classificação de gravidade da angina (Canadian Cardiovascular Society)

Classe	Descrição
I	Angina apenas com esforço físico intenso ou prolongado
II	Limitação leve com esforço moderado (subir escadas, andar rápido)
III	Limitação significativa com esforço leve (andar um quarteirão)
IV	Angina em repouso ou com qualquer nível de atividade física

**Quadro 4:** Subtipos clínicos de angina instável

Subtipo	Descrição
Angina de início recente	Menos de 2 meses, classe III ou IV de CCS
Angina em crescendo	Aumento de frequência, intensidade ou menos limiar de esforço
Angina em repouso	Episódios de dor com duração ≥ 20 minutos, em repouso

CCS: Canadian Cardiovascular Society.

Estudos mostram que a maioria dos pacientes com dor torácica suspeita de DAC apresenta sintomas atípicos ou não anginosos, sendo a angina típica responsável por apenas 10 a 15% dos casos.^19–21^

A Tabela 2 resume os principais aspectos clínicos a serem avaliados na dor torácica: tipo, localização, irradiação, duração, fatores desencadeantes e de alívio e sintomas associados.

**Tabela 2:** Aspectos clínicos da dor torácica a serem avaliados

Parâmetro	Aspectos a serem observados
Tipo de dor	Aperto, pressão, queimação, peso ou desconforto mal definido
Localização	Retroesternal ou precordial; pode irradiar para braços, mandíbula, pescoço ou dorso
Irradiação	Mais comum para o braço esquerdo, mas pode envolver ambos os braços, mandíbula e dorso
Duração	Costuma durar de 2 a 10 minutos; dor muito fugaz ou prolongada por horas é menos sugestiva
Fatores desencadeantes	Esforço físico, estresse emocional, exposição ao frio, refeições copiosas
Fatores de alívio	Cessação do esforço, repouso, uso de nitrato sublingual
Sintomas associados	Dispneia, sudorese, náuseas, sensação de morte iminente, fadiga ou síncope

**Figura 2:**
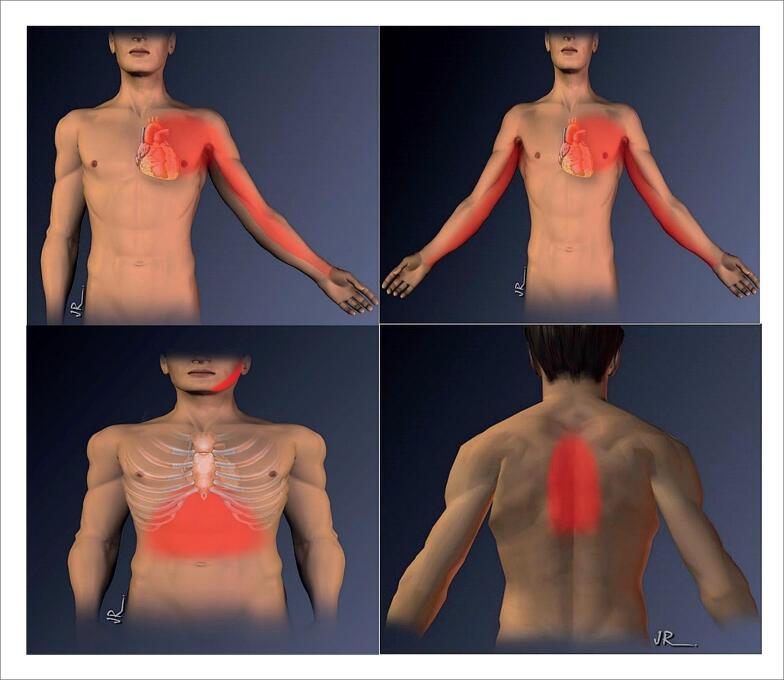
Distribuição das regiões do tórax mais comuns para queixa de angina e irradiações.

Na Figura 2, está uma representação de local e irradiações.

##### 2.1.2.2. Exame Físico

O exame físico de pacientes com DAC crônica costuma ser normal, mas pode revelar indícios indiretos de risco aterosclerótico ou de consequências da isquemia miocárdica.^22^ Sinais clínicos podem ser úteis tanto na estratificação de risco quanto no diagnóstico diferencial.

Alterações oculares, como o halo corneano (arco senil), especialmente em pacientes com menos de 40 anos, sugerem dislipidemia.^23^ O exame do fundo de olho pode mostrar alterações retinianas associadas à hipertensão arterial e ao diabetes.^24^

A presença de xantomas cutâneos ou tendinosos é indicativa de dislipidemias genéticas, como a hipercolesterolemia familiar, frequentemente acompanhada de história familiar de DAC precoce.^25^ O sinal de Frank (dobra diagonal no lóbulo da orelha) pode estar associado à DAC, doença arterial periférica (DAP) e, com o avançar da idade, torna-se frequentemente bilateral.^26^

A coexistência de doença carotídea ou vascular periférica aumenta a probabilidade de DAC. Por isso, a palpação e ausculta de artérias cervicais e femorais devem integrar o exame físico. A hipertensão arterial deve ser ativamente pesquisada por sua relevância como fator de risco.

O exame físico também contribui para o diagnóstico diferencial, podendo sugerir condições como cardiomiopatia hipertrófica ou valvopatias, responsáveis por angina não coronariana.

##### 2.1.2.3. Diagnóstico Diferencial da Dor Torácica

**Figura 3:**
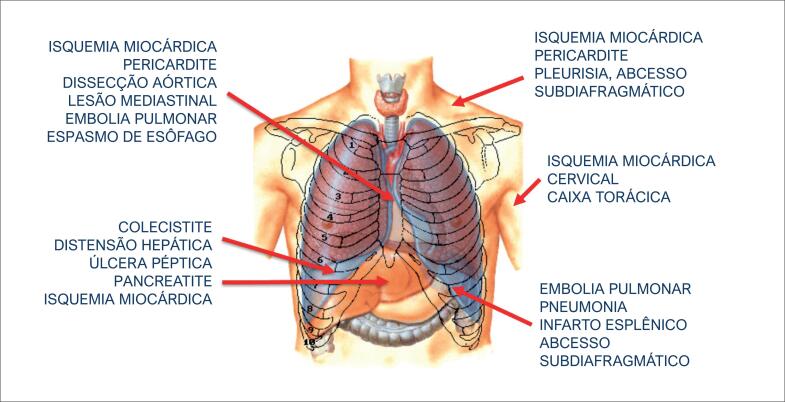
Localizações mais comuns de desconforto/dor torácica e suas causas.

Diversas condições clínicas podem gerar dor torácica semelhante à angina, principalmente por desbalanço entre oferta e consumo de oxigênio miocárdico. Essas situações devem ser reconhecidas, especialmente em pacientes com lesões coronarianas não críticas (Figura 3).^16,27^

Entre os estados de maior consumo de oxigênio, destacam-se: febre, hipertireoidismo, uso de cocaína, taquiarritmias e estresse emocional. O uso de cocaína tem se tornado causa frequente de síndrome coronariana aguda (SCA) em pacientes jovens, devido ao potente efeito vasoconstritor associado à descarga adrenérgica.^28,29^ O hipertireoidismo, por sua vez, aumenta a demanda metabólica e o consumo miocárdico de oxigênio.

A hipertensão arterial crônica, sobretudo se mal controlada, eleva a tensão parietal do ventrículo esquerdo (VE), podendo levar à isquemia subendocárdica. Mecanismo semelhante ocorre na estenose aórtica e na cardiomiopatia hipertrófica, com intensa hipertrofia e redução da reserva coronariana.^30,31^

O Quadro 5 lista condições cardíacas que precipitam angina por aumento do consumo ou redução da oferta de oxigênio. O Quadro 6 reúne causas não cardíacas de dor torácica, que devem ser consideradas no diagnóstico diferencial.

**Quadro 5:** Condições cardíacas que podem precipitar isquemia

Condição	Mecanismo provável
Estenose aórtica	Aumento da pós-carga e redução da perfusão subendocárdica
Insuficiência aórtica grave	Redução da pressão diastólica e fluxo coronariano
Cardiomiopatia hipertrófica	Hipertrofia com compressão intramiocárdica
Arritmias (taquiarritmias)	Aumento do consumo de oxigênio
Hipertensão artéria severa	Sobrecarga do ventrículo esquerdo e aumento da demanda
Insuficiência cardíaca	Redução do débito e da reserva coronariana

**Quadro 6:** Causas não cardíacas de dor torácica

Categoria	Exemplos
Gastrointestinal	Doença do refluxo gastroesofágico, úlcera, esofagite, colecistite, pancreatite
Pulmonar	Embolia pulmonar, pneumonia, pneumotórax, pleurite
Musculoesquelético	Costocondrite, distensão muscular, fraturas costais
Psicogênico	Ansiedade, transtorno de pânico
Outros	Herpes-zóster (fase inicial), anemia grave, síndrome de hiperviscosidade

#### 2.1.3. Abordagem Laboratorial

A abordagem laboratorial para a síndrome coronariana engloba a análise da presença de doença isquêmica, de fatores de risco e alterações metabólicas que possam contribuir para o agravamento do quadro obstrutivo arterial. O laboratório também deve monitorar as variáveis cujos níveis, quando alterados, associam-se a um pior prognóstico (Tabela 3).

**Tabela 3:** Testes laboratoriais para avaliação do paciente com suspeita de síndrome coronariana crônica*

Recomendação	Grau de recomendação	Nível de evidência
Perfil lipídico, incluindo LDL-C	**I**	**A**
Hemograma completo, incluindo valores de hemoglobina	**I**	**B**
Recomenda-se que a triagem para a investigação de diabetes nos pacientes com síndrome coronariana crônica seja realizada utilizando a glicose de jejum e HbA1c, com adição do teste de tolerância caso os resultados sejam inconclusivos	**I**	**B**
Medidas de creatinina e estimativa da função renal	**I**	**A**
Avaliação da função tireoidiana	**I**	**C**

LDL-C: colesterol de lipoproteínas de baixa densidade; HbA1c: hemoglobina glicada.

* Adaptado das Diretrizes da Sociedade Europeia de Cardiologia para o diagnóstico e tratamento das síndromes coronarianas crônicas.

Anemia, doenças da tireoide, diabetes e doença renal surgem como condições não cardíacas que devem ser avaliadas numa primeira abordagem, uma vez que estão associadas a eventos adversos na doença coronariana. Recomenda-se a avaliação do hemograma, TSH e T4 livre, glicose e hemoglobina glicada (HbA1c), creatinina e taxa de filtração glomerular.^1^

A anemia é uma condição associada a eventos cardiovasculares adversos maiores (ECAM). Sendo assim, pode ser considerada um indicador de mau prognóstico para pacientes com doenças cardiovasculares (DCVs). Um subestudo do estudo TIME (*Trial of Invasive vs Medical Therapy in Elderly Patients*) mostrou a associação entre redução de hemoglobina e aumento do risco de morte por causas cardiovasculares.^32^

Existem evidências sobre a associação entre hipotireoidismo subclínico e riscos cardiovasculares. O estudo de Rotterdam encontrou uma associação transversal entre hipotireoidismo subclínico e aterosclerose, medida pela calcificação da aorta abdominal e prevalência do infarto do miocárdio (IM).^33^ Cerca de 10 a 15% dos pacientes com hipotireoidismo desenvolvem algum tipo de disritmia.^34^ Além disso, o hipotireoidismo é causa secundária de dislipidemia.^35^ O tratamento com estatinas não está contraindicado para esses indivíduos, mas a introdução de estatina só deve ser iniciada após a regularização dos níveis hormonais, em função do risco aumentado de miosite nesses pacientes.^36^

O conhecimento do metabolismo da glicose é relevante pela associação entre o diabetes e complicações cardiovasculares. O risco elevado para doença coronariana é proporcional ao aumento dos níveis séricos de glicose. Valores próximos ao valor de corte para o diagnóstico de diabetes (126 mg/dL) já conferem um alto risco. Outro biomarcador da presença de hiperglicemia, a HbA1c, deve, preferencialmente, ser avaliada de forma individual, sendo a meta de tratamento para a maioria dos adultos a manutenção de valores abaixo de 7%.^38^

A disfunção renal tem impacto negativo no prognóstico da doença coronariana. A dosagem de creatinina sérica isolada não é suficiente para a avaliação da função renal, principalmente em pacientes com lesão leve ou moderada.^38^ A interferência na produção metabólica da creatinina de fatores como idade e gênero é de conhecimento geral e está fundamentada em toda prática clínica. Organizações internacionais (American Society of Nephrology–National Kidney Foundation [ASN-NKF] Task Force) aprovaram uma série de fórmulas e algoritmos que levam em conta esses fatores e a dosagem sérica da creatinina, possibilitando a estimativa da taxa de filtração glomerular.^39^

Um perfil lipídico, incluindo colesterol total, colesterol de lipoproteínas de baixa densidade (LDL-C), colesterol de lipoproteínas de alta densidade (HDL-C) e triglicérides, deve ser avaliado em pacientes com suspeita de doença coronariana para estabelecer o perfil de risco e determinar a necessidade de tratamento. Desde a flexibilização do jejum, em 2016, pelo Consenso Brasileiro para Normatização da Determinação Laboratorial do Perfil Lipídico,^40^ a identificação da condição pré-analítica em que se encontra o paciente no momento da coleta é crucial para a análise do risco cardiovascular estimado.

Além das variáveis já citadas, também a troponina vem sendo avaliada como marcador de risco para esses pacientes. A sensibilidade dos ensaios de troponina melhorou significativamente desde o lançamento inicial na década de 1990. Consequentemente, sua especificidade para infarto agudo do miocárdio (IAM) diminuiu drasticamente. Concentrações anormais são, muitas vezes, observadas em doenças não cardíacas, como insuficiência renal, sepse, embolia pulmonar e lesão cardíaca após quimioterapia. Existem muitas hipóteses sobre como a troponina é liberada no sangue de pacientes com isquemia miocárdica reversível e de pacientes com dano cardíaco não relacionado à isquemia, e os mecanismos para essa etiologia estão sendo revistos atualmente.^41^ Estudos que observaram elevação de troponina associada a comorbidades cardiovasculares e não cardiovasculares também classificaram essa elevação como preditora de eventos adversos maiores em pacientes internados, nos quais nenhum diagnóstico definitivo foi estabelecido.^42^

O estudo ARIC (*Atherosclerosis Risk in Communities*)^43^ também associou concentrações elevadas de troponina ao aumento da incidência global de DCVs na população em geral, independentemente dos fatores de risco tradicionais, sendo recomendada uma atenção especial com esses pacientes.

### 2.2. Testes Não Invasivos

#### 2.2.1. Teste Ergométrico

Método de fácil acesso devido à grande disponibilidade e baixo custo, o teste ergométrico (TE) tem indicações no paciente com DAC crônica ou suspeita, para confirmação diagnóstica, estabelecimento de prognóstico, apoio ao manejo clínico, como também para prescrição de exercícios e programas de reabilitação cardiovascular (Tabela 4).^1,44,45^

**Tabela 4:** Recomendações do TE no manejo diagnóstico inicial de pacientes com síndrome coronária crônica suspeita*

Recomendação	Grau de recomendação	Nível de evidência
O TE é recomendado em pacientes selecionados* para a avaliação de tolerância ao exercício, sintomas, arritmias, resposta da PA e estratificação de risco.^1^	**I**	**C**
Pode ser considerado como alternativa para confirmar e descartar DAC, quando métodos de imagem não invasivos não estão disponíveis.^1^	**IIb**	**B**
Pode ser considerado para refinar a estratificação de risco e tratamento.^46^	**IIb**	**B**
Não é recomendado com o intuito diagnóstico em pacientes com depressão do segmento ST ≥ 1 mm no repouso, bloqueio de ramo esquerdo ou que estão em uso de digitálicos.	**III**	**C**

DAC: doença arterial coronariana; PA: pressão arterial; TE: teste ergométrico.

* Quando houver uma perspectiva de que o TE impacte na estratégia diagnóstica ou manejo clínico.

* Todas as indicações de forma mais específica devem ser consultadas na diretriz de ergometria com subpopulações que não se aplicam aqui na diretriz de síndrome coronária crônica.^45^

A análise do TE deve incluir as variáveis eletrocardiográficas, clínicas, hemodinâmicas, autonômicas e a capacidade funcional (CF), pois acrescentam informações prognósticas no acompanhamento e na mudança de conduta do paciente com DAC. Na avaliação do eletrocardiograma (ECG) ao esforço, a depressão do segmento ST ≥ 1 mm, a 0,80 m/s do ponto J horizontal ou descendente, é a alteração mais preditiva de isquemia, sendo sua magnitude, precocidade do aparecimento, número de derivações com alterações e tempo de normalização na recuperação indicadores de maior gravidade da resposta isquêmica. A depressão do segmento ST assintomática durante o TE em homens de meia idade com fatores de risco foi preditora de morte súbita cardíaca (MSC) e morte de causa coronária (risco 2 a 2,5 vezes maior, respectivamente) quando comparada àquelas sem alteração. Já a depressão de ST assintomática na fase de recuperação, além do mesmo valor diagnóstico, tem ainda maior impacto no prognóstico (3 a 4 vezes mais eventos – MSC e morte coronária, respectivamente).^47^ O supradesnivelamento do segmento ST no TE em derivações sem área inativa é a alteração mais grave que pode ser encontrada e representa isquemia transmural. Apesar de raro (0,1%), tem alto risco de arritmias graves (taquicardia ventricular [TV], fibrilação ventricular [FV]). Semelhante ao infarto, também localiza a artéria envolvida.^45^ A concomitância de dor anginosa de intensidade progressiva e a queda da pressão arterial sistólica (PAS) reforçam o diagnóstico e a gravidade. É importante salientar que menos de 50% dos Tes isquêmicos têm manifestação clínica de angina. É considerada ECG não interpretável para o TE a presença de bloqueio do ramo esquerdo (BRE), marcapasso cardíaco artificial, pré-excitação ventricular, depressão de ST ≥ 1,0 mm em repouso e uso de digital, pois podem alterar o ST na ausência de DAC.^45^ Nessas condições, deve-se realizar método de imagem. Escores prognósticos: entre os escores prognósticos, o escore de Duke (ED) é o mais difundido e utiliza variáveis do TE que permitem a estratificação na DAC. Pode ser utilizado na avaliação diagnóstica e prognóstica.^48^

A curva deprimida da PAS ao esforço e/ou a sua queda progressiva, principalmente com a elevação paradoxal no 1° min da recuperação, são comportamentos que se associam a DAC grave, pois indicam comprometimento da função ventricular.^49,50^ A CF estimada em múltiplos de equivalentes metabólicos (METs) ao TE é a variável prognóstica de maior impacto já estabelecido na literatura. A baixa CF no esforço máximo associa-se à maior mortalidade total e cardiovascular em diferentes populações (homens, mulheres, portadores de fatores de risco, presença de DAC etc.). Evolutivamente nos pacientes com DAC estabelecida, a sua diminuição também é preditor de eventos, de acordo com a CF estimada.^51^ O risco de morte aumenta significativamente nos homens com fatores de risco que têm CF < 5 METs, com risco de morte em 6 anos de 4 a 4,5 vezes maior. A CF > 10 METs prediz excelente prognóstico, independentemente de fatores de risco.^51^ Nas mulheres, CF < 5 METs associa-se a três vezes maior mortalidade do que aquelas que atingem > 8 METs e, entre 5-8 METs, duas vezes maior mortalidade, independentemente da presença de infradesnivelamento do segmento ST e de fatores de risco.^52,53^ Além disso, a avaliação da CF é fundamental em pacientes com DAC estabelecida para a prescrição de exercícios (identificação de limiares isquêmicos, clínico e/ou ECG, caso a isquemia seja detectada) e ajustes no seguimento da programação de reabilitação cardiovascular. A Tabela 1 resume as principais recomendações do TE no manejo diagnóstico inicial de pacientes com SCC suspeita. Para informações mais detalhadas, consultar a diretriz brasileira de ergometria em população adulta.^40^

#### 2.2.2. Ecocardiografia

##### 2.2.2.1. Ecocardiografia Transtorácica

A ecocardiografia transtorácica é a modalidade de primeira linha na avaliação de pacientes com DAC subclínica ou manifesta. Esse método permite a análise da função sistólica global e segmentar do VE, função diastólica do VE, complicações relacionadas à DAC crônica, bem como a identificação de outras causas de dor torácica e dispneia, que, muitas vezes, podem ser coexistentes com a DAC (Tabela 5).^1,54,55^ A função sistólica global é comumente avaliada pela medida da fração de ejeção do VE (FEVE), que pode ser quantificada pelo método biplano de discos (Simpson), ao bidimensional ou, se disponível, pelo método 3D (mais acurado, pois não depende de suposições geométricas). A função segmentar do VE é estimada utilizando o *wall motion score index* (WMSI).^16^ A FEVE, por ser um índice volumétrico, e o WMSI, por ser um índice visual, podem não refletir acuradamente a função contrátil. Mais recentemente, o *strain* longitudinal (SL) global miocárdico, derivado do *speckle tracking*, tem se mostrado um marcador mais acurado, sensível e reprodutível de disfunção subclínica (se < 16%), na DAC aguda^56^ e na crônica.^1,54^ Já a redução do SL regional, evidenciada ao mapa polar ou pelas curvas de *strain*, pode sugerir, de forma mais acurada que o WMSI, a presença de isquemia ou fibrose.^57,58^

**Tabela 5:** Recomendações do uso da ecocardiografia transtorácica na avaliação diagnóstica inicial de indivíduos com síndrome coronária crônica suspeita

Recomendação	Grau de recomendação	Nível de evidência
Avaliar a função global e segmentar do VE, volumes, função diastólica, função ventricular direita, estimar a pressão sistólica da artéria pulmonar, refinar a estratificação de risco e guiar o tratamento.	**I**	**B**
Avaliar diagnósticos diferenciais ou coexistentes de dor precordial ou dispneia tais como doença valvar, hipertrofia, cardiomiopatia e derrame pericárdico.	**I**	**B**
Avaliar *strain* longitudinal global na avaliação da função sistólica global e regional do VE, se FEVE for normal e houver alta probabilidade clínica de DAC.*	**IIa**	**B**
Reavaliação periódica de pacientes estáveis sem mudança clínica.	**III**	**C**

DAC: doença arterial coronariana; FEVE: fração de ejeção do ventrículo esquerdo; VE: ventrículo esquerdo.

* Deve-se ressaltar que o strain longitudinal global deve ser interpretado de forma comparativa, dentro de um contexto clínico, usando o mesmo fabricante e mesma versão do software e em condições de carga semelhantes.

##### 2.2.2.2. Ecocardiografia sob Estresse

A ecocardiografia sob estresse é um método bem estabelecido no diagnóstico da DAC obstrutiva suspeita ou conhecida, além de permitir a avaliação do prognóstico, de viabilidade miocárdica e do impacto de terapias de revascularização (Tabela 6).^59,60^ O estresse cardiovascular causa isquemia miocárdica em regiões supridas por uma artéria coronária com grau significativo de estenose, que se manifesta sequencialmente por alterações na perfusão miocárdica, na contratilidade segmentar, nas alterações eletrocardiográficas e, por fim, na ocorrência de angina.^61–64^ O estresse pode ser desencadeado pelo esforço físico (esteira ou bicicleta ergométrica), uso de drogas vasodilatadoras (dipiridamol associado ou não à atropina) ou estimulantes adrenérgicos (dobutamina associada ou não à atropina), todos com boa acurácia (85 a 90%) na detecção de obstruções coronarianas em pacientes com probabilidade pré-teste intermediária ou alta.^65,66^ Um exame normal apresenta alto valor preditivo negativo (93 a 100%) para eventos cardiovasculares.

**Tabela 6:** Recomendações da utilização do ecocardiograma sob estresse na síndrome coronária crônica suspeita

Recomendação	Grau de recomendação	Nível de evidência
Investigação de isquemia e estimativa do risco de eventos cardiovasculares em indivíduo sintomático com probabilidade pré-teste intermediária ou alta	**I**	**B**

**Figura 4:**
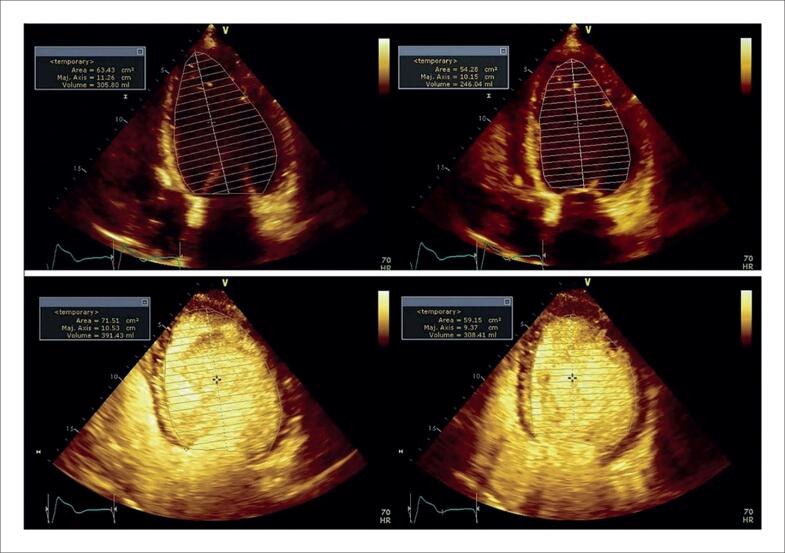
Diferenças nos volumes diastólico final e sistólico final observadas no mesmo paciente sem contraste (parte superior) e com UEAs e imagens de baixo IM (parte inferior). Linha superior, da esquerda para a direita: quantificação do VE pré-contraste do volume diastólico final (306 mL) e volume sistólico final (246 mL) para estimativa da FEVE. Linha inferior, da esquerda para a direita: quantificação do VE pós-contraste do volume diastólico final (391 mL) e do volume sistólico final (308 mL) para estimativa da FEVE. Um aumento acentuado no tamanho do volume é observado após o contraste. FEVE: fração de ejeção do ventrículo esquerdo; VE: ventrículo esquerdo; IM: infarto do miocárdio; UEA: unidade de avaliação de esforço.

**Figura 5:**
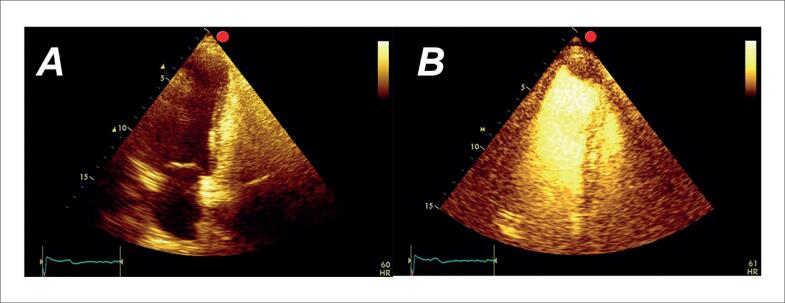
Imagem obtida no plano apical de três câmaras, demonstrando, em A, impossibilidade de visibilização da parede lateral inferior e ápice. Em B, observa-se além do delineamento adequado dos segmentos não visibilizados previamente, o aparecimento de um trombo apical.

**Figura 6:**
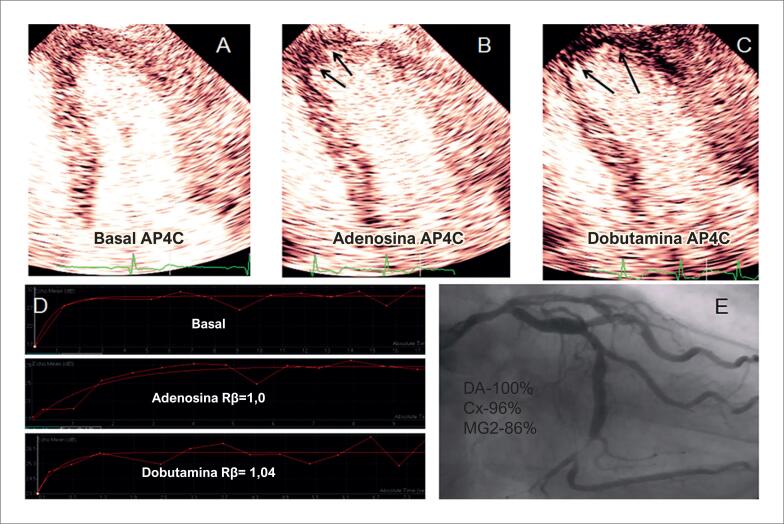
Plano apical de quatro câmaras de dois exames de estresse farmacológico no mesmo paciente, associado a contrastes por microbolhas a fim de se avaliar a motilidade e perfusão miocárdica. Em A, nota-se a perfusão normal em repouso; em B, um defeito de perfusão transmural em um único segmento apical durante o estresse pela adenosina; e, em C, um extenso defeito de perfusão apical transmural em ao menos dois segmentos, associado a alterações da motilidade no mesmo território (acinesia). Em D, por meio de um software específico, são geradas as curvas de intensidade acústica/tempo, demonstrando uma reserva de fluxo coronário muito deprimida em ambas as formas de estresse. Em E, há angiografia coronária em oblíqua anterior esquerda (OAE) demonstrando obstrução grave em artéria descendente anterior e circunflexa.

A ecocardiografia com contraste miocárdico (ECM), por meio da infusão de agentes de realce do ultrassom,^59,66^ deve ser utilizada no repouso ou durante o estresse em pacientes com estudos tecnicamente difíceis para melhorar o delineamento das bordas endocárdicas.^74–76^ Esses agentes permitem medidas mais precisas dos volumes e fração de ejeção (Figura 4), detecção de trombos intracavitários (Figura 5) e análise mais acurada da contratilidade segmentar, entre outras aplicações,^66,67^ aumentando o número de exames diagnósticos.^67^ Em indivíduos com função do VE normal, vários estudos também demonstraram incremento diagnóstico e prognóstico, com o uso adicional da ECM para avaliação qualitativa e quantitativa da perfusão miocárdica em repouso e sob estresse^69^ (vide exemplo na Figura 6). Sugerimos o posicionamento sobre indicações da ecocardiografia em adultos da SBC para mais detalhes.^55^

#### 2.2.3. Medicina Nuclear

Os métodos de imagem não invasivos desempenham papel fundamental no diagnóstico e manejo da doença isquêmica cardíaca (DIC).

**Figura 7:**
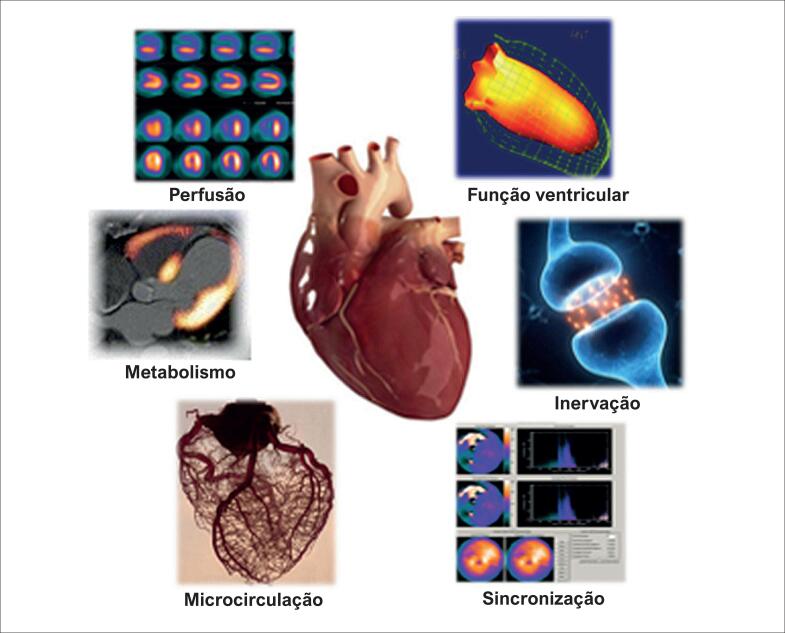
Aplicações da cardiologia nuclear na doença isquêmica cardíaca.

A cardiologia nuclear é uma modalidade de imagem fisiológica, que utiliza radiofármacos para o estudo dos mecanismos fisiopatológicos das DCVs, sendo capaz de avaliar todo o espectro da DIC, desde DAC epicárdica obstrutiva até DAC microvascular,^70^ abrangendo, além da perfusão e função ventricular, o metabolismo, a inervação e o sincronismo^71^ (Figura 7).

A cintilografia de perfusão miocárdica (NA) pela técnica de tomografia computadorizada por emissão de fóton único (*single photon emission computed tomography* [SPECT]) é utilizada há décadas na prática clínica devido à sua ampla disponibilidade e extensa literatura, que respalda seu valor no diagnóstico e estratificação de risco da DIC.^72,73^ Ela emprega os radiofármacos sestamibi ou tetrofosmina marcados com tecnécio 99 metaestável (^99m^Tc) para a pesquisa de isquemia e, menos frequentemente e em casos específicos, para avaliação de viabilidade miocárdica, o tálio 201(^201^Tl).^20,74–78^

**Figura 8:**
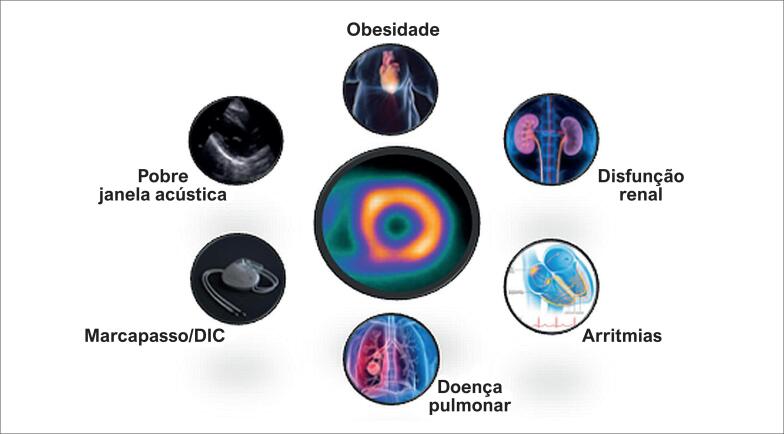
Possibilidades de aplicação das técnicas nucleares.

É o exame de imagem não invasivo mais comumente empregado na avaliação de pacientes com DIC crônica suspeita ou conhecida, podendo ser realizado com sucesso em praticamente todos os grupos de pacientes, independentemente da função renal, presença de arritmias, obesidade ou dispositivos intracardíacos, situações muitas vezes desafiadoras para técnicas alternativas de investigação^72,77^ (Figura 8).

**Figura 9:**
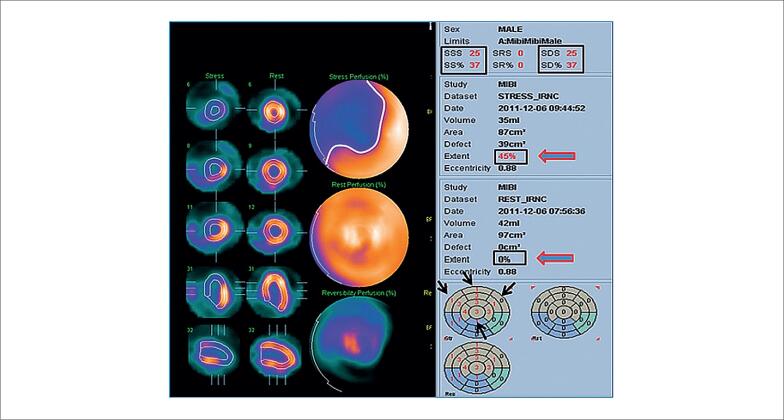
NA GATED-SPECT: isquemia estresse induzida de grande extensão. Nota-se extensa área de hipocaptação envolvendo as paredes anterior, região septal, ápice, estendendo-se, ainda, à porção distal das paredes inferior e lateral, evidenciada em coloração azul e delimitada no mapa polar (MP) de estresse por linha branca (imagem superior à esquerda). O MP representativo da perfusão em repouso evidencia distribuição homogênea do radiofármaco, traduzindo perfusão normal. A representação gráfica dos escores de estresse (setas pretas), evidencia a graduação numérica da intensidade da hipocaptação e o número de segmentos acometidos (canto inferior direito), com os somatórios SSS = 25, SRS = 0 e SDS – 25 (canto superior direito), além do porcentual de miocárdio em risco estimado em 45% do ventrículo esquerdo (carga isquêmica). A cinecoronariografia evidenciou lesão proximal suboclusiva de artéria descendente anterior. SDS: somatório da diferença dos escores (*summed difference score*); SRS: somatório do escore de repouso (*summed rest score*); SSS: somatório do escore de estresse (*summed stress score*).

A NA (SPECT) é capaz de fornecer, em um único exame, imagens da perfusão cardíaca relativa, avaliando, de modo predominante, a extensão e a intensidade da isquemia miocárdica, podendo quantificar o déficit perfusional em valores porcentuais do VE, bem como avaliar os volumes ventriculares e as frações de ejeção em repouso e após o estresse, entre outros marcadores (Figura 9).

Fonte: Arquivo pessoal.

A NA é realizada em duas etapas, de repouso e de estresse cardiovascular, seja físico ou farmacológico. O estresse físico (por TE ou teste cardiopulmonar de exercício) deve ser a técnica de escolha quando o paciente for capaz de se exercitar adequadamente (atingir gasto metabólico ≥ 5 METs), por fornecer importantes informações diagnósticas e prognósticas adicionais às imagens, sendo o estresse farmacológico reservado para aqueles com limitações físicas impeditivas, com contraindicações formais ao TE, com BRE ou dispositivos intracardíacos, entre outras situações.^72,77^

Pacientes sintomáticos com risco intermediário a alto para cardiopatia isquêmica são os que mais se beneficiam da NA. As principais indicações e níveis de evidência para as imagens funcionais pela cardiologia nuclear estão demonstradas na Tabela 7.^1,44,74,79,80^

**Tabela 7:** Recomendações da NA de estresse e repouso no manejo diagnóstico inicial de pacientes com síndrome coronária crônica suspeita

Recomendação	Grau de recomendação	Nível de evidência
Em pacientes com síndrome coronária crônica suspeita e probabilidade pré-teste de DAC obstrutiva moderada ou alta, a SPECT com estresse ou, preferencialmente, a imagem de perfusão pelo PET é recomendada para: diagnosticar e quantificar isquemia miocárdica e/ou cicatriz; estimar o risco de eventos cardiovasculares; quantificar o fluxo miocárdico (PET).	**I**	**B**

NA: cintilografia de perfusão miocárdica; DAC: doença arterial coronariana; PET: tomografia por emissão de pósitron; SPECT: tomografia computadorizada por emissão de fóton único.

Uma desvantagem da NA é a exposição à radiação, embora tecnologias mais recentes, como novas gama-câmaras de detectores de estado sólido de cádmio, zinco e telúrio (CZT), melhorias nos radiofármacos, protocolos e doses de traçadores individualizadas, tenham reduzido essa exposição.^73^

##### 2.2.3.1. Decisão Clínica após Estudos de Perfusão com Radionuclídeos – Impacto da Quantidade de Isquemia nos Desfechos Cardiovasculares^81–87^

**Figura 10:**
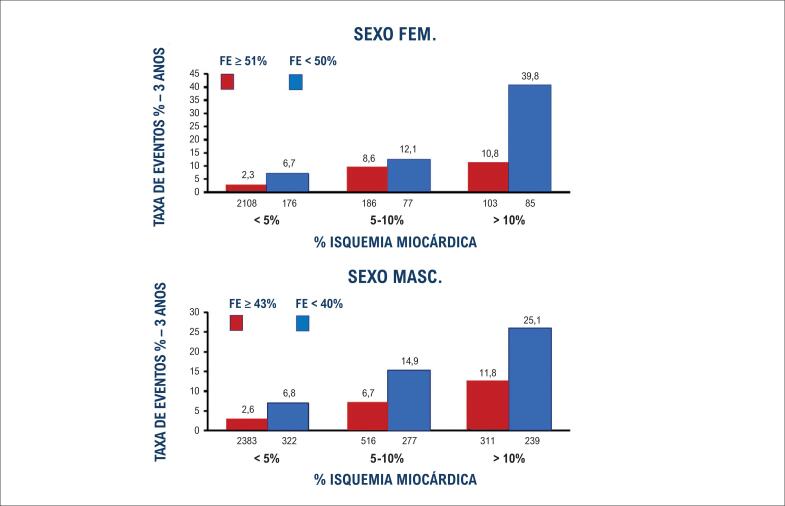
Taxa ajustada de morte cardíaca ou infarto do miocárdio em pacientes do sexo feminino e masculino encaminhados à cintilografia de perfusão miocárdica em função do porcentual de isquemia e função sistólica do ventrículo esquerdo (adaptado de Sharir et al.^87^). FE: fração de ejeção; FEM.: feminino; MASC.: masculino.

A NA apresenta consolidada base de evidências que demonstra o elevado valor preditivo negativo de um exame normal, com baixo risco de eventos cardiovasculares em períodos de seguimentos variados, a depender da população sob estudo, independente do sexo. O valor prognóstico da NA normal é excelente, com 99% de sobrevida livre de eventos segundo grande metanálise.^88^ Da mesma forma, eleva-se a probabilidade de eventos maiores (morte cardiovascular e por todas as causas, infarto não fatal) de modo exponencial à medida que a carga isquêmica (extensão e intensidade dos defeitos de captação) aumenta. Tais informações perfusionais devem estar integradas às da função ventricular, que agrega valor prognóstico incremental quando está diminuída (Figura 10).

**Figura 11:**
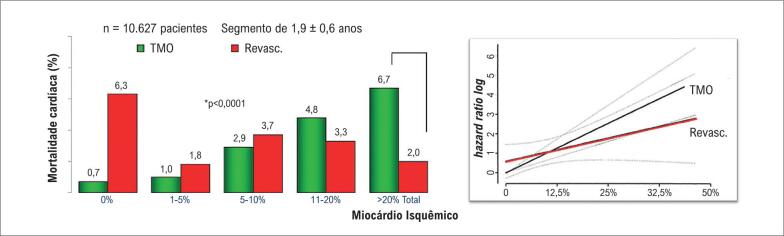
Taxa de morte cardíaca estratificada pela quantificação da isquemia (SPECT) e modalidade de tratamento (adaptado de Hachamovitch et al.^90^). Revasc.: revascularização; SPECT: tomografia computadorizada por emissão de fóton único; TMO: tratamento medicamentoso otimizado.

Estudos observacionais clássicos e robustos^82,89^ apontaram que a extensão da isquemia é um dos preditores mais importantes do benefício da revascularização miocárdica, não demonstrando redução de desfechos cardiovasculares na ausência de isquemia ou na presença de isquemia discreta, porém, com evidência de benefício quando a carga isquêmica ultrapassa 10 a 12% do VE (Figura 11).

Contrário aos trabalhos observacionais,^81,82,88,90–92^ o estudo randomizado ISCHEMIA (*International Study of Comparative Health Effectiveness with Medical and Invasive Approaches*)^93^ não evidenciou benefícios na diminuição dos eventos estabelecidos quando feita a comparação entre a revascularização miocárdica e o tratamento medicamentoso otimizado em pacientes com isquemia pelo menos moderada, caracterizada por provas funcionais não invasivas, num período de seguimento inicial de 3,2 anos. Ressalta-se que as imagens SPECT não representaram o único método de avaliação da isquemia na inclusão dos pacientes, sendo em torno de 25% os pacientes incluídos após testes ergométricos considerados de alto risco, sem imagens.

Tais resultados desencadearam uma acalorada discussão no meio médico, face à possibilidade de mudança de um paradigma até então estabelecido dentro do tratamento na doença isquêmica do coração. No entanto, destaca-se que subestudos do ISCHEMIA sugerem que, para pacientes com isquemia miocárdica, insuficiência cardíaca (IC) e FEVE de 35 a 45%, uma estratégia invasiva precoce pode melhorar a sobrevida livre de eventos.^93^ Além disso, as curvas de mortalidade do estudo principal começaram a se separar após 2 anos de seguimento médico, com aparente benefício para a revascularização e com possíveis implicações futuras a longo prazo, o que justificou a extensão do acompanhamento clínico dos pacientes, resultando na publicação recente do estudo *Extended* ISCHEMIA. Esse estudo prolonga o seguimento mediano para 5,7 anos, não mostrando diferenças entre os grupos randomizados de tratamento para mortalidade por todas as causas, mas exibindo menor mortalidade cardiovascular para a estratégia inicial invasiva em 7 anos, apesar de, paradoxalmente, exibir maior mortalidade "não cardiovascular".^94^

É importante ressaltar que, de qualquer modo, o estudo ISCHEMIA foi um grande e importante estudo de avaliação terapêutica e, em nenhum momento, dispôs-se a questionar a abordagem diagnóstica e a estratificação de risco dos pacientes com DAC suspeita ou conhecida. A estratificação de risco é peça fundamental para a individualização e o auxílio na tomada de decisão clínica, implicando no adequado manejo da DIC.

Por fim, vale ressaltar o papel da cardiologia nuclear na avaliação da viabilidade miocárdica, sendo o padrão-ouro a PET/CT (tomografia por emissão de pósitrons/tomografia computadorizada) com glicose marcada com flúor 18 para avaliação do metabolismo glicolítico (^18^F-FDG-PET/CT), podendo também ser utilizada a cintilografia SPECT com ^201^tálio quando a PET/CT não estiver disponível.^44,95–99^

##### 2.2.3.2. Tomografia por Emissão de Pósitrons (PET)

**Figura 12:**
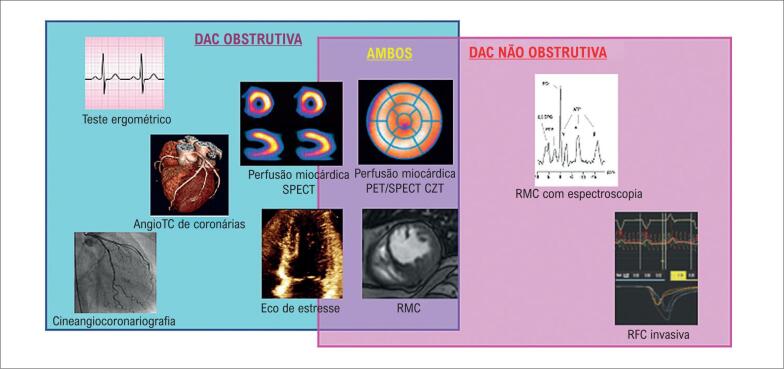
Modalidades para avaliação de doença arterial coronariana obstrutiva e não obstrutiva. AngioTC: angiotomografia; CZT: cádmio, zinco e telúrio; DAC: doença arterial coronariana; PET: tomografia por emissão de pósitron; RFC: reserva de fluxo coronariana; RMC: ressonância magnética cardíaca; SPECT: tomografia computadorizada por emissão de fóton único. Adaptado de Koilpillai et al.^100^

A PET é considerada o método padrão-ouro para avaliação não invasiva da perfusão miocárdica. Embora não disponível no país devido à indisponibilidade dos radiofármacos utilizados (rubídio-82 [^82^ Rb], amônia-N13 [^13^N – Amônia] ou água-O15 [^15^° – H2O]), merece destaque na DAC, considerando-se a capacidade em quantificar, de forma absoluta e não invasiva, o fluxo sanguíneo miocárdico (FSM) de estresse e repouso, além de determinar a reserva de fluxo coronariana (RFC). Esses dados fornecem informações úteis para refinar o diagnóstico, o prognóstico e o manejo dos pacientes, abrangendo o espectro da doença isquêmica do coração, desde a DAC epicárdica multiarterial, até a disfunção microvascular coronariana difusa (Figuras 12 e 13).

**Figura 13:**
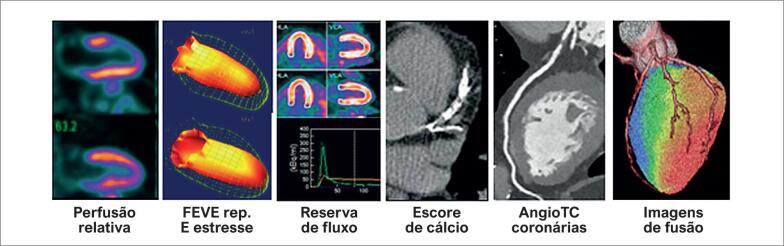
Imagens de perfusão por PET/TC: informações abrangentes. AngioTC: angiotomografia; TC: tomografia computadorizada; FEVE: fração de ejeção do ventrículo esquerdo; rep.: repouso; PET: tomografia por emissão de pósitrons. Adaptado Cheng et al.^21^

A diretriz europeia sobre SCC de 2019, divide os fenótipos da DAC crônica em três grupos e traz, como destaque, a importância da avaliação de doença da microcirculação coronariana, afirmando que a exclusão angiográfica de estenose epicárdica não mais significa a via final dos procedimentos diagnósticos. Inclui, ainda, a PET para avaliação não invasiva da RFC em pacientes com suspeita de angina microvascular coronariana.^97–103^

A angina microvascular é estabelecida após exclusão de estenoses coronarianas epicárdicas, implicando em prognóstico adverso. A disfunção microvascular precede o desenvolvimento de lesões epicárdicas, particularmente em mulheres,^104^ e está associada a pior evolução. A presença de RFC anormal está associada a maior risco de eventos, mesmo em pacientes sem estenoses coronarianas significativas, principalmente em diabéticos.^81,105^ Dessa forma, a detecção não invasiva de DAC em estágio subclínico identifica subgrupos de pacientes em risco para eventos cardíacos futuros que podem se beneficiar de prevenção precoce e agressiva. Adicionalmente, a resposta a eventuais medicações preventivas pode ser avaliada através de medidas seriadas da RFC, podendo demonstrar alterações favoráveis com intervenções apropriadas.^106–109^

##### 2.2.3.3. Reserva Coronariana com SPECT CZT

O advento de novos equipamentos SPECT CZT permite a realização de aquisições dinâmicas para o cálculo absoluto do fluxo sanguíneo coronariano (em repouso e na condição de estresse) e a determinação da reserva de fluxo coronariano utilizando radiotraçadores marcados com tecnécio-99m, amplamente disponíveis internacionalmente e em nosso meio. Assim, a disponibilidade de radiotraçadores, o menor custo em relação à PET/CT, além da "rapidez" na aquisição das imagens torna a quantificação de FSM e RFC uma ferramenta mais próxima de nossa realidade atual.

Vários estudos vêm demonstrando a boa correlação dos valores estimados de FSM e RFC utilizando radiotraçadores marcados com tecnécio-99m em equipamento SPECT CZT com aquelas medidas usando imagens de PET/CT com ^15^°-água ou ^13^N-amônia como marcadores de perfusão.

Embora bastante promissor, é importante ressaltar que a maior parte dos estudos até o momento é de centros únicos e conta com amostras pequenas, contudo, essa tecnologia desperta muito interesse da comunidade científica, uma vez que os radiofármacos utilizados são amplamente disponíveis, e os equipamentos têm um custo bem menor quando comparados à PET/CT.

#### 2.2.4. Abordagem Laboratorial

A investigação laboratorial na DAC estável busca identificar fatores de risco modificáveis e condições associadas que aumentam o risco cardiovascular. Os exames recomendados incluem lipidograma, glicemia de jejum, hemoglobina glicada, função renal (ureia, creatinina, estimativa do *clearance* de creatinina), hemograma e TSH (Tabela 8).^25^

**Tabela 8:** Testes laboratoriais para avaliação do paciente com suspeita de síndrome coronariana crônica*

Recomendação	Grau de recomendação	Nível de evidência
Perfil lipídico, incluindo LDL-C	**I**	**A**
Hemograma completo, incluindo valores de hemoglobina	**I**	**B**
Recomenda-se que a triagem para investigação de diabetes nos pacientes com síndrome coronariana crônica seja realizada utilizando a glicose de jejum e HbA1c, com adição do teste de tolerância caso os resultados sejam inconclusivos	**I**	**B**
Medidas de creatinina e estimativa da função renal	**I**	**A**
Avaliação da função tireoidiana	**I**	**C**

LDL-C: colesterol de lipoproteínas de baixa densidade.

* Adaptado Knuuti et al.^1^

O lipidograma deve incluir colesterol total, HDL, LDL (calculado ou direto) e triglicerídeos (TGs). A apolipoproteína B (apoB) e a lipoproteína (Lp) (a) podem ser úteis em pacientes de risco muito alto ou com histórico familiar de DAC precoce.^110^ A Lp(a), herdada geneticamente, confere risco independente de eventos aterotrombóticos e não é influenciada por dieta ou estilo de vida.^111^

A avaliação da função renal é essencial, pois a taxa de filtração glomerular < 60 mL/min/1,73 m^2^ está associada a aumento do risco cardiovascular. Em pacientes com hipertensão, a pesquisa de microalbuminúria pode ser considerada.

A dosagem de peptídeos natriuréticos (peptídeo natriurético tipo B [BNP] ou N-terminal do pró-hormônio do BNP [NT-proBNP]) pode ser útil na suspeita de disfunção ventricular esquerda, especialmente em pacientes com dispneia. A troponina de alta sensibilidade também pode ser usada como marcador prognóstico em casos selecionados, mesmo fora do contexto de SCA.^112,113^

Outros exames incluem ácido úrico (associado ao risco cardiovascular em níveis elevados), proteína C reativa ultrassensível (PCR-us) e, em casos específicos, marcadores genéticos ou perfil metabólico ampliado.

#### 2.2.5. Tomografia de Coronárias

É possível avaliar as artérias coronárias através da tomografia computadorizada de duas maneiras: pela avaliação da presença de cálcio nas artérias e pela luminografia, com uso de contraste, detectando a presença de placas de ateroma e estimando o grau de obstruções, quando existem, e, claro, também pode determinar o escore de cálcio coronário.

##### 2.2.5.1. Tomografia de Coronárias para Determinar o Escore de Cálcio Coronário

A tomografia de coronárias sem contraste para determinar o escore de cálcio coronário é utilíssima para a avaliação de pacientes de moderado risco e assintomáticos e para auxiliar na programação da prevenção do uso de fármacos antiateroscleróticos na prevenção primária. É um método simples, sem uso de contraste, com obtenção de imagem em menos de 10 minutos e até menos em tomógrafos mais recentes. Deveria ser incrementada a sua indicação, e essa é uma oportunidade de incluir esse exame, conforme as especificações desta diretriz, como exame muito útil. Para a avaliação desses pacientes de baixo risco e antecedentes ou de moderado risco assintomáticos, pois o incremento ou o decréscimo do risco de um evento isquêmico coronário é modificado muito com a presença ou ausência de cálcio coronário. É muito mais específico do que a presença ou ausência de placas em carótidas. Uma boa revisão sobre esse método pode ser vista em diretriz da ESC de escore de cálcio.^114^

De forma resumida as indicações são as abaixo, conforme a mesma diretriz citada acima (Tabela 9).

**Tabela 9:** Recomendações para a Tomografia de Coronárias para Determinar o Escore de Cálcio Coronário

Recomendações	Classe de recomendação	Nível de evidência
Reestratificação de risco em pacientes assintomáticos com escore de risco clínico intermediário	**I**	**A**
Reestratificação de risco em pacientes assintomáticos com diabetes mellitus ou síndrome metabólica e com escore de risco clínico intermediário	**I**	**B**
Reestratificação de risco em pacientes assintomáticos com escore de risco clínico intermediário para orientação quanto à prevenção primária medicamentosa		
Reestratificação de risco em pacientes assintomáticos portadores de hipercolesterolemia familiar heterozigótica		
Reestratificação de risco em pacientes assintomáticos com escore de risco clínico baixo e com história familiar de DAC precoce	**IIa**	**B**
Triagem para pesquisa de isquemia miocárdica em pacientes assintomáticos com diabetes mellitus		
Realização do escore de cálcio coronariano para melhor estratificação de risco em pacientes sem DAC conhecida submetidos a testes de isquemia com resultados negativos		
Para afastar estenose coronária significativa em pacientes sintomáticos com suspeita de angina estável ou síndrome coronária aguda	**III**	**B**
Uso em pacientes com DAC obstrutiva conhecida	**III**	**C**

DAC: doença arterial coronariana.

##### 2.2.5.2. Angiotomografia Coronária

A angioTC das artérias coronárias é uma forma não invasiva de avaliar as artérias que nutrem o miocárdio e que faculta a análise da luz desses vasos, de suas paredes e, mais recentemente, também possibilita o estudo das características dos ateromas que eventualmente possam comprometer aqueles vasos.^115^ Desde os períodos iniciais da aplicação clínica desse método, ele se destacava pelo seu elevado poder preditivo negativo, mostrando-se eficaz para descartar com segurança a presença de doença coronariana obstrutiva.^116,117^ Porém, à medida que a tecnologia e a experiência com esse exame aumentaram, houve aumento do poder preditivo positivo, que pode ser ainda mais elevado no caso de se utilizar a análise não invasiva da reserva de fluxo coronária (RFC), o que lhe dá grande potencial de uso clínico.^115,118^

A principal contribuição da tomografia é vista nos grupos de probabilidade intermediária, quanto à existência de doença coronariana obstrutiva e, do mesmo modo, percebe-se que testes de elaboração e refinamento mais recentes são mais fiéis em relação à probabilidade de haver ateromatose arterial, ao contrário de modelos mais antigos, que superestimam a chance da presença de estenoses significativas.^1,119–121^ Em decorrência disso, alguns autores afirmam que se deve proceder ao cálculo do risco a partir da estimativa de risco antes de se solicitar a angioTC diante da suspeita de doença coronariana crônica.^122^

Além disso, alguns investigadores sugerem que se empregue o escore de cálcio coronário nos pacientes com dor precordial e de baixo risco antes de prosseguir na solicitação de exames, pois, em ensaios populacionais, a maior parte dos eventos aconteceu nos casos em que havia detecção de cálcio (84% de eventos adversos maiores em pacientes com escore > 0 no estudo PROMISE).^1,120,121^ E mais, isso é verdadeiro em pessoas sem nenhum sintoma (prevenção primária).

Por outro lado, estudos já demonstraram que pode haver obstruções significativas mesmo na ausência de calcificação arterial, e esse critério deve ser usado com cautela, em especial nos pacientes de moderado mais para alto risco.^122^

Detalhes e minúcias sobre esse tema podem ser consultados na diretriz de tomografia e ressonância magnética cardiovascular recém-publicada.^123^ As indicações para angioTC das artérias coronárias em pacientes com doença coronariana crônica de baixo risco encontram-se na Tabela 10.

**Tabela 10:** Níveis de evidência para indicações de tomografia em pacientes com suspeita de doença coronariana crônica e baixo risco pré-teste

Recomendação	Grau de recomendação	Nível de evidência
Diagnóstico de doença coronariana em pacientes com testes funcionais de resultado incerto/conflitante, como alternativa ao estudo invasivo	**IIa**	**B**
Como primeira opção para o diagnóstico de pacientes com dor torácica	**IIb**	**B**
Para estimativa da carga aterosclerótica em pacientes sem o diagnóstico prévio de doença arterial coronariana	**IIb**	**B**

Nos casos de risco intermediário e intermediário-alto, a tomografia alcança seus melhores resultados.^118,122^ Já a análise da reserva de fluxo fracionada pela tomografia vem despertando grande interesse e faculta, a partir de uma única aquisição, a avaliação da anatomia e dos dados funcionais.^117^ Sua utilidade foi comprovada em estudos randomizados e em recente metanálise, sendo suas maiores limitações práticas ainda a sua disponibilidade ampla e o aspecto econômico.^114,117,123^

Finalmente, o estudo SYNTAX-III *Revolution* (*Synergy between PCI with Taxus and Cardiac Surgery*) demonstrou que a tomografia pode auxiliar também para definir quais pacientes serão os melhores candidatos para o tratamento percutâneo ou cirúrgico, demonstrando que a inclusão da análise da reserva de fluxo fracionada é fundamental para essa finalidade. Caso estudos posteriores possam confirmar esses achados, o papel da tomografia pode ser ainda maior.^117,124^

A Tabela 11 resume as indicações da tomografia para pacientes de risco intermediário, intermediário – alto.

**Tabela 11:** Níveis de evidência para indicações de tomografia: pacientes com suspeita de doença coronariana crônica e risco pré-teste intermediário, intermediário – alto

Recomendação	Grau de recomendação	Nível de evidência
Como exame inicial para o diagnóstico ou estratificação de risco de doença coronariana obstrutiva em pacientes sem diagnóstico anatômico prévio de doença coronariana	**I**	**A**
Diagnóstico de doença coronariana em pacientes com testes funcionais de resultado incerto/conflitante, como alternativa ao estudo invasivo	**I**	**A**

#### 2.2.6. Ressonância Magnética Cardiovascular

A ressonância magnética cardiovascular (RMC) avalia múltiplos parâmetros da cardiopatia isquêmica, como detecção de isquemia, presença de fibrose/necrose por IM e determinação da viabilidade miocárdica. Demonstra também elevada acurácia e reprodutibilidade na análise da função global e segmentar biventricular, independentemente da geometria ventricular e de características do paciente. Podem-se consultar detalhes na diretriz. Na avaliação após sequela de IAM, como aneurismas e pseudoaneurismas, a RMC detecta, com precisão, volumes e geometria das câmaras cardíacas, além da área infartada, sendo, muitas vezes, método essencial na avaliação da função cardíaca e anatomia ventricular após revascularização do miocárdio.^123,125,126^ Portanto, a RMC é o método apropriado para avaliar contratilidade e função ventricular global e segmentar, sendo o padrão-ouro para essa finalidade.^80^

A RMC com estresse farmacológico apresenta propriedades técnicas que resultam em maior acurácia diagnóstica comparada à cintilografia do miocárdio e ao ecocardiograma com estresse. Sua elevada resolução espacial permite identificar defeitos de perfusão no subendocárdio.

##### 2.2.6.1. Avaliação da Isquemia Miocárdica pela RMC

A presença de isquemia no miocárdio pode ser detectada por meio da perfusão de primeira passagem sob efeito do estresse farmacológico (usualmente dipiridamol e adenosina) ou por meio da avaliação da contratilidade, através da indução de isquemia com dose escalonadas de dobutamina, sendo a primeira com maior sensibilidade, e a segunda com maior especificidade.^127^

Avaliação da perfusão miocárdica: a forma mais comum de avaliar cardiopatia isquêmica pela RMC é através da perfusão miocárdica. As imagens de perfusão são obtidas durante o estresse farmacológico e em repouso, com a infusão de gadolínio para identificar áreas hipoperfundidas. Durante a administração de agentes de estresse vasodilatadores como dipiridamol e adenosina, ocorre hiperemia significativa de toda a microcirculação coronariana. Com isso, a resistência da microcirculação se equaliza em todos os territórios miocárdicos, e a perfusão miocárdica se torna proporcional à área de secção transversal e à resistência das artérias coronárias epicárdicas.

Em territórios miocárdicos dependentes de artérias coronárias com estenoses epicárdicas limitantes de fluxo, ocorre uma diferença de perfusão miocárdica (heterogeneidade da distribuição do fluxo) entre territórios, o que, na prática clínica, denominamos de isquêmicos e não isquêmicos. Essa diferença de perfusão é identificada como defeitos de perfusão miocárdica, sendo sugestivos de estenose significativa (normalmente ≥ 50%) nos territórios relacionadas a alguma artéria coronária epicárdica, fornecendo informações importantes para o manejo e prognóstico dos pacientes.^128^ É importante destacar que essa técnica pode também detectar defeitos perfusionais miocárdicos associados à doença da microcirculação coronariana, mesmo sem doença epicárdica associada (isquemia sem obstrução das artérias coronárias, ou INOCA [*ischemia with no obstructive coronary arteries*]), em geral, com defeitos perfusionais subendocárdicos difusos ou que não respeitam territórios coronários específicos.

A RMC para isquemia compara imagens sob estresse farmacológico e em repouso, após reversão com aminofilina. Imagens de realce tardio são, em geral, analisadas junto à perfusão miocárdica, uma vez que áreas de IM prévio podem apresentar defeito perfusional e não são consideradas isquemias miocárdicas. Outro ponto importante é que isquemias miocárdicas extensas (geralmente por > 90% obstrução) podem mostrar déficit perfusional em ambas as fases (repouso e estresse). A visualização direta da fibrose miocárdica pelo realce tardio tem propriedade de tornar a perfusão miocárdica de repouso menos necessária para definição da isquemia miocárdica. Por essa razão, alguns serviços optam pelo protocolo de perfusão miocárdica somente de estresse (*stress-only*), tornando o exame mais rápido.^129,130^

A avaliação de isquemia através da perfusão do miocárdio pela RMC apresenta elevada acurácia diagnóstica, tendo sido comparada com outros métodos de diagnósticos, demonstrando, inclusive, superioridade em relação à cintilografia e a valores semelhantes à PET.^131^ O valor prognóstico da RMC também é robusto e não inferior à reserva fracionada de fluxo (*fractional flow reserve* [FFR]) invasiva, conforme demostrado recentemente (3,7% vs. 3,6%).^132^

##### 2.2.6.2. Avaliação da Contratilidade Segmentar/Reserva Contrátil pela RMC

A RMC com estresse farmacológico com dobutamina é a forma utilizada para avaliar isquemias através da contratilidade segmentar, uma vez que a utilização do exercício físico na RMC ainda enfrenta desafios para sua implementação rotineira. Nesse contexto, isquemia miocárdica é definida como déficit contrátil novo ou resposta bifásica (aumento da contratilidade em baixas doses e disfunção em altas doses). As possíveis vantagens sobre o ecocardiograma incluem alta qualidade de imagem e reprodutibilidade, sem problemas de janela acústica.^133^ Dados demonstraram alta acurácia da RMC com dobutamina para a detecção de obstruções coronarianas ≥ 50%, com sensibilidade de 81 a 84%.^134^ Além disso, a RMC com dobutamina tem valor prognóstico, indicando baixa taxa de eventos com resultados normais (< 2% em 2 anos).^135^ Limitações incluem monitorização adequada durante o exame e contraindicações da dobutamina.

A Tabela 12 apresenta os principais cenários de indicação da ressonância magnética na pesquisa de isquemia miocárdica, de acordo com a III Diretriz de RMC e tomografia computadorizada cardíaca (TCC).

**Tabela 12:** Pesquisa de DAC pela ressonância magnética – isquemia miocárdica

Indicação/Cenário clínico	Classe	Nível de evidência
Avaliação da perfusão miocárdica sob estresse farmacológico com dipiridamol/adenosina	**I**	**A**
Avaliação da contratilidade ventricular sob estresse com dobutamina	**I**	**B**
Avaliação de angina estável/equivalente anginoso em pacientes com probabilidade pré-teste intermediária de DAC	**I**	**B**
Identificação e quantificação de isquemia miocárdica em pacientes com doença arterial coronariana conhecida (exceto pacientes com anatomia de alto risco	**I**	**B**
Avaliação de pacientes com DAC não obstrutiva conhecida e/ou suspeita de INOCA	**IIa**	**C**

DAC: doença arterial coronariana; INOCA: isquemia sem obstrução das artérias coronárias (*ischemia with no obstructive coronary arteries*).

* Definida como estenose > 50% em tronco de coronária esquerda (TCE) e triarteriais com acometimento coronariano proximal.

##### 2.2.6.3. Pesquisa de Doença Arterial Coronariana pela Ressonância Magnética – Viabilidade Miocárdica

A RMC com técnica de realce tardio miocárdico (RTM) é amplamente utilizada para avaliar a viabilidade miocárdica. Embora essa técnica ajude nas avaliações diagnóstica e prognóstica, ensaios clínicos recentes vêm questionando a utilidade da investigação da viabilidade para indicar revascularização e reduzir eventos cardíacos adversos.

Recentemente, novos dados de RMC e estudos de longo prazo reforçam a importância da avaliação da viabilidade miocárdica nesse contexto.^136^ No entanto, limitações significativas de todos os estudos clínicos randomizados já publicados ainda não permitem responder, de forma definitiva, qual o valor e impacto da avaliação da viabilidade miocárdica.^137^ Dessa forma, o consenso de especialistas reforça que, em pacientes específicos e selecionados, a avaliação da viabilidade miocárdica pode ter papel crucial na decisão de revascularização e no prognóstico de longo prazo.

Entre muitos cenários clínicos, um dos clássicos que se encaixa num benefício evidente da avaliação miocárdica é o paciente com disfunção ventricular esquerda com parede anterior acinética ou discinética, permanecendo a RMC uma ferramenta importante para avaliar a viabilidade miocárdica e melhorar o prognóstico do paciente.^138–140^

##### 2.2.6.4. Aplicando a RMC para Pesquisa de Viabilidade na DAC

**Figura 14:**
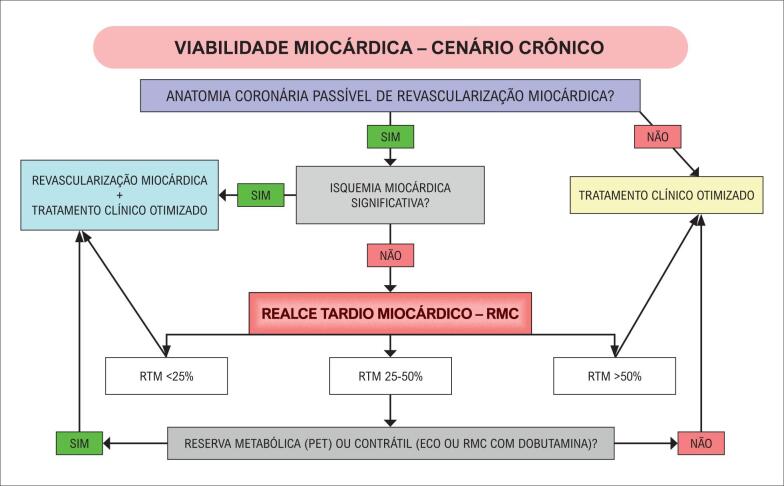
Avaliação de viabilidade miocárdica pela ressonância magnética (cenário crônico). ECO: ecocardiograma; PET: tomografia por emissão de pósitrons; RMC: ressonância magnética cardíaca; RTM: realce tardio miocárdico.

Um algoritmo proposto na III Diretriz de TC e RM da SBC (Figura 14) para o manejo de pacientes com DAC e planejamento de revascularização baseia-se na viabilidade e isquemia miocárdica. Em pacientes com disfunção crônica, a contratilidade e afilamento parietal são menos importantes, pois podem indicar hibernação miocárdica ou infarto transmural. Casos intermediários (25 a 50% de transmuralidade) podem beneficiar-se da avaliação da reserva contrátil com dobutamina ou PET.^123^

##### 2.2.6.5. Grau de Recomendação e Nível de Evidência

A Tabela 13 sumariza as principais recomendações do uso da RMC para a pesquisa de viabilidade miocárdica no contexto da DAC, sendo os detalhes encontrados na diretriz sobre o assunto de 2024, da SBC com o Colégio Brasileiro de Radiologia.^123^

**Tabela 13:** Pesquisa de DAC pela ressonância magnética – viabilidade miocárdica

Recomendação	Grau de recomendação	Nível de evidência
Detecção de infarto agudo e crônico do miocárdio pela técnica de realce tardio	**I**	**A**
Diagnóstico diferencial com etiologia não isquêmica pela técnica de realce tardio (outras CMPs)	**I**	**B**
Avaliação de viabilidade miocárdica no cenário clínico pré-revascularização pela técnica de realce tardio	**I** *	**B** *
Detectar trombo ventricular	**I**	**B**
Avaliação de aneurisma de ventrículo esquerdo	**I**	**B**
Avaliação da função ventricular regional em repouso e em estresse	**IIa**	**B**

DAC: doença arterial coronariana; CMPs: cardiomiopatias.

* Nível de evidência (NE) e grau de recomendação (GR) referentes à escolha do método de ressonância cardíaca como estratégia preferencial para pesquisa de viabilidade miocárdica. A classificação apresentada não representa NE e CR da pesquisa de viabilidade miocárdica (de maneira geral) como estratégia pré-revascularização miocárdica.

#### 2.2.7. Cateterismo Cardíaco

O cateterismo cardíaco para avaliação de pacientes com SCC permite caracterizar a anatomia coronariana, confirmar o diagnóstico e estratificar a gravidade da DAC, além de detectar diagnósticos alternativos, oferecendo parâmetros valiosos para decisões terapêuticas individualizadas.

Alguns desses parâmetros incluem quais vasos estão acometidos, a relevância desses vasos, o número e local das lesões, o envolvimento de bifurcações, o grau de estenose, o comprimento das lesões, o calibre de referência, o grau de calcificação, a presença de trombo ou de dissecção, a qualidade do leito distal, a presença e características da circulação colateral, a perviedade e grau de obstrução de stents previamente implantados ou de enxertos coronários cirúrgicos, bem como aspectos geométricos, como conformação da aorta, angulação de emergência das coronárias e tortuosidade no trajeto até as lesões, que, em conjunto, auxiliam a estimar a carga de doença, o potencial de revascularização (percutâneo e/ou cirúrgico) e os riscos para intervenções.^141^

Além da coronariografia, o cateterismo cardíaco pode incluir avaliação hemodinâmica invasiva com aferições de pressões no VE e na aorta (para mensuração das pressões de enchimento ventricular, por exemplo) e ventriculografia esquerda, permitindo avaliar a função ventricular esquerda global e segmentar, dados relevantes para auxiliar em decisões terapêuticas.

Esse exame diagnóstico é realizado no laboratório de hemodinâmica, requer anestesia local no sítio de punção, obtenção de acesso arterial (radial, ou femoral para casos específicos), cateterização seletiva das artérias coronárias e aquisição de imagens utilizando meios de contraste iodado e raios X. Embora seja um procedimento invasivo, permite definir o diagnóstico com grande acurácia e segurança, com taxas de complicações muito baixas, particularmente ao se utilizarem acesso radial, meios de contraste na-osmolar ou de baixa osmolaridade em pequeno volume e baixa exposição do paciente a radiação, possibilitada pelos angiógrafos contemporâneos.^142^

Indicações para cateterismo cardíaco em pacientes com síndromes coronárias crônicas devem, quando possível, ser precedidas pela otimização do tratamento clínico e incluem:

- Angina limitante e/ou progressiva ou redução da capacidade funcional, a despeito do tratamento clínico otimizado;
- Disfunção ventricular esquerda e/ou sintomas de IC novos;^53,143,166^
- Pacientes com isquemia miocárdica moderada a importante em testes não invasivos podem ser encaminhados para cateterismo cardíaco com intenção de revascularização, com o intuito de melhorar a capacidade funcional e/ou os sintomas anginosos; como alternativa, podem ser dirigidos inicialmente para estratégia conservadora (tratamento clínico isoladamente), desde que não apresentem disfunção ventricular esquerda importante (FEVE ≤ 35%), lesão grave no tronco da coronária esquerda (≥ 50% a angioTC) ou sintomas anginosos limitantes, mantendo a possibilidade de indicação de cateterismo cardíaco, conforme a evolução clínica do paciente.^92^

A despeito de ser considerado o padrão-ouro para o diagnóstico de DAC, a coronariografia apresenta algumas limitações. Uma delas é a definição do significado funcional de lesões coronárias intermediárias, moderadas ou ambíguas, especialmente em pacientes multiarteriais. Nesses cenários, particularmente quando não há disponibilidade ou consistência de achados de provas não invasivas, a avaliação fisiológica invasiva, através da aferição da reserva de fluxo fracionada ou de índices pressóricos coronários invasivos não hiperêmicos, deve ser considerada para auxiliar na identificação das lesões realmente limitadoras de fluxo, possibilitando reclassificar o paciente e simplificar ou mesmo evitar intervenções, com segurança.^143,144,146^ Mais recentemente, ferramentas de avaliação de fluxo baseadas nas imagens da coronariografia, sem a necessidade de utilizar fios-guias ou sensores pressóricos invasivos, foram desenvolvidas e validadas, demonstrando benefícios clínicos em sua aplicação.^147^

Uma outra limitação relevante da coronariografia, por se tratar de um luminograma, refere-se à paucidade de informações reveladas sobre as placas ateroscleróticas coronarianas. Para suprir essa limitação, ferramentas de imagem intravascular, como ultrassom intracoronário ou tomografia de coerência óptica, podem ser muito úteis no laboratório de hemodinâmica. Elas permitem caracterizar as placas geométrica e morfologicamente, determinando área luminal mínima da lesão, carga de placa, grau de remodelamento vascular, carga e distribuição de cálcio, lipídio e fibrose, identificação de fenótipos de placa associados à vulnerabilidade, bem como detecção de trombos e acidentes de placa (rotura, ulceração e nódulos calcificados).

Para lesões no tronco da coronária esquerda ou em anatomias em que a interpretação da avaliação fisiológica invasiva é limitada, como em lesões coronárias em série ou na presença de ponte miocárdica, o ultrassom intracoronário está validado clinicamente para deferir intervenções com segurança, apoiando-se em referências de área luminal mínima. A principal aplicação da imagem intravascular, entretanto, é guiar intervenções coronárias percutâneas complexas, em que proporciona melhor prognóstico e reduz a mortalidade. Em pacientes com SCC com falência de stent, a imagem intravascular é fundamental para esclarecer os mecanismos (hipoexpansão, dissecção nas bordas, cobertura incompleta da placa, subdimensionamento do calibre da prótese, proliferação neointimal, malaposição, encurtamento longitudinal ou fraturas das hastes) e direcionar o tratamento.^148–150^

Finalmente, há pacientes com SCC que apresentam sintomas anginosos e isquemia em provas funcionais não invasivas, que são encaminhados a angioTC coronária ou ao cateterismo cardíaco e não apresentam lesões obstrutivas significativas (> 50%). Esse cenário, conhecido como INOCA, relaciona-se à angina vasoespástica e à disfunção microvascular coronariana, podendo coexistir com a doença aterosclerótica, e seu diagnóstico tem implicações prognósticas e terapêuticas. A detecção de disfunção microvascular coronariana requer, além do cateterismo cardíaco, a avaliação fisiológica invasiva, para afastar a presença de obstruções epicárdicas funcionalmente significativas (FFR > 0,80), além de confirmar redução na RFC (< 2,0), espasmo microvascular ao teste de acetilcolina (angina, alteração eletrocardiográfica, sem espasmo epicárdico) ou alteração no índice de resistência microvascular (IMR ≥ 25) coronariana.^151–153^

## 3. Estratégias Clínicas para Avaliar o Risco Cardiovascular e a Estratificação da Doença Aterosclerótica Coronariana

Pacientes com DAC estável apresentam risco heterogêneo de eventos cardiovasculares maiores, especialmente morte e infarto. Por isso, é essencial realizar a estratificação de risco com base em dados clínicos e exames complementares.^80^

A avaliação inicial deve incluir história clínica, ecocardiograma para avaliação da FEVE e, quando indicado, exames não invasivos para a detecção de isquemia ou avaliação anatômica da árvore coronária. A partir dessas informações, os pacientes podem ser classificados em risco baixo (< 1% ao ano), intermediário (1–3%) ou alto (> 3%) de morte cardiovascular ou infarto não fatal.^80^

### 3.1. Avaliação Inicial do Risco

Alguns achados clínicos indicam maior risco: angina de início recente ou em crescendo, IC de provável causa isquêmica ou histórico prévio de SCA. O escore TRA 2°P, derivado de coorte de prevenção secundária,^154^ estima o risco cardiovascular com base em nove variáveis: idade, diabetes, hipertensão, tabagismo, doença arterial periférica, AVC prévio, cirurgia de revascularização miocárdica, IC e disfunção renal.^155^

Fatores socioeconômicos e acesso ao sistema de saúde também influenciam o prognóstico. Exames simples, como ECG e radiografia de tórax, ajudam na estratificação inicial. O ECG pode detectar infartos prévios ou arritmias, sendo útil mesmo em pacientes assintomáticos.^156^ O uso de inteligência artificial aplicada ao ECG tem mostrado potencial na predição de eventos cardiovasculares.^157^

A radiografia de tórax pode evidenciar cardiomegalia, aneurisma de VE e congestão pulmonar, todos associados a maior risco de eventos.

#### 3.1.1. Marcadores Laboratoriais de Risco na Doença Coronariana Crônica

A troponina cardíaca, mesmo em concentrações discretamente elevadas, é um marcador sensível de lesão miocárdica e se associa ao risco cardiovascular, mesmo na ausência de infarto agudo. Ensaios de alta sensibilidade permitiram detectar níveis subclínicos de troponina em diversas condições não isquêmicas, como insuficiência renal, sepse e embolia pulmonar.^41^

O estudo ARIC demonstrou que elevações de troponina estão associadas a maior incidência de eventos cardiovasculares, independentemente dos fatores de risco tradicionais.^43^ Em pacientes com DAC estável, níveis persistentemente elevados de troponina de alta sensibilidade estão correlacionados a maior risco de morte cardiovascular ou infarto não fatal.^112^ Concentrações acima de 10 ng/L identificam indivíduos com risco 50% maior de eventos, mesmo após ajuste para gravidade da DAC.

Outro marcador prognóstico importante é o BNP, liberado em resposta à sobrecarga de volume e estresse de parede. Em uma coorte com mais de 13 mil pacientes com DAC crônica, o NT-proBNP foi preditor independente de desfechos adversos, junto à troponina e ao LDL-C.^113^

Com base nesses achados, foi desenvolvido o escore ABC-CHD, que utiliza biomarcadores (troponina, NT-proBNP, LDL-C) combinados a dados clínicos como diabetes, tabagismo e DAP para estimar o risco cardiovascular individual.^158^

#### 3.1.2. TE para Avaliação do Prognóstico da DAC Estável

O TE fornece dados prognósticos importantes em pacientes com DAC estável. A presença de depressão do segmento ST em baixa carga, sintomas durante o esforço (angina ou dispneia), baixa capacidade funcional, arritmias ventriculares complexas e resposta pressórica anormal são indicadores de alto risco.^66^

Por outro lado, pacientes que superam o terceiro estágio do protocolo de Bruce apresentam mortalidade anual < 1%. Aqueles que não alcançam 5 METs têm mortalidade anual estimada em 5%.^159^ A resposta cronotrópica inadequada, queda da PAS durante o esforço, depressão do ST em múltiplas derivações ou persistente na recuperação (> 5 minutos) também indicam risco elevado.^160,161^

O Escore de Duke^48^ é uma ferramenta validada para a estratificação prognóstica com base em variáveis clínicas e ergométricas. A equação é:


Tempo de exercício (min)−5×depressão do ST (mm)−4× índice anginoso (1:sem angina; 2:com angina;3:angina limitante)

Classificação:

- Escore ≥ +5: baixo risco (mortalidade anual ≤ 1%);
- Escore entre –10 e +4: risco intermediário (1–3%);
- Escore < –10: alto risco (> 3%).

O TE deve ser usado preferencialmente em pacientes com capacidade funcional preservada e ECG basal interpretável.

#### 3.1.3. Ecocardiografia Transtorácica (Repouso e sob Estresse)

A FEVE é um dos mais importantes marcadores prognósticos na DAC estável.^162^ A ecocardiografia permite avaliar a função ventricular em repouso e detectar isquemia miocárdica sob estresse (físico ou farmacológico), com valor prognóstico robusto.

A presença de isquemia induzida durante o estresse ecocardiográfico — evidenciada por hipocinesia ou acinesia em segmentos previamente normais — está associada a maior risco de eventos cardiovasculares. A extensão da isquemia está diretamente relacionada ao risco: alterações em ≥ 3 segmentos (entre os 16 avaliados) identificam pacientes com risco anual > 3%.^163^

Por outro lado, a ausência de isquemia no ecocardiograma sob estresse indica risco baixo de eventos, semelhante ao de cintilografia de perfusão normal (< 1% ao ano).^164^ A técnica é particularmente útil em pacientes com história de infarto, permitindo identificar viabilidade miocárdica e extensão de necrose.

A ecocardiografia de estresse tem boa acurácia diagnóstica e é útil tanto na avaliação funcional quanto na decisão terapêutica, com impacto prognóstico validado por grandes estudos observacionais e consensos internacionais.^65,165–169^

#### 3.1.4. Cintilografia de Perfusão Miocárdica

A NA é um dos métodos mais utilizados para avaliação funcional na DAC estável. Ela fornece informações prognósticas sobre a extensão da isquemia, função ventricular, captação pulmonar e remodelamento ventricular induzido pelo estresse.^170^ A presença de defeito reversível de perfusão ≥ 10% da massa do VE está associada a risco elevado de eventos cardiovasculares (mortalidade ou infarto > 3% ao ano).^171^ Já um exame normal prediz risco anual < 1%, com alto valor preditivo negativo.^164^

A NA também avalia a fração de ejeção e o volume ventricular, parâmetros que influenciam o risco. A presença de captação pulmonar aumentada ou dilatação transitória do VE durante o estresse também indica maior risco de eventos.^172–174^

Diversos estudos multicêntricos validaram a acurácia da NA na estratificação de risco, incluindo pacientes com DAC conhecida, suspeita de isquemia ou após infarto prévio. É uma ferramenta amplamente aceita por sua robustez, aplicabilidade clínica e valor prognóstico bem estabelecido.^44,175,176^

#### 3.1.5. Ressonância Magnética Cardíaca

A ressonância magnética cardíaca (RMC) é um exame versátil que avalia, simultaneamente, anatomia, função, perfusão e viabilidade miocárdica. Em pacientes com DAC estável, fornece informações prognósticas comparáveis às de outros testes funcionais.^177^

Uma RMC de estresse negativa está associada a taxa anual de eventos < 1%.^178^ Já a presença de isquemia induzível e/ou fibrose miocárdica (realce tardio por gadolínio) está correlacionada a maior mortalidade e risco de infarto, independentemente da fração de ejeção ventricular.^1^

Estudos mostram que o número de segmentos com isquemia ou cicatriz miocárdica prediz eventos adversos, mesmo em pacientes com função ventricular aparentemente preservada.^179–181^ A detecção de fibrose subclínica é particularmente útil na identificação de pacientes em risco elevado, mesmo na ausência de sintomas.

Uma metanálise recente, envolvendo mais de 67 mil pacientes, confirmou que tanto a isquemia quanto o realce tardio na RMC são fortes preditores de desfechos cardiovasculares.^44^ A RMC deve ser considerada quando se busca avaliação detalhada da extensão da doença e do risco prognóstico.

#### 3.1.6. Angiotomografia de Coronárias

A angioTC das artérias coronárias (angioTC coronária) é uma ferramenta eficaz na estratificação de risco de pacientes com DAC estável. Ela permite avaliar não apenas a presença de estenoses, mas também a carga e composição das placas ateroscleróticas.^182^

A quantificação do volume total de placas coronárias, especialmente as de baixo realce e com remodelamento positivo, está associada a maior risco de eventos cardiovasculares adversos.^183^ A angioTC também permite a identificação de placas de alto risco, como as com núcleo necrótico ou com sinal do "anel de *napkin*".^184^

Estudos demonstram que a angioTC é superior à avaliação anatômica isolada, pois integra achados morfológicos e funcionais. A presença de placas de alto risco ou estenose significativa em segmentos proximais está associada a risco cardiovascular elevado em até 10 anos.^185^

A associação entre angioTC e algoritmos de pontuação integrados, como o *Comprehensive Atherosclerotic Risk Score*, melhora a predição de desfechos, orientando estratégias de prevenção mais intensivas.^44^

Na Tabela 14, são listadas algumas das definições de alto risco de eventos cardiovasculares por modalidade diagnóstica em pacientes com SCC.

**Tabela 14:** Definições de alto risco de eventos cardiovasculares por modalidade diagnóstica em pacientes com síndrome coronariana crônica

Modalidade diagnóstica	Definição de alto risco
Teste ergométrico	Escore de Duke < –10, correspondente a mortalidade cardiovascular > 3% ao ano
Cintilografia de perfusão miocárdica	Isquemia reversível ≥ 10% da massa do ventrículo esquerdo
Ecocardiografia de estresse	Alterações de contratilidade induzidas em ≥3 de 16 segmentos do ventrículo esquerdo
Ressonância magnética cardíaca	Isquemia induzida ≥ 2 segmentos ou ≥ 3 segmentos disfuncionais à dobutamina
AngioTC coronária/cinecoronariografia	Estenose de tronco de coronária esquerda, DAC de três vasos com lesões proximais ou DA proximal

AngioTC: angiotomografia; DA: descendente anterior; DAC: doença arterial coronariana.

#### 3.1.7. Resumo com Sugestão de como Investigar com Métodos Diagnósticos

A estimativa da PPT de DAC obstrutiva é essencial na escolha da estratégia diagnóstica inicial. Essa estimativa deve considerar idade, sexo e tipo de dor torácica (angina típica, atípica ou não cardíaca), além de fatores de risco e comorbidades relevantes.^18^

A diretriz da ESC de 2019 atualizou os valores de PPT baseados em grandes registros contemporâneos, reduzindo as estimativas inflacionadas dos modelos anteriores (como o de Diamond-Forrester).^1^ Pacientes com PPT < 5% não requerem investigação adicional. Aqueles entre 5 e 15% devem ser avaliados individualmente. PPT entre 15 e 85% indica a necessidade de testes funcionais ou anatômicos, e > 85% geralmente permite diagnóstico clínico de DAC.

A nossa sugestão é que se utilize o modelo de estimativa da PPT com o qual o avaliador esteja mais familiarizado, considerando a limitação de cada um deles e valorizando, também, a percepção pessoal (apesar de totalmente dependente da experiência clínica acumulada), visto que não há uma definição clara de qual é o modelo ideal para avaliação da população brasileira. No Quadro 7, é apresentada uma análise comparativa entre modelos de estimativa de PPT de DAC obstrutiva.^1,3,18,119,186^

**Quadro 7:** Comparação entre modelos de estimativa de probabilidade pré-teste (PPT) de DAC obstrutiva

Modelo	Ano/Origem	Variáveis consideradas	Características	Pontos fortes	Limitações
Diamond-Forrester (clássico)	1979 – EUA	Idade, sexo, tipo de dor torácica (típica, atípica ou não anginosa)	Modelo original para estimar PPT de DAC em população sintomática	Simples, rápido, historicamente influente	Superestima a PPT nas populações atuais; baseado em dados da década de 1970
Modified Diamond-Forrester (PROMISE Trial)	2018 – EUA/Europa	Idade, sexo, tipo de dor torácica	Recalibrado com dados do estudo PROMISE, mais representativo de populações atuais	Melhora a acurácia do modelo clássico em populações contemporâneas	Ainda limitado a três variáveis; sem outros fatores de risco associados
CAD Consortium Basic Model	2012 – Europa	Idade, sexo, tipo de dor torácica	Derivado de grande coorte europeia, usado nas diretrizes ESC 2013	Reprodutível; base para diretrizes anteriores	Ainda tende a superestimar em pacientes com sintomas atípicos
CAD Consortium Clinical Model (Genders et al.)	2012 – Europa	Idade, sexo, tipo de dor, tabagismo, diabetes, hipertensão	Modelo clínico completo, incorporado nas diretrizes ESC 2019	Mais acurado; considera fatores de risco adicionais	Requer mais informações; menos usado em serviços com limitações de dados clínicos
ESC 2019 Recalibrado	2019 – ESC Guidelines	Idade, sexo, tipo de dor, inclusão de dispneia como sintoma	Recalibrado com base em grandes registros europeus modernos	Mais próximo da realidade atual; PPT geralmente mais baixa; inclui "dispneia isolada"	Pode subestimar risco em pacientes com fatores de risco importantes não incluídos
Percepção Clínica (Julgamento Médico)	–	Avaliação subjetiva baseada na experiência e no contexto clínico	Complementa os modelos matemáticos quando há incerteza ou contexto não contemplado pelos modelos	Importante em sistemas de saúde heterogêneos, como o brasileiro	Alta variabilidade; depende da experiência individual

DAC: doença arterial coronariana; ESC: Sociedade Europeia de Cardiologia (European Society of Cardiology); EUA: Estados Unidos da América; PPT: probabilidade pré-teste.

A escolha do exame inicial deve levar em conta:

- **Capacidade funcional:** se preservada, iniciar com TE ou ecocardiograma de estresse;
- **ECG basal interpretável:** se não for interpretável, preferir métodos de imagem (ecocardiograma, cintilografia, RMC);
- **Acesso local e disponibilidade:** considerar custo, tempo de realização e familiaridade da equipe;
- **DRC ou alergia a contraste:** pode haver restrição para realização de angioTC.

**Figura 15:**
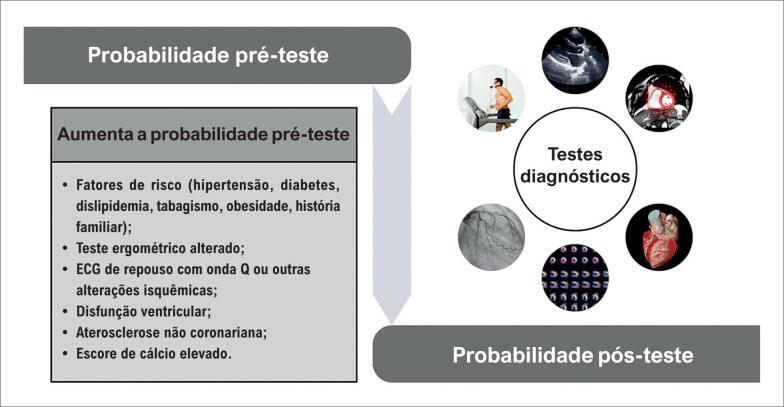
Tomada de decisão em pacientes sintomáticos com suspeita de SCC. ECG: eletrocardiograma.

**Figura 16:**
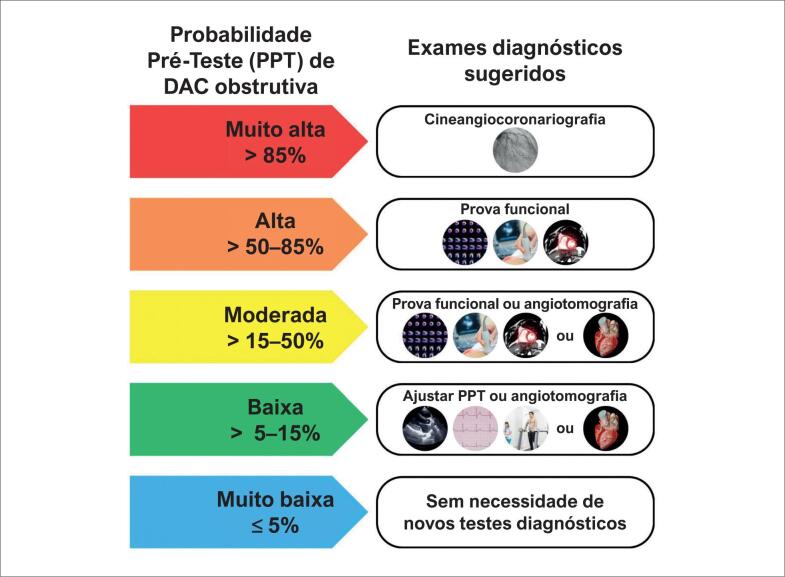
Exames diagnósticos de acordo com a probabilidade pré-teste de DAC obstrutiva. DAC: doença arterial coronariana.

A Figura 15 exemplifica o processo de tomada de decisão em pacientes sintomáticos com suspeita de SCC. Na Figura 16, estão listados os exames diagnósticos sugeridos de acordo com a PPT de DAC obstrutiva.

## 4. Tratamento Clínico-Farmacológico para Redução do Risco de Eventos

### 4.1. Terapia Antitrombótica nas Síndromes Coronarianas Crônicas

#### 4.1.1. Aspirina

Aspirina (ácido acetilsalicílico – AAS) é um inibidor irreversível da enzima ciclo-oxigenase (COX) e da síntese de tromboxano A2 (TXA2). Sua maior seletividade pela COX-1 em relação à COX-2 também justifica seu efeito antitrombótico predominante.^187^ É utilizada na dose de 75–100 mg/dia, e seu benefício foi comprovado em pacientes com DAC, com uma redução significativa da taxa de eventos cardiovasculares maiores de aproximadamente 21% em pacientes com infarto prévio (redução relativa de risco) e de 37% nos demais.^188,189^

#### 4.1.2. Inibidores do Receptor P2Y12

São representados pelo clopidogrel e prasugrel, tienopiridínicos que bloqueiam irreversivelmente o receptor plaquetário P2Y12, e o ticagrelor, um bloqueador reversível que não necessita de ativação metabólica. O estudo CAPRIE (*Clopidogrel vs. Aspirin in Patients at Risk of Ischaemic Events*) mostrou um discreto benefício do clopidogrel em relação à AAS, com redução das taxas de infarto, AVC e morte cardiovascular (5,32% vs. 5,83%, redução do risco relativo [RRR] 8,7% [0,3–16,5], p = 0,043), sendo que uma análise de subgrupo mostrou que esse benefício só foi significativo nos pacientes com doença arterial periférica.^190^ Seu metabólito ativo é formado por ação do citocromo P2C19, de tal forma que pacientes com perda de função do gene que codifica essa enzima podem ter uma menor resposta a essa medicação.^191^ Além disso, medicações que inibem esse citocromo, como o omeprazol, também reduzem o efeito do clopidogrel.^192^

Prasugrel e ticagrelor têm um efeito mais rápido e previsível, uma vez que não dependem da metabolização do citocromo P2C19.^193^ O prasugrel se mostrou superior ao clopidogrel em pacientes com SCA submetidos à angioplastia, mas com um aumento da taxa de sangramento em pacientes com AVC prévio e ausência de benefício em pacientes com idade > 75 anos ou peso < 60 kg.^194,195^ O ticagrelor, por sua vez, além do benefício comprovado na SCA, mostrou reduzir eventos cardiovasculares em pacientes com DAC em uso de aspirina e história de infarto nos últimos 3 anos, às custas de maior taxa de sangramento não fatal.^195^ Um evento adverso importante do ticagrelor é a dispneia, que, na maior parte das vezes, é leve e autolimitada.^196^

#### 4.1.3. Dupla Terapia Antiplaquetária

A dupla terapia antiplaquetária (*dual antiplatelet therapy* [DAPT]) com AAS e clopidogrel mostrou reduzir a taxa de eventos cardiovasculares em pacientes com SCA em até 1 ano, quando comparada à monoterapia com AAS.^197^ Ainda no contexto agudo, a DAPT com AAS + ticagrelor (para todas SCA) ou AAS + prasugrel (para apenas SCA com angioplastia) mostrou reduzir eventos em relação ao clopidogrel.^197^ Mais recentemente, o estudo ISAR-REACT (*Intracoronary Stenting and Antithrombotic Regimen: Rapid Early Action for Coronary Treatment*), um ensaio clínico randomizado *open-label*, mostrou aparente superioridade do prasugrel em relação ao ticagrelor em pacientes com SCA submetidos à angioplastia.^198^

Em relação à doença coronariana crônica (DCC), o estudo CHARISMA (*Clopidogrel for High Atherothrombotic Risk, Ischemic Stabilization, Management, and Avoidance*) randomizou 19.185 pacientes em uso de AAS, para associação com clopidogrel vs. Placebo, mostrando uma redução da taxa de eventos cardiovasculares no grupo clopidogrel, porém não significativa (razão de risco [HR] 0,93; intervalo de confiança de 95% [IC 95%] 0,83–1,05; p = 0,22).^199^ Posteriormente, uma análise de subgrupo desse estudo mostrou benefício apenas nos pacientes com infarto prévio, AVC ou DAP (HR 0,77; IC 95% 0,61–0,98; p = 0,031).^200^ Já o estudo DAPT comparou o uso de tienopiridínicos (65% clopidogrel, 35% prasugrel) associados à AAS por 30 meses vs. 12 meses após angioplastia, mostrando uma redução significativa de ECAM em pacientes com infarto prévio apenas (HR 0,56; IC 95% 0,42–0,76; p < 0,001).^201^

No entanto, a maior quantidade de evidência nesse cenário concentra-se no uso do ticagrelor associado à aspirina. O estudo PEGASUS (*The Prevention of Cardiovascular Events in Patients with Prior Heart Attack Using Ticagrelor Compared with Placebo on a Background of Aspirin*) mostrou que a DAPT com essas medicações, quando comparada a AAS + placebo, foi capaz de reduzir significativamente os MACE em pacientes com infarto prévio associado a outro fator de risco, como idade > 65 anos, diabetes, doença renal crônica (DRC) e doença multiarterial (HR 0,84; IC 95% 0,74–0,95; p = 0,008).^202^ Já o estudo THEMIS (*THE effect of ticagrelor on health outcomes in diabetes Mellitus patients Intervention Study*) comparou a DAPT com aspirina + ticagrelor em pacientes com diabetes sem infarto prévio, mostrando uma redução de eventos após seguimento médio de 40 meses (HR 0,90; IC95% 0,81–0,99; p = 0,04), mas às custas de um aumento significativo da taxa de sangramento maior (HR 2,32; IC 95% 1,82–2,94; p < 0,001).^203^

#### 4.1.4. Anticoagulação em Dose Baixa

Nesse cenário, o estudo COMPASS (*The Cardiovascular Outcomes for People Using Anticoagulation Strategies Study*) avaliou 27.395 pacientes com DAC e alto risco cardiovascular, que foram randomizados em três grupos: 1) AAS 100 mg; 2) rivaroxabana 5 mg; ou 3) AAS 100 mg + rivaroxabana 2,5 mg. Os participantes deveriam ter aterosclerose manifesta em pelo menos dois territórios vasculares ou pelo menos dois fatores de risco (tabagismo ativo, diabetes, filtração glomerular < 60 mL/min, IC ou AVC isquêmico há menos de 1 mês). Quando comparada à monoterapia com aspirina, a associação aspirina + rivaroxabana 2,5 mg mostrou redução significativa na taxa de eventos cardiovasculares em 23 meses de seguimento (HR 0,76; IC 95% 0,66–0,86; p < 0,001).^204^

#### 4.1.5. Anticoagulação a Longo Prazo

Inúmeros estudos têm comprovado o benefício da anticoagulação a longo prazo em pacientes com fibrilação atrial (FA) para a prevenção de eventos embólicos. Por exemplo, uma metanálise com 28.044 pacientes mostrou que a redução do risco relativo de AVC isquêmico desses pacientes é de aproximadamente 62%.^205^ No entanto, no subgrupo de pacientes com FA e DAC, que também fazem uso crônico de antiplaquetários, ainda não está claro qual é a melhor estratégia a ser utilizada.

Recentemente, o estudo AFIRE (*Atrial Fibrillation and Ischaemic Events with Rivaroxaban in Patients with Stable Coronary Artery Disease*) avaliou 2.236 pacientes com FA e DAC (35% com infarto prévio), randomizados para receber rivaroxabana isoladamente ou associada a aspirina/clopidogrel. Após um tempo médio de seguimento de 24 meses, a monoterapia com rivaroxabana mostrou-se não inferior na prevenção de AVC, eventos embólicos, infarto ou angina instável (HR 0,72; IC 95% 0,55–0,95). No entanto, a quantidade de pacientes com infarto prévio foi significativamente superior no grupo da monoterapia.^206^

#### 4.1.6. Antiplaquetários no Contexto da Intervenção Coronariana Percutânea (ICP)

Sabe-se que a DAPT reduz o risco de trombose de stent em pacientes submetidos a angioplastia eletiva e que deve ser mantida por apenas 6 meses após o procedimento, e não 12 meses como na SCA. O maior estudo nessa área é o ISAR-SAFE (*Intracoronary Stenting and Antithrombotic Regimen: Safety and Efficacy of 6 Months Dual Antiplatelet Therapy After Drug-Eluting Stenting*), que avaliou 4.005 pacientes com DAC submetidos a angioplastia (50% de forma eletiva), mostrando que o grupo que recebeu DAPT por 6 meses apresentou uma taxa não inferior de morte, infarto, trombose de stent, AVC ou sangramento, em comparação ao grupo que manteve a medicação por 12 meses (1,5%, IC 95% 0,9–2,0 vs. 1,6%, IC 95% 1,1–2,2, p < 0,001).^207^

Já em relação a outros antiplaquetários, como o prasugrel e ticagrelor, ainda não existe evidência de que eles sejam superiores ao clopidogrel no contexto da angioplastia eletiva. O estudo TWILIGHT (*Ticagrelor With Aspirin or Alone in High-Risk Patients after Coronary Intervention*) avaliou pacientes em uso de DAPT por 3 meses após angioplastia eletiva, mostrando que aqueles que continuaram com ticagrelor até completar 12 meses apresentaram menos sangramento do que o grupo que manteve DAPT (HR 0,56; IC 95% 0,45–0,68; p < 0,001).^208^

#### 4.1.7. Anticoagulantes no Contexto da Intervenção Coronariana Percutânea (ICP)

O estudo WOEST (*What is the Optimal Antiplatelet and Anticoagulant Therapy in Patients with Oral Anticoagulation and Coronary Stenting?*) foi o primeiro a comprovar que a combinação de clopidogrel com anticoagulante em pacientes submetidos a angioplastia eletiva reduziu a taxa de sangramentos, em comparação à terapia tripla com AAS + clopidogrel + anticoagulante.^209^ Nos anos seguintes, vários estudos comprovaram essa teoria. O maior deles, o AUGUSTUS (*Aspirin Placebo in Patients with Atrial Fibrillation and Acute Coronary Syndrome or Percutaneous Coronary Intervention*) randomizou 4.614 pacientes com FA submetidos a angioplastia de urgência ou eletiva, mostrando uma menor incidência de eventos hemorrágicos quando a apixabana foi associada a clopidogrel, em comparação à terapia tripla com AAS + clopidogrel + varfarina (HR 1,89; IC 95% 1,59–2,24; p < 0,001) (Tabela 15).^210^

**Tabela 15:** Recomendações para o Uso de Anticoagulantes no Contexto da Intervenção Coronariana Percutânea

Recomendação	Grau de recomendação	Nível de evidência
AAS 100mg/dia é recomendado para pacientes com infarto prévio ou pacientes submetidos à revascularização miocárdica com ou sem infarto prévio	**I**	**A**
Clopidogrel 75 mg/dia é recomendado como alternativa à aspirina em pacientes com intolerância. Pode ser uma alternativa ao AAS com efeito no mínimo similar para DAC ou em pacientes com doença arterial periférica ou antecedente de AVC isquêmico.	**I**	**B**
Clopidogrel pode ser uma alternativa ao AAS com efeito no mínimo similar para DAC ou em pacientes com doença arterial periférica ou antecedente de AVC isquêmico.	**IIa**	**B**
AAS 100 mg/dia pode ser considerado em pacientes com DAC sem infarto prévio ou revascularização.	**IIb**	**C**
DAPT por longo prazo pode ser considerada em pacientes com alto risco de eventos cardiovasculares e baixo risco de sangramento.	**IIa**	**A**
AAS 100 mg/dia é recomendada para pacientes submetidos a ICP.	**I**	**A**
Clopidogrel 75 mg/dia é recomendado para pacientes submetidos a ICP por pelo menos 6 meses após o procedimento.	**I**	**A**
Clopidogrel 75 mg/dia pode ser considerado para pacientes submetidos a ICP por pelo menos 3 meses após o procedimento, se elevado risco de sangramento.	**IIa**	**A**
Pacientes com indicação de anticoagulação após ICP devem, preferencialmente, fazer uso de DOAC associado a terapia antiplaquetária.	**I**	**A**
Pacientes com indicação de anticoagulação após ICP podem ter o AAS suspenso após 1 semana, mantendo apenas o anticoagulante e clopidogrel, se o risco de trombose for baixo.	**IIa**	**B**
Pacientes com indicação de anticoagulação após ICP podem manter terapia tripla com aspirina + clopidogrel + anticoagulante por pelo menos 1 mês, se o risco de trombose for muito alto.	**IIa**	**C**
Pacientes com indicação de anticoagulação após ICP podem receber terapia dupla com anticoagulante + prasugrel ou ticagrelor, em substituição à terapia tripla, se o risco de sangramento for elevado.	**IIb**	**C**
Prasugrel e ticagrelor não são recomendados como terapia tripla em associação a AAS e anticoagulante.	**III**	**C**

AAS: ácido acetilsalicílico; AVC: acidente vascular cerebral; DAC: doença arterial coronariana; DAPT: dupla terapia antiplaquetária; DOAC: anticoagulante oral direto (direct oral mpacto fante); ICP: intervenção coronariana percutânea.

### 4.2. Manejo Lipídico na Síndrome Coronária Crônica

Em pacientes com doença coronariana estabelecida, classificados como muito alto risco cardiovascular, as alterações lipídicas devem ser tratadas com terapia farmacológica associada a intervenções no estilo de vida. Exercício, dieta, e controle do peso devem ser recomendados para todos os pacientes.

#### 4.2.1. Estatinas e Outras Drogas que Atuam no LDL-C

A relação causal entre o LDL-C e o risco de DCVs tem sido muito bem demonstrada em estudos epidemiológicos, genéticos (randomização mendeliana) e ensaios clínicos randomizados;^110^ dessa maneira, o principal alvo terapêutico é o LDL-C. Por causa disso, novas alternativas terapêuticas estão surgindo.

Publicações recentes, em que foi testada a associação de estatinas e inibidores da PCSK9, têm demonstrado que níveis progressivamente mais baixos de LDL-C estão associados a contínua melhora no fenótipo da placa aterosclerótica,^211^ bem como redução de eventos cardiovasculares.^212^ Sendo assim, é recomendada, nesta diretriz, a abordagem que busca metas de LDL-C para reduzir o risco aterosclerótico.

Nos indivíduos com SCC, deve ser buscada a meta de LDL-C abaixo de 50 mg/dL, associada a uma redução superior a 50% dos níveis basais de LDL-C.^213^ A recomendação da redução percentual adicional é baseada na observação de que, mesmo aqueles indivíduos que têm LDL-C abaixo de 100 mg/dL no momento da primeira manifestação de doença coronariana terão grande benefício com a significativa redução dos níveis lipídicos. Uma meta terapêutica mais baixa, menor que 40 mg/dL, pode ser considerada em pacientes que tenham apresentado um segundo evento cardiovascular no período de até 2 anos após o primeiro.^214^

Considerando as inúmeras publicações que demonstraram consistentemente uma diminuição entre 20 e 25% do risco de eventos cardiovasculares recorrentes com a redução de cerca de 39 mg/dL de LDL-C, a primeira linha de tratamento são as estatinas potentes. Diversos estudos e metanálises demostraram maior benefício clínico quando foram utilizadas estatinas mais potentes vs. Estatinas menos potentes; da mesma maneira, doses maiores de uma mesma estatina foram mais eficazes que doses menores.^215^ Embora as estatinas potentes sejam capazes de reduzir o LDL-C em torno de 50%, para a obtenção das metas, frequentemente será necessária a associação de diferentes medicamentos.

A adição de ezetimiba às estatinas tem demonstrado diminuir adicionalmente o LDL-C entre 18 e 25%. O estudo IMPROVE-IT (*Improved Reduction of Outcomes: Vytorin Efficacy International Trial*) mostrou redução de eventos cardiovasculares em indivíduos após SCA que receberam a associação de sinvastatina e ezetimiba quando comparada ao uso isolado de estatina.^216^ Esse medicamento, embora eficaz e seguro, ainda é pouco utilizado nos pacientes de muito alto risco cardiovascular.

Ensaios clínicos publicados desde 2015 têm demonstrado que anticorpos monoclonais contra a PCSK9 são seguros e eficazes. Através da degradação extra-hepática da PCSK9, eles permitem a maior reciclagem dos receptores do LDL-C para atuar na superfície hepática. Inibidores da PCSK9 (evolocumabe e alirocumabe) reduzem o LDL-C em torno de 60%, mesmo naqueles pacientes tratados com estatinas. O estudo FOURIER (*Further Cardiovascular Outcomes Research with PCSK9 Inhibition in Subjects with Elevated Risk*) avaliou pacientes com DCV estabelecida. No seguimento médio de apenas 2,2 anos, os pacientes tratados com estatina + evolocumabe atingiram um LDL-C mediano de 30 mg/dL e tiveram redução do desfecho cardiovascular composto de 15% na comparação com aqueles que utilizaram apenas estatinas.^212^ Durante o período do estudo, não houve aumento no risco de efeitos adversos naqueles que ficaram com níveis baixos de LDL-C.

Mais recentemente publicado, o estudo FOURIER-OLE (*Further Cardiovascular Outcomes Research With PCSK9 Inhibition in Subjects With Elevated Risk – Open-Label Extension*) buscou avaliar a segurança de manter o LDL-C baixo durante um tempo maior, com a associação de estatina e evolocumabe. Nesse estudo, 6.559 pacientes que haviam sido incluídos no estudo FOURIER receberam evolocumabe pelo período médio de 5 anos (metade deles já havia utilizado estatina associada a evolocumabe por 2,2 anos, e a outra metade havia recebido apenas estatina). Na avaliação anual durante os 5 anos, não foi observado aumento de eventos adversos;^217^ em outra análise do mesmo estudo, recentemente publicada, verificou-se que, de acordo com o estrato de LDL-C atingido, mesmo aqueles que ficaram com LDL-C abaixo de 20 mg/dL não apresentaram maior taxa de eventos adversos.^218^ Em análise exploratória foi demonstrado que aqueles pacientes que já haviam recebido evolocumabe no estudo FOURIER original e que continuaram recebendo essa associação no FOURIER-OLE apresentaram redução de morte cardiovascular quando comparados com aqueles que passaram a receber evolocumabe apenas no segundo estudo. Esse achado foi atribuído ao "efeito legado" de iniciar mais precocemente a intensificação da redução lipídica.

Apesar do benefício clínico e segurança observados com o uso de inibidores da PCSK9 associado a estatinas e ezetimiba, seu uso em larga escala ainda é limitado pelo custo elevado.

Uma abordagem alternativa voltada para a PCSK9 e consequente redução do LDL-C consiste em interferência de RNA.^219^ Inclisirana é um pequeno interferente no RNA projetado para atingir o RNA mensageiro da PCSK9 e, então, inibir a síntese da PCSK9. Portanto, diferentemente dos anticorpos monoclonais contra a PCSK9, a inclisirana inibe a produção hepática da PCSK9 (intracelularmente). Uma grande virtude é que a droga, em sua formulação atual, é eficaz quando administrada subcutaneamente a cada 3 a 6 meses.

Esse medicamento tem sido comercializado desde 2020 na União Europeia e desde 2021 nos Estados Unidos. Recentemente, três ensaios de fase 3 da inclisirana foram publicados. Em todos esses estudos, aproximadamente 90% dos pacientes foram tratados com estatina. Inclisirana 300 mg ou placebo foi administrado na fase inicial, após 3 meses e a cada 6 meses depois. No estudo ORION-9, foram incluídos 482 pacientes com hipercolesterolemia familiar heterozigota. A média basal de LDL-C foi de 153 mg/dL em uso de uma estatina. No dia 510, a inclisirana reduziu o LDL-C em 47,9%, independente do genótipo subjacente. Os ensaios ORION-10 e ORION-11 tiveram desenho semelhante. Foram incluídos pacientes (n = 1.561 e n = 1.617, respectivamente) com DCV estabelecida e/ou com alto risco cardiovascular. A inclisirana reduziu o LDL-C em 52,3 e 49,9% no dia 510.^220^ A ocorrência de um desfecho cardiovascular pré-especificado foi numericamente menor naqueles em uso de inclisirana.

Nos estudos ORION-9, -10 e -11, colesterol total e não HDL, apoB, TG e Lp(a) foram reduzidos, e o nível de HDL-C aumentou. Em todos os três estudos, eventos adversos foram semelhantes em ambos os braços de tratamento; as exceções foram reações no local da injeção, geralmente leves e transitórias, que foram mais comuns naqueles que receberam inclisirana.

Para avaliar desfechos cardiovasculares, está em andamento o estudo ORION-4, que acompanha cerca de 15.000 pacientes com DCV estabelecida, tratados com estatina e com LDL-C acima de 79 mg/dL. A inclisirana (ou placebo) foi administrado na linha de base, após 3 meses e, então, a cada 6 meses por um total de 5 anos. O término está previsto para o ano de 2026.

O ácido bempedoico é um medicamento para reduzir o LDL-C, aprovado para uso na União Europeia e nos Estados Unidos desde 2020. Ele atua através da inibição da síntese hepática do colesterol, acima do local de ação das estatinas e é considerado uma pró-droga, pois necessita ser convertido em droga ativa pela enzima acil-CoA sintetase 1, presente no fígado e ausente nos tecidos musculares. Dessa maneira, sua grande virtude é não causar miopatia.

Em estudos para avaliar a eficácia e segurança, mostrou reduzir pouco o LDL-C quando associado a estatinas; no entanto, ao ser utilizado em monoterapia, em pacientes intolerantes às estatinas, reduziu pouco mais de 20% os níveis de LDL-C. Sua associação com ezetimiba já é comercializada fora do Brasil e proporciona uma redução do LDL-C similar à de uma estatina de moderada a alta potência. Recentemente publicado, o ensaio clínico CLEAR *Outcomes* (*Cholesterol Lowering via Bempedoic Acid, na ACL-Inhibiting Regimen*) avaliou o uso do ácido bempedoico em 13.970 pacientes com LDL-C acima de 100 mg/dL, 30% em prevenção primária de alto risco e 70% em prevenção secundária.^221^ Cerca de 22% dos pacientes faziam uso de doses muito baixas de estatinas, e a média do LDL-C nos dois grupos era 139 mg/dL. Deveriam ser intolerantes a duas ou mais estatinas ou intolerantes a uma estatina e que não estivessem dispostos a tentar uma segunda. Foi observada, 6 meses após o início do estudo, uma redução média do LDL-C de 21%; no decorrer de todo o estudo, a diferença média na redução do LDL-C entre o ácido bempedoico e o placebo foi de 22 mg/dL. Em relação ao desfecho primário (morte cardiovascular, IAM não fatal, AVC não fatal ou revascularização coronária) a taxa de eventos no grupo placebo foi de 13,3% e, no ácido bempedoico, foi de 11,7% (RRR de 13%; redução do risco absoluto [RRA] de 1,6%; IC95% 0,79–0,96). Em relação aos efeitos adversos, foi observada uma discreta elevação de enzimas hepáticas e maior incidência de gota e colelitíase no grupo que recebeu ácido bempedoico.

#### 4.2.2. Lipoproteína(a)

Estudos epidemiológicos têm demonstrado que concentrações plasmáticas de Lp(a) apresentam correlação linear com aumento do risco de aterosclerose e eventos cardiovasculares. Da mesma maneira, estudos genéticos têm, consistentemente, sugerido que a exposição ao longo da vida a níveis mais elevados de Lp(a) está forte e causalmente associada com risco aumentado de eventos cardiovasculares, bem como de estenose valvar aórtica calcificada.^222^

Medidas não farmacológicas, como dieta e atividade física, não reduzem os níveis de Lp(a). Estudos de randomização mendeliana têm sugerido que seria necessária uma redução entre 60 e 100 mg/dL (150 a 250 nmol/L) para obter redução entre 20 e 30% no risco de eventos cardiovasculares. Drogas que reduzem a Lp(a) entre 20 e 30% (incluindo niacina e inibidores da PCSK9) não têm oferecido evidências inequívocas de benefício clínico. Dessa maneira, atualmente, não existem medicamentos aprovados para reduzir a Lp(a).

Para isso, estão em andamento estudos de fase 3 em pacientes de prevenção secundária, utilizando drogas que atuam no RNA mensageiro para impedir a síntese da apolipoproteína(a). O pelacarsen é um oligonucleotídeo antissenso utilizado via subcutânea que reduz os níveis de Lp(a) em torno de 80%. No estudo Lp(a)HORIZON (*Assessing the mpacto f Lipoprotein(a) Lowering with TQJ230 on Major Cardiovascular Events in Patients With CVD*), iniciado em 2019, já foram randomizados mais de 8.300 pacientes com DCV e Lp(a) acima de 70 mg/dL, os quais serão acompanhados até o ano de 2025. O estudo OCEAN (*Olpasiran Trials of Cardiovascular Events and Lipoprotein(a) Reduction*) randomizou cerca de 7.297 pacientes com doença coronariana prévia e Lp(a) acima de 200 nmol/L para receber olpasiran (RNA interferente pequeno de fita dupla) ou placebo até o final de 2026; esse medicamento é capaz de reduzir em até 90% os níveis séricos de Lp(a). A partir da publicação desses estudos, será possível estabelecer definitivamente se há uma relação causal da Lp(a) com a DCV.^223^

#### 4.2.3. Lipoproteínas Ricas em Triglicerídeos

Uma nova visão em epidemiologia sugere que essas lipoproteínas, caracterizadas por grande conteúdo de TGs, são preditores fortes e independentes de eventos cardiovasculares ateroscleróticos e morte por todas as causas e que seu teor de colesterol (colesterol remanescente) é o principal responsável pela incidência de eventos cardiovasculares.^224^ Diferentemente do que foi observado com o HDL, estudos de randomização mendeliana sugerem que lipoproteínas ricas em TGs são causalmente associadas com eventos cardiovasculares ateroscleróticos. Adicionalmente, a evidência genética também demonstra que altas concentrações de lipoproteínas ricas em TGs estão causalmente associadas com inflamação, que é outro fator envolvido na gênese da aterosclerose.

É importante enfatizar que medidas não farmacológicas, como dieta, restrição de bebida alcoólica e atividade física, são fundamentais na redução dos níveis séricos de TGs. Quando for necessária intervenção farmacológica, a primeira linha de tratamento são as estatinas, com o objetivo de reduzir o colesterol não HDL ou a apoB.^225^ Após o atingimento dessas metas, para a redução do risco residual, podem ser consideradas outras alternativas terapêuticas associadas às estatinas.

Ao analisar estudos com fibratos, drogas que reduzem os níveis séricos de TGs, os resultados de análises secundárias ou *post hoc* vinham sugerindo que pacientes com a associação de altos níveis de TGs e baixos de HDL-C teriam benefício com o uso dessa terapia, mesmo que os resultados do desfecho primário tivessem sido neutros. No entanto, um estudo recentemente publicado, que utilizou o pemafibrato contra placebo, realizado em diabéticos com dislipidemia aterogênica e já tratados com estatinas, mostrou que a redução de TGs sem diminuição das partículas circulantes que contêm apoB não resultou em redução do risco cardiovascular.^266^ Dessa maneira, o uso de fibratos especificamente para a redução do risco cardiovascular não deve mais ser considerado.

Outra alternativa terapêutica para reduzir os TGs e eventos cardiovasculares seria o uso de ácidos graxos poli-insaturados de cadeia longa, que podem ser uma mistura de ácido eicosapentaenoico (EPA) e ácido docosa-hexaenoico (DHA) ou EPA isoladamente. O estudo REDUCE-IT (*Reduction of Cardiovascular Events with Icosapent Ethyl–Intervention Trial*) foi realizado em pacientes tratados com estatinas, com TGs elevados e DCV estabelecida ou diabéticos com fatores de risco adicionais. Avaliou-se que um dos componentes do ômega 3, o etil icosapente (EPA) ultrapurificado, poderia reduzir o risco de eventos cardiovasculares na comparação com o placebo. O benefício do tratamento com EPA foi muito significativo no desfecho combinado de morte cardiovascular, IAM ou AVC, com taxa de eventos de 20% no grupo placebo vs. 16,2% no tratamento ativo (RRR de 26%; RRA de 3,6%; número necessário [NNT]: 28).^225–227^

Por outro lado, o estudo STRENGTH (*Long-Term Outcomes Study to Assess Statin Residual Risk with Epanova in High Cardiovascular Risk Patients with Hypertriglyceridemia*) avaliou a combinação de EPA + DHA contra placebo em uma população similar à do estudo REDUCE-IT. O resultado do STRENGTH não mostrou benefício clínico para os pacientes tratados com ômega 3.^228^ Uma possível explicação para os resultados discrepantes é que, no REDUCE-IT, o placebo utilizado foi o óleo mineral, o qual foi associado com o aumento dos níveis de LDL-C e de marcadores inflamatórios; no STRENGTH, óleo de milho foi utilizado como placebo e se mostrou neutro. Para avaliar o real benefício do EPA ultrapurificado, o ideal seria repetir o estudo com EPA isolado, utilizando como placebo o óleo de milho (Tabela 16).

**Tabela 16:** Recomendações para Lipoproteínas Ricas em Triglicerídeos

Recomendações para obtenção de metas de LDL-C	Classe	Nível de evidência
Em pacientes com SCC recomenda-se uma redução do LDL-C de 50% a partir dos níveis basais, associado a uma meta abaixo de 50 mg/dL	**I**	**A**
Para pacientes com SCC que apresentam um segundo evento vascular dentro de 2 anos do primeiro e que estejam utilizando a terapia máxima tolerada em estatina, uma meta abaixo de 40 mg/dL pode ser considerada	**IIb**	**B**

Recomendações para a redução farmacológica do LDL-C em pacientes com SCC	Classe	Nível de evidência
É recomendado prescrever estatina de alta intensidade até a dose máxima tolerada para a obtenção da meta de LDL-C	**I**	**A**
Se a meta não for atingida com a dose máxima tolerada de estatina, a combinação com ezetimiba é recomendada	**I**	**B**
Em pacientes com SCC em uso de dose máxima tolerada de estatina, associada a ezetimiba, e que não atinjam a meta de LDL-C, a combinação com inibidor da PCSK9 é recomendada	**I**	**A**

Recomendação para a terapia farmacológica de pacientes com SCC e hipertrigliceridemia	Classe	Nível de evidência
Terapia com estatina é recomendada como primeira escolha para reduzir o risco cardiovascular em indivíduos com SCC e triglicerídeos acima de 200 mg/dL	**I**	**A**
Em pacientes com SCC e triglicerídeos acima de 135 mg/dL, em uso de estatinas e modificações do estilo de vida, o ácido graxo poli-insaturado ômega-3 (etil icosapente) a 4 gramas por dia pode ser considerado em combinação com estatina	**IIb**	**B**
Em pacientes com SCC em uso de estatina e que mantenham níveis de triglicerídeos acima de 200 mg/dL, fibratos não devem ser considerados para a redução do risco cardiovascular	**III**	**A**

LDL-C: colesterol de lipoproteínas de baixa densidade; SCC: síndrome coronária crônica.

### 4.3. Terapia de Reposição Hormonal

Os resultados de grandes estudos randomizados mostraram que a hormonioterapia de reposição não oferece benefício prognóstico e aumenta o risco de DCV em mulheres com idade > 60 anos.^229^

A terapia de reposição hormonal em mulheres na pós-menopausa não reduz o risco de doença isquêmica do miocárdio e, portanto, não é recomendada para prevenção primária e secundária (Tabela 17).^229–231^

**Tabela 17:** Recomendações para a Terapia de Reposição Hormonal

Recomendação	Grau de recomendação	Nível de evidência
A terapia de reposição hormonal não é recomendada para redução de risco em mulheres na pós-menopausa.	**III**	**C**

### 4.4. Bloqueio do Sistema Renina-Angiotensina-Aldosterona

Os inibidores da enzima conversora de angiotensina (iECA) mostraram benefícios em reduzir mortalidade, IM, AVC e IC entre os pacientes com fração de ejeção reduzida,^232–234^ doença vascular preexistente e de alto risco^235,236^ e diabetes.^237^ No entanto, em pacientes com doença coronariana estável e função ventricular esquerda preservada, nem todos os estudos demonstraram que os inibidores da ECA fornecem benefício adicional em termos de morte por todas as causas, morte cardiovascular, IM não fatal, AVC ou IC.^238^ Uma metanálise com 24 estudos demonstrou que, em pacientes com SCC e sem IC, o bloqueio do sistema renina-angiotensina reduziu eventos cardiovasculares e morte apenas quando comparado com placebo, e não quando comparado com controles ativos.^239^ Mesmo assim, o benefício dessa classe foi observado principalmente nos estudos com taxas de eventos mais altas.

Portanto, a terapia com iECA em pacientes com SCC sem IC ou sem alto risco cardiovascular geralmente não é recomendada. Orientamos o seu uso quando necessário para atingir as metas de pressão arterial.

Recomenda-se que os iECA ou bloqueadores do receptor de angiotensina, como alternativa para os pacientes que não toleram iECA, devam ser considerados para o tratamento de pacientes com SCC com hipertensão coexistente, FEVE reduzida < 40%, diabetes ou DRC, a menos que contraindicado (por exemplo, insuficiência renal grave, hipercalemia)

#### 4.4.1. Inibidores da Neprilisina e do Receptor da Angiotensina

Mais recentemente, o uso de inibidor farmacológico de neprilisina (endopeptidase neutra, presente na membrana de borda em escova, que degrada uma variedade de peptídeos bioativos) faz elevar os níveis de bradicinina e de peptídeos natriuréticos, aumentando, assim, a diurese e a natriurese, melhorando o relaxamento miocárdico e o remodelamento ventricular e reduzindo a secreção de renina e aldosterona. O primeiro da classe é o LCZ696, que combina valsartana e sacubitril (inibidor da neprilisina). Em pacientes com IC (FEVE ≤ 35%) que permaneciam sintomáticos apesar do tratamento otimizado, o uso de sacubitril/valsartana foi superior ao enalapril na redução dos riscos de morte e de hospitalização por IC.^240^ Nesse estudo, é importante enfatizar que 60% dos pacientes eram portadores de cardiopatia isquêmica. Assim, é recomendado como um substituto para um iECA para reduzir ainda mais o risco de hospitalização e morte por IC.

#### 4.4.2. Bloqueadores da Aldosterona

O bloqueio da aldosterona com espironolactona ou eplerenona é recomendado para uso em pacientes pós-IAM com medicação otimizada, fração de ejeção reduzida (FEVE ≤ 35%) e portadores de diabetes ou IC.^241,242^

Esses medicamentos devem ser usados com cautela em pacientes com função renal prejudicada e naqueles com hipercalemia (Tabela 18).

**Tabela 18:** Recomendações para uso de inibidores do sistema renina-angiotensina-aldosterona. Prioridade para inibidor da enzima conversora de angiotensina-1 (iECA); e aos intolerantes aos iECA, o uso dos antagonistas do receptor I da angiotensina

Recomendação	Grau de recomendação	Nível de evidência
De rotina, quando hà disfunção ventricular e/ou IC e/ou diabetes mellitus.	**I**	**A**
De rotina em todos os pacientes com DAC.	**IIa**	**B**
O uso de bloqueadores de aldosterona deve ser usado em pacientes pós-IAM com medicação otimizada, fração de ejeção reduzida (FEVE ≤ 35%) e portadores diabetes ou IC.	**IIa**	**A**

DAC: doença arterial coronariana; FEVE: fração de ejeção do ventrículo esquerdo; IAM: infarto agudo do miocárdio; IC: insuficiência cardíaca.

### 4.5. Colchicina

Causalidade tem sido amplamente estabelecida entre inflamação e doença aterosclerótica, indicando a contenção da atividade inflamatória sistêmica como alvo terapêutico. Nesse sentido, ensaios clínicos testaram terapias anti-inflamatórias abrangentes ou específicas na prevenção de eventos ateroscleróticos cardiovasculares. Nessas terapias, destacaram-se como alvo terapêutico a inibição da ativação ou o bloqueio da ação por anticorpos monoclonais da interleucina (IL) 1b, bloqueando a via canônica do inflamassoma NLRP3, ou da IL-6, agindo diretamente sobre a resposta inata.

Nos ensaios para testar as terapias genéricas, foram utilizados o metotrexato e a colchicina. No ensaio CIRT (*Cardiovascular Inflammation Reduction Trial*), foi delineado metotrexato em baixa dose (15 a 20 mg/semana) para comparar ao placebo, mas foi interrompido precocemente por futilidade. Além disso, no braço metotrexato, ocorreram elevações das enzimas hepáticas e reduções nas contagens de leucócitos e no hematócrito, além de uma maior incidência de câncer de pele quando comparado ao placebo.^243^

Utilizada por muitas décadas, a colchicina exerce sua ação anti-inflamatória ligando-se à tubulina e inibindo sua polimerização. Essa propriedade interrompe a função do citoesqueleto, reduzindo a quimiotaxia, migração e sinalização celular, e inibe a mitose celular. Outros mecanismos envolvidos são a diminuição da expressão das moléculas de adesão, a regulação negativa da via NF-κB e a supressão da formação de inflamassoma NLRP3.^244^ O estudo LODOCO-2 randomizou 5.522 pacientes com DAC crônica a colchicina 0,5 mg/dia ou placebo. O tratamento com colchicina reduziu o desfecho primário composto de morte cardiovascular, IM não procedural, revascularização coronariana orientada por isquemia, ou AVC isquêmico em 1,1%, quando comparado ao placebo (HR 0,69; IC 95% 0,57–0,83; p < 0,001). No entanto, a incidência de morte por causas não cardiovasculares foi 0,2% maior no grupo colchicina (HR 1,51; IC 95% 0,99–2,31).^245^

Recentes metanálises englobando estudos com pacientes em prevenção secundária avaliaram desfechos de eficácia e segurança para prevenção de ocorrências de eventos ateroscleróticos e de acidente vascular encefálico isquêmico, mostrando benefício em baixa dose de colchicina (0,5 mg/dia) na redução de tais eventos. Há de se ponderar que houve heterogeneidade entre os estudos, bem como nos critérios de inclusão dos pacientes em cada um deles.^246,247^

A fim de avaliar a capacidade da administração de colchicina como terapia anti-inflamatória em pacientes na fase aguda pós-IM, o estudo multicêntrico CLEAR SYNERGY incluiu 7.064 pacientes, randomizou-os (1:1) para receber colchicina vs. placebo e avaliou desfecho primário de eficácia composto por moxrte por causas cardiovasculares, infarto miocárdico recorrente, acidente vascular encefálico isquêmico ou revascularização miocárdica não planejada e guiada por isquemia numa análise de tempo até o evento. Em um seguimento médio de 3 anos, não houve benefício significativo no uso de colchicina nessa população específica de pacientes.^248,249^

A colchicina possui índice terapêutico estreito, o que significa que há uma pequena diferença entre a dose eficaz e a dose que pode causar efeitos adversos graves ou tóxicos. Além disso, a colchicina é metabolizada pelo citocromo P450 3A4 e pela p-glicoproteína, o que a torna suscetível a interações medicamentosas. Portanto, o monitoramento dos efeitos adversos é de extrema importância. Dado isso, é necessária uma abordagem altamente individualizada, limitando o uso da colchicina a pacientes que permanecem em alto risco apesar da terapia médica otimizada, até que mais dados estejam disponíveis.

Entre as terapias anti-inflamatórias específicas, dois alvos terapêuticos têm se destacado. O primeiro deles, o bloqueio da IL-1b, foi testado no estudo CANTOS (*Canakinumab Anti-inflammatory Thrombosis Outcome Study*), um ensaio clínico randomizado, duplo-cego e controlado por placebo, no qual se investigou o efeito do canaquinumabe na redução da recorrência de eventos cardiovasculares. Um total de 10.061 pacientes com histórico de IM e hs-CRP ≥ 2 mg/L foram designados para receber canaquinumabe ou placebo. O desfecho primário foi o composto de morte cardiovascular, IM não fatal ou AVC isquêmico não fatal reduzido com o canaquinumabe (HR 0,85; IC 95% 0,74–0,98; p = 0,021).^250^ A despeito da evidência e confirmação da teoria inflamatória da aterogênese, o canaquinumabe não se tornou terapia preventiva para aterosclerose devido à limitação relacionada ao alto custo.

O bloqueio por anticorpo monoclonal humano direcionado à IL-6, o ZiltiveKimab, foi testado no ensaio randomizado controlado, estudo RESCUE (*Randomized Evaluation of Patients with Stable Angina Comparing Utilization of Noninvasive Examinations*), que incluiu pacientes com DRC moderada a grave e hs-CRP acima de 2 mg/L.^251^ Houve reduções dependentes da dose em níveis de hs-CRP, fibrinogênio, haptoglobina, fosfolipase A2 e lipoproteína (a), indicando sua ação anti-inflamatória. Como consequência desses achados, um ensaio controlado randomizado fase 3, o estudo ZEUS (*Zotarolimus-eluting Endeavor Sprint Stent in Uncertain DES Candidates*), está em andamento com cerca de 5.000 pacientes com DAC crônica, DRC e hs-CRP elevado (Tabela 19).

**Tabela 19:** Sumário da indicação de terapia anti-inflamatória em pacientes com DAC crônica

Recomendação	Grau de recomendação	Nível de evidência
A adição de colchicina pode ser considerada para reduzir os eventos coronarianos agudos recorrentes	**IIb**	**B**

### 4.6. Terapia Antidiabética

Apesar de esforços persistentes, as taxas de eventos cardiovasculares permanecem altas, mesmo entre aqueles bem controlados, e a DAC continua sendo a principal causa de morbidade e morte.

Na maioria dos pacientes, o cenário é agravado pela não adoção das medidas terapêuticas bem estabelecidas para o diabetes melito tipo (DM2). Nesse espectro de ações, devem-se incluir modificações no estilo de vida e terapia medicamentosa para otimizar o controle de dislipidemia, hipertensão, manejo do peso e hiperglicemia. Em adição a essas medidas, na última década, consolidaram-se três classes de fármacos antidiabéticos, a saber, a pioglitazona, os inibidores de SGLT2 e agonistas do receptor GLP-1, comprovando sua ação de redução de eventos cardiovasculares, independentemente de seus efeitos sobre a glicemia. Esses medicamentos diminuem o risco de eventos cardiovasculares por diferentes mecanismos.

A pioglitazona foi testada como medida de prevenção de eventos ateroscleróticos em dois ensaios clínicos. No primeiro, o PROactive (*Prospective Pioglitazone Clinical Trial in Macrovascular Events*), 5.238 pacientes com DM2 e DAC foram tratados com pioglitazona (15 a 45 mg) ou placebo em adição ao tratamento recomendado para DM2. O tratamento não atingiu significância no desfecho primário composto de mortalidade por todas as causas, IM não fatal (incluindo IM silencioso), AVC, SCA, intervenção endovascular ou cirúrgica nas artérias coronárias ou na perna e amputação acima do tornozelo (HR 0,90; IC 95% 0,80–1,02; p = 0,095). O desfecho secundário, composto de morte cardiovascular, IM e AVC não fatais, demonstrou uma redução de 16% do risco relativo com a pioglitazona (HR 0,84; IC95% 0,72–0,98; p = 0,027).^252^ No ensaio IRIS (*Insulin Resistance Intervention after Stroke*), 3.876 pacientes que tiveram AVC isquêmico recente ou ataque isquêmico transitório foram aleatorizados para receber pioglitazona (45 mg/dia) ou placebo. O desfecho primário composto de AVC fatal ou não fatal ou IM fatal ou não fatal reduziu em 2,8% após 4,8 anos (HR 0,76; IC 95% 0,62–0,93; p = 0,007).^253^ O conjunto desses achados indica redução de eventos ateroscleróticos com a pioglitazona. No estudo PROactive, no entanto, houve aumento de 2% nas hospitalizações por IC no grupo tratado com pioglitazona. Em decorrência disso, não se deve indicar o tratamento com pioglitazona em pacientes com IC.

Os achados dos ensaios clínicos que testaram os inibidores de SGLT2 parecem refletir um efeito de menor magnitude na redução dos eventos ateroscleróticos do que na incidência ou agravamento da IC. No entanto, sendo o efeito sobre a IC muito mais precoce (cerca de 30 dias), o viés causado pela competição de riscos torna incalculável o real efeito mais tardio na prevenção de eventos ateroscleróticos. Metanálises tradicionais e de rede com efeito randômico, por meio de amplificação do poder estatístico, confirmam a existência da proteção contra eventos ateroscleróticos, particularmente em pacientes em prevenção.^254,255^ No entanto, ainda assim, esse efeito pode estar subestimado pela interrupção precoce do tratamento em decorrência do benefício em mais curto prazo nas manifestações de IC.

Os ensaios com agonistas do receptor GLP-1 demonstraram claramente a redução do risco de eventos ateroscleróticos, particularmente em prevenção secundária.^256,257^ Recentemente, agonista de GLP-1 foi comparado ao placebo em pacientes com doença aterosclerótica e obesidade, mas sem DM2. O tratamento com agonista de GLP-1 reduziu em 1,5% a incidência do desfecho composto de morte por causas cardiovasculares, IM não fatal ou AVC não fatal (HR 0,80; IC 95% 0,72–0,90).^258^ Portanto, tanto em indivíduos em prevenção secundária com DM2 quanto naqueles com obesidade, o tratamento com agonistas de GLP-1 se mostrou eficaz na redução de eventos ateroscleróticos cardiovasculares.

Dados os seus mecanismos de ação distintos, a redução do risco cardiovascular pode ser maior utilizando combinações das classes de medicamentos antidiabéticos em comparação com qualquer um dos medicamentos isoladamente. Os dados sobre o uso concomitante são principalmente limitados a desfechos de segurança e metabólicos, mas, nos em metanálise de rede, foi relatado efeito aditivo com a combinação de pioglitazona com agonistas dos receptores de GLP-1 e de inibidores de SGLT2i com agonistas dos receptores de GLP-1.^259^ Diante do amplo risco residual observado nos ensaios clínicos em que se testou cada um destes fármacos, é razoável que se busquem as associações terapêuticas desde o início da terapia naqueles pacientes em prevenção secundária e DM2 (Tabela 20).

**Tabela 20:** Sumário da indicação de terapia antidiabética em pacientes com DAC crônica

Recomendação	Grau de recomendação	Nível de evidência
Em pacientes com DM2, inibidores de SGLT2 devem ser utilizados para a prevenção secundária de eventos ateroscleróticos recorrentes	**I**	**A**
Em pacientes com DM2, agonistas dos receptores de GLP-1 devem ser utilizados para a prevenção secundária de eventos ateroscleróticos recorrentes	**I**	**A**
Em pacientes com DM2, a combinação de inibidores de SGLT2 e agonistas dos receptores de GLP-1 deve ser considerada com a finalidade de reduzir o risco residual	**I**	**C**
Em pacientes com DM2, sem IC, em terapia combinada com inibidores de SGLT2 e agonistas dos receptores de GLP-1, a adição de pioglitazona pode ser considerada como terapia de adição para prevenção secundária de eventos ateroscleróticos recorrentes	**IIa**	**B**

DM2: diabetes mellitus tipo 2; IC: insuficiência cardíaca.

## 5. Tratamento Clínico-Farmacológico para Controle Ótimo de Sintomas

O tratamento farmacológico na SCC tem como objetivo reduzir os sintomas de angina e a isquemia induzida pelo exercício, o desenvolvimento da disfunção ventricular, além de prevenir eventos cardiovasculares. O tratamento clínico deve ser priorizado na SCC, com a mudança de estilo de vida e com tratamento medicamentoso ótimo, que pode ser definido como aquele que controla satisfatoriamente os sintomas e previne eventos cardíacos associados à SCC, com máxima adesão do paciente e mínimos efeitos adversos. Esse tratamento, então, inclui drogas que proporcionam estabilidade da placa e redução de eventos, bem como drogas que promovam a redução da incidência de angina. Deve-se ressaltar, no entanto, que não há uma definição universal sobre quais seriam as drogas antianginosas "ideais" para o tratamento ótimo de pacientes com SCC, e as terapias medicamentosas devem ser adaptadas às características e preferências de cada paciente e à experiência do médico.^1^

A terapia antianginosa inicial geralmente consiste em um ou dois fármacos, conforme necessário. A escolha inicial do tratamento depende da tolerância esperada, interações medicamentosas, preferências do paciente e disponibilidade do medicamento. Os custos do tratamento invariavelmente também devem ser considerados, principalmente no contexto de atendimento de saúde pública. Dessa forma, ressalta-se que o tratamento antianginoso deve ser sempre individualizado a cada paciente. As ações, contraindicações e formas de uso de cada uma das drogas antianginosas são descritas a seguir.

### 5.1. Betabloqueadores

Os betabloqueadores ligam-se aos receptores adrenérgicos através da proteína G, inibindo, assim, os efeitos da adrenalina e da noradrenalina nesses receptores.^260^ Em pacientes com *angina pectoris*, o β-bloqueio reduz a isquemia e melhora a qualidade de vida ao diminuir a frequência cardíaca (FC) e a contratilidade miocárdica, especialmente durante o exercício, além de prevenir o aumento da pressão arterial induzido pelo esforço. Além disso, aumenta a perfusão em áreas isquêmicas por aumento no tempo de diástole e da resistência vascular em áreas não isquêmicas. A dose do betabloqueador deve ser ajustada para se ter como alvo uma FC de 55 a 60 bpm em repouso.^261^ Em caso de descontinuação, ela deve ser feita gradualmente, e não de forma abrupta.

Existem vários subtipos de betabloqueadores: não seletivos, sem atividade simpatomimética intrínseca (propranolol e sotalol); não seletivos, com atividade simpatomimética intrínseca (pindolol); β1-seletivo (atenolol, bisoprolol e metoprolol); β1-seletivo com atividade α-bloqueadora (carvedilol); e β1-seletivo com propriedades vasodilatadoras mediadas pelo óxido nítrico (nebivolol).^262^

Ainda hoje, os betabloqueadores são a classe inicial de tratamento mais utilizada, seja pela experiência de uso, pelo custo e por seus benefícios quanto à redução de mortalidade em pacientes com IC. Em pacientes com IC com fração de ejeção reduzida (ICfer), os betabloqueadores foram associados a uma redução significativa na mortalidade e/ou eventos cardiovasculares.^263–265^

Também em pacientes submetidos a cirurgia de revascularização miocárdica, os betabloqueadores foram associados a menor risco de eventos cardiovasculares e mortalidade geral.^266^

Após IM, estudos mais antigos sugeriram que a utilização rotineira de betabloqueadores poderia estar relacionada com menor incidência de eventos cardiovasculares.^267^ Entretanto, a maioria dos ensaios que demonstraram benefício do tratamento com betabloqueadores após IM incluiu pacientes com grandes IMs e foi conduzida em uma era anterior ao diagnóstico moderno de IM baseado em biomarcadores e ao tratamento com intervenção coronária percutânea (ICP), agentes antitrombóticos, estatinas de alta intensidade e antagonistas do sistema renina-angiotensina-aldosterona. Em contrapartida, em 2024, foi publicado o estudo REDUCE-AMI (*Randomized Evaluation of Decreased Usage of Beta-Blockers after Acute Myocardial Infarction*), que avaliou pacientes com IAM submetidos à angiografia coronária precoce e que apresentavam FEVE preservada (≥ 50%). Nesse estudo, o tratamento prolongado com betabloqueadores não levou a um menor risco do desfecho primário composto de morte por qualquer causa ou novo IM em relação aos pacientes que não utilizaram betabloqueadores.^268^

Além disso, em pacientes com SCC sem ICfer ou sem infarto no último ano, os estudos, de uma forma geral, não demonstraram benefício do uso de betabloqueadores na redução de mortalidade e/ou eventos cardiovasculares.^269^

Estudos clínicos randomizados que avaliaram os efeitos dos betabloqueadores no tratamento das SCC em vigência de sintomas ou de isquemia demonstraram redução muito significativa do número de crises de angina, da isquemia e também aumento da tolerância ao esforço físico.^270–272^ Em comparação com outras medicações, os efeitos antianginosos dos betabloqueadores são similares ao de outras medicações anti-isquêmicas.^273–275^ Mas é importante enfatizar que a combinação de diferentes antianginosos, incluindo betabloqueadores, é capaz de trazer benefícios adicionais e deve ser uma estratégia encorajada.^260^

Recomenda-se cautela quando um betabloqueador é combinado com verapamil ou diltiazem pelo risco de piora da IC, bradicardia excessiva e/ou bloqueio atrioventricular, mas os betabloqueadores podem ser combinados com ivabradina.^276,277^ Os principais efeitos colaterais dos betabloqueadores são fadiga, depressão, bradicardia, bloqueio cardíaco, broncoespasmo, vasoconstrição periférica, hipotensão postural, impotência e mascaramento dos sintomas de hipoglicemia.^260,262^ Eles não devem ser prescritos a pacientes com angina vasoespástica porque podem precipitar vasoespasmo mediado por aumento da atividade α-adrenérgica.

### 5.2. Trimetazidina

A trimetazidina é um agente antianginoso clinicamente eficaz e que não apresenta qualquer efeito na hemodinâmica cardiovascular, sem qualquer influência na pressão arterial ou na FC. Isso é particularmente útil em pacientes com duplo produto controlado e que permanecem sintomáticos.

A trimetazidina atua inibindo a enzima 3-cetoacil-CoA tiolase, que normalmente catalisa a última etapa da betaoxidação dos ácidos graxos no miocárdio. Ao inibir essa enzima, a trimetazidina reduz a utilização de ácidos graxos como fonte de energia pelo coração e aumenta a utilização de glicose. Isso melhora a eficiência energética do miocárdio, reduzindo a demanda por oxigênio e aumentando a tolerância à isquemia.

Os efeitos antianginosos clínicos da trimetazidina foram testados em estudos em pacientes com isquemia crônica, tanto como monoterapia quanto como em associação com outras medicações antianginosas, como bloqueadores dos canais de cálcio (BCCs) e betabloqueadores.^275^ Quando utilizada isoladamente, seus efeitos benéficos foram semelhantes aos da monoterapia com betabloqueadores ou antagonistas dos canais de cálcio no tratamento das SCC.

Em uma metanálise, a trimetazidina melhorou significativamente a tolerância ao exercício, reduziu episódios semanais de angina e reduziu o uso de nitratos de ação curta em comparação com o placebo.^280^ Além disso, estudos de mundo real demonstraram que a utilização de trimetazidina, associada a medicações antianginosas com efeito hemodinâmico, reduz o número de episódios semanais de angina e também reduz o consumo de nitrato sublingual.^281,282^

Existem duas preparações diferentes para uso comercial: trimetazidina 35 mg (que deve ser administrada em duas tomadas diárias) e trimetazidina 80 mg (que deve ser administrada uma vez por dia).

Geralmente, os efeitos adversos relacionados ao tratamento são leves e bem tolerados, compreendendo principalmente distúrbios gastrointestinais, como náuseas e vômitos, além de cefaleia. No estudo ATPCI (*Efficacy and Safety of Trimetazidine After Percutaneous Coronary Intervention*), a trimetazidina não foi associada a nenhum evento adverso comparativamente ao placebo. A incidência de eventos adversos julgados foi baixa e bem equilibrada entre os grupos de tratamento. Não houve aumento na ocorrência de sintomas neurológicos, como doença de Parkinson, parkinsonismo atípico ou parkinsonismo induzido por drogas, comparando os braços placebo e trimetazidina.^283^ Nesse estudo, a utilização de trimetazidina comparativamente ao placebo não reduziu eventos cardiovasculares nos pacientes com tratamento ótimo e após angioplastia bem-sucedida.

A trimetazidina não deve ser prescrita quando a taxa de filtração glomerular for < 30 mL/min/1,73 m^2^. Em pacientes com taxa de filtração glomerular < 60 mL/min/1,73 m^2^, a dose de trimetazidina deve ser corrigida para 35 mg, administrada uma vez por dia. E, recentemente, um estudo brasileiro confirmou a redução de angina e melhora da qualidade de vida em estudo observacional (VGOOD) com trimetazidina 80 mg LP uma vez ao dia.^284^

### 5.3. Antagonistas dos Canais de Cálcio

É um grupo de drogas heterogêneo com os seguintes efeitos farmacológicos: relaxamento da musculatura lisa, redução da pós-carga, efeitos inotrópicos negativos (verapamil e diltiazem) e redução do consumo de oxigênio. Os derivados di-hidropiridínicos (nifedipino, anlodipino e outros), os benzodiazepínicos (diltiazem) e as fenilalquilaminas (verapamil) constituem os três principais subgrupos de ACC que bloqueiam especificamente os canais de cálcio tipo L.

O verapamil reduz a condução atrioventricular, tem efeito inotrópico negativo e relaxa a musculatura lisa vascular, aumentando o fluxo coronariano e reduzindo a pós-carga. Os di-hidropiridínicos relaxam a musculatura lisa vascular, não modificam a velocidade da condução atrioventricular e, por mecanismos reflexos, aumentam a FC. O diltiazem tem efeitos similares aos do verapamil, exceto a depressão miocárdica, que é menos intensa nesse subgrupo.

Os antagonistas dos canais de cálcio (ACC) melhoram os sintomas e a isquemia miocárdica, mas não demonstraram reduzir os principais desfechos de morbimortalidade em pacientes com SCC.^260,285–287^ Esse grupo é indicado em todas as formas de angina, incluindo a vasoespástica, com algumas características individuais.

### 5.4. Verapamil

Possui boa segurança, mas com riscos de bloqueio cardíaco, bradicardia e IC. A combinação com betabloqueador não é recomendada (devido ao risco de bloqueio cardíaco).

### 5.5. Diltiazem

O diltiazem é bem tolerado e apresenta vantagens em relação ao verapamil no tratamento da angina de esforço e vasoespástica.^288^ Ele tem discreto efeito inotrópico negativo e inibição do nódulo sinusal.

A associação dos ACCs não di-hidropiridínicos com os betabloqueadores pode ser feita em pacientes selecionados, desde que se mantenha vigilância da FC. O uso dessas drogas na presença de disfunção ventricular não é recomendado.

### 5.6. Di-hidropiridínicos

Os mais utilizados são a nifedipino de ação prolongada e o anlodipino, que também possuem uma boa tolerabilidade, tendo como principal efeito colateral o edema de membros inferiores que, em alguns casos, limita o seu uso. A associação com betabloqueadores, quando necessária, está indicada e possui maior eficácia.^289^

### 5.7. Ivabradina

A ivabradina reduz a FC, aumentando o tempo de diástole e o tempo de perfusão, sem afetar a pressão arterial, o tônus vascular e a função sistólica do VE, através da inibição da corrente I_f_ no nó sinusal.^1,290–293^ A combinação de ivabradina com betabloqueadores não apenas reduz a FC, os episódios de angina e o consumo de nitratos, como também aumenta a tolerância ao exercício, aumentando o tempo total de esforço em um TE e melhorando a qualidade de vida em pacientes com angina.^276,294^ A ivabradina também aumenta a reserva de fluxo coronariano e parece afetar positivamente a função colateral e a perfusão miocárdica em pacientes com angina em SCCs.^295–298^

A ivabradina não reduziu desfechos clínicos em pacientes com angina e função ventricular deprimida ou preservada nos estudos BEAUTIFUL (*Morbidity-Mortality Evaluation of the I(f) Inhibitor Ivabradine in Patients with Coronary Disease and Left Ventricular Dysfunction*) e SIGNIFY (*Study Assessing the Morbidity-Mortality Benefits of the If Inhibitor Ivabradine in Patients with Coronary Artery Disease*), respectivamente.^299,300^ Entretanto, reduziu a mortalidade e hospitalização por IC em pacientes com IC e função ventricular reduzida no estudo SHIFT (*Systolic Heart Failure Treatment with the I(f) Inhibitor Ivabradine Trial*).^301^ O aumento do risco de mortalidade cardiovascular e de infarto não fatal em um subgrupo de pacientes com angina limitante do estudo SIGNIFY levantou preocupações com relação à segurança da ivabradina. Esse resultado pode ser explicado pelo uso de uma dose mais alta de ivabradina (10 mg duas vezes ao dia), de uma FC -alvo mais baixa (60 spm) e do uso concomitante de BCCs não di-hidropiridínicos, que impactam a redução do metabolismo da ivabradina pela inibição do citocromo P450 CYP3A4.^300^ Por outro lado, no estudo BEAUTIFUL, houve redução do risco de eventos cardiovasculares maiores no subgrupo de pacientes com angina limitante em uso de ivabradina.^299^

Em uma análise agrupada de estudos observacionais, a ivabradina foi segura e efetiva quando combinada aos betabloqueadores.^302^ Além da segurança, proporciona benefícios adicionais quando associada a outros antianginosos, embora a combinação com verapamil ou diltiazem deva ser evitada.^1,303^

Os principais efeitos colaterais são distúrbios visuais (fosfenos), cefaleia, tonturas, bradicardia, FA e bloqueio atrioventricular. É contraindicada com FC < 70 bpm, no IAM e na doença hepática severa. As interações medicamentosas podem ocorrer com drogas que aumentam o intervalo QTc e fortes inibidoras do citocromo P450 CYP3A4.^1^

A dose inicial não deve exceder 5 mg duas vezes ao dia, e a dose de manutenção não deve exceder 7,5 mg duas vezes ao dia. A descontinuação deve ser considerada se não houver melhora dos sintomas ou redução clinicamente relevante da FC dentro de 3 meses no início do tratamento. Se, durante o tratamento, a FC for reduzida para < 50 bpm ou o paciente apresentar sintomas relacionados a bradicardia, a dose deverá ser reduzida para 2,5 mg duas vezes ao dia. Se os sintomas persistirem ou a FC permanecer < 50 bpm apesar da redução da dose, o tratamento deverá ser descontinuado.^303^

### 5.8. Ranolazina

A ranolazina é um derivado ativo da piperazina, que promove seus efeitos através da redução de sobrecarga de cálcio no miócito isquêmico por meio da inibição da corrente de sódio tardia (*I_Na_*).^304^ Modelos animais também sugerem um possível efeito através do aumento de adenosina.^305^

Diferentes ensaios clínicos falharam em demonstrar uma associação entre o uso da ranolazina e uma redução de desfechos cardiovasculares como infarto, morte cardiovascular e necessidade de revascularização.^306–308^ Se, por um lado, não existem evidências que comprovem o benefício da ranolazina na redução de desfechos cardiovasculares, ensaios clínicos comprovaram que sua adição a outros antianginosos demonstrou capacidade de promover redução significativa da frequência de episódios de angina e da necessidade no uso de nitrato sublingual,^309,310^ redução na intensificação da classe funcional da angina,^307^ redução na recorrência de angina e aumento da distância percorrida no TE.^308,311^ Esses dados são corroborados por uma revisão sistemática que comprova a redução da angina com o uso da ranolazina.^274^ Contudo, o uso da ranolazina como monoterapia não parece demonstrar qualquer benefício clínico significativo em pacientes com SCC.^274^

Dados de pequenos estudos sugerem que os efeitos da ranolazina parecem particularmente úteis na redução da angina em paciente sem lesões coronarianas significativas em que a dor torácica está associada à doença microvascular.^312,313^ Outro quase-experimento sugere, ainda, efeito antianginoso em pacientes com cardiomiopatia hipertrófica,^314^ bem como um relato de caso propõe potencial efeito na redução do vasoespasmo.^315^

A ranolazina possui, ainda, um efeito potencialmente benéfico em pacientes com diabetes: em um ensaio clínico que avaliou pacientes com diabetes, o uso da ranolazina por 24 semanas como monoterapia resultou em uma taxa de hemoglobina glicada inferior a 7,0% em 41,2% dos pacientes, em comparação com 25,6% daqueles que receberam placebo. Esse efeito parece ocorrer devido à inibição da secreção de glucagon pelas células α pancreáticas, por meio do bloqueio dos canais de sódio e da inibição da atividade elétrica.^316^ Por sua vez, um ensaio clínico que avaliou pacientes com diabetes e SCC demonstrou que o uso de ranolazina por 8 semanas promoveu redução significativa da frequência de episódios de angina e da necessidade no uso de nitrato sublingual, sem aumento de efeitos colaterais. Uma possível justificativa poderia ser por uma ação mais eficaz sobre os canais lentos de sódio em pacientes com controle glicêmico inadequado.^309^

Outro aspecto que merece atenção é o potencial efeito antiarrítmico da ranolazina. Embora não se tenha comprovada a eficácia da ranolazina em pacientes com SCA sem elevação do segmento ST, seu uso nessa situação reduziu significativamente os episódios de taquicardia ventricular não sustentada com duração de pelo menos 8 batimentos.^317^ Um pequeno estudo não controlado evidenciou ainda que, em pacientes com FA, a administração de alta dose de ranolazina oral mostrou ser uma opção segura e potencialmente eficaz na conversão da FA aguda ou paroxística para o ritmo sinusal, com uma taxa de conversão de 72% e sem eventos adversos significativos.^318^ Esses dados sugerem um potencial efeito antiarrítmico da ranolazina, o que poderia sugerir sua utilização, por exemplo, em pacientes com angina e FA paroxística.

Há um aumento de 22% na incidência de eventos adversos com a adição da droga.^332^ A maioria dos efeitos colaterais, contudo, não é grave, sendo que os mais comuns são constipação, cefaleia, tontura, dispepsia, dor abdominal, náusea e astenia.^304,311^ Há, ainda, registros de aumento na incidência de síncopes de etiologia provavelmente vasovagal, bem como discreto aumento no intervalo QT.^307,311^ Dessa forma, sugere-se a avaliação do QTc antes e durante a terapia com a ranolazina.

A ranolazina é metabolizada principalmente pelas enzimas CYP3A4 e CYP2D6, com apenas uma pequena quantidade sendo excretada inalterada na urina. Ela é contraindicada em pacientes com insuficiência hepática, insuficiência renal, prolongamento do intervalo QTc ou em uso de medicamentos que prolongam esse intervalo. Diferentes interações limitam a sua utilização: possui efeito inibitório fraco no CYP3A, o que leva a um aumento na concentração plasmática de sinvastatina e aumenta a concentração de digoxina devido à inibição da glicoproteína-P. Deve-se ressaltar que pode ser considerada a coadministração de rosuvastatina, atorvastatina, pitavastatina, fluvastatina ou pravastatina com ranolazina se for clinicamente indicado; porém, no caso da sinvastatina, a dose deve ser limitada a 20 mg por dia quando prescrita junto a ranolazina. Além de aumentar a exposição plasmática a outros fármacos, a concentração de ranolazina é aumentada por medicamentos que inibem o CYP3A, como o cetoconazol, diltiazem e verapamil.^304,319,320^

Por não promover redução da FC ou da pressão arterial, a ranolazina é uma opção em pacientes hipotensos ou com a FC reduzida pelo betabloqueador que persistem com angina. A dose sugerida para uso é de 500 mg duas vezes ao dia, que deve ser aumentada para 1.000 mg duas vezes ao dia se bem tolerada.^274,304^

### 5.9. Nitratos de Ação Curta e Prolongada

Os nitratos aliviam a angina pelo efeito vasodilatador coronário e periférico, mediado pela ação do óxido nítrico, com redistribuição do fluxo coronário e reduções na resistência vascular sistêmica e pré-carga.^321^ Os nitratos de ação rápida disponíveis no Brasil são o dinitrato de isossorbida e o propatilnitrato, ambas formulações para uso sublingual. Esses medicamentos são a terapia-padrão para o alívio imediato da angina de esforço e vasoespástica, com início de ação em poucos minutos e duração inferior a 1 hora. A dose pode ser repetida com intervalos de 5 minutos até a melhora da angina ou até o alcance da dose máxima de 15 mg em 15 minutos, no caso do dinitrato de isossorbida. Ao receber a medicação, os pacientes devem repousar sentados, pois, em ortostase, há risco de síncope, e em decúbito, há aumento do retorno venoso e da pré-carga. Os nitratos de ação curta também são recomendados para a profilaxia de situações nas quais o paciente espera ter angina, como durante atividade física, estresse emocional, relação sexual, após refeições ou clima mais frio.^322^

Os nitratos de ação prolongada demonstraram melhorar a tolerância ao exercício, o tempo até a depressão do segmento ST e o tempo até o início da angina em pacientes com SCC, embora em estudos com pequeno número de pacientes e seguimento relativamente curto.^322–324^

Há, no entanto, limitações e preocupações com relação ao uso dos nitratos de ação prolongada. Quando utilizados por um período contínuo e prolongado, ocorre tolerância e perda de eficácia, o que requer a prescrição de um intervalo livre de nitratos de 10 a 14 horas.^324^ Adicionalmente, a interrupção deve ser gradual, e não abrupta, para evitar angina de rebote. A terapia de longo prazo com nitratos está associada à disfunção endotelial, por meio do acúmulo de radicais livres de oxigênio, que aumentam a sensibilidade arterial ao sistema simpático e aos vasoconstritores.^325^

Quanto ao impacto em desfechos cardiovasculares, a evidência mais robusta demonstrou uma maior taxa de eventos (morte, infarto não fatal e IC) com uso de nitratos de ação prolongada.^326^ Adicionalmente, estudos em pacientes com angina vasoespástica também demonstraram maior ocorrência de eventos cardiovasculares com o uso de nitrato de ação prolongada.^327,328^ Os efeitos colaterais mais comuns são hipotensão, dor de cabeça e rubor. As contraindicações incluem cardiomiopatia hipertrófica obstrutiva, estenose valvar aórtica grave e coadministração de inibidores da fosfodiesterase.^329,330^

### 5.10. Alopurinol

Altas doses de alopurinol (600 mg por dia), um inibidor da xantina oxidase, foram associadas à melhora dos sintomas de angina e dos parâmetros de isquemia pelo TE em poucos e pequenos ensaios clínicos randomizados, com um número bem reduzido de pacientes.^331,332^ Um grande ensaio clínico randomizado com mais de 5.000 pacientes e seguimento médio de quase 5 anos, recentemente publicado, demonstrou que o alopurinol não foi capaz de reduzir os eventos cardiovasculares em pacientes com SCC e sem história prévia de gota. Não houve evidência de diferença na incidência de eventos adversos graves entre os grupos, mas também não houve benefício do efeito do alopurinol em parâmetros de qualidade de vida e questionários de angina.^333^

### 5.11. Individualização do Tratamento Antianginoso

Diretrizes internacionais tradicionalmente têm recomendado fármacos para tratamento da angina, classificando-as como de primeira linha ou de segunda linha.^1^ A Diretriz de Doença Coronária Estável da SBC, de 2014, também recomendava fármacos de primeira linha, de segunda linha, de terceira linha e até de quarta linha.^16^

Entretanto, entre os antianginosos atualmente recomendados, não há evidências que demonstrem superioridade de uma classe de drogas em relação às outras em termos de eficácia antianginosa ou outros desfechos.^260,334^ Uma revisão sistemática de artigos escritos nos últimos 50 anos incluiu estudos randomizados, duplo-cegos, comparando grupos paralelos de tratamento de angina em pacientes com SCCs. Em nenhum dos estudos dessa análise houve evidência de que uma droga é superior às outras no tratamento da angina ou no prolongamento do tempo total de exercício.^334^

As medicações antianginosas mais novas, como ivabradina, ranolazina e trimetazidina, têm evidências substanciais de estudos clínicos randomizados contemporâneos que dão suporte à sua segurança e eficácia.^335,336^

Dessa forma, tem-se proposto uma abordagem individualizada para os pacientes com angina, ajustando-se as drogas antianginosas às características clínicas de cada paciente, seu perfil hemodinâmico, suas comorbidades e o mecanismo de isquemia, sem uma categorização em drogas de primeira linha ou de segunda linha.^276,335,336^

Além disso, a combinação precoce de 2 ou 3 antianginosos com mecanismos de ação diferentes e complementares frequentemente é necessária para controle dos sintomas, uma vez que pacientes com angina podem apresentar várias comorbidades e diferentes mecanismos de angina, os quais podem estar sobrepostos.^335^ Essa estratégia pode reduzir a frequência de angina e o consumo de nitratos, aumentar o tempo de esforço e o tempo de aparecimento de angina ou a isquemia ao teste de esforço.^1^

Independentemente da estratégia inicial, a resposta à terapia antianginosa deveria ser reavaliada em 30 dias do início do tratamento.^1^ Se disponível, a telemedicina pode ser oferecida para reavaliação dos pacientes em intervalos menores.

Como os sintomas de angina podem ter recorrência ou remissão ao longo do tempo, e as placas coronarianas podem se tornar quiescentes, uma avaliação apropriada da angina requer um acompanhamento cuidadoso e a averiguação sistemática dos sintomas relatados pelos pacientes e da qualidade de vida.^27^ Dessa forma, um horizonte de tempo suficiente (3 a 6 meses) frequentemente é necessário para que o tratamento médico baseado em evidências seja adequadamente avaliado quanto à sua efetividade.^27,337^

**Figura 17:**
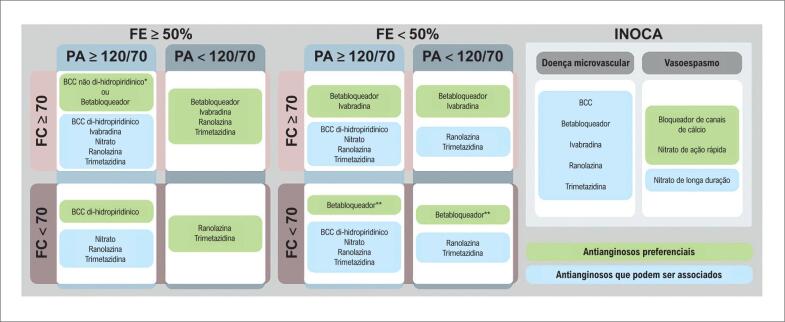
Abordagem brasileira para tratamento personalizado da angina. abordagem para tratamento personalizado da angina, considerando fração de ejeção, pressão arterial, frequência cardíaca e mecanismos por trás da isquemia miocárdica. Os antianginosos em cada quadro estão dispostos em ordem alfabética. Em verde, estão os antianginosos preferenciais em cada situação descrita; em azul, estão os antianginosos que podem sem associados para efeito aditivo no controle dos sintomas. BCC: bloqueadores de canais de cálcio; FC: frequência cardíaca (em bpm); FE: fração de ejeção; INOCA: isquemia sem obstrução das artérias coronárias (ischemia with no obstructive coronary arteries); PA: pressão arterial (em mmHg). *Não devem ser utilizados em associação com ivabradina e BCC di-hidropiridínicos. **Não utilizar em FC ≤ 55 bpm. As cores dessa figura não representam os graus de recomendação e níveis de evidência.

Abordagem individualizada (Figura 17):

Drogas especificas ou combinações de drogas são preferidas dependendo da fisiopatologia ou das comorbidades presentes.^335^

Frequência cardíaca elevada: medicações redutoras da FC, como betabloqueadores, BCCs não di-hidropiridínicos (verapamil e diltiazem) e ivabradina são as drogas preferidas quando a FC é > 70 bpm (Figura 17). A adição de ivabradina ao betabloqueador é segura e útil quando a FC permanece > 70 bpm,^276^ mas a combinação de ivabradina com diltiazem ou verapamil é contraindicada.^303^ A combinação de betabloqueadores com verapamil ou diltiazem deve ser utilizada com cautela devido ao risco de bloqueio atrioventricular de alto grau. Ranolazina e trimetazidina podem ser coadministradas. A dose das medicações antianginosas deve ser reduzida se a FC estiver < 50 spm.

Frequência cardíaca baixa (Figura 17): não se devem utilizar medicações redutoras da FC quando ela estiver < 55 spm. A preferência deveria ser por BCCs di-hidropiridínicos e nitratos, porque esses agentes podem aumentar a FC, provocando um reflexo simpático. Outros agentes, como a ranolazina e a trimetazidina, podem ser considerados.

**Figura 18:**
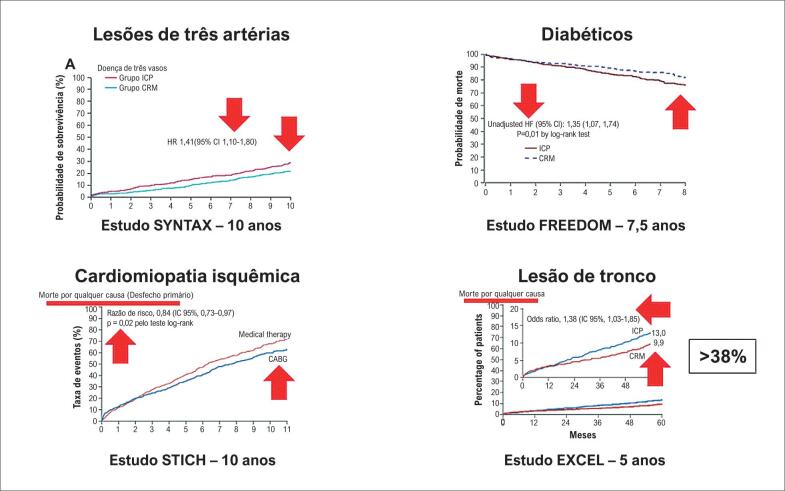
Redução de mortalidade por todas as causas em longo prazo. EXCEL: Evaluation of XIENCE Everolimus Eluting Stent Versus Coronary Artery Bypass Surgery for Effectiveness of Left Main Revascularization; FREEDOM: Future Revascularization Evaluation in Patients with Diabetes Mellitus: Optimal Management of Multivessel Disease; STICH: Surgical Treatment for Ischemic Heart Failure; SYNTAX: Synergy Between PCI with Taxus and Cardiac Surgery.

Hipertensão arterial: betabloqueadores e BCCs di-hidropiridínicos são preferidos quando há hipertensão (Figura 18). A pressão arterial não deveria ser reduzida abaixo de 120/70 mmHg, porque há uma associação de níveis mais baixos de pressão com aumento de risco de eventos cardiovasculares adversos em pacientes com SCCs.^338^ Entretanto, não se sabe se essa curva em J também se aplica a pacientes já revascularizados.

Hipotensão arterial: agentes antianginosos que reduzem significativamente a pressão arterial, como BCCs, nitratos e betabloqueadores, não deveriam ser utilizados em pacientes com angina e pressão arterial baixa, porque podem piorar a perfusão coronariana. Em uma análise de mais de 22 mil pacientes do estudo CLARIFY, pressão sistólica < 120 mmHg e pressão diastólica < 70 mmHg foram associadas ao aumento do risco de mortalidade cardiovascular, infarto e AVC.^338^ Nesses pacientes, a preferência é por ivabradina, se a FC também estiver elevada, trimetazidina ou ranolazina (Figura 17).

Disfunção do VE e IC: quando a angina está presente em pacientes com disfunção do VE, com ou sem IC, a indicação baseada em evidências é do uso de betabloqueadores (Figura 17), que podem reduzir a angina e, ao mesmo tempo, efetivamente reduzir a morbimortalidade cardiovascular nesses pacientes.^339,340^ Esses efeitos benéficos parecem estar relacionados diretamente ao efeito de redução da FC dos betabloqueadores, dessa forma, betabloqueadores com atividade simpatomimética intrínseca deveriam ser evitados. Se a FC permanecer > 70 bpm, apesar da maior dose tolerada do betabloqueador, a ivabradina deveria ser considerada. O estudo SHIFT^301^ mostrou um benefício prognóstico adicional da associação de ivabradina à terapia médica otimizada e baseada em evidências em pacientes com IC e fração de ejeção reduzida. Benefícios semelhantes foram vistos no subgrupo de pacientes com angina.^341^ Diltiazem e verapamil não deveriam ser usados nesse grupo de pacientes, porque podem piorar a disfunção do VE.^342^ Os BCCs di-hidropiridínicos podem ser usados com cautela. Uma metanálise de pequenos estudos em pacientes com disfunção do VE e/ou IC sugere que a trimetazidina pode ser benéfica se associada às terapias recomendadas.^343^

Fibrilação atrial: a FA pode agravar os sintomas de angina devido a FC elevada. Então, betabloqueadores e BCCs não di-hidropiridínicos são preferidos quando essa comorbidade está presente. Devido à seletividade pelos canais If, a ivabradina é inefetiva em pacientes com FA e pode até mesmo aumentar o risco dessa arritmia. Uma metanálise mostrou que o tratamento com ivabradina está associado a um aumento de 1,15 no risco relativo de FA.^344^ BCCs di-hidropiridínicos e nitratos deveriam ser evitados, porque podem causar aumento da FC. A associação de ranolazina, que tem se mostrado capaz de suprimir arritmias supraventriculares e FA, pode ser útil. A trimetazidina também pode ser associada.^317,345^

Diabetes: o tratamento da angina em pacientes com diabetes requer drogas com ação metabólica neutra ou positiva. A eficácia da ranolazina em pacientes diabéticos com angina foi avaliada em um estudo duplo-cego controlado por placebo.^309^ A ranolazina reduziu significativamente os níveis de hemoglobina glicada, de glicose de jejum e pós-prandial de 2 horas e mitigou a ocorrência de angina, aumentando a tolerância ao esforço.^309^ A ranolazina deveria ser a droga preferida nesse subgrupo de pacientes.^309,346^ A trimetazidina também poderia ter uma ação positiva, melhorando a utilização de glicose sob condições de isquemia, e alguns dados positivos estão disponíveis em subgrupos de pacientes com diabetes em estudos com a trimetazidina.^347^ Contudo, esses dados são de estudos com pequeno número de pacientes diabéticos, que tiveram desenho aberto. Tradicionalmente, betabloqueadores foram relacionados ao aparecimento de novos casos de diabetes e piora do controle glicêmico.^348^ Entretanto, betabloqueadores com propriedades vasodilatadoras, como o carvedilol e o nebivolol, têm sido associados à melhora da sensibilidade à insulina, superando a limitação metabólica dos betabloqueadores tradicionais. Os outros agentes podem ser utilizados para redução de angina e isquemia.^349^

Doença renal crônica: estudos clínicos frequentemente excluem pacientes com DRC, uma comorbidade frequente em pacientes com doença coronariana, portanto, a eficácia sobre os antianginosos é escassa nessa condição.^349^ A ranolazina e a trimetazidina não deveriam ser prescritas quando a taxa de filtração glomerular é < 30 mL/min/1,73 m^2^. Não há contraindicações para as outras drogas antianginosas.^3835^

Doença pulmonar obstrutiva crônica (DPOC): as evidências sugerem que os betabloqueadores com seletividade beta-1 são, geralmente, bem tolerados em pacientes com DPOC e podem melhorar a sobrevida.^350^ Devido à sua alta seletividade beta-1, o bisoprolol é o único betabloqueador não contraindicado na DPOC. A coexistência de asma, assim como a DPOC com broncorreatividade são, contudo, uma contraindicação aos betabloqueadores. Nesses pacientes, quando há necessidade de redução da FC, são preferidos ivabradina, diltiazem ou verapamil. Na presença de hipertensão pulmonar e disfunção do ventrículo direito, não são indicados BCCs não di-hidropiridínicos e betabloqueadores não seletivos.^335^

Doença arterial periférica: em 2013, a Associação Médica Britânica declarou que betabloqueadores eram contraindicados na DAP severa.^351^ No mesmo ano, uma revisão sistemática Cochrane não mostrou fortes evidências a favor ou contra o uso de betabloqueadores na DAP.^352^ Devido à escassez de dados confiáveis e contemporâneos, sugere-se que betabloqueadores deveriam ser evitados ou usados com cautela em pacientes com angina e doença arterial periférica.^335^ Da mesma forma (especialmente em casos de isquemia crítica), vasodilatadores como BCCs e nitratos deveriam ser evitados, porque reduções agudas da pressão arterial são deletérias. Os demais antianginosos (ivabradina, ranolazina e trimetazidina) são preferidos.^335^

Defeitos na condução atrioventricular: betabloqueadores e BCCs não di-hidropiridínicos reduzem a condução atrioventricular e podem causar bloqueio atrioventricular completo e dissincronia intraventricular em pacientes com essa condição.^353^ Então, há uma clara contraindicação para seu uso em pacientes com bloqueio atrioventricular de segundo grau. Outros agentes antianginosos deveriam ser usados para esses pacientes.^335^

Hipertireoidismo: pacientes com hipertireoidismo têm um aumento de três vezes no risco de FA e IC.^354^ Altos níveis de hormônio tireoidiano também afetam os fatores que determinam o aumento do consumo de oxigênio no miocárdio, levando ao aparecimento de angina em pacientes com e sem doença coronariana angiograficamente demonstrável. Os hormônios tireoidianos também podem causar espasmos coronarianos.^355^ Os tratamentos preferidos nesses pacientes são betabloqueadores não seletivos (propranolol), diltiazem, verapamil ou ivabradina, se betabloqueadores forem contraindicados. Vasodilatadores não deveriam ser usados pelo risco de taquicardia reflexa.^335^

Angina vasoespástica: as drogas preferidas para prevenir e/ou tratar espasmo coronariano são os BCCs. Os nitratos de ação prolongada podem ser utilizados em pacientes que permanecem sintomáticos (Figura 17). Todos os BCCs podem prevenir espasmo de aproximadamente 90% dos pacientes. Nitratos de ação prolongada são eficazes, mas a administração intermitente é importante para prevenir intolerância ao nitrato. Betabloqueadores são contraindicados, porque podem precipitar espasmo, deixando a vasoconstrição alfa-mediada sem oposição da vasodilatação beta-mediada. Em pacientes com angina refratária (AR), altas doses de BCCs podem ser tentadas.^335^

Angina microvascular: não há evidência conclusiva disponível para indicar uma classe específica de antianginosos, provavelmente pelo conhecimento limitado das causas da angina microvascular e da resposta variável aos diferentes tratamentos. Durante muitos anos, agentes tradicionais, como betabloqueadores, BCCs e nitratos, eram considerados a única opção, apesar de 20 a 30% dos pacientes permanecerem sintomáticos. Betabloqueadores podem ser preferidos quando há evidência de aumento da atividade adrenérgica.^1^ A ranolazina também pode reduzir a compressão mecânica da microcirculação coronária.^356,357^ Além disso, a ranolazina pode melhorar a autorregulação coronária.^358^ Um estudo pequeno com ranolazina sugeriu melhora dos sintomas de angina em mulheres com angina microvascular.^359^ Subsequentemente, em um estudo duplo-cego, randomizado, placebo-controlado, em mulheres com angina de esforço, mas sem doença coronariana obstrutiva, a ranolazina não levou a benefícios significativos, exceto em pacientes com redução da reserva de fluxo coronariano.^360^ De forma semelhante, estudos demostraram que a ivabradina melhorou o fluxo colateral e a reserva de fluxo coronário em pacientes com angina microvascular. Os efeitos da ivabradina foram superiores ao bisoprolol, apesar de reduções semelhantes da FC. Então, o tratamento da angina microvascular é bastante desafiador e necessariamente empírico. A redução da FC com betabloqueadores, BCCs não di-hidropiridínicos (diltiazem, verapamil) ou ivabradina pode ser considerada para aumentar o tempo de diástole e a perfusão coronariana. A coadministração de ranolazina ou trimetazidina pode ser útil (Figura 17). Em pacientes com aumento da percepção da dor, antagonistas da adenosina e drogas efetivas em síndromes de dor crônica, como a imipramina, são outra opção terapêutica.^335^

A efetividade de uma estratégia individualizada de tratamento de pacientes com angina por doença coronariana não obstrutiva (INOCA) foi investigada no estudo CorMiCa (*Coronary Microvascular Angina*), o qual randomizou 151 pacientes para tratamento médico estratificado (baseado em resultados de reserva de fluxo coronário, índice de resistência microvascular e teste de acetilcolina) contra um grupo-padrão (incluindo um procedimento de intervenção diagnóstica "*sham*"). Os pacientes tinham um diagnóstico de angina microvascular, angina vasoespástica ou dor torácica não cardíaca. Em 1 ano, houve uma diferença significativa nos escores de angina, favorecendo pacientes do grupo de tratamento estratificado.^361^

A escolha inicial do tratamento antianginoso deve ser reavaliada a cada consulta, levando-se em consideração modificações nas características clínicas do paciente (PA, FC, função ventricular), mecanismos de angina e isquemia, presença de comorbidades, tolerância às medicações, potenciais interações medicamentosas e desenvolvimento de efeitos colaterais (Tabela 21).

**Tabela 21:** Tratamento clínico (farmacológico) para controle ótimo de sintomas

Recomendação	Grau de recomendação	Nível de evidência
A escolha inicial do tratamento antianginoso deve ser individualizada, levando-se em consideração comorbidades, perfil hemodinâmico, tolerância, interações medicamentosas e preferencias do médico e paciente, além de disponibilidade das medicações.	**I**	**C**
A combinação de antianginosos com mecanismos de ação diferente é mais efetiva no controle de sintomas do que a utilização de um antianginoso em monoterapia.	**I**	**C**
Enquanto sintomático, o paciente deve ser avaliado a cada 30 dias, com ajuste progressivo da terapêutica antianginosa.	**I**	**C**
Se disponível, a telemedicina pode ser oferecida para reavaliação dos pacientes em intervalos menores.	**IIa**	**C**
Bisoprolol, carvedilol e succinato de metoprolol são recomendados no tratamento da angina em pacientes com FEVE ≤ 40%.	**I**	**A**
Nitratos de ação curta são recomendados para o alívio imediato da angina de esforço.	**I**	**B**
Betabloqueadores, bloqueadores de canal de cálcio, trimetazidina e/ou ranolazina são opções para tratamento da angina (em monoterapia ou terapia combinada).	**I**	**B**
Ivabradina deve ser considerada no tratamento da angina em pacientes com disfunção ventricular e que permanecem com FC ≥ 70 bpm apesar do uso de betabloqueadores ou quando esses são contraindicados.	**IIa**	**B**
Ivabradina pode ser considerada para tratamento da angina em pacientes sem disfunção ventricular e com FC ≥ 70 bpm e ritmo sinusal.	**IIb**	**B**
Nitratos de ação prolongada podem ser considerados para o tratamento da angina quando paciente permanece sintomático com o uso de outros antianginosos.	**IIb**	**B**
Nitratos de ação prolongada não devem ser utilizados em monoterapia, salvo impossibilidade de utilização de outros antianginosos.	**III**	**C**

FC: frequência cardíaca; FEVE: fração de ejeção do ventrículo esquerdo.

## 6. Tratamento com Medidas Invasivas

### 6.1. Tratamento Percutâneo para Controle de Sintomas e Redução do Risco de Eventos

#### 6.1.1. Escores de Complexidade Angiográfica na Tomada de Decisão

A carga aterosclerótica total, o grau de miocárdio em risco e a complexidade das lesões coronárias, juntamente com a expectativa de revascularização completa, risco residual e o risco de eventos clínicos, são elementos cruciais na determinação do tipo de revascularização mais adequado para pacientes com DAC.^362,363^ Múltiplos fatores contribuem para a avaliação da complexidade da DAC, como destacado na Tabela 22.

**Tabela 22:** Características angiográficas que contribuem para o aumento da complexidade da doença arterial coronariana

Doença arterial multivascular
Grande área total de miocárdio em risco (ex.: lesão em tronco de coronária esquerda ou proximal de descendente anterior)
Doença difusa em um vaso coronário alvo de revascularização
Oclusão crônica
Bifurcações complexas ou trifurcações
Calcificações severas
Tortuosidade acentuada
Comprimento total da lesão > 20 mm

O escore SYNTAX, originado a partir do estudo SYNTAX, desempenha um papel fundamental nesse processo ao oferecer uma medida objetiva para classificar a carga aterosclerótica total e a complexidade anatômica da DAC em pacientes com DAC multiarterial.^363^ Além disso, a capacidade do escore SYNTAX (*Synergy Between PCI with TAXUS™ and Cardiac Surgery*) como preditor independente de ECAM a longo prazo e mortalidade foi estabelecida dentro da coorte do próprio estudo SYNTAX e subsequentemente validada em estudos externos envolvendo pacientes tratados com intervenção coronariana percutânea (ICP).^362^ No entanto, o escore SYNTAX não funciona como ferramenta de estratificação de risco para pacientes submetidos a cirurgia de revascularização do miocárdio (CRM).^364^

Recentemente, o desenvolvimento retrospectivo dos escores SYNTAX II e SYNTAX Score II revisado em 2020, a partir da mesma coorte do estudo SYNTAX, incorporou variáveis clínicas adicionais às variáveis anatômicas, proporcionando uma avaliação mais abrangente.^365,366^ Esses escores, embora demonstrem uma capacidade de discriminação modesta na previsão de eventos clínicos adversos pós-revascularização, são ferramentas valiosas no auxílio à decisão clínica.^364,366–368^

#### 6.1.2. Recomendação

O escore SYNTAX permanece como o escore de risco mais amplamente utilizado e validado para orientar a escolha da revascularização em pacientes com doença multiarterial. No entanto, importantes limitações desse escore incluem o sistema de pontuação complexo necessário para cada lesão e a grande variabilidade interobservador em seu cálculo.^369^ Além disso, a ausência de variáveis clínicas limita seu uso na estimativa do risco de eventos clínicos após a CRM (Tabela 23).

**Tabela 23:** Recomendação de utilização do escore SYNTAX para estimar a complexidade angiográfica

Recomendação	Grau de recomendação	Nível de evidência
Em pacientes com DAC multiarterial, a avaliação da complexidade da DAC, como o escore SYNTAX, pode ser útil para estimar o risco de eventos e orientar a revascularização	**IIb**	**B**

Ao estimar a complexidade da doença de um paciente, é importante considerar variáveis que contribuam para essa complexidade, o que pode impactar o sucesso e os resultados da revascularização.^367,370^

### 6.2. Avaliação Funcional Invasiva

#### 6.2.1. Avaliação de Estenoses Coronárias Epicárdicas

A FFR é uma técnica invasiva utilizada para avaliar a gravidade funcional de estenoses coronarianas. Baseia-se na medição da pressão intracoronária durante a hiperemia máxima, que é induzida pela administração de agentes vasodilatadores, como a adenosina.^371^

O valor da FFR é a razão entre a pressão distal à estenose e a pressão aórtica, refletindo a capacidade da estenose em limitar o fluxo sanguíneo. Um valor de FFR ≤ 0,80 é, geralmente, considerado indicativo de isquemia significativa, justificando intervenção para revascularização. A FFR é o padrão atual para a avaliação funcional da gravidade das lesões em pacientes com estenose coronariana de grau intermediário (geralmente entre 40 e 70%) sem evidência de isquemia em testes não invasivos ou em pacientes com doença multiarterial.

Além da FFR, outros índices derivados de pressão, como iFR (*instantaneous wave-free ratio*), dPR (*diastolic pressure ratio*), RFR (*resting full-cycle ratio*), DFR (*diastolic fractional reserve*) etc., têm se mostrado úteis na avaliação funcional invasiva. Esses métodos oferecem a vantagem de não necessitarem da indução de hiperemia máxima, proporcionando uma avaliação mais confortável e rápida para o paciente. Estudos comparativos sugerem que esses índices não hiperêmicos possuem boa acurácia em identificar lesões isquêmicas em comparação com a FFR.^372^

Mais recentemente, avaliações funcionais têm sido derivadas de angiografias coronárias invasivas. O *quantitative flow ratio* ou o *Murray quantitative flow ratio* (QFR/μFR; Medis, Reino dos Países Baixos, ou Pulse Medical, China) são técnicas que utilizam imagens angiográficas invasivas para calcular um índice fisiológico que reflete a severidade da estenose, sem a necessidade de um fio de pressão ou a administração de adenosina.

O QFR/μFR é o único índice fisiológico baseado em angiografia que foi prospectivamente validado.^373^ Essa medida tem mostrado uma boa acurácia em relação à FFR e estar associada a melhores desfechos clínicos quando usada para decidir sobre a revascularização coronariana, em comparação com a angiografia convencional.^373,374^

#### 6.2.2. Recomendação

No estudo FAME-2 (*Fractional Flow Reserve versus Angiography for Multivessel Evaluation 2*), pacientes com DAC crônica e lesões com severidade angiográfica ≥ 50% e FFR ≤ 0,8 foram randomizados para tratamento medicamentoso vs. ICP e tratamento medicamentoso.^145^ A ICP reduziu significativamente o desfecho primário composto por morte, IM ou revascularização urgente.^145^ Em pacientes com DAC multiarterial, a FFR é particularmente útil para guiar a decisão sobre quais lesões necessitam de revascularização. A abordagem baseada na FFR permite uma estratégia mais precisa, focando apenas nas lesões que causam isquemia significativa, o que pode resultar em menos implantes de stent*s* e menores taxas de complicações associadas.^375^

Dois recentes grandes ensaios clínicos randomizados demonstraram resultados amplamente comparáveis entre estratégias de revascularização guiadas por FFR e iFR em pacientes com estenose de grau intermediário. A revascularização foi indicada em ambos os ensaios se a FFR fosse < 0,80 ou se o iFR fosse < 0,89. No ensaio DEFINE FLAIR (*Functional Lesion Assessment of Intermediate Stenosis to Guide Revascularization*), o desfecho primário de MACE em 1 ano ocorreu em 6,8% dos pacientes randomizados para revascularização guiada por iFR vs. 7,0% nos pacientes randomizados para revascularização guiada por FFR (p < 0,001 para não inferioridade; HR 0,95; IC 95% 0,68–1,33; p = 0,78; HR 1,12; IC 95% 0,79–1,58; p = 0,53).^376^

No ensaio SWEDEHEART (*Swedish Web-System for Enhancement and Development of Evidence-Based Care in Heart Disease Evaluated According to Recommended Therapies*), com 5 anos de seguimento, o desfecho primário de morte por qualquer causa, IAM não fatal ou revascularização não planejada foi de 21,5% no grupo iFR e de 19,9% no grupo FFR (HR 1,09; IC 95% 0,90–1,33).^377^

O estudo FAVOR III China objetivou estabelecer se os desfechos clínicos poderiam ser melhorados pela seleção de lesões para ICP usando o QFR/*μ*FR vs. estimativa visual. O desfecho primário (morte por qualquer causa, IM ou revascularização conduzida por isquemia) em 1 ano ocorreu em 5,8% no grupo guiado por fisiologia e em 8,8% no grupo guiado pela avaliação visual (HR 0,65; IC95% 0,51–0,83; p = 0,0004), impulsionado por menos IMs e revascularizações conduzidas por isquemia no grupo QFR-guiado do que no grupo angiografia-guiada.

#### 6.2.3. Angina Microvascular

Pacientes com angina microvascular tipicamente apresentam angina relacionada ao exercício, evidências de isquemia em testes não invasivos sem estenoses ou apresentam estenoses leves a moderadas (40 a 60%) na angiografia invasiva.

A presença de disfunção microcirculatória em pacientes com angina tem implicações prognósticas, provavelmente porque a maioria das evidências mais recentes tem sido baseada no acompanhamento de pacientes em quem as anormalidades na microcirculação foram objetivamente documentadas por meio de técnicas invasivas ou não invasivas.^378–380^

#### 6.2.4. Recomendação

A RFC e a resistência microcirculatória podem ser medida no laboratório de cateterização combinando pressão intracoronária com dados baseados em termodiluição (para calcular o IMR). Para fins de tomada de decisão, valores de IMR > 25 unidades ou RFC < 2,0 são indicativos de função microcirculatória anormal (Tabela 24).^361^

**Tabela 24:** Recomendações para o Uso de Avaliação Funcional Invasiva na Doença Coronariana Crônica

Recomendação	Grau de recomendação	Nível de evidência
Em pacientes com doença coronariana crônica que apresentam angina ou um equivalente anginoso, sem avaliação prévia de isquemia e estenoses angiograficamente intermediárias, recomenda-se o uso de avaliação funcional invasiva antes de prosseguir com a intervenção coronária percutânea.	**I**	**A**
A medição da resistência microcirculatória deve ser considerada em pacientes com sintomas persistentes, mas artérias coronárias que estejam angiograficamente normais ou que tenham estenoses moderadas com reserva fracionada de fluxo e índices não hiperêmicos preservados.	**IIa**	**C**

Tanto a RFC quanto o IMR são tipicamente medidos durante o uso de vasodilatadores intravenosos, como adenosina. A possibilidade de angina de origem microcirculatória deve ser considerada em pacientes com angina bem definida, testes funcionais não invasivos anormais e vasos coronários que estejam normais ou apresentem estenose leve e funcionalmente não significativa na angiografia invasiva ou angioTC.

### 6.3. Métodos de Imagem Intravascular

#### 6.3.1. Uso de Imagens Intravasculares no Diagnóstico e na Avaliação de Estenoses Coronárias

Os principais métodos de imagem intravascular em nosso meio são o ultrassom intracoronário (*intravascular ultrasound* [IVUS]) e a tomografia de coerência óptica (*optical coherence tomography* [OCT]). O IVUS é uma modalidade de processamento de imagens por meio de ecos de som refletidos pelos tecidos vasculares, que possui uma resolução axial de aproximadamente 150 μm.^381^ A OCT, por sua vez, utiliza feixes ópticos com bandas próximas ao espectro de onda infravermelha como sua fonte de energia, conferindo resolução axial de 10 a 15 *μ*m.^382^

Potenciais usos clínicos da imagem intravascular para avaliação diagnóstica em pacientes considerados para revascularização miocárdica incluem avaliação da gravidade da estenose em lesões com estenose de grau intermediário, avaliação da morfologia da lesão em lesões ambíguas com avaliação angiográfica e caracterização da composição da placa.

#### 6.3.2. Recomendação

Em relação à avaliação de estenose de grau intermediário (classicamente definida como entre 50 e 69%), vários estudos analisaram o ponto de corte ideal da área mínima do lúmen para identificar lesões hemodinamicamente relevantes (Tabela 25).

**Tabela 25:** Recomendações para o Uso de Imagens Intravasculares no Diagnóstico e na Avaliação de Estenoses Coronárias

Recomendação	Grau de recomendação	Nível de evidência
O ultrassom intracoronário deve ser considerado para a avalição da gravidade de estenoses em tronco de coronária esquerda.	**IIa**	**B**
O uso de imagem intracoronária (IVUS ou OCT) pode ser considerado na avaliação de estenoses coronárias intermediárias.	**IIb**	**B**

IVUS: ultrassom intracoronário (intravascular ultrasound); OCT: tomografia de coerência óptica (*optical coherence tomography*).

Um registro prospectivo mostrou uma correlação moderada geral da área luminal mínima (ALM) com valores de FFR, com valores de corte para detecção de estenose hemodinamicamente relevante (< 2,4, < 2,7 e < 3,6 mm^2^) dependendo do tamanho do vaso (diâmetros de referência dos vasos < 3,0, 3,0–3,5 e > 3,5 mm, respectivamente).^383^ Em um estudo randomizado, 1.682 pacientes foram avaliados para se submeterem a um procedimento de ICP de lesões moderadas (entre 40 e 70%) guiado por FFR ou IVUS. Nesse estudo, ambos os métodos foram utilizados para determinar a necessidade da ICP e para avaliar o sucesso do procedimento. No grupo IVUS, os critérios para ICP eram uma ALM de 3 mm^2^ ou menor ou entre 3 e 4 mm^2^ com uma carga de placa superior a 70%. O IVUS foi não inferior à FFR para o desfecho primário composto de morte, IM ou revascularização em 24 meses após a randomização.^384^

Outro estudo randomizado comparou a indicação de ICP guiada por OCT vs. ICP guiada por FFR em pacientes com lesões coronarianas intermediárias. No grupo OCT, a intervenção foi realizada considerando os critérios de área de estenose ≥ 75% ou de 50 a 75% com ALM < 2,5 mm^2^ ou ruptura de placa. Esse grupo apresentou uma menor taxa de eventos compostos em 13 meses (MACE e angina significante) comparado ao grupo com intervenção guiada por FFR (8,0% vs. 14,8%; p = 0,048).^385^ No entanto, trata-se de um estudo unicêntrico e com tamanho amostral total relativamente pequeno (n = 350 pacientes). De uma forma geral, a avaliação hemodinâmica com FFR deve ser preferida para avalição de estenoses coronárias intermediárias.

A presença de estenose em tronco de coronária esquerda de grau intermediário não é incomum, e a avaliação angiográfica pode ser desafiadora. A avaliação da doença aterosclerótica em tronco de coronária esquerda de grau intermediário utilizando IVUS em pacientes considerados para CRM ou ICP é respaldada por dados de vários estudos observacionais.

Em um estudo prospectivo multicêntrico, o corte para realização de revascularização foi o de ALM < 6 mm^2^, mantendo os pacientes em tratamento clínico isolado caso ALM ≥ 6 mm^2^.^386^ Após um acompanhamento de 2 anos, a sobrevida livre de morte cardíaca foi semelhante em ambos os grupos (97,7% no grupo de tratamento conservador e 94,5% no grupo revascularizado). Outro estudo sugeriu que o adiamento da intervenção em 131 pacientes com uma ALM > 7,5 mm^2^ mostrou resultados clínicos favoráveis.^387^

Em pacientes asiáticos, com tamanhos cardíacos geralmente menores, estudos têm sugerido que uma ALM pelo IVUS de 4,5 a 4,8 mm^2^ pode ser a mais apropriada como ponte de corte para intervenção.^388^

#### 6.3.3. Uso de Imagens Intravasculares para Guiar Intervenção Coronária Percutânea

As modalidades de imagem intravascular permitem a avaliação das características da placa e o dimensionamento preciso do vaso durante a ICP. Isso possibilita uma estratégia mais precisa, identificação dos locais ideais para implante do stent, preparo mais adequados das lesões, melhor expansão do stent, prevenção de malaposições importantes e identificação de dissecções significativas nas bordas do stent.

#### 6.3.4. Recomendação

O uso de IVUS ou OCT para guiar o implante de stent tem se refletido em redução de desfechos clínicos em registros de ensaios clínicos randomizados e metanálises.^144,389–396^ O benefício dessa estratégia guiada por imagem intracoronária se deve pela redução de MACE, morte cardíaca, trombose de stent, revascularização da lesão-alvo e revascularização do vaso-alvo (Tabela 26).

**Tabela 26:** Recomendações para a Utilização de Imagens Intravasculares no Planejamento e na Condução da Intervenção Coronária Percutânea

Recomendação	Grau de recomendação	Nível de evidência
O uso de imagem intracoronário (IVUS ou OCT) deve ser utilizado para guiar intervenções coronárias percutâneas complexas*	**I**	**A**
O uso de imagem intracoronário (IVUS ou OCT) pode ser considerado para guiar intervenções coronárias percutâneas de baixa complexidade	**IIb**	**B**

IVUS: ultrassom intracoronário (intravascular ultrasound); OCT: tomografia de coerência óptica (*optical coherence tomography*).

* Cabem ao operador o julgamento clínico sobre a necessidade de emergência para realização da intervenção coronariana percutânea (em que o uso da imagem intravascular pode retardar a resolução do caso), a avaliação da capacidade do cateter de imagem intracoronário em cruzar a estenose coronária (influenciada pela presença de calcificação, tortuosidade etc.) e a aptidão médica para realização do procedimento adequadamente.

Até o momento de confecção desta diretriz, um total de 20 estudos randomizados, 34 metanálises e 85 registros compararam desfechos clínicos da ICP guiada por imagem intracoronária vs. guiada por angiografia. Apesar disso, a adoção da imagem intravascular para guiar a ICP permanece baixa em nosso meio. Isso pode explicado pela falta de conhecimento e treinamento dos operadores; a percepção de tempo adicional do procedimento; os custos adicionais do procedimento; e dependendo dos sistemas de saúde específicos e a falta de vínculo com reembolso.^388^

ICPs complexas como aquelas em tronco de coronária esquerda, bifurcações verdadeiras com grande área de miocárdio em risco, falha de stents previamente implantados e lesões longas podem ter um benefício mais evidente do uso da imagem intravascular.^394^ No entanto, o benefício do uso generalizado da imagem para guiar intervenção em todas as estenoses coronárias não está evidente.^149,394^ Também cabem ao operador o julgamento clínico sobre a necessidade de emergência para a realização da ICP (em que o uso da imagem intravascular pode retardar a resolução do caso), a avaliação da capacidade do cateter de imagem intracoronário em cruzar a estenose coronária (influenciada pela presença de calcificação, tortuosidade etc.) e a aptidão médica para realização do procedimento adequadamente.

### 6.4. Intervenção Coronariana Percutânea como Estratégia para Melhora Prognóstica

O conjunto das evidências derivadas de estudos randomizados sugere que o tratamento intervencionista percutâneo pode modificar o risco de óbito ou infarto não fatal em subgrupos específicos de pacientes com DCC, quando comparado ao tratamento conservador, muito embora isso não seja aplicado à totalidade dos pacientes portadores de DAC.

A análise de 10 anos de evolução do estudo brasileiro MASS (*Medicine, Angioplasty and Surgery Study*), que randomizou pacientes multiarteriais para revascularização cirúrgica, intervenção percutânea ou tratamento medicamentoso isolado, demonstrou que a abordagem conservadora se associou a maior mortalidade cardíaca (p = 0,02) e maior incidência de IM (p = 0,01), quando comparada com cirurgia de revascularização.^398^

No estudo multicêntrico randomizado ISCHEMIA, que comparou o tratamento invasivo (cirúrgico ou percutâneo) ao conservador, o subgrupo de pacientes com maior severidade anatômica apresentou menor incidência de óbito ou infarto aos 4 anos, mas não de mortalidade isoladamente, quando tratado invasivamente (diferença absoluta de −6,3%; IC 95% −12,4% a −0,2%).^399^ Adicionalmente, nesse mesmo estudo, observou-se que pacientes tratados por intervenção invasiva apresentaram menor incidência de infarto espontâneo (HR ajustado 0,53; IC 95% 0,41–0,69; p < 0,0001) e que o risco de óbito na evolução daqueles com infarto espontâneo era aumentado em mais de duas vezes (p < 0,001).^400^

Uma metanálise que envolveu 19.806 pacientes incluídos em 25 estudos randomizados comparativos entre revascularização eletiva vs. terapia medicamentosa isolada, mas que incluiu vários estudos de mais de 25 anos, quando não havia terapêutica medicamentosa atual, acompanhados durante um seguimento médio de 5,7 anos, demonstrou uma redução significativa da mortalidade cardiovascular nos pacientes tratados invasivamente (*rate ratio* [RR] 0,79; IC 95% 0,67–0,93; p < 0,01).^401^ Todavia, os estudos dos últimos 10 a 15 anos não mantêm esse resultado, exceto para cirurgia de revascularização, especialmente o MASS-2, inclusive com maior eventos com angioplastia do que no tratamento clínico, e o ISCHEMIA com 28% sendo tratados com cirurgia, provavelmente os pacientes mais graves, que têm mais vasos doentes e que podem se beneficiar desse tratamento.

Em pacientes com acometimento significativo do tronco da coronária esquerda, há poucos estudos modernos avaliando o impacto do tratamento invasivo comparativamente à conduta conservadora. De forma recorrente, os estudos contemporâneos têm centrado o foco de interesse na comparação entre os tratamentos percutâneo e cirúrgico, sem avaliar o impacto da intervenção vs. um braço de tratamento clínico. Uma metanálise recente com 4.394 pacientes incluídos em quatro estudos que compararam angioplastia com stents farmacológicos vs. cirurgia revelou que as taxas de mortalidade após 5 anos eram semelhantes entre as duas modalidades terapêuticas, tanto para pacientes agudos (HR 0,93; IC 95% 0,68–1,27) quanto para crônicos (HR 1,19; IC 95% 0,95–1,50).^402^ Nesse mesmo estudo, pacientes tratados de maneira percutânea apresentaram menor risco de AVC, mas tiveram maior taxa de infarto espontâneo.

O estudo randomizado FAME-2 demonstrou que a avaliação invasiva da fisiologia coronariana, através da FFR, é capaz de diferenciar pacientes que se beneficiam do tratamento percutâneo. Entre aqueles que apresentam lesões com obstrução estenose ≥ 50% à angiografia e RFF ≤ 0,8, a angioplastia coronariana se associou a menor risco de revascularização urgente ou infarto espontâneo ao longo de 5 anos de evolução.^145^ Também, mais recentemente, a análise das imagens da angiografia invasiva através de métodos computacionais de dinâmica de fluxo para guiar o tratamento percutâneo tem se associado à melhora significativa do prognóstico pós-procedimento.^403,404^

No momento atual, a intervenção percutânea em pacientes com SCC é fundamentalmente indicada como estratégia de revascularização para territórios acometidos por obstrução luminal significativa e com repercussão hemodinâmica, ressaltando que a redução de eventos cardiovasculares só ocorre em subgrupos específicos de pacientes, não devendo ser recomendada de forma rotineira para tal objetivo.

#### 6.4.1. Intervenção Coronariana Percutânea como Estratégia para Alívio de Sintomas

Nas SCCs, são estabelecidos dois pilares fundamentais do tratamento: as terapias para redução de eventos cardiovasculares e as terapias para alívio de sintomas. A revascularização, quando indicada em pacientes selecionados, é capaz de reduzir o risco de eventos cardiovasculares, incluindo morte, infarto e necessidade de revascularização, especificamente em pacientes com doença multiarterial, disfunção ventricular esquerda e doença de tronco da coronária esquerda. Da mesma forma, em pacientes que permanecem com sintomas limitantes e com prejuízo da qualidade de vida a despeito do tratamento medicamentoso tolerado, a revascularização deve ser considerada com o objetivo de aprimorar o controle de angina e a qualidade de vida. A extensão e severidade da isquemia induzida por estresse, avaliada por métodos de imagem, pode ser útil para orientar a tomada de decisão clínica.

Entretanto, na prática clínica, a associação entre angina e isquemia miocárdica pode ser difícil de ser estabelecida em pacientes com angina estável. No estudo ORBITA-2 (*Objective Randomized Blinded Investigation with Optimal Medical Therapy of Angioplasty in Stable Angina-2*), embora a revascularização percutânea tenha reduzido os sintomas, em aproximadamente 60% dos pacientes os sintomas persistiam, apesar da eliminação de isquemia miocárdica.^404^ Faz-se necessária, portanto, a ponderação de se, de fato, a queixa clínica do paciente corresponde a uma dor anginosa.

Numa análise secundária do ORBITA-2, não foi observada relação entre a gravidade dos sintomas com a extensão da isquemia miocárdica por ecocardiografia de estresse, ou avaliação invasiva por reserva de fluxo fracionada ou com a severidade da estenose por angiografia invasiva.^405^ Nesse estudo, o maior preditor de redução na angina após a revascularização percutânea foi a presença de angina típica em pacientes com terapia médica otimizada.

Da mesma forma, no estudo ISCHEMIA, pacientes mais jovens ou com angina mais severa tiveram maior probabilidade de se beneficiar da intervenção percutânea para alívio dos sintomas.^406^

O estudo ORBITA-STAR (*Symptomatic Trial of Angina Assessment Prior to Revascularization*)^407^ avaliou se a isquemia induzida pela insuflação do balão de angioplastia em estenoses coronárias determinava sintoma similar à angina basal dos pacientes. Os autores demonstraram que, quando a obstrução coronariana pelo balão desencadeava angina equivalente, havia maior probabilidade de resolução da angina avaliada 12 semanas após a revascularização percutânea, quando comparada a pacientes em que os sintomas não eram reproduzidos pela insuflação do balão. Destaca-se aqui, fundamentalmente, a necessidade de se obter uma correlação acurada entre sintomatologia e documentação de lesão anatômica e/ou isquemia, a fim de propor um tratamento eficaz (Tabela 27).

**Tabela 27:** Indicações de tratamento percutâneo para controle de sintomas e melhora prognóstica

Recomendações	Grau de recomendação	Nível de evidência
Controle de sintomas em pacientes com estenoses coronarianas significativas e angina típica limitante sob terapia otimizada.	**I**	**A**
Doença coronariana multiarterial e morfologia apropriada para tratamento percutâneo (ex.: complexidade anatômica menor ou moderada).	**IIa**	**A**
Doença coronariana com acometimento significativo do tronco da coronária esquerda e morfologia apropriada para tratamento percutâneo (ex.: complexidade anatômica menor ou moderada).	**IIa**	**B**
Estenose luminal determinando acometimento significativo do fluxo coronariano aferido por método invasivo.	**I**	**B**
Estenose luminal não significativa com características de vulnerabilidade por métodos de imagem intravascular.	**IIb**	**B**

### 6.5. Escores de Risco de Sangramento na Tomada de Decisão

Sangramento afeta significativamente o prognóstico de pacientes com DAC, com uma associação clara entre eventos hemorrágicos e aumento da mortalidade e morbidade.^408–411^

Sangramentos maiores, especialmente aqueles que requerem transfusão de sangue ou hospitalização prolongada, podem precipitar descompensações hemodinâmicas e cardíacas, além de interromper ou modificar terapias antitrombóticas essenciais para a prevenção de eventos isquêmicos. Além disso, a ocorrência de sangramento pode ser indicativa de uma fragilidade subjacente maior, refletindo uma complexa interação entre fatores clínicos, como idade avançada, comorbidades e a necessidade de tratamentos invasivos.^408,412^

Em suma, o manejo adequado do risco de sangramento, através de uma avaliação minuciosa e da implementação de estratégias preventivas eficazes, é crucial para melhorar os desfechos clínicos em pacientes com DAC.

Os critérios do ARC-HBR (*Academic Research Consortium for High Bleeding Risk*) foram desenvolvidos para identificar pacientes com risco elevado de sangramento, fornecendo uma definição consensual e prática para uso em ensaios clínicos e prática clínica.^408^ Esses critérios incluem uma série de fatores clínicos e demográficos que aumentam a predisposição ao sangramento (Tabela 28). Um critério maior para o ARC-HBR é definido como qualquer critério que, isoladamente, seja considerado para conferir um risco de sangramento maior ≥ 4% em 1 ano, ou qualquer critério considerado associado a um risco de hemorragia intracraniana (HIC) de ≥ 1% em 1 ano. Um critério menor para o ARC-HBR é definido como qualquer critério que, isoladamente, seja considerado para conferir um risco aumentado de sangramento maior ≤ 4%. No entanto, a avaliação do risco de sangramento com base nos critérios do ARC-HBR pode ser difícil de aplicar na prática clínica diária, tendo em vista a complexidade dos critérios, contribuindo para dificultar uma estratificação de risco mais individualizada, especialmente para a combinação de fatores clínicos.

**Tabela 28:** Critérios maiores e menores para sangramento de alto risco no momento da intervenção coronária percutânea

Critérios maiores	Critérios menores
	Idade ≥ 75 anos
Uso crônico de anticoagulantes orais	Uso prolongado de AINEs ou esteroides
Doença renal crônica (clearance de creatinina < 30 mL/min)	Doença renal crônica (clearance de creatinina 30 a 59 mL/min)
Anemia (hemoglobina < 11 g/dL)	Anemia (hemoglobina 11 a 12,9 g/dL para homens e 11 a 11,9 g/dL para mulheres)
Hemorragia necessitando hospitalização ou transfusão nos últimos 6 meses ou em qualquer momento, se recorrente	Hemorragia necessitando hospitalização ou transfusão nos últimos 12 meses
Plaquetas < 100.000/*μ*L	
Diátese hemorrágica	
Doença hepática avançada (cirrose ou hipertensão portal)	
Malignidade ativa (excluindo câncer de pele não melanoma) nos últimos 12 meses	
Hemorragia intracerebral espontânea anterior (em qualquer momento) Hemorragia intracerebral traumática nos últimos 12 meses Presença de uma MAVc Acidente vascular cerebral isquêmico moderado ou grave nos últimos 6 meses	Qualquer acidente vascular cerebral isquêmico prévio que não tenha critério maior
Grande cirurgia não adiável Grande cirurgia ou trauma nos últimos 30 dias	

AINEs: anti-inflamatórios não esteroides; MAVc: malformação arteriovenosa cerebral.

Os escores PRECISE-DAPT (*Predicting Bleeding Complications in Patients Undergoing Stent Implantation and Subsequent Dual Antiplatelet Therapy*), ARC-HBR *Trade Off* e DAPT^412–414^ foram desenvolvidos para orientar e informar a tomada de decisões sobre a duração da DAPT em pacientes submetidos a implante de stent.

A utilidade do escore PRECISE-DAPT foi avaliada retrospectivamente em pacientes randomizados para diferentes durações de DAPT (n = 10.081) para identificar o efeito sobre sangramento e isquemia de um tratamento longo (12 a 24 meses) ou curto (3 a 6 meses) em relação ao risco de sangramento basal.

Entre os pacientes com alto risco de sangramento (*high bleeding risk* – HBR) de acordo com o escore PRECISE-DAPT (≥ 25), a DAPT prolongada não demonstrou benefício isquêmico e aumentou o risco de eventos hemorrágicos. Em contraste, o tratamento prolongado em pacientes sem HBR (escore PRECISE-DAPT < 25) não foi associado a um aumento no sangramento, mas foi associado a uma redução significativa no desfecho isquêmico composto de IM, trombose definitiva de stent, AVC e revascularização da lesão-alvo. Uma validação externa do escore PRECISE-DAPT em 4.424 pacientes com SCA submetidos a ICP e tratados com prasugrel ou ticagrelor mostrou um valor preditivo modesto para sangramento maior em um seguimento mediano de 14 meses (*c-statistic* = 0,65). O escore PRECISE-DAPT é aplicado na alta do paciente.

O modelo de *trade-off* ARC-HBR é uma ferramenta desenvolvida para avaliar os riscos de sangramento vs. eventos trombóticos em pacientes com alto risco de sangramento após a implantação de stents coronarianos. Esse modelo considera características individuais dos pacientes para prever o risco de sangramento maior não relacionado ao procedimento e eventos trombóticos (IM e/ou trombose de stent) após a intervenção coronariana. A precisão do modelo foi avaliada em diferentes populações e demonstrou boa discriminação para eventos trombóticos em um período de um ano. No entanto, em relação ao sangramento, o modelo tende a superestimar o risco de sangramento maior, e sua capacidade de discriminação foi menos satisfatória em alguns grupos de pacientes.^415^

O escore DAPT (7) foi desenvolvido para estimar o risco de sangramento e orientar a duração da terapia antiplaquetária dupla em pacientes que passaram por ICP. Pacientes tratados por um ano com DAPT sem eventos significativos se beneficiam de DAPT prolongada se tiverem um escore DAPT alto (≥ 2), pois reduz eventos isquêmicos e hemorrágicos combinados. Em contrapartida, para aqueles com escore baixo (< 2), prolongar a DAPT aumenta o risco de sangramento sem reduzir eventos isquêmicos. Uma limitação do escore DAPT é que ele foi desenvolvido para predição de risco para 1 ano de terapia antiplaquetária dupla, e a orientação atual é de terapia antiplaquetária dupla mais curta para pacientes com alto risco de sangramento.

#### 6.5.1. Recomendação

Os antiplaquetários desempenham um papel crucial após ICP. A escolha da estratégia antiplaquetária e de sua duração deve considerar o risco de sangramento do paciente. Sugere-se a aplicação dos escores de risco de sangramento para uma tomada de decisão individualizada, lembrando das limitações, como avaliação de fragilidade ou queda, principalmente em idosos (Tabela 29).

**Tabela 29:** Recomendação de utilização do escore de risco de sangramento na tomada de decisão

Recomendação	Grau de recomendação	Nível de evidência
Usar os critérios ARC-HBR para identificação de pacientes com alto risco de sangramento para uso de DAPT encurtada.	**IIa**	**B**
PRECISE-DAPT ≥ 25 para identificação de pacientes com alto risco de sangramento para uso de DAPT encurtada.	**IIa**	**B**
Usar modelo de trade-off ARC-HBR para predição de risco de infarto, trombose de stent e sangramento maior.	**IIb**	**C**
Usar o escore DAPT para decisão do tempo de DAPT.	**III**	**C**

ARC-HBR: Academic Research Consortium for High Bleeding Risk; DAPT: dupla terapia antiplaquetária (*dual antiplatelet therapy*); PRECISE-DAPT: Predicting Bleeding Complications in Patients Undergoing Stent Implantation and Subsequent Dual Antiplatelet Therapy.

Fatores associados a um risco elevado de sangramento podem ser determinados pelos critérios do ARC-HBR ou um escore PRECISE-DAPT ≥ 25. O uso dessas duas ferramentas já mostrou ser válido para decidir sobre um regime antiplaquetário duplo encurtado (1 mês) ou padrão (6 meses) em estudo randomizado.^8^

O escore DAPT foi desenvolvido para uma estratégia padrão de dupla antiagregação plaquetária (DAPT) de 1 ano, sem representação para as atuais gerações de stents farmacológicos. O modelo de *trade-off* ARC-HBR possui utilidade para auxiliar na decisão do tempo de terapia antiplaquetária, mas com menor embasamento clínico.

### 6.6. Preparo em Situações Específicas (Pacientes com Disfunção Renal ou Alérgicos ao Contraste)

#### 6.6.1. Disfunção Renal

A coronariografia e a ICP utilizando meios de contraste (MCs) têm sido amplamente utilizadas na prática clínica para o diagnóstico e tratamento da DAC. Apesar das melhorias da estrutura química, os MCs ainda apresentam efeitos deletérios podendo causar nefropatia induzida pelo contraste (NIC).^416^

As causas mais aceitas para o desenvolvimento da NIC são: redução do fluxo renal, resultando em isquemia medular, produção de radicais livres e efeito tóxico direto nas células tubulares.^417^ A definição de NIC inclui um aumento absoluto > 0,5 mg/dL/44 *μ*mol/L ou relativo > 25% dos níveis basais de creatinina sérica, 48 a 72 horas após a exposição aos MCs.^418^

A incidência de NIC na população geral mostra-se < 3%, entretanto, pode atingir 50% dos pacientes com múltiplos fatores de risco, tais como: doença renal avançada, diabetes mellitus, hipertensão arterial sistêmica e IC congestiva.^419^

A NIC é a terceira causa mais comum de insuficiência renal adquirida na fase hospitalar,^420^ aumentando, a curto e longo prazo, o risco de eventos adversos, incluindo terapia de substituição renal, IAM, AVC e mortalidade.

#### 6.6.2. Medidas de Proteção Renal

Como a ocorrência de NIC pode ser previsível na maioria dos casos, medidas preventivas representam a única maneira de tratamento desses pacientes, que têm como objetivo reduzir a injúria renal e os efeitos adversos associados a essa ocorrência.

#### 6.6.3. Medidas Gerais

A história clínica e a avaliação da função renal podem ser utilizadas para calcular o risco de desenvolvimento de NIC. A creatinina sérica tem sido reconhecida atualmente como um marcador pouco sensível da função renal, visto que pacientes com comprometimento moderado podem exibir níveis normais. Por esse motivo, a taxa de filtração glomerular deve ser calculada, e algumas equações foram criadas para uso na prática clínica.^421^ Uma taxa de filtração glomerular estimada (TFGe) < 60 mL/min deve ser considerada como uma condição de "alto risco" para o desenvolvimento de NIC.^422^

Quando a relação entre o volume total de contraste injetado (mL) e a taxa de filtração glomerular (mL/min) excede 3,7, o risco de NIC aumenta significativamente.^422^

Informações baseadas em múltiplos estudos possibilitaram a elaboração de um escore preditor de risco ao desenvolvimento de NIC em pacientes submetidos a ICP (Tabelas 30 e 31).

**Tabela 30:** Escore de risco para o desenvolvimento de NIC

Fatores de risco	Escore (pontos)
Hipotensão	5
Balão intra-aórtico	5
ICC (NYHA III/IV)	5
Idade > 75 anos	4
Anemia	3
Diabetes	3
Volume de contraste	1 para cada 100 mL
Creatinina plasmática > 1,5 mg/dL ou	4
TFG < 60 mL/min/1,73 m^2^	2 para 40–60 4 para 20–40 6 para < 20

ICC: insuficiência cardíaca congestiva; NIC: nefropatia induzida pelo contraste; NYHA: New York Heart Association; TFG: taxa de filtração glomerular.

**Tabela 31:** Risco de desenvolvimento de NIC e diálise

Escore (pontuação)	Risco de NIC	Risco de diálise
= 5	7,5%	0,04%
6 a 10	14%	0,12%
11 a 16	26,1%	1,09%
≥ 16	57,3%	12,6%

NIC: nefropatia induzida pelo contraste.

#### 6.6.4. Administração de Fluídos

Até o momento, as únicas estratégias que demonstraram diminuição do risco de NIC são a hidratação e a redução da quantidade de contraste utilizada. Outras medidas mostraram resultados neutros, ou deletérios, ou foram caracterizadas por dados heterogêneos e conflitantes.

Estudos com a hidratação sugerem que a solução salina isotônica (0,9%) é preferível quando comparada à solução isotônica hipotônica (0,45%), bem como a via endovenosa em comparação com a via oral. A hidratação por horas, antes e após a exposição aos MCs, é recomendada em vez da infusão em "*bolus*", e a administração da solução salina é preferível à solução salina associada ao manitol ou furosemida.^423^

De acordo com esses achados, um regime de hidratação em procedimentos eletivos seria solução salina isotônica 0,9% (1,0 a 1,5 mL/kg/h) por 3 a 12 horas antes e mantido por 6 a 24 horas após o término do procedimento.

A utilização do bicarbonato de com o intuito de alcalinizar o fluído renal e diminuir a geração de espécies reativas de oxigênio poderia minimizar o dano tubular. Entretanto, sua utilização mostra resultados divergentes.^424^

#### 6.6.5. Prevenção Farmacológica

Vários agentes farmacológicos foram testados com o objetivo de reduzir o risco de NIC em pacientes submetidos a administração de MCs.

Estudos iniciais com a utilização da N-acetil-L-cisteína demonstraram resultados conflitantes. Uma recente revisão sistemática evidenciou que sua utilização não previne o desenvolvimento da NIC.^425^ Com base nesses dados, não há evidencias que suportem a utilização da N-acetil-L-cisteína na prevenção da NIC.

Outros medicamentos, como BCC, prostaglandinas, antagonistas da adenosina, peptídeo natriurético atrial e iECA,^2^ foram testados e não demonstraram benefício nem apresentaram resultados conflitantes com sua utilização.^426^

#### 6.6.6. Meios de Contraste

Os MCs iodados recomendados são os contrastes hiposmolares e os isosmolares, que possuem uma osmolaridade mais baixa e semelhante ao do plasma sanguíneo, respectivamente. Ambos apresentam menos incidência de efeitos colaterais como: menor incidência de bradicardia, hipotensão arterial sistêmica, e sensação de calor, náuseas, vômitos, reações alérgicas e efeito nefrotóxico.

Recentemente, a técnica de volume de contraste administrado ultrabaixo, a qual é definida como uma intervenção, em que a relação de contraste administrada e a taxa de filtração glomerular estimada é menor que 1, tem sido aplicada em alguns contextos clínicos como: DRC, injúria renal aguda prévia, transplante renal, disfunção ventricular esquerda severa e intervenções coronárias percutâneas complexas para se evitar, não somente a ocorrência de NIC, como também de instabilidade hemodinâmica.^427^

#### 6.6.7. Alergia aos Meios de Contraste

A incidência de reações alérgicas aos MCs ocorre em ≤ 1% dos procedimentos, e as reações graves em 0,04%. Essas reações não são verdadeiramente alérgicas, pois não são mediadas por imunoglobulina (Ig) E. A melhor caracterização é que elas são anafilactoides, envolvendo degranulação de mastócitos e basófilos circulantes através da ativação direta do complemento. Mais detalhes podem ser consultados em diretriz específica.^428^

Pacientes que já apresentaram reação anafilactoide prévia aos MCs são considerados de alto risco para episódios recorrentes. Reações anafilactoides repetidas têm sido reportadas em 16 a 44% dessa população.

A utilização de prednisona 20 mg associada a um anti-histamínico de segunda geração (cloridrato de fexofenadina 180 mg) via oral (VO) 3 dias antes do procedimento é recomendada. Os pacientes submetidos a esse tratamento apresentam taxas de eventos graves próxima a zero.

Nos procedimentos de emergência, a administração de hidrocortisona 500 mg intravenosa (IV), cloridrato de fexofenadina 180 mg VO e cloridrato de difenidramina 50 mg IV ou intramuscular (IM) (bloqueador do receptor H1) deve ser empregada (Tabelas 32 e 33).^429^

**Tabela 32:** Profilaxia de reações alérgicas: recomendações

Recomendação	Grau de recomendação	Nível de evidência
Utilização de contraste isosmolar ou hiposmolar.	**I**	**A**
Prednisona 50 mg. 13 h, 7 h, 1 h antes do procedimento.	**I**	**B**
Cloridrato de difenidramina 50 mg IV ou IM 1 h antes do procedimento.	**I**	**B**
Cloridrato de fexofenadina 180 mg. 1 cp/dia durante 3 dias antes do procedimento.	**I**	**C**

cp: comprimido; IM: intramuscular; IV: intravenoso.

**Tabela 33:** Profilaxia de reação alérgicas para procedimentos de emergência

Medicação	Grau de recomendação	Nível de evidência
Hidrocortisona 500 mg IV	**I**	**B**
Cloridrato de difenidramina 50 mg IV ou IM 1 h antes do procedimento	**I**	**B**
Cloridrato de fexofenadina 180 mg VO 1 h antes do procedimento	**I**	**C**

IM: intramuscular; IV: intravenoso; VO: via oral.

#### 6.6.8. Conclusões

A evolução dos MCs impactou diretamente a redução das complicações relacionadas aos procedimentos angiográficos.

O conhecimento de prevenção, diagnóstico e tratamento da NIC e das reações alérgicas é essencial aos cardiologistas clínicos, intervencionistas e profissionais de imagem.

A aplicação das recomendações descritas é de fundamental importância para a redução e controle das complicações relacionadas aos MCs.

### 6.7. Tratamento Cirúrgico para Controle de Sintomas e Redução do Risco de Eventos

A DAC apresenta considerável morbimortalidade devido a sua característica de induzir o aparecimento do IM, aumentando o risco de morte e de IC. Além disso, o processo inflamatório local e sistêmico e a disfunção endotelial inerentes à aterosclerose resultam no desenvolvimento da *angina* *pectoris*, comprometendo a qualidade de vida e a capacidade de esforço dos pacientes. Portanto, as terapias destinadas ao tratamento da DAC devem ter esses alvos e focar a redução desses desfechos.

A seleção do procedimento da CRM deve levar em consideração fatores importantes, como a extensão e complexidade anatômica da DAC, os resultados esperados com a terapêutica considerada, o risco associado a complicações e morte, entre outros desfechos adversos.

Inúmeras evidências históricas e contemporâneas confirmam que a CRM é o procedimento mais eficaz para o tratamento da DAC aterosclerótica avançada, reduzindo o risco de IM, aumento da sobrevida e melhora da angina e qualidade de vida nesse grupo de pacientes. Essas evidências incluem estudos randomizados controlados, metanálises e registros nacionais e regionais.

O principal objetivo para melhorar o prognóstico na DAC está relacionado a uma terapia capaz de proteger contra futuros eventos trombóticos coronarianos agudos. Evidências recentes mostram que o evento coronariano agudo não ocorre primariamente devido à oclusão da placa no local da estenose grave, como observado na cinecoronariografia convencional, mas, sim, devido à ruptura ou erosão de uma placa aterosclerótica coronária, mais frequentemente com estenose leve a moderada (a placa instável ou vulnerável), comumente localizada distante da placa estável. Portanto, o principal mecanismo de morte na DAC não está mais atribuído à placa estável (a estenose significativa acometendo > 50% do lúmen do vaso), mas, sim, ao IAM, causado pela ruptura de uma placa instável com trombose intracoronariana secundária. A eficácia da CRM em reduzir eventos decorre da capacidade de tratar a lesão obstrutiva existente e as futuras lesões trombóticas e oclusivas, anastomosando um enxerto (arterial ou venoso) distalmente no leito coronariano e protegendo contra eventos isquêmicos agudos, enquanto a ICP aborda apenas a lesão estável e deixa em risco as futuras lesões (as placas instáveis).^430–435^ Vários fatores influenciam e devem ser ponderados para a seleção de pacientes nos quais o procedimento da CRM leva a resultados de redução do risco de IM, com aumento de sobrevida e melhora da angina (Tabela 34).

**Tabela 34:** Subgrupos de pacientes com Indicação para CRM

Anatomia – Extensão da DAC
Doença de múltiplos vasos
Escore SYNTAX > 23
Envolvimento proximal da artéria descendente anterior
Lesão de tronco da artéria coronária esquerda
**Especialmente com morbidades associadas**
Diabetes
Disfunção ventricular esquerda
Infarto agudo do miocárdio sem supra do segmento ST

CRM: cirurgia de revascularização do miocárdio; DAC: doença arterial coronariana; SYNTAX: *Synergy Between PCI with TAXUS™ and Cardiac Surgery*.

#### 6.7.1. Pacientes com Doença de Múltiplos Vasos Coronarianos e Escore SYNTAX > 23

Pacientes com DAC avançada com doença de múltiplos vasos coronarianos e escore SYNTAX > 23 têm benefícios de redução de eventos conforme demonstrado em vários estudos randomizados controlados.

Na análise de 5 anos do estudo SYNTAX, que incluiu, majoritariamente, pacientes com FEVE preservada com lesões de múltiplos vasos, a CRM foi associada à redução significativa na morte cardiovascular e cardíaca, principalmente por diminuição da morte relacionada ao IM em comparação com a ICP. Em contraste, o tratamento com ICP foi um preditor independente de morte cardíaca, com mortalidade 40% maior em pacientes com doença de 3 vasos (3V) com ICP do que com CRM. A diferença na morte relacionada ao IM foi observada principalmente em pacientes com diabetes, lesão de 3V ou com escores SYNTAX mais elevados (> 23).^436^ Em 10 anos de acompanhamento, o SYNTAX *Extended Survival* (SYNTAXES) reportou que a mortalidade por todas as causas foi significativamente menor com CRM do que com ICP, com o expressivo benefício de sobrevida sendo observado em pacientes com 3V, e com as curvas de sobrevida divergindo continuamente ao longo do tempo favorecendo a CRM (Figura 17).^141^

O estudo MASS-II, o único estudo a comparar CRM, ICP e tratamento médico otimizado (TMO) em pacientes com DAC multiarterial, com o desfecho composto de mortalidade total, IM com onda Q ou AR exigindo revascularização, demonstrou que, em 5 anos de acompanhamento, a CRM foi superior ao TMO em termos de desfechos primários, atingindo uma redução significativa de 44% nos desfechos primários. Não houve diferença estatística entre ICP e TMO na incidência do desfecho composto estudado.^437^

No estudo MASS-II, todos os pacientes foram colocados em um tratamento médico ideal desde o início do estudo até o final do acompanhamento, consistindo em aspirina, β-bloqueadores, inibidores da ECA, BCC, nitratos e agentes hipolipemiantes, juntamente com uma dieta com baixo teor de gordura. Todos os medicamentos foram dispensados gratuitamente aos pacientes ao longo dos 10 anos de acompanhamento para garantir a adesão ao protocolo. Embora o desenho do estudo não tivesse poder suficiente para avaliar os componentes individuais do desfecho composto, uma incidência significativamente menor de IM não fatal com CRM vs. TMO foi observada em 10 anos (20,7 vs. 10,3, p = 0,010), mas não em 5 anos (p = 0,785). A morte cardíaca foi significativamente maior com TMO vs. CRM (20,7% vs. 10,8%, p = 0,019), mas, aos 5 anos, não foi significativa em 12,3% vs. 7,9% (p = 0,631). A mortalidade por todas as causas foi reduzida com CRM vs. TMO (25,1% vs. 31,0%, p = 0,089), embora não tenha atingido significância estatística, em 5 anos, foi de 12,8% vs. 16,2% (p = 0,824). A análise de comparação pareada mostrou um aumento significativo de 2,02 e 2,77 vezes no risco de morte cardíaca e IM subsequente com TMO vs. CRM, respectivamente, demonstrando o prognóstico progressivamente melhor em longo prazo dos pacientes cirúrgicos.^398^

Os resultados do estudo MASS-II reforçam os achados dos estudos SYNTAX, e também STICH (*Surgical Treatment for Ischemic Heart Failure*) e FREEDOM (*Future Revascularization Evaluation in Patients with Diabetes Mellitus: Optimal Management of Multivessel Disease*), em que os benefícios adicionais e robustos da CRM aumentaram progressivamente no acompanhamento de longo prazo, além do escrutínio inicial de 5 anos.

Mais recentemente, a publicação do estudo FAME-3, que randomizou pacientes com lesão de 3V com função de VE predominantemente preservada (> 80% dos pacientes tinham fração de ejeção > 50%) para CRM ou ICP guiada por FFR com uso de stents farmacológicos de segunda geração com liberação de zotarolimus. Na análise de 1 ano do desfecho primário composto (morte, infarto, AVC e revascularização repetida), a tecnologia de ICP mais avançada não foi eficaz na redução de eventos cardiovasculares e cerebrovasculares adversos maiores em comparação com a CRM, reforçando as vantagens da CRM em pacientes com 3VD. A incidência de infarto espontâneo do miocárdio (3,3% vs. 2,3%), morte por qualquer causa (1,6% vs. 0,9%) e morte cardiovascular (0,8% vs. 0,5%) foi maior com ICP vs. CRM, respectivamente. Os achados são notáveis, pois o benefício da CRM foi evidente no estágio inicial do estudo, com apenas 1 ano de acompanhamento, em contraste com estudos anteriores, nos quais os resultados favoráveis da CRM foram observados apenas após um acompanhamento de > 3 anos.^438^

O acompanhamento de 3 anos do estudo FAME-3 mostrou uma divergência cada vez maior entre os resultados da ICP e da CRM em relação ao desfecho composto primário, 18,6% vs. 12,5%, respectivamente (p = 0,002), sendo a que diferença entre a incidência de IM tornou-se estatisticamente significativa em favor da CRM (7,0% vs. 4,2%; p = 0,02). Da mesma forma, a diferença no desfecho secundário de morte, IM ou AVC em 3 anos com 9,2% no grupo de CRM e 12,0% no grupo de ICP, ampliando a diferença obtida em 1 ano com 5,2% vs. 7,3%, respectivamente. A publicação focou nos resultados do desfecho secundário, entretanto, não possui poder estatístico para recomendações.^439^

A análise planejada de 5 anos, avaliando desfecho primário composto por morte, AVC ou IM, não evidenciou diferença entre os grupos ICP e CRM. Observando os componentes de forma isolada, as taxas de IM e revascularização repetida foram maiores no grupo ICP.^440^

#### 6.7.2. Pacientes com DAC Avançada E Diabetes Mellitus

Múltiplas evidências têm demonstrado que a CRM oferece resultados superiores a terapias alternativas, reduzindo as taxas de morte e IM em pacientes com DAC avançada e diabetes. O estudo BARI 2D (*Bypass Angioplasty Revascularization Investigation 2 Diabetes*) incluiu pacientes com diabetes tipo 2 para revascularização coronária imediata (REV) mais terapia médica intensiva vs. terapia médica intensiva (MED) isolada e não encontrou nenhuma diferença significativa na mortalidade entre os grupos de pacientes. Entretanto, no estrato CRM, a taxa de eventos cardiovasculares maiores foi significativamente menor no grupo de revascularização (22,4%) do que no grupo de terapia medicamentosa (30,5%, p = 0,01). No estrato ICP, não houve diferença significativa nos desfechos primários entre o grupo de revascularização e o grupo de terapia médica. O risco de morte/IM/AVC em 5 anos foi de 36,8% para MED em comparação com 24,8% para REV entre os 381 pacientes selecionados para CRM no tercil de risco angiográfico mais alto (p = 0,005); esse efeito do tratamento foi amplificado em pacientes com alto risco angiográfico e alto de Framingham (47,3% MED vs. 27,1% REV, p = 0,010).^441^

O estudo FREEDOM randomizou pacientes com diabetes e DAC multiarterial para ICP com stent farmacológico ou CRM, com acompanhamento médio de 3,8 anos. O desfecho primário foi um composto de morte por qualquer causa, IM não fatal ou AVC, e a CRM foi considerada superior à ICP com redução significativa das taxas de morte e IM, com maior taxa de AVC. As taxas de IM espontâneo devido a novas lesões foram significativamente maiores no grupo ICP comparado com a CRM.^442^ O estudo FREEDOM *Follow-On* acompanhou os pacientes por 7,5 anos, e a taxa de mortalidade por todas as causas foi significativamente maior no grupo ICP do que no grupo CRM (24,3% vs. 18,3%; p = 0,01) (Figura 17).^443^

A análise dos três estudos financiados pelo governo americano (BARI 2D,^441^ COURAGE [*Clinical Outcomes Utilizing Revascularization and Aggressive Drug Evaluation*]^85^ e FREEDOM^44^) teve como objetivo determinar o efeito do TMO, com ou sem ICP ou CRM, em resultados de longo prazo em pacientes com DAC e diabetes tipo 2. Durante um acompanhamento médio de 4,5 anos, CRM + TMO foi superior a ICP + TMO para o desfecho primário (composto de morte, IM ou AVC), assim como morte e IM. A CRM + TMO também foi superior ao TMO isolado para a prevenção do desfecho primário e IM, e foi superior ao grupo ICP + TMO para o desfecho primário em pacientes com 3VD e FEVE normal. Não houve diferenças significativas ao comparar TMO vs. ICP + TMO.^444^

Uma metanálise incluindo dados de 28.846 pacientes demonstrou resultados consistentes, com aumento do risco de mortalidade em 5 anos entre os pacientes tratados com ICP do que entre aqueles tratados com CRM. A revascularização de pacientes com diabetes empregando a CRM está associada a uma redução significativa da mortalidade em longo prazo, IM, eventos cardíacos e cerebrovasculares adversos maiores (*major adverse cardiac and cerebrovascular events* [MACCE]) e revascularizações repetidas.^445^

#### 6.7.3. Pacientes com Disfunção Ventricular Esquerda

A eficácia do tratamento da miocardiopatia isquêmica em pacientes com disfunção ventricular esquerda foi investigada no estudo STICH, que comparou a adição da revascularização cirúrgica do miocárdio ao TMO comparado a TMO isolado. No seguimento de 5 anos, houve um benefício significativo observado nos desfechos secundários no grupo cirúrgico, com redução de morte cardiovascular e do desfecho composto de mortalidade por todas as causas ou hospitalização. Nessa análise, a terapia cirúrgica não reduziu estatisticamente o desfecho primário de mortalidade por todas as causas.^446^

Com a extensão da análise do estudo STICH, aos 10 anos de seguimento, o grupo de pacientes submetidos a CRM (associada ao TMO) teve menor taxa de morte por qualquer causa, morte por causas cardiovasculares e morte por qualquer causa ou hospitalização por causas cardiovasculares quando comparado com aqueles que receberam TMO apenas. A CRM reduziu significativamente as mortes súbitas e as causadas por IM, com um efeito nominalmente significativo nos eventos fatais por falha de bomba. O efeito favorável na morte súbita pela CRM também pode estar relacionado a um impacto benéfico na progressão da IC, como indicado por uma redução nos eventos fatais de falha de bomba. A Figura 1 mostra o seguimento de 10 anos do estudo STICH, com as curvas de sobrevida se divergindo com o tempo a favor dos pacientes submetidos a CRM.^136^

O desenvolvimento de IC comumente ocorre por um IM e está relacionado com a extensão do IM, o território envolvido, o desenvolvimento de insuficiência mitral e a presença de certas taquiarritmias. O estado de inflamação sistêmica elevada que ocorre na IC propicia maior desestabilização das placas ateromatosas, com maior risco de eventos coronarianos agudos. No paciente com IC e redução da FEVE, mesmo infartos de pequena extensão conferem maior risco de mortalidade.

#### 6.7.4. Pacientes com Lesão de Tronco de Artéria Coronária Esquerda

O estudo SYNTAX foi crucial e decisivo para avaliar a eficácia das terapêuticas de CRM e ICP na lesão de tronco de artéria coronária esquerda (LTACE). As evidências derivadas do estudo mostraram benefícios definitivos, com significância estatística, em favor do tratamento cirúrgico em pacientes com LTACE de alto risco (escore SYNTAX > 32), conferindo recomendação classe I para cirurgia e classe III para ICP na diretriz europeia de revascularização miocárdica. No entanto, em pacientes com escore SYNTAX < 32 (risco baixo e intermediário), não houve diferença estatística, mas o estudo não teve poder estatístico para resolver definitivamente esse aspecto.^447^ Consequentemente, foram necessários estudos adicionais para resolver essa questão, e os ensaios EXCEL (*Evaluation of XIENCE Everolimus Eluting Stent Versus Coronary Artery Bypass Surgery for Effectiveness of Left Main Revascularization*) e NOBLE (*Nordic–Baltic–British Left Main Revascularization*) foram concebidos para esse fim. Ambos foram ensaios randomizados de não inferioridade, desenhados para avaliar comparativamente a revascularização cirúrgica e percutânea em pacientes com LTACE e escore SYNTAX < 32.

O estudo NOBLE envolveu 1.200 pacientes com LTACE, e o desfecho primário foi um composto dos principais eventos adversos cardíacos e cerebrovasculares (MACCE; morte por qualquer causa, IM, nova revascularização e AVC). O acompanhamento de 5 anos concluiu que as taxas de MACCE foram de 28% para ICP e de 19% para CRM, excedendo o limite de não inferioridade, e que a CRM foi significativamente melhor que ICP nesses pacientes. A mortalidade por todas as causas foi semelhante nos dois procedimentos, 9% com ICP vs. 9% após CRM.^448^

O estudo EXCEL mostrou que a mortalidade por todas as causas foi estatisticamente significante, favorecendo a CRM aos 5 anos (9,9% com CRM vs. 13,0% com ICP) e continua se acelerando em favor da cirurgia, pois as curvas de sobrevida continuam se divergindo ao longo do tempo. A mortalidade em 5 anos foi 38% maior no grupo da ICP do que no da CRM. No entanto, o estudo concluiu que não foi encontrada diferença significativa entre ICP e CRM em relação aos resultados do desfecho composto de morte, AVC ou IM aos 5 anos.^449,450^

Uma metanálise reunindo dados de quatro estudos de ICP vs. CRM em pacientes com LTACE mostrou que a mortalidade em 5 anos foi semelhante para ICP e CRM (11,2% vs. 10, 2%). Entretanto, IM espontâneo foi mais comum com ICP (6,2% vs. 2,6%), enquanto não houve diferença no risco de AVC (2,7% vs. 3,1%).^451^

Do ponto de vista fisiopatológico, a LTACE não pode ser considerada uma entidade separada dentro do espectro da DAC, já que o principal mecanismo de morte na DAC não pode mais ser atribuído à placa estável (a lesão de tronco), mas ao IM, as lesões instáveis distribuídas em toda a circulação coronariana são as causadoras dos eventos adversos, infarto e morte no paciente com LTACE. A CRM, realizando os enxertos distais nas artérias coronárias, protege contra esses eventos e melhora a sobrevida em 5 anos, como demonstrado no estudo EXCEL.^452^

A ESC e a Associação Europeia de Cirurgia Cardiotorácica (EACTS), conjuntamente, estabeleceram, em 2022, um grupo de trabalho para rever as recomendações das Diretrizes da ESC/EACTS de 2018 sobre revascularização do miocárdio, em pacientes com LTACE com escore SYNTAX baixo a intermediário (0 a 32). Em pacientes estáveis com LTACE e indicação de revascularização, com anatomia coronariana adequada para ambos os procedimentos e baixa mortalidade cirúrgica prevista, o grupo concluiu que ambas as opções de tratamento são clinicamente razoáveis com base na preferência do paciente, experiência disponível e volumes do operador local. As recomendações sugeridas para revascularização com CRM foram Classe I, Nível de evidência A. As recomendações para ICP foram Classe IIa, Nível de evidência A.^453^

#### 6.7.5. Alívio do Sintoma Anginoso e Qualidade de Vida

A melhora da qualidade de vida do paciente propiciando o alívio do sintoma anginoso é outro objetivo da revascularização miocárdica. Para alguns subgrupos de pacientes, como octogenários e nonagenários, os benefícios de melhora da qualidade de vida são de maior importância que o reduzido aumento na expectativa de vida (Tabela 35).

**Tabela 35:** Recomendações para indicação da cirurgia de revascularização miocárdica para redução do risco de infarto do miocárdio e aumento de sobrevida em comparação com a intervenção coronária percutânea e/ou tratamento clínico otimizado

Recomendação	Grau de recomendação	Nível de evidência
Doença de múltiplos vasos principais – envolvendo a DA com escore SYNTAX > 23 – FEVE preservada, com envolvimento proximal da ADA	**I**	**A**
Doença de múltiplos vasos principais e disfunção ventricular esquerda	**I**	**A**
Lesão de tronco da ACE	**I**	**A**

ACE: artéria coronária esquerda; ADA: artéria descendente anterior; DA: descendente anterior; FEVE: fração de ejeção do ventrículo esquerdo.

No período inicial até um mês pós-revascularização, tanto os pacientes submetidos a CRM como a ICP relatam melhora na frequência de angina. No entanto, aos 6 meses e nos anos seguintes, o alívio da angina é significativamente melhor após CRM em comparação com ICP. Da mesma forma, o uso de medicamentos antianginosos é significativamente maior após ICP, mesmo com o uso da nova geração de stents farmacológicos nos últimos anos.

No período pós-procedimento de até um mês, os pacientes submetidos a ICP se recuperaram mais rapidamente do que aqueles que foram operados, relatando menos limitações físicas, menos dor corporal e maior qualidade de vida e satisfação com o tratamento. Essas diferenças desaparecem em 6 meses e, nos anos seguintes, os pacientes com CRM relatam menos problemas físicos e limitações em comparação com aqueles que foram submetidos a ICP. Cerca de 5 anos após a revascularização, benefícios significativos permanecem favorecendo a CRM em termos de saúde física, emocional e mental.

No estudo SYNTAX, o subestudo de qualidade de vida mostrou que, no seguimento de 5 anos, a CRM foi superior à ICP em vários domínios, incluindo frequência e limitação física da angina, bem como nas escalas físicas e emocionais. A análise de subgrupos demonstrou uma interação significativa entre a complexidade angiográfica (avaliada pelo escore SYNTAX) e o alívio da angina. O alívio da angina aos 5 anos foi aumentado com a CRM entre os pacientes com altos escores SYNTAX, um achado que reforça a recomendação de que a revascularização miocárdica deve ser fortemente preferida nesses pacientes.

Nos pacientes com diabetes mellitus, o estudo FREEDOM apresentou a análise de qualidade de vida dos participantes do estudo, usando os dados do *Seattle Angina Questionnaire* coletados no período basal e após revascularização em 1 e 6 meses, e anualmente após. Comparado com o basal, ambos os grupos obtiveram melhora significativa na frequência de angina no seguimento. Entretanto, pacientes submetidos a CRM tiveram melhor alívio da angina nos períodos de 1 e 2 anos. Após 2 anos, as duas estratégias de revascularização proveram resultados semelhantes.

No estudo NOBLE, os autores demonstraram que os pacientes com ICP apresentaram mais sintomas de angina em 5 anos em comparação com aqueles tratados com CRM, sendo que as diferenças nos resultados foram observadas principalmente após 1 ano de acompanhamento.

A Figura 18 mostra a evolução dos pacientes em relação ao desfecho de mortalidade por todas as causas em longo prazo comparando CRM, ICP e TMO, mostrando a continuada divergência das curvas de sobrevida ao longo do tempo, reforçando como a CRM é protetora contra o desfecho de IM na DAC.

**Figura 19:**
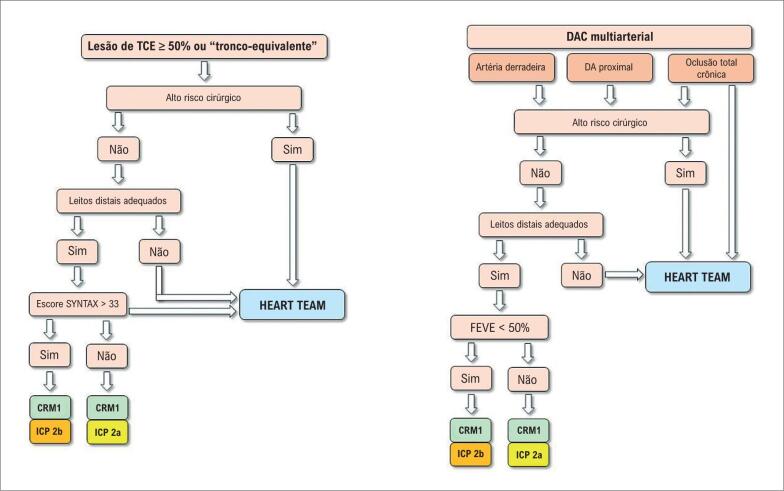
Para redução de morte cardiovascular, especialmente em diabéticos, essa é a orientação inicial para indicar revascularização miocárdica conforme a anatomia e as variáveis básicas de avaliação clínica e de exames habituais de avaliação da fração de ejeção do ventrículo esquerdo. CRM: cirurgia de revascularização do miocárdio; DA: descendente anterior; DAC: doença arterial coronariana; FEVE: fração de ejeção do ventrículo esquerdo; ICP: intervenção coronária percutânea; SYNTAX: Synergy between PCI with Taxus and Cardiac Surgery; TCE: tronco da coronária esquerda.

Na Figura 19, há caminhos que podem servir de orientação inicial nas escolhas para revascularização miocárdica.

## 7. Vacinação Contra Pneumonia, Influenza e COVID-19

Os cardiopatas apresentam maior risco de complicações após algumas doenças infecciosas. Sendo assim, está indicada a vacinação dessa população. As recomendações estão de acordo com o calendário de vacinação do Ministério da Saúde do Brasil de 2024 e a Sociedade Brasileira de Imunizações 2023/2024 (Tabela 36.1).

**Tabela 36.1:** Calendário de vacinação para pacientes cardiopatas

Vacina	Esquema e recomendação	Local de vacinação
Influenza	> 60 anos, preferível a vacina quadrivalente com altas concentrações (high dose HD4V); Vacina quadrivalente (4V) preferível à vacina trivalente (3V).	Vacina trivalente – UBS; Vacina quadrivalente – centro de vacinação privado.
Pneumocócica conjugada (VPC13, VPC15 e VPC20)	Indicada a partir dos 2 meses de idade. No adulto > 60 anos, fazer uma dose de reforço, preferencialmente VPC20.	Disponível no CRIE (VP13 e VP 15) e clínicas privada (VP20)
Pneumocócica polissacarídica 23-valente (VPP23)	Uma dose com dose de reforço após 5 anos	Disponível no CRIE
Vacina para covid-19	Esquema primário = duas doses com intervalo de 4 semanas + terceira dose após 8 semanas; Reforço anual.	Vacina disponível na UBS

CRIE: Centro de Referência para Imunobiológicos Especiais; UBS: Unidade Básica de Saúde. Fonte: Calendários de vacinação da Sociedade Brasileira de Imunizações (SBIm) para pacientes especiais – 2023/2024.^454^

### 7.1. Vacina para Influenza

As vacinas de influenza em uso no Brasil são todas inativadas (de vírus mortos), portanto sem capacidade de causar doença. Até 2014, estava disponível no país apenas a vacina trivalente, com uma cepa A/H1N1, uma cepa A/H3N2 e uma cepa B (linhagem Yamagata ou Victoria). As vacinas quadrivalentes, licenciadas desde 2015, incluem uma segunda cepa B, contendo as duas linhagens: Victoria e Yamagata. Da mesma forma que a trivalente, são inativadas e não possuem adjuvantes. Em 2023, uma nova vacina quadrivalente foi disponibilizada nos serviços privados de vacinação.

A vacina influenza quadrivalente de alta concentração (*influenza high dose*, HD4V) é uma vacina que contém quatro vezes o antígeno em comparação às vacinas influenza quadrivalentes de dose padrão. A vacina é licenciada para indivíduos com 60 anos ou mais. A recomendação da Sociedade Brasileira de Imunizações (SBIm) é a de que pessoas a partir dessa idade – em especial as imunodeprimidas – sejam vacinadas preferencialmente com a vacina HD4V, porque a proteção para influenza e suas complicações oferecida pelas vacinas de dose padrão para a faixa etária é inferior à verificada em jovens. O desenvolvimento de formulações com maior quantidade de antígenos permitiu aumentar a resposta do sistema imunológico dos idosos à vacina, particularmente contra Influenza A (H3N2), mais comum e grave nessa parcela da população.^454^

### 7.2. Efeitos e Eventos Adversos

- Manifestações locais como dor, vermelhidão e endurecimento ocorrem em 15 a 20% dos vacinados. Essas reações costumam ser leves e desaparecem em até 48 horas;
- Manifestações sistêmicas também são benignas e breves. Febre, mal-estar e dor muscular acometem 1 a 2% dos vacinados, tendo início de 6 a 12 horas após a vacinação e persistindo por 1 a 2 dias, sendo mais comuns na primeira vez em que tomam a vacina. Reações anafiláticas são raríssimas.

Sabe-se que a Síndrome de Guillain-Barré (SGB) pode ocorrer por mais de um motivo, mas, em raras ocasiões, seu surgimento coincidiu com a aplicação de uma vacina. Nesses casos, surgiu entre 1 dia e 6 semanas após a vacinação. Até hoje, não se sabe se a vacina influenza pode de fato aumentar o risco de recorrência da SGB em indivíduos que já a tiveram.^454^

### 7.3. Vacina para Pneumococo

Dois tipos de vacinas pneumocócicas estão disponíveis para uso clínico: vacina pneumocócica polissacarídica (VPP) e vacina pneumocócica conjugada (VPC). Os componentes ativos de ambos os tipos de vacina são polissacarídeos capsulares de sorotipos pneumocócicos que comumente causam doença invasiva.

A VPP é composta por polissacarídeos capsulares pneumocócicos parcialmente purificados. A única formulação disponível contém 23 polissacarídeos pneumocócicos dos 23 sorotipos que eram a causa mais comum de doença pneumocócica em adultos na década de 1980.

Vacinas conjugadas (VPC) consistem em polissacarídeos capsulares pneumocócicos ligados covalentemente (conjugados) a uma proteína. Uma vez que foram desenvolvidas pela primeira vez para uso pediátrico, as formulações anteriores incluíam sorotipos que causavam mais doenças em crianças (VPC 10). Formulações mais recentes selecionaram sorotipos que comumente causam doenças em adultos (VPC 13 e VP15 e, a partir de 2023, VP20).

As vacinas são administradas por via muscular (0,5 mL por dose) e podem ser administradas concomitantemente com outras vacinas, em locais diferentes.^455^

### 7.4. Observações para Esquema Sequencial VPC13 e VPP23

1. Sempre iniciar esquema com a vacina conjugada (VPC13), seguida pela aplicação da vacina VPP23, respeitando o intervalo mínimo de 2 meses entre elas;
2. Para indivíduos que já receberam a VPP23 e não foram anteriormente vacinados com VPC13, recomenda-se um intervalo de 12 meses para a aplicação de VPC13, 15 ou 20 e de 5 anos para a aplicação da segunda dose da VPP23, com intervalo mínimo de 2 meses entre as vacinas conjugada e polissacarídica.^455^

### 7.5. Vacina para COVID-19

Atualmente, a vacinação está indicada para toda a população brasileira a partir de 6 meses de idade, e a meta de vacinação preconizada no Brasil é de 90% para o esquema primário completo (Dose 1 e Dose 2 ou dose única [vacina Janssen]) e reforços. Destaca-se que os reforços foram indicados de forma gradativa, iniciando pelo público de 80 anos e mais e reduzindo para as faixas etárias menores, conforme evidências científicas e disponibilidade do produto.^455^

O planejamento da vacinação para 2023 teve como proposta de esquema o uso de vacinas bivalentes com cepas atualizadas como dose de reforço (*booster*) para grupos específicos em maior vulnerabilidade, riscos para complicação e óbito e maior exposição e o uso de vacinas monovalentes para dar início ou completar esquema vacinal das pessoas que não fazem parte dos grupos prioritários elegíveis para vacinação bivalente.

A vacina bivalente está indicada como reforço para população a partir dos 12 anos de idade. Deve ser aplicada a partir de 3 meses após a série primária de vacina ou reforço anterior.

Vale ressaltar que ambas as vacinas, monovalentes e bivalentes, agem do mesmo modo no organismo: estimulando o sistema imunológico a produzir anticorpos protetores e células de defesa contra o vírus SARS-CoV-2. Quando infectada pelo vírus, a pessoa vacinada conseguirá combatê-lo rapidamente, pois já tem imunidade contra ele e, assim, evoluirá de modo assintomático ou com doença leve (maioria dos casos).

O que difere a vacina monovalente da vacina bivalente é a capacidade das vacinas bivalentes de estimularem uma resposta imune mais efetiva contra a variante Ômicron, garantindo, assim, maior proteção contra a infecção pelo SARS-CoV-2. Cabe ressaltar, que as vacinas monovalentes continuam a proteger as pessoas completamente vacinadas contra hospitalizações, síndrome respiratória aguda grave (SRAG) e óbito. As recomendações atuais para imunização contra a covid-19 no país são estabelecidas de acordo com as faixas etárias, os imunizantes disponíveis, as recomendações dos fabricantes e os resultados de estudos nacionais e internacionais.^456^

### 7.6. Vacinas Recomendadas

- Vacina adsorvida covid-19 (inativada) CoronaVac (Butantan);
- Vacina Covid-19-RNAm Comirnaty (Pfizer/Wyeth);
- Vacina Covid-19-recombinante Oxford/Covishield (Fiocruz e Astrazeneca);
- Vacina Covid-19-recombinante Janssen Vaccine (Janssen-Cilag);
- Vacina Comirnaty bivalente Pfizer (para reforço, acima dos 12 anos de idade).

### 7.7. Risco de Miocardite/Pericardite após Vacinação da COVID-19

Segundo dados do Programa Nacional de Imunizações (PNI), até 31 de dezembro de 2022, foram administradas 501.573.962 doses de vacinas contra covid-19 no país. Nesse período, foram notificados 154 casos de miocardite, definidos segundo critérios da Brighton Collaboration (rede global de pesquisa de segurança de vacinas sem fins lucrativos para profissionais de saúde), ocorridos após as vacinas. Isso corresponde a uma incidência de 0,031 a cada 100 mil doses aplicadas. Até o momento, não houve nenhum óbito por miocardite ou pericardite com associação causal com a vacina contra covid-19 no Brasil. Por sua vez, a literatura aponta que a incidência dessas doenças associada à vacina varia entre 0,58 e 2,4 a cada 100 mil doses aplicadas.^457^

A taxa de miocardite associada à covid-19 é de 30 casos por milhão para a população em geral.

## 8. Dieta e Álcool/Controle de Peso

A dieta saudável, caracterizada pela inclusão dos grupos alimentares das frutas, hortaliças, legumes, cereais integrais, castanhas e peixe, está associada a redução dos fatores de risco de mortalidade por DCV (Quadro 8).^458–460^

**Quadro 8:** Principais Recomendações da Dieta

Aumento do consumo diário de frutas e hortaliças para 400 g ou cinco porções (exceção de vegetais ricos em amido, tais como batata e mandioca);
Aumento do consumo de cereais integrais (mínimo 50% do consumo total);
Substituição parcial do consumo de proteína de origem animal por vegetal, tais como castanhas, sementes e grãos (feijão, grão de bico, lentilha etc.);
Redução do consumo de açúcar em até 10% do consumo energético total e até 5% para a obtenção de benefícios adicionais;
Consumo mínimo de produtos ultraprocessados e isenção do consumo de gordura trans;
Substituição parcial dos ácidos graxos saturados por insaturados;
Limitar consumo de bebidas alcoólicas abaixo de 100 g de etanol/semana para indivíduos de alto risco cardiovascular;
Limitar o consumo de sal em 5 g/dia (2 g/sódio).

Os padrões de dieta baseada em vegetais (*plant-based diets*), a exemplo da dieta vegetariana, dieta DASH (*Dietary Approaches to Stop Hypertension*) e dieta do Mediterrâneo, têm sido associados com menor risco de mortalidade por todas as causas e DCVs.^461,462^ Em estudos de avaliação de prevenção secundária, a dieta do Mediterrâneo reduziu o risco de DCV e a progressão de aterosclerose a longo prazo quando comparada a dieta baixa em gordura.^463,464^

O maior consumo de proteína de origem animal, principalmente a carne bovina, comparativamente ao de origem vegetal, aumenta o risco de mortalidade por DCV.^461,462^

No estudo de coorte adventista 2 (*Adventist Health Study* [AHS-2]), o consumo de proteína da carne foi associado com o aumento de 61% do risco de mortalidade, e a sua substituição reduziu esse risco em 40%.^458^

O consumo mínimo semanal de duas porções de peixe ricos em ácido eicosapentaenoico (EPA) e ácido docosahexaenoico (DHA) está associado à redução do risco cardiovascular em prevenção primária e secundária.^213^

A adoção de dietas com quantidades reduzidas de carboidrato e aumentadas em proteína e gordura de origem animal está associada ao maior risco de mortalidade, incluindo a de causa cardiovascular. Dados do estudo ARIC mostram aumento de 18% no risco de mortalidade de indivíduos que seguem dieta baixa em carboidrato e apresentam consumo aumentado de proteína e gordura de origem animal. Ao contrário, quando parte do carboidrato da dieta é substituída por alimentos ricos em proteína e gordura de origem vegetal, o risco de mortalidade é reduzido. Os mesmos investigadores do ARIC realizaram uma análise compilada com o estudo PURE (*Prospective Urban Rural Epidemiology*) relacionada ao consumo de carboidrato e risco de mortalidade. Nessa análise, tanto o baixo quanto o alto consumo de carboidrato aumentaram o risco de mortalidade, sendo o consumo ideal entre 50 e 55%.^465^

A dieta caracterizada pela ingestão de alimentos industrializados, ricos em açúcar, sal e gordura saturada, está associada ao aumento de risco cardiovascular. O consumo de açúcar acima de 10% do total de consumo calórico tem sido associado ao aumento de risco de DCV.^466,467^ O consumo de bebidas açucaradas aumenta o risco de DM2 e DCV aterosclerótica, sendo o risco de diabetes elevado em 20% para cada consumo diário de um copo.^468^ A dieta caracterizada pelo consumo de sucos, doces, bebidas açucaradas, cereais refinados e frituras aumenta eventos coronários mais do que a ingestão de produtos de origem animal.^469^

O consumo de gorduras saturadas está associado com o aumento do risco de mortalidade. A gordura saturada aumenta o colesterol e o risco de SCC; a sua substituição parcial por gordura poli-insaturada, como de óleo de soja, reduz o risco da doença em 29%.^470^

A substituição de qualquer tipo de gordura por trans aumenta o LDL-c, a apoB, os triglicérides, a lipoproteína (a) e reduz o HDL-C e a apolipoproteína A1. O consumo de gordura trans está associada ao aumento do risco de SCC.^470^

O consumo leve a moderado de bebidas alcoólicas, correspondente a uma a duas doses ou 15 a 30 g/etanol/dia não aumenta o risco de IM. A ingestão acima de 100 g/semana, entretanto, está associada com o aumento do risco de mortalidade por várias causas, incluindo as cardiovasculares e a reincidência de eventos entre indivíduos portadores da doença.^471,472^. O consumo de bebidas alcoólicas não deve ser recomendado, como medida de proteção cardiovascular.^473^

### 8.1. Controle de Peso

A perda de 5% do peso inicial está associada com a melhora de vários parâmetros metabólicos, incluindo a redução de LDL-C.^474^

A redução calórica ≥ 500 kcal/dia geralmente é prescrita para indivíduos com sobrepeso e obesidade.^475^

Intervenções de um programa estruturado que compreendem a automonitoração do consumo calórico e checagem de peso, além da atividade física, proporcionam perda de 5 a 10% do peso inicial a curto (≥ 6 meses) e médio prazo (6 a 12 meses).^474,475^

A recomendação de atividade física aeróbica para a população adulta é ≥ 150 min/semana ou 75 a 150 min de moderada e elevada intensidade, respectivamente, em associação ao exercício de resistência.^475^ O aumento de tempo para no mínimo 300 min/semana pode ser recomendado para indivíduos com obesidade, e a combinação do exercício aeróbico com o de resistência pode ser superior na redução de gordura abdominal e aumento de massa muscular.^476^

O índice de massa corporal (IMC) é o método mais utilizado e padronizado para a classificação de sobrepeso e obesidade. O cálculo do IMC é a principal avaliação a ser realizada no atendimento do paciente que necessita de perda de peso. O seu resultado deve ser interpretado com cautela em indivíduos com maior massa muscular, em idosos e asiáticos. Além do IMC, a circunferência da cintura detecta a obesidade central e está associada com o aumento do risco cardiometabólico e aumento do risco da DCV aterosclerótica.^477,478^ A circunferência da cintura é considerada elevada com valores ≥ 102 cm para homens e ≥ 88 cm para mulheres.^474,479^ A circunferência da cintura é um dos critérios de diagnóstico da síndrome metabólica, e sua combinação com o IMC é o melhor critério de avaliação de risco relacionado a obesidade.^474^

## 9. Atividade Física, Reabilitação Cardíaca e Atividade Sexual

### 9.1. Mudanças no Estilo de Vida e Outros Fatores

#### 9.1.1. Sedentarismo, Atividade Física e Exercício Físico

Três conceitos são fundamentais quando abordamos a atividade física:

- Comportamento sedentário: refere-se ao tempo dispendido sentado ou deitado, quando acordado, em atividades com gasto energético ≤ 1,5 múltiplos de METs;
- Atividade física: definida como qualquer movimento corporal que aumente o gasto energético acima do que representa o metabolismo basal e inclui os domínios ocupacional, doméstico, transporte e lazer;
- Exercício físico: é uma forma de atividade física, estruturada e programada, com o principal objetivo de melhorar ou manter a saúde e/ou o desempenho esportivo.^475^

O comportamento sedentário favorece o desenvolvimento da DAC e está associado a mortalidade por todas as causas, por DCVs, por câncer e ao aumento da incidência de diabetes tipo 2.^475,480,481^ Além de aumentar os níveis de atividade física, reduzir o comportamento sedentário é de extrema importância: a) 6 horas de tempo sentado influencia na função vascular de indivíduos saudáveis;^482^ b) interromper esse comportamento, levantando e caminhando por 10 minutos, reduz a disfunção vascular;^483^ c) relação dose-resposta com todas as causas de morte tem um acréscimo pronunciado a partir de 9,5 horas de tempo sedentário.^484^ Diante dessas evidências, a diretriz da Organização Mundial da Saúde (OMS) de 2020 recomenda os níveis de atividade física apresentados na (Tabela 36.2),^475,485^ defendendo que qualquer atividade física deva ser incentivada para a manutenção da saúde global e para redução da morbimortalidade cardiovascular e por todas as causas.

**Tabela 36.2:** Recomendação de atividade física para adultos, incluindo indivíduos com doença cardiovascular estável

Tipo	Recomendação	Exemplos de mensuração de intensidade	Grau de recomendação	Nível de evidência
Aeróbico (caminhada, corrida, pedalada em bicicleta, natação, contínuos ou por breves períodos).	150 a 300 min/semana de MI; 75 a 150 min/semana de AI.	Escala de PSE Borg de 10 a 13 para MI e acima de 13 para AI;^488^ Respiração ofegante, porém, de modo que se consiga completar uma frase sem pausa para MI, respiração ofegante sem que se consiga completar uma frase completa para AI.^488^	**I**	**A**
Fortalecimento muscular (exercícios contra resistência ou exercícios resistidos).	MI ou AI envolvendo grandes grupos musculares 2 dias/semana.	Escala de PSE OMNI de 6-8 para MI e 9 e 10 para AI.*^489^	**I**	**A**

AI: alta intensidade; MI: moderada intensidade; PSE: percepção subjetiva de esforço.

* Para utilização da escala OMNI de percepção subjetiva de esforço nos exercícios resistidos, o Grau de recomendação é IIa, e o Nível de evidência é C.

Em estudo longitudinal com 15.486 pacientes portadores de DAC e seguimento de 3,7 anos, a diminuição da mortalidade cardiovascular e por todas as causas foi gradualmente associada com o aumento da atividade física diária.^486^

Ao que se refere às atividades de fortalecimento muscular (treinamento resistido), uma revisão sistemática recente, com 16 estudos incluídos e seguimento máximo de 25,2 anos, observou redução de 10 a 17% no risco de morte cardiovascular, com 30 a 60 minutos por semana dessa atividade.^487^

#### 9.1.2. Prevenção Secundária (Reabilitação Cardíaca Baseada em Exercício)

A indicação de reabilitação cardíaca para o tratamento de portadores de SCC é consenso entre a diretriz nacional^488^ e as diretrizes internacionais^1,80^ (Classe: I, Nível de evidência: A). Tal recomendação se deve à comprovada eficiência da reabilitação cardíaca na melhora da qualidade de vida, redução dos fatores de risco modificáveis e mortalidade, além de prevenção nas readmissões por novos eventos cardiovasculares. Nesse contexto, a reabilitação cardíaca também evidencia uma relação positiva de custo-efetividade para o sistema de saúde.^490–492^ Um estudo com 5 anos de acompanhamento descreveu redução de 32% no risco de morte em pacientes previamente infartados ou revascularizados, assim como redução naqueles com angina instável e estável que participaram de programas de reabilitação, sempre em relação aos que não participaram.^493^

Em 2021, uma revisão sistemática, com inclusão de 85 estudos clínicos randomizados e um total de 23.430 pacientes com DAC, concluiu que a reabilitação cardíaca baseada em exercício diminui o risco de IM. Nessa mesma linha, promoveu redução na mortalidade e hospitalização por todas as causas, juntamente com redução nos custos de saúde e melhora na qualidade de vida em até 12 meses de acompanhamento. No seguimento de longo prazo (> 3 anos), observou-se redução na mortalidade cardiovascular e no risco de IM.^494^

O exercício como intervenção é a principal estratégia para melhora significativa da capacidade física (consumo de oxigênio). Cada aumento de 1 mL.kg^-1^.min^-1^ no consumo máximo de oxigênio foi associado a uma redução de ~15% no risco de morte cardiovascular e de ~17% por mortes por todas as causas em pacientes com DAC.^495^ Em pacientes com DAC crônica, o exercício aeróbico é capaz de impactar muito positivamente na perfusão miocárdica. Em trabalho clássico, Hambrecht e colaboradores expuseram pacientes com DAC ao treinamento aeróbico realizado em bicicleta ergométrica, sete vezes por semana (60 minutos por dia), a 80% da FC-pico alcançada em teste de exercício máximo. Nesse experimento de eficácia, houve melhora da função endotelial arterial coronariana daqueles treinados em relação aos do grupo-controle.^496,497^

Cabe salientar que, com a manutenção da prática do exercício, o aumento na perfusão miocárdica é observado por meio do aumento do limiar isquêmico e da redução do duplo produto em cargas submáximas, além de ser observada melhora perfusional cintilográfica,^498^ na modulação autonômica,^499^ bem como pelo aumento do tempo diastólico. Todas essas adaptações corroboram a redução das taxas de mortalidade cardiovascular e hospitalizações, além da melhora na qualidade de vida (Classe: I; Nível de evidência: A).

Bem menos evidências existem a respeito do exercício resistido isolado na melhora do consumo de oxigênio e perfusão miocárdica em paciente com DAC. No entanto, o exercício resistido pode ser uma atividade complementar essencial de um programa de reabilitação, já que, ao ocorrer melhora na força muscular, há diminuição na sobrecarga cardíaca para a mesma carga absoluta de exercício executado para atividades de vida diária, assim como para melhora da qualidade de vida.^500,501^

A avaliação de risco do paciente antes da prescrição de um programa de exercício físico é recomendada.^488,502,503^ A Diretriz Brasileira de Reabilitação Cardiovascular de 2020,^488^ previamente publicada e exposta na Tabela 37, também vai ao encontro dessa premissa. A estratificação de risco do paciente com SCC é dinâmica, podendo ser modificada ao longo do tempo. Reavaliações semestrais ou anuais (a depender do risco inicial identificado) são necessárias. Para a classificação de risco, deve-se considerar pelo menos um dos parâmetros da Tabela 38. Recomendações sobre o treinamento físico estão dispostas na Tabela 38.

**Tabela 37:** Estratificação de risco para o exercício físico em pacientes com doença coronariana crônica

	Risco baixo	Risco intermediário	Risco alto
**Capacidade funcional**	TE: > 7 METs; TCPE: Classe A de Weber ou VO_2_ pico > 85% do predito.	TE: 5 a 7 METs; TCPE: VO_2_ pico de 60 a 85% do predito.	TE: < 5 METs; TCPE: VO_2_ pico < 60% do predito.
**Sintomatologia**	Ausente	CF I a II, angina e/ou IC	CF III e IV, angina e/ou IC
**Sinais e sintomas de isquemia miocárdica**	Ausente	TE: isquemia ou sintoma acima de 6 METs; TCPE: isquemia ou sintoma acima de 15 mL.kg^-1^.min^-1^.	Em baixas cargas: TE: abaixo de 6 METs; TCPE: abaixo de 15 mL.kg^-1^.min^-1^.
**Outras características clínicas**	Ausência de arritmias ventriculares complexas.	Ausência de arritmias ventriculares complexas.	IRC dialítica; queda da saturação de oxigênio em esforço; arritmia ventricular complexa.

CF: classe funcional; IC: insuficiência cardíaca; IRC: insuficiência renal crônica; METs: múltiplos de equivalentes metabólicos; TCPE: teste cardiopulmonar de exercício; TE: teste ergométrico. Adaptado de Carvalho et al.^488^

**Tabela 38:** Recomendação de exercício físico para pacientes com doença coronariana crônica

Tipo de atividade	Risco baixo	Grau de recomendação	Nível de evidência
Exercício aeróbico	**Intensidade:** PSE (Borg 6 a 10) ou teste da fala (execução do exercício quando a respiração é ofegante, porém, de modo que se consiga completar uma frase sem pausa. 40 a 80% da FC de reserva: ([FC máxima - FC repouso] x intensidade) + FC repouso. Em previamente ativos, 50 a 80%. FC entre LV1 e LV2 do TCPE (progredindo para alcançar o LV2).	I	A
	**Frequência e duração:** mínimo de duas vezes/semana, iniciar com 30 minutos por sessão e progredir gradualmente para 60 minutos ao longo do período de TF (> 60 minutos; prevenir a desidratação; caso a FC fique acima do limite superior com a manutenção da carga, reduzir a intensidade).		
	**Tipo:** bicicleta*, caminhada, corrida etc.		
Exercícios de fortalecimento (resistido)	**Frequência e volume por sessão:** duas a três vezes/semana, um a dois exercícios para os principais grandes grupos musculares, com uma a três séries de 10 a 15 repetições até a fadiga moderada (repetição em que há redução da velocidade e perda do padrão do movimento). **Intensidade:** 60% de 1RM. Pausas passivas: 90 a 120 s; ou pausa ativa – alternar por segmento (MMII e MMSS) e/ou musculatura agonista e antagonista. Evitar manobra de Valsalva.	I	A
	**Risco intermediário e alto.**		
Exercício aeróbico	**Intensidade:** PSE (Borg 11 a 15) ou teste da fala (execução dos exercícios quando a respiração é ofegante, porém de modo que se consiga completar uma frase sem pausa.^#^ 40 a 80% da FC de reserva: ([FC máxima - FC repouso] x intensidade) + FC repouso. Com disfunção ventricular de 40 a 60% e, previamente ativos, 50 a 80%. FC entre o LV1 e LV2 do TCPE (progredindo para o alcance do LV2). Se apresentar sinais e/ou sintomas de isquemia miocárdica, utilizar intensidade de 10 bpm abaixo da FC de positivação (determinada por teste funcional).^16^	I	A
	**Frequência e duração:** mínimo duas vezes/semana. Iniciar com 30 minutos e progredir gradualmente para 60 minutos ao longo do período de TF.		
	**Tipo:** bicicleta*, caminhada, corrida.		
Exercícios de fortalecimento (resistido)	**Frequência e volume por sessão:** duas a três vezes/semana, um a dois exercícios para os principais grandes grupos musculares, com uma a três séries de 10 a 15 repetições até a fadiga moderada (repetição em que há redução da velocidade e perda do padrão do movimento). **Intensidade:** 60% de 1RM. Pausas passivas: 90 a 120 s; ou pausa ativa – alternar por segmento (MMII e MMSS) e/ou musculatura agonista e antagonista. Evitar manobra de Valsalva.	IIa	C

bpm: batimentos por minuto; FC: frequência cardíaca; LV1: 1° limiar ventilatório; LV2: 2° limiar ventilatório; MMII: membros inferiores; MMSS: membros superiores; PSE: percepção subjetiva de esforço, RM: repetições máximas; TCPE: teste cardiopulmonar de exercício; TF: treinamento físico; *Se o teste de exercício for realizado na bicicleta, seguir a prescrição de intensidade de FC sugerida. Caso tenha sido realizado na esteira ergométrica, realizar a atividade 10% abaixo da sugerida. #Utilizar o monitoramento da intensidade pela PSE ou teste da fala somente como estratégia complementar.

Em pacientes com maior limitação, como aqueles com AR, que apresentam limiar de isquemia próximo da FC de repouso, o treinamento aeróbico pode ser realizado no limiar de isquemia, porém, em ambiente hospitalar e monitorizado (Classe: IIb, Nível: C).^488,504–506^ Estudos apontam que curtos períodos de isquemia miocárdica induzida pelo exercício podem contribuir na cardioproteção (precondicionamento isquêmico),^505^ mas essa estratégia não costuma ser amplamente utilizada no dia a dia da reabilitação.

Recomenda-se que a sessão de exercício seja composta por aquecimento (intensidade abaixo da prescrita), parte principal (intensidade prescrita, sugerida na Tabela 38) e recuperação, com redução lenta e gradual da carga, com 15 a 30 minutos de exercícios resistidos no final do treino aeróbico. Alongamento e relaxamento são opcionais, dependendo da disponibilidade de tempo para a sessão de exercício.

Alguns cuidados devem ser incorporados à sessão de exercício físico em pacientes com SCC, conforme citados a seguir:

- Aquecimento de 5 a 10 minutos (em ambientes com temperatura mais baixa, realizar 10 minutos de aquecimento);
- A recuperação do exercício deve ser gradual, entre 5 a 10 minutos. Sugere-se 1 minuto a cada 1 km/h de redução de velocidade, no caso de exercício de caminhada;
- Deve ser realizada a mensuração de pressão arterial antes do início, durante e após a sessão, principalmente em hipertensos. Para valores em repouso superiores a 160/105 mmHg, recomenda-se o ajuste dos fármacos anti-hipertensivos para melhor controle pressórico antes de iniciar às sessões de exercício;507
- Durante o exercício, é recomendado que a pressão arterial se mantenha ≤ 180/105 mmHg. Se estiver superior a esse nível, a redução da intensidade do exercício é recomendada.488,507

Apesar de o exercício aeróbico moderado ser mais amplamente prescrito em relação ao exercício aeróbico de alta intensidade (treinamento intervalado de alta intensidade [TIAI]), este pode ser uma boa opção para otimização no tempo. Sabe-se que essa estratégia é segura para pacientes com DAC^508,509^ e que, de fato, promove maior aumento da capacidade aeróbica.^510^ Entretanto, um período de adaptação prescrito em moderada intensidade (Tabela 38), de 1 a 3 meses,^511^ antes do aumento da intensidade do exercício, é de extrema importância para se evitarem lesões musculares, que são mais frequentes nesse tipo de exercício (Classe: I, Nível: A).

#### 9.1.3. Reabilitação Domiciliar

A reabilitação cardíaca supervisionada demonstrou ter efeitos positivos no controle da DAC, como já mencionado. No entanto, existem algumas barreiras à participação em programas tradicionais de reabilitação cardíaca, como o deslocamento do paciente e, principalmente, a dificuldade da reabilitação em ambientes e clínicas com poucos recursos. Programas de reabilitação cardíaca domiciliar ou semissupervisionada e a telerreabilitação (supervisionada remotamente), que incluem educação em saúde associada à prática do exercício, podem ser uma opção.^512,513^

Há mais de 30 anos, a reabilitação realizada em ambiente domiciliar, para pacientes estáveis, já trazia indícios de benefícios cardiovascular.^514,515^ Atualmente, depois do advento da pandemia da covid-19, mais atenção foi dada a esse tipo de intervenção terapêutica baseada no exercício. Além dos benefícios clássicos, o fator custo-efetividade faz com que esse método seja de grande valia. Contudo, a reabilitação domiciliar ainda precisa ser mais estudada para a prescrição em casos de pacientes com risco intermediário e alto, assim como para indivíduos sem experiência prévia com a prática do exercício físico^516–519^ (Classe: IIA, Nível: A, para baixo risco).

#### 9.1.4. Atividade Sexual

A atividade sexual tem relação com qualidade de vida e habitualmente, é reduzida em pacientes com DCVs. Fatores psicológicos influenciados pela condição cardíaca, como ansiedade e depressão, além de disfunção erétil e perda da libido podem ser responsáveis por essa redução. Portanto, abordar o tema e oferecer aconselhamento e tratamento adequados é de fundamental importância e deve fazer parte da boa prática clínica, assim como é feito com orientações relacionadas ao retorno ao trabalho, execução de exercícios e outras atividades de vida diária.^520^

É possível que a atividade sexual seja a forma de exercício mais prazerosa que existe, podendo trazer diversos benefícios para a saúde física e emocional do paciente, incluindo: 1) melhora da qualidade de vida, contribuindo para o bem-estar geral e redução do estresse, da ansiedade e da depressão; 2) fortalecimento do relacionamento e do vínculo entre o paciente e seu parceiro(a); e 3) manutenção da saúde cardiovascular, com melhora da circulação sanguínea e da saúde cardiovascular geral.

Apesar de ser de grande preocupação tanto para os agentes de saúde quanto para os pacientes com DAC, o risco de morte súbita ou infarto durante a atividade sexual é muito baixo. Isso é particularmente verdadeiro quando ela é realizada com um parceiro(a) estável, em um ambiente familiar, sem estresse exagerado ou sem consumo de drogas e/ou consumo excessivo de bebida alcoólica.^520,521^ Nesses casos, costuma ser uma atividade de baixa a moderada intensidade (2 a 4 METs). Aliás, estima-se que morte súbita durante atividade sexual corresponda a menos de 2% de todas as mortes relacionadas ao exercício.^522–524^ Já o risco de infarto é transitoriamente aumentado, por volta de três vezes nas primeiras 2 a 3 horas após o coito, quando comparado a atividades com gasto energético similar. No entanto, cabe salientar que esse risco é reduzido nos indivíduos fisicamente ativos.^525–527^

**Figura 20:**
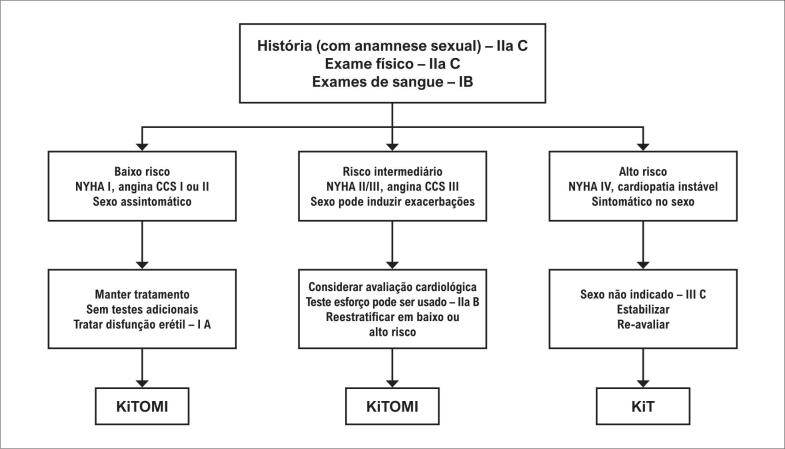
Cardiopata isquêmico que deseja ter atividade sexual. NYHA: New York Heart Association; CCS: Canadian Cardiovascular Society; Kit: Kiss and Touch; KITOM: Kiss, Touch, Oral and Masturbation; KiTOMI: Kiss, Touch, Oral, Masturbation and Intercourse. Com permissão do Canadian Journal of Cardiology.

A atividade sexual é bem tolerada pela maioria dos pacientes com DAC crônica e abrange vários comportamentos, como beijo (Ki), toque (To), estimulação oral (O) dos órgãos genitais, masturbação (M) e relação sexual com penetração (I). Ao utilizar as iniciais dessas palavras, pesquisadores brasileiros propuseram o acrônimo KiTOMI (*Kiss, Touch, Oral Sex, Masturbation e Intercourse)*.^41^ Após eventos/procedimentos cardíacos maiores, ela deve ser retomada gradualmente, começando com o KiT e avançando progressivamente para o KiTOM antes de atingir todas as atividades do KiTOMI. O grau de recomendação e o nível de evidência, quando disponíveis, são relatados (Figura 20).^520^

Por fim, é importante citar que, em casos de disfunção sexual relacionada à SCC, intervenções psicológicas podem ser úteis para abordar fatores psíquicos que contribuem para o problema, como ansiedade, depressão e baixa autoestima. A terapia individual ou em casal pode ser eficaz no tratamento da disfunção erétil, falta de desejo e de outros distúrbios sexuais. Além disso, alguns medicamentos podem ter relação com disfunção sexual, como betabloqueadores e diuréticos tiazídicos. Entretanto, com classes mais novas de medicação, essa relação não tem sido comprovada. Entre os betabloqueadores, o nebivolol parece ter menor ação no quesito disfunção sexual. Cuidado especial precisa ser reforçado quando há o uso de inibidores da fosfodiesterase-5 para tratar a disfunção erétil, os quais são geralmente seguros em pacientes com SCC, mas não devem ser usados nos pacientes que fazem uso de nitratos.

## 10. Tabagismo e Poluição Ambiental

Tanto a fumaça do tabaco quanto a poluição do ar contêm dióxido de carbono, monóxido de carbono e óxidos de nitrogênio, sendo que a fumaça do cigarro também contém quantidades significativas de cianeto de hidrogênio, nicotina, alcatrão, benzeno, acroleína e nitrosaminas.^528^ A poluição do ar proveniente da combustão de combustíveis fósseis tende a conter níveis mais altos do gás dióxido de nitrogênio (NO2).^529^ Ambas as fontes contêm material particulado de tamanhos variados, especialmente partículas muito pequenas de tamanho nano, chamadas de partículas ultrafinas. As partículas provenientes da combustão tendem a ser compostas de carbono, com uma infinidade de produtos químicos em sua superfície, incluindo espécies de carbono orgânico e metais-traço, ambos implicados em efeitos na saúde.

O uso de tabaco em ambientes internos contribui significativamente para a poluição do ar.^528^ Quando o tabaco é queimado, ele libera substâncias tóxicas como nicotina, monóxido de carbono, óxidos de nitrogênio, compostos orgânicos voláteis (COVs) e material particulado fino (PM2.5 – partículas menores que 2,5 micrômetros de diâmetro) para o ar circundante.^529^ Essas partículas são especialmente preocupantes, porque podem penetrar profundamente no sistema respiratório e são altamente patogênicas. A fumaça passiva é definida como a combinação da fumaça emitida da ponta acesa de um cigarro ou outros produtos de tabaco e a fumaça exalada pelo fumante. É uma preocupação significativa de saúde pública devido aos seus efeitos adversos na saúde humana^530^ e é uma importante fonte de PM2.5 em ambientes internos.^528^ Assim, a exposição à fumaça passiva consiste na inalação não intencional de fumaça que ocorre perto de pessoas fumando e/ou em ambientes internos onde produtos de tabaco foram usados recentemente. A fumaça de terceira mão (*thirdhand smoke* [THS]) consiste em gases residuais de fumaça de cigarro e partículas que se depositam na superfície.^531^ Esses poluentes persistem muito tempo após a dispersão da fumaça de segunda mão. Estudos que avaliam a presença de nitrosaminas específicas do tabaco (*tobacco-specific nitrosamines* [TSNA]) na THS indicam que essas são importantes substâncias carcinogênicas.^532^

Estudos têm mostrado que os níveis de PM2.5, compostos orgânicos voláteis e concentração de monóxido de carbono em ambientes internos podem ser significativamente mais altos em locais onde é permitido fumar em comparação com ambientes livres de fumaça.^533^ Após a implementação de políticas de ambientes livres de fumaça em locais de hospitalidade internos (incluindo bares, cafeterias, restaurantes e *pubs*), fumantes foram deslocados para áreas externas e pesquisas avaliaram a exposição à fumaça de tabaco ao ar livre (*outdoor tobacco smoking* [OTS]) nessas áreas abertas para onde os fumantes foram deslocados. Sureda et al.^534^ encontraram que a exposição ao OTS é prevalente em locais de hospitalidade ao ar livre, especialmente em locais com ventilação inadequada. Hwang et al.^535^ e Kim et al.^536^ demonstraram que a exposição ao OTS é detectável mesmo a distâncias de 9 e 3 m, respectivamente, de uma fonte de fumo. Ruprecht et al.^537^ enfatizaram ainda mais os efeitos adversos do OTS na qualidade do ar, com implicações significativas para a saúde pública. Essas descobertas destacam a necessidade de políticas e intervenções eficazes para reduzir a exposição ao OTS em ambientes externos. Instalações de fumantes ao ar livre devem ter uma zona de amortecimento para reduzir a exposição ao OTS.^536^

É importante considerar o impacto dos cigarros eletrônicos (*vapes*) em ambientes internos, também chamado de *vaping* de segunda mão (*second hand vaping)*, que ocorre quando o aerossol do cigarro eletrônico é exalado por usuários em locais públicos ou ambientes privados fechados.^538^ Portanto, embora o uso de cigarros convencionais em ambientes internos seja reconhecido como uma das principais fontes de poluentes tanto na fase gasosa quanto na fase particulada, resultando em altos níveis de exposição tanto para fumantes ativos quanto passivos a compostos carcinogênicos e material particulado, a exposição ao aerossol de cigarros eletrônicos de segunda mão não é isenta de riscos devido à alta concentração de partículas ultrafinas (nanopartículas, partículas menores de 1 micrômetro),^539^ cuja capacidade de penetração e ação de entrega de produtos químicos/contaminantes nas regiões mais profundas do sistema respiratório humano causa inflamação ao sistema respiratório. Portanto, o *vape* leva a exposições ao aerossol de cigarro eletrônico de segunda e terceira mão.

Alguns trabalhos sugerem que exposições de terceira mão induzidas por cigarro eletrônico são comparáveis às induzidas pelo tabagismo convencional considerando a quantidade elevada de particular ultrafinas e nitrosaminas.^538,539^ Os pesquisadores^538^ monitoraram poluentes em lojas que vendem exclusivamente *vapes*. As concentrações de PM2.5, formaldeído, acetaldeído e nicotina no ar durante o horário de funcionamento da loja foram 21, 3,3, 4,0 e 3,8 vezes mais altas do que os níveis durante o horário de fechamento da loja, respectivamente. As concentrações de PM2.5 estavam correlacionadas com o número de usuários de e-cigarros presentes nas lojas de vapor nicotina na superfície, 4-(N-metil-N-nitrosamino)-4-(3-piridil)butanal (NNA) e 4-(metilnitrosamino)-1-(3-piridil)-1-butanona (NNK) também foram detectadas em níveis de 223,6 ± 313,2 *μ*g/m^2^, 4,78 ± 11,8 ng/m^2^ e 44,8 ± 102,3 ng/m^2^, respectivamente. Quantidades substanciais de nicotina (até 2.073 *μ*g/m^2^) depositadas nos materiais colocados dentro das lojas de vapor, NNA (até 474,4 ng/m^2^) e NNK (até 184,0 ng/m^2^) também foram formadas nesses materiais. As concentrações de nicotina depositadas estavam fortemente correlacionadas com o número médio de usuários ativos de e-cigarros presentes em uma loja de vapor por hora. Os níveis de NNK nas superfícies dos materiais estavam significativamente associados aos níveis de nicotina na superfície.

A associação entre poluição, incluindo poluição tabagística, e DCVs é bem estabelecida, com vários estudos epidemiológicos documentando uma relação consistente entre a exposição a poluentes e o aumento do risco de eventos cardiovasculares adversos, como IM, AVC e morte súbita cardíaca.^540^ Os mecanismos subjacentes a essa associação são complexos e envolvem uma série de processos biológicos que contribuem para a patogênese das DCVs. Estresse oxidativo, inflamação, disfunção endotelial, disfunção autonômica e remodelação vascular são todos considerados mediadores importantes dos efeitos cardiovasculares da poluição do ar.^541^

O estresse oxidativo é um processo-chave na patogênese das DCVs induzidas pela poluição.^542^ A exposição a poluentes, como partículas finas (PM2.5), presentes em cigarros eletrônicos e convencionais, leva à geração aumentada de espécies reativas de oxigênio (*reactive oxygen species* [ROS]) e diminuição da capacidade antioxidante do organismo.^543^ Essa desregulação do equilíbrio redox resulta em dano oxidativo a componentes celulares, incluindo lipídios, proteínas e DNA, levando à ativação de vias inflamatórias e pró-aterogênicas.^544^ A inflamação desempenha um papel fundamental na resposta biológica à exposição à poluição do ar. A inalação de poluentes do ar desencadeia uma resposta inflamatória nas vias aéreas e nos pulmões, caracterizada pela produção aumentada de citocinas pró-inflamatórias, quimiocinas e mediadores lipidícos.^544^ Esses mediadores inflamatórios circulam pelo corpo e podem desencadear inflamação sistêmica e disfunção endotelial, promovendo, assim, a formação e progressão de aterosclerose.^545^

A exposição aguda a poluentes do ar diminui a biodisponibilidade de óxido nítrico (NO), um regulador-chave da função vascular, e aumenta a produção de endotélio de ROS, incluindo peróxido de hidrogênio (H_2_O_2)_ e superóxido (O_2_•−).^543^ Essas mudanças promovem a vasoconstrição, inflamação vascular e agregação plaquetária, todas as quais contribuem para o desenvolvimento de eventos cardiovasculares agudos.^541^

A poluição pode afetar o sistema nervoso autônomo,^547^ perturbando a regulação cardiovascular e promovendo arritmias cardíacas^548^ e o aumento da pressão arterial.^549^ A exposição a poluentes do ar pode desencadear respostas autonômicas disfuncionais, incluindo aumento da atividade simpática e diminuição da atividade parassimpática, que estão associadas a alterações na variabilidade da FC e maior risco de eventos cardiovasculares.^548^

A remodelação vascular é outro mecanismo pelo qual a poluição do ar pode contribuir para o desenvolvimento de DCVs. A exposição crônica a poluentes do ar está associada a alterações estruturais nas artérias, incluindo espessamento da parede arterial, aumento da rigidez vascular e formação de placas ateroscleróticas.^550^ Essas mudanças na morfologia vascular comprometem a função arterial e aumentam o risco de eventos cardiovasculares.

Em resumo, a poluição do ar exerce efeitos adversos sobre o sistema cardiovascular por meio de uma variedade de mecanismos, incluindo estresse oxidativo, inflamação, disfunção endotelial, disfunção autonômica e remodelação vascular. A compreensão desses mecanismos é crucial para o desenvolvimento de estratégias eficazes de prevenção e intervenção destinadas a mitigar os efeitos cardiovasculares da poluição do ar.

## 11. Fatores Psicossociais e Adesão ao Tratamento

### 11.1. Fatores Psicossociais

Fatores psicológicos negativos (ansiedade, estresse, depressão), traços de personalidade e transtornos mentais apresentam uma relação bidirecional com a doença coronariana crônica, ou seja, são fatores de risco para sua ocorrência e também complicações desta.^551,552^ Os mecanismos causais entre transtornos mentais e a DAC são complexos e permanecem obscuros.^551–554^ Além da relação causal, considera-se haver um mecanismo fisiopatológico comum entre transtornos mentais e a DAC, bem como a coexistência de outros fatores de risco como variáveis de confusão (sedentarismo, tabagismo, hipertensão, obesidade),^617^ que dificultam o desenvolvimento de evidências claras.

A relação entre ansiedade e estresse com mortalidade e morbidade cardiovascular também permanece inconclusiva.^551^ Há evidências de que cerca de 40% de pessoas com doenças cardíacas, incluindo DAC, sofrem de depressão ou ansiedade.^553^ Quanto à depressão, contudo, estudos prospectivos mostraram que é fator de risco independente de morbidade e mortalidade por doença coronariana.^551^

Além de piores desfechos, fatores psicossociais (estado de humor depressivo, estresse, ansiedade e isolamento social) dificultam a adoção de mudanças no estilo de vida e podem comprometer a adesão ao tratamento.^1^

Além do tratamento medicamentoso, inclui-se a necessidade de apoio emocional para tratamento do estresse, ansiedade e depressão de pacientes com angina estável^555^ e o encaminhamento para psicoterapia.^1^ Há evidências quanto à eficácia de intervenções psicológicas específicas (incluindo as tradicionais como a terapia cognitivo-comportamental e a terceira onda, como *mindfulness*) de nível de evidência moderado para redução de depressão e ansiedade; nível baixo para melhora da qualidade de vida relacionada à saúde mental, nas não à saúde física. As intervenções não foram eficazes na redução de mortalidade (nível de evidência moderado) e redução de risco de eventos cardíacos (nível de evidência baixo).^553^

Apesar disso, destaca-se o benefício das intervenções psicológicas na valorização da personalização dos cuidados em saúde mental para favorecer que o tratamento da SCC seja adaptado às necessidades específicas, possibilidades e preferências de cada indivíduo, ou seja, para que o paciente seja colocado no centro do cuidado, e não a sua doença.^552,554^ A inclusão deste tópico na presente diretriz em relação à anterior de 2014 representa um avanço nesse sentido; para além dos resultados nos desfechos clínicos, considera-se a atenção com o processo de saúde e doença e como o paciente o enfrenta.

### 11.2. Adesão ao Tratamento

A adesão ao tratamento medicamentoso e não medicamentoso é um grande desafio e também uma necessidade. Diversas pesquisas têm objetivado estudar intervenções que possam favorecer a adesão ao tratamento, envolvendo tecnologia digital, como aplicativos de *smartphone*^556^ e envio de mensagens de texto,^557,558^ sem resultados favoráveis; e intervenção de autogerenciamento por telefone celular^559^ e aplicativos de lembrete de medicação;^560^ com melhora de adesão ao tratamento, mas necessitando de evidências mais robustas. Há resultados efetivos também em adesão à dieta alimentar, atividade física e medicação através da educação centrada no paciente,^561^ contudo, também necessitando de mais estudos nesse sentido.

A relação de outras variáveis com a adesão ao tratamento também tem sido estudada. Mulheres aderem menos ao tratamento de reabilitação cardíaca do que homens,^562^ e a baixa alfabetização em saúde foi associada à menor adesão a antianginosos.^563^

## 12. Angina e Isquemia sem Obstrução das Artérias Coronárias (INOCA, ANOCA)

O termo "doença isquêmica cardíaca" (DIC) é mais adequado para se referir às diversas afecções coronárias que causam isquemia, anteriormente conhecidas como "doença arterial coronariana" (DAC). A DIC abrange as seguintes condições: DAC (agora denominada de forma mais precisa como doença aterosclerótica coronária), INOCA, angina sem obstrução das artérias coronárias (do inglês *angina with no obstructive coronary artery disease* [ANOCA]), infarto do miocárdio sem obstrução das artérias coronárias (do inglês *myocardial infarction with non-obstructive coronary arteries* [MINOCA]), dissecção espontânea de artéria coronária (DEAC), doença microvascular (DMV), vasoespasmo coronariano (VPC) e embolia/trombose coronária.^564^

A dor torácica é o sintoma fundamental do IAM em ambos os sexos. No entanto, as mulheres são mais propensas a apresentar sintomas considerados atípicos, como dor no dorso e no pescoço, fadiga, náuseas e vômitos. A maioria das mulheres com IAM apresenta sintomas prodrômicos, como falta de ar, fadiga incomum ou desconforto no braço/mandíbula nas semanas que antecedem o evento agudo. Angina estável é a apresentação clínica mais comum em mulheres com DIC, entretanto, as manifestações clínicas específicas das mulheres podem levar a um atraso no diagnóstico da isquemia.^564,565^

Dessa forma, o conceito de DAC obstrutiva como sinônimo de angina e isquemia miocárdica deve ser reconsiderado, uma vez que não se aplica mais devido aos novos conhecimentos sobre a fisiopatologia da isquemia, pois nem sempre há estenose limitando fluxo sanguíneo. Além disso, aproximadamente dois terços das mulheres e ao menos um terço dos homens com isquemia miocárdica não apresentam DAC obstrutiva na angiografia. Nas últimas décadas, houve uma melhor compreensão da fisiopatologia da doença das artérias coronárias, embora vários aspectos ainda não sejam totalmente compreendidos.^566^

As disparidades dependentes do sexo parecem diminuir quando as mulheres atingem a menopausa. A partir da sétima década de vida, são observadas taxas similares de prevalência de DAC e mortalidade por IAM em homens e mulheres. Vários modelos foram estabelecidos para explicar essas descobertas, abrangendo a exposição específica do sexo a fatores de risco cardiovascular tradicionais, fatores específicos das mulheres e determinantes psicossociais da saúde, que têm um impacto maior no sexo feminino.^566^

### 12.1. Características Gerais da Doença Isquêmica do Coração Feminino

#### 12.1.1. Doença Aterosclerótica Coronária^564,565,567d^

A doença aterosclerótica é mais prevalente em mulheres idosas. Nas mulheres, a placa aterosclerótica é menos volumosa e menos calcificada, e a erosão é um mecanismo frequente. A doença aterosclerótica é mais difusa e acomete toda a extensão arterial, associando-se à disfunção microvascular e endotelial.

Prevenção: a prevenção da DAC na mulher, tanto primária quanto secundária, passa pela necessidade de se conhecer as diferenças no quadro clínico da SCA/SCC e suas particularidades no sexo feminino para um diagnóstico e tratamento adequados. Além, claro, da identificação precoce dos fatores de risco cardiovascular (FRCV) clássicos, fatores de risco (FR) específicos da mulher e fatores psicossociais.

Desfechos: a mulher com DAC apresenta maior mortalidade hospitalar, e mulheres mais jovens (< 50 anos) têm mortalidade ainda mais elevada, constituindo um grupo de especial atenção para compreensão dos mecanismos fisiopatológicos.

#### 12.1.2. ANOCA e INOCA (isquemia sem Obstrução das Artérias Coronárias)^564,568^

A INOCA é mais comum em mulheres do que em homens, com prevalência especialmente alta entre as mulheres com idade de 45 a 65 anos.

Prevenção: para uma prevenção efetiva, é necessário identificar e tratar agressivamente os FRCV clássicos e FR específicos da mulher, bem como identificar os fatores potencializadores de risco (fatores psicossociais e determinantes sociais de saúde).

Desfechos: a presença da INOCA na mulher está associada a quadros de angina recorrente, hospitalizações frequentes, coronariografias repetidas e altas taxas de eventos cardiovasculares maiores.

#### 12.1.3. MINOCA (Infarto do Miocárdio sem Obstrução das Artérias Coronárias)^564,569^

O MINOCA está relacionado a mecanismos fisiopatológicos, como vasoespasmo, disfunção microvascular, trombose/embolia, ruptura de placa e dissecção espontânea de artéria coronária. Ele apresenta prevalência de 5 a 10%, considerando-se todos os IAM, sendo que cerca de dois terços dos pacientes apresentam infarto agudo do miocárdio sem supradesnivelamento do segmento ST (IAMSSST).

O MINOCA é mais comum em mulheres mais jovens do que em homens (10,5% vs. 3,4%; p < 0,0001). Os FRCV podem estar presentes, porém são menos frequentes do que em pacientes com DAC.

O diagnóstico de MINOCA é transitório e requer confirmação das várias causas. Faz-se extremamente importante buscar a causa subjacente, visto que a falha na identificação da causa básica pode resultar em tratamento inadequado e informações incorretas.

Prevenção: identificar e tratar os FRCV clássicos e FR específicos da mulher; identificar os fatores potencializadores de risco (fatores psicossociais e determinantes sociais de saúde).

Desfechos: o prognóstico depende da causa subjacente; a morbimortalidade é semelhante à DAC.

#### 12.1.4. Doença Microvascular (DMV)^564,567^

A fisiopatologia resulta do remodelamento estrutural com consequente redução da condutância ou de distúrbios vasomotores afetando as arteríolas, ou de ambos. A confirmação diagnóstica de DMV deve preencher os seguintes critérios: presença de sintomas (angina e/ou dispneia de esforço); ausência de doença obstrutiva; evidência objetiva de isquemia e alteração da função microvascular (defeitos reversíveis, anormalidades nos testes funcionais invasivos – FFR > 0,8; RFC < 2,0 e índice de resistência microvascular [IRM] > 25). Mais recentemente um novo método sem utilização de provas farmacológicas pode inferir a resistência coronária para cada artéria principal, sendo uma maneira que tem sido já utilizada e que, com o tempo, será aprimorada para evitar provocar vasoespasmos, como o teste com acetilcolina intravenoso.

A persistência e/ou recorrência de dor torácica em mulheres representa um dos principais desafios clínicos. Essa condição não apenas afeta o prognóstico das pacientes, como também contribui para o aumento da ansiedade relacionada ao diagnóstico, prejudica a qualidade de vida e resulta em custos elevados para a investigação diagnóstica e tratamento.

Prevenção: modificação dos FRCV (especialmente perda de peso e controle do estresse).

Desfechos: a presença de DMV está relacionada à angina recorrente, hospitalizações frequentes, coronariografias repetidas e altas taxas de eventos cardiovasculares maiores.

#### 12.1.5. Dissecção Espontânea de Artéria Coronária (DEAC)^564,567^

A DEAC é causa rara de IM, representando 1 a 4% de todas as SCA. A DEAC é cada vez mais reconhecida como causa importante de IM em mulheres com menos de 50 anos. Segundo alguns estudos, 25 a 35% dos casos de DEAC ocorrem em mulheres antes dos 50 anos, e 25% em mulheres com mais de 60 anos. É a causa mais comum de IM associado a gravidez (até 43%) ocorrendo principalmente no terceiro trimestre ou no pós-parto. O risco de eventos recorrentes é substancial.^564^

Nessa condição, é comum a ausência de FRCV tradicionais, embora existam FR predisponentes para a dissecção, são eles: displasia fibromuscular (50 a 86%), distúrbios do tecido conjuntivo (5%), doenças inflamatórias sistêmicas (5 a 12%), uso de terapia hormonal (estrogênio, progesterona, gonadotrofina, clomifeno ou tratamento de infertilidade) e múltiplas gestações anteriores.

Prevenção: minimizar os gatilhos emocionais, evitar terapia hormonal e gravidez futura para a prevenção secundária. Recomendar reabilitação cardíaca, preferencialmente com protocolo modificado, evitando exercícios isométricos pesados e atividades aeróbicas intensas.

Desfecho: a recorrência da DEAC é frequente, principalmente no período pós-parto e em portadoras de doenças do tecido conjuntivo.

#### 12.1.6. Vasoespasmo Coronariano (VPC)^564,570^

O VPC é caracterizado pela vasoconstrição reversível, tanto difusa quanto focal, nas artérias coronárias. É uma condição comumente observada em pacientes com DIC e está presente nos mecanismos de INOCA, MINOCA e DMV, independentemente de variações raciais, genéticas e geográficas. A prevalência do VPC é mais elevada em mulheres entre 40 e 70 anos de idade.

O teste provocativo com acetilcolina intracoronária continua sendo a principal ferramenta diagnóstica para reproduzir o VPC e avaliar a reatividade aos nitratos, apesar de não ser amplamente utilizado na prática clínica. É importante notar que as mulheres geralmente requerem doses menores de acetilcolina para desencadear a resposta desejada.

Prevenção: em relação à prevenção, é fundamental evitar fatores agravantes, como o uso de drogas ilícitas, anfetaminas, gás butano, álcool e medicamentos para enxaqueca que possam induzir à vasoconstrição.

Desfechos: quanto aos desfechos, a taxa de recorrência do VPC varia entre 3,9 e 18,6%. Além disso, arritmias cardíacas e morte súbita também podem estar associadas a essa condição.

#### 12.1.7. Trombose/Embolia Coronária^564,571^

A embolia da artéria coronária é considerada um fenômeno raro, com estimativa de incidência em torno de 0,06%. As etiologias da embolia coronária incluem: patologia valvar, cardiomiopatia e FA, com endocardite bacteriana respondendo por cerca de 40 a 53% dos casos.

A embolia de artéria coronária é uma causa subdiagnosticada de SCA, sendo dividida em três tipos: direta, paradoxal (trombo oriundo de trombose venosa profunda que transpõe o forame oval) e iatrogênica. Nessa última, a ICP é a causa mais comum de embolia, e o risco é aumentado com técnicas de rotação, valvuloplastia e anticoagulação inadequada do procedimento.

Prevenção: o melhor método de prevenção é a profilaxia da trombose/ infecção e o diagnóstico precoce das causas subjacentes.

Desfechos: o prognóstico é bom, na maioria dos casos, se o diagnóstico e a trombectomia forem precoces.

Recentemente, uma excelente revisão colocou os dados epidemiológicos mais recentes e os mecanismos fisiopatológicos mais bem elucidados para INOCA e ANOCA e revelou uma prevalência de INOCA em homens quase igual ao de mulheres, chegando a 46% no estudo ISCHEMIA, talvez por os homens serem mais submetidos a exames funcionais de isquemia e, na presença desta, avaliados por exame anatômico.^571,572^

## 13. Doença Isquêmica na Mulher – Aspectos Específicos do Sexo Feminino

A cardiomiopatia isquêmica em mulheres engloba um espectro de complexidades influenciadas por fatores fisiológicos, hormonais e clínicos distintos dos homens. Abordar essas diferenças por meio de pesquisas específicas de gênero e abordagens terapêuticas personalizadas é imperativo para melhorar os resultados nas mulheres. Uma melhor compreensão dessas disparidades levará a melhores estratégias diagnósticas, prognósticas e terapêuticas, em última análise, preenchendo a lacuna no cuidado cardiovascular entre os gêneros.^565^

As mulheres tendem a ter pior prognóstico após o IAM. Elas apresentam maiores taxas de reospitalização e mortalidade em comparação aos homens, com valores permanecendo elevados em 1 ano (26% vs. 19%) e 5 anos (47% vs. 36%) pós-IAM. A IC sintomática pós-IAM é mais prevalente em mulheres mais velhas, com o risco aumentado estendendo-se além do episódio inicial.^573^

Apesar de apresentarem melhor FEVE e menor carga de DAC obstrutiva, mulheres com cardiomiopatia isquêmica (CMI) relatam menor capacidade funcional e qualidade de vida, embora suas taxas de mortalidade sejam comparáveis às dos homens.^574^ A hipertensão arterial e o diabetes mellitus contribuem de forma mais significativa para o risco de IC em mulheres, apresentando um padrão fenotípico distinto.^575^

### 13.1. Apresentação Clínica e Diagnóstico

As mulheres frequentemente apresentam mais IC sintomática, apresentando dispneia e ortopneia com mais frequência do que os homens. A obesidade, mais prevalente entre mulheres com CMI, correlaciona-se com pior prognóstico em relação aos homens.^575^

As modalidades diagnósticas não invasivas desempenham um papel crucial na avaliação da CMI em mulheres. Técnicas como angiografia e RMC demonstram alta sensibilidade e desempenho, auxiliando na identificação de lesões hemodinamicamente significativas e fornecendo informações sobre etiologias não isquêmicas. A seleção de modalidades diagnósticas deve considerar fatores específicos de gênero e condições especiais, como gravidez.^576^

### 13.2. Tratamento e Manejo

As disparidades de tratamento persistem entre os sexos. As mulheres são menos propensas a receber terapias oportunas e direcionadas por diretrizes para o IAM, contribuindo para desfechos desfavoráveis. Os homens são mais prontamente encaminhados para tratamento e recebem terapias direcionadas por diretrizes com maior frequência.^565^

Avaliações invasivas, como a cineangiocoronariografia, permanecem essenciais, particularmente em casos graves de cardiomiopatia ou quando a incerteza diagnóstica persiste apesar dos métodos não invasivos. Apesar dos avanços, as mulheres estão sub-representadas nos ensaios clínicos, limitando a otimização de estratégias terapêuticas para elas.^577^

O impacto dos hormônios sexuais e fatores epigenéticos na remodelação cardíaca em condições isquêmicas é digno de nota. As mitocôndrias femininas demonstram melhor tolerância à privação de oxigênio e ao dano oxidativo do que as mitocôndrias masculinas, potencialmente devido aos efeitos protetores do estrogênio. O estrogênio também pode reduzir os níveis de cálcio antes da isquemia, atenuando a lesão de isquemia-reperfusão em mulheres.^575^

### 13.3. Fatores Prognósticos e Desfechos

Vários fatores, incluindo a extensão da lesão isquêmica e a presença de comorbidades, como hipertensão e diabetes, influenciam o prognóstico de mulheres com CMI. As mulheres apresentam um padrão distinto de IC com fração de ejeção preservada (ICFEP), que é mais comum entre elas, em comparação com a etiologia isquêmica, manifestando-se como cardiomiopatia dilatada com fração de ejeção reduzida em homens.^578^

Mulheres com MCI também enfrentam maiores riscos de complicações pós-revascularização e transplante cardíaco. Elas apresentam taxas mais altas de complicações, como rejeição, mas tendem a ter melhores taxas de sobrevida pós-transplante em comparação com os homens. Entretanto, o uso de enxertos arteriais na revascularização do miocárdio é menos frequente em mulheres, impactando negativamente os resultados pós-operatórios.^578^

Apesar das discussões em curso sobre as disparidades de gênero no cuidado cardiovascular, as mulheres permanecem menos incluídas nos ensaios clínicos, afetando a generalização dos achados da pesquisa para populações femininas. A participação limitada das mulheres em estudos clínicos ressalta a necessidade de pesquisas mais inclusivas para otimizar os protocolos de tratamento e melhorar os resultados para mulheres com MCI.^579^

### 13.4. Influência Hormonal e Fatores Genéticos

As variações hormonais, particularmente o estrogênio, desempenham um papel protetor contra o estresse oxidativo e a apoptose, que é crucial na lesão isquêmica. Os níveis elevados de cálcio, que exacerbam a lesão de isquemia-reperfusão, são modulados pelo estrogênio, reduzindo os danos nas mulheres.^578^

Além disso, cromossomos sexuais e mecanismos epigenéticos contribuem para diferenças de gênero na remodelação cardíaca. Genes relacionados a processos adversos de remodelação cardíaca, como ativação de macrófagos e metabolismo lipídico, estão localizados no cromossomo X, influenciando diferentemente os desfechos em homens e mulheres.^580^

## 14. Angina Refratária

É uma condição crônica definida pela presença de angina limitante, ou seu equivalente, com duração > 3 meses, em decorrência isquemia miocárdica na presença de DAC, não controlada pelo tratamento clínico otimizado, incluindo a combinação de drogas antianginosas toleradas em indivíduos inelegíveis à revascularização miocárdica.^581,582^

A prevalência da AR é estimada em 5 a 15% da população com DAC.^583^ A incidência de eventos combinados e de morte dessa população, de acordo com dados brasileiros,^584^ é de 7,7 e 4,4% em 1 ano, respectivamente, e de 24,4 e 13,5% em 5 anos.

Os pacientes com AR apresentam um importante prejuízo da qualidade de vida,^585,586^ sendo esse o foco do tratamento. Centros multiprofissionais dedicados e com experiência em AR promovem maior eficácia do tratamento e melhora da qualidade de vida, e o referenciamento dos pacientes deve ser considerado.^586–588^

Existem opções promissoras no tratamento da AR (Tabela 39), algumas ainda experimentais. Os resultados de segurança e eficácia dos estudos avaliando as diversas terapias são variáveis. No Brasil, até o momento, algumas opções estão disponíveis para aplicação clínica (Tabela 40).

**Tabela 39:** Terapias alternativas na angina refratária

Terapias alternativas
**1. Reabilitação cardiovascular baseada em exercício** A terapia adjuvante não invasiva de baixo custo promove melhora da capacidade funcional e sintomas anginosos^589,590^ e pode ser considerada como opção terapêutica na AR,^504,591^ desde que realizada uma adequada avaliação pré-participação do paciente e de forma assistida, em ambiente hospitalar.
**2. Neuromodulação** A neuroestimulação medular apresenta resultados de eficácia variáveis; pode melhorar os sintomas anginosos e a qualidade de vida dos pacientes, podendo ser considerada opção terapêutica na AR na falência de outras terapias.^592–595^ A neuromodulação subcutânea e transcutânea e a simpatectomia não apresentam resultados de eficácia consistentes que corroborem sua indicação na AR.^596,597^
**3. Recanalização percutânea de oclusão total crônica** As taxas de sucesso têm aumentado de forma considerável, podendo alcançar até 90%, com baixas taxas de complicação.^598,599^ Promove melhora da angina e da qualidade de vida,^600–602^ sendo uma opção terapêutica para o tratamento da AR em centros experientes.
**4.** **Terapia por ondas de choque** Terapia não invasiva utilizando ondas de choque de baixa energia, estimulando a angiogênese no miocárdio isquêmico Promove melhora da perfusão miocárdica, entretanto, não foi demonstrada redução consistente de sintomas anginosos.^593–605^ Dessa forma, não existe evidência suficiente para ser considerada uma opção terapêutica na AR.
**5. Acupuntura** Promove o controle dos episódios de angina, a redução do consumo de nitrato sublingual, o aumento do tempo de exercício, além de melhora do nível de ansiedade e depressão.^606–608^ Pode ser considerada como opção terapêutica no tratamento da AR, em centros com experiência em medicina chinesa tradicional.
**6.** **Redutor do seio coronário** Técnica percutânea de implante de dispositivo em forma de ampulheta que cria uma estenose no seio coronário, aumentando a pressão no seio coronariano e, consequentemente, a perfusão do miocárdio isquêmico no território coronariano esquerdo. Promove melhora dos sintomas anginosos, da qualidade de vida e do limiar isquêmico.609 Ainda não está disponível no Brasil.
**7. Contrapulsação externa facilitada** Técnica que utiliza a insuflação diastólica de manguitos nos membros inferiores do paciente, coordenada pela sincronização eletrocardiográfica, aumentando a pressão arterial e o fluxo sanguíneo aórtico retrógrado e, consequentemente, o enchimento coronariano. Reduz episódios anginosos e melhora o limiar de isquemia, sendo terapia bem tolerada apesar de efeitos adversos não limitantes.^610^ Não está disponível no Brasil.
**8. Terapia celular e gênica** Terapias experimentais com resultados iniciais promissores na melhora sintomática.^611,612^
**9. Aférese de Lp(a)** Promove melhora da angina, qualidade de vida, tempo de exercício e melhora perfusional do miocárdio.613 Terapia não disponível no Brasil.
**10. Transplante cardíaco** Permanece como última opção terapêutica na AR, especialmente para pacientes com função ventricular preservada, visto que a taxa de mortalidade após transplante cardíaco no Brasil permanece elevada, podendo chegar até 30% em 1 ano.^614^
**11. Revascularização transmiocárdica a laser** Técnica que utiliza ablação a laser para criar canais transmurais no miocárdio isquêmico, a fim de provocar neovascularização e melhorar sintomas anginosos. Não existe evidência estabelecida de eficácia no tratamento sintomático,^615^ com elevado risco de complicações relacionadas ao procedimento, estando contraindicada no tratamento da AR.

AR: angina refratária.

**Tabela 40:** Recomendações para o Tratamento da Angina Refratária

Recomendação	Grau de recomendação	Nível de evidência
Recanalização percutânea de oclusão total crônica	**IIa**	**A**
Reabilitação cardiovascular baseada em exercício	**IIb**	**B**
Neuroestimulação medular	**IIb**	**C**
Acupuntura	**IIb**	**A**
Transplante cardíaco	**IIb**	**C**
Revascularização trasmiocárdica a laser	**III**	**A**

## 15. Viabilidade Miocárdica

Viabilidade miocárdica significa músculo vivo, portanto, viável. A revascularização de segmentos miocárdicos disfuncionantes, porém viáveis, poderia levar à melhora da contratilidade e função ventricular. A revascularização desses segmentos poderia reduzir a chance de eventos futuros, independente da melhora da contratilidade. Extensas áreas de fibrose miocárdica são improváveis de recuperarem contratilidade. Apesar de esses fundamentos terem plausibilidade biológica, existe muito pouca evidência de que a revascularização miocárdica melhore a função ventricular ou reduza desfechos clínicos.

Por outro lado, o tratamento farmacológico está definitivamente associado com a redução de desfechos clínicos nessa população. O subestudo de viabilidade STICH^616^ e o HEART (*Heart Failure Revascularization*)^344^ não conseguiram demonstrar que a avaliação de viabilidade miocárdica identifica pacientes portadores de miocardiopatia isquêmica que irão se beneficiar da revascularização cirúrgica do miocárdio. O recente estudo REVIVED–BCI2 (*Revascularization for Ischemic Ventricular Dysfunction – British Cardiovascular Society-2*)^143^ também falhou na associação entre viabilidade miocárdica em segmentos disfuncionantes e redução de desfechos com a ICP. Esses achados têm feito clínicos, cirurgiões, hemodinamicistas e diretrizes refletirem sobre qual seria o papel da viabilidade na seleção de pacientes para procedimentos intervencionistas.

### 15.1. Viabilidade Miocárdica

Viabilidade significa simplesmente miócitos sem dano irreversível. A viabilidade se fundamenta justamente no conceito de miocárdio hibernado, em que a disfunção é decorrente de um mecanismo de adaptação e sobrevivência dos cardiomiócitos submetidos a episódios recorrentes de isquemia. Essa definição é o conceito de miocárdio hibernante, um processo adaptativo de "*down-regulation*" da função miocárdica, favorecendo a sobrevida do miócito devido à redução no aporte de oxigênio.

O miocárdio hibernante existe em um espectro clínico de disfunção causado por isquemia, que inclui: 1) atordoamento miocárdico, causado por isquemia aguda e redução da contratilidade durante período de hipoperfusão, sem causar morte celular. Após o tratamento da obstrução coronariana, a disfunção segmentar permanece por horas a dias com resolução espontânea; 2) o miocárdio hibernante apresenta comprometimento prolongado da contratilidade, secundário a uma lesão obstrutiva fixa associada a episódios recorrentes de isquemia não letal; e 3) fibrose, causada por um período prolongado de isquemia, resultando em necrose celular completa e substituição de tecido normal por fibrose.^618^ A recuperação da contratilidade é altamente improvável.

### 15.2. Estudos Observacionais de Viabilidade

A hibernação representa disfunção contrátil reversível, e a revascularização levaria a uma melhora na função ventricular e, portanto, melhora da sobrevida. Essa suposição, que tem um racional biológico, foi observada em uma série de estudos retrospectivos observacionais.^619^

Esses estudos foram todos retrospectivos, em geral, em centros únicos, com enorme *bias* de seleção, em que os pacientes com melhor prognóstico e menor risco eram direcionados para o tratamento cirúrgico, enquanto pacientes de pior prognóstico e maior risco eram mantidos em tratamento clínico. Esses estudos, ainda, foram realizados antes da terapia farmacológica atual, tanto para o tratamento de IC quanto da doença coronariana.

### 15.3. Viabilidade e Melhora da Função Ventricular

Existem diferentes metodologias para avaliação da viabilidade miocárdica. Esses testes são validados na detecção de viabilidade e reversão da disfunção ventricular após revascularização. A melhora da contratilidade pode ser imediata com a revascularização ou ocorrer dentro de horas a dias ou mesmo meses, de acordo com o grau de acometimento, do miocárdio atordoado ao hibernante avançado. Cada metodologia de deteção de viabilidade utiliza diferentes aspectos da fisiopatologia da hibernação e produz medidas quantitativas e/ou qualitativas de viabilidade na predição da recuperação funcional.^620,621^

Vários estudos observacionais corroboram que testes de detecção de viabilidade predizem melhora da função segmentar e global do VE.^619^ Uma metanálise de 158 estudos demonstrou que todas as modalidades apresentam acurácia na detecção de viabilidade.^95^ Vários estudos demonstram uma interação de quantidade de tecido viável e probabilidade de recuperação da contratilidade regional. Deve ser ressaltado que o efeito da revascularização não pode ser dissociado do efeito farmacológico, uma vez que a viabilidade identifica o substrato miocárdico que pode melhorar em resposta a uma série de intervenções, não apenas a revascularização.^622^

### 15.4. Estudos Contemporâneos: STICH e REVIVED–BCIS2

Diferente dos estudos prévios, retrospectivos e com *bias* de seleção de pacientes, os estudos contemporâneos STICH e REVIVED-BCIS2 são estudos prospectivos e randomizados, nos quais não houve *bias* de seleção de pacientes com menor risco para procedimentos invasivos e maior risco mantidos em tratamento clínico. Adicionalmente, foram realizados na vigência de terapia médica otimizada, tanto para DAC como para ICfer.

O estudo STICH envolveu 1.212 pacientes com disfunção, no qual Velazquez et al.^623^ demonstraram que a CRM associada ao TMO evolui com uma menor taxa de mortalidade, redução de morte por todas as causas, morte cardiovascular e morte ou hospitalização por causas cardíacas, sendo considerada indicação Classe I-B pela ESC 2018 e American Heart Association (AHA)/American College of Cardiology (ACC), independente da pesquisa de viabilidade miocárdica.

O subestudo de viabilidade do STICH,^624^ com 601 pacientes, identificou como viáveis 81% dos pacientes. Pacientes com viabilidade miocárdica apresentaram menores taxas de mortalidade em relação aos sem viabilidade miocárdica (HR 0,64; IC 95% 0,48–0,86; p = 0,003). Entretanto, após ajuste de outras variáveis significativas em um modelo multivariado, o *status* pré-especificado de viabilidade não foi mais significativo (p = 0,21).

Na análise de 10 anos (23) desses 601 pacientes incluídos no estudo de viabilidade, não foi evidenciada associação entre viabilidade miocárdica e melhor sobrevida com a CRM. A viabilidade foi associada a melhora discreta da FEVE, tanto no tratamento cirúrgico como no tratamento médico isolado, mas essa melhora não foi relacionada a melhor sobrevida.

A ausência de correlação entre viabilidade miocárdica e menor mortalidade com a CRM indica que a estratégia de avaliação de viabilidade miocárdica não deve ser a única quando na tomada de decisão da estratégia de tratamento. Esse achado foi diferente dos resultados encontrados nos estudos iniciais de viabilidade miocárdica.

O estudo REVIVED-BCIS2^615^ foi o primeiro que se dedicou a avaliar o papel da ICP em pacientes com disfunção ventricular e presença de viabilidade, sendo um estudo prospectivo, multicêntrico, aberto e randomizado. Os pacientes eram randomizados para ICP ou TMO isolado. Foram incluídos 700 pacientes com FEVE ≤ 35% e presença de viabilidade em ≥ 4 segmentos disfuncionantes, passíveis de revascularização pela ICP. Pacientes com infarto até 4 semanas antes da randomização foram excluídos. O desfecho primário era composto de morte e internação por IC, e o secundário por melhora da fração de ejeção e qualidade de vida. Após um seguimento mediano de 41 meses, não houve diferença na ocorrência do desfecho primário entre os grupos angioplastia vs. tratamento clínico otimizado (37,2% vs. 38,0%, respectivamente; HR 0,99; IC 95% 0,78–1,27; p = 0,96). Também não houve diferença entre os grupos na FEVE e na qualidade de vida em 2 anos.

Adicionalmente, foi identificado que a fibrose, avaliada pela técnica de realce tardio com o gadolínio e quantificada por ressonância miocárdica, está associada a pior prognóstico nesses pacientes, nos quais, a cada 10% de aumento na fibrose, houve um aumento de 18% do risco de eventos combinados (HR 1,18; IC 95% 1,04–1,33; p = 0,009). Além disso, a quantidade de fibrose foi também associada à probabilidade de recuperação da FEVE com razão de chances (*odds ratio* [OR]): 0,69 (0,56–0,84). Outro achado interessante foi a associação da melhora da fração de ejeção com a sobrevida, em que os pacientes que tiveram uma melhora ≥ 4,7% na fração de ejeção tiveram menor incidência de eventos combinados (morte e internação por IC) (HR 0,62; IC 95% 0,41–0,95; p = 0,029).

A quantidade de miocárdio viável também não foi associada à redução do desfecho primário ou melhora da FEVE com o procedimento de revascularização.^625^

Outra importante análise foi o impacto da revascularização completa anatômica ou guiada por viabilidade miocárdica,^626^ sendo que o procedimento de revascularização percutânea também não foi associado com melhora da sobrevida livre de eventos.

O estudo REVIVED-BCIS2 incluiu apenas pacientes com viabilidade miocárdica, portanto, de menor risco. Havia menor quantidade de pacientes com lesões acometendo três artérias em relação ao estudo STICH, portanto, também de menor risco. Entretanto, no estudo REVIVED, os pacientes apresentavam uma média de idade de 70 anos e incluíram pacientes com TCE que eram excluídos em outros estudos de ICP.

Esse resultado confirma os achados já encontrados no estudo STICH. A hipótese de que, em pacientes com insuficiência cardíaca com fração de ejeção reduzida e recebendo terapia médica recomendada pelas diretrizes, a revascularização de segmentos miocárdicos viáveis com disfunção contrátil não foi associada a uma melhor sobrevida livre de eventos, independentemente da quantidade de tecido viável e revascularização completa.

### 15.5. Terapia Farmacológica na DAC e ICfer

A terapia farmacológica baseada em evidências inclui fármacos para o tratamento tanto da DAC quanto para a ICfer.

No estudo STICH, ainda não estavam disponíveis os i-SGLT2 e os inibidores de neprilisina e do receptor da angiotensina (*angiotensin receptor neprilysin inhibitors* [ARNIs]), os quais já estavam disponíveis por ocasião do estudo REVIVED. Tecidos viáveis disfuncionantes podem apresentar melhora da contratilidade segmentar quando em uso de betabloqueadores. Esses fármacos podem melhorar a função de tecidos miocárdicos viáveis pela redução do consumo de oxigênio e aumento da perfusão diastólica, conforme demonstrado no estudo CHRISTMAS (*Carvedilol Hibernation Reversible Ischaemia Trial, Marker of Success*).^727^

A terapia médica otimizada em pacientes com DAC e ICfer isoladamente são associadas com redução da mortalidade. Uma subanálise do estudo STICH, por Farsky et al.,^628^ avaliou o efeito da terapia médica otimizada da DAC e ICfer na evolução dos pacientes com cardiomiopatia isquêmica grave, passível de CRM. Na randomização e em 4 meses, 58,7 e 73,3% dos pacientes estavam recebendo terapia médica otimizada, respectivamente. Em uma análise multivariada pelo modelo de Cox, o uso de terapia médica otimizada na randomização foi associado com uma menor mortalidade por todas as causas (HR 0,78; IC95% 0,66–0,91; p = 0,04). Resultado independente da randomização para tratamento clínico ou CRM, o tratamento médico foi associado com menor mortalidade total.

A presença de viabilidade miocárdica não deve ser considerada como pré-requisito para decisão da estratégia terapêutica a ser indicada, conforme corrobora o estudo STICH, no qual o benefício da revascularização cirúrgica foi independente da presença de viabilidade. Em relação à ICP na cardiomiopatia isquêmica, o estudo REVIDED-BCIS2 não conseguiu demonstrar benefício na sobrevida quando comparado ao TMO isolado, mesmo em pacientes com extensa viabilidade miocárdica.

Uma metanálise recentemente publicada^629^ considerou os quatro principais estudos randomizados que avaliaram viabilidade na cardiomiopatia isquêmica: STICH, PARR-2 (*Positron Emission Tomography and Recovery Following Revascularization Phase 2*), HEART e REVIVED-BCIS2. Não houve diferença significativa em mortalidade entre os grupos (OR 0,92; IC95% 0,75–1,14), com uma taxa de eventos semelhante no longo prazo 34% vs. 36%, respectivamente.

### 15.6. Resumo

- Pacientes portadores de DAC com FEVE ≤ 35% passíveis de cirurgia de revascularização devem ser encaminhados prontamente para a CRM;
- O benefício da CRM ocorre independentemente da presença de viabilidade;
- A ICP não reduziu morte/internação por IC;
- A presença de viabilidade miocárdica leva a melhor prognóstico e melhora da FEVE com TMO otimizado isolado ou associado à cirurgia;
- A quantidade de fibrose prediz pior prognóstico e menor probabilidade de recuperação do VE.

## 16. Doença Renal Crônica na SCC

Pacientes com DRC constituem um dos grupos de maior risco cardiovascular, com taxas de mortalidade mais de 10 vezes maiores do que aquelas encontradas na população geral. Entre as complicações cardiovasculares mais frequentes, destaca-se a DAC. Estima-se que 30 a 70% dos pacientes renais apresentam evidência angiográfica de DAC, mas, paradoxalmente, esses pacientes costumam ser assintomáticos. A DAC é uma das principais causas de eventos cardiovasculares em todos os estágios da evolução da DRC e permanece relevante mesmo após um transplante renal bem-sucedido. Por esses motivos, todo indivíduo com taxa de filtração glomerular comprometida ou proteinúria deve ser considerado como de risco de DAC elevado. Existem poucas evidências que orientem a estratificação de risco coronariano, porque as equações de Framingham subestimam o risco de pacientes renais, e a DRC é, não raramente, considerada um critério de exclusão nos estudos utilizados na construção das diretrizes. Consequentemente, na maioria das vezes, as recomendações baseiam-se em níveis de evidência C.

Nesta seção, estamos nos referindo a pacientes sem dor torácica típica. Pacientes com angina devem ser abordados de acordo com as diretrizes desenvolvidas para a população geral.^630^ O objetivo é fornecer ao cardiologista os meios de identificar os indivíduos que devem ser investigados, discutir o tipo de exames indicados e levantar alguns pontos do tratamento em uma população de alto risco frequentemente assintomática e excluída dos estudos aleatorizados. Além de recorrer aos dados recentes da literatura, utilizamos observações originárias da coorte KiHeart,^631^ desenvolvida no nosso centro, que engloba cerca de 3.000 pacientes com DRC estágio 5 acompanhados por um período de tempo máximo e 25 anos. Considerando a complexidade da DRC, é recomendável utilizar uma abordagem multidisciplinar na tomada de decisões diagnósticas e terapêuticas (Tabelas 41 e 42).

**Tabela 41:** Estratégia de avaliação e tratamento

Recomendação	Grau de recomendação	Nível de evidência
Recomenda-se que a tomada de decisão diagnóstica e definição de estratégia terapêutica sejam discutidas por um "Heart-Kidney Team", incluindo cardiologista clínico, cardiologista intervencionista, cirurgião cardiovascular e nefrologista.	**I**	**C**

**Tabela 42:** Estratificação clínica de risco

Recomendação	Grau de recomendação	Nível de evidência
Pacientes com DRC estágios 3 a 5 devem ser estratificados para DAC utilizando critérios clínicos (fatores de risco) independentemente de sintomas e nível funcional.	**I**	**A**

DAC: doença arterial coronariana; DRC: doença renal crônica.

A estratificação clínica é importante por permitir identificar os pacientes que precisam ser investigados para DAC, além de ser eficaz e de custo reduzido. A American Society of Transplantation^632^ recomenda estratificar pacientes estágio 5 utilizando apenas três fatores: idade ≥ 50 anos, diabete e DCV concomitante. Na população geral, outras características, como sexo masculino, raça branca, tabagismo e dislipidemia são fatores de risco significativos para DAC. A importância desses fatores diminui à medida que DRC progride, de tal forma que deixam de ser relevantes em pacientes em diálise.

**Figura 21:**
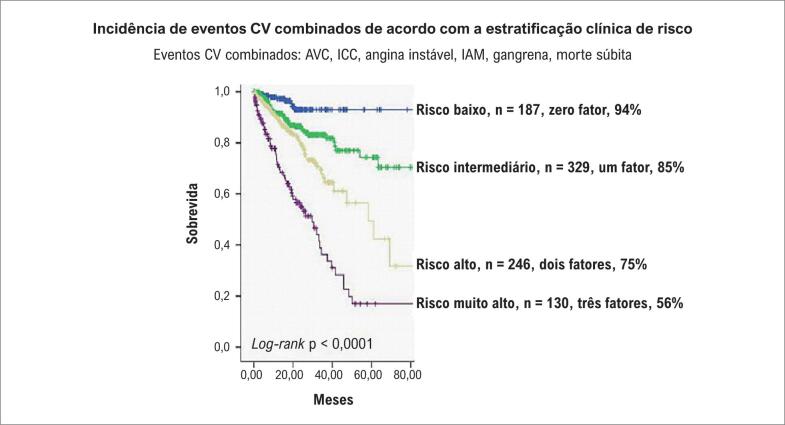
Sobrevida livre de eventos cardiovasculares combinados de acordo com o número de fatores de risco. AVC: acidente vascular cerebral; CV: cardiovascular; IAM: infarto agudo do miocárdio; ICC: insuficiência cardíaca congestiva.

Um ponto de importância prática dessa estratificação clínica é que a sobrevida livre de eventos cardiovasculares diminui em função do número dos três fatores, como indicado na Figura 21.^633^

Fonte: De Lima, Gowdak & de Paula, 2012.

Observa-se que pacientes sem nenhum dos três fatores apresentam sobrevida livre de eventos cardiovascular superior a 90% em 80 meses de acompanhamento. A sobrevida cai para próximo a 50% nos indivíduos com os três fatores (Tabela 43).

**Tabela 43:** Recomendações de Estratificação de Risco para Doença Arterial Coronariana com Base em Fatores Clínicos

Recomendação	Grau de recomendação	Nível de evidência
Indivíduos sem nenhum dos três fatores de risco principais (idade ≥ 50 anos, diabete, DCV) são considerados de baixo risco e não necessitam de investigação aprofundada para DAC.	**I**	**C**
Pacientes com um ou mais de um dos fatores de risco importantes devem ser estratificados para DAC usando métodos não invasivos e invasivos.	**I**	**C**

DAC: doença arterial coronariana; DCV: doença cardiovascular.

### 16.1. Estratificação por Testes Não Invasivos

Na população geral, as diretrizes de uso corrente não recomendam avaliação coronariana aprofundada em indivíduos assintomáticos com boa capacidade funcional, mesmos naqueles candidatos a cirurgias não cardíacas de médio ou grande porte.^630,634^ Por outro lado, a diretriz da NKF KDOQI (National Kidney Foundation Kidney Disease Outcomes Quality Initiative),^635^ concebida para indivíduos com DRC, avalia que pacientes diabéticos e/ou com DAC, mesmo assintomáticos, devem ser submetidos rotineiramente a testes não invasivos para isquemia.

Os testes não invasivos têm acurácia reduzida na DRC. O mais indicado é a cintilografia miocárdica com estresse farmacológico. Existem testes não invasivos de imagem que podem ajudar na definição de risco.^636^ A angioTC coronária é útil em pacientes renais crônicos, mas seu uso é limitado pelo risco de nefrotoxicidade naqueles ainda não em diálise e pelo alto grau de calcificação coronária que dificulta a visualização do lume. Exames baseados em gadolínio são contraindicados em pacientes com DRC avançada.

Como acontece com outros testes dessa natureza, a cintilografia tem valor limitado em indivíduos de risco muito alto, como é o caso de boa parte dos pacientes com DRC, e é desnecessária quando o risco clínico é baixo.

### 16.2. Estratificação por Testes Invasivos

**Figura 22:**
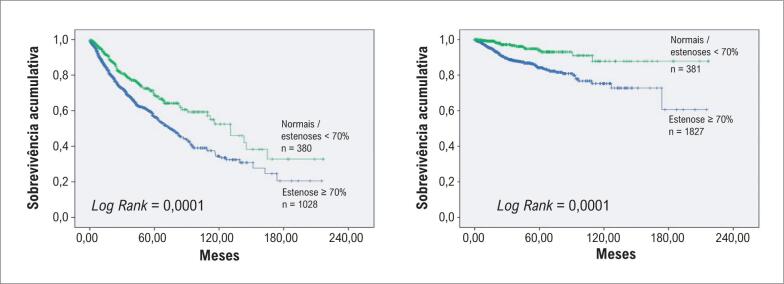
Sobrevida livre de eventos cardiovasculares combinados (esquerda) e infarto do miocárdio (direita) em pacientes com DRC estágio 5 submetidos à cinecoronariografia (coorte KiHeart). DRC: doença renal crônica.

A cinecoronariografia define a anatomia dos vasos e requer a utilização de meio de contraste. Seu uso é problemático em indivíduos ainda não em diálise. A DAC, diagnosticada por angiografia, tem uma forte associação com prognóstico em pacientes em diálise (Figura 22 e Tabela 44).

**Tabela 44:** Recomendações para a Estratificação por Testes Invasivos

Recomendação	Grau de recomendação	Nível de evidência
Pacientes com teste não invasivo positivo e aqueles de alto risco clínico devem ser encaminhados ao estudo invasivo.	**IIb**	**C**
Pacientes assintomáticos com doença coronariana obstrutiva quanto a indicar revascularização: não devem ser encaminhados para revascularização miocárdica de rotina pela possibilidade de serem submetidos ao transplante ("intervenção profilática"), a menos que haja inequívoco impacto prognóstico da intervenção.	**III**	**C**

### 16.3. Crítica à Estratégia Invasiva de Investigação da DAC

Considerando a elevada prevalência de aterosclerose, a baixa acurácia dos testes não invasivos e a forte associação da DAC com eventos, conclui-se que pacientes com teste positivo ou múltiplos fatores de risco devem ser investigados invasivamente e tratados por revascularização, se indicado. Por outro lado, não existe evidência definitiva de efeito benéfico da revascularização miocárdica (percutânea ou cirúrgica) em pacientes renais com DAC assintomática.^637^ De fato, os resultados do recente estudo ISCHEMIA^638^ sugerem que a avaliação aprofundada da doença coronariana na DRC avançada não altera o prognóstico. Esse estudo avaliou o possível benefício de uma estratégia invasiva, que incluiu cinecoronariografia e intervenção, se indicados, contra TMO em indivíduos com DRC estágios 4 e 5 com isquemia documentada de média ou grande extensão. Após 2,2 anos de observação, não se observou diferença na incidência de eventos cardiovasculares importantes entre os grupos. No entanto, após o seguimento mais longo de 5,7 anos, a estratégia invasiva foi associada com uma redução significativa na mortalidade cardiovascular que foi, no entanto, contrabalançada por aumento na mortalidade não cardiovascular. Portanto, a necessidade de investigação rotineira da DAC na doença renal avançada não está definida, e os resultados do ISCHEMIA precisam ser ainda confirmados. Finalmente, é importante ressaltar que a possibilidade de indicação de transplante renal por si só não deve ser levada em consideração quando escolhemos o tratamento da DAC a ser indicado: intervenção ou farmacológico.^639^

Resumindo, a estratégia invasiva é útil para avaliar o risco de eventos cardiovasculares, mas não influencia necessariamente o prognóstico.

### 16.4. Medicação e Ajuste de Dose em Pacientes com DCV e DRC

A DRC confere um grau de risco de eventos não inferior ao associado ao IAM, justificando a opção pela prevenção secundária.^640,641^ Isso significa prescrever estatinas, aspirina, betabloqueador e iECA/bloqueador de receptor de angiotensina (BRA) para todos os pacientes, salvo se houver contraindicação.

A DRC afeta a absorção, biodisponibilidade, metabolismo e eliminação de muitas drogas. O uso inapropriado da medicação e erros na dosagem contribuem para o aumento da toxicidade e a redução da eficácia do tratamento. O Quadro 3 mostra alguns medicamentos utilizados em pacientes com cardiopatias e DRC não dialítica e as doses recomendadas levando em conta a taxa de filtração glomerular.

Os iECA/BRA são fármacos de primeira linha no tratamento da hipertensão e proteinúria e retardam a progressão da DRC. A elevação da creatinina sérica em até 15% é um efeito esperado, e não é motivo para a redução da dosagem. É aconselhável suspender essas drogas quando a elevação for > 30% em comparação ao basal. Atenolol e outros betabloqueadores hidrofílicos (bisoprolol, nadolol) são eliminados pelos rins, e as doses devem ser reduzidas. Tal não acontece com propranolol e metoprolol, que são metabolizados pelo fígado e podem ser usados em dose plena.

Em pacientes com DRC avançada, a hidroclorotiazida é ineficaz, e a espironolactona deve ser evitada. Metformina é contraindicada em pacientes com DRC estágio ≥ 3 devido ao risco de acidose láctica. A gliclazida e outras sulfonilureias devem ser usadas com cautela, sendo a primeira preferível, uma vez que tem meia vida mais curta e não origina metabólitos ativos. Os inibidores do cotransporte Na-glicose, como a dapagliflozina, são, em geral, considerados seguros na DRC, mas os dados em pacientes com filtração glomerular < 30 mL/min ainda não são suficientes para definir conduta. As estatinas não requerem ajuste de dosagem, mas sua utilidade na prevenção primária de eventos na DRC é controvertida, especialmente em pacientes em diálise.^642–644^ A KDIGO Organization^645^ não recomenda o uso rotineiro de estatinas para pacientes em diálise assintomáticos e sem outras evidências de DAC. As ACC/AHA deixam indefinida a indicação de estatinas.^646^ O AAS é incerto em pacientes renais na prevenção primária.^647^ A varfarina e a apixabana são os anticoagulantes orais indicados para pacientes com função renal.^648^

Para pacientes com DRC estágios 3 a 5, as recomendações abaixo são as indicadas (Tabela 45).

**Tabela 45:** Recomendações para Medicação e Ajuste de Dose em Pacientes com DCV e DRC

Recomendação	Grau de recomendação	Nível de evidência
Recomenda-se que a tomada de decisão diagnóstica e a definição de estratégia terapêutica sejam discutidas por um "Heart-Kidney Team" incluindo cardiologista clínico, cardiologista intervencionista, cirurgião cardiovascular e nefrologista.	**I**	**C**
Pacientes com DRC estágios 3 a 5 devem ser avaliados quanto à presença e gravidade da doença coronariana com base na história clínica, exame físico e exames rotineiros.	**I**	**A**
Pacientes sem os fatores de risco principais* são considerados de baixo risco cardiovascular e não necessitam de investigação complementar. Pacientes com um ou mais fatores de risco devem ser estratificados para DAC usando métodos não invasivos e invasivos.	**I**	**C**
Pacientes com teste não invasivo positivo e os que apresentem múltiplos fatores de risco são considerados de alto risco coronariano e deverão ser encaminhados para estudo invasivo.	**IIb**	**C**
Pacientes com DAC obstrutiva envolvendo os segmentos proximais das artérias coronárias epicárdicas principais têm indicação de revascularização miocárdica cirúrgica ou percutânea visando a redução do risco CV.	**IIb**	**C**
Pacientes assintomáticos com doença coronariana obstrutiva não devem ser encaminhados para revascularização miocárdica de rotina com base na possibilidade de serem submetidos ao transplante ("intervenção profilática"), a menos que haja inequívoco impacto prognóstico da intervenção.	**III**	**C**
Pacientes com DRC estágios 3 a 5 devem ser tratados para prevenção secundária de eventos.	**IIa**	**C**

CV: cardiovascular; DAC: doença arterial coronariana; DRC: doença renal crônica.

* Idade ≥ 50 anos, diabetes mellitus e evidência de doença cardiovascular.

## 17. Aspectos Cardio-Oncológicos na SCC

A oncologia desafia a medicina por englobar pacientes em diferentes estágios de evolução de uma mesma doença e por impor a necessidade de respostas rápidas no que tange à tomada de decisão. Por essas razões, pacientes com neoplasia ativa são comumente excluídos dos grandes estudos clínicos. Além disso, é cada vez mais frequente a coexistência de DCVs em pacientes com câncer, visto que compartilham fatores de risco em comum.^649,560^ Disfunção endotelial, estresse oxidativo, hipercoagulabilidade e inflamação crônica são usuais nesse perfil de pacientes e o tratamento instituído, muitas vezes, contribui para o desenvolvimento da DCV.^649–651^ A ocorrência de DAC em pacientes com câncer é uma realidade, e esse número só cresce,^649,652^ tanto no contexto de quem já tem DAC e recebe o diagnóstico de câncer quanto em quem enfrenta os efeitos adversos na saúde cardiovascular advindos da radioterapia no tórax e da quimioterapia.^649,651,652^ As neoplasias aumentam, de forma independente, o risco de morte cardiovascular e de sangramento maior.^650^

A abordagem terapêutica nos pacientes com DAC inclui a definição de intervenção coronariana e o uso de antiplaquetário.^653,654^ Em pacientes oncológicos e com DAC, algumas variáveis devem ser analisadas antes da tomada dessa decisão, conforme a Tabela 46.^653^ O alto risco de sangramento em alguns cenários tornou o manejo clínico e a definição de conduta ainda mais complexos.^655^ Na Tabela 47, estão alguns elementos que podem aumentar o risco de sangramento.

**Tabela 46:** Variáveis para a tomada de decisão

**Necessidade de cirurgia oncológica precoce**
**Risco de sangramento aumentado**
**Alterações laboratoriais como disfunção renal, anemia e coagulopatias**
**Expectativa de vida**

**Tabela 47:** Fatores que aumentam o risco de sangramento

**Local do tumor** Exemplo: tumores do trato gastrointestinal, próstata
**Local da cirurgia** Exemplo: neurocirurgia, ressecção transuretral de próstata
**Discrasia sanguínea pelo tipo de tumor ou extensão da doença** Exemplo: plaquetopenia, acometimento hepático

As evidências científicas ajudam a amparar e conduzir esses pacientes, conforme a Tabela 48.

**Tabela 48:** Recomendações Cardio-Oncológicas para Terapia Intervencionista

Recomendação	Grau de recomendação	Nível de evidência
A decisão por intervenção deve basear-se no tipo de tumor, expectativa de vida, risco de sangramento e urgência do procedimento cirúrgico.^652^	**I**	**C**
Ao indicar intervenção em paciente frágil, o procedimento deve ser o menos invasivo possível.^652^	**I**	**C**
A indicação da intervenção deve ser compartilhada entre cardiologista e oncologista.	**I**	**C**
O stent farmacológico deve ser preferível em pacientes oncológicos.^651^	**IIa**	**B**
A DAPT pode ser mantida em pacientes com plaquetopenia > 30 mil, se não houver contraindicação.^651^	**IIa**	**B**

DAPT: dupla antiagregação plaquetária.

Há um aumento claro nas angioplastias coronarianas em pacientes com câncer em atividade e, ainda mais, nos pacientes com histórico prévio da doença.^650^ Pacientes oncológicos têm taxas maiores de IAM, trombose de stent, sangramento e necessidade de novas revascularizações quando comparados aos demais.^656^ Segundo Guo et al., os pacientes com câncer sob maior risco de eventos trombóticos e isquêmicos após angioplastia poderiam ser identificados através dos altos valores do escore DAPT.^656^

Nos mais de 6 milhões de pacientes submetidos a angioplastia nos Estados Unidos, segundo Potts et al., as quatro neoplasias mais comumente encontradas foram: próstata, mama, cólon e pulmão.^650^ Nessa publicação, o câncer de pulmão esteve associado a maior risco de complicações intra-hospitalares, incluindo duas vezes mais óbito, enquanto o atual diagnóstico de câncer de cólon esteve associado, de forma independente, a um maior risco de sangramento.^650^ O câncer ativo de próstata associou-se somente a maior risco de sangramento, ao passo que o câncer de mama não demonstrou associação significativa com nenhuma complicação intra-hospitalar. A presença de metástase, independentemente do tipo de câncer, também impactou as taxas de morte e complicações intra-hospitalares.^650^ Com exceção ao câncer de pulmão, o histórico prévio de neoplasia não influenciou o risco de eventos adversos em pacientes submetidos à angioplastia coronariana.^650^ A propósito, os eventos hemorrágicos maiores, bem como a mortalidade intra-hospitalar, foram maiores nos pacientes que receberam stent convencional em comparação ao stent farmacológico.^650^

Com o avanço da tecnologia e com o maior acesso aos stents farmacológicos, o tempo de terapia antiplaquetária tem sido reduzido consideravelmente ao longo dos últimos anos.^653^ Até 2015, indicava-se angioplastia com stent convencional para pacientes com alto risco de sangramento, visto que essa era a conduta mais segura para reduzir tempo de dupla terapia antitrombótica (DAPT) especialmente nos pacientes em pré-operatório de cirurgia não cardíaca.^653^ Contudo, estudos com novas plataformas de stent mostram que é possível atenuar o risco de sangramento sem comprometer a segurança de curto prazo com DAPT reduzida em alguns cenários.^657^ O estudo MASTER DAPT (*The Management of High Bleeding Risk Patients Post Bioresorbable Polymer Coated Stent Implantation with an Abbreviated versus Standard DAPT Regimen*) comparou DAPT por 30 dias vs. 90 dias após implante de stent eluído com sirolimus. Entre os mais de 4 mil pacientes estudados, o tempo reduzido de tratamento foi não inferior à terapia padrão.^657^ Resultados semelhantes foram vistos nos estudos GLOBAL LEADERS (*Ticagrelor plus aspirin for 1 month, followed by ticagrelor monotherapy for 23 months vs aspirin plus clopidogrel or ticagrelor for 12 months, followed by aspirin monotherapy for 12 months after implantation of a drug-eluting stent: a multicentre, open-label, randomised superiority trial*)^658^ e STOP DAPT 2 (*Short and Optimal Duration of Dual Antiplatelet Therapy After Everolimus-Eluting CobaltChromium Stent*),^659^ no cenário de pacientes com baixo risco de sangramento.

Com a publicação do estudo ZEUS, em 2016, o uso do stent farmacológico mostrou ser superior ao stent convencional para redução dos desfechos com uso de DAPT por apenas 30 dias em pacientes de alto risco de sangramento.^660^ Um ano antes, a publicação do LEADERS FREE (*Polymer-free Drug-Coated Coronary Stents in Patients at High Bleeding Risk*) já havia fomentado esse novo conceito. Com quase 2.500 pacientes randomizados, esse trabalho mostrou que o stent sem polímero (BioFreedom) foi superior ao stent convencional nessa população.^661^ O estudo SENIOR (*Drug-eluting stents in elderly patients with coronary artery disease: a randomised single-blind trial*) mostrou que encurtar a duração da DAPT em pacientes idosos submetidos à angioplastia com stent bioabsorvível foi a melhor opção ao se comparar com o implante de stent convencional e DAPT prolongada.^662^

Mais recentemente, foram publicados dados do ONYX ONE (*Polymer-based or Polymer-free Stents in Patients at High Bleeding Risk*), o primeiro trabalho que comparou stent eluído com zotarolimus vs. stent sem polímero para estratégia de DAPT por 30 dias em pacientes de alto risco de sangramento.^663^ Quase 2 mil pacientes foram avaliados, e o stent farmacológico mostrou ser não inferior ao stent sem polímero em termos de segurança e eficácia no seguimento de 1 ano.^663^ Stents mais seguros e mais eficazes, aliados à imagem intravascular, propiciam um cenário mais estável para a tomada de decisão nos quase 30% de pacientes com alto risco de sangramento que são submetidos à angioplastia.^663^

A avaliação da ocorrência de eventos isquêmicos e hemorrágicos em pacientes com câncer foi comparada a outros pacientes com alto risco de sangramento. Campos e colaboradores analisaram, pela primeira vez, dados de quatro grandes estudos que utilizaram stents farmacológicos, DAPT de curta duração e pacientes de alto risco para sangramento. Cerca de 10% da população avaliada era oncológica e apresentou maiores taxas de sangramento e mortalidade por todas as causas. No entanto, não houve aumento no risco de infarto, trombose de stent ou revascularização adicional. Curiosamente, a maior taxa de sangramento nos pacientes com câncer ocorreu após a descontinuação da DAPT.^415^

Aceitar o desafio de atender pacientes oncológicos envolve o esforço de minimizar o risco de sangramento às custas de pouca trombose. Os riscos e benefícios a longo prazo, bem como a análise dos diferentes tipos de stents farmacológicos e a aplicação dos escores de risco ainda precisam ser mais bem esclarecidos nessa população. Mas com base no julgamento clínico e na individualização de cada caso, parece ser seguro usar a DAPT por tempo reduzido após o implante de stent farmacológico ou stent sem polímero na população com alto risco de sangramento, incluindo pacientes com diagnóstico atual de câncer.^661,663,664^

## 18. Tratamento da Hipertensão Arterial no Portador de Doença Arterial Coronariana Crônica

A hipertensão arterial é o principal fator de risco modificável para DAC.^1,80,507^ No estudo INTERHEART,^665^ 25% dos IAM foram atribuídos à hipertensão arterial. A metanálise que avaliou o impacto da redução da pressão arterial sobre o risco cardiovascular demonstrou que, para cada 10 mmHg de redução da PAS, essa queda é acompanhada de uma redução de 17% de DAC.^666^

O tratamento da hipertensão arterial associada a DAC inclui pacientes pós-IAM, com angina de peito e pós-revascularização miocárdica, portanto, classificados como hipertensos de alto risco cardiovascular.^507^ Dessa forma, a meta de pressão arterial a ser atingida deve considerar a possibilidade de efeito da curva J, demonstrado em diferentes estudos.^338,667^ O objetivo é alcançar uma PAS < 130 mmHg e pressão arterial diastólica (PAD) < 80 mmHg (Grau de recomendação: IIa; Nível de evidência: B), devendo-se evitar níveis abaixo de 120/70 mmHg.^1,80,507^

A recomendação de tratamento para hipertensos portadores de DAC envolve adoção de hábitos de vida saudáveis e tratamento medicamentoso com combinação de dois fármacos de forma imediata. Devendo-se considerar, preferencialmente, os inibidores do sistema renina angiotensina aldosterona (iSRAA; iECA ou BRA) em combinação com betabloqueadores ou BCCs de longa duração.^1,80,507^ Particularmente no cenário pós-IAM, a combinação de iSRAA e betabloqueadores representa o benefício de redução da pressão arterial, efeito antianginoso, redução de eventos e de mortalidade, especialmente no período até 2 anos após o evento agudo.^668,669^ Em pacientes com angina sintomática, os betabloqueadores e os BCCs podem ser igualmente escolhidos para a estratégia de tratamento medicamentoso combinado por serem eficientes na redução dos sintomas.^1,80,507^

Quando a meta de pressão arterial não for alcançada com a combinação dupla, deve-se progredir para combinação tripla, introduzindo um diurético tiazídico (preferencialmente de longa duração). Havendo necessidade de um quarto fármaco, o inibidor mineralocorticoide (espironolactona) é a classe de escolha e, subsequentemente, os agonistas alfa-2 adrenérgicos (Tabela 49).^1,80,507^

**Tabela 49:** Resumo das recomendações para tratamento anti-hipertensivo no portador de DCC^1,80,507^

Recomendação	Classe de indicação	Nível de evidência
Em adultos com DAC crônica, são recomendadas estratégias não farmacológicas como terapia de primeira linha para reduzir a PA em todos os estágios de hipertensão e níveis de risco cardiovascular.	**I**	**A**
As metas de PA a serem alcançadas nos portadores de DAC crônica são: Adultos: PAS 120–129 mmHg/PAD 70-79 mmHg; Idoso hígido: PAS 130–139 mmHg/70-79 mmHg; Idoso frágil: PAS 140–149 mmHg/70-79 mmHg.	**I**	**A**
Hipertensos com história de IAM recente (< 12 meses) têm indicação para uso de betabloqueadores e inibidores do sistema renina angiotensina aldosterona como primeira linha de tratamento.	**I**	**A**
Em pacientes com sintomas anginosos, betabloqueadores e/ou bloqueadores de canal de cálcio di-hidropiridínicos de longa duração são classes terapêuticas recomendadas.	**I**	**A**
Outros medicamentos anti-hipertensivos adicionais, como bloqueadores de canais de cálcio di-hidropiridínicos de longa duração, diuréticos tiazídicos de ação prolongada, antagonistas dos receptores mineralocorticoides e/ou agonistas alfa-2 adrenérgicos podem ser adicionados conforme necessário para alcance da meta de PA.	**I**	**A**
O uso concomitante de iECA e BRA é contraindicado por não apresentar benefícios e aumentar os riscos de eventos adversos.	**III**	**A**

BRA: bloqueador de receptor de angiotensina; DAC: doença arterial coronariana; IAM: infarto agudo do miocárdio; iECA: inibidores da enzima conversora de angiotensina; PA: pressão arterial; PAD: pressão arterial diastólica; PAS: pressão arterial sistólica.

## 19. Aspectos do Tratamento na Presença da Fibrilação Atrial

### 19.1. Novos Anticoagulantes Orais Associados ao Ácido Acetilsalicílico

Nesse cenário de alta carga isquêmica, alguns ensaios clínicos de larga escala avaliaram a associação de outras drogas com ação na cascata de coagulação em associação aos antiagregantes plaquetários, especialmente o AAS. No estudo COMPASS,^670^ pacientes elegíveis com doença arterial de membros inferiores, carótidas ou DAC, sem indicação prévia de anticoagulação, foram randomizados para rivaroxabana oral em baixa dose (2,5 mg duas vezes ao dia, ¼ da dose para anticoagulação plena) mais aspirina (100 mg), rivaroxabana duas vezes ao dia (5 mg com placebo de AAS) ou AAS uma vez ao dia. A rivaroxabana em baixa dose reduziu eventos adversos cardiovasculares e de membros adversos graves em comparação com o AAS, às custas de aumento de sangramentos maiores. Os limiares de significância pré-especificados para mortalidade, entretanto, não foram alcançados para toda a população, mas reduções absolutas de risco foram observadas para indivíduos de mais alto risco, como diabéticos, portadores de DAP ou DRC e tabagistas ativos.

Esse conceito sobre o benefício da associação de rivaroxabana e AAS nos pacientes de risco mais elevado foi avaliado adicionalmente em uma subanálise do COMPASS, que demonstrou uma melhora significativa da qualidade de vida, medida através do teste de caminhada de 6 minutos nos indivíduos recebendo terapia dupla, em comparação ao AAS isoladamente, sem eventos hemorrágicos observados.^671^ Dessa forma, considera-se adequada a recomendação da associação de uma segunda droga anti-isquêmica, incluindo a rivaroxabana em baixas doses, para pacientes com SCCs e elevada carga isquêmica, levando-se em conta, na decisão, o risco hemorrágico individual.

### 19.2. Anticoagulação em Indivíduos com Síndromes Coronarianas Crônicas e Fibrilação Atrial

De forma geral, a anticoagulação plena é recomendada para pacientes com FA e SCCs para a redução de eventos embólicos, sobretudo o AVC isquêmico, havendo documentada superioridade sobre a monoterapia com aspirina ou terapia antiplaquetária dupla baseada em clopidogrel.^672^ Quando existe a possibilidade de prescrição de um novo anticoagulante oral (apixabana, dabigatrana, edoxabana ou rivaroxabana), em vez de um antagonista da vitamina K, essas drogas são preferíveis, sobretudo em termos de segurança em relação a eventos hemorrágicos.^672,673^

Em relação aos pacientes com indicação eletiva de ICP, até o momento, nenhum grande ensaio clínico a avaliou especificamente em pacientes com SCCs e FA. Dessa forma, as decisões devem ser baseadas em estudos que incluíram uma grande proporção ou exclusivamente pacientes com SCAs. Nesse cenário, no estudo AUGUSTUS, pacientes com FA e histórico recente de SCA ou ICP e indicação de um inibidor P2Y12 foram randomizados entre apixabana (5 mg duas vezes ao dia) ou um antagonista da vitamina K, além de AAS ou placebo correspondente por 6 meses. O esquema com apixabana sem AAS resultou em menos sangramentos e hospitalizações, sem diferenças significativas na incidência de eventos isquêmicos em comparação com o antagonista da vitamina K, aspirina ou ambos.^210^ Os resultados foram consistentes no subgrupo mais grave, de pacientes com histórico de eventos cerebrovasculares prévios.^674^

Tais achados foram confirmados em uma metanálise de rede que incluiu cinco estudos e mais de 11.000 pacientes (a maioria pós-SCA ou intervenção coronária) com FA submetidos a ICP: um regime com novo anticoagulante oral não antagonista da vitamina K + inibidor P2Y12 foi associado a uma redução de complicações hemorrágicas da ordem de 48%, incluindo sangramento intracraniano, sem diferença significativa em eventos isquêmicos, em comparação com antagonista da vitamina K + terapia antiplaquetária dupla.^675^

Dessa forma, em pacientes com FA e SCA ou ICP recentes, a terapia com anticoagulante oral não antagonista da vitamina K na dose plena apropriada para prevenção de AVC mais um inibidor de P2Y12 sem aspirina deve ser a primeira opção de tratamento, e regimes com antagonista da vitamina K mais dupla antiagregação com inibidor de P2Y12 e AAS devem ser geralmente evitados e restritos ao 1° mês em pacientes com risco trombótico muito elevado e que não possam receber anticoagulantes orais diretos. Se o risco de trombose de stent e outros eventos isquêmicos for alto, o uso de aspirina por 30 dias parece razoável. No período crônico, após 12 meses do evento, o anticoagulante oral isoladamente deve ser a abordagem padrão (Tabelas 50 e 51).^1,675^

**Tabela 50:** Terapia antitrombótica em pacientes com síndromes coronarianas crônicas e fibrilação atrial

Recomendação	Grau de recomendação	Nível de evidência
Quando há indicação de anticoagulação oral em paciente com FA elegível para NOAC, o NOAC deve ser recomendado preferencialmente em vez de um antagonista da vitamina K.	**I**	**A**
A anticoagulação oral de longo prazo (NOAC ou antagonista da vitamina K) é recomendada em pacientes com FA e escore CHA2DS2-VASC ≥ 2 no sexo masculino e ≥ 3 no feminino.	**I**	**A**
A anticoagulação oral de longo prazo (NOAC ou antagonista da vitamina K) pode ser considerada para pacientes com FA e escore CHA2DS2-VASC ≥ 1 no sexo masculino e ≥ 2 no feminino.	**IIa**	**B**

FA: fibrilação atrial; NOAC: novo anticoagulante oral (*novel oral anticoagulante*).

**Tabela 51:** Terapia antitrombótica em pacientes pós-angioplastia coronariana e com fibrilação atrial ou outra indicação de anticoagulação

Recomendação	Grau de recomendação	Nível de evidência
AAS e clopidogrel devem ser administrados pré-procedimento para pacientes submetidos a implante de stent coronário eletivo.	**I**	**C**
Em pacientes com indicação de anticoagulação com NOAC, recomenda-se que a medicação (dabigatrana 150 mg duas vezes ao dia, rivaroxabana 20 mg/dia, apixabana 5 mg duas vezes ao dia ou edoxabana 60 mg/dia) deva ser usada preferencialmente em vez de AVK em combinação com terapia antiplaquetária.	**I**	**A**
Doses ajustadas de rivaroxabana, dabigatrana, edoxabana ou apixabana podem ser utilizadas conforme recomendações atuais em bula, estritamente de acordo com os critérios clínicos definidos na bula e juntamente com terapia antiplaquetária concomitante simples ou dupla.	**IIa**	**B**
Após ICP sem complicações ou fatores associados à trombose de stent, a suspensão precoce (< 1 semana) do AAS e a continuação da terapia dupla com anticoagulante oral e clopidogrel devem ser consideradas em indivíduos com baixo risco de trombose ou se o risco de sangramento prevalecer sobre o de trombose de stent, independentemente do tipo de stent.	**IIa**	**B**
A terapia tripla com AAS, clopidogrel e anticoagulante oral por 1 mês deve ser considerada quando o risco de trombose de stent superar o risco de sangramento.	**IIa**	**C**
A terapia dupla com anticoagulante oral e ticagrelor pode ser considerada como alternativa à terapia tripla com anticoagulante oral, AAS e clopidogrel em pacientes com risco moderado a alto de trombose de stent, independentemente do tipo de stent ou em doentes com conhecida resistência ao clopidogrel.	**IIa**	**B**
Ticagrelor ou prasugrel não são recomendados como parte da terapia antitrombótica tripla com AAS e qualquer anticoagulante oral.	**IIb**	**C**
A terapia dupla ou tripla com anticoagulante oral e prasugrel com ou sem aspirina não é recomendada como alternativa à terapia tripla com anticoagulante oral, AAS e clopidogrel em pacientes com risco moderado a alto de trombose de stent, independentemente do tipo de stent.	**III**	**A**

AAS: ácido acetilsalicílico; AVK: antagonistas da vitamina K; ICP: intervenção coronária percutânea; NOAC: novo anticoagulante oral (*novel oral anticoagulante*).

## 20. Prevenção Secundária PÓS-SCA

Indivíduos com histórico de eventos cardiovasculares agudos prévios apresentam risco cinco vezes maior de ter recorrência do evento em comparação com indivíduos sem DCV conhecida. Ademais, pacientes com DAC clinicamente estabelecida estão em risco muito alto de eventos cardiovasculares recorrentes. Após um episódio de SCA, a adesão ao tratamento antitrombótico e a outras terapias medicamentosas (bloqueadores do sistema renina-angiotensina-aldosterona, betabloqueadores e estatinas) é prioritária.^1,676^

Após alta por um episódio de SCA, os pacientes devem ser idealmente direcionados para linhas de cuidado apropriados ao seu nível de risco individual, garantindo, assim, um manejo adequado. Medidas não farmacológicas com foco em hábitos de vida e reabilitação devem ser priorizadas na fase imediata pós-evento, e deve-se investir, junto ao paciente, na adesão adequada aos programas de prevenção secundária, para os quais protocolos de manejo compartilhado na fase pós-aguda são condições indispensáveis.^676^

### 20.1. Prioridades no Período Pós-Alta por SCA

A alta é marco fundamental para o manejo do paciente com SCA. Nessa etapa, os profissionais responsáveis pelos cuidados primários desempenham papel importante na orientação educacional pós-SCA, como parte da estratégia geral de controle de fatores de risco. Estratégias de aconselhamento devem ser aplicadas aos pacientes pós-SCA para garantir impactos positivos em desfechos de saúde e qualidade de vida dos indivíduos e suas famílias.

Sumariamente, recomenda-se que uma equipe multidisciplinar desenvolva protocolos de alta customizados para cada paciente, abrangendo a hospitalização, medicações de alta e ações a serem tomadas em caso de mudanças do quadro clínico. Esse plano deve coordenar o planejamento dos cuidados de seguimento e os canais de contato com o provedor de cuidados.^1,676^ Educação em saúde, reabilitação cardíaca e seguimento clínico oportuno são os componentes essenciais do protocolo de alta.^676^

### 20.2. Estratificação de Risco pós-SCA para Planejamento da Prevenção Secundária

Ferramentas de estratificação para predição de risco de MACE a longo prazo incluem escores de risco validados como o SMART (*Secondary Manifestations of Arterial Disease*) e o modelo de risco EUROASPIRE (*European Action on Secondary and Primary Prevention by Intervention to Reduce Events*).^677,678^

O escore SMART, desenvolvido na Holanda, em uma população de 5.788 indivíduos com DCV prévia, e validado externamente em três coortes internacionais, estima o risco de 10 anos para IAM, AVC ou morte cardiovascular. As variáveis incluídas no modelo de risco SMART são idade, sexo, tabagismo atual, diabetes, pressão arterial, colesterol, DAC, doença cerebrovascular, doença arterial periférica, creatinina e valores de proteína C-reativa de alta sensibilidade.^677^

Já a calculadora EUROASPIRE, derivada em uma população aleatória de 8.000 indivíduos com seguimento mediano de 1,7 ano e validada externamente em 4.484 pacientes, estima o risco de 2 anos de recorrência de eventos cardiovasculares em pacientes com DAC estável.^678^ A calculadora avalia a associação entre fatores de risco e a incidência do desfecho primário do escore, composto de evento cardiovascular fatal ou novas hospitalizações por IAM não fatal, AVC, IC, CRM ou ICP.^678^

Outros escores de risco, como o GRACE (*Global Registry of Acute Coronary Events*), o ProACS e outros já demonstraram acurácia similar e também podem ser aplicados.^679^ Dessa forma, deve ser realizada a estratificação de risco para MACE após um episódio de SCA através de escores validados para todos os pacientes admitidos com essa condição e o mais brevemente possível no período hospitalar.^676^

### 20.3. Variáveis de Risco para Insuficiência Cardíaca e Eventos Trombóticos

A disfunção ventricular esquerda e a presença de sinais ou sintomas de IC representam as variáveis mais adequadas para realizar uma estratificação prognóstica e delinear linhas de cuidado específicas pós-alta por SCA.^680^ O desenvolvimento de IC durante a hospitalização e precocemente após a alta são importantes preditores de mortalidade durante o seguimento.

Nesse contexto, as variáveis clínicas associadas a alto risco de IC e/ou disfunção ventricular esquerda após um episódio de SCA, a partir de estudos envolvendo essas populações, são:^676^

1. Classe de Killip durante o evento;
2. FEVE < 40%;
3. FEVE entre 40 e 45% associada a padrão de enchimento diastólico restritivo, regurgitação mitral significativa, índice do escore de mobilidade parietal elevado e VE não dilatado;
4. Variação importante do peptídeo natriurético do tipo B;
5. Necessidade de diuréticos de alça.

Revisões da literatura demonstraram que a reabilitação cardíaca se associa a uma significativa melhora dos desfechos dessa subpopulação, incluindo a redução do risco de IAM, discreta redução na mortalidade por todas as causas e significativa redução de hospitalização por todas as causas, resultando em custos significativamente menores para os sistemas de saúde e na melhora da qualidade de vida em 12 meses de seguimento.^681,682^ A longo prazo, há uma tendência no sentido de redução das taxas de mortalidade cardiovascular e IAM.^682^ Apesar das evidências desses benefícios, registros internacionais como o EUROASPIRE V estimam taxas de participação em tais programas de reabilitação inferiores a 35%.^678^

De forma similar, pacientes com alto risco isquêmico a partir de variáveis clínicas e angiográficas têm elevadas taxas de recorrência de eventos cardiovasculares adversos, especialmente trombóticos. A ocorrência prévia de um episódio de SCA, por si só, coloca o indivíduo nesse estrato de risco. Para esses pacientes, devem ser fornecidos seguimento e reabilitação cardíaca ambulatorial através contatos e consultas frequentes, especialmente no 1° ano pós-evento, além de programas adequados ao quadro clínico e às variáveis de risco associadas.^681–683^

Essas características estão associadas a alto risco trombótico a partir de dados de estudos envolvendo indivíduos com DAC documentada e devem ser levadas em conta para a definição de estratégias mais agressivas de prevenção secundária:^666,676,784,688–694^

1. Doença arterial periférica;
2. Histórico de angina ou IAM prévios;
3. Doença coronariana multivascular;
4. Revascularização incompleta;
5. Pacientes não revascularizados em abordagens prévias.

### 20.4. Estratégias para Pacientes de Baixo Risco

Pacientes sem IC e/ou disfunção ventricular esquerda e sem fatores para alto risco trombótico podem ser encaminhados para linhas de cuidado e programas de reabilitação de menor intensidade, com uma única consulta cardíaca especializada no 1° ano e encaminhamento subsequente para a atenção primária. Indicações para prevenção secundária também devem ser detalhadamente fornecidas no relatório de alta, através de aconselhamento dedicado, com possibilidade de acompanhamento remoto. Dessa forma, a avaliação sistemática do risco de IC/disfunção ventricular esquerda e de eventos trombóticos deve ser provida a todos os pacientes pós-SCA.^676,679^

### 20.5. Seguimento Clínico

Após a revascularização miocárdica e/ou após a estabilização clínica pós-SCA (< 1 ano), os pacientes devem ser rigorosamente monitorados devido ao risco de recorrência de eventos isquêmicos. Evidências atuais sobre manejo das SCCs recomendam reavaliação da função ventricular esquerda 8 a 12 semanas após um procedimento de revascularização para se acessar a evolução da função miocárdica (atordoamento ou hibernação) ou possível deterioração devido a DCVs ou condições clínicas concomitantes.^1,676^ Um ano após a revascularização, mesmo que o paciente esteja assintomático, é necessária uma avaliação anual com a realização de um ECG de 12 derivações para avaliar o estado clínico, a adesão à medicação e o cumprimento das metas dos fatores de risco cardiovascular.

Um teste de estresse não invasivo para avaliar a isquemia silenciosa e uma ecocardiografia transtorácica podem ser realizados a cada 3 a 5 anos.^695^ Testes laboratoriais (perfil lipídico, função renal, hemograma completo, perfil glicêmico, biomarcadores etc.) devem ser reavaliados periodicamente, de acordo com as metas e resultados obtidos.^676,695^ Ainda nesse contexto, a reavaliação remota através de telemedicina, quando possível, também se mostrou eficaz em aumentar a interação médico-paciente, reduzindo desfechos como nova hospitalização, procura por pronto-atendimento, revascularização não programada e controle de sintomas.^696^

## 21. Cuidados Pós-Intervenção Coronária Percutânea na SCC

### 21.1. Antiagregação Plaquetária após ICP com Stent em Paciente sem Indicação de Uso de Anticoagulante

A DAPT com AAS e inibidor do receptor P2Y12 (IP2Y12) após implante de stent e a terapia padrão são utilizados com a finalidade de reduzir o risco de trombose e eventos isquêmicos.^697–700^ O AAS é o antiplaquetário preferencial, devendo ser prescrito após implante de stent, exceto em casos de intolerância ou eventos adversos.^1,700,701^ O clopidogrel é o IP2Y12 de escolha em pacientes submetidos a implante de *stent* na SCC.^702,703^ O estudo randomizado ALPHEUS (*Ticagrelor versus clopidogrel in elective percutaneous coronary intervention*) comparou se o ticagrelor era superior ao clopidogrel após a ICP com maior complexidade em pacientes com DAC estável. Foram randomizados 1.910 pacientes, não havendo diferença quanto ao desfecho primário, definido como o composto de IM tipo 4 (a ou b) relacionado a ICP ou lesão miocárdica grave entre os grupos. No grupo ticagrelor, houve um aumento de sangramento menor aos 30 dias.^704^

O tempo ideal de DAPT na DAC estável, com a utilização dos stents farmacológicos, vem se reduzindo no decorrer dos anos. Vários estudos têm demonstrado que a DAPT por 6 meses, quando comparada a 1 ano, tem se mostrado eficaz e segura.^1,657,700–714^ Contudo, uma duração mais curta de DAPT, por 3 meses, poderá ser considerada naqueles com alto risco de sangramento.^1,657,700,701,710–714^ Alguns estudos têm avaliado o uso da monoterapia com um IP2Y12 após um breve período de DAPT.^715^ O estudo japonês STOP DAPT-2 avaliou o uso da DAPT por 1 mês em paciente submetidos a implante de stent de cromo-cobalto com eluição de everolimo, seguido por monoterapia com uso do clopidogrel, evidenciando diminuição de eventos hemorrágicos sem aumento significativo de eventos isquêmicos.^659^ O estudo TWILIGHT randomizou 7.119 pacientes submetidos a ICP com stent após 3 meses de DAPT, sendo 35% no cenário de SCC; o uso do ticagrelor isolado em comparação com o ticagrelor mais aspirina teve uma menor incidência de sangramento clinicamente relevante sem maior risco de morte, IAM ou AVC.^208^

A utilização da DAPT por mais de 6 meses, chegando aos 30 meses, pode ser considerada nos casos de risco isquêmico elevado e baixo risco hemorrágico.^716–718^ O escore DAPT, que pode ser utilizado para essa tomada decisão, foi criado a partir de uma coorte do DAPT STUDY (*Dual Antiplatelet Therapy Study*), pacientes com escore de risco alto (≥ 2) apresentam redução de desfechos isquêmicos à custa de um pequeno aumento no risco de sangramento com DAPT por 30 meses. Em contrapartida, nos pacientes com escore baixo (< 2), não se evidenciou redução em eventos isquêmicos.^414^

**Figura 23:**
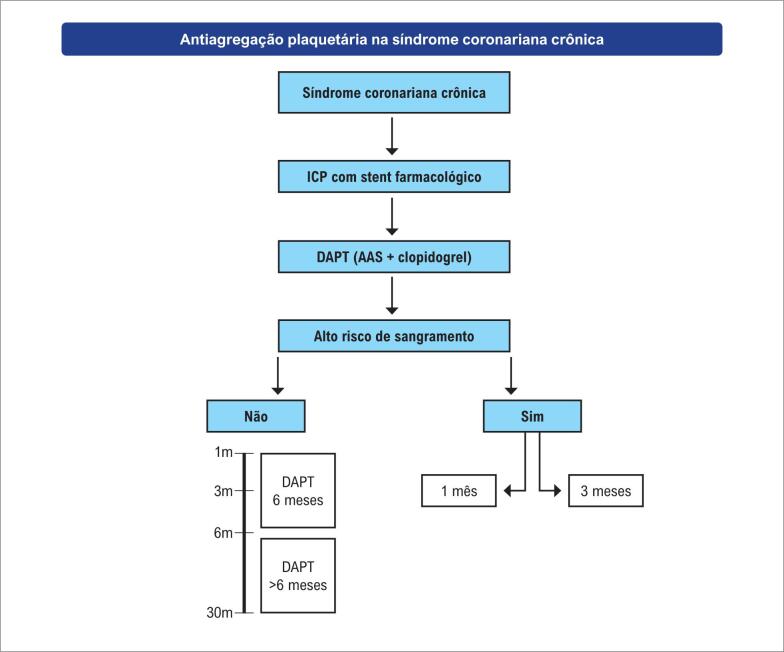
Antiagregação plaquetária após ICP na síndrome coronariana crônica. AAS: ácido acetilsalicílico; DAPT: dupla antiagregação plaquetária; ICP: intervenção coronária percutânea

O estudo COMPASS^204,719^ demonstrou que a inibição de via dupla (IVD) com rivaroxabana 2,5 mg duas vezes ao dia associada a AAS 100 mg uma vez ao dia, quando comparada a AAS 100 mg isoladamente, reduziu o desfecho primário de evento cardiovasculares maiores (ECAM) constituído de morte cardiovascular, IAM ou AVC, bem como mortalidade, em pacientes com SCC e/ou doença arterial periférica. Em uma análise de subgrupo desse estudo (COMPASS-PCI), foram avaliados os resultados de pacientes com SCC com ou sem ICP prévia, que foram tratados com IVD vs. apenas AAS. Dos 27.395 pacientes no COMPASS, houve 16.560 pacientes com SCC e, desses, 9.862 (59,6%) tiveram ICP prévia. O tempo médio de ICP até a randomização foi de 5,4 anos, com tempo médio de acompanhamento de 1,98 ano. Naqueles com ICP prévia, a IVD em comparação com AAS produziu reduções consistentes em ECAM, independentemente do tempo da ICP, à custa de aumento de sangramento (Figura 23).^720^

### 21.2. Antiagregação Plaquetária após ICP com Stent em Paciente com Indicação de Uso de Anticoagulação

**Figura 24:**
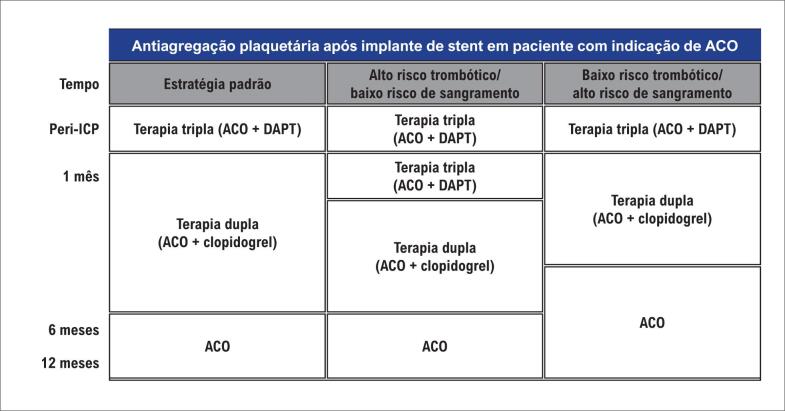
Antiagregação plaquetária após ICP em pacientes em uso de ACO. ACO: anticoagulação oral; DAPT: dupla antiagregação plaquetária; ICP: intervenção coronária percutânea.

Muito pacientes que são submetidos a ICP com stent na SCC têm FA com indicação de anticoagulação oral (ACO) cronicamente. A combinação de ACO e DAPT aumenta significativamente o risco de sangramento.^721–723^ Nessa situação, foi avaliada a melhor combinação da terapia antitrombótica e por quanto tempo em diversos estudos clínicos. Estudos randomizados demonstraram que o uso dos anticoagulantes orais diretos (*direct oral anticoagulants* [DOACs]) são mais seguros que o uso da varfarina, no cenário da ICP, sendo o ACO preferencial (Figura 24).^724–726^

O primeiro estudo randomizado a avaliar o uso do DOAC após ICP com stent foi o PIONEER AF-PCI. O uso da rivaroxabana em baixas doses com um IP2Y12 por 12 meses foi associado a uma menor taxa de sangramento clinicamente significativo do que a terapia padrão com um antagonista da vitamina K, não havendo diferença quanto aos desfechos isquêmicos.^727^ O RE-DUAL PCI também mostrou o benefício da dabigatrana nesse cenário.^728^

O estudo AUGUSTUS foi fundamental para esclarecer o papel do AAS após a ICP em pacientes em uso de ACO. Foram randomizados 4.614 pacientes, em 33 países, num desenho fatorial 2 x 2, sendo incluídos pacientes com FA no cenário de SCA e/ou submetidos a ICP com stent. Os pacientes foram randomizados para receber apixabana ou um antagonista da vitamina K, eles eram novamente randomizados para receber AAS ou placebo associados a um IP2Y12. Houve menor risco de sangramento com a apixabana comparada a varfarina. No grupo que utilizou a AAS em comparação ao placebo, houve uma maior incidência de sangramento sem diferença quanto aos eventos isquêmicos.^210^

O ENTRUST-AF PCI foi um estudo randomizado, multicêntrico, com inclusão de pacientes com FA em uso de ACO que foram submetidos a ICP para DAC estável ou SCA. Eles foram randomizados para receber a terapia dupla com edoxabana associado a clopidogrel ou a terapia tripla tradicional (varfarina com DAPT com AAS e clopidogrel). A terapia com edoxabana não foi inferior para sangramento em comparação com o regime baseado nos antagonistas da vitamina K.^728^

A DAPT com AAS e um IP2Y12, preferencialmente com clopidogrel, deve ser administrada a todos os pacientes durante o procedimento da ICP até o momento da alta, podendo chegar até 1 mês após a intervenção, dependendo do risco trombótico. A terapia dupla com clopidogrel e DOAC deve ser administrada por 6 a 12 meses dependendo do balanço entre o risco isquêmico e hemorrágico, após o qual os pacientes devem descontinuar a terapia antiplaquetária e receber ACO isoladamente.^730–732^

## 22. Pós-Operatório de Cirurgia de Revascularização Miocárdica – Cuidados e Controles de Rotina

A cirurgia de revascularização miocárdica restabelece o fluxo coronariana distal por contornar um segmento coronariano obstruído, no entanto, sem modificar a doença aterosclerótica em si. Assim, medidas farmacológicas e não farmacológicas de prevenção secundária continuam a ser necessárias no cenário de pós-operatório.

1. Recomendamos que as medidas de prevenção secundária que incluem medidas farmacológicas e não farmacológicas sejam reiniciadas após a cirurgia de revascularização.
  A reabilitação cardíaca, por exemplo, tem sido a estratégia associada à redução de desfechos cardiovasculares no cenário de pós-operatório de cirurgia de revascularização e deve ser recomendada para redução de mortalidade cardiovascular, IM e hospitalizações.^490^
2. Recomendamos que, após a realização de CRM, o paciente seja encaminhando a um programa de reabilitação cardíaca com a finalidade de redução de eventos cardiovasculares subsequentes.
  Aspecto fundamental da terapia medicamentosa é a estratégia antitrombótica. A reintrodução de aspirina de forma precoce tem sabidamente se associado a maior patência de enxertos venosos e à redução de eventos cardiovasculares,^733^ sendo sua reintrodução recomendada a todos os pacientes. Em alguns cenários clínicos, o uso de DAPT após a cirurgia tem se mostrado útil,^734^ como nas cirurgias no contexto das SCAs^734^ e cirurgias sem circulação extracorpórea (CEC), na manutenção da patência de enxertos venosos.^736,737^
3. Recomendamos a introdução de aspirina (100 a 325 mg) 6 horas após a realização da cirurgia, mantendo-se seu uso indefinidamente para redução de oclusão de enxertos venosos e de eventos cardiovasculares.
4. Em pacientes selecionados, sem alto risco de sangramento, pode-se considerar o uso de DAPT com clopidogrel ou ticagrelor por até 1 ano após a cirurgia, para redução de oclusão de enxertos.
  Em relação ao uso de estatinas e inibidores do sistema renina-angiotensina-aldosterona, não há evidência consistente de sua reintrodução precoce.^737,738^ No entanto, seus benefícios em longo prazo justificam a reintrodução antes da alta hospitalar ou logo que o paciente tolere.
  Já em relação aos betabloqueadores, a despeito de resultados conflitantes em relação aos benefícios prognósticos de sua introdução precoce em cirurgias eletivas, seu potencial de redução da incidência de FA de pós-operatório (FAPO) justifica sua introdução precoce, logo que tolerada.^739^
5. Recomendamos a introdução de betabloqueadores logo que tolerado pelo paciente, com o objetivo de reduzir a incidência de FAPO.
  O uso de BCCs também se mostrou útil não apenas na redução de oclusão de enxertos arteriais espásticos, como os enxertos radiais, por exemplo, mas também na redução de eventos cardiovasculares em pacientes que receberam esse tipo de enxerto.^479^
6. Recomendamos a introdução precoce de BCCs em pacientes submetidos a cirurgia de revascularização com uso de enxerto radial e manutenção de pelo menos 1 ano, com objetivo de redução de oclusão do enxerto e de eventos cardiovasculares.
  O uso de drogas antiarrítmicas e anticoagulação plena também pode ser necessário no contexto pós-operatório em caso da ocorrência de FAPO. Trata-se da arritmia mais frequente no pós-operatório de cirurgia de revascularização miocárdica, ocorrendo em cerca de 20% dos casos.^741^ A cardioversão química, elétrica, ou o controle de FC são estratégias possíveis, uma vez que mais de 90% dos pacientes, independentemente da estratégia escolhida, estão em ritmo sinusal 60 dias após a cirurgia.^742^ Quanto à anticoagulação, a despeito de evidências conflitantes quanto à necessidade de uso em todos os casos,^741^ está indicada para os episódios de FAPO revertidos ou com duração superior a 24 horas por pelo menos 4 semanas. Além da varfarina, o uso de DOACs parece ser seguro e custo-efetivo nessa população.^743^
7. Recomendamos o uso de anticoagulação plena (com varfarina ou DOAC) após a cirurgia de revascularização miocárdica quando da ocorrência de FAPO revertida ou com duração de pelo menos 24 horas, devendo ser mantida por pelo menos 4 semanas.
  O seguimento de longo prazo de pacientes submetidos a cirurgia de revascularização tem como objetivo não apenas o acompanhamento de eventuais complicações relacionadas ao procedimento, mas também a vigilância da adesão medicamentosa e da ocorrência de eventuais eventos cardiovasculares. Não há consenso na literatura sobre o uso rotineiro de reestratificação da SCC nesses casos. Nos casos de recorrência de sintomas ou perda de função ventricular no seguimento, normalmente recomenda-se o uso de provas funcionais com imagem como método preferencial.^744^ O uso de angioTC de coronárias também tem se mostrado útil na detecção de oclusão de enxertos.^745^ Em casos de pacientes assintomáticos, em algumas populações de maior risco de evento, tem-se recomendando que, após 5 anos da realização do procedimento, uma reestratificação funcional ou anatômica poderia ser realizada (Tabela 52).^746^
8. Recomenda-se a reestratificação com método de imagem para pacientes após a cirurgia de revascularização com recorrência de sintomas ou em caso de perda de função ventricular.
9. Pode-se considerar, em populações de maior risco cardiovascular, uma reestratificação utilizando método com imagem em pacientes assintomáticos após 5 anos da cirurgia.

**Tabela 52:** Prevenção secundária após cirurgia de revascularização miocárdica

Terapia/ação	Recomendação	Grau de recomendação	Nível de evidência
Medidas gerais	Reintrodução da terapia médica otimizada	**I**	**A**
	Encaminhar a serviço de reabilitação	**I**	**A**
Estratégia antitrombótica	Reintrodução de AAS em até 6 horas após e uso por tempo indefinido	**I**	**A**
	DAPT com ticagrelor ou clopidogrel em populações selecionadas com baixo risco de sangramento	**IIb**	**B**
	Uso de varfarina ou DOAC por pelo menos 4 semanas em caso de FAPO (revertida ou de duração de pelo menos 24 horas)	**IIa**	**B**
iECA/BRA/ARNI	Reintrodução logo que tolerado	**I**	**C**
Estatinas	Reintrodução logo que tolerado	**I**	**C**
Betabloqueadores	Reintrodução precoce para redução da incidência de FAPO	**I**	**B**
BCC	Introdução precoce em pacientes submetidos a cirurgia com uso de enxerto radial e manutenção por pelo menos 1 ano	**I**	**B**
Reestratificação no seguimento	Exames com uso de imagem (funcionais ou anatômicos) para pacientes com recorrência de sintomas ou perda de função ventricular	**I**	**B**
	Reestratificação após 5 anos de cirurgia em assintomáticos com alto risco de eventos	**IIb**	**C**

ARNI: inibidor de neprilisina e do receptor da angiotensina (angiotensin receptor neprilysin inhibitor); BCC: bloqueador dos canais de cálcio; BRA: bloqueador de receptor de angiotensina; AAS: ácido acetilsalicílico; DAPT: dupla antiagregação plaquetária; DOAC: anticoagulante oral direto (direct oral anticoagulant); FAPO: fibrilação atrial de pós-operatório; iECA: inibidor da enzima conversora de angiotensina.
