# Errata: Diretriz de Síndrome Coronariana Crônica – 2025

**DOI:** 10.36660/abc.20260565

**Published:** 2026-07-28

No artigo Cesar LAM, et al. Diretriz de Síndrome Coronariana Crônica – 2025. Arq Bras Cardiol. 2025; 122(9):e20250619. doi: https://doi.org/10.36660/abc.20250619 foram adicionados dois nomes na seção de autoria: Erika Maria Gonçalves Campana, instituição: Hospital Universitário Pedro Ernesto, Rio de Janeiro, RJ – Brasil, incluída como instituição 48; Ricardo Stein, instituição: Universidade Federal do Rio Grande do Sul, Porto Alegre, RS – Brasil, incluída como instituição 49. Na seção com a lista das declarações de conflito de interesse, pág. 3, foram adicionadas as declarações conforme abaixo:

**Table:** 

Diretriz de Síndrome Coronariana Crônica – 2025	
O relatório abaixo lista as declarações de interesse conforme relatadas à SBC pelos especialistas durante o período de desenvolvimento deste posicionamento, 2024/2025.	
Especialista	Tipo de relacionamento com a indústria
Erika Maria Gonçalves Campana	Declaração financeira A - Pagamento de qualquer espécie e desde que economicamente apreciáveis, feitos a (i) você, (ii) ao seu cônjuge/ companheiro ou a qualquer outro membro que resida com você, (iii) a qualquer pessoa jurídica em que qualquer destes seja controlador, sócio, acionista ou participante, de forma direta ou indireta, recebimento por palestras, aulas, atuação como proctor de treinamentos, remunerações, honorários pagos por participações em conselhos consultivos, de investigadores, ou outros comitês, etc. Provenientes da indústria farmacêutica, de órteses, próteses, equipamentos e implantes, brasileiras ou estrangeiras: - Palestras: Servier: Triplixam; Biolab: Press Pluss, Bhena; AstraZeneca: Xarelto; EMS Bramicar. Outros relacionamentos Financiamento de atividades de educação médica continuada, incluindo viagens, hospedagens e inscrições para congressos e cursos, provenientes da indústria farmacêutica, de órteses, próteses, equipamentos e implantes, brasileiras ou estrangeiras: - Servier: Congresso Europeu de Cardiologia.
Ricardo Stein	Nada a ser declarado

**Equipe editorial**

