# Supplementary material for: Diretriz de Síndrome Coronariana Crônica – 2025
Source: Arq Bras Cardiol. 2025 Sep 16;122(9):e20250619. [Article in Portuguese] doi: 10.36660/abc.20250619 (PMC13400070; doi:10.36660/abc.20250619)
Supplement: Translation

# Translation: Guideline for Chronic Coronary Syndrome – 2025

**DOI:** 10.36660/abc.20250619i

**Authors:** Luiz A. Machado Cesar, Luis Henrique W. Gowdak, Ricardo Pavanello, João Fernando M. Ferreira, Bruno M. Mioto, Nilson T. Poppi, Caio Menezes M. de Mendonça, Adenalva Lima de Souza Beck, Adriana Soares Xavier de Brito, Alexandre Abizaid, Andréa Araujo Brandão, Andrea Maria Gomes Marinho Falcão, Andrei Sposito, Antônio Carlos Sobral Sousa, Antonio de Padua Mansur, Ariane Vieira Scarlatelli Macedo, Áurea Jacob Chaves, Breno de Alencar Araripe Falcão, Brivaldo Markman, Bruno Ramos Nascimento, Camila Paixão Jordão, Carlos Augusto Homem de Magalhães Campos, Carlos Eduardo Lucena Montenegro, Carlos Eduardo Rochitte, Carlos Vicente Serrano, Celia Maria Cassaro Strunz, Daniel Medeiros Moreira, Danielle Misumi Watanabe, Eduardo Gomes Lima, Eduardo Nagib Gaui, Elizabeth Regina Giunco Alexandre, Fabiana Hanna Rached, Fabio Biscegli Jatene, Gentil Barreira de Aguiar, Gilson Soares Feitosa-Filho, Henrique Patrus Mundim Pena, Ibraim Masciarelli Francisco Pinto, Iran Castro, Jaqueline R. Scholz, João Carlos Ferreira Leal, José Armando Mangione, José Jayme Galvão de Lima, Jose Rocha Faria, José Soares, Kleisson Antônio Pontes Maia, Lara Cristiane Terra Ferreira Carreira, Luciana Diniz Nagem Janot de Matos, Luciana Oliveira Cascaes Dourado, Luhanda Leonora Cardoso Monti Sousa, Luis Alberto Oliveira Dallan, Luiz Eduardo Mastrocola, Marcia Maria Godoy Gowdak, Olimpio Ribeiro França, Otavio Rizzi Coelho, Otávio R. Coelho-Filho, Paulo Eduardo Ballvé Behr, Paulo Ricardo Avancini Caramori, Pedro Alves Lemos, Pedro Silvio Farsky, Raul Dias dos Santos, Renato D. Lopes, Salvador Manoel Serra, Sarah Fagundes Grobe, Sérgio Tavares Montenegro, Silvio Henrique Barberato, Tania Mara Varejão Strabelli, Ursula Maria Moreira Costa Burgos, Vinicius José da Silva Nina, Walter Jose Gomes, William Azem Chalela, Wilson Mathias

**Affiliations:** 1 São Paulo SP Brazil Instituto do Coração (Incor) do Hospital das Clínicas da Faculdade de Medicina da Universidade de São Paulo (HCFMUSP), São Paulo, SP – Brazil; 2 São Paulo SP Brazil Instituto Dante Pazzanese de Cardiologia, São Paulo, SP – Brazil; 3 Brasília DF Brazil Instituto de Cardiologia e Transplantes do Distrito Federal, Brasília, DF – Brazil; 4 Brasília DF Brazil Hospital Sírio Libanês Brasília, Brasília, DF – Brazil; 5 Rio de Janeiro RJ Brazil Instituto Nacional de Cardiologia, Rio de Janeiro, RJ – Brazil; 6 Rio de Janeiro RJ Brazil Rede D’Or, Rio de Janeiro, RJ – Brazil; 7 Rio de Janeiro RJ Brazil DASA, Rio de Janeiro, RJ – Brazil; 8 Rio de Janeiro RJ Brazil Universidade do Estado do Rio de Janeiro (UERJ), Rio de Janeiro, RJ – Brazil; 9 Campinas SP Brazil Universidade Estadual de Campinas (UNICAMP), Campinas, SP – Brazil; 10 Aracaju SE Brazil Universidade Federal de Sergipe (UFS), Aracaju, SE – Brazil; 11 Aracaju SE Brazil Hospital São Lucas, Rede D'Or de Aracaju, Aracaju, SE – Brazil; 12 São Paulo SP Brazil Faculdade de Ciências Médicas da Santa Casa de São Paulo, São Paulo, SP – Brazil; 13 Fortaleza CE Brazil Hospital de Messejana, Fortaleza, CE – Brazil; 14 Fortaleza CE Brazil Hospital Universitário Walter Cantídio, Universidade Federal do Ceará, Fortaleza, CE – Brazil; 15 Fortaleza CE Brazil Hospital Monte Klinikum, Fortaleza, CE – Brazil; 16 Recife PE Brazil Universidade Federal de Pernambuco, Recife, PE – Brazil; 17 Belo Horizonte MG Brazil Universidade Federal de Minas Gerais, Belo Horizonte, MG – Brazil; 18 Recife PE Brazil Hospital do Coração de PE (PROCAPE), Recife, PE – Brazil; 19 Recife PE Brazil Universidade de Pernambuco (UPE), Recife, PE – Brazil; 20 Recife PE Brazil Hospital Esperança Recife, Rede D'or, Recife, PE – Brazil; 21 São José SC Brazil Instituto de Cardiologia de Santa Catarina, São José, SC – Brazil; 22 Rio de Janeiro RJ Brazil Hospital Municipal Miguel Couto, Rio de Janeiro, RJ – Brazil; 23 São Paulo SP Brazil Hospital do Coração (HCor), São Paulo, SP – Brazil; 24 Salvador BA Brazil Escola Bahiana de Medicina e Saúde Pública, Salvador, BA – Brazil; 25 Salvador BA Brazil Hospital Santa Izabel, Salvador, BA – Brazil; 26 Salvador BA Brazil Santa Casa de Misericórdia da Bahia, Salvador, BA – Brazil; 27 Belo Horizonte MG Brazil Rede Mater Dei de Saúde, Belo Horizonte, MG – Brazil; 28 São Paulo SP Brazil Grupo Fleury, São Paulo, SP – Brazil; 29 Porto Alegre RS Brazil Instituto de Cardiologia do Rio Grande do Sul, Porto Alegre, RS – Brazil; 30 São José do Rio Preto SP Brazil Faculdade de Medicina de São José do Rio Preto (FAMERP), São José do Rio Preto, SP – Brazil; 31 São José do Rio Preto SP Brazil Hospital Beneficência Portuguesa de São José do Rio Preto, São José do Rio Preto, SP – Brazil; 32 São Paulo SP Brazil Hospital Beneficência Portuguesa de São Paulo, São Paulo, SP – Brazil; 33 Curitiba PR Brazil Pontifícia Universidade Católica do Paraná, Curitiba, PR – Brazil; 34 Belo Horizonte MG Brazil Faculdade Ciências Médicas de Minas Gerais, Belo Horizonte, MG – Brazil; 35 Curitiba PR Brazil Cardiologia Nuclear de Curitiba (CNC), Curitiba, PR – Brazil; 36 São Paulo SP Brazil Hospital Israelita Albert Einstein, São Paulo, SP – Brazil; 37 São Paulo SP Brazil Escola Vera Cruz, São Paulo, SP – Brazil; 38 Curitiba PR Brazil Quanta Diagnóstico, Curitiba, PR – Brazil; 39 Porto Alegre RS Brazil Santa Casa de Porto Alegre, Porto Alegre, RS – Brazil; 40 Porto Alegre RS Brazil Hospital São Lucas da Pontifícia Universidade Católica do Rio Grande do Sul (PUCRS), Porto Alegre, RS – Brazil; 41 São Paulo SP Brazil Universidade de São Paulo (USP), São Paulo, SP – Brazil; 42 Durham USA Duke University Medical Center, Durham – USA; 43 Rio de Janeiro RJ Brazil Instituto Estadual de Cardiologia Aloysio de Castro, Rio de Janeiro, RJ – Brazil; 44 Curitiba PR Brazil Centro de Diagnóstico Cardiovascular (CardioEco), Curitiba, PR – Brazil; 45 Aracaju SE Brazil Universidade Tiradentes, Aracaju, SE – Brazil; 46 São Luís MA Brazil Universidade Federal do Maranhão, São Luís, MA – Brazil; 47 São Paulo SP Brazil Universidade Federal de São Paulo, São Paulo, SP – Brazil

**Table:** 

Guideline for Chronic Coronary Syndrome – 2025	
The report below lists declarations of interest as reported to the SBC by the experts during the period of the development of these statement, 2024/2025.	
Expert	Type of relationship with industry
**Adenalva Lima de Souza Beck**	Nothing to be declared
Adriana Soares Xavier de Brito	Nothing to be declared
Alexandre Abizaid	Other relationships Funding of continuing medical education activities, including travel, accommodation and registration in conferences and courses, from the brazilian or international pharmaceutical, orthosis, prosthesis, equipment and implants industry: - CRF, Polares, Shiffamed.
Andréa Araujo Brandão	Financial declaration A - Economically relevant payments of any kind made to (i) you, (ii) your spouse/partner or any other person living with you, (iii) any legal person in which any of these is either a direct or indirect controlling owner, business partner, shareholder or participant; any payments received for lectures, lessons, training instruction, compensation, fees paid for participation in advisory boards, investigative boards or other committees, etc. from the brazilian or international pharmaceutical, orthosis, prosthesis, equipment and implants industry: - Biolab: Nebivolol; AstraZeneca: Metoprolol; Servier: Perindopril, Hypera; Mantecorp: Olmesartana e combinações; Torrent: Olmesartana e combinações; Daiichi Sankyo: Olmesartana e combinações. B - Research funding under your direct/personal responsibility (directed to the department or institution) from the brazilian or international pharmaceutical, orthosis, prosthesis, equipment and implants industry: - Daiichi Sankyo: Olmesartan; Servant: Elfie; Brainfarma: Olmesartan. Other relationships Funding of continuing medical education activities, including travel, accommodation and registration in conferences and courses, from the brazilian or international pharmaceutical, orthosis, prosthesis, equipment and implants industry: - Servier: Perindopril; Novo Nordisk: Semaglutide.
Andrea Maria Gomes Marinho Falcão	Nothing to be declared
Andrei Sposito	Financial declaration A - Economically relevant payments of any kind made to (i) you, (ii) your spouse/partner or any other person living with you, (iii) any legal person in which any of these is either a direct or indirect controlling owner, business partner, shareholder or participant; any payments received for lectures, lessons, training instruction, compensation, fees paid for participation in advisory boards, investigative boards or other committees, etc. from the brazilian or international pharmaceutical, orthosis, prosthesis, equipment and implants industry: - Lilly, Novo Nordisk, Daiichi Sankyo. B - Research funding under your direct/personal responsibility (directed to the department or institution) from the brazilian or international pharmaceutical, orthosis, prosthesis, equipment and implants industry: - AstraZeneca. Other relationships Funding of continuing medical education activities, including travel, accommodation and registration in conferences and courses, from the brazilian or international pharmaceutical, orthosis, prosthesis, equipment and implants industry: - Daiichi, Novo Nordisk.
Antônio Carlos Sobral Sousa	Nothing to be declared
Antonio de Padua Mansur	Nothing to be declared
Ariane Vieira Scarlatelli Macedo	Financial declaration A - Economically relevant payments of any kind made to (i) you, (ii) your spouse/partner or any other person living with you, (iii) any legal person in which any of these is either a direct or indirect controlling owner, business partner, shareholder or participant; any payments received for lectures, lessons, training instruction, compensation, fees paid for participation in advisory boards, investigative boards or other committees, etc. from the brazilian or international pharmaceutical, orthosis, prosthesis, equipment and implants industry: - Adium: breast cancer; Amgen: Myeloma; Astellas: prostate cancer; AstraZeneca: Forxiga; BeOne: Zanubrutinib; BMS: Myelodysplasia and Mavacantene; Bayer: prostate cancer; Edwards: aortic stenosis; Ferring: prostate cancer; Johnson: prostate cancer and CLL; Novo Nordisk: Diabetes; Novartis: Sybrava, Entresto; Pfizer: amyloidosis and CML. B - Research funding under your direct/personal responsibility (directed to the department or institution) from the brazilian or international pharmaceutical, orthosis, prosthesis, equipment and implants industry: - Ferring: prostate cancer. Other relationships Funding of continuing medical education activities, including travel, accommodation and registration in conferences and courses, from the brazilian or international pharmaceutical, orthosis, prosthesis, equipment and implants industry: - Novo Nordisk: diabetes; Novartis: Entresto, Sybrava.
Áurea Jacob Chaves	Nothing to be declared
Breno de Alencar Araripe Falcão	Nothing to be declared
Brivaldo Markman Filho	Nothing to be declared
Bruno Mahler Mioto	Financial declaration A - Economically relevant payments of any kind made to (i) you, (ii) your spouse/partner or any other person living with you, (iii) any legal person in which any of these is either a direct or indirect controlling owner, business partner, shareholder or participant; any payments received for lectures, lessons, training instruction, compensation, fees paid for participation in advisory boards, investigative boards or other committees, etc. from the brazilian or international pharmaceutical, orthosis, prosthesis, equipment and implants industry: - Servier: coronary heart disease; Novo Nordisk: diabetes and obesity; Novartis: dyslipidemia; Daiichi Sankyo: dyslipidemia; Boehringer Ingelheim: diabetes. Other relationships Funding of continuing medical education activities, including travel, accommodation and registration in conferences and courses, from the brazilian or international pharmaceutical, orthosis, prosthesis, equipment and implants industry: - Servier: coronary heart disease; Novo Nordisk: diabetes and obesity.
Bruno Ramos Nascimento	Nothing to be declared
Caio Menezes Machado de Mendonça	Financial declaration A - Economically relevant payments of any kind made to (i) you, (ii) your spouse/partner or any other person living with you, (iii) any legal person in which any of these is either a direct or indirect controlling owner, business partner, shareholder or participant; any payments received for lectures, lessons, training instruction, compensation, fees paid for participation in advisory boards, investigative boards or other committees, etc. from the brazilian or international pharmaceutical, orthosis, prosthesis, equipment and implants industry: - Servier: Vastarel, chronic coronary syndrome; Novartis: Sybrava, dyslipidemia; Chiesi: Trimbow, COPD; Libbs: Plenance, dyslipidemia; Novo Nordisk: Rybelsus. Other relationships Funding of continuing medical education activities, including travel, accommodation and registration in conferences and courses, from the brazilian or international pharmaceutical, orthosis, prosthesis, equipment and implants industry: - Servier: Brazilian Congress of Cardiology 2024; Daiichi-Sankyo: SOCESP Congress 2024; Novo Nordisk: SOCESP Congress 2025; Novartis: Congress.
Camila Paixão Jordão	Nothing to be declared
Carlos Augusto Homem de Magalhães Campos	Nothing to be declared
Carlos Eduardo Lucena Montenegro	Financial declaration A - Economically relevant payments of any kind made to (i) you, (ii) your spouse/partner or any other person living with you, (iii) any legal person in which any of these is either a direct or indirect controlling owner, business partner, shareholder or participant; any payments received for lectures, lessons, training instruction, compensation, fees paid for participation in advisory boards, investigative boards or other committees, etc. from the brazilian or international pharmaceutical, orthosis, prosthesis, equipment and implants industry: - Bayer: Firialta (HF); Pfizer: Vyndaquel/Vynkella (Amyloidosis); Alnylan: Amvuttra (Amyloidosis) Servier: Vastarel (CAD); Novartis: Entresto, Sybrava (HF, DLP); AstraZeneca: Forxiga, Lokelma, Breztri (HF, CKD, COPD); Viatris: Inspra (HF); Merck: Concor HF, CAD); Novo Nordisk: Wegovy (Obesity, CAD); EMS: Vynaxa (CAD). Other relationships Funding of continuing medical education activities, including travel, accommodation and registration in conferences and courses, from the brazilian or international pharmaceutical, orthosis, prosthesis, equipment and implants industry: - Novo Nordisk: Wegovy (Obesity, HF).
Carlos Eduardo Rochitte	Financial declaration A - Economically relevant payments of any kind made to (i) you, (ii) your spouse/partner or any other person living with you, (iii) any legal person in which any of these is either a direct or indirect controlling owner, business partner, shareholder or participant; any payments received for lectures, lessons, training instruction, compensation, fees paid for participation in advisory boards, investigative boards or other committees, etc. from the brazilian or international pharmaceutical, orthosis, prosthesis, equipment and implants industry: - GE Healthcare, Canon Medical Systems, Novartis, Cleerly.
Carlos Vicente Serrano Jr.	Nothing to be declared
Celia Maria Cassaro Strunz	Nothing to be declared
Daniel Medeiros Moreira	Nothing to be declared
Danielle Misumi Watanabe	Nothing to be declared
Eduardo Gomes Lima	Financial declaration A - Economically relevant payments of any kind made to (i) you, (ii) your spouse/partner or any other person living with you, (iii) any legal person in which any of these is either a direct or indirect controlling owner, business partner, shareholder or participant; any payments received for lectures, lessons, training instruction, compensation, fees paid for participation in advisory boards, investigative boards or other committees, etc. from the brazilian or international pharmaceutical, orthosis, prosthesis, equipment and implants industry: - Novo Nordisk: obesity and diabetes; Daiichi Sankyo: dyslipidemia and antiplatelet therapy; Novartis: Inclisiran; Bayer: finerenone; AstraZeneca: forxiga; Lilly: Mounjaro. B - Research funding under your direct/personal responsibility (directed to the department or institution) from the brazilian or international pharmaceutical, orthosis, prosthesis, equipment and implants industry: - Lilly: Mounjaro; Novo Nordisk: Ziltivekimab; Novartis: Inclisirana. Other relationships Funding of continuing medical education activities, including travel, accommodation and registration in conferences and courses, from the brazilian or international pharmaceutical, orthosis, prosthesis, equipment and implants industry: - Novo Nordisk: obesity and diabetes; Lilly: obesity.
Eduardo Nagib Gaui	Nothing to be declared
Elizabeth Regina Giunco Alexandre	Financial declaration A - Economically relevant payments of any kind made to (i) you, (ii) your spouse/partner or any other person living with you, (iii) any legal person in which any of these is either a direct or indirect controlling owner, business partner, shareholder or participant; any payments received for lectures, lessons, training instruction, compensation, fees paid for participation in advisory boards, investigative boards or other committees, etc. from the brazilian or international pharmaceutical, orthosis, prosthesis, equipment and implants industry: - Servier: Vastarel MR; Lilly: Mounjaro; Libbs: Ebatz e Stanglitz, Novo Nordisk: Ozempic; AstraZeneca: Breztri; Boehringer-Ingelhein: Glyxambi; Mantecorpp: Nesina/Addera. Other relationships Funding of continuing medical education activities, including travel, accommodation and registration in conferences and courses, from the brazilian or international pharmaceutical, orthosis, prosthesis, equipment and implants industry: - Lilly.
Fabiana Hanna Rached	Financial declaration A - Economically relevant payments of any kind made to (i) you, (ii) your spouse/partner or any other person living with you, (iii) any legal person in which any of these is either a direct or indirect controlling owner, business partner, shareholder or participant; any payments received for lectures, lessons, training instruction, compensation, fees paid for participation in advisory boards, investigative boards or other committees, etc. from the brazilian or international pharmaceutical, orthosis, prosthesis, equipment and implants industry: - Novo Nordisk: semaglutide; Novartis: inclisiran; Daiichi Sankyo: bempedoic acid. Other relationships Funding of continuing medical education activities, including travel, accommodation and registration in conferences and courses, from the brazilian or international pharmaceutical, orthosis, prosthesis, equipment and implants industry: - Novo Nordisk: semaglutide; Novartis: inclisiran; Daiichi Sankyo: bempedoic acid.
Fabio Biscegli Jatene	Nothing to be declared
Gentil Barreira de Aguiar Filho	Nothing to be declared
Gilson Soares Feitosa-Filho	Declaração financeira B - Research funding under your direct/personal responsibility (directed to the department or institution) from the brazilian or international pharmaceutical, orthosis, prosthesis, equipment and implants industry: - Amgen: Olpasiran; Idors ia: Selatogrel; Anthos: Abelacimabe; Jansen e Bayer: Milvexiana. Other relationships Funding of continuing medical education activities, including travel, accommodation and registration in conferences and courses, from the brazilian or international pharmaceutical, orthosis, prosthesis, equipment and implants industry: - Servier/Trimetazidine: Congress Registration.
Henrique Patrus Mundim Pena	Financial declaration A - Economically relevant payments of any kind made to (i) you, (ii) your spouse/partner or any other person living with you, (iii) any legal person in which any of these is either a direct or indirect controlling owner, business partner, shareholder or participant; any payments received for lectures, lessons, training instruction, compensation, fees paid for participation in advisory boards, investigative boards or other committees, etc. from the brazilian or international pharmaceutical, orthosis, prosthesis, equipment and implants industry: - AstraZeneca: heart failure/dapaglizin/hyperkalemia; Viatris: heart failure/eplerenone. Other relationships Any economically relevant equity interest in companies in the healthcare or education industry or in any companies competing with or supplying to SBC: - Novo Nordisk: obesity, cardiometabolism.
Ibraim Masciarelli Francisco Pinto	Nothing to be declared
Iran Castro	Nothing to be declared
Jaqueline R Scholz	Nothing to be declared
João Carlos Ferreira Leal	Financial declaration A - Economically relevant payments of any kind made to (i) you, (ii) your spouse/partner or any other person living with you, (iii) any legal person in which any of these is either a direct or indirect controlling owner, business partner, shareholder or participant; any payments received for lectures, lessons, training instruction, compensation, fees paid for participation in advisory boards, investigative boards or other committees, etc. from the brazilian or international pharmaceutical, orthosis, prosthesis, equipment and implants industry: - Braile Biomédica: proctor and lectures; Neomex: proctor and lectures. B - Research funding under your direct/personal responsibility (directed to the department or institution) from the brazilian or international pharmaceutical, orthosis, prosthesis, equipment and implants industry: - Braile Biomédica: biological prosthesis. Other relationships Funding of continuing medical education activities, including travel, accommodation and registration in conferences and courses, from the brazilian or international pharmaceutical, orthosis, prosthesis, equipment and implants industry: - Braile Biomédica: lectures.
João Fernando Monteiro Ferreira	Nothing to be declared
José Armando Mangione	Financial declaration A - Economically relevant payments of any kind made to (i) you, (ii) your spouse/partner or any other person living with you, (iii) any legal person in which any of these is either a direct or indirect controlling owner, business partner, shareholder or participant; any payments received for lectures, lessons, training instruction, compensation, fees paid for participation in advisory boards, investigative boards or other committees, etc. from the brazilian or international pharmaceutical, orthosis, prosthesis, equipment and implants industry: - Edwards Lifescience: TAVI proctor; Medtronic: TAVI proctor.
José Jayme Galvão de Lima	Nothing to be declared
José Rocha Faria Neto	Financial declaration A - Economically relevant payments of any kind made to (i) you, (ii) your spouse/partner or any other person living with you, (iii) any legal person in which any of these is either a direct or indirect controlling owner, business partner, shareholder or participant; any payments received for lectures, lessons, training instruction, compensation, fees paid for participation in advisory boards, investigative boards or other committees, etc. from the brazilian or international pharmaceutical, orthosis, prosthesis, equipment and implants industry: - Aché: CAD and dyslipidemia; Daiichi Sankyo: CAD and dyslipidemia; Libbs: CAD and dyslipidemia; Novartis: dyslipidemia; AstraZeneca: diabetes; Lilly: diabetes and obesity; Novo Nordisk: diabetes and obesity; Sanofi and Medley: dyslipidemia; Bayer: cardiovascular risk. Other relationships Funding of continuing medical education activities, including travel, accommodation and registration in conferences and courses, from the brazilian or international pharmaceutical, orthosis, prosthesis, equipment and implants industry: - Novo Nordisk; Bayer; Daiichi Sankyo; AstraZeneca.
José Soares Junior	Nothing to be declared
Kleisson Antônio Pontes Maia	Financial declaration A - Economically relevant payments of any kind made to (i) you, (ii) your spouse/partner or any other person living with you, (iii) any legal person in which any of these is either a direct or indirect controlling owner, business partner, shareholder or participant; any payments received for lectures, lessons, training instruction, compensation, fees paid for participation in advisory boards, investigative boards or other committees, etc. from the brazilian or international pharmaceutical, orthosis, prosthesis, equipment and implants industry: - Novartis: hypercholesterolemia; GSK: vaccines; Biolab: hypertension; Lilly: diabetes, obesity; Novo Nordisk: diabetes, obesity; Servier: coronary heart disease. B - Research funding under your direct/personal responsibility (directed to the department or institution) from the brazilian or international pharmaceutical, orthosis, prosthesis, equipment and implants industry: - Lilly: Lp(a). Other relationships Funding of continuing medical education activities, including travel, accommodation and registration in conferences and courses, from the brazilian or international pharmaceutical, orthosis, prosthesis, equipment and implants industry: - Servier: coronary heart disease; Lilly: diabetes; Novo Nordisk: diabetes, obesity; Viatris: hypercholesterolemia.
Lara Cristiane Terra Ferreira Carreira	Nothing to be declared
Luciana Diniz Nagem Janot de Matos	Nothing to be declared
Luciana Oliveira Cascaes Dourado	Nothing to be declared
Luhanda Leonora Cardoso Monti Sousa	Other relationships Funding of continuing medical education activities, including travel, accommodation and registration in conferences and courses, from the brazilian or international pharmaceutical, orthosis, prosthesis, equipment and implants industry: - Novo Nordisk: semaglutide.
Luis Alberto Oliveira Dallan	Nothing to be declared
Luís Henrique Wolff Gowdak	Financial declaration A - Economically relevant payments of any kind made to (i) you, (ii) your spouse/partner or any other person living with you, (iii) any legal person in which any of these is either a direct or indirect controlling owner, business partner, shareholder or participant; any payments received for lectures, lessons, training instruction, compensation, fees paid for participation in advisory boards, investigative boards or other committees, etc. from the brazilian or international pharmaceutical, orthosis, prosthesis, equipment and implants industry: - Servier: angina; Novartis: hypercholesterolemia; GSK: vaccination. B - Research funding under your direct/personal responsibility (directed to the department or institution) from the brazilian or international pharmaceutical, orthosis, prosthesis, equipment and implants industry: - Servier: angina. Other relationships Funding of continuing medical education activities, including travel, accommodation and registration in conferences and courses, from the brazilian or international pharmaceutical, orthosis, prosthesis, equipment and implants industry: - Novo Nordisk: obesity and diabetes; Lilly: obesity.
Luiz Antonio Machado Cesar	Financial declaration A - Economically relevant payments of any kind made to (i) you, (ii) your spouse/partner or any other person living with you, (iii) any legal person in which any of these is either a direct or indirect controlling owner, business partner, shareholder or participant; any payments received for lectures, lessons, training instruction, compensation, fees paid for participation in advisory boards, investigative boards or other committees, etc. from the brazilian or international pharmaceutical, orthosis, prosthesis, equipment and implants industry: - Servier: Vastarel/ angina. Other relationships Funding of continuing medical education activities, including travel, accommodation and registration in conferences and courses, from the brazilian or international pharmaceutical, orthosis, prosthesis, equipment and implants industry: - EMS: Vynaxa/anticoagulation.
Luiz Eduardo Mastrocola	Nothing to be declared
Marcia Maria Godoy Gowdak	Nothing to be declared
Nilson Tavares Poppi	Financial declaration A - Economically relevant payments of any kind made to (i) you, (ii) your spouse/partner or any other person living with you, (iii) any legal person in which any of these is either a direct or indirect controlling owner, business partner, shareholder or participant; any payments received for lectures, lessons, training instruction, compensation, fees paid for participation in advisory boards, investigative boards or other committees, etc. from the brazilian or international pharmaceutical, orthosis, prosthesis, equipment and implants industry: - EMS: coronary heart disease; Boehringer-Ingelheim: anticoagulation; Servier: coronary heart disease. Other relationships Funding of continuing medical education activities, including travel, accommodation and registration in conferences and courses, from the brazilian or international pharmaceutical, orthosis, prosthesis, equipment and implants industry: - Servier: coronary disease.
Olimpio Ribeiro França Neto	Financial declaration A - Economically relevant payments of any kind made to (i) you, (ii) your spouse/partner or any other person living with you, (iii) any legal person in which any of these is either a direct or indirect controlling owner, business partner, shareholder or participant; any payments received for lectures, lessons, training instruction, compensation, fees paid for participation in advisory boards, investigative boards or other committees, etc. from the brazilian or international pharmaceutical, orthosis, prosthesis, equipment and implants industry: - Servier: angina; Biolab: hypertension. B - Research funding under your direct/personal responsibility (directed to the department or institution) from the brazilian or international pharmaceutical, orthosis, prosthesis, equipment and implants industry: - Servier: Angina. Other relationships Funding of continuing medical education activities, including travel, accommodation and registration in conferences and courses, from the brazilian or international pharmaceutical, orthosis, prosthesis, equipment and implants industry: - Servier: angina; Biolab: hypertension.
Otavio Rizzi Coelho	Financial declaration A - Economically relevant payments of any kind made to (i) you, (ii) your spouse/partner or any other person living with you, (iii) any legal person in which any of these is either a direct or indirect controlling owner, business partner, shareholder or participant; any payments received for lectures, lessons, training instruction, compensation, fees paid for participation in advisory boards, investigative boards or other committees, etc. from the brazilian or international pharmaceutical, orthosis, prosthesis, equipment and implants industry: - Novartis: Inclisiran; Novo Nordisk: Semaglutide; AstraZeneca: Dapaglifozin; Daiichi Sankyo: Ticagrelor; Bayer: Phenirinone. Other relationships Funding of continuing medical education activities, including travel, accommodation and registration in conferences and courses, from the brazilian or international pharmaceutical, orthosis, prosthesis, equipment and implants industry: - AstraZeneca: Dapaglifozin; Novo Nordisk: Semaglutide.
Paulo Eduardo Ballvé Behr	Financial declaration A - Economically relevant payments of any kind made to (i) you, (ii) your spouse/partner or any other person living with you, (iii) any legal person in which any of these is either a direct or indirect controlling owner, business partner, shareholder or participant; any payments received for lectures, lessons, training instruction, compensation, fees paid for participation in advisory boards, investigative boards or other committees, etc. from the brazilian or international pharmaceutical, orthosis, prosthesis, equipment and implants industry: - Novartis: Sybrava; Novartis: Cosentyx; Biolab: Livalo; Aché: Thirteen; PTC: Volanesorsen; Libbs: Zinpass; Novo Nordisk: Ozempic; Daiichi Sankyo: Nustendi; Amgen: Olpasiran. Other relationships Funding of continuing medical education activities, including travel, accommodation and registration in conferences and courses, from the brazilian or international pharmaceutical, orthosis, prosthesis, equipment and implants industry: - Novartis: Sybrava; Daiichi: Nustendi; Novo Nordisk: Ozempic.
Otávio R. Coelho-Filho	Financial declaration A - Economically relevant payments of any kind made to (i) you, (ii) your spouse/partner or any other person living with you, (iii) any legal person in which any of these is either a direct or indirect controlling owner, business partner, shareholder or participant; any payments received for lectures, lessons, training instruction, compensation, fees paid for participation in advisory boards, investigative boards or other committees, etc. From the brazilian or international pharmaceutical, orthosis, prosthesis, equipment and implants industry: - Pfizer: amiloidose/Tafamides; AstraZeneca: insuficiência cardíaca/Forxiga; Bayer: Firialta; Norvartis: insuficiência cardíaca/dislipidemia; EMS: insuficiência cardíaca. Other relationships Funding of continuing medical education activities, including travel, accommodation and registration in conferences and courses, from the brazilian or international pharmaceutical, orthosis, prosthesis, equipment and implants industry: - Bayer; Pfizer; AstraZeneca.
Paulo Ricardo Avancini Caramori	Financial declaration B - Research funding under your direct/personal responsibility (directed to the department or institution) from the brazilian or international pharmaceutical, orthosis, prosthesis, equipment and implants industry: - Speaker: Novo Nordisk, Viatris, Abbott, Boston Scientific, Meril Life Sciences.
Pedro Alves Lemos Neto	Financial declaration A - Economically relevant payments of any kind made to (i) you, (ii) your spouse/partner or any other person living with you, (iii) any legal person in which any of these is either a direct or indirect controlling owner, business partner, shareholder or participant; any payments received for lectures, lessons, training instruction, compensation, fees paid for participation in advisory boards, investigative boards or other committees, etc. from the brazilian or international pharmaceutical, orthosis, prosthesis, equipment and implants industry: - Novo Nordisk: inflammation; Abbott Vascular: stent; Daiichi Sankyo: antiplatelet; Edwards Lifesciences: TAVI; B. Braun Melsungen AG: drug-eluting balloon; Boston Scientific: left atrial appendage occlusion. B - Research funding under your direct/personal responsibility (directed to the department or institution) from the brazilian or international pharmaceutical, orthosis, prosthesis, equipment and implants industry: - Novo Nordisk: inflammation.
Pedro Silvio Farsky	Other relationships Funding of continuing medical education activities, including travel, accommodation and registration in conferences and courses, from the brazilian or international pharmaceutical, orthosis, prosthesis, equipment and implants industry: - Novo Nordisk: Semaglutide; Lilly: Tirzepatide; Novartis: Inclisirana.
Raul Dias dos Santos Filho	Financial declaration A - Economically relevant payments of any kind made to (i) you, (ii) your spouse/partner or any other person living with you, (iii) any legal person in which any of these is either a direct or indirect controlling owner, business partner, shareholder or participant; any payments received for lectures, lessons, training instruction, compensation, fees paid for participation in advisory boards, investigative boards or other committees, etc. from the brazilian or international pharmaceutical, orthosis, prosthesis, equipment and implants industry: - Amgen; Novartis;, Arrowhead; Ionis; Torrent, Sanofi; Daiichi Sankyo; Aché: hypolipidemic agents; Novo Nordisk, Eli-Lilly: hypoglycemic agents. B - Research funding under your direct/personal responsibility (directed to the department or institution) from the brazilian or international pharmaceutical, orthosis, prosthesis, equipment and implants industry: - Amgen; Arrowhead, Ionis; Eli-Lilly: hypoglycemic agents.
Renato D. Lopes	Financial declaration A - Economically relevant payments of any kind made to (i) you, (ii) your spouse/partner or any other person living with you, (iii) any legal person in which any of these is either a direct or indirect controlling owner, business partner, shareholder or participant; any payments received for lectures, lessons, training instruction, compensation, fees paid for participation in advisory boards, investigative boards or other committees, etc. from the brazilian or international pharmaceutical, orthosis, prosthesis, equipment and implants industry: - Pfizer, Daiichi Sankyo, Novo Nordisk, Bayer, Boehringer Ingelheim, Bristol-Myers Squibb. B - Research funding under your direct/personal responsibility (directed to the department or institution) from the brazilian or international pharmaceutical, orthosis, prosthesis, equipment and implants industry: - Amgen, Bristol-Myers Squibb, GlaxoSmithKline, Medtronic, Pfizer, Sanofi-Aventis. Other relationships Funding of continuing medical education activities, including travel, accommodation and registration in conferences and courses, from the brazilian or international pharmaceutical, orthosis, prosthesis, equipment and implants industry: - Pfizer, Daiichi Sankyo, Novo Nordisk, Novartis.
Ricardo Pavanello	Nothing to be declared
Salvador Manoel Serra	Nothing to be declared
Sarah Fagundes Grobe	Financial declaration A - Economically relevant payments of any kind made to (i) you, (ii) your spouse/partner or any other person living with you, (iii) any legal person in which any of these is either a direct or indirect controlling owner, business partner, shareholder or participant; any payments received for lectures, lessons, training instruction, compensation, fees paid for participation in advisory boards, investigative boards or other committees, etc. from the brazilian or international pharmaceutical, orthosis, prosthesis, equipment and implants industry: - Biolab: Doble; Novartis: Sybrava; Ache: dyslipidemia; Servier: Vastarel; Mantecorp: Women's health; Daiichi Sankyo: Nustenti. B - Research funding under your direct/personal responsibility (directed to the department or institution) from the brazilian or international pharmaceutical, orthosis, prosthesis, equipment and implants industry: - Servier: Vastarel; Daiichi Sankyo: Nustendi. Other relationships Funding of continuing medical education activities, including travel, accommodation and registration in conferences and courses, from the brazilian or international pharmaceutical, orthosis, prosthesis, equipment and implants industry: - Servier; Novartis.
Sérgio Tavares Montenegro	Financial declaration A - Economically relevant payments of any kind made to (i) you, (ii) your spouse/partner or any other person living with you, (iii) any legal person in which any of these is either a direct or indirect controlling owner, business partner, shareholder or participant; any payments received for lectures, lessons, training instruction, compensation, fees paid for participation in advisory boards, investigative boards or other committees, etc. from the brazilian or international pharmaceutical, orthosis, prosthesis, equipment and implants industry: - Servier: Ticagrelor; Merck: Bisoprolol. Other relationships Funding of continuing medical education activities, including travel, accommodation and registration in conferences and courses, from the brazilian or international pharmaceutical, orthosis, prosthesis, equipment and implants industry: - Servier.
Silvio Henrique Barberato	Financial declaration A - Economically relevant payments of any kind made to (i) you, (ii) your spouse/partner or any other person living with you, (iii) any legal person in which any of these is either a direct or indirect controlling owner, business partner, shareholder or participant; any payments received for lectures, lessons, training instruction, compensation, fees paid for participation in advisory boards, investigative boards or other committees, etc. from the brazilian or international pharmaceutical, orthosis, prosthesis, equipment and implants industry: - Pfizer: amyloidosis; Bristol: Camzyos. Other relationships Funding of continuing medical education activities, including travel, accommodation and registration in conferences and courses, from the brazilian or international pharmaceutical, orthosis, prosthesis, equipment and implants industry: - Pfizer: amyloidosis.
Tania Mara Varejão Strabelli	Nothing to be declared
Ursula Maria Moreira Costa Burgos	Nothing to be declared
Vinicius José da Silva Nina	Nothing to be declared
Walter Jose Gomes	Nothing to be declared
William Azem Chalela	Nothing to be declared
Wilson Mathias Junior	Nothing to be declared

**Table of contents**
**1. Introduction** 13
**2. Diagnosis** 15
  **2.1. Clinical Evaluation** 15
    **2.1.1. Definition of Angina** 16
    **2.1.2. Clinical Assessment of Patients with Chest Pain** 16
      ***2.1.2.1. Medical History*** 16
      ***2.1.2.2. Physical Examination*** 17
      ***2.1.2.3. Differential Diagnosis of Chest Pain*** 17
    **2.1.3. Laboratory Assessment** 18
  **2.2. Noninvasive Testing** 20
    **2.2.1. Exercise Stress Test** 20
    **2.2.2. Echocardiography** 21
      ***2.2.2.1. Transthoracic Echocardiography*** 21
      ***2.2.2.2. Stress Echocardiography*** 22
    **2.2.3. Nuclear Medicine** 23
      ***2.2.3.1. Clinical Decision-making after Radionuclide Perfusion Studies – the Impact of Ischemic Burden on Cardiovascular Outcomes*** 23
      ***2.2.3.2. PET*** 25
      ***2.2.3.3. Coronary Flow Reserve Using Single-photon Emission Computed Tomography with Cadmium-zinc-tellurium*** 26
    **2.2.4. Laboratory Assessment** 29
    **2.2.5. Coronary Computed Tomography** 29
      ***2.2.5.1. Coronary Computed Tomography for Coronary Calcium Scoring*** 29
      ***2.2.5.2. Coronary Computed Tomography Angiography*** 29
    **2.2.6. Cardiovascular Magnetic Resonance Imaging** 31
      ***2.2.6.1. Assessment of Myocardial Ischemia by Cardiovascular Magnetic Resonance Imaging*** 31
      ***2.2.6.2. Assessment of Segmental Contractility and Contractile Reserve by Cardiovascular Magnetic Resonance Imaging*** 31
      ***2.2.6.3. Assessment of Coronary Artery Disease by Cardiovascular Magnetic Resonance Imaging – Myocardial Viability*** 32
      ***2.2.6.4. Applying Cardiovascular Magnetic Resonance Imaging to Assess Viability in Coronary Artery Disease*** 32
      ***2.2.6.5. Recommendation Grade and Level of Evidence*** 32
    **2.2.7. Cardiac Catheterization** 32
**3. Clinical Strategies for Assessing Cardiovascular Risk and Stratifying Coronary Atherosclerotic Disease** 34
  **3.1. Initial Assessment of Risk** 34
    **3.1.1. Laboratory Risk Markers in Chronic Coronary Artery Disease** 34
    **3.1.2. Exercise Stress Test for Prognostic Assessment in Stable Coronary Artery Disease** 35
    **3.1.3. Transthoracic Echocardiography (at Rest and Under Stress)** 35
    **3.1.4. Myocardial Perfusion Scintigraphy** 35
    **3.1.5. Cardiovascular Magnetic Resonance Imaging** 35
    **3.1.6. Coronary Computed Tomography Angiography** 35
    **3.1.7. Summary and Recommendation for Diagnostic Investigation** 36
**4. Clinical Pharmacological Treatment for Reducing the Risk of Major Adverse Cardiovascular Events** 36
  **4.1. Antithrombotic Therapy in Chronic Coronary Syndrome** 36
    **4.1.1. Aspirin** 36
    **4.1.2. P2Y12 Inhibitor**s 36
    **4.1.3. Dual Antiplatelet Therapy** 37
    **4.1.4. Low-Dose Anticoagulation** 39
    **4.1.5. Long-Term Anticoagulation** 39
    **4.1.6. Dual antiplatelet Therapy in Percutaneous Coronary Intervention** 39
    **4.1.7. Anticoagulants in Percutaneous Coronary Intervention** 39
  **4.2. Lipid Management in Chronic Coronary Syndrome** 39
    **4.2.1. Statins and Other Low-Density Lipoprotein Cholesterol-Lowering Agents** 39
    **4.2.2. Lipoprotein(a)** 41
    **4.2.3. Triglyceride-Rich Lipoproteins** 42
  **4.3. Hormone Replacement Therapy** 42
  **4.4. Renin-Angiotensin-Aldosterone System Blockade** 42
    **4.4.1. Neprilysin Inhibitors and Angiotensin Receptor Blockers** 42
    **4.4.2. Aldosterone Antagonists** 43
  **4.5. Colchicine** 43
  **4.6. Antidiabetic Therapy** 44
**5. Clinical-Pharmacological Treatment for Optimal Symptom Control** 45
  **5.1. Beta-Blockers** 46
  **5.2. Trimetazidine** 46
  **5.3. Calcium Channel Blockers** 46
  **5.4. Verapamil** 46
  **5.5. Diltiazem** 46
  **5.6. Dihydropyridines** 46
  **5.7. Ivabradine** 46
  **5.8. Ranolazine** 48
  **5.9. Short- and Long-Acting Nitrates** 48
  **5.10. Allopurinol** 49
  **5.11. Individualized Antianginal Treatment** 49
**6. Invasive Treatment Strategies** 51
  **6.1. Percutaneous Treatment for Symptom Control and Risk Reduction** 51
    **6.1.1. Angiographic Complexity Scores in Decision-Making** 51
      ***6.1.1.1. Recommendation*** 52
  **6.2. Invasive Functional Assessment** 53
    **6.2.1. Assessment of Epicardial Coronary Stenoses** 53
      ***6.2.1.1. Recommendation*** 53
    **6.2.2. Microvascular Angina** 53
      ***6.2.2.1. Recommendation*** 54
  **6.3. Intravascular Imaging Techniques** 54
    **6.3.1. Use of Intravascular Imaging in the Diagnosis and Assessment of Coronary Stenoses** 54
      ***6.3.1.1. Recommendation*** 54
    **6.3.2. Use of Intravascular Imaging to Guide Percutaneous Coronary Intervention** 55
      ***6.3.2.1. Recommendation*** 55
  **6.4. Percutaneous Coronary Intervention as a Strategy for Prognostic Benefit** 55
    **6.4.1. Percutaneous Coronary Intervention as a Strategy for Symptom Relief** 56
  **6.5. Bleeding Risk Scores in Clinical Decision-Making** 56
    **6.5.1. Recommendation** 57
  **6.6. Preparation in Specific Clinical Situations (Patients with Renal Dysfunction or Contrast Allergy)** 57
    **6.6.1. Renal Dysfunction** 57
    **6.6.2. Renal Protection Measures** 58
    **6.6.3. General Measures** 58
    **6.6.4. Fluid Administration** 58
    **6.6.5. Pharmacological Prevention** 59
    **6.6.6. Contrast Media** 59
    **6.6.7. Allergy to Contrast Media** 59
    **6.6.8. Conclusions** 60
  **6.7. Surgical Treatment for Symptom Control and Risk Reduction** 60
    **6.7.1. Patients with Multivessel Coronary Artery Disease and SYNTAX Score > 23** 61
    **6.7.2. Patients with Advanced Coronary Artery Disease and Diabetes** 61
    **6.7.3. Patients with Left Ventricular Dysfunction** 62
    **6.7.4. Patients with Left Main Coronary Artery Disease** 62
    **6.7.5. Relief of Anginal Symptoms and Quality of Life** 63
**7. Vaccination Against Pneumonia, Influenza, and COVID-19** 64
  **7.1. Influenza Vaccine** 64
  **7.2. Adverse Effects and Events** 64
  **7.3. Pneumococcal Vaccine** 64
  **7.4. Guidelines for Sequential PCV13 and PPSV23 Vaccination** 66
  **7.5. COVID-19 Vaccine** 66
  **7.6. Recommended Vaccines** 66
  **7.7. Risk of Myocarditis/Pericarditis Following COVID-19 Vaccination** 66
**8. Diet, Alcohol, and Weight Control** 66
  **8.1. Weight Control** 67
**9. Physical Activity, Cardiac Rehabilitation, and Sexual Activity** 67
  **9.1. Lifestyle Changes and Related Factors** 67
    **9.1.1. Sedentary Behavior, Physical Activity, and Exercise** 67
    **9.1.2. Secondary Prevention (Exercise-Based Cardiac Rehabilitation)** 68
    **9.1.3. Home-Based Rehabilitation** 69
    **9.1.4. Sexual Activity** 71
**10. Tobacco Use and Environmental Pollution** 72
**11. Psychosocial Factors and Treatment Adherence** 73
  **11.1. Psychosocial Factors** 73
  **11.2. Treatment Adherence** 73
**12. Angina and Ischemia with Nonobstructive Coronary Arteries (INOCA, ANOCA)** 73
  **12.1. General Characteristics of Female Ischemic Heart Disease** 74
    **12.1.1. Atherosclerotic Cardiovascular Disease** 74
    **12.1.2. ANOCA and INOCA** 74
    **12.1.3. MINOCA** 74
    **12.1.4. Microvascular Disease** 74
    **12.1.5. Spontaneous Coronary Artery Dissection** 74
    **12.1.6. Coronary Vasospasm** 75
    **12.1.7. Coronary Thrombosis/Embolism** 75
**13. Ischemic Disease in Women – Female-Specific Aspects** 75
  **13.1. Clinical Presentation and Diagnosis** 75
  **13.2. Treatment and Management** 75
  **13.3. Prognostic Factors and Outcomes** 76
  **13.4. Hormonal Influence and Genetic Factors** 76
**14. Refractory Angina** 76
**15. Myocardial Viability** 76
  **15.1. Myocardial Viability** 76
  **15.2. Observational Studies on Viability** 76
  **15.3. Viability and Improvement in Ventricular Function** 78
  **15.4. Contemporary Studies: STICH and REVIVED-BCIS2** 78
  **15.5. Pharmacological Therapy in Coronary Artery Disease and Heart Failure with Reduced Ejection Fraction** 79
  **15.6. Summary** 79
**16. Chronic Kidney Disease IN Chronic Coronary Syndrome** 79
  **16.1. Stratification Using Noninvasive Tests** 80
  **16.2. Stratification Using Invasive Tests** 80
  **16.3. Criticism of the Invasive Strategy for Coronary Artery Disease Investigation** 80
  **16.4. Medication and Dose Adjustment in Patients with Cardiovascular Disease and Chronic Kidney Disease** 81
**17. Cardio-Oncological Aspects in Chronic Coronary Syndrome** 82
**18. Treatment of Hypertension in Patients with Chronic Coronary Artery Disease** 84
**19. Treatment Considerations in the Presence of Atrial Fibrillation** 85
  **19.1. New Oral Anticoagulants Associated with Acetylsalicylic Acid** 85
  **19.2. Anticoagulation in Patients with Chronic Coronary Syndrome and Atrial Fibrillation** 85
**20. Secondary Prevention after Acute Coronary Syndrome** 86
  **20.1. Priorities in the Postdischarge Period after Acute Coronary Syndrome** 86
  **20.2. Risk Stratification after Acute Coronary Syndrome for Secondary Prevention Planning** 86
  **20.3. Risk Variables for Heart Failure and Thrombotic Events** 87
  **20.4. Strategies for Low-Risk Patients** 88
  **20.5. Clinical Follow-Up** 88
**21. Postpercutaneous Coronary Intervention Care in Chronic Coronary Syndrome** 88
  **21.1. Antiplatelet Therapy after Percutaneous Coronary Intervention with Stent Placement in Patients without an Indication for Anticoagulants** 88
  **21.2. Antiplatelet Therapy after Percutaneous Coronary Intervention with Stent in Patients with Indication for Anticoagulation** 88
**22. Postoperative Care after Coronary Artery Bypass Grafting – Routine Monitoring and Management** 90
**References** 91

## 1. Introduction

One of the primary activities of the Brazilian Society of Cardiology (*Sociedade Brasileira de Cardiologia*, SBC) is the development of guidelines for several cardiovascular (CV) diseases (CVDs), based on the most recent scientific evidence and grounded in robust clinical data. These guidelines aim to assist health care professionals in the prevention, diagnosis, and treatment of CVDs. This guideline on chronic coronary syndrome (CCS) was developed by volunteer members of SBC, without any commercial support, and with a strong emphasis on ethical principles to guide clinical cardiology practices and the involvement of all stakeholders.

CVDs are the leading cause of death in the Brazilian population, and CCS stands out as the most significant in terms of morbidity and mortality among noncommunicable diseases. Therefore, this guideline plays a crucial role in supporting health professionals, particularly cardiologists, with recommendations for managing a number of clinical scenarios encountered in patients with CCS.

CCS encompasses a wide range of patient profiles, which are examined in detail throughout this document. The concept was introduced at the European Society of Cardiology (ESC) Congress 2019, aiming to understand coronary artery disease (CAD) as a continuum – typically progressing silently over years, with intermittent acute exacerbations. Each stage of this progression requires distinct strategies, including diagnostic approaches, general lifestyle guidance, medication adjustments, and indications for revascularization procedures. Moreover, it highlights how the occurrence of an acute event significantly alters the trajectory of future CV risk (Central Illustration). Importantly, the CCS framework also emphasizes that many patients – particularly women – may experience angina and ischemia in the absence of obstructive coronary lesions, although atherosclerosis is often present.

Accordingly, Table 1 summarizes patients by clinical scenarios, with or without atherosclerosis, which, when present, may or may not involve obstructive lesions in the coronary tree.

**Table 1:** Clinical profiles of chronic coronary syndrome

1. Patients discharged after hospitalization for an acute coronary syndrome event or following coronary revascularization.
2. Patients with left ventricular systolic dysfunction and known or suspected coronary artery disease, or those with established cardiomyopathy considered to be of ischemic origin.
3. Patients with stable angina symptoms (or ischemic equivalents, such as exertional dyspnea or arm pain) managed clinically, with or without positive findings on imaging tests.
4. Patients presenting with angina symptoms and evidence of CVS or microvascular angina, with or without the presence of atherosclerosis.
5. Patients diagnosed solely based on the results of a screening test (e.g., stress testing, coronary computed tomography angiography), in which the responsible physician determines the presence of CAD.

A useful reference for review is the 2024 ESC guidelines,^1^ although some information should be interpreted with caution. For instance, epidemiological studies conducted in European populations using coronary computed tomography angiography (CCTA) have shown that men over the age of 70 with "typical angina" have a less than 30% likelihood of presenting with obstructive coronary disease.^2^ This represents a substantial departure from the classic Diamond–Forrester classification.^3^ However, such data must be considered carefully, particularly regarding the accuracy of clinical history. In the DISCHARGE (Diagnostic Imaging Strategies for Patients with Stable Chest Pain and Intermediate Risk of Coronary Artery Disease) trial,^4^ conducted across multiple European countries, patients with suspected CAD referred by general practitioners to cardiology centers were randomized to undergo either CCTA or invasive coronary angiography (ICA) to assess diagnostic accuracy and clinical outcomes. Notably, randomization occurred before the clinical assessment at cardiology centers, and 38% of these patients were later found to have nonanginal chest pain, while 36% had atypical angina. These findings highlight how population-based studies may yield misleading conclusions regarding symptom assessment, ultimately leading to inadequate information. In contrast, major cardiology centers frequently encounter a significantly higher prevalence of obstructive CAD in men presenting with typical angina.

Primary and secondary prevention, as well as the diagnosis and treatment of CCS, have been undergoing significant advances, driven by a better understanding of its etiology and pathophysiology, along with the introduction of new or updated diagnostic procedures. Furthermore, clinical management has evolved considerably since the previous guideline.

We hope this guideline proves to be a valuable resource for health professionals in supporting clinical decision-making related to CCS, and we wish all readers a productive and insightful experience.

## 2. Diagnosis

### 2.1. Clinical Evaluation

The initial assessment of patients with suspected CCS is based on a detailed clinical history and physical examination, including the evaluation of comorbidities, risk factors, and impact on quality of life (QoL). These data guide risk stratification and indicate the need for further diagnostic testing.^5^

A thorough clinical history is essential, as angina is the initial symptom in approximately 50% of patients with CCS.^6^ Its presence is associated with a twofold increase in the incidence of major adverse cardiovascular events (MACE) and related health care costs.^7^

Comorbid conditions such as chronic kidney disease (CKD), stroke, and peripheral vascular disease (PVD) should be investigated, as well as classical risk factors: hypertension, smoking, dyslipidemia, diabetes, obesity, physical inactivity, psychosocial stress, and a family history of premature CAD.^8^

Although the physical examination rarely provides specific diagnostic findings, it may reveal indirect signs of CV risk and manifestations of comorbidities. It is also essential for ruling out noncardiac causes of chest pain.

Estimating the pretest probability (PTP) of CAD is a critical step and should be performed using validated clinical algorithms.

Figure 1 provides a schematic illustration of the natural history of CCS.^1^

**Figure 1:** Schematic illustration of the natural history of chronic coronary syndrome. ACS: acute coronary syndrome; ACE: angiotensin-converting enzyme; MACE: major adverse cardiovascular events.

#### 2.1.1. Definition of Angina

Stable angina pectoris has traditionally been considered a manifestation of CAD in stable clinical settings and is almost always associated with obstructive lesions in the coronary tree. However, for several years now, it has been recognized that even individuals with typical angina may not present with obstructive findings on ICA or more recently on CCTA, as previously discussed in the introduction to this section.

The classic description of angina was provided by William Heberden in 1772 as chest discomfort triggered by exertion and relieved with rest.^9^ Today, accurate diagnosis requires a thorough clinical history considering both typical and atypical symptoms. Anginal discomfort is usually located retrosternally or in the precordial area, often radiating to the arms, jaw, or back, and is typically described as tightness, pressure, or burning. It is generally associated with physical exertion, lasts up to 20 minutes, and improves with rest or nitrate use.

Other symptoms, such as dyspnea and fatigue – referred to as "ischemic equivalents" – are common but have limited diagnostic value when they occur in isolation. In patients over 50 years of age, the probability of obstructive CAD associated with isolated dyspnea is approximately 20%-32% in men and 9%-14% in women.^4^ Due to their nonspecific nature, it is recommended to refer to these as "symptoms suggestive of myocardial ischemia" when chest pain is absent.^10^

Angina may result from mechanisms other than epicardial obstruction, including endothelial dysfunction, coronary vasospasm (CVS), microvascular alterations, or myocardial metabolic abnormalities.^11,12^ In a study involving nearly 400,000 patients undergoing elective ICA, only 41% of those with functional ischemia had obstructive CAD.^13^

Such mechanisms may coexist. Atherosclerosis – even when nonobstructive – can trigger ischemia through endothelial dysfunction, which contributes to the pathophysiology of angina.^14^

Angina is a symptom; ischemia is an objective finding on functional testing. Angina may be present without detectable ischemia, and silent ischemia is also common. In such cases, alternative causes of chest pain and dyspnea should be considered. Chart 1 presents the main etiologies of pain in the chest, epigastrium, jaw, and neck.

**Chart 1:** Causes of chest, epigastric, jaw, or neck pain

System	Possible causes
Cardiac	Stable angina, unstable angina, acute infarction, pericarditis, myocarditis
Vascular	Aortic dissection, pulmonary thromboembolism
Pulmonary	Pneumothorax, pneumonia, pleuritis, pulmonary hypertension
Gastrointestinal	Gastroesophageal reflux disease, peptic ulcer, pancreatitis, cholecystitis
Musculoskeletal	Costochondritis, muscle contractures, cervical injuries
Other	Anxiety, herpes zoster (pre-vesicular phase), severe anemia

#### 2.1.2. Clinical Assessment of Patients with Chest Pain

##### 2.1.2.1. Medical History

A detailed clinical history is the primary tool for diagnosing angina.^15^ Chest pain is a common complaint with a broad differential diagnosis, including cardiac, pulmonary, gastrointestinal, and musculoskeletal etiologies.^16^ Symptom characterization is essential to distinguish ischemic from noncardiac causes of chest discomfort.

The evaluation should consider pain location, radiation, quality, duration, triggering and relieving factors as well as associated symptoms.^1^ The terms most frequently used by patients to describe angina include "tightness," "pressure," "burning," or "heaviness." Patients do not always report pain explicitly, but rather describe a general "discomfort." Stabbing pain or pain associated with respiration or changes in position is rarely ischemic.^16^

Typical angina pain is usually located in the retrosternal or precordial area and may radiate to the arms, neck, jaw, or back – most commonly on the left side. It typically lasts a few minutes and is relieved by rest or sublingual nitrates. Sudden and fleeting discomfort, or pain lasting for hours without interruption, is unlikely to be of coronary origin.

Symptoms such as diaphoresis, nausea, presyncope, and dyspnea may accompany the chest pain and are considered anginal equivalents. Isolated dyspnea may represent a manifestation of CAD, but should be interpreted with caution.^1^ Symptoms occurring after meals or in the early morning hours are also common in angina.

The classification of chest pain as typical, atypical, or noncardiac is based on three criteria: location and quality of pain, relationship with physical exertion, and relief with rest or nitrates. The presence of all three criteria defines typical angina; two criteria indicate atypical angina; and one or none characterizes nonanginal chest pain.^17^ Although this classification is subjective, it is useful for estimating the probability of CAD.^18^

Chart 2 presents this classification. Chart 3 describes the severity of angina according to the Canadian Cardiovascular Society. Chart 4 outlines the clinical subtypes of unstable angina – a condition associated with a high risk of progression to acute myocardial infarction (MI).

**Chart 2:** Classification of chest pain based on reported symptoms

Criteria:	
1. Retrosternal discomfort with characteristic quality and duration	
2. Triggered by physical exertion or emotional stress
3. Relief with rest and/or sublingual nitrate within 10 minutes	
**Type of pain**	**Criteria met**
Typical angina	All three criteria present
Atypical angina	Two of the three criteria present
Nonanginal pain	Only one or none of the criteria present

**Chart 3:** Grading of angina severity (Canadian Cardiovascular Society)

Class	Description
I	Angina only with strenuous or prolonged physical exertion
II	Slight limitation with moderate effort (e.g., climbing stairs, brisk walking)
III	Marked limitation with mild effort (e.g., walking one block)
IV	Angina at rest or with any level of physical activity

**Chart 4:** Clinical subtypes of unstable angina

Subtype	Description
New-onset angina	Less than 2 months in duration, CCS Grade III or IV
Worsening angina	Increased frequency, intensity, or lower threshold of exertion
Rest angina	Pain episodes lasting ≥20 minutes, occurring at rest

CCS: Canadian Cardiovascular Society.

Studies have shown that most patients presenting with chest pain suspected to be related to CAD exhibit atypical or nonanginal symptoms. Typical angina accounts for only 10% to 15% of cases.^19–21^

Table 2 summarizes the main clinical aspects to be evaluated in chest pain: type, location, radiation, duration, triggering and relieving factors, and associated symptoms.

**Table 2:** Clinical aspects of chest pain to be evaluated

Parameter	Aspects to be observed
Type of pain	Tightness, pressure, burning, heaviness, or poorly defined discomfort
Location	Retrosternal or precordial; may radiate to arms, jaw, neck, or back
Radiation	Most commonly to the left arm, but may involve both arms, jaw, and back
Duration	Usually lasts 2 to 10 minutes; very brief or prolonged pain (lasting hours) is less suggestive
Triggering factors	Physical exertion, emotional stress, cold exposure, heavy meals
Relieving factors	Cessation of exertion, rest, use of sublingual nitrates
Associated symptoms	Dyspnea, sweating, nausea, sense of impending doom, fatigue, or syncope

Figure 2 shows a representation of common locations and radiation patterns.

**Figure 2:** Common chest regions for angina-related discomfort and radiation patterns.

##### 2.1.2.2. Physical Examination

The physical examination of patients with chronic CAD is often unremarkable but may reveal indirect signs of atherosclerotic risk or evidence of myocardial ischemia.^22^ Clinical findings can aid in both risk stratification and differential diagnosis.

Ocular changes, such as a corneal arcus (arcus senilis), particularly in patients under 40 years of age, suggest dyslipidemia.^23^ Fundoscopic examination may reveal retinal changes associated with hypertension and diabetes.^24^

The presence of cutaneous or tendon xanthomas is indicative of genetic dyslipidemias, such as familial hypercholesterolemia, which is frequently associated with a family history of premature CAD.^25^ Frank's sign (diagonal earlobe crease) may be associated with CAD and peripheral artery disease (PAD), and it commonly becomes bilateral with advancing age.^26^

The coexistence of carotid or PVD increases the likelihood of CAD. Therefore, palpation and auscultation of the cervical and femoral arteries should be part of the physical examination. Hypertension must be actively assessed due to its relevance as a major risk factor.

The physical examination also contributes to the differential diagnosis and may suggest conditions such as hypertrophic cardiomyopathy or heart valve disease, both of which can cause noncoronary angina.

##### 2.1.2.3. Differential Diagnosis of Chest Pain

Several clinical conditions may cause chest pain resembling angina, particularly due to an imbalance between myocardial oxygen supply and demand. These situations must be recognized, especially in patients with noncritical coronary lesions (Figure 3).^16,27^

**Figure 3:** Common locations of chest discomfort/pain and their causes.

Clinical states associated with increased oxygen demand include fever, hyperthyroidism, cocaine use, tachyarrhythmias, and emotional stress. Cocaine use has become an increasingly common cause of acute coronary syndrome (ACS) in young patients, owing to its potent vasoconstrictive effects and associated adrenergic surge.^28,29^ Hyperthyroidism in turn increases metabolic demand and myocardial oxygen consumption.

Chronic arterial hypertension, particularly when poorly controlled, increases left ventricular (LV) wall stress and may lead to subendocardial ischemia. A similar mechanism is observed in aortic stenosis and hypertrophic cardiomyopathy, where marked hypertrophy and reduced coronary reserve are present.^30,31^

Chart 5 lists cardiac conditions that precipitate angina by increasing oxygen demand or reducing oxygen supply. Chart 6 summarizes noncardiac causes of chest pain that should be considered in the differential diagnosis.

**Chart 5:** Cardiac conditions that may precipitate ischemia

Condition	Likely mechanism
Aortic stenosis	Increased afterload and reduced subendocardial perfusion
Severe aortic regurgitation	Decreased diastolic pressure and coronary blood flow
Hypertrophic cardiomyopathy	Hypertrophy with intramyocardial compression
Arrhythmias (tachyarrhythmias)	Increased oxygen consumption
Severe hypertension	Left ventricular overload and increased demand
Heart failure	Reduced cardiac output and coronary reserve

**Chart 6:** Noncardiac causes of chest pain

Category	Examples
Gastrointestinal	Gastroesophageal reflux disease, peptic ulcer, esophagitis, cholecystitis, pancreatitis
Pulmonary	Pulmonary embolism, pneumonia, pneumothorax, pleuritis
Musculoskeletal	Costochondritis, muscle strain, rib fractures
Psychogenic	Anxiety, panic disorder
Other	Herpes zoster (early phase), severe anemia, hyperviscosity syndrome

#### 2.1.3. Laboratory Assessment

The laboratory assessment for coronary syndrome includes the evaluation of ischemic disease, risk factors, and metabolic abnormalities that may contribute to the progression of arterial obstruction. Laboratory testing should also monitor parameters whose abnormal levels are associated with worse prognosis (Table 3).

**Table 3:** Laboratory tests for evaluating patients with suspected chronic coronary syndrome*

Recommendation	Recommendation grade	Level of evidence
Perfil lipídico, incluindo LDL-C	**I**	**A**
Hemograma completo, incluindo valores de hemoglobina	**I**	**B**
Recomenda-se que a triagem para a investigação de diabetes nos pacientes com síndrome coronariana crônica seja realizada utilizando a glicose de jejum e HbA1c, com adição do teste de tolerância caso os resultados sejam inconclusivos	**I**	**B**
Medidas de creatinina e estimativa da função renal	**I**	**A**
Avaliação da função tireoidiana	**I**	**C**

LDL-C: colesterol de lipoproteínas de baixa densidade; HbA1c: hemoglobina glicada.

* Adaptado das Diretrizes da Sociedade Europeia de Cardiologia para o diagnóstico e tratamento das síndromes coronarianas crônicas.

Anemia, thyroid disorders, diabetes, and kidney disease are noncardiac conditions that should be assessed in the initial workup, as they are associated with adverse outcomes in CAD. Recommended tests include complete blood count, TSH and free T4, glucose, glycated hemoglobin (HbA1c), creatinine, and estimated glomerular filtration rate (GFR) (eGFR).^1^

Anemia is associated with MACE and may therefore be considered a marker of poor prognosis in patients with CVD. A subanalysis of the TIME (Trial of Invasive vs Medical Therapy in Elderly Patients) trial demonstrated a correlation between hemoglobin reduction and increased risk of CV mortality.^32^

Evidence also supports an association between subclinical hypothyroidism and CV risk. The Rotterdam Study found a cross-sectional association between subclinical hypothyroidism and atherosclerosis, measured by abdominal aortic calcification and the prevalence of acute MI.^33^ Approximately 10-15% of patients with hypothyroidism develop some form of arrhythmia.^34^ In addition, hypothyroidism is a secondary cause of dyslipidemia.^35^ Statin therapy is not contraindicated in these individuals; however, initiation of statins should occur only after normalization of thyroid hormone levels due to the increased risk of myositis.^36^

Understanding glucose metabolism is essential due to the well-established association between diabetes and CV complications. The increased risk of CAD is directly proportional to rising serum glucose levels. Values near the diagnostic threshold for diabetes (126 mg/dL) already confer a significantly elevated risk. Another biomarker of hyperglycemia, HbA1c, should preferably be evaluated on an individual basis. For most adults, the recommended treatment target is to maintain HbA1c levels below 7%.^38^

Renal dysfunction negatively impacts the prognosis of CAD. Serum creatinine alone is insufficient to assess renal function, especially in patients with mild to moderate kidney impairment.^38^ Creatinine production is widely recognized to be influenced by factors such as age and sex in clinical practice. International organizations – including the American Society of Nephrology–National Kidney Foundation (ASN-NKF) Task Force – have endorsed several formulas and algorithms that incorporate these variables, along with serum creatinine levels, to estimate GFR.^39^

A lipid profile – including total cholesterol, low-density lipoprotein cholesterol (LDL-C), high-density lipoprotein cholesterol (HDL-C), and triglycerides (TGs) – should be assessed in patients with suspected CAD to determine CV risk and guide the need for treatment. Since the 2016 recommendation by the Brazilian Consensus for the Normalization of Laboratory Determination of Lipid Profile,^40^ which allowed for fasting flexibility by identifying patient pre-analytical condition at the time of sample collection has become critical for accurately estimating CV risk.

In addition to the previously mentioned variables, troponin has also been evaluated as a risk marker in these patients. The sensitivity of troponin assays has significantly improved since their initial introduction in the 1990s. As a consequence, their specificity for acute MI has substantially decreased. Elevated troponin concentrations are often observed in noncardiac conditions such as renal failure, sepsis, pulmonary embolism, and chemotherapy-induced cardiac injury. There are several hypotheses regarding how troponin is released into the bloodstream in patients with reversible myocardial ischemia and in those with cardiac injury unrelated to ischemia. The mechanisms underlying these processes are currently under review.^41^ Studies have shown that elevated troponin levels – whether associated with CV or nonCV comorbidities – are predictive of major adverse events in hospitalized patients, even when no definitive diagnosis has been established.^42^

Moreover, the ARIC (Atherosclerosis Risk In Communities) Study^43^ found that elevated troponin concentrations were independently associated with an increased incidence of CVDs in the general population, regardless of traditional risk factors. These findings support the need for special attention to patients with elevated troponin levels.

### 2.2. Noninvasive Testing

#### 2.2.1. Exercise Stress Test

Due to its wide availability and low cost, exercise stress test (EST) is a readily accessible method with several indications in patients with known or suspected chronic CAD. EST is used for diagnostic confirmation, prognostic assessment, support in clinical management, and also for exercise prescription and enrollment in cardiac rehabilitation programs (Table 4).^1,44,45^

**Table 4:** Recommendations for EST in the initial diagnostic management of patients with suspected chronic coronary syndrome*

Recommendation	Recommendation grade	Level of evidence
EST is recommended in selected patients* to assess exercise tolerance, symptoms, arrhythmias, BP response, and risk stratification.^1^	**I**	**C**
May be considered as an alternative to confirm or exclude CAD when noninvasive imaging modalities are unavailable.^1^	**IIb**	**B**
May be considered to refine risk stratification and guide treatment decisions.^46^	**IIb**	**B**
Not recommended for diagnostic purposes in patients with resting ST-segment depression ≥1 mm, left bundle branch block, or current use of digitalis.	**III**	**C**

BP: blood pressure; CAD: coronary artery disease; EST: exercise stress test.

* When there is a reasonable expectation that EST will impact the diagnostic strategy or clinical management.

** For more specific indications and recommendations, refer to the exercise testing guideline, which includes subpopulations not covered in this chronic coronary syndrome guideline.^45^

Interpretation of EST should include electrocardiographic (ECG), clinical, hemodynamic, autonomic, and functional capacity (FC) variables, as they provide prognostic information relevant to patient follow-up and potential changes in clinical management for those with CAD. In the ECG evaluation during exertion, horizontal or downsloping ST-segment depression ≥ 1 mm measured 0.08 m/s after the J point is the most predictive alteration for ischemia. The magnitude, early onset, number of leads involved, and time to normalization during recovery are all indicators of more severe ischemic response. Asymptomatic ST-segment depression during EST in middle-aged men with CV risk factors has been shown to predict sudden cardiac death (SCD) and coronary death, with a 2- to 2.5-fold higher risk compared to those without ST changes. ST depression during the recovery phase, even when asymptomatic, carries similar diagnostic value but has an even greater prognostic impact – associated with 3- to 4-fold increases in SCD and coronary death, respectively.^47^ ST-segment elevation in leads without inactive myocardium is the most severe ECG alteration during EST and indicates transmural ischemia. Although rare (0.1%), it is associated with a high risk of serious arrhythmias such as ventricular tachycardia (VT) and ventricular fibrillation (VF). Like MI, it also helps localize the affected coronary artery.^45^ The combination of progressively worsening anginal pain and a drop in systolic blood pressure (BP) reinforces the diagnosis and suggests higher severity. Importantly, fewer than 50% of ischemic ESTs are accompanied by clinical angina. An ECG is considered uninterpretable for diagnostic purposes during EST in the presence of left bundle branch block (LBBB), artificial pacemaker, ventricular pre-excitation, resting ST-segment depression ≥ 1.0 mm, or digitalis use, as these factors may alter the ST segment in the absence of CAD.^45^ In such cases, imaging-based stress testing is recommended. Prognostic scores: Among the various prognostic tools, the Duke Treadmill Score (DTS) is the most widely used. DTS incorporates EST variables to stratify CAD risk and can be used for both diagnostic and prognostic purposes.^48^

A flattened or progressively declining systolic BP curve during exercise, especially when followed by a paradoxical increase in the first minute of recovery, is associated with severe CAD, as it reflects impaired ventricular function.^49,50^ FC estimated in metabolic equivalents of task (METs) during an EST is the most powerful prognostic variable established in the literature. Low FC at peak exertion is associated with increased all-cause and CV mortality across different populations, including men, women, individuals with risk factors, and patients with established CAD. A progressive decline in FC in patients with established CAD is also predictive of adverse events, in accordance with MET-based thresholds.^51^ Among men with CV risk factors, an FC <5 METs is associated with a 4- to 4.5-fold increase in 6-year mortality. In contrast, achieving > 10 METs predicts excellent prognosis, regardless of risk factors.^51^ In women, an FC < 5 METs is associated with threefold higher mortality compared to those who achieve > 8 METs, and between 5-8 METs, the risk is doubled – independent of the presence of ST-segment depression or other risk factors.^52,53^ Moreover, the assessment of FC is essential in patients with established CAD to guide exercise prescription – particularly to identify ischemic thresholds (clinical and/or ECG-based when ischemia is present) – and to adjust CV rehabilitation programs accordingly. Table 1 summarizes the main recommendations for the use of EST in the initial diagnostic management of patients with suspected CCS. For more detailed information, refer to the Brazilian guideline on exercise testing in adult populations.^45^

#### 2.2.2. Echocardiography

##### 2.2.2.1. Transthoracic Echocardiography

Transthoracic echocardiography (TTE) is the first-line imaging modality for evaluating patients with either subclinical or overt CAD. This method enables the assessment of global and segmental LV systolic function, LV diastolic function, complications related to chronic CAD, and the identification of alternative causes of chest pain and dyspnea, which may coexist with CAD (Table 5).^1,54,55^ Global systolic function is commonly evaluated by measuring the LV ejection fraction (LVEF), which can be quantified using the biplane disk summation method (Simpson's rule) in two-dimensional imaging or, when available, through three-dimensional (3D) echocardiography, which provides greater accuracy by avoiding geometric assumptions. Segmental LV function is estimated using the wall motion score index (WMSI).^16^ As a volumetric index, LVEF, and as a visual index, WMSI, may not always accurately reflect myocardial contractility. More recently, global longitudinal strain (GLS), derived from speckle-tracking echocardiography, has emerged as a more sensitive, accurate, and reproducible marker of subclinical dysfunction (GLS < 16%) in both acute^56^ and chronic^1,54^ CAD. Additionally, regional reductions in strain – visualized through polar maps or strain curves – may suggest ischemia or fibrosis more accurately than WMSI.^57,58^

**Table 5:** Recommendations for the use of transthoracic echocardiography in the initial diagnostic evaluation of individuals with suspected chronic coronary syndrome

Recommendation	Recommendation grade	Level of evidence
Assess global and segmental LV function, volumes, diastolic function, RV function, estimate pulmonary artery systolic pressure, refine risk stratification, and guide treatment.	**I**	**B**
Evaluate differential or coexisting diagnoses of chest pain or dyspnea such as valvular disease, hypertrophy, cardiomyopathy, and pericardial effusion.	**I**	**B**
Assess global longitudinal strain to evaluate global and regional LV systolic function when LVEF is normal and clinical probability of CAD is high.*	**IIa**	**B**
Routine re-evaluation of stable patients without clinical change.	**III**	**C**

CAD: coronary artery disease; LV: left ventricle; LVEF: LV ejection fraction.

* Global longitudinal strain should be interpreted comparatively, within a clinical context, using the same vendor and software version, and under similar loading conditions.

##### 2.2.2.2. Stress Echocardiography

Stress echocardiography is a well-established method for diagnosing suspected or known obstructive CAD. In addition to its diagnostic value, it provides important prognostic information, assesses myocardial viability, and evaluates the impact of revascularization therapies (Table 6).^59,60^ CV stress induces myocardial ischemia in regions supplied by coronary arteries with significant stenosis. This ischemic cascade typically manifests in sequence: myocardial perfusion abnormalities, segmental contractility alterations, ECG changes, and finally, the onset of angina.^61–64^ Stress may be induced by physical exercise (treadmill or cycle ergometer), vasodilator drugs (e.g., dipyridamole with or without atropine), or adrenergic stimulants (e.g., dobutamine with or without atropine). All these modalities demonstrate high diagnostic accuracy (85% to 90%) for detecting coronary obstructions in patients with intermediate or high PTP.^65,66^ A normal stress echocardiogram carries a high negative predictive value (93% to 100%) for future MACE.

**Table 6:** Recommendations for the use of stress echocardiography in suspected chronic coronary syndrome

Recommendation	Recommendation grade	Level of evidence
Investigation of ischemia and estimation of the risk of cardiovascular events in symptomatic individuals with intermediate or high pretest probability	**I**	**B**

Myocardial contrast echocardiography (MCE), using the infusion of ultrasound contrast agents^59,66^ should be performed at rest or during stress in patients with technically challenging studies to improve endocardial border delineation.^74–76^ These agents allow for more accurate measurements of ventricular volumes and LVEF (Figure 4), detection of intracavitary thrombi (Figure 5) and enhanced assessment of segmental contractility.^66,67^ Their use increases the proportion of conclusive diagnostic studies.^67^ In patients with normal LV function, several studies have also demonstrated diagnostic and prognostic enhancement when MCE is used for qualitative and quantitative assessment of myocardial perfusion, both at rest and under stress^69^ (Figure 6). For further detail, we recommend referring to SBC guidelines on indications for adult echocardiography.^55^

**Figure 4:** Differences in end-diastolic and end-systolic volumes observed in the same patient without contrast (top) and with UEAs and low MI imaging (bottom). Top row, left to right: Pre-contrast quantification of LV end-diastolic volume (306 mL) and end-systolic volume (246 mL) for LVEF estimation. Bottom row, left to right: Post-contrast quantification of LV end-diastolic volume (391 mL) and end-systolic volume (308 mL) for LVEF estimation. A marked increase in measured volumes is observed following contrast administration. LV: left ventricle; LVEF: LV ejection fraction; MI: myocardial infarction; UEA: ultrasound enhancing agent.

**Figure 5:** Apical three-chamber view. In (A), the lateral wall and apex are not visualized. In (B), contrast enhancement allows proper delineation of previously nonvisualized segments and reveals the presence of an apical thrombus.

**Figure 6:** Apical four-chamber view from two pharmacologic stress studies in the same patient using microbubble contrast to assess myocardial wall motion and perfusion. (A) Normal perfusion at rest. (B) Transmural perfusion defect in a single apical segment during adenosine stress. (C) Extensive transmural apical perfusion defect involving at least two segments, associated with wall motion abnormality (akinesia) in the same territory during dobutamine stress. (D) Acoustic intensity-time curves generated by dedicated software, demonstrating severely impaired coronary flow reserve under both stress protocols. (E) Coronary angiography in left anterior oblique view showing severe stenosis in the left anterior descending, circumflex, and marginal arteries.

#### 2.2.3. Nuclear Medicine

Noninvasive imaging modalities play a fundamental role in the diagnosis and management of ischemic heart disease (IHD).

Nuclear cardiology is a physiological imaging modality that uses radiopharmaceuticals to study the pathophysiological mechanisms of CVDs. Nuclear cardiology enables the assessment of the entire spectrum of IHD – from obstructive epicardial CAD to microvascular disease (MVD)^70^ – and extends beyond perfusion and ventricular function to include metabolism, innervation, and mechanical synchrony^71^ (Figure 7).

**Figure 7:** Applications of nuclear cardiology in ischemic heart disease.

Myocardial perfusion scintigraphy (MPS) using single-photon emission computed tomography (SPECT) has been employed for decades in clinical practice due to its wide availability and the extensive literature supporting its value in the diagnosis and risk stratification of IHD.^72,73^ MPS uses the radiopharmaceuticals sestamibi or tetrofosmin labeled with technetium-99m (^99m^Tc) to assess ischemia and, less frequently and in specific cases, thallium-201 (^201^Tl) to evaluate myocardial viability.^20,74–78^

MPS is the most commonly used noninvasive imaging test for the evaluation of patients with suspected or known chronic IHD. MPS can be successfully performed in virtually all patient groups, regardless of renal function, presence of arrhythmias, obesity, or intracardiac devices – conditions that often pose challenges for alternative diagnostic techniques^72,77^ (Figure 8).

**Figure 8:** Potential applications of nuclear techniques. IHD: ischemic heart disease.

MPS using SPECT can provide, in a single examination, images of relative myocardial perfusion, primarily assessing the extent and severity of myocardial ischemia. MPS can quantify the perfusion deficit as a percentage of the LV and evaluate ventricular volumes and LVEFs at rest and after stress, among other markers (Figure 9).

**Figure 9:** MPS GATED-SPECT: large-extent stress-induced ischemia. A large area of hypoperfusion is observed, involving the anterior wall, septal region, and apex, extending distally to the inferior and lateral walls. This is visualized in blue on the stress polar map (PM) (upper left image), delineated by a white contour line. The PM representing rest perfusion shows a homogeneous distribution of the radiotracer, indicating normal perfusion. The graphical representation of stress scores (black arrows) shows the numerical grading of hypoperfusion intensity and the number of affected segments (bottom right corner), with summed scores of SSS = 25, SRS = 0, and SDS = 25 (top right corner), along with an estimated myocardial ischemic burden of 45% of the left ventricle. Invasive coronary angiography revealed a proximal subocclusive lesion in the anterior descending artery. MPS GATED-SPECT: myocardial perfusion scintigraphy gated single photon emission computed tomography; SDS: summed difference score; SRS: summed rest score; SSS: summed stress score. Source: Personal archive.

MPS is performed in two phases: rest and CV stress, which may be either physical or pharmacological. Physical stress (via exercise testing or cardiopulmonary exercise testing) should be the preferred method when the patient is capable of exercising adequately (achieving a metabolic workload ≥5 METs), as it provides important additional diagnostic and prognostic information beyond imaging. Pharmacological stress is reserved for patients with physical limitations, formal contraindications to exercise testing, LBBB, or intracardiac devices, among other conditions.^72,77^

Symptomatic patients with intermediate to high risk of IHD benefit the most from MPS. The main indications and levels of evidence for functional imaging in nuclear cardiology are presented in Table 7.^1,44,74,79,80^

**Table 7:** Recommendations for stress and rest MPS in the initial diagnostic management of patients with suspected chronic coronary syndrome

Recommendation	Recommendation grade	Level of evidence
In patients with suspected chronic coronary syndrome and moderate to high pretest probability of obstructive CAD, stress SPECT or, preferably, perfusion imaging by PET is recommended to: diagnose and quantify myocardial ischemia and/or scar; estimate the risk of cardiovascular events; quantify myocardial blood flow (PET).	**I**	**B**

CAD: coronary artery disease; MPS: myocardial perfusion scintigraphy; PET: positron emission tomography; SPECT: single-photon emission computed tomography.

A disadvantage of MPS is radiation exposure, although more recent technologies – such as new gamma cameras with cadmium-zinc-tellurium (CZT) solid-state detectors, improvements in radiopharmaceuticals, updated protocols, and individualized tracer doses – have helped reduce this exposure.^73^

##### 2.2.3.1. Clinical Decision-making after Radionuclide Perfusion Studies – the Impact of Ischemic Burden on Cardiovascular Outcomes^81–87^

MPS has a well-established evidence base demonstrating the high negative predictive value of a normal scan, with low CV risk over varying follow-up periods depending on the study population, regardless of sex. The prognostic value of a normal MPS is excellent, with a 99% event-free survival rate according to a large meta-analysis.^88^ Likewise, the probability of major adverse events (CV and all-cause death, nonfatal MI) increases exponentially with greater ischemic burden (extent and severity of perfusion defects). These perfusion findings should be integrated with information on ventricular function, which adds incremental prognostic value when reduced (Figure 10).

**Figure 10:** Adjusted rate of cardiac death or myocardial infarction in female and male patients referred for myocardial perfusion scintigraphy according to the percentage of ischemia and left ventricular systolic function (adapted from Sharir et al.87). LVEF: left ventricular ejection fraction.

Classic and robust observational studies^82,89^ have shown that the extent of ischemia is one of the most important predictors of the benefit of myocardial revascularization. These studies did not demonstrate a reduction in CV outcomes in the absence of ischemia or in the presence of mild ischemia, but they did show evidence of benefit when the ischemic burden exceeds 10%-12% of the LV (Figure 11).

**Figure 11:** Cardiac mortality rate stratified by ischemia quantification (SPECT) and treatment modality (adapted from Hachamovitch et al.^90^). OMT: optimal medical therapy; Revasc.: revascularization; SPECT: single-photon emission computed tomography.

Contrary to observational studies,^81,82,88,90–92^ the ISCHEMIA (International Study of Comparative Health Effectiveness with Medical and Invasive Approaches) trial^93^ did not demonstrate a reduction in established events when comparing myocardial revascularization versus optimal medical therapy (OMT) in patients with at least moderate ischemia, as assessed by noninvasive functional tests, over an initial follow-up period of 3.2 years. Importantly, SPECT imaging was not the sole method used to assess ischemia for patient inclusion; approximately 25% of patients were enrolled based on high-risk EST results without imaging.

These findings sparked intense debate in the medical community due to the potential shift in a long-standing paradigm for the treatment of CAD. However, ISCHEMIA subanalyses suggest that in patients with myocardial ischemia, heart failure (HF), and an LVEF between 35% and 45%, an early invasive strategy may improve event-free survival.^93^ Moreover, the mortality curves in the main trial began to diverge after 2 years of follow-up, showing an apparent benefit for revascularization, with possible long-term implications. This led to the extension of clinical follow-up and the recent publication of the ISCHEMIA-EXTEND study. With a median follow-up of 5.7 years, the extended analysis showed no difference between treatment groups in all-cause mortality, but did reveal lower CV mortality in the early invasive strategy group over seven years, despite paradoxically higher nonCV mortality.^94^

In any case, ISCHEMIA was a major therapeutic evaluation study and was never intended to challenge the diagnostic approach or risk stratification in patients with suspected or known CAD. Risk stratification remains a cornerstone in guiding individualized clinical decision-making and appropriate management of IHD.

Finally, the role of nuclear cardiology in assessing myocardial viability should be noted. The gold standard is 18F-FDG positron emission tomography (PET)/CT using fluorine-18-labeled glucose to evaluate glycolytic metabolism. SPECT with thallium-201 can also be used when PET/CT is not available.^44,95–99^

##### 2.2.3.2. PET

PET is considered the gold standard for the noninvasive assessment of myocardial perfusion. Although not available in the country due to the lack of radiopharmaceuticals – Rubidium-82 (^82^Rb), Ammonia-N13 (^13^N-Ammonia), or Oxygen-15 water (^15^O-H_2_O) – it deserves emphasis in CAD, given its ability to noninvasively and absolutely quantify myocardial blood flow (MBF) during stress and rest, as well as to determine coronary flow reserve (CFR). Such data provide valuable insights to refine diagnosis, prognosis, and patient management, covering the spectrum of CAD, from multivessel epicardial CAD to diffuse coronary microvascular dysfunction (CMD) (Figures 12 and 13).

**Figure 12:** Modalities for the assessment of obstructive and nonobstructive coronary artery disease. CAD: coronary artery disease; CCTA: coronary computed tomography angiography; CFR: coronary flow reserve; CMR: cardiovascular magnetic resonance; CZT: cadmium, zinc, and tellurium; EST: exercise stress test; ICA: invasive coronary angiography; PET: positron emission tomography; SPECT: single-photon emission computed tomography. Adapted from Koilpillai et al.^100^

**Figure 13:** PET/CT perfusion imaging: comprehensive information. CCTA: coronary computed tomography angiography; LVEF: left ventricular ejection fraction; PET/CT: positron emission tomography/computed tomography. Adapted Cheng et al.^21^

The ESC 2019 guidelines for the diagnosis and management of CCS categorizes chronic CAD phenotypes into three groups and highlights the importance of assessing coronary MVD. They emphasize that the angiographic exclusion of epicardial stenosis no longer represents the last step in the diagnostic process. The guidelines also include PET as a noninvasive method for evaluating CFR in patients with suspected coronary microvascular angina.^97–103^

Microvascular angina is diagnosed after ruling out epicardial coronary stenosis and carries an adverse prognosis. Microvascular dysfunction precedes the development of epicardial lesions, particularly in women,^104^ and is associated with worse outcomes. The presence of abnormal CFR is linked to a higher risk of MACE even in patients without significant coronary stenosis, especially among individuals with diabetes.^81,105^ Thus, the noninvasive detection of CAD in its subclinical stage allows the identification of patient subgroups at risk for future cardiac events, who may benefit from early and aggressive preventive strategies. Moreover, the response to preventive medications can be monitored through serial CFR measurements, which may show favorable changes following appropriate interventions.^106–109^

##### 2.2.3.3. Coronary Flow Reserve Using Single-photon Emission Computed Tomography with Cadmium-zinc-tellurium

The advent of new SPECT CZT systems enables dynamic acquisitions for the absolute quantification of coronary blood flow (at rest and under stress conditions) and the calculation of CFR using technetium-99m-labeled radiotracers, which are widely available both internationally and locally. Thus, the availability of these radiotracers, the lower cost compared to PET/CT, and the faster image acquisition make the quantification of MBF and CFR a more feasible tool within our current clinical setting.

Several studies have shown good correlation between MBF and CFR values estimated using technetium-99m-labeled tracers with SPECT CZT and those measured by PET/CT imaging with 15O-water or 13N-ammonia as perfusion markers.

Although highly promising, most studies to date come from single centers and involve small sample sizes. Nonetheless, this technology has garnered significant interest from the scientific community, given the wide availability of the radiopharmaceuticals and the lower cost of the equipment compared to PET/CT.

#### 2.2.4. Laboratory Assessment

Laboratory investigation in stable CAD aims to identify modifiable risk factors and associated conditions that increase CV risk. Recommended tests include a lipid profile, fasting glucose, HbA1c, renal function (urea, creatinine, and estimated creatinine clearance), complete blood count, and TSH (Table 8).^25^

**Table 8:** Laboratory tests for the evaluation of patients with suspected chronic coronary syndrome*

Recommendation	Recommendation grade	Level of evidence
Lipid profile, including LDL-C	**I**	**A**
Complete blood count, including hemoglobin levels	**I**	**B**
Screening for diabetes in patients with chronic coronary syndrome is recommended using fasting glucose and HbA1c, with the addition of an oral glucose tolerance test if results are inconclusive	**I**	**B**
Creatinine measurement and estimation of renal function	**I**	**A**
Assessment of thyroid function	**I**	**C**

HbA1c: glycated hemoglobin; LDL-C: low-density lipoprotein cholesterol.

* Adapted Knuuti et al.^1^

The lipid profile should include total cholesterol, HDL, LDL (calculated or direct), and TGs. Apolipoprotein B (apoB) and lipoprotein(a) [Lp(a)] may be useful in very high-risk patients or those with a family history of premature CAD,^110^ which is genetically inherited, confers an independent risk for atherothrombotic events and is not influenced by diet or lifestyle.^111^

Assessment of renal function is essential, as a GFR < 60 mL/min/1.73 m^2^ is associated with increased CV risk. In patients with hypertension, screening for microalbuminuria may be useful.

Measurement of natriuretic peptides (BNP or NT-proBNP) may be useful in cases of suspected LV dysfunction, especially in patients presenting with dyspnea. High-sensitivity troponin may also be used as a prognostic marker in selected cases, even outside the context of ACS.^112,113^

Other tests include uric acid (which is associated with increased CV risk when elevated), high-sensitivity C-reactive protein (hs-CRP), and, in specific cases, genetic markers or extended metabolic profiling.

#### 2.2.5. Coronary Computed Tomography

Coronary arteries can be assessed by computed tomography in two ways: by evaluating the presence of coronary calcium and by performing luminography with contrast administration, allowing for the detection of atheromatous plaques and estimation of the degree of obstruction when present. This method also enables the calculation of the coronary calcium score.

##### 2.2.5.1. Coronary Computed Tomography for Coronary Calcium Scoring

Noncontrast coronary CT for calcium scoring is extremely useful in evaluating asymptomatic patients at moderate risk, and in supporting decisions about initiating anti-atherosclerotic therapy for primary prevention. It is a simple method, does not require contrast, and image acquisition takes less than 10 minutes – or even less on newer scanners. Its use should be encouraged, and this guideline provides an opportunity to formally include this exam as a highly valuable tool. It is particularly relevant for assessing low- to moderate-risk asymptomatic individuals since the presence or absence of coronary calcium significantly alters the estimated risk of coronary ischemic events. It is also far more specific than detecting the presence or absence of carotid plaques. A comprehensive review of this method is available in the ESC guidelines on calcium scoring.^114^

A summary of the main indications is provided below, according to the aforementioned guideline (Table 9).

**Table 9:** Recommendations for Coronary CT for Coronary Calcium Scoring

Recommendation	Recommendation grade	Level of evidence
Risk re-stratification in asymptomatic patients with intermediate clinical risk score	**I**	**A**
Risk re-stratification in asymptomatic patients with diabetes mellitus or metabolic syndrome and intermediate clinical risk score	**I**	**B**
Risk re-stratification in asymptomatic patients with intermediate clinical risk score to guide primary preventive pharmacotherapy		
Risk re-stratification in asymptomatic patients with heterozygous familial hypercholesterolemia		
Risk re-stratification in asymptomatic patients with low clinical risk score and a family history of premature CAD	**IIa**	**B**
Screening for myocardial ischemia in asymptomatic patients with diabetes mellitus		
Use of coronary calcium scoring to improve risk stratification in patients without known CAD and with negative ischemia testing		
To rule out significant coronary stenosis in symptomatic patients with suspected stable angina or acute coronary syndrome	**III**	**B**
Use in patients with known obstructive CAD	**III**	**C**

CAD: coronary artery disease.

##### 2.2.5.2. Coronary Computed Tomography Angiography

CCTA is a noninvasive method used to assess the arteries that supply the myocardium. It enables the analysis of the vessel lumen, vessel walls, and, more recently, the characteristics of atheromatous plaques that may compromise these arteries.^115^ Since its early clinical application, this technique has stood out for its high negative predictive value, proving effective in safely ruling out the presence of obstructive CAD.^116,117^ However, as technology and clinical experience with this method have advanced, its positive predictive value has also increased – especially when combined with noninvasive assessment of CFR – giving it strong clinical potential.^115,118^

The main contribution of CCTA is observed in patients with an intermediate PTP of obstructive CAD. Furthermore, newer models used to refine risk estimation are more accurate in identifying the presence of coronary atherosclerosis, in contrast to older models that tend to overestimate the likelihood of significant stenosis.^1,119–121^ As a result, some authors recommend calculating the pretest risk estimate before ordering CCTA when evaluating suspected CCS.^122^

Moreover, some researchers suggest using the coronary calcium score in patients with chest pain and low clinical risk before proceeding with further testing. In population-based trials, most adverse events occurred in individuals with detectable coronary calcium – 84% of MACE in patients with a score > 0 in the PROMISE (Prospective Multicenter Imaging Study for Evaluation of Chest Pain) trial.^1,120,121^ Furthermore, this finding also holds true for asymptomatic individuals, reinforcing its role in primary prevention.

On the other hand, studies have shown that significant coronary obstructions may occur even in the absence of arterial calcification. Therefore, this criterion should be applied with caution, especially in patients under moderate-to-high CV risk.^122^

Further details and nuances on this topic can be found in the recently published CV CT and magnetic resonance imaging (MRI) guideline.^123^ The indications for CCTA in patients with low-risk CCS are presented in Table 10.

**Table 10:** Levels of evidence for computed tomography indications in patients with suspected chronic coronary syndrome and low pretest risk

Recommendation	Recommendation grade	Level of evidence
Diagnosis of coronary artery disease in patients with inconclusive or conflicting functional test results, as an alternative to invasive study	**IIa**	**B**
As a first-line diagnostic option in patients with chest pain	**IIb**	**B**
To estimate atherosclerotic burden in patients without a prior diagnosis of coronary artery disease	**IIb**	**B**

In patients under intermediate or intermediate-to-high CV risk, CCTA achieves its best performance.^118,122^ The assessment of fractional flow reserve (FFR) derived from CT (FFR-CT) has generated great interest, as it allows both anatomical and functional evaluation from a single acquisition.^117^ Its utility has been confirmed by randomized studies and a recent meta-analysis, though its main current limitations remain broad availability and cost.^114,117,123^

Finally, the SYNTAX-III Revolution trial demonstrated that CT can also assist in identifying which patients are better candidates for percutaneous or surgical revascularization. It showed that including FFR-CT analysis is crucial for this purpose. If future studies confirm these findings, the role of CT may become even more significant.^117,124^

Table 11 summarizes the indications for CT in patients under intermediate or intermediate-to-high CV risk.

**Tabela 11:** Levels of evidence for computed tomography indications: patients with suspected chronic coronary syndrome and intermediate or intermediate-high pretest risk

Recommendation	Recommendation grade	Level of evidence
As the initial test for diagnosis or risk stratification of obstructive coronary artery disease in patients without prior anatomical diagnosis	**I**	**A**
Diagnosis of coronary artery disease in patients with inconclusive or conflicting functional test results, as an alternative to invasive study	**I**	**A**

#### 2.2.6. Cardiovascular Magnetic Resonance Imaging

Cardiovascular magnetic resonance (CMR) assesses multiple parameters of IHD, including detection of ischemia, presence of fibrosis/necrosis from MI, and determination of myocardial viability. CMR also demonstrates high accuracy and reproducibility in evaluating global and segmental biventricular function, regardless of ventricular geometry or patient characteristics. For further details, refer to the guideline. In the post-MI setting, such as in the presence of aneurysms or pseudoaneurysms, CMR accurately detects chamber volumes and geometry, as well as the infarcted area. It is often an essential method for assessing cardiac function and ventricular anatomy after myocardial revascularization.^123,125,126^ Therefore, CMR is the appropriate method for evaluating contractility and both global and segmental ventricular function, being the gold standard for this purpose.^80^

CMR with pharmacological stress offers technical advantages that result in greater diagnostic accuracy when compared to myocardial scintigraphy and stress echocardiography. Its high spatial resolution allows for the identification of subendocardial perfusion defects.

##### 2.2.6.1. Assessment of Myocardial Ischemia by Cardiovascular Magnetic Resonance Imaging

The presence of myocardial ischemia can be detected through first-pass perfusion under pharmacological stress (usually dipyridamole or adenosine) or by assessing contractility using incremental doses of dobutamine to induce ischemia. The former approach offers greater sensitivity, while the latter provides higher specificity.^127^

The most common method for evaluating IHD using CMR is myocardial perfusion imaging. Perfusion images are acquired during pharmacological stress and at rest following gadolinium infusion to identify hypoperfused areas. Vasodilator stress agents such as dipyridamole and adenosine induce significant hyperemia across the entire coronary microcirculation, equalizing microvascular resistance in all myocardial territories. As a result, myocardial perfusion becomes proportional to the cross-sectional area and resistance of the epicardial coronary arteries.

In myocardial regions supplied by epicardial arteries with flow-limiting stenoses, differences in perfusion (i.e., heterogeneous flow distribution) occur between territories, which, in clinical practice, are classified as ischemic and nonischemic. These perfusion differences appear as myocardial perfusion defects, which are suggestive of significant stenoses (typically ≥ 50%) in territories supplied by specific epicardial coronary arteries, providing critical information for patient management and prognosis.^128^ Notably, this technique can also detect myocardial perfusion defects associated with CMD, even in the absence of epicardial disease (ischemia and no obstructive coronary artery disease [INOCA]). These are usually characterized by diffuse subendocardial defects or defects that do not conform to specific coronary artery territories. CMR for ischemia involves comparing images obtained during pharmacological stress and at rest, with reversal of the stress effect using aminophylline. Late gadolinium enhancement (LGE) images are typically analyzed alongside perfusion data, as prior MI areas may present as perfusion defects but do not represent current ischemia. Importantly, extensive myocardial ischemia (usually due to > 90% obstruction) may show perfusion deficits in both phases (stress and rest). Direct visualization of myocardial fibrosis via LGE may reduce the need for rest perfusion imaging to define ischemia. For this reason, some centers adopt a stress-only myocardial perfusion protocol, making the exam faster.^129,130^

CMR-based perfusion imaging has demonstrated high diagnostic accuracy for ischemia, showing superiority over scintigraphy and comparable results to PET.^131^ Its prognostic value is also well established and has been shown to be noninferior to invasive FFR, as demonstrated in recent studies (3.7% vs. 3.6%).^132^

##### 2.2.6.2. Assessment of Segmental Contractility and Contractile Reserve by Cardiovascular Magnetic Resonance Imaging

Pharmacological stress CMR using dobutamine is a method used to assess ischemia by evaluating segmental contractility, as the use of physical exercise in CMR still faces implementation challenges in routine practice. Myocardial ischemia is hence defined as a new contractile deficit or a biphasic response (increased contractility at low doses and dysfunction at high doses). Potential advantages over echocardiography include high image quality and reproducibility, with no limitations related to acoustic windows.^133^ Studies have demonstrated high accuracy of dobutamine stress CMR for detecting coronary obstructions ≥ 50%, with a sensitivity of 81%-84%.^134^ Additionally, dobutamine CMR holds prognostic value, indicating a low event rate when results are normal (< 2% over 2 years).^135^ Limitations include the need for appropriate monitoring during the exam and contraindications to dobutamine.

Table 12 presents the main clinical scenarios for the indication of CMR in the assessment of myocardial ischemia, according to the III Brazilian Guidelines for CMR and CCT.

**Table 12:** Assessment of CAD by cardiovascular magnetic resonance – myocardial ischemia

Indication/Clinical scenario	Recommendation grade	Level of evidence
Assessment of myocardial perfusion under pharmacologic stress with dipyridamole/adenosine	**I**	**A**
Assessment of ventricular contractility under stress with dobutamine	**I**	**B**
Evaluation of stable angina / angina equivalent in patients with intermediate pretest probability of CAD	**I**	**B**
Identification and quantification of myocardial ischemia in patients with known CAD (except high-risk anatomy)	**I**	**B**
Evaluation of patients with known nonobstructive CAD and/or suspected INOCA	**IIa**	**C**

CAD: coronary artery disease; INOCA: ischemia with nonobstructive coronary arteries.

* Defined as > 50% stenosis in the left main coronary artery and three-vessel disease involving proximal segments.

##### 2.2.6.3. Assessment of Coronary Artery Disease by Cardiovascular Magnetic Resonance Imaging – Myocardial Viability

CMR with LGE is widely used to assess myocardial viability. Although this technique provides valuable diagnostic and prognostic information, recent clinical trials have questioned the usefulness of viability assessment for guiding revascularization and reducing adverse cardiac events.

Recently, new CMR data and long-term studies have reinforced the relevance of viability assessment in this context.^136^ However, significant limitations in all published randomized clinical trials still prevent a definitive conclusion about the value and impact of myocardial viability evaluation.^137^ Therefore, expert consensus supports that in specific and carefully selected patients, viability assessment may play a crucial role in revascularization decision-making and long-term prognosis.

Among many clinical scenarios, a classical indication with clear benefit from myocardial viability assessment is the patient with LV dysfunction and akinetic or dyskinetic anterior wall. In such cases, CMR remains an important tool for evaluating myocardial viability and improving patient outcomes.^138–140^

##### 2.2.6.4. Applying Cardiovascular Magnetic Resonance Imaging to Assess Viability in Coronary Artery Disease

An algorithm proposed by the III Guideline on CT and CMR of SBC (Figure 14) for the management of patients with CAD and planning of revascularization is based on the assessment of myocardial viability and ischemia. In patients with chronic dysfunction, contractility and wall thinning are less relevant, as they may reflect either myocardial hibernation or transmural infarction. Intermediate cases (25%-50% transmurality) may benefit from additional evaluation of contractile reserve using dobutamine stress or PET.^123^

**Figure 14:** Assessment of myocardial viability by CMR (chronic setting). CMR: cardiovascular magnetic resonance imaging; LGE: late gadolinium enhancement; PET: positron emission tomography.

##### 2.2.6.5. Recommendation Grade and Level of Evidence

Table 13 summarizes the main recommendations for the use of CMR in the assessment of myocardial viability in the context of CAD. Further details can be found in the 2024 guidelines jointly published by SBC and Brazilian College of Radiology.^123^

**Tabela 13:** Pesquisa de DAC pela ressonância magnética – viabilidade miocárdica

Recommendation	Recommendation grade	Level of evidence
Detecção de infarto agudo e crônico do miocárdio pela técnica de realce tardio	**I**	**A**
Diagnóstico diferencial com etiologia não isquêmica pela técnica de realce tardio (outras CMPs)	**I**	**B**
Avaliação de viabilidade miocárdica no cenário clínico pré-revascularização pela técnica de realce tardio	**I** *	**B** *
Detectar trombo ventricular	**I**	**B**
Avaliação de aneurisma de ventrículo esquerdo	**I**	**B**
Avaliação da função ventricular regional em repouso e em estresse	**IIa**	**B**

DAC: doença arterial coronariana; CMPs: cardiomiopatias.

* Nível de evidência (NE) e grau de recomendação (GR) referentes à escolha do método de ressonância cardíaca como estratégia preferencial para pesquisa de viabilidade miocárdica. A classificação apresentada não representa NE e CR da pesquisa de viabilidade miocárdica (de maneira geral) como estratégia pré-revascularização miocárdica.

#### 2.2.7. Cardiac Catheterization

Cardiac catheterization for the evaluation of patients with CCS allows for characterization of the coronary anatomy, confirmation of the diagnosis, assessment of the severity of CAD, and identification of alternative diagnoses, providing valuable parameters for individualized therapeutic decisions.

Some of these parameters include which vessels are affected, their clinical relevance, the number and location of lesions, involvement of bifurcations, degree of stenosis, lesion length, reference vessel diameter, degree of calcification, presence of thrombus or dissection, quality of the distal bed, patency and degree of obstruction of previously implanted stents or coronary bypass grafts, as well as geometric aspects such as aortic configuration, angulation of coronary origin, and vessel tortuosity. Together, these factors help estimate disease burden, the potential for revascularization (percutaneous and/or surgical), and the risks associated with interventions.^141^

In addition to ICA, cardiac catheterization may include invasive hemodynamic assessment with measurements of pressures in the LV and aorta (e.g., for evaluating ventricular filling pressures) as well as left ventriculography, allowing for assessment of global and segmental LV function – important data to guide therapeutic decision-making.

This diagnostic test is performed in the cardiac catheterization laboratory. It requires local anesthesia at the puncture site, arterial access (radial, or femoral in specific cases), selective catheterization of the coronary arteries, and image acquisition using iodinated contrast agents and X-rays. Although it is an invasive procedure, it allows for highly accurate and safe diagnosis, with very low complication rates – especially when performed via radial access, using iso-osmolar or low-osmolar contrast agents in small volumes and minimizing patient radiation exposure through modern angiography systems.^142^

Indications for cardiac catheterization in patients with CCS should, when possible, be preceded by OMT and include:

- Limiting and/or progressive angina or reduced FC despite OMT;
- New-onset LV dysfunction and/or symptoms of HF;^53,143,166^
- Patients with moderate to severe myocardial ischemia on noninvasive testing may be referred for cardiac catheterization with the intent of revascularization to improve FC and/or anginal symptoms. Alternatively, they may initially be managed with a conservative strategy (OMT alone), provided they do not have significant LV dysfunction (LVEF < 35%), severe left main CAD (LMCAD) (> 50% on CCTA), or limiting anginal symptoms – maintaining the possibility of indicating cardiac catheterization based on the patient's clinical evolution.^92^

Despite being considered the gold standard for diagnosing CAD, ICA has some limitations. One of them is the assessment of the functional significance of intermediate, moderate, or ambiguous coronary lesions, especially in patients with multivessel disease. In such cases – particularly when noninvasive tests are unavailable or yield inconsistent findings – invasive physiological assessment, through FFR measurement or nonhyperemic invasive coronary pressure indices, should be considered to help identify truly flow-limiting lesions. This can enable reclassification of the patient and the simplification or even avoidance of interventions, with safety.^143,144,146^ More recently, flow assessment tools based on angiographic images, which do not require pressure wires or invasive sensors, have been developed and validated, demonstrating clinical benefits in their use.^147^

Another relevant limitation of ICA, as it is a luminogram, lies in the limited information it provides about coronary atherosclerotic plaques. Intravascular imaging tools such as intravascular ultrasound (IVUS) and optical coherence tomography (OCT) can be very useful in the catheterization lab. These modalities allow geometric and morphological characterization of plaques, including determination of minimum luminal area, plaque burden, degree of vascular remodeling, calcium, lipid, and fibrous content, identification of vulnerable plaque phenotypes, and detection of thrombi and plaque disruption (rupture, ulceration, and calcified nodules).

In left main coronary artery lesions or anatomical scenarios where invasive physiological assessment is limited – such as serial coronary lesions or myocardial bridges – IVUS is clinically validated to safely defer interventions, based on minimum luminal area references. However, the main role of intravascular imaging is to guide complex percutaneous coronary interventions (PCIs), leading to improved prognosis and reduced mortality. In patients with CCS and stent failure, intravascular imaging is essential to clarify the underlying mechanism (underexpansion, edge dissection, incomplete plaque coverage, stent undersizing, neointimal proliferation, malapposition, longitudinal shortening, or strut fractures) and to guide therapy.^148–150^

Finally, there are patients with CCS who present with anginal symptoms and ischemia on noninvasive functional testing, but show no significant obstructive lesions (> 50%) on CCTA or cardiac catheterization. This condition, known as INOCA, is related to CVS and CMD, which may coexist with atherosclerotic disease. Its diagnosis has prognostic and therapeutic implications. Detection of CMD requires not only cardiac catheterization but also invasive physiological assessment to rule out functionally significant epicardial obstruction (FFR > 0.80), as well as to confirm reduced CFR (< 2.0), microvascular spasm on acetylcholine testing (angina and ECG changes without epicardial spasm), or elevated index of microvascular resistance (IMR > 25).^151–153^

## 3. Clinical Strategies for Assessing Cardiovascular Risk and Stratifying Coronary Atherosclerotic Disease

Patients with stable CAD present with a heterogeneous risk of MACE, especially death and MI. Therefore, it is essential to perform risk stratification based on clinical data and complementary tests.^80^

The initial assessment should include clinical history, echocardiography to evaluate LVEF, and, when indicated, noninvasive tests to detect ischemia or anatomical assessment of the coronary tree. Patients can be thus classified as under low (< 1% per year), intermediate (1%-3%), or high risk (> 3%) for CV death or nonfatal MI.^80^

### 3.1. Initial Assessment of Risk

Certain clinical findings indicate higher risk: recent-onset or progressive angina, HF likely due to ischemic origin, or a history of ACS. The TRA 2°P score, derived from a secondary prevention cohort,^154^ estimates CV risk based on nine variables: age, diabetes, hypertension, smoking, PAD, prior stroke, coronary artery bypass grafting (CABG), HF, and renal dysfunction.^155^

Socioeconomic factors and access to health care also influence prognosis. Simple tests such as ECG and chest X-ray contribute to the initial stratification. ECG can detect previous infarctions or arrhythmias and is useful even in asymptomatic patients.^156^ The use of artificial intelligence applied to ECG has shown potential in predicting MACE.^157^

Chest X-ray may reveal cardiomegaly, LV aneurysm, or pulmonary congestion, all of which are associated with a higher risk of MACE.

#### 3.1.1. Laboratory Risk Markers in Chronic Coronary Artery Disease

Cardiac troponin, even at slightly elevated concentrations, is a sensitive marker of myocardial injury and is associated with CV risk, even in the absence of MI. High-sensitivity assays have enabled the detection of subclinical troponin levels in various nonischemic conditions, such as renal failure, sepsis, and pulmonary embolism.^41^

The ARIC study demonstrated that elevated troponin levels are associated with a higher incidence of MACE, independently of traditional risk factors.^43^ In patients with stable CAD, persistently elevated high-sensitivity troponin levels correlate with an increased risk of CV death or nonfatal MI.^112^ Concentrations above 10 ng/L identify individuals with a 50% higher risk of events, even after adjusting for CAD severity.

Another important prognostic marker is B-type natriuretic peptide (BNP), which is released in response to volume overload and wall stress. In a cohort of over 13,000 patients with chronic CAD, NT-proBNP was an independent predictor of adverse outcomes, along with troponin and LDL-C.^113^

The ABC-CHD score was then developed, which uses biomarkers (troponin, NT-proBNP, LDL-C) combined with clinical data such as diabetes, smoking, and PAD to estimate individual CV risk.^158^

#### 3.1.2. Exercise Stress Test for Prognostic Assessment in Stable Coronary Artery Disease

EST provides important prognostic information in patients with stable CAD. Indicators of high risk include ST-segment depression at low workload, symptoms during exertion (angina or dyspnea), low exercise capacity, complex ventricular arrhythmias, and abnormal BP response.^166^

On the other hand, patients who complete the third stage of the Bruce protocol have an annual mortality rate of less than 1%. Those who fail to reach 5 METs have an estimated annual mortality of 5%.^159^ Additional high-risk features include an inadequate chronotropic response, systolic BP drop during exercise, ST-segment depression in multiple leads, or persistent depression during recovery (>5 minutes).^160,161^

The DTS^48^ is a validated tool for prognostic stratification based on clinical and exercise test variables. The equation is:


Exercise duration (min)−5×ST depression (mm)−4× angina index (1:no angina;2:nonlimiting angina;3:exercise-limiting angina)

Risk classification:

- Score ≥ +5: low risk (annual mortality ≤1%)
- Score between –10 and +4: intermediate risk (1%-3%)
- Score < –10: high risk (annual mortality >3%)

EST should preferably be used in patients with preserved FC and an interpretable baseline ECG.

#### 3.1.3. Transthoracic Echocardiography (at Rest and Under Stress)

LVEF is one of the most important prognostic markers in stable CAD.^162^ Echocardiography allows for the assessment of ventricular function at rest and the detection of myocardial ischemia during stress testing (either exercise- or drug-induced), and it has strong prognostic value.

The presence of stress-induced ischemia – identified by new hypokinesia or akinesia in previously normal segments – is associated with a higher risk of MACE. The extent of ischemia correlates directly with risk: abnormalities in ≥ 3 segments (out of 16 evaluated) identify patients with an annual risk > 3%.^163^

On the other hand, the absence of ischemia on stress echocardiography indicates a low event risk, comparable to a normal perfusion scintigraphy (< 1% per year).^164^ The technique is particularly useful in patients with a history of MI, enabling assessment of myocardial viability and extent of necrosis.

Stress echocardiography has good diagnostic accuracy and is useful for both functional evaluation and therapeutic decision-making, with prognostic impact validated by large observational studies and international consensus guidelines.^65,165–169^

#### 3.1.4. Myocardial Perfusion Scintigraphy

MPS is one of the most widely used functional imaging methods for assessing stable CAD. It provides prognostic information regarding the extent of ischemia, ventricular function, pulmonary uptake, and stress-induced ventricular remodeling.^170^

The presence of a reversible perfusion defect involving ≥ 10% of LV mass is associated with a high risk of MACE (mortality or infarction > 3% per year).^171^ Conversely, a normal MPS indicates an annual risk <1%, with a high negative predictive value.^164^ MPS also evaluates LVEF and ventricular volume, both of which influence risk. Increased pulmonary uptake or transient LV dilation during stress testing are also markers of higher event risk.^172–174^

Multiple multicenter studies have validated the accuracy of MPS for risk stratification, including in patients with known CAD, suspected ischemia, or prior MI. It is a widely accepted tool due to its robustness, clinical applicability, and well-established prognostic value.^44,175,176^

#### 3.1.5. Cardiovascular Magnetic Resonance Imaging

CMR is a versatile test that simultaneously assesses anatomy, function, perfusion, and myocardial viability. In patients with stable CAD, it provides prognostic information comparable to other functional tests.^177^

A negative stress CMR is associated with an annual event rate of < 1%.^178^ In contrast, the presence of inducible ischemia and/or myocardial fibrosis (LGE) is correlated with increased mortality and risk of MI, regardless of LVEF.^1^

Studies have shown that the number of segments with ischemia or myocardial scarring predicts adverse events, even in patients with apparently preserved ventricular function.^179–181^ The detection of subclinical fibrosis is particularly useful for identifying high-risk patients, even in the absence of symptoms.

A recent meta-analysis involving more than 67,000 patients confirmed that both ischemia and late enhancement on CMR are strong predictors of CV outcomes.^44^ CMR should be considered when a detailed assessment of disease extent and prognostic risk is needed.

#### 3.1.6. Coronary Computed Tomography Angiography

CCTA is an effective tool for risk stratification in patients with stable CAD. It enables the assessment not only of stenosis but also of atherosclerotic plaque burden and composition.^182^

Quantification of total coronary plaque volume – especially low-attenuation plaques and those with positive remodeling – is associated with a higher risk of MACE.^183^ CCTA also allows for the identification of high-risk plaques, such as those with necrotic cores or the napkin-ring sign.^184^

Studies have shown that CCTA is superior to isolated anatomical assessment, as it combines morphological and functional findings. The presence of high-risk plaques or significant proximal stenosis is linked to an elevated MACE risk over a 10-year period.^185^

Combining CCTA findings with integrated scoring algorithms, such as using a comprehensive atherosclerotic risk score, enhances outcome prediction and helps guide more intensive preventive strategies.^44^

Table 14 lists selected high-risk definitions for MACE based on diagnostic modality in patients with CCS.

**Table 14:** High-risk definitions for cardiovascular events by diagnostic modality in patients with chronic coronary syndrome

Diagnostic modality	High-risk definition
Exercise stress test	Duke Score < –10, corresponding to annual cardiovascular mortality >3%
Myocardial perfusion scintigraphy	Reversible ischemia ≥10% of left ventricular mass
Stress echocardiography	Stress-induced wall motion abnormalities in ≥3 out of 16 left ventricular segments
CMR	Ischemia in ≥2 segments or ≥3 dysfunctional segments with dobutamine stress
CCTA/ICA	Left main coronary artery stenosis, 3-vessel CAD with proximal lesions, or proximal LAD involvement

CCTA: coronary computed tomography angiography; CMR: cardiovascular magnetic resonance; ICA: invasive coronary angiography.

#### 3.1.7. Summary and Recommendation for Diagnostic Investigation

Estimating the PTP of obstructive CAD is essential for selecting the appropriate initial diagnostic strategy. This estimate should consider patient age, sex, and chest pain characteristics (typical angina, atypical angina, or noncardiac pain) as well as relevant risk factors and comorbidities.^18^

The 2019 ESC guideline revised PTP values based on large contemporary registries, correcting the overestimated probabilities found in earlier models, such as the Diamond–Forrester model.^1^ Patients with a PTP < 5% generally do not require further testing. Those with a PTP between 5%-15% should be evaluated on an individual basis. A PTP of 15%-85% indicates the need for either functional or anatomical testing, while a PTP > 85% usually supports a clinical diagnosis of CAD.

We recommend using the PTP estimation model with which the clinician is most familiar, taking into consideration the limitations of each model and giving appropriate weight to clinical judgment – although inherently dependent on accumulated experience – since no single model has been clearly established as ideal for assessing the Brazilian population. A comparative analysis of PTP models for obstructive CAD is presented in Chart 7.^1,3,18,19,186^

**Chart 7:** Comparison of PTP models for obstructive CAD

Model	Year/Origin	Variables considered	Characteristics	Strengths	Limitations
Diamond–Forrester (classic)	1979/USA	Age, sex, chest pain type (typical, atypical, or nonanginal)	Original model for estimating PTP of CAD in symptomatic patients	Simple, fast, historically influential	Overestimates PTP in contemporary populations; based on 1970s data
Modified Diamond–Forrester (PROMISE trial)	2018/USA–Europe	Age, sex, chest pain type	Recalibrated using PROMISE study data, more representative of current populations	Improves accuracy of the classic model in modern populations	Limited to three variables; does not include other risk factors
CAD Consortium Basic Model	2012/Europe	Age, sex, chest pain type	Derived from a large European cohort; used in 2013 ESC Guidelines	Reproducible; basis for previous guidelines	Still tends to overestimate risk in patients with atypical symptoms
CAD Consortium Clinical Model (Genders et al.)	2012/Europe	Complete clinical model; included in 2019 ESC Guidelines	Complete clinical model; included in 2019 ESC Guidelines	More accurate; includes additional risk factors	Requires more clinical data; less used in settings with limited clinical access
ESC 2019 Recalibrated	2019/ESC Guidelines	Age, sex, chest pain type, includes dyspnea as a symptom	Recalibrated using data from large contemporary European registries	Reflects current reality; generally lower PTP; includes "isolated dyspnea"	May underestimate risk in patients with relevant risk factors not included
Clinical judgment	–	Subjective assessment based on clinical experience and context	Complements mathematical models when uncertainty or unmodeled scenarios exist	Important in heterogeneous health systems such as Brazil	Highly variable; dependent on individual experience

CAD: coronary artery disease; ESC: European Society of Cardiology; PTP: pretest probability.

The choice of initial diagnostic testing should consider:

- **FC:** If preserved, begin with an EST or stress echocardiography;
- **Interpretability of baseline ECG:** If uninterpretable, prioritize imaging modalities (echocardiography, MPS, or CMR);
- **Local availability and access:** Consider cost, test duration, and the team's familiarity with the method.
- **CKD or contrast allergy:** These conditions may limit the use of CCTA.

Figure 15 illustrates the decision-making process for symptomatic patients with suspected CCS. Figure 16 outlines the recommended diagnostic tests according to the PTP of obstructive CAD.

**Figure 15:** Decision-making in symptomatic patients with suspected chronic coronary syndrome. ECG: electrocardiogram.

**Figure 16:** Recommended diagnostic tests based on PTP of obstructive CAD. CAD: coronary artery disease; CCTA: coronary computed tomography angiography; ICA: invasive coronary angiography; PTP: pretest probability.

## 4. Clinical Pharmacological Treatment for Reducing the Risk of Major Adverse Cardiovascular Events

### 4.1. Antithrombotic Therapy in Chronic Coronary Syndrome

#### 4.1.1. Aspirin

Acetylsalicylic acid (ASA) is an irreversible inhibitor of the cyclooxygenase (COX) enzyme and thromboxane A2 (TXA2) synthesis. Its greater selectivity for COX-1 over COX-2 contributes to its predominant antithrombotic effect.^187^ ASA is administered at a daily dose of 75-100 mg and has demonstrated benefit in patients with CAD, with a significant reduction in MACE – approximately 21% relative risk reduction (RRR) in patients with prior MI and 37% in other groups.^188,189^

#### 4.1.2. P2Y12 Inhibitors

This class includes clopidogrel and prasugrel – thienopyridines that irreversibly block the P2Y12 platelet receptor – as well as ticagrelor, a reversible inhibitor that does not require metabolic activation. The CAPRIE (Clopidogrel Versus Aspirin in Patients at Risk of Ischemic Events) trial demonstrated a modest benefit of clopidogrel over ASA, with reduced rates of MI, stroke, and CV death (5.32% vs. 5.83%, RRR 8.7% [0.3-16.5], p = 0.043). Subgroup analysis revealed that this benefit was significant only among patients with PAD.^190^ The active metabolite of clopidogrel is generated via cytochrome P2C19. Therefore, patients with a loss-of-function allele of the gene that encodes this enzyme may exhibit reduced drug response.^191^ Moreover, drugs that inhibit this enzyme (e.g., omeprazole) can decrease clopidogrel efficacy.^192^

Prasugrel and ticagrelor offer faster and more predictable antiplatelet effects as they do not depend on P2C19 metabolism.^193^ Prasugrel was shown to be superior to clopidogrel in patients with ACS undergoing PCI, but with an increased risk of bleeding in those with prior stroke and no benefit in patients aged > 75 years or weighing < 60 kg.^194,195^ Ticagrelor, in addition to its proven benefit in ACS, has demonstrated a reduction in MACE in patients with CAD on ASA and a history of MI within the previous 3 years, although at the cost of higher rates of nonfatal bleeding.^195^ A common adverse effect of ticagrelor is dyspnea, which is typically mild and self-limiting.^197^

#### 4.1.3. Dual Antiplatelet Therapy

Dual antiplatelet therapy (DAPT) with ASA and clopidogrel has been shown to reduce MACE rates in patients with ACS for up to 1 year when compared with ASA monotherapy.^197^ In the acute setting, DAPT with ASA plus ticagrelor (for all ACS presentations) or ASA plus prasugrel (limited to ACS treated with PCI) was superior to ASA plus clopidogrel in reducing MACE.^197^ More recently, ISAR-REACT 5 (Intracoronary Stenting and Antithrombotic Regimen: Rapid Early Action for Coronary Treatment-5) – an open-label, randomized clinical trial – suggested a potential superiority of prasugrel over ticagrelor in patients with ACS undergoing PCI.^198^

Regarding CCS, the CHARISMA (Clopidogrel for High Atherothrombotic Risk, Ischemic Stabilization, Management, and Avoidance) trial randomized a total of 19,185 patients on ASA to receive either clopidogrel or placebo. Although the group receiving clopidogrel showed a lower MACE rate, the difference was not statistically significant (HR 0.93, 95% CI 0.83-1.05, p = 0.22).^199^ A subsequent subgroup analysis however demonstrated benefit only among patients with prior MI, stroke, or PAD (HR 0.77, 95% CI 0.61-0.98, p = 0.031).^200^ The DAPT study compared thienopyridines (65% clopidogrel, 35% prasugrel) plus ASA for 30 months versus 12 months after PCI, showing a significant reduction in MACE, but only in patients with prior MI (HR 0.56, 95% CI 0.42-0.76, p < 0.001).^201^

However, the strongest body of evidence in this setting supports the use of ticagrelor in combination with ASA. The PEGASUS-TIMI 54 (Prevention of Cardiovascular Events in Patients with Prior Heart Attack Using Ticagrelor Compared to Placebo on a Background of Aspirin–Thrombolysis in Myocardial Infarction 54) trial demonstrated that DAPT with these agents, compared to ASA plus placebo, significantly reduced MACE in patients with prior MI and at least one additional risk factor – such as age > 65 years, diabetes, CKD, or multivessel disease (HR 0.84, 95% CI 0.74-0.95, p = 0.008).^202^ The THEMIS (Effect of ticagrelor on Health Outcomes in diabEtes Mellitus patients Intervention Study) trial assessed DAPT with ASA plus ticagrelor in patients with diabetes and no prior MI, demonstrating a modest reduction in MACE after a mean follow-up of 40 months (HR 0.90, 95% CI 0.81-0.99, p = 0.04), at the cost of a significant increase in major bleeding (HR 2.32, 95% CI 1.82-2.94, p < 0.001).^203^

#### 4.1.4. Low-Dose Anticoagulation

The COMPASS (Cardiovascular Outcomes for People Using Anticoagulation Strategies) trial evaluated 27,395 patients with CAD and high CV risk, who were randomized into three groups: (1) ASA 100 mg; (2) rivaroxaban 5 mg; or (3) ASA 100 mg plus rivaroxaban 2.5 mg. Eligible participants had either established atherosclerosis in at least two vascular territories or at least two risk factors (e.g., active smoking, diabetes, eGFR < 60 mL/min, HF, or recent ischemic stroke). Compared with ASA monotherapy, the combination of ASA plus rivaroxaban 2.5 mg significantly reduced MACE over a median follow-up of 23 months (HR 0.76, 95% CI 0.66-0.86, p < 0.001).^204^

#### 4.1.5. Long-Term Anticoagulation

Numerous studies have demonstrated the benefit of long-term anticoagulation in patients with atrial fibrillation (AF) for the prevention of embolic events. For example, a meta-analysis involving 28,044 patients showed that oral anticoagulants (OAC) reduces the relative risk of ischemic stroke by approximately 62%.^205^ However, in the subgroup of patients with both AF and CAD who are also on chronic antiplatelet therapy, the optimal management strategy remains unclear.

More recently, the AFIRE (Atrial Fibrillation and Ischemic Events With Rivaroxaban in Patients With Stable Coronary Artery Disease) trial evaluated 2,236 patients with AF and stable CAD (35% with prior MI), randomized to receive rivaroxaban alone or in combination with ASA or clopidogrel. After a median follow-up of 24 months, rivaroxaban monotherapy was found to be noninferior for the prevention of stroke, systemic embolism, MI, or unstable angina (HR 0.72, 95% CI 0.55-0.95). However, the proportion of patients with prior MI was significantly higher in the monotherapy group.^206^

#### 4.1.6. Dual antiplatelet Therapy in Percutaneous Coronary Intervention

DAPT reduces the risk of stent thrombosis in patients undergoing elective PCI and should be maintained for 6 months following the procedure, rather than 12 months as in the case of ACS. The largest study in this area is the ISAR-SAFE (Intracoronary Stenting and Antithrombotic Regimen: Safety And EFficacy of 6 Months Dual Antiplatelet Therapy After Drug-Eluting Stenting) trial, which evaluated 4,005 patients with CAD undergoing PCI (50% electively). ISAR-SAFE demonstrated that patients who received DAPT for 6 months had a noninferior rate of death, MI, stent thrombosis, stroke, or bleeding compared with those who continued therapy for 12 months (1.5%, 95% CI 0.9-2.0 vs. 1.6%, 95% CI 1.1-2.2; p < 0.001).^207^

As for other antiplatelet agents such as prasugrel and ticagrelor, there is currently no evidence that they are superior to clopidogrel in the setting of elective PCI. The TWILIGHT (Ticagrelor with Aspirin or Alone in High-Risk Patients after Coronary Intervention) trial evaluated patients on DAPT for 3 months following elective PCI and found that those who continued on ticagrelor monotherapy through 12 months experienced less bleeding than those who remained on DAPT (HR 0.56, 95% CI 0.45-0.68; p < 0.001).^208^

#### 4.1.7. Anticoagulants in Percutaneous Coronary Intervention

The WOEST (What is the Optimal antiplatElet and anticoagulant therapy in patients with oral anticoagulation and coronary StenTing) trial was the first to demonstrate that combining clopidogrel with an OAC in patients undergoing elective PCI significantly reduced bleeding rates compared with triple therapy (ASA + clopidogrel + anticoagulant).^209^ In the following years, several studies confirmed this finding. The largest among them – the AUGUSTUS (Aspirin Placebo in Patients with Atrial Fibrillation and Acute Coronary Syndrome or Percutaneous Coronary Intervention) trial – randomized 4,614 patients with AF undergoing either urgent or elective PCI. The study showed a lower incidence of bleeding events when apixaban was combined with clopidogrel, compared with triple therapy using ASA, clopidogrel, and warfarin (HR 1.89, 95% CI 1.59-2.24; p < 0.001) (Table 15).^210^

**Table 15:** Recommendations for the Use of Anticoagulants in the Context of Percutaneous Coronary Intervention

Recommendation	Recommendation grade	Level of evidence
ASA 100 mg/day is recommended for patients with a previous myocardial infarction or for those who have undergone myocardial revascularization, with or without a prior infarction	**I**	**A**
Clopidogrel 75 mg/day is recommended as an alternative to ASA in patients with intolerance. It may also be considered as an alternative with at least similar efficacy for patients with CAD, PAD, or prior ischemic stroke	**I**	**B**
Clopidogrel may be considered an alternative to ASA with at least similar efficacy for CAD, PAD, or prior ischemic stroke.	**IIa**	**B**
ASA 100 mg/day may be considered in patients with CAD without prior MI or revascularization	**IIb**	**C**
Long-term DAPT may be considered in patients at high CV risk and low bleeding risk.	**IIa**	**A**
ASA 100 mg/day is recommended for patients undergoing PCI	**I**	**A**
Clopidogrel 75 mg/day is recommended for patients undergoing PCI for at least 6 months after the procedure	**I**	**A**
Clopidogrel 75 mg/day may be considered for patients undergoing PCI for at least 3 months if bleeding risk is high	**IIa**	**A**
In patients with an indication for anticoagulation after PCI, DOACs are preferred in combination with antiplatelet therapy	**I**	**A**
In patients with an indication for anticoagulation after PCI, ASA may be discontinued after 1 week, maintaining only the anticoagulant and clopidogrel, if thrombotic risk is low	**IIa**	**B**
In patients with an indication for anticoagulation after PCI, triple therapy with ASA + clopidogrel + anticoagulant may be maintained for at least 1 month if thrombotic risk is very high	**IIa**	**C**
In patients with an indication for anticoagulation after PCI, dual therapy with anticoagulant + prasugrel or ticagrelor may be considered as an alternative to triple therapy if bleeding risk is high	**IIb**	**C**
Prasugrel and ticagrelor are not recommended as part of triple therapy with ASA and anticoagulant	**III**	**C**

ASA: acetylsalicylic acid; CAD: coronary artery disease; DAPT: dual antiplatelet therapy; DOAC: direct oral anticoagulant; PCI: percutaneous coronary intervention.

### 4.2. Lipid Management in Chronic Coronary Syndrome

In patients with established CAD, classified as being at very high MACE risk, lipid abnormalities should be treated with pharmacological therapy in combination with lifestyle interventions. Exercise, dietary modification, and weight control should be recommended for all patients.

#### 4.2.1. Statins and Other Low-Density Lipoprotein Cholesterol-Lowering Agents

The causal relationship between LDL-C and CVD risk has been well established through epidemiological studies, Mendelian randomization analyses, and randomized clinical trials.^110^ Accordingly, LDL-C is the primary therapeutic target, and new treatment strategies have emerged in recent years.

Recent studies evaluating the combination of statins and PCSK9 inhibitors (PCSK9i) have demonstrated that progressively lower LDL-C levels are associated with continued improvement in atherosclerotic plaque characteristics^211^ and a reduction in MACE.^212^ Therefore, this guideline recommends a goal-directed LDL-C-lowering strategy to reduce atherosclerotic risk.

In individuals with CCS, an LDL-C target of less than 50 mg/dL should be pursued along with a reduction of more than 50% from baseline levels.^213^ The recommendation for an additional percentage reduction is based on the observation that even patients with LDL-C levels below 100 mg/dL at the time of their first coronary event derive substantial benefit from further lipid-lowering. A lower therapeutic target – less than 40 mg/dL – may be considered in patients who experience a second MACE within 2 years of the first.^214^

Due to the substantial body of evidence indicating a 20% to 25% decrease in the risk of recurrent MACE with a 39 mg/dL reduction in LDL-C, high-intensity statins are recommended as the primary treatment. Numerous studies and meta-analyses have shown greater clinical benefit with more potent statins compared to less potent ones; similarly, higher doses of the same statin have been more effective than lower doses.^215^ Although high-intensity statins can reduce LDL-C by approximately 50%, achieving target levels often requires combination therapy.

The addition of ezetimibe to statins has been shown to further reduce LDL-C levels by 18% to 25%. The IMPROVE-IT (Improved Reduction of Outcomes: Vytorin Efficacy International Trial) demonstrated a reduction in MACE among individuals post-ACS who received simvastatin plus ezetimibe compared to statin monotherapy.^216^ Despite its proven efficacy and safety, ezetimibe remains underutilized in patients at very high CV risk.

Since 2015, clinical trials have confirmed the safety and efficacy of monoclonal antibodies targeting PCSK9. By promoting extrahepatic degradation of PCSK9, these agents enhance the recycling of LDL-C receptors to the hepatic cell surface. PCSK9i (evolocumab and alirocumab) reduce LDL-C levels by approximately 60%, even in patients already on statins. The FOURIER (Further Cardiovascular Outcomes Research With PCSK9 Inhibition in Subjects With Elevated Risk) trial evaluated patients with established CVD and, after a median follow-up of only 2.2 years, showed that those treated with statins plus evolocumab reached a median LDL-C of 30 mg/dL and experienced a 15% reduction in the composite CV outcome compared to those on statins alone.^212^ Importantly, no increased risk of adverse events was observed among patients who achieved very low LDL-C levels during the study.

More recently, the FOURIER-OLE (FOURIER Open-Label Extension) study was conducted to assess the long-term safety of sustained LDL-C reduction using a combination of statins and evolocumab. In this study, 6,559 patients previously enrolled in FOURIER received evolocumab for an average of 5 years – half had already been treated with statins plus evolocumab for 2.2 years, while the other half had received only statin therapy. No increase in adverse events was observed during annual follow-ups over the 5-year period.^217^ In a subsequent analysis from the same trial, recently published, the incidence of adverse events remained unchanged even among those who achieved LDL-C levels below 20 mg/dL.^218^ An exploratory analysis revealed that patients who had already received evolocumab in FOURIER and continued this combination in FOURIER-OLE experienced a reduction in CV mortality compared to those who initiated evolocumab only in the latter study. This finding was attributed to a "legacy effect" associated with earlier initiation of intensive lipid-lowering therapy.

Despite the clinical benefit and favorable safety profile demonstrated by PCSK9i when combined with statins and ezetimibe, their widespread use remains limited by high cost.

An alternative therapeutic approach targeting PCSK9 and resulting in LDL-C reduction is RNA interference.^219^ Inclisiran is a small interfering RNA (siRNA) designed to bind and degrade PCSK9 mRNA, thereby inhibiting hepatic PCSK9 synthesis at the intracellular level. Unlike monoclonal antibodies that block circulating PCSK9, inclisiran suppresses its production. One key advantage of this therapy is its long duration of action, with the current formulation allowing for subcutaneous administration every 3 to 6 months.

This medication has been commercially available since 2020 in the European Union and since 2021 in the United States. Recently, three phase 3 clinical trials evaluating inclisiran were published. In all studies, approximately 90% of participants were on statin therapy. Inclisiran 300 mg or placebo was administered at baseline, at 3 months, and then every 6 months thereafter. The ORION-9 trial included 482 patients with heterozygous familial hypercholesterolemia. The mean baseline LDL-C level was 153 mg/dL despite statin use. On day 510, inclisiran reduced LDL-C levels by 47.9%, regardless of the underlying genotype. The ORION-10 and ORION-11 trials had similar designs and included patients with established CVD and/or high CV risk (n = 1,561 and n = 1,617, respectively). On day 510, LDL-C reductions with inclisiran were 52.3% and 49.9%, respectively.^220^ The incidence of a prespecified CV outcome was numerically lower among patients treated with inclisiran.

Across the ORION-9, -10, and -11 trials, reductions were also observed in total cholesterol, non-HDL cholesterol, apoB, TGs, and Lp(a), while HDL-C levels increased. Adverse events were comparable between treatment groups in all three studies, with the exception of injection site reactions – typically mild and transient – which were more frequent in the inclisiran group.

To assess CV outcomes, the ORION-4 trial is currently underway, enrolling approximately 15,000 patients with established CVD, all receiving statin therapy and with baseline LDL-C levels above 79 mg/dL. Inclisiran (or placebo) is administered at baseline, at 3 months, and every 6 months thereafter for a total of 5 years. ORION-4 is expected to be finished in 2026.

Bempedoic acid is a lipid-lowering agent approved for use in the European Union and the United States since 2020. Bempedoic acid acts by inhibiting hepatic cholesterol synthesis at a step upstream of the site of statin action. It is considered a prodrug, as it requires activation by the enzyme acyl-CoA synthetase 1, which is present in the liver but absent in muscle tissue. This selective activation is a key advantage because it minimizes the risk of myopathy.

In clinical trials evaluating its efficacy and safety, bempedoic acid showed modest LDL-C reduction when used in combination with statins. However, when administered as monotherapy in patients intolerant to statins, it achieved an LDL-C reduction of just over 20%. Its fixed-dose combination with ezetimibe, already available outside Brazil, provides an LDL-C reduction comparable to that of moderate- to high-intensity statin therapy. The recently published CLEAR (Cholesterol Lowering via Bempedoic Acid, an ACL-Inhibiting Regimen) Outcomes trial evaluated bempedoic acid in 13,970 patients with baseline LDL-C levels above 100 mg/dL, of whom 30% were at high-risk primary prevention and 70% were in secondary prevention.^221^ Approximately 22% of participants were on very low doses of statins. The mean LDL-C at baseline in both groups was 139 mg/dL. Eligible participants had to be intolerant to at least two statins or to one statin with unwillingness to try another. A total of 6 months after the start of treatment, a mean LDL-C reduction of 21% was observed. Throughout the study, the mean difference in LDL-C reduction between bempedoic acid and placebo was 22 mg/dL. Regarding the primary composite endpoint (CV death, nonfatal MI, nonfatal stroke, or coronary revascularization), the event rate was 13.3% in the placebo group and 11.7% in the bempedoic acid group, representing a RRR of 13%, an absolute risk reduction (ARR) of 1.6% (95% CI 0.79-0.96). In terms of adverse events, a slight increase in hepatic enzyme levels was observed, along with a higher incidence of gout and cholelithiasis in the bempedoic acid group.

#### 4.2.2. Lipoprotein(a)

Epidemiological studies have shown that plasma concentrations of Lp(a) are linearly correlated with an increased risk of atherosclerosis and MACE. Similarly, genetic studies have consistently suggested that lifelong exposure to elevated Lp(a) levels is strongly and causally associated with a higher risk of MACE as well as calcific aortic valve stenosis.^222^

Nonpharmacological interventions, such as diet and physical activity, do not lower Lp(a) levels. Mendelian randomization studies have suggested that a reduction of approximately 60 to 100 mg/dL (150 to 250 nmol/L) would be necessary to achieve a 20% to 30% reduction in CV risk. Drugs that reduce Lp(a) levels by 20-30% – including niacin and PCSK9i – have not demonstrated unequivocal clinical benefit. As such, no therapies are currently approved specifically for Lp(a) reduction.

Phase 3 trials are currently underway in secondary prevention populations using drugs that target messenger RNA to inhibit the synthesis of apolipoprotein(a). Pelacarsen, a subcutaneously administered antisense oligonucleotide, has been shown to lower Lp(a) levels by approximately 80%. The Lp(a)HORIZON (Assessing the Impact of Lipoprotein (a) Lowering With Pelacarsen [TQJ230] on Major Cardiovascular Events in Patients With CVD) trial, initiated in 2019, has already randomized over 8,300 patients with established CVD and Lp(a) levels >70 mg/dL and will continue follow-up until 2025. The OCEAN(a)-DOSE (Olpasiran Trials of Cardiovascular Events and Lipoprotein[a] Reduction–Dose Finding Study) trial randomized approximately 7,297 patients with prior CAD and Lp(a) levels >200 nmol/L to receive olpasiran (a small double-stranded interfering RNA) or placebo through the end of 2026; this agent can reduce serum Lp(a) levels by up to 90%. The results of these studies will be critical to definitively establishing whether there is a causal relationship between Lp(a) and CVD.^223^

#### 4.2.3. Triglyceride-Rich Lipoproteins

A new perspective in epidemiology suggests that these lipoproteins, characterized by high TG content, are strong and independent predictors of atherosclerotic MACE and all-cause mortality. Their cholesterol content – referred to as remnant cholesterol – is considered the main driver of MACE incidence.^224^ In contrast to what has been observed with HDL, Mendelian randomization studies suggest that TG-rich lipoproteins are causally associated with atherosclerotic MACE. Additionally, genetic evidence has shown that elevated concentrations of TG-rich lipoproteins are also causally associated with inflammation, which is another key factor in the development of atherosclerosis.

Nonpharmacological interventions (e.g., dietary modifications, alcohol restriction, and physical activity) play a central role in reducing serum TG levels. When pharmacologic therapy is indicated, statins remain the first-line treatment, with the goal of reducing non-HDL cholesterol or apoB.^225^ Once these targets are achieved, additional therapies may be considered in combination with statins to address residual CV risk.

Analyses of studies involving fibrates – drugs that lower serum TG levels – have suggested, through secondary and post hoc analyses, that patients with both elevated TG levels and low HDL-C may benefit from fibrate therapy, despite neutral results in the primary outcomes. However, a recently published trial evaluating pemafibrate versus placebo in patients with diabetes and atherogenic dyslipidemia already receiving statin therapy showed that TG reduction without a concomitant decrease in circulating ApoB-containing particles did not result in reduced MACE risk.^266^ Therefore, the use of fibrates specifically for CV risk reduction is no longer recommended.

An alternative therapeutic strategy for lowering TG levels and reducing MACE involves long-chain polyunsaturated fatty acids, either as a combination of eicosapentaenoic acid (EPA) and docosahexaenoic acid (DHA), or EPA alone. The REDUCE-IT (Reduction of Cardiovascular Events with Icosapent Ethyl-Intervention Trial) trial, conducted in patients treated with statins who had elevated TG levels and either established CVD or diabetes with additional risk factors, assessed whether highly purified ethyl EPA could lower the risk of MACE compared with placebo. Treatment with EPA was associated with a significant reduction in the composite outcome of CV death, MI, or stroke, with event rates of 20% in the placebo group and 16.2% in the EPA group (RRR of 26%; ARR of 3.6%; NNT = 28).^225–227^

In contrast, the STRENGTH (Long-Term Outcomes Study to Assess Statin Residual Risk with Epanova in High Cardiovascular Risk Patients with Hypertriglyceridemia) trial, which evaluated a combination of EPA and DHA versus placebo in a population similar to that of REDUCE-IT, showed no clinical benefit of omega-3 therapy.^228^ A possible explanation for the discrepant findings lies in the choice of placebo: REDUCE-IT used mineral oil, which has been associated with increased LDL-C levels and inflammatory markers, whereas STRENGTH used corn oil, which had a neutral effect. To clarify the true benefit of highly purified EPA, an ideal approach would involve replicating the study using EPA monotherapy with corn oil as the placebo (Table 16).

**Table 16:** Recommendations for Triglyceride-Rich Lipoproteins

Recommendations for achieving LDL-C targets	Recommendation grade	Level of evidence
In patients with CCS, a 50% reduction in LDL-C from baseline, combined with a target below 50 mg/dL, is recommended	**I**	**A**
For patients with CCS who have a second vascular event within 2 years of the first and are on maximum tolerated statin therapy, a target below 40 mg/dL may be considered	**IIb**	**B**

Recommendations for pharmacological reduction of LDL-C in patients with CCS	Recommendation grade	Level of evidence
It is recommended to prescribe high-intensity statin therapy up to the maximum tolerated dose to achieve LDL-C targets	**I**	**A**
If the target is not achieved with the maximum tolerated dose of statin, combination with ezetimibe is recommended	**I**	**B**
Patients CCS on the maximum tolerated dose of statin plus ezetimibe who do not reach LDL-C targets should be treated with a PCSK9i	**I**	**A**

Recommendations for pharmacological therapy in patients with CCS and hypertriglyceridemia	Recommendation grade	Level of evidence
Statin therapy is recommended as the first-line option to reduce MACE risk in individuals with CCS and TGs above 200 mg/dL	**I**	**A**
In patients with CCS with TGs above 135 mg/dL on statin therapy and lifestyle modifications, omega-3 polyunsaturated fatty acid (icosapent ethyl) at 4 grams per day may be considered in combination with statins	**IIb**	**B**
In patients with CCS on statin therapy with persistent TGs above 200 mg/dL, fibrates should not be considered for MACE risk reduction	**III**	**A**

LDL-C: low-density lipoprotein cholesterol; CCS: chronic coronary syndrome; MACE: major adverse cardiovascular events; TG: triglyceride.

### 4.3. Hormone Replacement Therapy

The results of large randomized trials have shown that hormone replacement therapy does not provide prognostic benefit and increases the risk of CVD in women over 60 years of age.^229^

Hormone replacement therapy in postmenopausal women does not reduce the risk of CAD and is therefore not recommended for either primary or secondary prevention (Table 17).^229–231^

**Table 17:** Recommendations for Hormone Replacement Therapy

Recommendation	Recommendation grade	Level of evidence
Hormone replacement therapy is not recommended for risk reduction in postmenopausal women	**III**	**C**

### 4.4. Renin-Angiotensin-Aldosterone System Blockade

Angiotensin-converting enzyme inhibitors (ACEi) have demonstrated benefits in reducing mortality, MI, stroke, and HF in patients with reduced LVEF,^232–234^ preexisting vascular disease and high CV risk^235,236^ and diabetes.^237^ However, in patients with stable coronary disease and preserved LV function, not all studies have shown that ACEi provide additional benefit in terms of all-cause mortality, CV mortality, nonfatal MI, stroke, or HF.^238^ A meta-analysis of 24 studies demonstrated that in patients with CCS without HF, RAAS blockade reduced MACE and mortality only when compared with placebo, but not when compared with active controls.^236^ Even so, the benefit of this drug class was observed primarily in studies with higher event rates.

Therefore, ACEi therapy in patients with CCS without HF or without high CV risk is generally not recommended. Its use is advised when necessary to achieve BP targets.

ACEi, or angiotensin receptor blockers (ARBs) as an alternative for patients who do not tolerate ACEi, should be considered for the treatment of patients with CCS and coexisting hypertension, reduced LVEF (< 40%), diabetes, or CKD, unless contraindicated (e.g., severe renal failure or hyperkalemia).

#### 4.4.1. Neprilysin Inhibitors and Angiotensin Receptor Blockers

More recently, the pharmacological inhibition of neprilysin – a membrane-bound neutral endopeptidase that degrades a variety of bioactive peptides – has been shown to increase levels of bradykinin and natriuretic peptides. This leads to enhanced diuresis and natriuresis, improved myocardial relaxation and ventricular remodeling, and reduced renin and aldosterone secretion. The first agent in this class is LCZ696, a combination of valsartan and sacubitril (a neprilysin inhibitor). In patients with HF and reduced LVEF (≤35%) who remained symptomatic despite OMT, sacubitril/valsartan was superior to enalapril in reducing the risk of mortality and hospitalization due to HF.^240^ Notably, 60% of patients in this study had IHD. Therefore, sacubitril/valsartan is recommended as a replacement for ACEi to further reduce the risk of HF-related hospitalization and death.

#### 4.4.2. Aldosterone Antagonists

Aldosterone blockade with spironolactone or eplerenone is recommended for use in post-MI patients receiving OMT, with reduced LVEF (≤35%), and who have diabetes or HF.^241,242^ These agents should be used with caution in patients with impaired renal function or hyperkalemia (Table 18).

**Table 18:** Recommendations for the use of renin–angiotensin–aldosterone system inhibitors. Priority should be given to angiotensin-converting enzyme inhibitors (ACEIs); in patients intolerant to ACEIs, angiotensin receptor blockers (ARBs) should be used

Recommendation	Recommendation grade	Level of evidence
Routinely indicated in the presence of ventricular dysfunction and/or HF and/or diabetes.	**I**	**A**
Routinely indicated in all patients with CAD.	**IIa**	**B**
The use of aldosterone blockers is recommended in post-AMI patients on OMT, with reduced LVEF (≤35%) and with diabetes or HF.	**IIa**	**A**

AMI: acute myocardial infarction; CAD: coronary artery disease; HF: heart failure; LVEF: left ventricular ejection fraction; OMT: optimal medical therapy.

### 4.5. Colchicine

A strong causal relationship has been established between inflammation and atherosclerotic disease, indicating that controlling systemic inflammatory activity is a promising therapeutic target. Clinical trials have tested both broad-spectrum and targeted anti-inflammatory therapies for the prevention of atherosclerotic MACE. Among these strategies, key therapeutic targets have included inhibition or blockade of interleukin (IL)-1β by monoclonal antibodies, suppression of the canonical NLRP3 inflammasome pathway, and IL-6 inhibition – acting directly on the innate immune response.

Among the generic therapies evaluated, methotrexate and colchicine were investigated. In the Cardiovascular Inflammation Reduction Trial (CIRT), low-dose methotrexate (15 to 20 mg/week) was compared with placebo but was discontinued early due to futility. Additionally, patients receiving methotrexate experienced elevated liver enzyme levels, decreased leukocyte and hematocrit counts, and a higher incidence of skin cancer compared with placebo.^243^

Colchicine, used for decades, exerts its anti-inflammatory effects by binding to tubulin and inhibiting its polymerization. This mechanism disrupts cytoskeletal function, reducing chemotaxis, cellular migration and signaling, and mitosis. Other mechanisms include downregulation of adhesion molecule expression, suppression of the NF-κB pathway, and inhibition of NLRP3 inflammasome formation.^244^ In the LoDoCo2 (Low-Dose Colchicine 2) trial, 5,522 patients with chronic CAD were randomized to receive colchicine 0.5 mg/day or placebo. Colchicine treatment reduced the primary composite outcome of CV death, nonprocedural MI, ischemia-driven coronary revascularization, or ischemic stroke by 1.1% compared to placebo (HR, 0.69; 95% CI, 0.57-0.83; p < 0.001). However, the incidence of nonCV death was 0.2% higher in the colchicine group (HR, 1.51; 95% CI, 0.99-2.31).^245^

Recent meta-analyses including patients in secondary prevention settings have assessed the efficacy and safety of colchicine for preventing atherosclerotic events and ischemic stroke, demonstrating benefit from low-dose colchicine (0.5 mg/day) in reducing such events. However, there was heterogeneity across the studies, including in patient inclusion criteria.^246,247^

To evaluate the potential of colchicine as an anti-inflammatory therapy in the acute post-MI phase, the multicenter CLEAR SYNERGY (OASIS 9) trial enrolled 7,064 patients and randomized them (1:1) to receive colchicine or placebo. The trial assessed a composite primary efficacy outcome including CV death, recurrent MI, ischemic stroke, or unplanned ischemia-driven coronary revascularization, using a time-to-event analysis. After a median follow-up of 3 years, no significant benefit was observed in this specific patient population.^248,249^

Colchicine has a narrow therapeutic index, meaning there is a small margin between the effective dose and the dose that may cause serious or toxic adverse effects. Furthermore, colchicine is metabolized by cytochrome P450 3A4 and P-glycoprotein, making it susceptible to drug-drug interactions. Therefore, close monitoring for adverse effects is essential. Thus, a highly individualized approach is warranted, limiting colchicine use to patients who remain at high risk despite OMT, until further evidence becomes available.

Among specific anti-inflammatory therapies, two therapeutic targets have gained prominence. The first is the inhibition of IL-1β, evaluated in the Canakinumab Anti-Inflammatory Thrombosis Outcomes Study (CANTOS) – a randomized, double-blind, placebo-controlled clinical trial that investigated the effect of canakinumab on reducing the recurrence of MACE. A total of 10,061 patients with a history of MI and hs-CRP ≥2 mg/L were randomized to receive canakinumab or placebo. The primary endpoint – a composite of CV death, nonfatal MI, or nonfatal ischemic stroke – was significantly reduced with canakinumab (HR 0.85; 95% CI 0.74-0.98; p = 0.021).^250^ Despite this evidence and the confirmation of the inflammatory theory of atherogenesis, canakinumab has not become a preventive therapy for atherosclerosis due to its high cost.

The blockade with a human monoclonal antibody targeting IL-6, ziltivekimab, was evaluated in the RESCUE trial, which included patients with moderate to severe CKD and hs-CRP levels above 2 mg/L.^251^ Dose-dependent reductions were observed in levels of hs-CRP, fibrinogen, haptoglobin, phospholipase A2, and Lp(a), indicating its anti-inflammatory action. As a consequence of these findings, a phase 3 randomized controlled trial – ZEUS (Zotarolimus-eluting Endeavor Sprint Stent in Uncertain DES Candidates) – is currently underway, enrolling approximately 5,000 patients with chronic CAD, CKD, and elevated hs-CRP levels (Table 19).

**Table 19:** Summary of anti-inflammatory therapy recommendations for patients with chronic coronary artery disease

Recommendation	Recommendation grade	Level of evidence
The addition of colchicine may be considered to reduce recurrent acute coronary syndrome events	**IIb**	**B**

### 4.6. Antidiabetic Therapy

Despite persistent efforts, MACE rates remain high, even among well-controlled individuals, and CAD continues to be the leading cause of morbidity and mortality.

In most patients, the situation is worsened by the lack of adoption of well-established therapeutic measures for type 2 diabetes. Within this scope of actions, lifestyle modifications and pharmacological therapy should be included to optimize the management of dyslipidemia, hypertension, weight control, and hyperglycemia. In addition to these measures, over the past decade, three classes of antidiabetic drugs have emerged as effective in reducing MACE, regardless of their effects on glycemia. These include pioglitazone, SGLT2 inhibitors (SGLT2i), and GLP-1 receptor agonists. These drugs reduce the risk of MACE through different mechanisms.

Pioglitazone has been tested as a preventive measure for atherosclerotic events in two clinical trials. In the first, the PROactive (PROspective pioglitAzone Clinical Trial In macroVascular Events) trial, 5,238 patients with type 2 diabetes and CAD were treated with pioglitazone (15-45 mg) or placebo in addition to standard therapy for type 2 diabetes. The treatment did not achieve significance in the primary endpoint – a composite of all-cause mortality, nonfatal MI (including silent MI), stroke, ACS, endovascular or surgical intervention in coronary or lower limb arteries, or above-ankle amputation (HR 0.90, 95% CI 0.80-1.02, p = 0.095). The secondary composite endpoint of CV death, nonfatal MI, and nonfatal stroke showed a 16% RRR with pioglitazone (HR 0.84, 95% CI 0.72-0.98, p = 0.027).^252^ In the IRIS (Insulin Resistance Intervention After Stroke) trial, 3,876 patients with a recent ischemic stroke or transient ischemic attack were randomized to receive pioglitazone (45 mg/day) or placebo. The primary endpoint – a composite of fatal or nonfatal stroke or fatal or nonfatal MI – was reduced by 2.8% after 4.8 years (HR 0.76; 95% CI 0.62-0.93; p = 0.007).^253^ Altogether, these findings indicate a reduction in atherosclerotic events with pioglitazone. However, in PROactive, there was a 2% increase in hospitalizations for HF in the pioglitazone group. Therefore, pioglitazone is not recommended for patients with HF.

Findings from clinical trials investigating SGLT2i suggest a smaller effect on the reduction of atherosclerotic events compared to their impact on the incidence or worsening of HF. However, given the early onset of the benefit on HF (approximately 30 days), the bias introduced by competing risks makes it difficult to estimate the true later effect on atherosclerotic event prevention. Traditional and network meta-analyses using random effects models – by amplifying statistical power – have confirmed the protective effect against atherosclerotic events, particularly in patients undergoing secondary prevention.^254,255^ Still, this effect may be underestimated due to early treatment discontinuation following short-term benefits related to HF outcomes.

Trials with GLP-1 receptor agonists have clearly demonstrated a reduction in the risk of atherosclerotic events, particularly in secondary prevention.^256,257^ More recently, a GLP-1 receptor agonist was compared to placebo in patients with atherosclerotic disease and obesity but without type 2 diabetes. Treatment with the GLP-1 agonist reduced the incidence of the composite endpoint – CV death, nonfatal MI, or nonfatal stroke – by 1.5% (HR 0.80; 95% CI, 0.72-0.90).^258^ Therefore, GLP-1 receptor agonist therapy has proven effective in reducing atherosclerotic MACE in both patients with type 2 diabetes undergoing secondary prevention and in those with obesity.

Given their distinct mechanisms of action, MACE risk reduction may be greater when combining classes of antidiabetic medications compared to using any of these agents in isolation. Data on concomitant use are primarily limited to safety and metabolic outcomes; however, network meta-analyses have reported an additive effect with the combination of pioglitazone and GLP-1 receptor agonists as well as with SGLT2i and GLP-1 receptor agonists.^259^ In light of the substantial residual risk observed in clinical trials testing each of these drugs individually, it is reasonable to consider initiating combination therapy from the outset in patients undergoing secondary prevention and with type 2 diabetes (Table 20).

**Table 20:** Summary of the indication of antidiabetic therapy in patients with chronic coronary artery disease

Recommendation	Recommendation grade	Level of evidence
In patients with type 2 diabetes, SGLT2 inhibitors should be used for secondary prevention of recurrent atherosclerotic events	**I**	**A**
In patients with type 2 diabetes, GLP-1 receptor agonists should be used for secondary prevention of recurrent atherosclerotic events	**I**	**A**
In patients with type 2 diabetes, the combination of SGLT2 inhibitors and GLP-1 receptor agonists should be considered to reduce residual risk	**I**	**C**
In patients with type 2 diabetes without HF, on combination therapy with SGLT2 inhibitors and GLP-1 receptor agonists, the addition of pioglitazone may be considered as adjunctive therapy for secondary prevention of recurrent atherosclerotic events	**IIa**	**B**

HF: heart failure.

## 5. Clinical-Pharmacological Treatment for Optimal Symptom Control

Pharmacological treatment in CCS aims to reduce angina symptoms and exercise-induced ischemia, prevent the development of ventricular dysfunction, and reduce MACE. Clinical treatment should be prioritized in CCS, with lifestyle modification and OMT, which can be defined as therapy that satisfactorily controls symptoms and prevents MACE associated with CCS, with maximum patient adherence and minimal adverse effects. This treatment includes medications that provide plaque stabilization and reduce events as well as drugs that help reduce the incidence of angina. Importantly, there is no universal definition of what constitutes the "ideal" antianginal drugs for optimal treatment in patients with CCS, and pharmacologic therapies should be tailored to each patient's characteristics and preferences as well as the physician's experience.^1^

Initial antianginal therapy typically consists of one or two medications, as needed. The initial choice depends on expected tolerance, potential drug interactions, patient preferences, and drug availability. Treatment costs must also be taken into account, especially in the context of public health care systems. Therefore, antianginal treatment should always be individualized for each patient. The actions, contraindications, and usage guidelines for each antianginal drug are described below.

### 5.1. Beta-Blockers

Beta-blockers (BBs) bind to adrenergic receptors via G proteins, thereby inhibiting the effects of adrenaline and noradrenaline on these receptors.^260^ In patients with angina pectoris, beta-blockade reduces ischemia and improves QoL by decreasing heart rate (HR) and myocardial contractility, especially during exercise. It also helps prevent effort-induced increases in BP. Additionally, BBs enhance perfusion in ischemic areas by prolonging diastolic time and increasing vascular resistance in nonischemic regions. The dose of BBs should be adjusted to target a resting HR of 55 to 60 bpm.^261^ If discontinuation is necessary, it should be done gradually, not abruptly.

There are several subtypes of BBs: nonselective without intrinsic sympathomimetic activity (propranolol and sotalol); nonselective with intrinsic sympathomimetic activity (pindolol); beta-1-selective (atenolol, bisoprolol, and metoprolol); beta-1-selective with alpha-blocking activity (carvedilol); and beta-1-selective with nitric oxide-mediated vasodilatory properties (nebivolol).^262^

BBs remain the most commonly used first-line treatment, due to their long history of use, affordability, and their proven benefits in reducing mortality in patients with HF. In patients with HF with reduced ejection fraction (HFrEF), BBs have been associated with a significant reduction in mortality and/or MACE.^263–265^

BBs have also been associated with a lower risk of MACE and all-cause mortality in patients undergoing CABG.^266^

Following MI, older studies suggested that routine use of BBs was linked to a lower incidence of MACE.^267^ However, most of the trials showing benefit from BB therapy after MI included patients with large infarctions and were conducted in an era before the modern diagnosis of MI using biomarkers, as well as before treatment with PCI, antithrombotic agents, high-intensity statins, and RAAS antagonists. By contrast, the REDUCE-AMI trial, published in 2024, evaluated patients with acute MI who underwent early coronary angiography and had preserved LVEF (≥ 50%). In this study, prolonged BB therapy did not reduce the risk of the primary composite endpoint of all-cause mortality or recurrent MI compared to patients who did not receive BBs.^268^

Furthermore, in patients with CCS without HFrEF or without a MI in the past year, studies in general have not demonstrated a benefit of BBs in reducing mortality and/or MACE.^369^

Randomized clinical trials evaluating the effects of BBs in the treatment of CCS in the presence of symptoms or ischemia have shown a significant reduction in the number of angina episodes, ischemia, and an increase in exercise tolerance.^270–272^ When compared with other medications, the anti-anginal effects of BBs are similar to those of other anti-ischemic drugs.^273–275^ However, combining different anti-anginal medications, including BBs, can provide additional benefits and should be encouraged as a therapeutic strategy.^260^

Caution is advised when combining a BB with verapamil or diltiazem due to the risk of worsening HF, excessive bradycardia, and/or atrioventricular (AV) block. However, BBs can be combined with ivabradine.^276,277^ The main side effects of BBs include fatigue, depression, bradycardia, heart block, bronchospasm, peripheral vasoconstriction, postural hypotension, impotence, and masking of hypoglycemia symptoms.^260,262^ They should not be prescribed to patients with CVS since they may precipitate vasospasm due to increased alpha-adrenergic activity.

### 5.2. Trimetazidine

Trimetazidine is a clinically effective antianginal agent that has no effect on CV hemodynamics, with no influence on BP or HR. This is particularly useful in patients with controlled double product who remain symptomatic.

Trimetazidine works by inhibiting the enzyme 3-ketoacyl-CoA thiolase, which normally catalyzes the final step of fatty acid beta-oxidation in the myocardium. By inhibiting this enzyme, trimetazidine reduces the use of fatty acids as an energy source in the heart and increases the use of glucose. This improves myocardial energy efficiency, reducing oxygen demand and increasing ischemic tolerance.

The clinical antianginal effects of trimetazidine have been tested in studies on patients with chronic ischemia, both as monotherapy and in combination with other antianginal drugs such as calcium channel blockers (CCBs) and BBs.^275^ When used alone, its beneficial effects were similar to monotherapy with BBs or CCBs in the treatment of CCS.

In a meta-analysis, trimetazidine significantly improved exercise tolerance, reduced weekly episodes of angina, and reduced the use of short-acting nitrates compared to placebo.^280^ Furthermore, real-world studies have shown that the use of trimetazidine in combination with antianginal drugs with hemodynamic effects reduces the number of weekly angina episodes and also reduces the use of sublingual nitrates.^281,282^

There are two different commercial formulations: trimetazidine 35 mg (to be taken twice daily) and trimetazidine 80 mg (to be taken once daily).

Adverse effects related to treatment are generally mild and well tolerated, mainly including gastrointestinal disturbances such as nausea and vomiting, and headache. In the ATPCI (efficAcy and safety of Trimetazidine in patients with angina pectoris treated by Percutaneous Coronary Intervention) trial, trimetazidine was not associated with any adverse events compared to placebo. The incidence of adjudicated adverse events was low and well balanced between treatment groups. There was no increase in the occurrence of neurological symptoms such as Parkinson's disease, atypical parkinsonism, or drug-induced parkinsonism when comparing the placebo and trimetazidine arms.^283^ n that study, the use of trimetazidine compared to placebo did not reduce MACE in patients under optimal treatment and after successful PCI.

Trimetazidine should not be prescribed when the GFR is < 30 mL/min/1.73 m^2^. In patients with a GFR < 60 mL/min/1.73 m^2^, the dose of trimetazidine should be adjusted to 35 mg once daily. Recently, a Brazilian study confirmed the reduction of angina and improvement in QoL in the V-GOOD observational study with trimetazidine 80 mg sustained-release taken once daily.^284^

### 5.3. Calcium Channel Blockers

This is a heterogeneous group of drugs with the following pharmacological effects: smooth muscle relaxation, afterload reduction, negative inotropic effects (verapamil and diltiazem), and decreased oxygen consumption. The three main subgroups of CCBs that specifically block L-type calcium channels include dihydropyridine derivatives (nifedipine, amlodipine, and others), benzothiazepines (diltiazem), and phenylalkylamines (verapamil).

Verapamil reduces AV conduction, has a negative inotropic effect, and relaxes vascular smooth muscle, increasing coronary flow and reducing afterload. Dihydropyridines relax vascular smooth muscle, do not affect AV conduction velocity, and increase HR through reflex mechanisms. Diltiazem has effects similar to verapamil, except that its myocardial depressant effect is less intense.

CCBs improve symptoms and myocardial ischemia, but have not been shown to reduce the major morbidity and mortality outcomes in patients with CCS.^260,285–287^ This drug class is indicated for all forms of angina, including CVS, with some individual characteristics.

### 5.4. Verapamil

Verapamil is generally safe, but it can cause heart block, bradycardia, and HF. Combining it with BBs is not recommended due to the risk of AV block.

### 5.5. Diltiazem

Diltiazem is well tolerated and offers advantages over verapamil in the treatment of both exertional and CVS.^288^ It has a mild negative inotropic effect and inhibits the sinoatrial node.

The combination of nondihydropyridine CCBs with BBs may be used in selected patients, provided that HR is closely monitored. These drugs should not be used in patients with ventricular dysfunction.

### 5.6. Dihydropyridines

The most commonly used dihydropyridines are long-acting nifedipine and amlodipine. Both are generally well tolerated, with peripheral edema being the most common adverse effect, which may limit their use in some cases. When needed, combining them with BBs is recommended and has demonstrated greater efficacy.^289^

### 5.7. Ivabradine

Ivabradine reduces HR by inhibiting the I_f_ current in the sinoatrial node, thereby prolonging diastole and improving perfusion time, without affecting BP, vascular tone, or LV systolic function.^1,290–293^ When combined with BBs, ivabradine not only lowers HR, angina episodes, and nitrate consumption, but also increases exercise tolerance, prolongs total exercise duration in stress testing, and improves QoL in patients with angina.^276,294^ Ivabradine also enhances CFR and appears to positively influence collateral function and myocardial perfusion in patients with angina due to CCS.^295–298^

Ivabradine did not reduce clinical outcomes in patients with angina and either preserved or reduced ventricular function in the BEAUTIFUL (morBidity-mortality EvAlUaTion of the If inhibitor ivabradine in patients with coronary disease and left-ventricULar dysfunction) and SIGNIFY (Study Assessing the Morbidity–Mortality Benefits of the I_f_ Inhibitor Ivabradine in Patients with Coronary Artery Disease) trials, respectively.^299,300^ However, it did reduce mortality and hospitalizations for HF in patients with reduced ventricular function in the SHIFT trial.^301^ The increased risk of CV death and nonfatal MI observed in a subgroup of patients with limiting angina in the SIGNIFY trial raised safety concerns. This result may be explained by the use of a higher ivabradine dose (10 mg twice daily), a lower target HR (60 bpm), and concomitant use of nondihydropyridine CCBs, which impair ivabradine metabolism through CYP3A4 inhibition.^300^ On the other hand, in the BEAUTIFUL trial, ivabradine reduced the risk of MACE in the subgroup of patients with limiting angina.^299^

In a pooled analysis of observational studies, ivabradine was found to be safe and effective when combined with BBs.^302^ In addition to its safety, it offers additional benefits when used with other antianginal agents, although its combination with verapamil or diltiazem should be avoided.^1,303^

The most common adverse effects include visual disturbances (phosphenes), headache, dizziness, bradycardia, AF, and AV block. Ivabradine is contraindicated in patients with HR <70 bpm, in acute MI, and in severe hepatic impairment. Drug interactions may occur with agents that prolong the QTc interval and with strong CYP3A4 inhibitors (CYP3A4i).^1^

The initial dose should not exceed 5 mg twice daily, and the maintenance dose should not exceed 7.5 mg twice daily. Discontinuation should be considered if there is no symptom improvement or clinically significant HR reduction within 3 months of treatment initiation. If HR falls below 50 bpm or if symptoms related to bradycardia develop during treatment, the dose should be reduced to 2.5 mg twice daily. If symptoms persist or HR remains <50 bpm despite dose reduction, treatment should be discontinued.^303^

### 5.8. Ranolazine

Ranolazine is a piperazine-derived compound that exerts its effects primarily by reducing calcium overload in ischemic myocytes through inhibition of the late sodium current (I_Na_).^304^ Animal models also suggest a potential mechanism involving increased adenosine levels.^305^

Several clinical trials have failed to demonstrate an association between ranolazine use and a reduction in CV outcomes such as MI, CV death, or the need for revascularization.^306–308^ Although there is no evidence supporting the benefit of ranolazine in reducing MACE, clinical trials have shown that adding ranolazine to other antianginal therapies significantly reduces the frequency of angina episodes and the use of sublingual nitrates,^319,310^ delays progression in angina functional class,^307^ lowers angina recurrence, and increases exercise tolerance as measured by treadmill testing.^308,311^ These findings are supported by systematic reviews confirming the efficacy of ranolazine in reducing angina symptoms.^274^ However, ranolazine as monotherapy does not appear to offer significant clinical benefit in patients with CCS.^274^

Data from small studies suggest that ranolazine may be particularly effective in reducing angina symptoms in patients without significant coronary lesions, where chest pain is associated with MVD.^312,313^ A quasi-experimental study has also suggested an antianginal effect in patients with hypertrophic cardiomyopathy,^314^ and a case report proposed its potential to reduce CVS.^315^

Ranolazine may also have potential benefits for patients with diabetes. In a clinical trial involving diabetic patients, 24 weeks of ranolazine monotherapy resulted in HbA1c levels below 7.0% in 41.2% of patients, compared to 25.6% in the placebo group. This effect appears to be due to inhibition of glucagon secretion by pancreatic alpha-cells through sodium channel blockade and suppression of electrical activity.^316^ Another clinical trial in patients with diabetes and CCS demonstrated that an 8-week course of ranolazine significantly reduced the frequency of angina episodes and the need for sublingual nitrates, without increasing adverse effects. This benefit may be explained by a more effective action on slow sodium channels in patients with poor glycemic control.^309^

Another noteworthy aspect is the potential antiarrhythmic effect of ranolazine. Although its efficacy in patients with non-ST-elevation ACS has not been conclusively proven, ranolazine use in this setting significantly reduced episodes of nonsustained ventricular tachycardia lasting at least 8 bpm.^317^ Additionally, a small uncontrolled study found that high-dose oral ranolazine was a safe and potentially effective option for converting acute or paroxysmal AF to sinus rhythm, with a conversion rate of 72% and no significant adverse events.^318^ These findings suggest a possible antiarrhythmic role for ranolazine, particularly in patients with angina and paroxysmal AF.

There is a 22% increase in the incidence of adverse events with the addition of ranolazine.^332^ Most side effects, however, are not severe, with the most common being constipation, headache, dizziness, dyspepsia, abdominal pain, nausea, and asthenia.^304,311^ There have also been reports of increased incidence of syncope, likely vasovagal in origin as well as a slight prolongation of the QT interval.^307,311^ Therefore, QTc interval monitoring is recommended before and during ranolazine therapy.

Ranolazine is primarily metabolized by the CYP3A4 and CYP2D6 enzymes, with only a small fraction excreted unchanged in the urine. It is contraindicated in patients with hepatic impairment, renal failure, QTc prolongation, or those taking drugs known to prolong the QT interval. Multiple drug interactions limit its use: it acts as a weak inhibitor of CYP3A, which increases plasma concentrations of simvastatin, and also raises digoxin levels through inhibition of P-glycoprotein. Importantly, coadministration of rosuvastatin, atorvastatin, pitavastatin, fluvastatin, or pravastatin with ranolazine may be considered when clinically indicated. However, if simvastatin is co-prescribed, its dose should be limited to 20 mg per day. In addition to increasing the plasma exposure of other drugs, ranolazine concentrations are elevated by CYP3Ai such as ketoconazole, diltiazem, and verapamil.^304,319,320^

Since ranolazine does not lower HR or BP, it is a suitable option for patients who are hypotensive or who continue to experience angina despite reduced HR due to the use of BBs. The recommended starting dose is 500 mg twice daily, which may be increased to 1,000 mg twice daily if well tolerated.^274,304^

### 5.9. Short- and Long-Acting Nitrates

Nitrates relieve angina through coronary and peripheral vasodilation mediated by nitric oxide, leading to redistribution of coronary blood flow and reductions in systemic vascular resistance and preload.^321^ In Brazil, the short-acting nitrates available are isosorbide dinitrate and propatylnitrate, both in sublingual formulations. These drugs are the standard therapy for immediate relief of effort- and vasospastic-induced angina, with onset of action within minutes and duration of less than 1 hour. The dose may be repeated at 5-minute intervals until symptom relief or until a maximum of 15 mg is reached within 15 minutes, in the case of isosorbide dinitrate. Patients should remain seated while taking the medication, as standing increases the risk of syncope and lying down increases venous return and preload. Short-acting nitrates are also recommended prophylactically in situations likely to provoke angina, such as physical exertion, emotional stress, sexual activity, postprandial periods, or exposure to cold weather.^322^

Long-acting nitrates have been shown to improve exercise tolerance, time to ST-segment depression, and time to onset of angina in patients with CCS, although evidence is limited to studies with small sample sizes and relatively short follow-up.^322–324^

However, there are limitations and concerns regarding the use of long-acting nitrates. When used continuously over an extended period, tolerance develops, leading to reduced efficacy. Therefore, a nitrate-free interval of 10 to 14 hours is required.^324^ Additionally, discontinuation should be gradual rather than abrupt to avoid rebound angina. Long-term nitrate therapy has been associated with endothelial dysfunction due to the accumulation of oxygen free radicals, which increase arterial sensitivity to sympathetic stimulation and vasoconstrictors.^325^

Regarding CV outcomes, the most robust evidence demonstrated higher event rates (e.g., death, nonfatal MI, and HF) associated with long-acting nitrate use.^326^ Furthermore, studies in patients with CVS have also shown increased MACE with long-acting nitrates.^327,328^ The most common side effects are hypotension, headache, and flushing. Contraindications include hypertrophic obstructive cardiomyopathy, severe aortic valve stenosis, and concomitant use of phosphodiesterase inhibitors.^329,330^

### 5.10. Allopurinol

High doses of allopurinol (600 mg per day), a xanthine oxidase inhibitor, have been associated with improvement in angina symptoms and exercise test ischemic parameters in a few small randomized clinical trials involving a very limited number of patients.^331,332^ A large randomized clinical trial including over 5,000 patients and with a mean follow-up of nearly 5 years, recently published, demonstrated that allopurinol did not reduce MACE in patients with CCS and no prior history of gout. There was no evidence of a difference in the incidence of serious adverse events between groups, but also no benefit in quality-of-life measures or angina questionnaire scores with allopurinol.^333^

### 5.11. Individualized Antianginal Treatment

International guidelines have traditionally recommended drugs for angina management by classifying them as first-line or second-line agents.^1^ The 2014 SBC Guideline on Stable Coronary Disease also categorized drugs into first-, second-, third-, and even fourth-line options.^16^

However, among the currently recommended antianginal agents, there is no evidence demonstrating the superiority of one drug class over another in terms of angina relief or other outcomes.^260,334^ A systematic review of studies published over the last 50 years included randomized, double-blind trials with parallel-group comparisons of angina treatment in patients with CCS. None of the studies in this review demonstrated that one drug was superior to another for relieving angina or increasing total exercise time.^334^

Newer antianginal medications such as ivabradine, ranolazine, and trimetazidine have substantial evidence from contemporary randomized clinical trials supporting their safety and efficacy.^335,336^

As a result, an individualized approach has been proposed for patients with angina, tailoring antianginal therapy to each patient's clinical characteristics, hemodynamic profile, comorbidities, and the underlying mechanism of ischemia, without classifying therapies as first- or second-line.^276,335,336^

Moreover, early combination therapy with two or three antianginal agents that act through different and complementary mechanisms is often necessary to achieve symptom control, as patients with angina frequently present with multiple comorbidities and overlapping mechanisms of ischemia.^335^ This strategy can reduce angina frequency, decrease nitrate consumption, and increase exercise duration and time to onset of angina or ischemia during stress testing.^1^

Regardless of the initial strategy, the response to antianginal therapy should be reassessed 30 days after treatment initiation.^1^ If available, telemedicine may be offered for earlier follow-up.

Since angina symptoms may recur or remit over time and coronary plaques may become quiescent, appropriate assessment of angina requires careful monitoring and systematic evaluation of patient-reported symptoms and QoL.^27^Accordingly, a sufficient time horizon (3 to 6 months) is often necessary for evidence-based medical therapy to be properly assessed in terms of effectiveness.^27,337^

Individualized approach (Figure 17):

**Figure 17:** Brazilian approach to personalized treatment of angina. Personalized treatment approach for angina, considering ejection fraction, BP, HR, and the underlying mechanisms of myocardial ischemia. Antianginal agents in each section are listed in alphabetical order. Green indicates the preferred antianginal agents for each clinical scenario; blue indicates agents that may be added for an additive effect in symptom control. EF, ejection fraction; BP, blood pressure (in mm Hg); HR, heart rate (in bpm); CCB, calcium channel blockers; INOCA, ischemia with nonobstructive coronary arteries. *Should not be used in combination with ivabradine or dihydropyridine CCBs. **Should not be used when HR ≤ 55 bpm. The colors in this figure do not represent recommendation grades or levels of evidence.

Specific drugs or drug combinations are preferred depending on the underlying pathophysiology and comorbidities present.^335^

Elevated HR: HR-reducing medications such as BBs, nondihydropyridine CCBs (verapamil and diltiazem), and ivabradine are the preferred agents when the HR is > 70 bpm (Figure 17). The addition of ivabradine to a BB is safe and beneficial when the HR remains > 70 bpm.^276^ However, the combination of ivabradine with diltiazem or verapamil is contraindicated.^303^ The use of BBs in combination with verapamil or diltiazem should be approached with caution due to the risk of high-grade AV block. Ranolazine and trimetazidine may be coadministered. The dosage of antianginal medications should be reduced if the HR drops below 50 bpm.

Low HR (Figure 17): HR-reducing medications should be avoided when the HR is < 55 bpm. Dihydropyridine CCBs and nitrates are preferred, as these agents may increase HR through reflex sympathetic activation. Other agents such as ranolazine and trimetazidine may also be considered.

Arterial hypertension: BBs and dihydropyridine CCBs are preferred in patients with hypertension (Figure 17). BP should not be reduced below 120/70 mm Hg, as lower levels have been associated with an increased risk of MACE in patients with CCS.^338^ However, it remains unclear whether this J-shaped relationship also applies to revascularized patients.

Arterial hypotension: Antianginal agents that significantly lower BP, such as CCBs, nitrates, and BBs, should be avoided in patients with angina and low BP, as they may worsen coronary perfusion. In an analysis of over 22,000 patients from the CLARIFY study, systolic BP < 120 mm Hg and diastolic BP < 70 mm Hg were associated with increased risk of CV mortality, MI, and stroke.^338^ In such patients, preferred agents include ivabradine (if HR is also elevated), trimetazidine, or ranolazine (Figure 17).

LV dysfunction and HF: When angina is present in patients with LV dysfunction, with or without HF, the evidence-based recommendation is the use of BBs (Figure 17), which not only alleviate angina but also effectively reduce CV morbidity and mortality in these patients.^339,340^ These benefits appear to be directly related to the HR-lowering effect of BBs; therefore, BBs with intrinsic sympathomimetic activity should be avoided. If HR remains > 70 bpm despite the maximum tolerated dose of BB, ivabradine should be considered. The SHIFT (Systolic Heart Failure Treatment with the I_f_ Inhibitor Ivabradine Trial)^301^ demonstrated an additional prognostic benefit from adding ivabradine to evidence-based OMT in patients with HF and reduced EF. Similar benefits were observed in the subgroup of patients with angina.^341^ Diltiazem and verapamil should not be used in this population, as they may worsen LV dysfunction.^342^ Dihydropyridine CCBs may be used with caution. A meta-analysis of small studies in patients with LV dysfunction and/or HF suggests that trimetazidine may be beneficial when added to recommended therapies.^343^

AF: AF may worsen anginal symptoms due to increased HR. Therefore, BBs and nondihydropyridine CCBs are preferred when this comorbidity is present. Due to its selective action on I_f_ channels, ivabradine is ineffective in patients with AF and may even increase the risk of this arrhythmia. A meta-analysis showed that treatment with ivabradine is associated with a 1.15-fold increase in the relative risk of AF.^344^ Dihydropyridine CCBs and nitrates should be avoided because they may lead to an increase in HR. The addition of ranolazine, which has demonstrated the ability to suppress supraventricular arrhythmias and AF, may be useful. Trimetazidine may also be considered.^317,345^

Diabetes: The treatment of angina in patients with diabetes requires drugs with either neutral or favorable metabolic effects. The efficacy of ranolazine in diabetic patients with angina was evaluated in a double-blind, placebo-controlled trial.^309^ Ranolazine significantly reduced HbA1c levels, fasting glucose, and 2-hour postprandial glucose levels, while also decreasing the frequency of angina and increasing exercise tolerance.^309^ Ranolazine should be considered the preferred agent in this subgroup of patients.^309,346^ Trimetazidine may also have beneficial effects by improving glucose utilization under ischemic conditions, and some positive data have been reported in diabetic subgroups of trimetazidine trials.^347^ However, these findings are derived from open-label studies with small diabetic populations. Traditionally, BBs have been associated with the onset of new cases of diabetes and worsening glycemic control.^348^ Nevertheless, vasodilating BBs such as carvedilol and nebivolol have been linked to improved insulin sensitivity, thereby overcoming the metabolic limitations of traditional BBs. Other agents may be used to reduce angina and ischemia.^349^

CKD: Clinical trials often exclude patients with CKD, a common comorbidity in those with CAD, resulting in limited data on the efficacy of antianginal agents in this population.^349^ Ranolazine and trimetazidine should not be prescribed when the eGFR is < 30 mL/min/1.73 m^2^. There are no contraindications for other antianginal drugs.^335^

Chronic obstructive pulmonary disease (COPD): Evidence suggests that cardioselective (beta-1 selective) BBs are generally well tolerated in patients with COPD and may improve survival.^350^ Due to its high beta-1 selectivity, bisoprolol is the only BB not contraindicated in COPD. However, the coexistence of asthma or COPD with bronchial hyperreactivity constitutes a contraindication to BBs. In such patients, when HR reduction is needed, ivabradine, diltiazem, or verapamil are preferred. In cases of pulmonary hypertension and right ventricular (RV) dysfunction, nondihydropyridine CCBs and nonselective BBs are not recommended.^335^

PAD: In 2013, the British Medical Association stated that BBs were contraindicated in severe PAD.^351^ In the same year, a Cochrane systematic review found no strong evidence either for or against the use of BBs in PAD.^352^ Due to the lack of reliable and contemporary data, BBs should be avoided or used with caution in patients with angina and PAD.^335^ Similarly (particularly in cases of critical limb ischemia), vasodilators such as CCBs and nitrates should be avoided, as acute reductions in BP may be harmful. Other antianginal agents (e.g., ivabradine, ranolazine, and trimetazidine) are preferred.^335^

AV conduction defects: BBs and nondihydropyridine CCBs reduce AV conduction and may cause complete AV block and intraventricular dyssynchrony in patients with conduction abnormalities.^353^ These drugs are clearly contraindicated in patients with second-degree AV block. Alternative antianginal agents should be used in such cases.^335^

Hyperthyroidism: Patients with hyperthyroidism have a threefold increased risk of AF and HF.^354^ Elevated thyroid hormone levels also affect multiple factors that increase myocardial oxygen demand, leading to angina in patients with or without angiographically documented CAD. Thyroid hormones may also trigger coronary artery spasm (CAS).^355^ Preferred treatments in these patients include nonselective BBs (e.g., propranolol), diltiazem, verapamil, or ivabradine when BBs are contraindicated. Vasodilators should be avoided due to the risk of reflex tachycardia.^335^

CVS: CCBs are the preferred agents for preventing and/or treating CAS. Long-acting nitrates may be used in patients who remain symptomatic (Figure 17). All CCBs can prevent coronary spasm in approximately 90% of patients. Long-acting nitrates are effective, but intermittent administration is important to prevent nitrate tolerance. BBs are contraindicated because they may worsen vasospasm by allowing unopposed alpha-mediated vasoconstriction in the absence of beta-mediated vasodilation. In patients with refractory angina (RA), high doses of CCBs may be considered.^335^

Microvascular angina: No conclusive evidence is currently available to support a specific class of antianginal agents, likely due to the limited understanding of the underlying causes of microvascular angina and the variable response to different treatments. For many years, traditional agents such as BBs, CCBs, and nitrates were considered the only available options, despite 20% to 30% of patients remaining symptomatic. BBs may be preferred when there is evidence of increased adrenergic activity.^1^ Ranolazine may also help reduce mechanical compression of the coronary microcirculation^356,357^ and improve coronary autoregulation.^358^ A small study with ranolazine suggested symptom improvement in women with microvascular angina.^359^ Subsequently, in a double-blind, randomized, placebo-controlled trial involving women with exertional angina and no obstructive CAD, ranolazine did not show significant benefits – except in patients with impaired CFR.^360^ Similarly, studies have shown that ivabradine improves collateral flow and CFR in patients with microvascular angina. Ivabradine outperformed bisoprolol, even though both reduced HR similarly. Therefore, the treatment of microvascular angina remains challenging and necessarily empirical. HR reduction using BBs, nondihydropyridine CCBs (e.g., diltiazem and verapamil), or ivabradine may be considered to prolong diastolic duration and enhance coronary perfusion. The coadministration of ranolazine or trimetazidine may also be beneficial (Figure 1). In patients with increased pain perception, adenosine antagonists and drugs used in chronic pain syndromes, such as imipramine, are therapeutic options.^335^

The effectiveness of a personalized treatment strategy for patients with angina and INOCA was evaluated in the CorMiCa (CORonary MICrovascular Angina) trial, which randomized 151 patients to either stratified OMT (based on CFR, IMR, and acetylcholine testing) or standard care (including a sham diagnostic procedure). Patients were diagnosed with microvascular angina, CVS, or noncardiac chest pain. After 1 year, there was a significant improvement in angina scores favoring the stratified treatment group.^361^

The initial choice of antianginal therapy should be reevaluated at each visit. This evaluation should consider changes in the patient's clinical status, such as BP, HR, and ventricular function; the mechanisms of angina and ischemia; the presence of comorbidities; medication tolerance; potential drug interactions; and the emergence of adverse effects (Table 21).

**Table 21:** Clinical (pharmacological) treatment for optimal symptom control

Recommendation	Recommendation grade	Level of evidence
The initial choice of antianginal therapy should be individualized, considering comorbidities, hemodynamic profile, tolerability, drug interactions, physician and patient preferences, and drug availability.	**I**	**C**
The combination of antianginal agents with different mechanisms of action is more effective for symptom control than monotherapy with a single agent.	**I**	**C**
While the patient remains symptomatic, clinical reassessment should be performed every 30 days, with progressive adjustments in antianginal therapy.	**I**	**C**
If available, telemedicine may be offered for more frequent follow-up.	**IIa**	**C**
Bisoprolol, carvedilol, and metoprolol succinate are recommended for the treatment of angina in patients with LVEF ≤ 40%.	**I**	**A**
Short-acting nitrates are recommended for immediate relief of exertional angina.	**I**	**B**
Beta-blockers, calcium channel blockers, trimetazidine, and/or ranolazine are options for the treatment of angina (either as monotherapy or in combination).	**I**	**B**
Ivabradine should be considered for angina treatment in patients with ventricular dysfunction who remain with HR ≥70 bpm despite beta-blocker therapy or when beta-blockers are contraindicated.	**IIa**	**B**
Ivabradine may be considered for angina treatment in patients without ventricular dysfunction, with HR ≥70 bpm and sinus rhythm.	**IIb**	**B**
Long-acting nitrates may be considered for angina treatment when the patient remains symptomatic despite other antianginal therapies.	**IIb**	**B**
Long-acting nitrates should not be used as monotherapy, except when other antianginal agents are not feasible.	**III**	**C**

HR: heart rate; LVEF: left ventricular ejection fraction.

## 6. Invasive Treatment Strategies

### 6.1. Percutaneous Treatment for Symptom Control and Risk Reduction

#### 6.1.1. Angiographic Complexity Scores in Decision-Making

Total atherosclerotic burden, the extent of myocardium at risk, lesion complexity, the likelihood of achieving complete revascularization, residual risk, and the probability of clinical events are all critical elements in determining the most appropriate revascularization strategy for patients with CAD.^362,363^ Multiple factors contribute to the assessment of CAD complexity (Table 22).

**Table 22:** Angiographic features that contribute to increased coronary artery disease complexity

Multivessel coronary artery disease
Large total area of myocardium at risk (e.g., left main trunk or proximal left anterior descending lesion)
Diffuse disease in a target coronary vessel
Chronic total occlusion
Complex bifurcations or trifurcations
Severe calcifications
Marked vessel tortuosity
Total lesion length > 20 mm

The SYNTAX score, developed from the SYNTAX trial, plays a central role in this process by offering an objective tool to quantify total atherosclerotic burden and the anatomical complexity of CAD in patients with multivessel disease.^363^ Moreover, the SYNTAX score has been validated as an independent predictor of long-term MACE and mortality within the original SYNTAX (SYNergy between percutaneous coronary intervention with TAXus and cardiac surgery) cohort and in external studies involving patients undergoing PCI.^362^ However, the SYNTAX score is not suitable as a risk stratification tool for patients undergoing CABG.^364^

More recently, the retrospective development of the SYNTAX Score II and its revised 2020 version, derived from the same SYNTAX cohort, incorporated additional clinical variables into the anatomical model, allowing for a more comprehensive assessment.^365,366^ Although these scores demonstrate only modest discriminatory power in predicting post-revascularization clinical events, they remain valuable tools to support clinical decision-making.^364,366–368^

##### 6.1.1.1. Recommendation

The SYNTAX score remains the most widely used and validated risk score to guide revascularization strategies in patients with multivessel disease. Nonetheless, important limitations include the complex scoring system required for each lesion and high interobserver variability in its calculation.^369^ Furthermore, the absence of clinical variables limits its usefulness for estimating the risk of clinical events following CABG (Table 23).

**Table 23:** Recommendation for use of the SYNTAX score to estimate angiographic complexity

Recommendation	Recommendation grade	Level of evidence
In patients with multivessel coronary artery disease, assessment of disease complexity – such as with the SYNTAX score – may be useful for estimating event risk and guiding coronary artery bypass grafting	**IIb**	**B**

When evaluating disease complexity, it is important to account for factors that increase lesion complexity, as these can impact both the feasibility and the outcomes of revascularization.^367,370^

### 6.2. Invasive Functional Assessment

#### 6.2.1. Assessment of Epicardial Coronary Stenoses

FFR is an invasive technique used to evaluate the functional severity of coronary stenoses. It is based on the measurement of intracoronary pressure during maximal hyperemia, which is induced by the administration of vasodilators such as adenosine.^371^

FFR is defined as the ratio of the distal coronary pressure to aortic pressure and reflects the stenosis-induced limitation of blood flow. An FFR value ≤0.80 is generally considered indicative of significant ischemia and supports the decision for CABG. FFR is currently the gold standard for functional assessment of coronary lesions in patients with intermediate-grade stenosis (typically between 40% and 70%) in the absence of ischemia on noninvasive testing, or in patients with multivessel disease.

In addition to FFR, other pressure-derived indices – such as the instantaneous wave-free ratio (iFR), diastolic pressure ratio (dPR), resting full-cycle ratio (RFR), and diastolic FFR (DFR) – have proven useful for invasive functional assessment. These non-hyperemic indices have the advantage of not requiring pharmacologic induction of maximal hyperemia, offering a faster, more comfortable evaluation for the patient. Comparative studies suggest that these indices have good accuracy in identifying ischemia-producing lesions when compared to FFR.^372^

More recently, functional assessments have been derived from ICA. The quantitative flow ratio (QFR) and the Murray-based quantitative flow ratio (*μ*FR) – developed by Medis (Netherlands) and Pulse Medical (China), respectively – are techniques that calculate a physiological index based on angiographic imaging alone, without the need for a pressure wire or adenosine administration.

QFR/*μ*FR is the only angiography-based physiological index that has been prospectively validated.^373^ This approach has demonstrated good concordance with FFR and has been associated with improved clinical outcomes when used to guide coronary revascularization decisions, compared with conventional angiographic assessment.^373,374^

##### 6.2.1.1. Recommendation

In the FAME 2 (Fractional Flow Reserve Versus Angiography for Multivessel Evaluation 2) trial, patients with chronic CAD and lesions with angiographic severity ≥ 50% and FFR ≤ 0.80 were randomized to receive either medical therapy alone or PCI plus medical therapy.^145^ PCI significantly reduced the primary composite endpoint of death, MI, or urgent revascularization.^145^ In patients with multivessel CAD, FFR is particularly useful in guiding decisions about which lesions require revascularization. An FFR-guided approach allows for a more precise strategy, focusing only on lesions causing significant ischemia, potentially resulting in fewer stent implantations and lower complication rates.^375^

Two recent large randomized clinical trials demonstrated broadly comparable outcomes between revascularization strategies guided by FFR and iFR in patients with intermediate-grade stenoses. In both trials, revascularization was indicated if FFR was < 0.80 or if iFR was < 0.89. In the DEFINE-FLAIR (Functional Lesion Assessment of Intermediate Stenosis to Guide Revascularisation) trial, the primary endpoint of MACE at 1 year occurred in 6.8% of patients randomized to iFR-guided revascularization versus 7.0% in those randomized to FFR-guided revascularization (p < 0.001 for noninferiority; HR 0.95, 95% CI 0.68-1.33; p = 0.78). HR 1.12, 95% CI 0.79-1.58; p = 0.53.^376^

In the SWEDEHEART trial, after 5 years of follow-up, the primary endpoint of all-cause death, nonfatal MI, or unplanned revascularization occurred in 21.5% of the iFR group and 19.9% of the FFR group (HR 1.09; 95% CI 0.90-1.33).^377^

The FAVOR III China trial aimed to determine whether clinical outcomes could be improved by selecting lesions for PCI using QFR/*μ*FR versus visual assessment. The primary endpoint – composite of all-cause death, MI, or ischemia-driven revascularization – at 1 year occurred in 5.8% of the physiology-guided group versus 8.8% in the angiography-guided group (HR 0.65, 95% CI 0.51-0.83; p = 0.0004), driven by lower rates of MI and ischemia-driven revascularization in the QFR-guided group compared to the angiography-guided group.

#### 6.2.2. Microvascular Angina

Patients with microvascular angina typically present with exertional chest pain and evidence of ischemia on noninvasive testing in the absence of obstructive lesions or with mild to moderate stenoses (40%-60%) on ICA.

The presence of microcirculatory dysfunction in patients with angina carries prognostic implications, likely because most of the recent evidence has been derived from studies in which microvascular abnormalities were objectively documented using invasive or noninvasive techniques.^378–380^

##### 6.2.2.1. Recommendation

CFR and microcirculatory resistance can be measured in the catheterization laboratory by combining intracoronary pressure with thermodilution-based data to calculate the index of microcirculatory resistance (IMR). For clinical decision-making, IMR values > 25 units or CFR < 2.0 are indicative of abnormal microvascular function (Table 24).^361^ Both CFR and IMR are typically assessed during the administration of intravenous vasodilators, such as adenosine. The possibility of microvascular angina should be considered in patients with well-defined anginal symptoms, abnormal noninvasive functional testing, and angiographically normal coronary arteries or only mild, functionally nonsignificant stenoses identified on ICA or CCTA.

**Table 24:** Recommendations for the Use of Invasive Functional Assessment in Chronic Coronary Disease

Recommendation	Recommendation grade	Level of evidence
In patients with chronic coronary syndrome presenting with angina or angina-equivalent symptoms, without prior ischemia assessment and with angiographically intermediate stenoses, invasive functional assessment is recommended before proceeding with percutaneous coronary intervention.	**I**	**A**
Measurement of microcirculatory resistance should be considered in patients with persistent symptoms but angiographically normal coronary arteries or moderate stenoses with preserved fractional flow reserve and nonhyperemic indices.	**IIa**	**C**

### 6.3. Intravascular Imaging Techniques

#### 6.3.1. Use of Intravascular Imaging in the Diagnosis and Assessment of Coronary Stenoses

The main intravascular imaging modalities currently available are IVUS and OCT. IVUS is an imaging modality based on the processing of sound wave echoes reflected from vascular tissues, with an axial resolution of approximately 150 *μ*m.^381^ In contrast, OCT uses optical beams in wavelengths near the infrared spectrum as its energy source, providing an axial resolution of 10-15 *μ*m.^382^

Potential clinical applications of intravascular imaging in the diagnostic evaluation of patients considered for CABG include: assessing the severity of intermediate-grade lesions, evaluating lesion morphology in angiographically ambiguous cases, and characterizing plaque composition.

##### 6.3.1.1. Recommendation

With regard to the assessment of intermediate-grade stenosis (typically defined as 50%-69%), several studies have investigated the optimal cutoff value for minimum lumen area to identify hemodynamically significant lesions (Table 25).

**Table 25:** Recommendations for the Use of Intravascular Imaging in the Diagnosis and Assessment of Coronary Stenoses

Recommendation	Recommendation grade	Level of evidence
IVUS should be considered for the assessment of stenosis severity in the left main coronary artery.	**IIa**	**B**
The use of intravascular imaging (IVUS or OCT) may be considered for the evaluation of intermediate coronary stenoses.	**IIb**	**B**

IVUS: intravascular ultrasound; OCT: optical coherence tomography.

A prospective registry demonstrated a moderate overall correlation between minimum lumen area (MLA) and FFR values, with cutoff points for detecting hemodynamically significant stenosis (< 2.4, < 2.7, and < 3.6 mm^2^) depending on vessel size (reference vessel diameters < 3.0, 3.0-3.5, and > 3.5 mm, respectively).^383^ In a randomized trial, 1,682 patients were evaluated for PCI of moderate lesions (40%-70%) guided by either FFR or IVUS. In this study, both methods were used to determine the need for PCI and to assess procedural success. In the IVUS group, PCI was indicated for an MLA ≤ 3 mm^2^, or for MLA between 3 and 4 mm^2^ when plaque burden exceeded 70%. IVUS was noninferior to FFR for the primary composite endpoint of death, MI, or CABG at 24 months after randomization.^384^

Another randomized study compared OCT-guided versus FFR-guided PCI in patients with intermediate coronary lesions. In the OCT group, intervention was performed based on criteria of ≥ 75% area stenosis, or 50-75% stenosis with MLA < 2.5 mm^2^ or plaque rupture. This group showed a lower rate of composite events at 13 months (MACE and significant angina) compared to the FFR-guided group (8.0% vs. 14.8%; p = 0.048).^385^ However, this was a single-center study with a relatively small sample size (n = 350 patients). Overall, FFR-based hemodynamic assessment should be preferred for evaluating intermediate coronary stenoses.

The presence of intermediate-grade left main coronary artery stenosis is not uncommon, and angiographic assessment can be challenging. The use of IVUS to evaluate atherosclerotic disease in intermediate left main coronary artery stenosis among patients considered for CABG or PCI is supported by data from several observational studies.

In a prospective multicenter study, revascularization was indicated for patients with MLA < 6 mm^2^, while those with MLA ≥6 mm^2^ were managed conservatively.^386^ After a 2-year follow-up, cardiac death-free survival was similar between groups (97.7% in the conservatively managed group vs. 94.5% in the revascularized group). Another study suggested that deferring intervention in 131 patients with an MLA > 7.5 mm^2^ was associated with favorable clinical outcomes.^387^

In Asian populations, who generally have smaller cardiac dimensions, studies have suggested that an IVUS-derived MLA of 4.5-4.8 mm^2^ may be a more appropriate cutoff for intervention.^388^

#### 6.3.2. Use of Intravascular Imaging to Guide Percutaneous Coronary Intervention

Intravascular imaging modalities enable detailed assessment of plaque characteristics and precise vessel sizing during PCI. This allows for a more tailored strategy, optimal stent placement, more appropriate lesion preparation, better stent expansion, prevention of significant malapposition, and identification of relevant edge dissections.

##### 6.3.2.1. Recommendation

The use of IVUS or OCT to guide stent implantation has been associated with improved clinical outcomes in registries, randomized clinical trials, and meta-analyses.^144,389–396^ The benefit of image-guided PCI is attributed to reductions in MACE, cardiac death, stent thrombosis, target lesion revascularization, and target vessel revascularization (Table 26).

**Table 26:** Recommendations for the Use of Intravascular Imaging in the Planning and Guidance of Percutaneous Coronary Intervention

Recommendation	Recommendation grade	Level of evidence
Intravascular imaging (IVUS or OCT) should be used to guide complex PCIs*	**I**	**A**
Intravascular imaging (IVUS or OCT) may be considered to guide low-complexity PCIs.	**IIb**	**B**

IVUS: intravascular ultrasound; OCT: optical coherence tomography; PCI: percutaneous coronary intervention.

* Clinical judgment by the operator is essential when determining the need for urgent PCI – where intravascular imaging may delay case resolution – as well as evaluating the feasibility of advancing the imaging catheter across the coronary lesion (which may be affected by calcification, tortuosity, etc.) and the operator's proficiency in performing the procedure appropriately.

As of the writing of this guideline, a total of 20 randomized trials, 34 meta-analyses, and 85 observational registries have compared the clinical outcomes of image-guided versus angiography-guided PCI. Despite this evidence, the adoption of intravascular imaging to guide PCI remains low in our setting. Possible contributing factors include limited operator training and familiarity, the perception of increased procedure time, additional procedural costs, and, depending on the health care system, lack of reimbursement coverage.^388^

Complex PCIs – such as those involving the left main coronary artery, true bifurcations with a large myocardial area at risk, failed previously implanted stents, and long lesions – may derive greater benefit from intravascular imaging.^450^ However, there is no clear evidence supporting the universal use of intravascular imaging to guide all coronary interventions.^149,394^ Clinical judgment remains essential, particularly in urgent PCI settings (where intravascular imaging could delay case resolution), when considering whether the imaging catheter can cross the lesion (affected by factors such as calcification or vessel tortuosity), and when determining whether the operator is technically prepared to perform the procedure effectively.

### 6.4. Percutaneous Coronary Intervention as a Strategy for Prognostic Benefit

The body of evidence from randomized trials suggests that PCI may reduce the risk of death or nonfatal MI in specific subgroups of patients with chronic CAD, although this benefit does not apply universally to all patients with CAD.

A 10-year follow-up of the Brazilian Medicine, Angioplasty, or Surgery Study (MASS), which randomized multivessel disease patients to CABG, PCI, or medical therapy alone, showed that conservative management was associated with higher cardiac mortality (p = 0.02) and increased incidence of MI (p = 0.01) compared to surgical revascularization.^398^

In the multicenter, randomized ISCHEMIA trial, which compared invasive (either surgical or percutaneous) versus conservative treatment strategies, patients with greater anatomical severity experienced a lower incidence of death or MI at 4 years – but not lower all-cause mortality – when treated invasively (absolute difference –6.3%; 95% CI –12.4% to –0.2%).^399^ Additionally, in the same study, patients undergoing invasive intervention had a lower incidence of spontaneous MI (adjusted HR 0.53; 95% CI 0.41-0.69; p < 0.0001), and the risk of death following spontaneous infarction was more than doubled (p < 0.001).^400^

A meta-analysis involving 19,806 patients from 25 randomized trials comparing elective revascularization versus medical therapy alone – many of which were conducted over 25 years ago, prior to the widespread availability of current pharmacologic treatments – found a significant reduction in CV mortality in patients treated invasively (RR 0.79; 95% CI 0.67-0.93; p < 0.01) after a mean follow-up of 5.7 years.^401^ However, studies conducted over the last 10-15 years have not consistently reproduced this result, except in the case of CABG. Notably, the MASS II trial showed higher event rates with PCI compared to medical therapy, and in the ISCHEMIA trial, 28% of patients underwent surgery – likely representing the most severe cases with more extensive disease who may benefit most from the strategy.

In patients with significant LMCAD, modern studies comparing invasive versus conservative management remain limited. Contemporary trials have primarily focused on comparing PCI and surgery, without including a conservative (OMT) arm. A recent meta-analysis including 4,394 patients from four studies comparing drug-eluting stent PCI with surgery found that 5-year mortality rates were similar between treatment strategies, both in acute (HR 0.93; 95% CI: 0.68-1.27) and chronic presentations (HR 1.19; 95% CI 0.95-1.50).^402^ In the same analysis, PCI-treated patients had a lower risk of stroke but a higher rate of spontaneous MI.

The randomized FAME 2 trial demonstrated that invasive assessment of coronary physiology using FFR can identify patients who benefit from percutaneous treatment. Among those with angiographically documented stenoses ≥50% and FFR ≤0.80, PCI was associated with a lower risk of urgent CABG or spontaneous MI over a 5-year follow-up period.^145^ More recently, the use of computational flow dynamics derived from ICA to guide percutaneous treatment has also been associated with significant improvement in post-procedural prognosis.^403,404^

At present, PCI in patients with CCS is primarily indicated as a revascularization strategy for vascular territories affected by significant luminal obstruction with hemodynamic repercussions. The reduction in MACE is confined to specific patient subgroups, and PCI should not be routinely recommended for this purpose in the general population.

#### 6.4.1. Percutaneous Coronary Intervention as a Strategy for Symptom Relief

In CCS, two core pillars of treatment are well established: therapies aimed at reducing MACE and therapies focused on symptom relief. When indicated in selected patients, revascularization can reduce the risk of MACE – including death, MI, and the need for further CABG – particularly in those with multivessel disease, LV dysfunction, or LMCAD. Similarly, in patients who continue to experience limiting symptoms and impaired QoL despite tolerated medical therapy, revascularization should be considered to improve angina control and improve QoL. The extent and severity of stress-induced ischemia, as assessed by imaging modalities, may be useful in guiding clinical decision-making.

However, in clinical practice, the relationship between angina and myocardial ischemia may be difficult to establish in patients with stable angina. In the ORBITA-2 trial (Objective Randomized Blinded Investigation With Optimal Medical Therapy of Angioplasty in Stable Angina), although PCI reduced symptoms, approximately 60% of patients continued to report symptoms despite the resolution of myocardial ischemia.^404^ Therefore, careful evaluation is necessary to determine whether the patient's clinical complaint truly reflects anginal pain.

A secondary analysis of the ORBITA-2 trial found no correlation between symptom severity and the extent of myocardial ischemia assessed by stress echocardiography, invasive FFR, or angiographic stenosis severity.^405^ In that study, the strongest predictor of angina relief following PCI was the presence of typical angina in patients receiving OMT.

Similarly, in the ISCHEMIA trial, younger patients and those with more severe angina were more likely to benefit from PCI in terms of angina relief.^406^

The ORBITA-STAR trial (Symptomatic Trial of Angina Assessment Prior to Revascularization) trial^407^ evaluated whether ischemia induced by balloon inflation during PCI in coronary stenoses reproduced symptoms similar to patients’ baseline angina. In that trial, when balloon-induced coronary obstruction triggered equivalent anginal symptoms, there was a higher likelihood of symptom resolution at 12 weeks after PCI compared to patients whose symptoms were not reproduced by balloon inflation. This finding underscores the fundamental importance of achieving an accurate correlation between symptomatology and documented anatomical lesion and/or ischemia in order to propose an effective treatment strategy (Table 27).

**Table 27:** Indications for percutaneous treatment for symptom control and prognostic benefit

Recommendation	Recommendation grade	Level of evidence
Symptom control in patients with significant coronary stenoses and limiting typical angina despite optimal medical therapy.	**I**	**A**
Multivessel coronary artery disease with suitable anatomy for percutaneous treatment (e.g., low or moderate anatomical complexity).	**IIa**	**A**
Coronary artery disease involving significant left main coronary stenosis with suitable anatomy for percutaneous treatment (e.g., low or moderate anatomical complexity).	**IIa**	**B**
Luminal stenosis causing significant impairment of coronary flow as assessed by invasive physiological testing.	**I**	**B**
Nonsignificant luminal stenosis with features of plaque vulnerability identified by intravascular imaging.	**IIb**	**B**

### 6.5. Bleeding Risk Scores in Clinical Decision-Making

Bleeding has a significant impact on the prognosis of patients with CAD, with a clear association between bleeding events and increased mortality and morbidity.^405–411^

Major bleeding – particularly when requiring blood transfusion or prolonged hospitalization – can lead to hemodynamic and cardiac decompensation and may also necessitate interruption or modification of antithrombotic therapies that are essential for preventing ischemic events. Additionally, the occurrence of bleeding may indicate underlying frailty, reflecting a complex interaction among clinical factors such as advanced age, comorbidities, and the need for invasive treatments.^408,412^

In summary, proper management of bleeding risk – through careful assessment and implementation of effective preventive strategies – is crucial for improving clinical outcomes in patients with CAD.

The Academic Research Consortium for High Bleeding Risk (ARC-HBR) criteria were developed to identify patients under high risk of bleeding, providing a consensus-based, practical definition for use in clinical trials and everyday practice.^408^ These criteria include a set of clinical and demographic factors that increase the likelihood of bleeding (Table 28). A major ARC-HBR criterion is defined as any individual factor that alone confers a ≥ 4% risk of major bleeding at 1 year or a ≥ 1% risk of intracranial hemorrhage (ICH) at 1 year. A minor ARC-HBR criterion is defined as any factor that alone confers an increased risk of major bleeding ≤ 4%. However, applying the ARC-HBR criteria in routine clinical practice may be challenging due to their complexity, which can limit individualized risk stratification, especially when multiple clinical factors coexist.

**Table 28:** Major and minor criteria for high bleeding risk at the time of percutaneous coronary intervention

Major criteria	Minor criteria
	Age ≥ 75 years
Long-term use of oral anticoagulants	Prolonged use of NSAIDs or corticosteroids
Chronic kidney disease (creatinine clearance < 30 mL/min)	Chronic kidney disease (creatinine clearance 30-59 mL/min)
Anemia (hemoglobin < 11 g/dL)	Anemia (hemoglobin 11-12.9 g/dL in men or 11-11.9 g/dL in women)
Bleeding requiring hospitalization or transfusion within the past 6 months, or at any time if recurrent	Bleeding requiring hospitalization or transfusion within the past 12 months
Platelet count < 100,000/*μ*L	
Bleeding diathesis	
Advanced liver disease (cirrhosis or portal hypertension)	
Active malignancy (excluding non-melanoma skin cancer) within the past 12 months	
Prior spontaneous intracerebral hemorrhage (any time) Traumatic intracerebral hemorrhage within the past 12 months Presence of cAVM Moderate or severe ischemic stroke within the past 6 months	Any prior ischemic stroke not meeting a major criterion
Urgent major surgery required Major surgery or trauma within the past 30 days	

NSAIDs: nonsteroidal anti-inflammatory drugs; cAVM: cerebral arteriovenous malformation.

The PRECISE-DAPT, ARC-HBR Trade-Off, and DAPT scores^412–414^ have been developed to guide and inform decision-making regarding the duration of DAPT in patients undergoing stent implantation.

The utility of the PRECISE-DAPT score was retrospectively evaluated in 10,081 patients randomized to different DAPT durations to assess the impact of prolonged (12-24 months) versus short (3-6 months) treatment based on baseline bleeding risk.

Among patients with high bleeding risk (HBR), defined by a PRECISE-DAPT score ≥25, prolonged DAPT did not provide ischemic benefit and increased the risk of bleeding events. In contrast, in patients without HBR (score <25), prolonged DAPT was not associated with increased bleeding and significantly reduced the composite ischemic endpoint of MI, definite stent thrombosis, stroke, and target lesion revascularization. An external validation of the PRECISE-DAPT score in 4,424 patients with ACS undergoing PCI and treated with prasugrel or ticagrelor showed modest predictive value for major bleeding over a median follow-up of 14 months (c-statistic = 0.65). The PRECISE-DAPT score is applied at the time of hospital discharge.

The ARC-HBR trade-off model was developed by the Academic Research Consortium for High Bleeding Risk to evaluate the competing risks of bleeding and thrombotic events in patients at high bleeding risk following coronary stent implantation. The model incorporates individual patient characteristics to estimate the likelihood of nonprocedure-related major bleeding and thrombotic events (MI and/or stent thrombosis) after PCI. While the model demonstrated good discriminatory capacity for predicting thrombotic events at 1 year across various populations, it tended to overestimate major bleeding risk, and its performance for bleeding prediction was less satisfactory in some patient groups.^415^

The DAPT(7) score was developed to estimate bleeding risk and guide the duration of DAPT in patients who have undergone PCI. Patients who remain event-free during the first year of DAPT may benefit from extended therapy if they have a high DAPT score (≥ 2), as it reduces the combined risk of ischemic and bleeding events. In contrast, in patients with a low score (< 2), extending DAPT increases bleeding risk without reducing ischemic events. A limitation of the DAPT score is that it was designed to predict risk for a 1-year DAPT duration, whereas current recommendations support shorter DAPT durations in patients at high bleeding risk.

#### 6.5.1. Recommendation

Antiplatelet agents play a critical role following PCI. The selection of the antiplatelet strategy and its duration should be guided by the patient's bleeding risk. Bleeding risk scores are recommended to support individualized decision-making, while acknowledging their limitations – particularly in assessing frailty or fall risk, especially among older adults (Table 29).

**Table 29:** Recommendation for the use of bleeding risk scores in clinical decision-making

Recommendation	Recommendation grade	Level of evidence
Use ARC-HBR criteria to identify patients at high bleeding risk who may benefit from shortened DAPT	**IIa**	**B**
Use PRECISE-DAPT ≥25 to identify patients at high bleeding risk who may benefit from shortened DAPT	**IIa**	**B**
Use the ARC-HBR trade-off model to predict the risk of MI, stent thrombosis, and major bleeding	**IIb**	**C**
Use of the DAPT score to determine DAPT duration is not recommended	**III**	**C**

ARC-HBR: Academic Research Consortium for High Bleeding Risk; DAPT: dual antiplatelet therapy; PRECISE-DAPT: Predicting Bleeding Complications in Patients Undergoing Stent Implantation and Subsequent Dual Antiplatelet Therapy.

Factors associated with high bleeding risk can be identified using the Academic Research Consortium for High Bleeding Risk (ARC-HBR) criteria or a PRECISE-DAPT score ≥ 25. Both tools have been validated in randomized trials for guiding decisions on shortened (1-month) versus standard (6-month) DAPT regimens.^8^

The DAPT score was developed to support a standard 1-year DAPT strategy and does not account for the newer generations of drug-eluting stents. The ARC-HBR trade-off model may assist in determining DAPT duration, but it is supported by less robust clinical evidence.

### 6.6. Preparation in Specific Clinical Situations (Patients with Renal Dysfunction or Contrast Allergy)

#### 6.6.1. Renal Dysfunction

ICA and PCI using contrast media (CM) are widely employed in clinical practice for the diagnosis and treatment of CAD. Despite advancements in chemical composition, contrast agents still pose potential risks and may cause contrast-induced nephropathy (CIN).^416^

The most accepted mechanisms for the development of CIN include reduced renal blood flow – resulting in medullary ischemia – free radical production, and direct tubular cytotoxicity.^417^ CIN is defined as either an absolute increase in serum creatinine > 0.5 mg/dL (44 *μ*mol/L) or a relative increase > 25% from baseline, occurring within 48 to 72 hours following CM exposure.^418^

While the incidence of CIN in the general population is < 3%, it may reach up to 50% in patients with multiple risk factors such as advanced kidney disease, diabetes, hypertension, and congestive HF.^419^

CIN is the third most common cause of hospital-acquired acute kidney injury^420^ and is associated with an increased short- and long-term risk of adverse outcomes, including renal replacement therapy, acute MI, stroke, and mortality.

#### 6.6.2. Renal Protection Measures

As CIN is largely predictable in most cases, preventive strategies remain the only effective approach for these patients, aiming to minimize renal injury and reduce associated adverse outcomes.

#### 6.6.3. General Measures

Clinical history and renal function assessment can be used to estimate the risk of developing CIN. Serum creatinine is currently recognized as an insensitive marker of renal function, as patients with moderate impairment may still exhibit normal serum levels. Therefore, eGFR should be calculated, and several formulas have been developed for routine clinical use.^421^ An eGFR < 60 mL/min should be considered a high-risk condition for the development of CIN.^422^

When the ratio between the total volume of contrast administered (in mL) and the GFR (in mL/min) exceeds 3.7, the risk of CIN increases significantly.^422^

Data from multiple studies have supported the development of a predictive risk score for CIN in patients undergoing PCI (Tables 30 and 31).

**Table 30:** Risk score for the development of CIN

Risk factors	Score (points)
Hypotension	5
Intra-aortic balloon pump	5
Congestive HF (NYHA class III/IV)	5
Age > 75 years	4
Anemia	3
Diabetes	3
Contrast volume	1 per 100 mL
Plasma creatinine > 1.5 mg/dL or	4
eGFR < 60 mL/min/1.73 m^2^	2 for 40-60 4 for 20-40 6 for < 20

eGFR: estimated glomerular filtration rate; HF: heart failure; NYHA: New York Heart Association.

**Table 31:** Risk of CIN and dialysis

Score (points)	Risk of CIN	Risk of dialysis
5	7.5%	0.04%
6 to 10	14%	0.12%
11 to 16	26.1%	1.09%
≥ 16	57.3%	12.60%

CIN: contrast-induced nephropathy.

#### 6.6.4. Fluid Administration

To date, the only strategies consistently shown to reduce the risk of CIN are hydration and minimizing the amount of contrast used. Other measures have shown neutral or harmful effects, or have been supported by heterogeneous and conflicting data.

Studies on hydration suggest that isotonic saline (0.9%) is preferable to hypotonic isotonic saline (0.45%), and that intravenous administration is superior to oral intake. Hydration over several hours – both before and after contrast exposure – is recommended rather than bolus infusion. Additionally, saline alone is preferred over saline combined with mannitol or furosemide.^423^

Based on these findings, a suggested hydration regimen for elective procedures includes isotonic saline 0.9% at 1.0-1.5 mL/kg/h for 3-12 hours before the procedure, continued for 6-24 hours afterward.

The use of sodium bicarbonate to alkalinize renal tubular fluid and reduce the formation of reactive oxygen species may theoretically mitigate tubular damage. However, clinical evidence on its effectiveness remains inconsistent.^424^

#### 6.6.5. Pharmacological Prevention

Several pharmacological agents have been tested with the aim of reducing the risk of CIN in patients receiving CM.

Early studies evaluating the use of N-acetyl-L-cysteine yielded conflicting results. A recent systematic review demonstrated that its use does not prevent the development of CIN.^425^ There is no support for the use of N-acetyl-L-cysteine in CIN prevention.

Other agents – such as CCBs, prostaglandins, adenosine antagonists, atrial natriuretic peptide, and ACEi – have also been tested, but have shown no benefit or have produced inconsistent results.^426^

#### 6.6.6. Contrast Media

The recommended iodinated contrast agents are low-osmolar and iso-osmolar contrast media, which have lower osmolarity and osmolarity similar to that of blood plasma, respectively. Both are associated with a lower incidence of adverse effects, including bradycardia, systemic hypotension, and sensations of warmth, nausea, vomiting, allergic reactions, and nephrotoxicity.

Recently, the ultra-low contrast volume technique, defined as a procedure in which the ratio of contrast volume administered to eGFR is less than 1, has been adopted in selected clinical scenarios – such as CKD, previous acute kidney injury, kidney transplant, severe LV dysfunction, and complex PCI. This strategy aims not only to reduce the risk of CIN, but also to minimize hemodynamic instability.^427^

#### 6.6.7. Allergy to Contrast Media

The incidence of allergic reactions to CM is ≤1% of procedures, with severe reactions occurring in approximately 0.04%. These reactions are not true allergic responses, as they are not IgE-mediated. They are more accurately described as anaphylactoid reactions, involving mast cell and circulating basophil degranulation through direct complement activation. Further details can be found in specific guidelines.^428^

Patients with a history of prior anaphylactoid reactions to CM are considered under high risk for recurrence. Repeated anaphylactoid reactions have been reported in 16% to 44% of this population.

It is recommended that prednisone 20 mg combined with a second-generation antihistamine (fexofenadine hydrochloride 180 mg, orally) be administered for 3 days prior to elective procedures. Patients receiving this regimen exhibit near-zero rates of severe reactions.

In emergency procedures, the administration of hydrocortisone 500 mg intravenously (IV), fexofenadine hydrochloride 180 mg orally, and diphenhydramine hydrochloride 50 mg IV or intramuscularly (IM) (an H1 receptor blocker) is recommended (Tables 32 and 33).^429^

**Table 32:** Prophylaxis of allergic reactions: recommendations

Recommendation	Recommendation grade	Level of evidence
Use of iso-osmolar or low-osmolar contrast media.	**I**	**A**
Prednisone 50 mg at 13 h, 7 h, and 1 h before the procedure.	**I**	**B**
Diphenhydramine hydrochloride 50 mg IV or IM 1 h before the procedure.	**I**	**B**
Fexofenadine hydrochloride 180 mg: 1 tablet/day for 3 days before the procedure.	**I**	**C**

IM: intramuscularly; IV: intravenously.

**Table 33:** Prophylaxis of allergic reactions for emergency procedures

Drug	Grau de recomendação	Nível de evidência
Hydrocortisone 500 mg IV	**I**	**B**
Diphenhydramine hydrochloride 50 mg IV or IM 1 h before the procedure	**I**	**B**
Fexofenadine hydrochloride 180 mg orally 1 h before the procedure	**I**	**C**

IM: intramuscularly; IV: intravenously.

#### 6.6.8. Conclusions

The development of CM has had a direct impact on reducing complications associated with angiographic procedures.

Knowledge of the prevention, diagnosis, and treatment of CIN and allergic reactions is essential for clinical cardiologists, interventionalists, and imaging professionals.

The implementation of the recommendations described herein is of fundamental importance for minimizing and controlling CM-related complications.

### 6.7. Surgical Treatment for Symptom Control and Risk Reduction

CAD carries considerable morbidity and mortality due to its association with MI, increasing the risk of death and HF. In addition, the local and systemic inflammatory process and endothelial dysfunction inherent to atherosclerosis contribute to the development of angina pectoris, thereby compromising patients’ QoL and exercise capacity. Therefore, therapies aimed at treating CAD should target both event reduction and symptom control.

The choice of CABG should consider several key factors, including the extent and anatomical complexity of CAD, expected outcomes of the selected therapy, and the risks of complications and mortality, among other adverse outcomes.

A wealth of historical and contemporary evidence confirms that CABG remains the most effective treatment for advanced atherosclerotic CAD, providing reduced risk of MI, increased survival, and improved angina and QoL in this patient population. This evidence includes randomized controlled trials, meta-analyses, and national and regional registries.

The primary goal of improving prognosis in CAD lies in implementing therapies that can protect against future ACS events. Recent evidence shows that ACS events do not primarily result from the occlusion of severely stenotic plaques identified on ICA. Instead, they are more often due to rupture or erosion of a coronary atherosclerotic plaque – typically with mild to moderate stenosis – located away from the stable plaque. The main mechanism of death in CAD is no longer attributed to the stable plaque (i.e., a fixed lesion with > 50% lumen stenosis) but rather to acute MI caused by the rupture of an unstable plaque with secondary intracoronary thrombosis. CABG effectively reduces events by treating both the current obstructive lesions and potential future thrombotic occlusions. It achieves this by grafting arterial or venous conduits distal to the diseased coronary bed, thereby protecting against acute ischemic events. In contrast, PCI targets only the stable lesion and leaves vulnerable plaques at risk.^430–435^ Several factors must be considered in selecting patients in whom CABG offers risk reduction for MI, increased survival, and angina relief (Table 34).

**Table 34:** Patient subgroups with indication for CABG

Anatomy – Extent of CAD
Multivessel CAD
SYNTAX score > 23
Involvement of the proximal left anterior descending artery
Left main coronary artery lesion
**Especially with associated comorbidities**
Diabetes
Left ventricular dysfunction
Non-ST-elevation acute myocardial infarction

CAD: coronary artery disease; CABG: coronary artery bypass grafting.

#### 6.7.1. Patients with Multivessel Coronary Artery Disease and SYNTAX Score > 23

Patients with advanced CAD involving multivessel disease and a SYNTAX score > 23 derive event reduction benefits, as demonstrated in several randomized controlled trials.

In the 5-year analysis of the SYNTAX trial – which included primarily patients with preserved LVEF and multivessel disease – CABG was associated with a significant reduction in CV and cardiac mortality, mainly due to lower infarction-related death, compared to PCI. In contrast, PCI was an independent predictor of cardiac death, with 40% higher mortality in patients with three-vessel disease (3VD) undergoing PCI than with CABG. The difference in infarction-related death was most notable in patients with diabetes, 3VD, or higher SYNTAX scores (> 23).^436^ At 10-year follow-up, the SYNTAX Extended Survival (SYNTAXES) study reported significantly lower all-cause mortality with CABG compared to PCI. A substantial survival benefit was observed among patients with 3VD, with the survival curves continuing to diverge over time in favor of CABG (Figure 17).^141^

The MASS II trial, the only study comparing CABG, PCI, and OMT in patients with multivessel CAD using a composite outcome of total mortality, Q-wave MI, or RA requiring revascularization, showed that at 5 years, CABG was superior to OMT for the primary outcome, achieving a 44% relative reduction. No statistical difference was observed between PCI and OMT for the composite endpoint.^437^

In the MASS II study, all patients received OMT from the start through the end of follow-up, including ASA, BBs, ACEi, CCBs, nitrates, and lipid-lowering agents, along with a low-fat diet. All medications were provided free of charge throughout the 10-year follow-up to ensure adherence to the protocol. Although the study was not powered to detect differences in individual components of the composite endpoint, there was a significantly lower incidence of nonfatal MI with CABG versus OMT at 10 years (20.7% vs. 10.3%, p = 0.010), but not at 5 years (p = 0.785). Cardiac mortality was significantly higher with OMT versus CABG (20.7% vs. 10.8%, p = 0.019), although this was not significant at 5 years (12.3% vs. 7.9%, p = 0.631). All-cause mortality was reduced with CABG compared to OMT (25.1% vs. 31.0%, p = 0.089), although this did not reach statistical significance; at 5 years, mortality was 12.8% vs. 16.2% (p = 0.824). Paired comparison analysis showed a significant 2.02- and 2.77-fold increased risk of cardiac death and subsequent MI with OMT vs. CABG, respectively, highlighting the progressively better long-term prognosis associated with surgical treatment.^398^

The findings of the MASS II trial reinforce those from SYNTAX, STICH, and FREEDOM trials, showing that the robust benefits of CABG continue to increase in the long-term follow-up beyond the initial 5-year evaluation.

More recently, the publication of the FAME 3 (Fractional Flow Reserve versus Angiography for Multivessel Evaluation) trial, which randomized patients with 3VD and predominantly preserved LV function (LVEF > 50% in over 80% of patients) to undergo either CABG or FFR-guided PCI using second-generation zotarolimus-eluting stents, further reinforced the benefits of CABG. In the 1-year analysis of the primary composite endpoint (death, MI, stroke, or repeat CABG), the more advanced PCI technology was not effective in reducing MACE and cerebrovascular events compared to CABG, highlighting the superiority of CABG in patients with 3VD. The incidence of spontaneous MI (3.3% vs. 2.3%), all-cause mortality (1.6% vs. 0.9%), and CV mortality (0.8% vs. 0.5%) was higher in the PCI group compared to the CABG group, respectively. The benefit of CABG was already evident at an early stage of follow-up – within just 1 year – unlike previous studies where favorable outcomes with CABG emerged only after more than 3 years of follow-up.^438^

The 3-year follow-up of the FAME 3 trial demonstrated a growing divergence between the outcomes of PCI and CABG in relation to the primary composite endpoint, which occurred in 18.6% of PCI patients and 12.5% of patients undergoing CABG (p = 0.002). The difference in MI incidence became statistically significant in favor of CABG (7.0% vs. 4.2%; p = 0.02). Similarly, the secondary composite outcome of death, MI, or stroke at 3 years occurred in 9.2% of patients undergoing CABG versus 12.0% of patients undergoing PCI, further expanding the gap observed at 1 year (5.2% vs. 7.3%, respectively). Although the publication emphasized these secondary outcome results, it did not have sufficient statistical power to support formal recommendations based on them.^439^

The planned 5-year analysis evaluating the primary composite endpoint (death, stroke, or MI) showed no significant difference between the PCI and CABG groups. However, when the components were analyzed separately, rates of MI and repeat revascularization remained higher in the PCI group.^440^

#### 6.7.2. Patients with Advanced Coronary Artery Disease and Diabetes

Multiple lines of evidence have shown CABG provides superior outcomes compared to alternative therapies, reducing mortality and MI rates in patients with advanced CAD and diabetes. The BARI 2D (Bypass Angioplasty Revascularization Investigation 2 Diabetes) trial included patients with type 2 diabetes who were assigned to either prompt coronary revascularization (REV) plus intensive medical therapy or intensive medical therapy alone (MED). While no significant difference in mortality was observed between treatment groups overall, in the CABG stratum, the rate of MACE was significantly lower in the REV group (22.4%) than in the MED group (30.5%, p = 0.01). In contrast, no significant differences in primary outcomes were found in the PCI stratum. Among the 381 patients selected for CABG in the highest angiographic risk tertile, the 5-year risk of death/MI/stroke was 36.8% for the MED group versus 24.8% for the REV group (p = 0.005); this treatment effect was amplified in patients with both high angiographic and high Framingham risk scores (47.3% MED vs. 27.1% REV, p = 0.010).^441^

The FREEDOM trial randomized patients with diabetes and multivessel CAD to PCI with drug-eluting stents or CABG, with a median follow-up of 3.8 years. The primary outcome – a composite of all-cause death, nonfatal MI, or stroke – favored CABG, which was associated with significantly lower rates of death and MI, although stroke rates were higher. The incidence of spontaneous MI due to new lesions was significantly greater in the PCI group compared to the CABG group.^442^ The FREEDOM Follow-On Study, with a median follow-up of 7.5 years, showed a significantly higher all-cause mortality rate in the PCI group than in the CABG group (24.3% vs. 18.3%; p = 0.01) (Figure 17).^443^

An analysis of three U.S. government-funded trials (BARI 2D,^441^ COURAGE,^85^ and FREEDOM^444^) aimed to determine the long-term outcomes of OMT, with or without PCI or CABG, in patients with CAD and type 2 diabetes. Over a mean follow-up of 4.5 years, CABG plus OMT was superior to PCI plus OMT for the primary composite endpoint (death, MI, or stroke) as well as for all-cause death and MI. CABG plus OMT was also superior to OMT alone for preventing the primary endpoint and MI, and it outperformed PCI plus OMT in patients with 3VD and preserved LVEF. No significant differences were observed when comparing OMT alone versus PCI plus OMT.^444^

A meta-analysis including data from 28,846 patients confirmed these findings, showing consistently higher 5-year mortality risk among patients treated with PCI compared to those treated with CABG. Among individuals with diabetes, revascularization via CABG was associated with significantly lower long-term mortality, MI, MACE and cerebrovascular events, and repeat revascularizations.^445^

#### 6.7.3. Patients with Left Ventricular Dysfunction

The efficacy of treatment for ischemic cardiomyopathy (ICM) in patients with LV dysfunction was investigated in the STICH trial, which compared the addition of surgical myocardial revascularization to OMT versus OMT alone. After 5 years of follow-up, significant benefits were observed in the surgical group in terms of secondary outcomes, including reduced CV mortality and the composite endpoint of all-cause mortality or hospitalization. However, in this analysis, surgical therapy did not produce a statistically significant reduction in the primary endpoint of all-cause mortality.^446^

In the extended 10-year follow-up of the STICH trial, patients who underwent CABG (in addition to OMT) had lower rates of all-cause mortality, CV mortality, and the composite endpoint of all-cause mortality or hospitalization for CV causes compared to those who received OMT alone. CABG significantly reduced sudden deaths and deaths due to MI, with a nominally significant effect on fatal pump failure events. The favorable impact of CABG on sudden death may also be attributed to a beneficial effect on the progression of HF, as indicated by a reduction in fatal pump failure events. Figure 1 displays the 10-year follow-up survival curves from the STICH trial, which increasingly diverged over time in favor of patients who underwent CABG.^136^

HF often develops following MI and is associated with infarct size, the affected territory, the development of mitral regurgitation, and the presence of certain tachyarrhythmias. The heightened systemic inflammatory state in HF promotes destabilization of atheromatous plaques, increasing the risk of ACS events. In patients with HF and reduced LVEF, even small infarcts confer a markedly increased risk of mortality.

#### 6.7.4. Patients with Left Main Coronary Artery Disease

The SYNTAX trial was pivotal in evaluating the efficacy of CABG and PCI in patients with LMCAD. The evidence from the study demonstrated definitive and statistically significant benefits favoring surgical treatment in high-risk patients (SYNTAX score > 32), supporting a Class I recommendation for CABG and a Class III recommendation for PCI in the European guidelines on myocardial revascularization. However, in patients with a SYNTAX score < 32 (low or intermediate risk), no statistical difference was observed, though the study lacked the statistical power to definitively resolve this question.^447^ As a result, additional trials were needed, leading to the design of the EXCEL and NOBLE trials – both randomized noninferiority studies comparing CABG and PCI in patients with LMCAD and SYNTAX scores < 32.

The NOBLE trial enrolled 1,200 patients with LMCAD. The primary endpoint was a composite of MACE and cerebrovascular events, including all-cause death, MI, repeat revascularization, and stroke. The 5-year follow-up showed MACE and cerebrovascular events rates of 28% for PCI and 19% for CABG, exceeding the noninferiority margin, indicating that CABG was significantly superior to PCI in this population. All-cause mortality was similar between groups: 9% with PCI versus 9% with CABG.^448^

The EXCEL trial demonstrated a statistically significant difference in all-cause mortality at 5 years favoring CABG (9.9% with CABG vs. 13.0% with PCI), with the survival curves continuing to diverge over time. All-cause mortality at 5 years was 38% higher in the PCI group than in the CABG group. However, the trial concluded that there was no significant difference between PCI and CABG in the composite endpoint of death, stroke, or MI at 5 years.^449,450^

A meta-analysis combining data from four trials comparing PCI and CABG in patients with LMCAD reported similar 5-year mortality rates between the two strategies (11.2% for PCI vs. 10.2% for CABG). However, spontaneous MI was more frequent with PCI (6.2% vs. 2.6%), while the risk of stroke was similar between groups (2.7% vs. 3.1%).^451^

From a pathophysiological perspective, LMCAD should not be viewed as a distinct entity within the spectrum of CAD, since the primary mechanism of death in CAD is no longer attributed solely to stable plaques (e.g., left main coronary artery lesion), but rather to MI caused by unstable plaques throughout the coronary circulation. CABG, by grafting distal coronary segments, offers protection against such events and improves 5-year survival, as demonstrated in the EXCEL trial.^452^

In 2022, the ESC and the European Association for Cardio-Thoracic Surgery (EACTS) jointly established a task force to review the recommendations from the 2018 ESC/EACTS Guidelines on Myocardial Revascularization in patients with LMCAD and a low to intermediate SYNTAX score (0-32). In stable patients with LMCAD and an indication for revascularization, with coronary anatomy suitable for both procedures and a low predicted surgical mortality, the task force concluded that both treatment options are clinically reasonable, depending on patient preference, local expertise, and operator volume. The proposed recommendation for revascularization with CABG was Class I, Level of Evidence A. The recommendation for PCI was Class IIa, Level of Evidence A.^453^

#### 6.7.5. Relief of Anginal Symptoms and Quality of Life

Improving patients’ QoL by relieving anginal symptoms is another important goal of myocardial revascularization. For specific subgroups – such as octogenarians and nonagenarians – improving QoL may be more relevant than the modest increase in life expectancy (Table 35).

**Table 35:** Recommendations for coronary artery bypass grafting to reduce the risk of myocardial infarction and increase survival compared to percutaneous coronary intervention and/or optimal medical therapy

Recommendation	Recommendation grade	Level of evidence
Multivessel CAD – involving the LAD, SYNTAX score > 23 – preserved LVEF, with proximal LAD involvement	**I**	**A**
Multivessel CAD with LV dysfunction	**I**	**A**
LMCA lesion	**I**	**A**

CAD: coronary artery disease; LAD: left anterior descending artery; LMCA: left main coronary artery; LV: left ventricle; LVEF: LV ejection fraction.

In the early post-revascularization period (up to 1 month), both patients undergoing CABG and those undergoing PCI report improvement in angina frequency. However, at 6 months and in subsequent years, angina relief is significantly greater following CABG compared with PCI. Likewise, the use of antianginal medications remains significantly higher in patients treated with PCI, even with the use of new-generation drug-eluting stents in recent years.

In the first month after the procedure, PCI patients tend to recover more quickly than those who undergo surgery, reporting fewer physical limitations, less bodily pain, and greater QoL and satisfaction with treatment. These differences disappear by 6 months, and in the years that follow, patients treated with CABG report fewer physical problems and limitations compared with those who underwent PCI. Approximately 5 years after revascularization, significant benefits continue to favor CABG in terms of physical, emotional, and mental health.

In the SYNTAX trial, the QoL substudy showed that at 5-year follow-up, CABG was superior to PCI across several domains, including angina frequency, physical limitation due to angina, and physical and emotional health scores. Subgroup analysis revealed a significant interaction between angiographic complexity (as assessed by the SYNTAX score) and angina relief; notably, angina relief at 5 years was greater with CABG among patients with high SYNTAX scores. This finding reinforces the recommendation that CABG should be strongly preferred in such patients.

Among patients with diabetes, the FREEDOM trial included a QoL analysis using the Seattle Angina Questionnaire, collected at baseline, 1 and 6 months post-revascularization, and annually thereafter. Compared with baseline, both groups showed significant improvement in angina frequency during follow-up. However, CABG patients experienced greater angina relief during the first and second years. After year 2, both revascularization strategies yielded similar results.

In the NOBLE trial, the authors demonstrated that patients treated with PCI experienced more angina symptoms at 5 years compared with those treated with CABG, with differences in outcomes becoming most apparent after the first year of follow-up.

Figure 18 shows the long-term evolution of patients regarding the outcome of all-cause mortality, comparing CABG, PCI, and OMT. The continued divergence of survival curves over time reinforces the protective effect of CABG against MI in CAD.

**Figure 18:** Long-term reduction in all-cause mortality. LMCAD: left main coronary artery disease.

Figure 19 shows pathways that may serve as an initial guide for decision-making regarding myocardial revascularization.

**Figure 19:** To reduce cardiovascular mortality, especially in individuals with diabetes, this is the initial guidance for recommending myocardial revascularization based on coronary anatomy and key clinical and imaging parameters for assessment of left ventricular ejection fraction. CAD: coronary artery disease; CABG: coronary artery bypass grafting; LAD: left anterior descending artery; LMCAD: left main coronary artery disease; LVEF: left ventricular ejection fraction; PCI: percutaneous coronary intervention.

## 7. Vaccination Against Pneumonia, Influenza, and COVID-19

Individuals with CVDs are at increased risk of complications from some infectious diseases, so vaccination is recommended for this population. The following recommendations are based on the 2024 Brazilian Ministry of Health immunization schedule and the 2023/2024 Brazilian Society of Immunizations (SBIm) guidelines (Table 36.1).

**Table 36.1:** Vaccination schedule for patients with cardiovascular disease

Vaccine	Schedule and recommendation	Location of vaccination
Vaccine	Adults > 60 years: high-dose quadrivalent vaccine (HD4V) preferred. Quadrivalent vaccine (4V) preferred over trivalent vaccine (3V).	3V available at UBS; 4V available at private vaccination clinics
Influenza	Recommended from 2 months of age. For adults >60 years: one booster dose, preferably with PCV20.	Available at CRIE (PCV13 and PCV15) and private clinics (PCV20)
Pneumococcal conjugate vaccine (PCV13, PCV15, and PCV20)	1 dose with a booster dose after 5 years	Available at CRIE
23-valent pneumococcal polysaccharide vaccine (PPSV23)	Primary series: 2 doses with 4-week interval + third dose after 8 weeks. Annual booster.	Available at UBS

CRIE: *Centro de Referência para Imunobiológicos Especiais* (Reference Center for Special Immunobiologicals); UBS: *Unidade Básica de Saúde* (Primary Health Care Unit). Source: Vaccination schedules of the Brazilian Society of Immunizations for special populations – 2023/2024.^454^

### 7.1. Influenza Vaccine

All influenza vaccines currently used in Brazil are inactivated (containing killed virus), and therefore incapable of causing disease. Until 2014, only the trivalent vaccine was available in the country, containing one A/H1N1 strain, one A/H3N2 strain, and one B strain (from either the Yamagata or Victoria lineage). Since 2015, quadrivalent vaccines, which include a second B strain (covering both the Victoria and Yamagata lineages), have been licensed and are in use. Like the trivalent version, these are inactivated and do not contain adjuvants. In 2023, a new quadrivalent vaccine became available in private vaccination clinics.

The high-dose quadrivalent influenza vaccine (HD4V) contains four times the amount of antigen compared to standard-dose quadrivalent vaccines. It is licensed for individuals aged 60 years or older. The SBIm recommends that individuals in this age group – particularly those who are immunocompromised – receive the HD4V, as the protection offered by standard-dose vaccines in older adults is lower than that observed in younger populations. The development of high-antigen formulations has enhanced the immune response in older adults, especially against Influenza A (H3N2), which is more prevalent and severe in this population.^454^

### 7.2. Adverse Effects and Events

- Local reactions such as pain, redness, and induration occur in 15% to 20% of vaccine recipients. These reactions are usually mild and resolve within 48 hours.
- Systemic symptoms are also generally mild and transient. Fever, malaise, and myalgia affect approximately 1%-2% of recipients. These symptoms typically begin 6-12 hours after vaccination and last 1-2 days, being more common in individuals receiving the vaccine for the first time. Anaphylactic reactions are extremely rare.

Guillain-Barré Syndrome (GBS) can be triggered by multiple causes. In rare cases, its onset has coincided with vaccination – typically occurring between 1 day and 6 weeks post-vaccination. To date, it remains unclear whether influenza vaccination increases the risk of GBS recurrence in individuals with a prior history of the syndrome.^454^

### 7.3. Pneumococcal Vaccine

Two types of pneumococcal vaccines are currently available for clinical use: the pneumococcal polysaccharide vaccine (PPSV) and the pneumococcal conjugate vaccine (PCV). The active components in both types are capsular polysaccharides from pneumococcal serotypes commonly responsible for invasive disease.

The PPSV is composed of partially purified capsular polysaccharides from pneumococci. The only formulation available includes polysaccharides from 23 pneumococcal serotypes, which were the most common causes of pneumococcal disease in adults during the 1980s.

Conjugate vaccines (PCVs) are composed of pneumococcal capsular polysaccharides covalently linked (conjugated) to a carrier protein. Initially developed for pediatric use, early formulations (e.g., PCV10) targeted serotypes associated with childhood disease. More recent formulations have been designed to cover serotypes that more commonly affect adults (e.g., PCV13, PCV15, and since 2023, PCV20).

These vaccines are administered intramuscularly (0.5 mL per dose) and may be given concurrently with other vaccines at separate anatomical sites.^455^

### 7.4. Guidelines for Sequential PCV13 and PPSV23 Vaccination

1. Vaccination should always begin with a conjugate vaccine (PCV13), followed by the polysaccharide vaccine (PPSV23), observing a minimum interval of 2 months between doses.
2. For individuals who have previously received PPSV23 but have not been vaccinated with a conjugate vaccine, it is recommended to wait at least 12 months before administering PCV13, PCV15, or PCV20, and 5 years before administering a second dose of PPSV23. A minimum interval of 2 months should be maintained between the conjugate and polysaccharide vaccines.^455^

### 7.5. COVID-19 Vaccine

Currently, vaccination is recommended for the entire Brazilian population aged 6 months and older. The national immunization goal is to achieve 90% coverage of the full primary series (two doses or a single dose for the Janssen vaccine) and booster doses. Booster doses were introduced gradually, beginning with individuals aged 80 years and older, and later extended to younger age groups based on scientific evidence and vaccine availability.^455^

The 2023 vaccination plan proposed the use of bivalent vaccines with updated strains as booster doses for specific high-risk groups, including those more vulnerable to complications, death, or higher exposure. Monovalent vaccines are recommended to initiate or complete the vaccination series for individuals outside these priority groups.

The bivalent vaccine is indicated as a booster for individuals aged 12 years and older. It should be administered at least 3 months after completion of the primary series or the previous booster dose.

both monovalent and bivalent vaccines work by stimulating the immune system to produce protective antibodies and defense cells against SARS-CoV-2. If a vaccinated individual becomes infected with the virus, the immune system will respond more rapidly, often preventing symptoms or limiting illness to a mild form – as observed in most cases.

The primary difference between monovalent and bivalent vaccines is that bivalent formulations elicit a more effective immune response against the Omicron variant, thus providing enhanced protection against SARS-CoV-2 infection. Nevertheless, monovalent vaccines continue to protect fully vaccinated individuals against hospitalization, severe acute respiratory syndrome (SARS), and death. Current vaccination recommendations in Brazil are based on age group, vaccine availability, manufacturer guidelines, and both national and international research findings.^456^

### 7.6. Recommended Vaccines

- Adsorbed COVID-19 vaccine (inactivated) – CoronaVac (Butantan);
- COVID-19 mRNA vaccine – Comirnaty (Pfizer/Wyeth);
- Recombinant COVID-19 vaccine – Oxford/Covishield (Fiocruz and AstraZeneca);
- Recombinant COVID-19 vaccine – Janssen Vaccine (Janssen-Cilag);
- Bivalent Comirnaty (Pfizer) vaccine – for booster use in individuals over 12 years of age.

### 7.7. Risk of Myocarditis/Pericarditis Following COVID-19 Vaccination

According to data from the Brazilian National Immunization Program (*Programa Nacional de Imunizações*, PNI), a total of 501,573,962 doses of COVID-19 vaccines were administered in the country as of December 31, 2022. During this period, 154 cases of myocarditis were reported based on Brighton Collaboration criteria – a global, nonprofit vaccine safety research network for health professionals. This corresponds to an incidence of 0.031 per 100,000 doses administered. To date, no deaths from myocarditis or pericarditis with a causal association to the COVID-19 vaccine have been reported in Brazil. In contrast, the incidence of vaccine-associated myocarditis ranges from 0.58 to 2.4 cases per 100,000 doses.^457^

The overall rate of myocarditis associated with COVID-19 infection itself is approximately 30 cases per million in the general population.

## 8. Diet, Alcohol, and Weight Control

A healthy diet – characterized by the inclusion of fruits, vegetables, legumes, whole grains, nuts, and fish – has been associated with a reduction in CV mortality risk factors (Chart 8).^458–460^

**Chart 8:** Key Dietary Recommendations

Increase daily intake of fruits and vegetables to at least 400g (about five servings), excluding starchy vegetables such as potatoes and cassava.
Raise whole-grain consumption so that ≥ 50 % of total cereal intake comes from whole grains.
Partially replace animal protein with plant sources – e.g., nuts, seeds, and legumes (beans, chickpeas, lentils, etc.).
Limit added sugars to ≤ 10 % of total energy (ideally ≤ 5 % for additional benefit).
Minimize ultra-processed foods and eliminate trans-fat intake.
Substitute a portion of saturated fatty acids with unsaturated fats (monounsaturated and polyunsaturated).
Restrict alcohol to < 100 g of ethanol per week in individuals at high cardiovascular risk (≤ seven standard drinks).
Limit salt intake to ≤ 5 g per day (about 2 g of sodium).

Plant-based dietary patterns, such as vegetarian diets, the DASH (Dietary Approaches to Stop Hypertension) diet, and the Mediterranean diet, have been linked to lower all-cause and CV mortality.^461,462^ In secondary prevention studies, the Mediterranean diet has been shown to reduce the risk of CVD and the long-term progression of atherosclerosis when compared to a low-fat diet.^463,464^

Higher consumption of animal protein, particularly red meat, compared to plant-based protein, is associated with an increased risk of CV mortality.^461,462^

In the Adventist Health Study 2 (AHS-2), meat protein consumption was linked to a 61% increase in mortality risk, while substituting it with plant-based protein reduced this risk by 40%.^458^

The weekly consumption of at least two servings of fish rich in EPA and DHA is associated with reduced CV risk in both primary and secondary prevention.^213^

Low-carbohydrate diets that are high in animal protein and fat have been linked to increased mortality, including CV mortality. Data from the ARIC study showed an 18% increase in mortality risk among individuals following low-carbohydrate diets with high animal protein and fat intake. Conversely, when part of the dietary carbohydrate is replaced with plant-based protein and fat sources, mortality risk decreases. The same ARIC investigators conducted a pooled analysis with data from the PURE (Prospective Urban Rural Epidemiology) study on carbohydrate intake and mortality risk. This analysis showed that both low and high carbohydrate intake were associated with increased mortality, with the optimal range being between 50% and 55%.^465^

A diet characterized by the intake of processed foods high in sugar, salt, and saturated fat is associated with increased CV risk. Sugar intake exceeding 10% of total caloric consumption has been linked to a higher risk of CVD.^466,467^ The consumption of sugar-sweetened beverages increases the risk of type 2 diabetes and atherosclerotic cardiovascular disease (ASCVD), with a 20% increase in diabetes risk for each daily serving.^468^ Diets rich in juices, sweets, sugar-sweetened beverages, refined grains, and fried foods lead to a greater incidence of coronary events than those high in animal-based products.^469^

Saturated fat intake is associated with increased mortality risk. Saturated fats raise cholesterol levels and the risk of CCS; partial replacement with polyunsaturated fats, such as soybean oil, reduces disease risk by 29%.^470^

Replacing any type of fat with trans fats raises LDL cholesterol, apoB, TGs, and Lp(a), while lowering HDL cholesterol and apolipoprotein A1. Trans fat consumption is associated with increased CCS risk.^470^

Light to moderate alcohol consumption – defined as one to two drinks or 15 to 30 grams of ethanol per day – does not increase the risk of MI. However, intake above 100 grams per week is associated with increased all-cause and CV mortality as well as higher recurrence of events in individuals with existing CVD.^473,472^ Alcohol consumption should not be recommended as a CV protective measure.^473^

### 8.1. Weight Control

A 5% reduction in initial body weight is associated with improvements in several metabolic parameters, including a decrease in LDL-C levels.^474^

A daily caloric reduction of ≥ 500 kcal is commonly recommended for individuals who are overweight or obese.^475^

Structured programs that include self-monitoring of caloric intake and weight, along with physical activity, typically result in 5%-10% weight loss over the short (≥ 6 months) and medium term (6-12 months).^474,475^

For adults, the recommended amount of aerobic physical activity is at least 150 minutes/week of moderate intensity or 75-150 minutes/week of vigorous intensity, combined with resistance training.^475^ For individuals with obesity, increasing activity to ≥300 minutes/week and combining aerobic and resistance exercises may yield better outcomes, including greater abdominal fat loss and muscle mass gain.^476^

Body mass index (BMI) is the most widely used and standardized method for classifying overweight and obesity. BMI should be interpreted with caution in individuals with high muscle mass, older individuals, and Asian populations. In addition to BMI, waist circumference helps identify central obesity and is strongly associated with increased cardiometabolic and atherosclerotic CV risk.^477,478^ Waist circumference is considered elevated at ≥ 102 cm in men and ≥ 88 cm in women.^474,479^ Waist circumference is also a diagnostic criterion for metabolic syndrome, and when used together with BMI, it provides the most accurate assessment of obesity-related risk.^474^

## 9. Physical Activity, Cardiac Rehabilitation, and Sexual Activity

### 9.1. Lifestyle Changes and Related Factors

#### 9.1.1. Sedentary Behavior, Physical Activity, and Exercise

When addressing physical activity, it is essential to distinguish between three core concepts:

- Sedentary behavior: the time spent sitting or lying down while awake, involving activities with an energy expenditure of ≤ 1.5 METs;
- Physical activity: any body movement that increases energy expenditure above basal metabolic rate, including occupational, household, commuting, and leisure-time activities;
- Exercise: a subset of physical activity that is structured, planned, and performed with the primary goal of improving or maintaining health and/or athletic performance.^475^

Sedentary behavior contributes to the development of CAD and is associated with increased all-cause mortality, CV and cancer-related mortality, and higher incidence of type 2 diabetes.^405,480,481^ In addition to increasing physical activity levels, reducing sedentary time is critical: a) sitting for 6 hours can impair vascular function in healthy individuals;^482^ b) interrupting sedentary behavior by standing and walking for 10 minutes improves vascular function;^483^ c) a dose-response relationship exists between sedentary time and all-cause mortality, with a sharp increase in risk beyond 9.5 hours of sedentary behavior per day.^484^ The 2020 World Health Organization (WHO) Guidelines recommend the physical activity levels shown in Table 36.2^475,485^ and emphasize that any physical activity is beneficial for overall health and for reducing CV and all-cause morbidity and mortality.

**Table 36.2:** Physical activity recommendations for adults, including individuals with stable cardiovascular disease

Type	Recommendation	Examples of intensity measurement	Recommendation grade	Level of evidence
Aerobic (e.g., walking, running, cycling, swimming; continuous or in brief intervals)	150-300 min/week MI exercises; 75-150 min/week of HI exercises.	Borg RPE scale: 10-13 for MI; > 13 for HI^488^ Shortness of breath that still allows completing a sentence without pausing (MI); shortness of breath that prevents completing a full sentence (HI)^488^	**I**	**A**
Muscle strengthening (resistance or weight training)	MI or HI involving major muscle groups, 2 days/week	OMNI RPE scale: 6-8 for MI; 9-10 for HI*^489^	**I**	**A**

HI: high intensity; MI: moderate intensity; RPE: rating of perceived exertion.

* Use of the OMNI scale for RPE in resistance exercises has a Class IIa recommendation and Level C evidence.

A longitudinal study involving 15,486 individuals with CAD and a 3.7-year follow-up demonstrated a progressive reduction in CV and all-cause mortality associated with increased daily physical activity.^486^

Regarding muscle-strengthening activities (resistance training), a recent systematic review of 16 studies (maximum follow-up of 25.2 years) showed a 10% to 17% reduction in CV mortality risk with 30 to 60 minutes of resistance training per week.^487^

#### 9.1.2. Secondary Prevention (Exercise-Based Cardiac Rehabilitation)

The indication for cardiac rehabilitation as part of the treatment for individuals with CCS is a well-established consensus in both national^488^ and international guidelines^1,80^ (Class I, Level of Evidence A). This strong recommendation is based on the proven effectiveness of cardiac rehabilitation in improving QoL, reducing modifiable risk factors and mortality, and preventing hospital readmissions due to recurrent MACE. Cardiac rehabilitation has also demonstrated favorable cost-effectiveness for health care systems.^490–492^ A 5-year follow-up study reported a 32% reduction in mortality risk among patients with a history of MI or revascularization as well as among those with stable and unstable angina who participated in rehabilitation programs, compared to nonparticipants.^493^

In 2021, a systematic review including 85 randomized clinical trials and a total of 23,430 patients with CAD concluded that exercise-based cardiac rehabilitation significantly reduced the risk of MI. Exercise-based cardiac rehabilitation also reduced all-cause mortality and hospitalizations, lowered health care costs, and improved QoL within a 12-month follow-up. In the long term (more than 3 years), a reduction in CV mortality and MI risk was observed.^494^

Exercise is the core intervention in cardiac rehabilitation, particularly in enhancing physical capacity (i.e., oxygen consumption). An increase of just 1 mL·kg^-1^ ·min^-1^ in peak oxygen uptake (VO_2_max) has been associated with an approximate 15% reduction in CV mortality and a 17% reduction in all-cause mortality among patients with CAD.^495^ In individuals with chronic CAD, aerobic exercise also yields significant improvements in myocardial perfusion. In a landmark study, Hambrecht et al. subjected patients with CAD to daily aerobic training on a cycle ergometer (60 minutes per day, 7 days a week) at 80% of peak HR achieved during maximal exercise testing. The trained group showed improved coronary endothelial function compared to the control group.^496,497^

Continued exercise leads to sustained improvements in myocardial perfusion, evidenced by an increased ischemic threshold, decreased rate-pressure product during submaximal exertion, improved scintigraphic perfusion imaging,^498^ enhanced autonomic modulation,^499^ and prolonged diastolic duration. These adaptations collectively contribute to lower CV mortality and hospitalization rates, along with improved QoL (Class I, Level of Evidence A).

Although there is less evidence supporting the isolated use of resistance training for improving VO_2_max and myocardial perfusion in patients with CAD, resistance training is a valuable complementary component of a cardiac rehabilitation program. Enhanced muscular strength reduces cardiac workload during both daily activities and exercise, thereby improving FC and QoL.^500,501^

Assessment of risk prior to prescribing an exercise program is recommended.^488,503,503^ The Brazilian Cardiovascular Rehabilitation Guideline – 2020^588^ supports this approach (Table 37). Risk stratification in patients with CCS is dynamic and may change over time, necessitating periodic reassessment – semiannual or annual, depending on the initial risk classification. At least one criterion from Table 38 should be considered for risk classification. Training recommendations are presented in Table 38.

**Table 37:** Risk stratification for physical exercise in patients with chronic coronary syndrome

	Low risk	Intermediate risk	High risk
Functional capacity	ET: >7 METs CPET: Weber Class A or VO_2_ peak >85% predicted	ET: 5-7 METs CPET: VO_2_ peak 60-85% predicted	ET: <5 METs CPET: VO_2_ peak <60% predicted
Symptoms	Absent	Functional Class I-II angina and/or HF	Functional Class III-IV angina and/or HF
Signs and symptoms of myocardial ischemia	Absent	ET: ischemia or symptoms above 6 METs CPET: ischemia or symptoms above 15 mL·kg^-1^·min^-1^	ET: ischemia or symptoms below 6 METs CPET: below 15 mL·kg^-1^·min^-1^
Other clinical features	No complex ventricular arrhythmias	No complex ventricular arrhythmias	Dialysis-dependent CKD; oxygen desaturation during exertion; complex ventricular arrhythmias

CKD: chronic kidney disease; CPET: cardiopulmonary exercise test; ET: exercise test; MET: metabolic equivalents. Adapted from Carvalho et al.^488^

**Table 38:** Exercise recommendations for patients with chronic coronary syndrome

Type of activity	Low risk	Recommendation grade	Level of evidence
Aerobic exercise	**Intensity:** RPE (Borg scale 6-10) or talk test (exercise performed with shortness of breath but still able to complete a sentence without pause). 40%-80% of HRR: ([maximum HR – resting HR] × intensity) + resting HR. For previously active individuals: 50%-80%. HR between VT1 and VT2 on CPET (progressively aiming to reach VT2).	**I**	**A**
	**Frequency and duration:** At least 2 sessions/week. Start with 30 minutes per session and gradually increase to 60 minutes over time. For sessions > 60 minutes, ensure adequate hydration. If HR exceeds the upper limit during sustained effort, reduce intensity.		
	**Type:** Bicycle*, walking, running, etc.		
**Resistance training**	**Frequency and volume:** Two to three sessions/week, one to two exercises per major muscle group, one to three sets of 10 to 15 repetitions until moderate fatigue (defined by reduced movement speed or loss of form). **Intensity:** 60% of 1RM. Rest intervals: passive (90-120 s) or active (alternate between upper/lower limbs or agonist/antagonist groups). Avoid Valsalva maneuver.	**I**	**A**
	**Intermediate and high risk**		
Aerobic exercise	**Intensity:** RPE (Borg scale 11-15) or talk test (exercise performed with shortness of breath but still able to complete a sentence without pause).^#^ 40%-80% of HRR: ([maximum HR – resting HR] × intensity) + resting HR. For individuals with ventricular dysfunction: 40%-60%; for previously active individuals: 50%-80%. HR between VT1 and VT2 on CPET (progressively aiming to reach VT2). If signs/symptoms of myocardial ischemia occur, set training HR at least 10 bpm below the threshold determined during functional testing.^16^	**I**	**A**
	**Frequency and duration:** At least 2 sessions/week. Start with 30 minutes per session and gradually increase to 60 minutes over the training period.		
	**Type:** Bicycle*, walking, running.		
**Resistance training**	**Frequency and volume per session:** Two to three times per week, one to two exercises for the major muscle groups, with one to three sets of 10 to 15 repetitions until moderate fatigue (defined as a repetition with reduced speed and loss of proper movement pattern). **Intensity:** 60% of 1RM. Passive rest – 90 to 120 seconds or active rest – alternating by segment (lower and upper limbs) and/or agonist and antagonist muscles. Avoid the Valsalva maneuver.	**IIa**	**C**

1RM: one-repetition maximum; bpm: beats per minute; CPET: cardiopulmonary exercise test; ET: exercise training; HR: heart rate; LL: lower limbs; RPE: rating of perceived exertion; UL: upper limbs; VT1: first ventilatory threshold; VT2: second ventilatory threshold.

* If the exercise test is performed on a bicycle, follow the suggested HR intensity prescription. If it is performed on a treadmill, carry out the activity at 10% below the suggested level. #Use intensity monitoring by RPE or the talk test only as a complementary strategy.

Patients with greater limitations, such as those with RA who present an ischemic threshold close to their resting HR, may undergo aerobic training at the ischemic threshold. However, this should be conducted in a hospital setting under continuous monitoring (Class: IIb, Level: C).^488,504–506^ Studies suggest that short periods of exercise-induced myocardial ischemia may contribute to cardioprotection (ischemic preconditioning),^505^ but this strategy is not routinely implemented in daily rehabilitation practice.

It is recommended that the exercise session consist of a warm-up (at an intensity below the prescribed level), a main phase (at the prescribed intensity, as suggested in Table 38), and a cool-down, with a slow and gradual reduction in workload. Additionally, 15 to 30 minutes of resistance training may be included at the end of the aerobic session. Stretching and relaxation exercises are optional, depending on the time available for the session.

Specific precautions should be taken during exercise sessions for patients with CCS, including:

- Warm-up period of 5-10 minutes (extend to 10 minutes in colder environments);
- Cool-down should be gradual, lasting 5-10 minutes. For walking exercises, a reduction of 1 km/h per minute is suggested;
- BP should be measured before, during, and after the session, especially in hypertensive patients. For resting BP values above 160/105 mm Hg, adjustment of antihypertensive medications is recommended to achieve better pressure control before initiating exercise sessions;^507^
- During exercise, BP should remain ≤ 180/105 mm Hg. If it exceeds this level, exercise intensity should be reduced.^488,507^

Although moderate-intensity aerobic exercise is more commonly prescribed, high-intensity interval training (HIIT) may be a suitable alternative for time optimization. This strategy has been shown to be safe for patients with CAD^508,509^ and can significantly improve aerobic capacity.^510^ However, a preparatory phase involving moderate-intensity training (Table 38), lasting 1 to 3 months,^511^ is essential to reduce the risk of muscle injuries, which are more common with high-intensity exercise (Class: I, Level A).

#### 9.1.3. Home-Based Rehabilitation

Supervised cardiac rehabilitation has demonstrated positive effects in the management of CAD. However, several barriers may limit participation in traditional cardiac rehabilitation programs, such as patient transportation and most notably the lack of resources in certain clinical and community settings. Home-based or semi-supervised cardiac rehabilitation programs as well as telerehabilitation (remotely supervised), which include health education combined with physical exercise, may therefore represent viable alternatives.^512,513^

Home-based rehabilitation for stable patients has shown indications of CV benefit for more than 30 years.^514,515^ More recently, following the onset of the COVID-19 pandemic, this therapeutic approach has gained increased attention. In addition to its established benefits, the cost-effectiveness of home-based rehabilitation makes it particularly valuable. Nevertheless, further studies are required to support its prescription for patients under intermediate or high risk as well as for individuals without prior experience in physical exercise (Class IIa, Level A for low-risk patients).^516–519^

#### 9.1.4. Sexual Activity

Sexual activity is closely linked to QoL and is often reduced in individuals with CVD. Psychological factors associated with cardiac conditions – such as anxiety and depression – as well as erectile dysfunction and decreased libido, may contribute to this decline. Therefore, addressing this issue and offering appropriate counseling and treatment is essential and should be integrated into standard clinical practice, just as guidance on returning to work, physical exercise, and other daily activities is provided.^520^

Sexual activity may be one of the most pleasurable forms of physical exertion and can offer various benefits for patients’ physical and emotional health, including: (1) improved QoL through enhanced overall well-being and reduced stress, anxiety, and depression; (2) strengthened relationship and emotional bond between the patient and their partner; and (3) maintenance of CV health through improved blood circulation and general CV function.

Although a frequent concern for both health professionals and patients with CAD, the risk of sudden death or MI during sexual activity is very low. This is especially true when it occurs with a stable partner, in a familiar environment, without excessive emotional stress or the use of drugs and/or heavy alcohol consumption.^520,521^ In such scenarios, sexual activity is typically considered low to moderate in intensity (2-4 METs). Notably, sudden death during sexual activity accounts for less than 2% of all exercise-related deaths.^522–524^ The risk of MI is transiently increased – approximately threefold – for 2 to 3 hours after intercourse when compared to other activities with similar energy expenditure. However, this risk is significantly lower in physically active individuals.^525–527^

Sexual activity is generally well tolerated by most patients with chronic CAD and encompasses a range of behaviors, including kissing (Ki), touching (To), oral stimulation (O) of the genitals, masturbation (M), and penetrative intercourse (I). Using the initials of these actions, Brazilian researchers proposed the acronym KiTOMI (from Kiss, Touch, Oral sex, Masturbation, and Intercourse).^41^ After major cardiac events or procedures, sexual activity should be resumed gradually, beginning with KiT and progressively advancing to KiTOM, before returning to full engagement in KiTOMI behaviors. The recommendation grade and level of evidence, when available, are presented in Figure 20.^520^

**Figure 20:** Ischemic heart disease and recommendations for sexual activity. NYHA: New York Heart Association; CCS: Canadian Cardiovascular Society; KiT: Kiss and Touch; KiTOM: Kiss, Touch, Oral, and Masturbation; KiTOMI: Kiss, Touch, Oral, Masturbation, and Intercourse. Reproduced with permission from the Canadian Journal of Cardiology.

Lastly, in cases of sexual dysfunction related to CCS, psychological interventions may be helpful in addressing contributing psychological factors such as anxiety, depression, and low self-esteem. Individual or couples therapy can be effective in treating erectile dysfunction, low libido, and other sexual disorders. In addition, certain medications – such as BBs and thiazide diuretics – may be associated with sexual dysfunction. However, this association has not been confirmed with newer classes of medications. Among BBs, nebivolol appears to have a lower impact on sexual function. Special caution is warranted when using phosphodiesterase type 5 inhibitors to treat erectile dysfunction; although generally safe for patients with CCS, these drugs should not be used in patients taking nitrates.

## 10. Tobacco Use and Environmental Pollution

Both tobacco smoke and air pollution contain carbon dioxide, carbon monoxide, and nitrogen oxides. Tobacco smoke, however, also contains significant amounts of hydrogen cyanide, nicotine, tar, benzene, acrolein, and nitrosamines.^528^ Air pollution resulting from fossil fuel combustion tends to have higher levels of nitrogen dioxide (NO_2_).^529^ Both sources contain particulate matter (PM) of varying sizes, particularly ultrafine particles in the nanometer range. These combustion-derived particles are primarily composed of carbon and carry a wide array of surface-bound chemicals, including organic carbon species and trace metals – both of which have been implicated in adverse health effects.

Indoor tobacco use contributes significantly to air pollution.^528^ When tobacco is burned, it releases toxic substances such as nicotine, carbon monoxide, nitrogen oxides, volatile organic compounds (VOCs), and fine particulate matter (PM2.5 – particles smaller than 2.5 micrometers in diameter) into the surrounding air.^529^ These fine particles are particularly concerning because they can penetrate deep into the respiratory system and are highly pathogenic. Secondhand smoke is defined as a combination of the smoke emitted from the burning end of a cigarette or other tobacco products and the smoke exhaled by the smoker. It is a major public health concern due to its well-documented harmful effects on human health^530^ and is a significant source of indoor PM2.5.^528^ Consequently, exposure to secondhand smoke involves the involuntary inhalation of tobacco smoke in the vicinity of active smokers or in indoor environments where tobacco products were recently used. Thirdhand smoke (THS) refers to residual gases and particulate matter from tobacco smoke that settle on surfaces.^531^ These pollutants can persist long after secondhand smoke has dissipated. Studies evaluating the presence of tobacco-specific nitrosamines (TSNAs) in THS have shown that these compounds are potent carcinogens.^532^

Studies have demonstrated that levels of PM2.5, VOCs, and carbon monoxide are significantly higher in indoor environments where smoking is allowed compared to smoke-free environments.^533^ After the implementation of indoor smoke-free policies in hospitality venues (including bars, cafés, restaurants, and pubs), smokers were displaced to outdoor areas, prompting studies to assess outdoor tobacco smoke (OTS) exposure in these new settings. Sureda et al.^534^ found that OTS exposure is prevalent in outdoor hospitality environments, particularly in poorly ventilated areas. Hwang et al.^535^ and Kim et al.^536^ demonstrated that OTS exposure is detectable at distances of up to 9 meters and 3 meters, respectively, from a smoking source. Ruprecht et al.^537^ further highlighted the negative effects of OTS on air quality, with significant implications for public health. These findings underscore the need for effective policies and interventions to mitigate OTS exposure in outdoor environments. Outdoor smoking areas should include buffer zones to reduce exposure to OTS.^536^

The impact of electronic cigarettes (vapes) in indoor environments, referred to as secondhand vaping, should also be considered. Secondhand vaping occurs when the aerosol from e-cigarettes is exhaled by users in public or private enclosed spaces.^538^ While the use of conventional cigarettes indoors is well established as a major source of both gaseous and particulate pollutants – resulting in high levels of exposure to carcinogenic compounds and particulate matter for both active and passive smokers – exposure to secondhand e-cigarette aerosol is not risk-free. This is due to the high concentration of ultrafine particles (nanoparticles, i.e., particles smaller than 1 micrometer),^539^ which can deeply penetrate the human respiratory system and deliver chemical contaminants to these regions, causing inflammation. Therefore, vaping can lead to both secondhand and thirdhand exposure to e-cigarette aerosol. Some studies suggest that thirdhand exposures from e-cigarettes may be comparable to those from conventional smoking, particularly regarding the elevated quantities of ultrafine particles and nitrosamines.^538,539^ One study^538^ monitored pollutants in vape shops. During business hours, the concentrations of PM2.5, formaldehyde, acetaldehyde, and nicotine in the air were 21, 3.3, 4.0, and 3.8 times higher, respectively, compared to levels after business hours. PM2.5 concentrations were positively correlated with the number of e-cigarette users present in the vape shops. Surface contamination with nicotine, 4-(N-methyl-N-nitrosamino)-4-(3-pyridyl)butanal (NNA), and 4-(methylnitrosamino)-1-(3-pyridyl)-1-butanone (NNK) was also detected at levels of 223.6 ± 313.2 *μ*g/m^2^, 4.78 ± 11.8 ng/m^2^, and 44.8 ± 102.3 ng/m^2^, respectively. Substantial amounts of nicotine (up to 2073 *μ*g/m^2^) were deposited on materials placed inside vape shops, along with NNA (up to 474.4 ng/m^2^) and NNK (up to 184.0 ng/m^2^). The deposited nicotine concentrations were strongly correlated with the average number of active e-cigarette users per hour in a given vape shop. NNK levels on material surfaces were significantly associated with nicotine levels on those same surfaces.

The association between pollution, including tobacco-related pollution, and CVDs is well established, with multiple epidemiological studies consistently documenting a link between pollutant exposure and increased risk of adverse MACE such as MI, stroke, and SCD.^540^ The underlying mechanisms are complex and involve a series of biological processes contributing to the pathogenesis of CVDs. Oxidative stress, inflammation, endothelial dysfunction, autonomic imbalance, and vascular remodeling are all considered key mediators of the CV effects of air pollution.^541^

Oxidative stress is a key process in the pathogenesis of pollution-induced CVDs.^542^ Exposure to pollutants such as fine particulate matter (PM2.5), present in both conventional and electronic cigarettes, leads to increased generation of reactive oxygen species (ROS) and a reduction in the body's antioxidant capacity.^543^ This disruption of redox balance results in oxidative damage to cellular components, including lipids, proteins, and DNA, triggering inflammatory and pro-atherogenic pathways.^544^ Inflammation plays a central role in the biological response to air pollution exposure. Inhalation of air pollutants triggers an inflammatory response in the airways and lungs, characterized by increased production of pro-inflammatory cytokines, chemokines, and lipid mediators.^544^ These inflammatory mediators circulate throughout the body and can initiate systemic inflammation and endothelial dysfunction, thereby promoting the formation and progression of atherosclerosis.^645^

Acute exposure to air pollutants decreases the bioavailability of nitric oxide (NO), a key regulator of vascular function, and increases the endothelial production of ROS, including hydrogen peroxide (H_2_O_2_) and superoxide (O_2_•^–^).^543^ These changes promote vasoconstriction, vascular inflammation, and platelet aggregation, all of which contribute to the development of MACE.^541^

Air pollution can affect the autonomic nervous system,^547^ disrupting CV regulation and promoting cardiac arrhythmias^548^ and elevated BP.^549^ Exposure to air pollutants may trigger dysfunctional autonomic responses, including increased sympathetic activity and decreased parasympathetic activity, both of which are associated with alterations in HR variability and a higher risk of MACE.^548^

Vascular remodeling is another mechanism through which air pollution can contribute to the development of CVDs. Chronic exposure to air pollutants is associated with structural changes in the arteries, including arterial wall thickening, increased vascular stiffness, and the formation of atherosclerotic plaques.^550^ These morphological changes impair arterial function and increase the risk of MACE.

In summary, air pollution exerts adverse effects on the CV system through a variety of mechanisms, including oxidative stress, inflammation, endothelial dysfunction, autonomic dysfunction, and vascular remodeling. Understanding these mechanisms is crucial for the development of effective prevention and intervention strategies aimed at mitigating the CV effects of air pollution.

## 11. Psychosocial Factors and Treatment Adherence

### 11.1. Psychosocial Factors

Negative psychological factors (e.g., anxiety, stress, and depression), personality traits, and mental disorders have a bidirectional relationship with CCS. In other words, they are risk factors for its development and also for its complications.^551,552^ The causal mechanisms linking mental disorders and CAD are complex and remain unclear.^551–554^ In addition to causality, a common pathophysiological mechanism is believed to exist between mental disorders and CAD, along with the coexistence of other confounding risk factors (such as physical inactivity, smoking, hypertension, and obesity),^617^ which hinders the development of clear evidence.

The association between anxiety and stress with CV morbidity and mortality also remains inconclusive.^551^ However, evidence shows that approximately 40% of individuals with CVD, including CAD, experience depression or anxiety.^553^ Regarding depression, prospective studies have demonstrated that it is an independent risk factor for both morbidity and mortality related to coronary heart disease.^551^

In addition to poorer outcomes, psychosocial factors (such as depressive mood, stress, anxiety, and social isolation) hinder the adoption of lifestyle changes and may compromise treatment adherence.^1^

In addition to pharmacological treatment, emotional support is also needed to address stress, anxiety, and depression in patients with stable angina,^555^ along with referral to psychotherapy.^1^ here is evidence supporting the efficacy of specific psychological interventions (including traditional ones such as cognitive-behavioral therapy and third-wave therapies like mindfulness) with moderate-level evidence for reducing depression and anxiety; and low-level evidence for improving mental health-related QoL, but not physical health. These interventions have not proven effective in reducing mortality (moderate-level evidence) or in lowering the risk of MACE (low-level evidence).^553^

Nevertheless, psychological interventions are beneficial in enhancing personalized mental health care, allowing CCS treatment to be tailored to each individual's specific needs, capabilities, and preferences – in other words, placing the patient at the center of care rather than the disease.^552,554^ The inclusion of this topic in the present guideline, compared to the 2014 version, represents significant progress. Beyond clinical outcomes, it reflects a shift toward recognizing the patient's experience with illness and the health-disease process.

### 11.2. Treatment Adherence

Adherence to both pharmacological and nonpharmacological treatment is a major challenge and a critical need. Several studies have aimed to identify interventions that can improve adherence, including the use of digital technologies such as smartphone apps^556^ and text messaging,^557,558^ which have shown no favorable results; and mobile phone-based self-management interventions^559^ and medication reminder apps,^560^ which showed improvements in treatment adherence but still require more robust evidence. Positive results have also been observed in adherence to diet, physical activity, and medication through patient-centered education,^591^ although further research is also needed in this area.

Other variables related to treatment adherence have also been studied. Women are less likely than men to adhere to cardiac rehabilitation programs,^562^ and low health literacy has been associated with reduced adherence to anti-anginal medications.^563^

## 12. Angina and Ischemia with Nonobstructive Coronary Arteries (INOCA, ANOCA)

The term "ischemic heart disease" (IHD) is now more appropriate to describe the broad spectrum of coronary conditions that cause ischemia, previously referred to as "coronary artery disease" (CAD). IHD includes CAD (now more precisely termed atherosclerotic cardiovascular disease [ASCVD]), INOCA, angina with nonobstructive coronary arteries (ANOCA), MI with nonobstructive coronary arteries (MINOCA), spontaneous coronary artery dissection (SCAD), CMD, CAS, and coronary thrombosis/embolism (CTE).^564^

Chest pain is the key symptom of acute MI in both sexes. However, women are more likely to present with so-called atypical symptoms, such as back and neck pain, fatigue, nausea, and vomiting. Most women with acute MI report prodromal symptoms such as shortness of breath, unusual fatigue, or discomfort in the arm/jaw in the weeks preceding the acute event. Stable angina is the most common clinical presentation in women with IHD, but women's specific clinical manifestations may lead to delays in the diagnosis of ischemia.^564,565^

Therefore, the concept of obstructive CAD as synonymous with angina and myocardial ischemia should be reconsidered, as it no longer applies given the current understanding of ischemia pathophysiology – since stenosis is not always present to restrict blood flow. Additionally, approximately two-thirds of women and at least one-third of men with myocardial ischemia do not present with obstructive CAD on angiography. Over recent decades, the pathophysiology of CAD has been better understood, although many aspects remain unclear.^566^

Sex-related disparities tend to diminish after menopause. From the seventh decade of life onward, similar rates of CAD prevalence and acute MI-related mortality are observed in men and women. Several models have been proposed to explain these findings, including sex-specific exposure to traditional CV risk factors, female-specific biological factors, and psychosocial health determinants, which have a greater impact on women.^566^

### 12.1. General Characteristics of Female Ischemic Heart Disease

#### 12.1.1. Atherosclerotic Cardiovascular Disease^564,565,567^

ASCVD is more prevalent in older women. In women, atherosclerotic plaques tend to be less voluminous and less calcified, and plaque erosion is a more frequent mechanism. The disease is more diffuse, affecting the entire arterial system, and is often associated with microvascular dysfunction and endothelial dysfunction.

Prevention: Prevention of ASCVD in women – both primary and secondary – requires awareness of the clinical differences in ACS and CCS as well as their specific features in females in order to ensure accurate diagnosis and treatment. Early identification of traditional CV risk factors (CVRFs), female-specific risk factors, and psychosocial variables is also essential.

Outcomes: Women with CAD present higher in-hospital mortality, and younger women (< 50 years) have even higher mortality rates, representing a high-priority group for understanding the underlying pathophysiological mechanisms.

#### 12.1.2. ANOCA and INOCA^564,568^

INOCA is more common in women than in men, with particularly high prevalence among women aged 45 to 65 years.

Prevention: Effective prevention requires aggressive identification and management of both traditional CVRFs and female-specific risk factors, as well as identifying amplifying factors such as psychosocial stressors and social determinants of health.

Outcomes: INOCA in women is associated with recurrent angina, frequent hospitalizations, repeated ICAs, and high rates of MACE.

#### 12.1.3. MINOCA^564,569^

MINOCA is associated with pathophysiological mechanisms such as CVS, microvascular dysfunction, CTE, plaque rupture, and SCAD. Its prevalence ranges from 5% to 10% of all acute MI cases, with about two-thirds of patients presenting with non-ST-elevation acute MI (NSTEMI).

MINOCA is more common in younger women than in men (10.5% vs. 3.4%; p < 0.0001). Traditional CVRFs may be present, but are less frequent compared to patients with CAD.

The diagnosis of MINOCA is provisional and requires confirmation of the underlying cause. Identifying this cause is critical, as failure to do so may lead to inappropriate treatment and incorrect clinical information.

Prevention: Identify and manage both traditional CVRFs and female-specific risk factors. Identify amplifying factors such as psychosocial stressors and social determinants of health.

Outcomes: Prognosis depends on the underlying cause; morbidity and mortality are similar to those observed in DAC.

#### 12.1.4. Microvascular Disease^564,567^

The pathophysiology of MVD results from structural remodeling that reduces conductance, vasomotor dysfunction affecting arterioles, or both. A confirmed diagnosis of MVD must meet the following criteria: presence of symptoms (e.g., angina and/or exertional dyspnea); absence of obstructive coronary disease; objective evidence of ischemia; and microvascular dysfunction (reversible defects, abnormalities in invasive functional testing – FFR > 0.8; CFR < 2.0; and index of microcirculatory resistance [IMR] > 25). A newer method not requiring pharmacologic testing can estimate coronary resistance in each main artery. This approach has already been adopted and is being refined to avoid vasospasm, as seen with intravenous acetylcholine testing.

The persistence and/or recurrence of chest pain in women remains one of the greatest clinical challenges. This condition not only worsens patient prognosis, but also contributes to anxiety related to diagnosis, impairs QoL, and results in high health care costs related to diagnosis and treatment.

Prevention: Modification of CVRFs (particularly weight loss and stress control).

Outcomes: MVD is associated with recurrent angina, frequent hospitalizations, repeated ICAs, and high rates of MACE.

#### 12.1.5. Spontaneous Coronary Artery Dissection^564,567^

SCAD is a rare cause of MI, accounting for 1% to 4% of all ACS. SCAD is increasingly recognized as a significant cause of MI in women under 50 years of age. According to some studies, 25% to 35% of SCAD cases occur in women under 50 years, and 25% in women over 60 years. It is the most common cause of pregnancy-associated MI (up to 43%), especially during the third trimester or postpartum period. The risk of recurrence is substantial.^564^

In this condition, traditional CVRFs are often absent. However, there are predisposing factors for dissection, including fibromuscular dysplasia (50% to 86%), connective tissue disorders (5%), systemic inflammatory diseases (5% to 12%), use of hormonal therapy (estrogen, progesterone, gonadotropin, clomiphene, or infertility treatment), and history of multiple pregnancies.

Prevention: Minimize emotional triggers, avoid hormonal therapy and future pregnancies as secondary prevention strategies. Cardiac rehabilitation is recommended, preferably with a modified protocol avoiding heavy isometric exercises and high-intensity aerobic activities.

Outcomes: SCAD recurrence is common, particularly in the postpartum period and in individuals with connective tissue disorders.

#### 12.1.6. Coronary Vasospasm^564,570^

CVS is characterized by reversible vasoconstriction, either diffuse or focal, in the coronary arteries. This condition is commonly observed in patients with IHD and is involved in the mechanisms of INOCA, MINOCA, and MVD, regardless of racial, genetic, or geographic differences. The prevalence of CVS is higher among women aged 40 to 70 years.

Intracoronary acetylcholine provocative testing remains the main diagnostic tool to reproduce CVS and assess nitrate reactivity, although it is not widely used in clinical practice. Notably, women typically require lower doses of acetylcholine to elicit the desired response.

Prevention: Prevention involves avoiding aggravating factors such as illicit drugs, amphetamines, butane gas, alcohol, and migraine medications that may induce vasoconstriction.

Outcomes: The recurrence rate of CVS ranges from 3.9% to 18.6%. Cardiac arrhythmias and SCD may also be associated with CVS.

#### 12.1.7. Coronary Thrombosis/Embolism^564,571^

CTE is considered a rare phenomenon, with an estimated incidence of approximately 0.06%. The etiologies of CTE include valvular disease, cardiomyopathy, and AF, with bacterial endocarditis accounting for about 40% to 53% of cases.

CTE is an underdiagnosed cause of ACS and can be classified into three types: direct, paradoxical (thrombus originating from deep vein thrombosis crossing through a patent foramen ovale), and iatrogenic. In the latter, PCI is the most common cause of embolism, with increased risk when rotational atherectomy, valvuloplasty, or inadequate procedural anticoagulation is used.

Prevention: The best method of prevention is prophylaxis against thrombosis/infection and early diagnosis of underlying causes.

Outcomes: The prognosis is favorable in most cases if both diagnosis and thrombectomy are performed early.

A recent review provided updated epidemiological data and clearer pathophysiological mechanisms for INOCA and ANOCA. It revealed that the prevalence of INOCA in men is nearly equal to that in women, reaching 46% in the ISCHEMIA study – possibly because men undergo more functional ischemia testing and, when ischemia is present, are further evaluated by anatomical imaging.^571,572^

## 13. Ischemic Disease in Women – Female-Specific Aspects

ICM in women encompasses a spectrum of complexities influenced by physiological, hormonal, and clinical factors that differ from those observed in men. Addressing these differences through sex-specific research and personalized therapeutic approaches is imperative to improve outcomes in women. A better understanding of these disparities will lead to improved diagnostic, prognostic, and therapeutic strategies, ultimately narrowing the CV care gap between sexes.^565^

Women tend to have worse outcomes after acute MI. They show higher rates of rehospitalization and mortality compared to men, with values remaining elevated at 1 year (26% vs. 19%) and 5 years (47% vs. 36%) after acute MI. Symptomatic HF after acute MI is more prevalent in older women, with the increased risk extending beyond the initial episode.^573^

Despite having better LVEF and a lower burden of obstructive CAD, women with ICM report lower FC and QoL, although their mortality rates are similar to those of men.^574^ Hypertension and diabetes contribute more significantly to the risk of HF in women, presenting a distinct phenotypic pattern.^575^

### 13.1. Clinical Presentation and Diagnosis

Women often present with more symptomatic HF, including dyspnea and orthopnea more frequently than men. Obesity, more prevalent among women with ICM, correlates with worse outcomes compared to men.^643^

Noninvasive diagnostic modalities play a crucial role in the assessment of ICM in women. Techniques such as CCTA and CMR demonstrate high sensitivity and performance, aiding in the identification of hemodynamically significant lesions and providing information on nonischemic etiologies. The selection of diagnostic modalities should consider sex-specific factors and special conditions such as pregnancy.^576^

### 13.2. Treatment and Management

Treatment disparities persist between the sexes. Women are less likely to receive timely and guideline-directed therapies for acute MI, contributing to unfavorable outcomes. Men are more frequently referred for treatment and receive guideline-directed therapies more consistently.^565^

Invasive evaluations, such as ICA, remain essential particularly in severe cases of cardiomyopathy or when diagnostic uncertainty persists despite noninvasive methods. Despite advances, women remain underrepresented in clinical trials, limiting the optimization of therapeutic strategies for them.^577^

The impact of sex hormones and epigenetic factors on cardiac remodeling under ischemic conditions is noteworthy. Female mitochondria demonstrate better tolerance to oxygen deprivation and oxidative damage than male mitochondria, potentially due to the protective effects of estrogen. Estrogen may also reduce calcium levels prior to ischemia, attenuating ischemia-reperfusion injury in women.^575^

### 13.3. Prognostic Factors and Outcomes

Several factors, including the extent of ischemic injury and the presence of comorbidities such as hypertension and diabetes, influence the prognosis of women with ICM. Women are more likely to develop HF with preserved ejection fraction (HFpEF), whereas men more commonly experience HFrEF, typically due to CAD leading to dilated cardiomyopathy.^578^

Women with ICM also face higher risks of complications after myocardial revascularization and heart transplantation. They experience higher rates of complications such as graft rejection but tend to have better posttransplant survival rates compared to men. However, the use of arterial grafts in myocardial revascularization is less frequent in women, negatively impacting postoperative outcomes.^578^

Despite ongoing discussions regarding sex disparities in CV care, women remain underrepresented in clinical trials, which affects the generalizability of research findings to female populations. The limited participation of women in clinical studies highlights the need for more inclusive research to optimize treatment protocols and improve outcomes for women with ICM.^579^

### 13.4. Hormonal Influence and Genetic Factors

Hormonal variations – particularly estrogen – play a protective role against oxidative stress and apoptosis, which is crucial in ischemic injury. Elevated calcium levels, which exacerbate ischemia-reperfusion injury, are modulated by estrogen, thereby reducing damage in women.^578^

Additionally, sex chromosomes and epigenetic mechanisms contribute to sex differences in cardiac remodeling. Genes related to adverse cardiac remodeling processes, such as macrophage activation and lipid metabolism, are located on the X chromosome and influence outcomes differently in men and women.^580^

## 14. Refractory Angina

RA is a chronic condition defined by the presence of limiting angina, or its equivalent, lasting for more than 3 months, due to myocardial ischemia in the presence of CAD, not controlled by OMT – including a combination of tolerated antianginal drugs – in individuals who are not eligible for myocardial revascularization.^581,582^

The prevalence of RA is estimated to range from 5% to 15% of the population with CAD.^583^ According to Brazilian data,^584^ the incidence of combined events and death in this population is 7.7% and 4.4% at 1 year, respectively, and 24.4% and 13.5% at 5 years.

Patients with RA experience significant impairment in QoL,^585,586^ and this is the focus of treatment. Dedicated multidisciplinary centers with experience in RA promote greater treatment effectiveness and improved QoL, and referral of patients should be considered.^586–588^

There are promising options for the treatment of RA (Table 39), some of which are still experimental. The safety and efficacy outcomes of studies evaluating various therapies are variable. In Brazil, some options are currently available for clinical use (Table 40).

**Table 39:** Alternative therapies for refractory angina

Alternative therapies
**1. Exercise-based cardiac rehabilitation** A low-cost, noninvasive adjuvant therapy that improves functional capacity and anginal symptoms.^589,590^ It may be considered a treatment option for RA^504,591^ provided patients undergo a proper preparticipation evaluation and the program is conducted in a supervised, hospital-based setting.
**2. Neuromodulation** Spinal cord stimulation has shown variable efficacy results; it may improve anginal symptoms and quality of life, and can be considered a treatment option in RA when other therapies have failed.^592–595^ Subcutaneous and transcutaneous neuromodulation and sympathectomy have not demonstrated consistent efficacy to support their use in RA.^596,597^
**3. Percutaneous recanalization of chronic total occlusion** Success rates have increased significantly, reaching up to 90%, with low complication rates.^598,599^ This approach improves angina and quality of life^600–602^ and is a therapeutic option for RA in experienced centers.
**4.** **Shockwave therapy** A noninvasive technique that uses low-energy shockwaves to stimulate angiogenesis in ischemic myocardium. Although it improves myocardial perfusion, consistent symptom reduction has not been demonstrated.^593–605^ Therefore, there is insufficient evidence to recommend it as a treatment for RA.
**5. Acupunture** Provides control of angina episodes, reduces sublingual nitrate use, increases exercise duration, and improves anxiety and depression levels.^606–608^ May be considered as a therapeutic option for RA in centers experienced with traditional Chinese medicine.
**6.** **Coronary sinus reducer** A percutaneous technique involving implantation of an hourglass-shaped device that creates stenosis in the coronary sinus, increasing coronary sinus pressure and thereby enhancing perfusion of ischemic myocardium in the left coronary territory. It improves anginal symptoms, quality of life, and ischemic threshold.^609^ Not currently available in Brazil.
**7. Enhanced external counterpulsation** A technique that uses diastolic inflation of cuffs on the lower limbs, synchronized via electrocardiogram, to increase arterial pressure and retrograde aortic blood flow, enhancing coronary perfusion. It reduces anginal episodes and improves ischemic threshold. The therapy is generally well tolerated, despite some nonlimiting side effects.^610^ Not currently available in Brazil.
**8. Cell and gene therapy** Experimental therapies with initial promising results in symptom improvement.^611,612^
**9. Lp(a) apheresis** Improves angina, quality of life, exercise duration, and myocardial perfusion.^613^ Not currently available in Brazil.
**10. Heart transplant** Remains the last-line therapeutic option for RA, especially in patients with preserved ventricular function. Posttransplant mortality in Brazil remains high, reaching up to 30% at 1 year.^614^
**11. Transmyocardial laser revascularization** A technique that uses laser ablation to create transmural channels in the ischemic myocardium to stimulate neovascularization and relieve anginal symptoms. There is no established evidence of efficacy,^615^ and the procedure carries a high risk of complications, making it contraindicated in the treatment of RA.

RA: refractory angina.

**Table 40:** Recommendations for the Treatment of Refractory Angina

Recommendation	Recommendation grade	Level of evidence
Percutaneous recanalization of chronic total occlusion	**IIa**	**A**
Exercise-based cardiac rehabilitation	**IIb**	**B**
Spinal cord stimulation	**IIb**	**C**
Acupuncture	**IIb**	**A**
Heart transplant	**IIb**	**C**
Transmyocardial laser revascularization	**III**	**A**

## 15. Myocardial Viability

Myocardial viability refers to living, viable myocardial tissue. Revascularization of dysfunctional yet viable myocardial segments may lead to improved contractility and ventricular function. Revascularizing these segments could reduce the risk of future events, regardless of improvements in contractility. Extensive areas of myocardial fibrosis are unlikely to recover contractile function.

Although these concepts are biologically plausible, there is very limited evidence that myocardial revascularization improves ventricular function or reduces clinical outcomes. In contrast, pharmacological therapy is definitively associated with reduced clinical events in this population. The viability substudy of the STICH (Surgical Treatment for Ischaemic Heart Failure) trial^616^ and the HEART (Heart Failure Revascularization Trial)^344^ failed to demonstrate that myocardial viability assessment identifies patients with ICM who would benefit from surgical myocardial revascularization. Similarly, the recent REVIVED-BCIS2 trial^143^ failed to show an association between viability in dysfunctional segments and outcome reduction with PCI. These findings have led clinicians, surgeons, interventional cardiologists, and guideline committees to reconsider the role of viability in selecting patients for revascularization procedures.

### 15.1. Myocardial Viability

Viability simply refers to cardiomyocytes without irreversible damage. The concept is grounded in the pathophysiology of hibernating myocardium, in which dysfunction arises from an adaptive and survival mechanism in cardiomyocytes subjected to recurrent ischemic episodes. This definition corresponds to the concept of myocardial hibernation – an adaptive process involving downregulation of myocardial function to promote cell survival due to decreased oxygen supply.

Hibernating myocardium exists along a clinical spectrum of ischemia-induced dysfunction, which includes 1) myocardial stunning – caused by acute ischemia and reduced contractility during a period of hypoperfusion, without cell death. After coronary obstruction is resolved, segmental dysfunction persists for hours to days, but resolves spontaneously; 2) hibernating myocardium – characterized by prolonged contractile impairment due to a fixed obstructive lesion combined with repeated nonlethal ischemic episodes; 3) fibrosis – resulting from prolonged ischemia, leading to complete cellular necrosis and replacement of normal tissue by fibrotic tissue,^618^ with a very low likelihood of functional recovery.

### 15.2. Observational Studies on Viability

Myocardial hibernation represents reversible contractile dysfunction, and revascularization would be expected to improve ventricular function and, consequently, survival. This biologically plausible assumption has been observed in a number of retrospective observational studies.^619^

These studies were all retrospective, typically conducted in single centers, and subject to significant selection bias. Patients with better prognosis and lower risk were generally selected for surgical treatment, while those with poorer prognosis and higher risk remained under medical management. Furthermore, these studies were conducted before the advent of contemporary pharmacological therapies for both HF and CAD.

### 15.3. Viability and Improvement in Ventricular Function

Several methodologies are available for the assessment of myocardial viability. These tests are validated for detecting viable myocardium and predicting ventricular dysfunction recovery following revascularization. Improvement in contractility may be immediate after revascularization or occur over hours, days, or even months, depending on the severity of myocardial involvement – ranging from stunned myocardium to advanced hibernation. Each viability detection method targets different pathophysiological aspects of hibernation and provides either quantitative and/or qualitative measures of viability to predict functional recovery.^600,621^

Numerous observational studies have shown that viability detection tests can predict improvement in both segmental and global LV function.^619^ A meta-analysis of 158 studies demonstrated that all modalities are accurate in detecting myocardial viability.^95^

Several studies have demonstrated a relationship between the amount of viable tissue and the likelihood of regional contractile recovery. Importantly, the effects of revascularization cannot be completely separated from the effects of pharmacological therapy, as viability identifies a myocardial substrate that may respond positively to a range of interventions – not exclusively to revascularization.^622^

### 15.4. Contemporary Studies: STICH and REVIVED-BCIS2

Unlike earlier retrospective studies with significant patient selection bias, the contemporary STICH and REVIVED-BCIS2 trials were prospective and randomized. These trials avoided selecting lower-risk patients for invasive procedures and higher-risk patients for medical treatment. Moreover, they were conducted in the context of OMT for both CAD and HFrEF.

The STICH (Surgical Treatment for Ischemic Heart Failure) trial enrolled 1,212 patients with ventricular dysfunction. Velazquez et al.^623^ demonstrated that CABG in addition to OMT led to lower mortality, fewer deaths from all causes, reduced CV mortality, and fewer deaths or hospitalizations due to cardiac causes. As a result, this approach received a Class I-B recommendation by the 2018 ESC and 2021 American Heart Association (AHA)/American College of Cardiology (ACC) guidelines, regardless of assessment of myocardial viability.

The STICH viability substudy^624^ included 601 patients, of whom 81% were identified as having viable myocardium. Patients with myocardial viability had lower mortality rates compared to those without viability (HR 0.64; 95% CI 0.48-0.86; p = 0.003). However, after adjustment for other significant variables in a multivariate model, prespecified viability status was no longer statistically significant (p = 0.21).

In the 10-year follow-up analysis (23) of these 601 patients, no association was found between myocardial viability and improved survival after CABG. Although viability was associated with a modest improvement in LVEF in both surgical and medical treatment groups, this improvement did not correlate with better survival outcomes.

The lack of association between myocardial viability and reduced mortality after CABG suggests that viability assessment alone should not guide therapeutic decision-making. These findings contrast with earlier viability studies.

The REVIVED-BCIS2 trial^615^ was the first study specifically designed to assess the role of PCI in patients with ventricular dysfunction and evidence of viability. It was a prospective, multicenter, open-label, randomized trial. Patients were assigned to either PCI or OMT alone. A total of 700 patients were included, all with LVEF ≤ 35% and viability in ≥4 dysfunctional segments deemed suitable for PCI. Patients with recent MI (within 4 weeks prior to randomization) were excluded. The primary outcome was a composite of death and hospitalization for HF, while secondary outcomes included changes in LVEF and QoL. After a median follow-up of 41 months, there was no significant difference in the primary outcome between the PCI and OMT groups (37.2% vs. 38.0%; HR 0.99; 95% CI 0.78-1.27; p = 0.96). Additionally, no differences were observed in LVEF or QoL between the groups at 2 years.

Additionally, fibrosis assessed by LGE and quantified by CMR was found to be associated with worse prognosis in these patients. For every 10% increase in fibrosis, there was an 18% increase in the risk of combined events (HR 1.18; 95% CI 1.04-1.33; p = 0.009). Moreover, the extent of fibrosis was also associated with the probability of LVEF recovery (OR 0.69; 95% CI 0.56-0.84). Another relevant finding was the association between LVEF improvement and survival: patients who had an improvement of ≥ 4.7% in LVEF showed a lower incidence of combined events (death and hospitalization due to HF) (HR 0.62; 95% CI 0.41-0.95; p = 0.029).

The amount of viable myocardium was also not associated with a reduction in the primary outcome or with improvement in LVEF after revascularization.^625^

Another important analysis evaluated the impact of complete anatomical revascularization or revascularization guided by myocardial viability.^626^ PCI was also not associated with improved event-free survival.

The REVIVED-BCIS2 trial included only patients with viable myocardium, who were therefore considered at lower risk. There was a lower proportion of patients with 3VD compared with the STICH trial, also suggesting a lower-risk population. However, patients enrolled in REVIVED had a mean age of 70 years and included those with LMCAD, who had been excluded from other PCI studies.

These results confirm the findings from the STICH trial. The hypothesis that patients with HFrEF, receiving guideline-directed medical therapy (GDMT), would benefit from revascularization of viable but dysfunctional myocardial segments was not supported. Revascularization was not associated with improved event-free survival, regardless of the amount of viable myocardium or the completeness of revascularization.

### 15.5. Pharmacological Therapy in Coronary Artery Disease and Heart Failure with Reduced Ejection Fraction

Evidence-based pharmacological therapy includes medications targeting both CAD and HFrEF.

In the STICH trial, SGLT2i and angiotensin receptor-neprilysin inhibitors (ARNIs) were not yet available, whereas they were already in use at the time of the REVIVED trial. Dysfunctional but viable myocardial tissue may exhibit improved segmental contractility with the use of BBs. These drugs can enhance the function of viable myocardium by reducing oxygen consumption and increasing diastolic perfusion, as demonstrated in the CHRISTMAS trial.^727^

OMT in patients with CAD and HFrEF alone is associated with reduced mortality. A subanalysis of the STICH trial by Farsky et al.^628^ evaluated the effect of OMT for CAD and HFrEF in patients with severe ICM eligible for CABG. At the time of randomization and after 4 months, 58.7% and 73.3% of patients, respectively, were receiving OMT. In a multivariate analysis using a Cox model, the use of OMT at baseline was associated with a lower all-cause mortality (HR 0.78; 95% CI 0.66-0.91; p = 0.04). Regardless of the treatment strategy (OMT vs. CABG), OMT was associated with reduced overall mortality.

The presence of viable myocardium should not be considered a prerequisite for determining the appropriate therapeutic strategy. This is supported by the STICH trial, in which the benefit of surgical revascularization was independent of myocardial viability. Regarding PCI for ICM, the REVIVED-BCIS2 trial did not demonstrate a survival benefit compared with OMT alone, even in patients with extensive viable myocardium.

A recently published meta-analysis^629^ evaluated the four main randomized trials that investigated myocardial viability in ICM: STICH, PARR-2, HEART, and REVIVED-BCIS2. No significant difference in mortality was found between the groups (OR 0.92; 95% CI 0.75-1.14), with similar long-term event rates (34% vs. 36%, respectively).

### 15.6. Summary

- Patients with CAD and a LVEF ≤35% who are eligible for CABG should be promptly referred for surgery.
- The benefit of CABG is observed regardless of the presence of myocardial viability.
- PCI does not reduce mortality or hospitalization due to HF.
- The presence of myocardial viability is associated with a better prognosis and improvement in LVEF, whether treated with OMT alone or in combination with surgery.
- The extent of myocardial fibrosis predicts worse prognosis and a lower likelihood of LV functional recovery.

## 16. Chronic Kidney Disease in Chronic Coronary Syndrome

Patients with CKD represent one of the highest CV risk groups, with mortality rates more than 10 times higher than those in the general population. Among the most frequent CV complications, CAD stands out. Although it is estimated that 30% to 70% of renal patients have angiographic evidence of CAD, these patients are often asymptomatic. CAD is one of the main causes of MACE at all stages of CKD progression and remains relevant even after a successful kidney transplant. For these reasons, any individual with a reduced GFR or proteinuria should be considered at high risk for CAD. There is limited evidence to guide coronary risk stratification in this group, as Framingham equations tend to underestimate risk in renal patients, and CKD is often an exclusion criterion in studies used to develop clinical guidelines. As a result, most recommendations are based on Level C evidence.

This section refers to patients without typical chest pain. Patients with angina should be managed according to the guidelines developed for the general population.^630^ The aim is to provide cardiologists with tools to identify individuals who should undergo further investigation, to discuss the appropriate diagnostic tests, and to highlight some treatment considerations in this high-risk, frequently asymptomatic population that is commonly excluded from randomized trials. In addition to recent data from the literature, we include findings from the KiHeart Cohort,^731^ developed at our center, which includes approximately 3,000 patients with stage 5 CKD followed for up to 25 years. Given the complexity of CKD, a multidisciplinary approach is recommended for both diagnostic and therapeutic decision-making (Tables 41 and 42).

**Table 41:** Diagnostic and therapeutic strategy

Recommendation	Recommendation grade	Level of evidence
It is recommended that diagnostic decision-making and definition of therapeutic strategy be discussed by a Heart-Kidney Team, including a clinical cardiologist, interventional cardiologist, cardiovascular surgeon, and nephrologist.	**I**	**C**

**Table 42:** Clinical risk stratification

Recommendation	Recommendation grade	Level of evidence
Regardless of symptoms and functional status, patients with stages 3 to 5 CKD should be stratified for CAD using clinical criteria (risk factors).	**I**	**A**

CAD: coronary artery disease; CKD: chronic kidney disease.

Clinical stratification is important as it allows for the identification of patients who need to be investigated for CAD. It is effective and low-cost. The American Society of Transplantation^632^ recommends stratifying patients at stage 5 CKD using only three factors: age ≥ 50 years, diabetes, and concomitant CVD. In the general population, other characteristics such as male sex, White race, smoking, and dyslipidemia are significant risk factors for CAD. However, the importance of these factors decreases as CKD progresses, to the point where they are no longer relevant in dialysis patients.

A key practical point of this clinical stratification is that event-free CV survival decreases with the number of the three risk factors (Figure 21).^633^

**Figure 21:** Event-free survival according to the number of risk factors. AMI: acute myocardial infarction; HF: heart failure; MACE: major adverse cardiovascular events..

Font: De Lima, Gowdak & de Paula, 2012.

Patients with none of the three risk factors show a MACE-free survival rate above 90% over 80 months of follow-up. In contrast, survival drops to around 50% in individuals with all three factors (Table 43). CAD: coronary artery disease; CVD: cardiovascular disease.

**Table 43:** Recommendations for Risk Stratification of Coronary Artery Disease Based on Clinical Factors

Recommendation	Recommendation grade	Level of evidence
Individuals without any of the three main risk factors (age ≥50 years, diabetes, CVD) are considered low-risk and do not require in-depth investigation for CAD.	**I**	**C**
Patients with one or more of these important risk factors should undergo CAD risk stratification using both noninvasive and invasive methods.	**I**	**C**

CAD: coronary artery disease; CVD: cardiovascular disease.

### 16.1. Stratification Using Noninvasive Tests

In the general population, current guidelines do not recommend in-depth coronary evaluation in asymptomatic individuals with good FC, even among those undergoing intermediate or high-risk noncardiac surgeries.^630,634^ On the other hand, the NKF/KDOQI guideline,^635^ designed for individuals with CKD, recommends that patients with diabetes and/or those with CAD – even if asymptomatic – should routinely undergo noninvasive tests for ischemia.

Noninvasive tests have reduced accuracy in CKD. The most indicated test is myocardial scintigraphy with pharmacological stress. There are also noninvasive imaging tests that can aid in assessment of risk.^636^ CCTA can be useful in patients with CKD; however, its use is limited by the risk of nephrotoxicity in those not yet on dialysis and by the high degree of coronary calcification, which hinders lumen visualization. Gadolinium-based imaging tests are contraindicated in patients with advanced CKD.

As with other tests of this nature, scintigraphy has limited value in very high-risk individuals, which includes a large proportion of patients with CKD, and is unnecessary when clinical risk is low.

### 16.2. Stratification Using Invasive Tests

Coronary angiography defines vessel anatomy and requires the use of contrast agents. Its use is problematic in individuals who are not yet on dialysis. CAD diagnosed by angiography is strongly associated with prognosis in dialysis patients (Figure 22 and Table 44).

**Figure 22:** Event-free survival from combined cardiovascular events (left) and myocardial infarction (right) in patients with stage 5 CKD undergoing coronary angiography (KiHeart Cohort). CKD: chronic kidney disease.

**Table 44:** Recommendations for Stratification by Invasive Testing

Recommendation	Recommendation grade	Level of evidence
Patients with a positive noninvasive test and those at high clinical risk should be referred for invasive evaluation.	**IIb**	**C**
Asymptomatic patients with obstructive coronary artery disease, regarding the indication for revascularization: they should not be routinely referred for myocardial revascularization solely due to the possibility of undergoing transplantation ("prophylactic intervention"), unless there is unequivocal prognostic benefit from the procedure.	**III**	**C**

### 16.3. Criticism of the Invasive Strategy for Coronary Artery Disease Investigation

Because of the high prevalence of atherosclerosis, the low accuracy of noninvasive tests, and the strong association between CAD and adverse events, patients with a positive test or multiple risk factors should undergo invasive investigation and revascularization if indicated. On the other hand, there is no definitive evidence of the benefit of myocardial revascularization (either percutaneous or surgical) in patients with asymptomatic CAD and CKD.^637^ Indeed, results from the recent ISCHEMIA trial^638^ suggest that an in-depth evaluation of coronary disease in advanced CKD does not alter prognosis. The study assessed whether an invasive strategy – which included coronary angiography and intervention if necessary – offered benefit compared to OMT in patients with stage 4 or 5 CKD and documented moderate or extensive ischemia. After 2.2 years of follow-up, there was no difference in MACE between the groups. However, after 5.7 years, the invasive strategy was associated with a significant reduction in CV mortality, which was offset by an increase in nonCV mortality. Therefore, routine investigation of CAD in advanced CKD remains undefined, and the ISCHEMIA results still need to be confirmed. Finally, the possibility of future kidney transplantation alone should not influence the choice of treatment for CAD – whether interventional or pharmacological.^639^

In summary, the invasive strategy is useful to assess CV risk but does not necessarily improve prognosis.

### 16.4. Medication and Dose Adjustment in Patients with Cardiovascular Disease and Chronic Kidney Disease

CKD carries a CV risk level comparable to that of acute MI, justifying the implementation of secondary prevention.^640,641^ This includes prescribing statins, ASA, BBs, and ACEi/ARBs for all patients, unless contraindicated.

CKD affects the absorption, bioavailability, metabolism, and elimination of many drugs. Inappropriate use or dosage errors contribute to increased toxicity and reduced therapeutic efficacy. Chart 3 presents commonly used medications for patients with CVDs and nondialysis CKD, along with recommended doses adjusted for GFR.

ACEi/ARBs are first-line agents for treating hypertension and proteinuria, and they slow CKD progression. An increase in serum creatinine of up to 15% is expected and not a reason for dose reduction. Discontinue the drug if creatinine increases by > 30% from baseline. Hydrophilic BBs (e.g., atenolol, bisoprolol, nadolol) are eliminated by the kidneys and require dose adjustment. Lipophilic BBs (e.g., propranolol, metoprolol) are metabolized in the liver and can be used at full dose. Hydrochlorothiazide is ineffective in advanced CKD. Spironolactone should be avoided due to the risk of hyperkalemia. Metformin is contraindicated in CKD stage ≥3 due to the risk of lactic acidosis. Gliclazide is the preferred sulfonylurea due to its shorter half-life and lack of active metabolites. Others should be used with caution. SGLT2i (e.g., dapagliflozin) are generally considered safe, but data are insufficient for patients with GFR < 30 mL/min. Statins do not require dose adjustment, but their role in primary prevention for dialysis patients is controversial.^642–944^ The KDIGO Organization^645^ does not recommend routine statin use in asymptomatic dialysis patients without CAD. The ACC leaves statin indication undefined.^646^ ASA has uncertain benefit for primary prevention in renal patients.^647^ Warfarin and apixaban are the OACs recommended for patients with renal dysfunction.^648^

For patients with stage 3 to 5 CKD, the recommendations below are indicated (Table 45).

**Table 45:** Recommendations for Medication and Dose Adjustment in Patients with CVD and CKD

Recommendation	Recommendation grade	Level of evidence
It is recommended that diagnostic decision-making and therapeutic strategy planning be discussed by a "Heart-Kidney Team" including a clinical cardiologist, interventional cardiologist, CV surgeon, and nephrologist.	**I**	**C**
Patients with stage 3 to 5 CKD should be evaluated for the presence and severity of CAD based on clinical history, physical examination, and routine tests.	**I**	**A**
Patients without the main risk factors* are considered to be at low CV risk and do not require further investigation. Patients with one or more risk factors should undergo risk stratification for CAD using noninvasive and invasive methods.	**I**	**C**
Patients with a positive noninvasive test and those with multiple risk factors are considered at high coronary risk and should be referred for invasive evaluation.	**IIb**	**C**
Patients with obstructive CAD involving the proximal segments of the main epicardial coronary arteries are indicated for surgical or percutaneous myocardial revascularization aiming to reduce CV risk.	**IIb**	**C**
Asymptomatic patients with obstructive CAD should not be referred for routine myocardial revascularization based solely on the possibility of undergoing transplantation ("prophylactic intervention"), unless there is unequivocal prognostic benefit from the intervention.	**III**	**C**
Patients with stage 3 to 5 CKD should be treated for secondary prevention of events.	**IIa**	**C**

CAD: coronary artery disease; CKD: chronic kidney disease; CV: cardiovascular.

* Age ≥50 years, diabetes, and evidence of CV disease.

## 17. Cardio-Oncological Aspects in Chronic Coronary Syndrome

Oncology challenges medicine by encompassing patients at different stages of the same disease and by requiring rapid decision-making. For these reasons, patients with active neoplasia are commonly excluded from large clinical trials. Moreover, the coexistence of CVD in cancer patients is increasingly frequent, as they share common risk factors.^649,650^ Endothelial dysfunction, oxidative stress, hypercoagulability, and chronic inflammation are common in this patient profile, and the treatments administered often contribute to the development of CVD.^649–651^ he occurrence of CAD in cancer patients is a reality and is steadily increasing,^649,652^ both among those with preexisting CAD who are later diagnosed with cancer and those who face CV effects resulting from thoracic radiotherapy and chemotherapy.^649,561,652^ Neoplasms independently increase the risk of CV death and major bleeding.^650^

Therapeutic management of patients with CAD includes decisions regarding coronary intervention and antiplatelet therapy.^7653,654^ In oncology patients with CAD, several variables must be considered before making such decisions (Table 46).^653^ The high risk of bleeding in some scenarios has made clinical management and decision-making even more complex.^655^ Table 47 presents some factors that may increase bleeding risk.

**Table 46:** Variables for decision-making

**Need for early cancer surgery**
**Increased risk of bleeding**
**Laboratory abnormalities such as renal dysfunction, anemia, and coagulopathies**
**Life expectancy**

**Table 47:** Factors that increase bleeding risk

**Tumor site** Example: gastrointestinal tract tumors, prostate
**Surgical site** Example: neurosurgery, transurethral resection of the prostate
**Blood dyscrasia due to tumor type or disease extent** Example: thrombocytopenia, liver involvement

Scientific evidence helps guide and support the management of these patients (Table 48).

**Table 48:** Cardio-Oncology Recommendations for Interventional Therapy

Recommendation	Recommendation grade	Level of evidence
The decision for intervention should be based on tumor type, life expectancy, bleeding risk, and urgency of the surgical procedure.^652^	**I**	**C**
When indicating an intervention in frail patients, the procedure should be as minimally invasive as possible.^652^	**I**	**C**
The decision for intervention should be shared between the cardiologist and the oncologist.	**I**	**C**
A drug-eluting stent should be preferred in oncology patients.^651^	**IIa**	**B**
DAPT may be maintained in patients with platelet count > 30,000 if there are no contraindications.^651^	**IIa**	**B**

DAPT: dual antiplatelet therapy.

There is a clear increase in PCI among patients with active cancer and even more so in those with a prior history of the disease.^650^ Oncology patients show higher rates of acute MI, stent thrombosis, bleeding, and need for repeat revascularization compared to other patients.^656^ According to Guo et al., cancer patients at higher risk of thrombotic and ischemic events after PCI can be identified by high DAPT scores.^656^

In a study of more than 6 million patients who underwent PCI in the United States, Potts et al. identified the four most common cancers as prostate, breast, colon, and lung.^650^ In that publication, lung cancer was associated with a higher risk of in-hospital complications, including a twofold increase in mortality. Additionally, a current diagnosis of colon cancer was independently associated with a higher bleeding risk.^650^ Active prostate cancer was associated only with a higher bleeding risk, while breast cancer showed no significant association with any in-hospital complications. The presence of metastasis, regardless of cancer type, also impacted rates of in-hospital mortality and complications.^650^ Except for lung cancer, a prior history of neoplasia did not influence the risk of adverse events in patients undergoing PCI.^650^ Notably, major bleeding events and in-hospital mortality were higher in patients who received bare-metal stents compared to those who received drug-eluting stents.^560^

With the advancement of technology and greater access to drug-eluting stents, the duration of antiplatelet therapy has been considerably reduced in recent years.^653^ Until 2015, PCI with bare-metal stents was recommended for patients at high bleeding risk, as this was the safest strategy to minimize the duration of DAPT, especially for those awaiting noncardiac surgery.^653^ However, studies with new stent platforms have shown that it is possible to reduce bleeding risk without compromising short-term safety, even with shorter DAPT durations in some scenarios.^657^ The MASTER DAPT (Management of High Bleeding Risk Patients Post Bioresorbable Polymer Coated Stent Implantation With an Abbreviated Versus Standard DAPT Regimen) study compared 30-day versus 90-day DAPT after implantation of a sirolimus-eluting stent. Among the more than 4,000 patients studied, the shorter treatment duration was noninferior to standard therapy.^657^ Similar results were observed in the GLOBAL LEADERS trial^658^ and the STOPDAPT 2 (Short and Optimal Duration of Dual Antiplatelet Therapy After Everolimus-Eluting Cobalt-Chromium Stent-2) trial,^659^ in patients with low bleeding risk.

The publication of ZEUS in 2016 demonstrated that drug-eluting stents were superior to bare-metal stents in reducing outcomes with just 30 days of DAPT in patients at high bleeding risk.^660^ A year earlier, the LEADERS FREE (Polymer-free Drug-Coated Coronary Stents in Patients at High Bleeding Risk) trial had already introduced this new concept. With nearly 2,500 randomized patients, this trial showed that the polymer-free stent (BioFreedom) was superior to the bare-metal stent in this population.^661^ The SENIOR (Short Duration of Dual antiplatElet Therapy With SyNergy II Stent in Patients Older Than 75 Years Undergoing Percutaneous Coronary Revascularization) trial demonstrated that shortening DAPT duration in older patients undergoing PCI with bioresorbable stents was a better option compared to conventional stent implantation with prolonged DAPT.^662^

More recently, the ONYX ONE (A Randomized Controlled Trial With Resolute Onyx in One Month Dual Antiplatelet Therapy [DAPT] for High-Bleeding Risk Patients) trial was published – the first study comparing a zotarolimus-eluting stent with a polymer-free stent using a 30-day DAPT strategy in high bleeding risk patients.^663^ Nearly 2,000 patients were evaluated, and the drug-eluting stent was shown to be noninferior to the polymer-free stent in terms of safety and efficacy at 1-year follow-up.^663^ Safer and more effective stents, combined with intravascular imaging, provide a more stable setting for decision-making in the approximately 30% of patients with high bleeding risk who undergo PCI.^663^

The occurrence of ischemic and bleeding events in patients with cancer was compared to that of other high bleeding risk patients. Campos et al. analyzed for the first time data from four large studies that included drug-eluting stents, short-term DAPT, and high bleeding risk patients. Around 10% of the population analyzed had cancer and showed higher rates of bleeding and all-cause mortality. However, there was no increased risk of MI, stent thrombosis, or repeat revascularization. Interestingly, the highest bleeding rate in cancer patients occurred after discontinuation of DAPT.^415^

Accepting the challenge of treating oncology patients involves the effort to minimize bleeding risk while maintaining a low rate of thrombosis. The long-term risks and benefits, as well as the evaluation of different types of drug-eluting stents and the application of risk scores, still need to be better clarified in this population. However, based on clinical judgment and individualized case assessment, it appears to be safe to use short-term DAPT after the implantation of either drug-eluting or polymer-free stents in patients at high bleeding risk, including those with an active cancer diagnosis.^661,663,664^

## 18. Treatment of Hypertension in Patients with Chronic Coronary Artery Disease

Hypertension is the main modifiable risk factor for CAD.^1,80,507^ In the INTERHEART study,^665^ 25% of acute MI were attributed to hypertension. A meta-analysis evaluating the impact of BP reduction on CV risk showed that for every 10 mm Hg decrease in systolic BP, there was an associated 17% reduction in CAD.^666^

The treatment of hypertension associated with CAD includes patients post-acute MI, with angina pectoris, and postmyocardial revascularization. These individuals are therefore classified as hypertensive patients at high CV risk.^507^ Thus, the target BP should take into account the possibility of the J-curve effect, which has been demonstrated in different studies.^338,667^ he goal is to achieve a systolic BP < 130 mm Hg and diastolic BP < 80 mm Hg (Recommendation Grade: IIa; Level of Evidence: B), while avoiding levels below 120/70 mm Hg.^1,80,507^

The recommended treatment for hypertensive patients with CAD includes lifestyle changes and immediate pharmacological therapy with a combination of two drugs. Preferably, this should include RAAS inhibitors (RAASi) – ACEi or ARBs – in combination with BBs or long-acting CCBs.^1,80,507^ Particularly in the post-MI setting, the combination of RAASi and BBs offers benefits in BP reduction, anti-anginal effects, and reductions in events and mortality, especially within two years after the acute event.^668,669^ In patients with symptomatic angina, BBs and CCBs can both be selected for combined drug therapy due to their effectiveness in symptom reduction.^1,80,507^

If the BP goal is not reached with dual therapy, a triple combination should be used, adding a thiazide diuretic (preferably long-acting). If a fourth medication is needed, a mineralocorticoid receptor antagonist (e.g., spironolactone) is the drug of choice, followed by alpha-2 adrenergic agonists (Table 49).^1,80,507^

**Table 49:** Summary of recommendations for antihypertensive treatment in patients with chronic CAD^1,80,507^

Recommendation	Recommendation grade	Level of evidence
In adults with chronic CAD, nonpharmacological strategies are recommended as first-line therapy to reduce BP at all stages of hypertension and levels of CV risk.	**I**	**A**
The BP targets to be achieved in individuals with chronic CAD are: Adults: systolic BP 120-129 mm Hg / diastolic BP 70-79 mm Hg; Healthy older adults: systolic BP 130-139 mm Hg / diastolic BP 70-79 mm Hg; Frail older adults: systolic BP 140-149 mm Hg / diastolic BP 70-79 mm Hg.	**I**	**A**
Hypertensive patients with a history of recent acute MI (<12 months) are indicated for the use of BBs and RAASi as first-line treatment.	**I**	**A**
In patients with anginal symptoms, BBs and/or long-acting dihydropyridine CCBs are recommended therapeutic classes.	**I**	**A**
Other additional antihypertensive medications such as long-acting dihydropyridine CCBs, long-acting thiazide diuretics, mineralocorticoid receptor antagonists, and/or alpha-2 adrenergic agonists may be added as needed to achieve BP targets.	**I**	**A**
Concomitant use of ACEIs and ARBs is contraindicated as it provides no benefit and increases the risk of adverse events.	**III**	**A**

## 19. Treatment Considerations in the Presence of Atrial Fibrillation

### 19.1. New Oral Anticoagulants Associated with Acetylsalicylic Acid

In this high ischemic burden scenario, large-scale clinical trials have evaluated the combination of other drugs acting on the coagulation cascade with antiplatelet agents, especially ASA. In the COMPASS trial,^670^ eligible patients with PAD, carotid disease, or CAD, without previous indication for anticoagulation, were randomized to receive low-dose oral rivaroxaban (2.5 mg twice daily – one-fourth of the full anticoagulation dose) plus ASA (100 mg), rivaroxaban 5 mg twice daily (with ASA placebo), or ASA once daily. Low-dose rivaroxaban reduced MACE and severe limb adverse events compared to ASA, at the cost of increased major bleeding. The predefined significance thresholds for mortality were not reached for the entire population. However, ARRs were observed in higher-risk individuals such as patients with diabetes, PAD, CKD, and active smokers.

This benefit of rivaroxaban and ASA combination therapy in higher-risk patients was further assessed in a COMPASS subanalysis, which showed a significant improvement in QoL, measured by the 6-minute walk test, in those receiving dual therapy compared to ASA alone, without hemorrhagic events.^671^ Thus, the recommendation for adding a second anti-ischemic drug, including low-dose rivaroxaban, for patients with CCS and high ischemic burden, is considered appropriate, while factoring in individual bleeding risk in decision-making.

### 19.2. Anticoagulation in Patients with Chronic Coronary Syndrome and Atrial Fibrillation

Overall, full anticoagulation is recommended for patients with AF and CCS to reduce embolic events, especially ischemic stroke, with documented superiority over ASA monotherapy or DAPT based on clopidogrel.^672^ When prescribing a novel OAC (NOAC) – such as apixaban, dabigatran, edoxaban, or rivaroxaban – rather than a vitamin K antagonist (VKA), NOACs are preferred, especially in terms of safety and reduced hemorrhagic events.^672,673^

For patients with elective indication for PCI, no large clinical trials have specifically evaluated those with CCS and AF. Therefore, decisions should be based on studies that included a large proportion or exclusively patients with ACS. The AUGUSTUS trial evaluated patients with AF and recent ACS or PCI, and indication for a P2Y12 inhibitor (P2Y12i). Patients were randomized to receive apixaban (5 mg twice daily) or a VKA, along with ASA or matching placebo for 6 months. The apixaban regimen without ASA resulted in fewer bleeding events and hospitalizations, with no significant difference in ischemic events compared to the VKA, ASA, or both.^210^ These results were consistent in the more severe subgroup – patients with prior cerebrovascular events.^674^

These findings were confirmed in a network meta-analysis including five studies and over 11,000 patients (most post-ACS or PCI) with AF undergoing PCI: a regimen with NOAC plus a P2Y12i was associated with a 48% reduction in bleeding complications, including intracranial hemorrhage, without significant difference in ischemic events compared to VKA plus DAPT.^675^

Thus, in patients with AF and recent ACS or PCI, the preferred treatment is a full-dose NOAC for stroke prevention plus a P2Y12i without ASA. Regimens with VKA plus DAPT (P2Y12i and ASA) should generally be avoided and limited to the first month in patients with very high thrombotic risk who cannot receive NOACs. If the risk of stent thrombosis or other ischemic events is high, using ASA for 30 days appears reasonable. In the chronic phase, after 12 months, OAC alone should be the standard approach (Tables 50 and 51).^1,675^

**Table 50:** Antithrombotic therapy in patients with chronic coronary syndrome and atrial fibrillation

Recommendation	Recommendation grade	Level of evidence
When there is an indication for oral anticoagulation in a patient with AF eligible for a NOAC, the NOAC should preferably be recommended instead of a VKA.	**I**	**A**
Long-term oral anticoagulation (NOAC or VKA) is recommended for patients with AF and a CHA2DS2-VASc score ≥2 in men and ≥3 in women.	**I**	**A**
Long-term oral anticoagulation (NOAC or VKA) may be considered for patients with AF and a CHA2DS2-VASc score ≥1 in men and ≥2 in women.	**IIa**	**B**

AF: atrial fibrillation; NOAC: novel oral anticoagulant; VKA: vitamin K antagonist.

**Table 51:** Antithrombotic therapy in patients after percutaneous coronary intervention and with atrial fibrillation or other indication for anticoagulation

Recommendation	Recommendation grade	Level of evidence
ASA and clopidogrel should be administered before the procedure in patients electively undergoing coronary stent implantation.	**I**	**C**
In patients with an indication for anticoagulation with a NOAC, it is recommended that the medication (dabigatran 150 mg twice daily, rivaroxaban 20 mg once daily, apixaban 5 mg twice daily, or edoxaban 60 mg once daily) be used preferably over a VKA in combination with antiplatelet therapy.	**I**	**A**
Adjusted doses of rivaroxaban, dabigatran, edoxaban, or apixaban may be used in accordance with current label recommendations, strictly based on clinical criteria defined in the label, and in combination with single or dual antiplatelet therapy.	**IIa**	**B**
After PCI without complications or stent thrombosis risk factors, early discontinuation (<1 week) of ASA and continuation of dual therapy with OAC and clopidogrel should be considered in individuals with low stent thrombosis risk or if bleeding risk outweighs stent thrombosis risk, regardless of stent type.	**IIa**	**B**
Triple therapy with ASA, clopidogrel, and OAC for 1 month should be considered when stent thrombosis risk outweighs bleeding risk.	**IIa**	**C**
Dual therapy with OAC and ticagrelor may be considered as an alternative to triple therapy with OAC, ASA, and clopidogrel in patients at moderate to high stent thrombosis risk, regardless of stent type, or in patients with known clopidogrel resistance.	**IIa**	**B**
Ticagrelor or prasugrel are not recommended as part of triple antithrombotic therapy with ASA and any OAC.	**IIb**	**C**
Dual or triple therapy with OAC and prasugrel, with or without ASA, is not recommended as an alternative to triple therapy with OAC, ASA, and clopidogrel in patients at moderate to high stent thrombosis risk, regardless of stent type.	**III**	**A**

ASA: acetylsalicylic acid; NOAC: novel oral anticoagulant; OAC: oral anticoagulant; PCI: percutaneous coronary intervention; VKA: vitamin K antagonist.

## 20. Secondary Prevention after Acute Coronary Syndrome

Individuals with a history of previous MACE have a 5-fold higher risk of recurrence compared to those without known CVD. Furthermore, patients with clinically established CAD are under very high risk of recurrent MACE. After an episode of ACS, adherence to antithrombotic therapy and other pharmacological treatments (e.g., RAAS blockers, BBs, and statins) is a priority.^1,676^

After hospital discharge following an ACS episode, patients should ideally be directed to care pathways appropriate to their individual risk level, ensuring proper management. Nonpharmacological measures focusing on lifestyle habits and rehabilitation should be prioritized in the immediate post-event phase, with investment in patient adherence to secondary prevention programs, for which shared management protocols during the post-acute phase are indispensable.^676^

### 20.1. Priorities in the Postdischarge Period after Acute Coronary Syndrome

Discharge is a key milestone in the management of patients with ACS. At this stage, primary health care professionals play an important role in post-ACS educational counseling as part of the overall risk factor control strategy. Counseling strategies should be applied to post-ACS patients to ensure positive impacts on health outcomes and QoL for individuals and their families.

In summary, a multidisciplinary team should develop customized discharge protocols for each patient. These protocols should cover hospitalization, discharge medications, and actions to be taken in case of clinical changes. This plan should coordinate follow-up care planning and contact channels with the care provider.^1,676^ Health education, cardiac rehabilitation, and timely clinical follow-up are essential components of the discharge protocol.^676^

### 20.2. Risk Stratification after Acute Coronary Syndrome for Secondary Prevention Planning

Tools for stratifying long-term risk of MACE include validated risk scores such as SMART (Secondary Manifestations of Arterial Disease) and the EUROASPIRE (European Action on Secondary and Primary Prevention by Intervention to Reduce Events) risk model.^677,678^

The SMART score, developed in the Netherlands in a population of 5,788 individuals with established CVDs and externally validated in three international cohorts, estimates the 10-year risk of MI, stroke, or CV death. The variables included in the SMART risk model are age, sex, current smoking, diabetes, BP, cholesterol, CAD, cerebrovascular disease, PAD, creatinine, and hs-CRP values.^677^

The EUROASPIRE calculator – derived from a random population of 8,000 individuals with a median follow-up of 1.7 years and externally validated in 4,484 patients – estimates the 2-year risk of recurrent MACE in patients with stable CAD.^678^ The calculator assesses the association between risk factors and the incidence of the score's primary endpoint, which includes fatal MACE or new hospitalizations for nonfatal MI, stroke, HF, CABG, or PCI.^678^

Other risk scores, such as GRACE and ProACS, have shown similar accuracy and may be applied.^679^ Therefore, risk stratification for MACE after an episode of ACS using validated scores should be conducted for all patients admitted with this condition, as early as possible during hospitalization.^676^

### 20.3. Risk Variables for Heart Failure and Thrombotic Events

LV dysfunction and the presence of signs or symptoms of HF are the most appropriate variables for prognostic stratification and for designing specific postdischarge care pathways following ACS.^680^ The development of HF during hospitalization or soon after discharge are important predictors of mortality during follow-up.

The clinical variables associated with high risk of HF and/or LV dysfunction after an episode of ACS, based on studies involving these populations, are as follows:^676^

1. Killip class during the event;
2. LVEF <40%;
3. LVEF between 40% and 45% associated with restrictive diastolic filling pattern, significant mitral regurgitation, high wall motion score index, and nondilated LV;
4. Significant variation in B-type natriuretic peptide;
5. Need for loop diuretics.

Literature reviews have shown that cardiac rehabilitation is associated with significant improvement in outcomes for this subpopulation, including reduced acute MI risk, slight reduction in all-cause mortality, and a significant reduction in all-cause hospitalizations, resulting in significantly lower health care costs and improved QoL within 12 months of follow-up.^681,682^ In the long term, there is a trend toward lower CV mortality and acute MI rates.^682^ Despite the evidence of these benefits, international registries such as EUROASPIRE V estimate participation rates in such rehabilitation programs below 35%.^678^

Similarly, patients with high ischemic risk based on clinical and angiographic variables have high recurrence rates of MACE, especially thrombotic events. The prior occurrence of an episode of ACS alone places the individual in this high-risk category. For these patients, outpatient follow-up and cardiac rehabilitation should be provided through frequent visits and appointments, especially during the first year after the event, and through programs tailored to the clinical condition and associated risk variables.^681–683^

These characteristics are associated with a high thrombotic risk based on data from studies involving individuals with documented CAD and should be considered when defining more aggressive secondary prevention strategies:^666,676,784,688–694^

- PAD;
- History of angina or previous acute MI;
- Multivessel CAD;
- Incomplete revascularization;
- Patients not revascularized in prior procedures.

### 20.4. Strategies for Low-Risk Patients

Patients without HF and/or LV dysfunction and without high thrombotic risk factors may be referred to lower-intensity care pathways and rehabilitation programs, with a single specialized cardiac consultation within the first year and subsequent referral to primary health care. Secondary prevention recommendations should also be clearly detailed in the discharge report through dedicated counseling, with the possibility of remote follow-up. Therefore, a systematic assessment of the risk of HF/LV dysfunction and thrombotic events should be provided for all post-ACS patients.^676,679^

### 20.5. Clinical Follow-Up

After myocardial revascularization and/or stabilization following an ACS (<1 year), patients should be closely monitored due to the risk of recurrent ischemic events. Current evidence on the management of CCS recommends reassessing LV function 8 to 12 weeks after a revascularization procedure to evaluate the recovery of myocardial function (stunning or hibernation) or potential deterioration due to CVD or other comorbidities.^1,676^ 1 year after revascularization, even in asymptomatic patients, an annual assessment is recommended, including a 12-lead ECG to evaluate clinical status, medication adherence, and achievement of CV risk factor targets.

A noninvasive stress test to detect silent ischemia and TTE can be performed every 3 to 5 years.^695^ Laboratory tests (e.g., lipid profile, renal function, complete blood count, glycemic profile, biomarkers) should be reassessed periodically based on goals and previous results.^676,695^ Remote follow-up via telemedicine, when feasible, has also proven effective in improving physician-patient interaction, reducing outcomes such as rehospitalization, emergency visits, unplanned revascularization, and symptom control.^696^

## 21. Postpercutaneous Coronary Intervention Care in Chronic Coronary Syndrome

### 21.1. Antiplatelet Therapy after Percutaneous Coronary Intervention with Stent Placement in Patients without an Indication for Anticoagulants

DAPT with ASA and a P2Y12i is the standard treatment after stent implantation to reduce the risk of thrombosis and ischemic events.^697–700^ ASA is the preferred antiplatelet agent and should be prescribed after stent implantation, except in cases of intolerance or adverse events.^1,700,701^ Clopidogrel is the P2Y12i of choice for patients undergoing stent implantation in the setting of CCS.^702,703^ The ALPHEUS (Assessment of Loading With the P2Y12 Inhibitor Ticagrelor or Clopidogrel to Halt Ischemic Events in Patients Undergoing Elective Coronary Stenting) randomized trial evaluated whether ticagrelor was superior to clopidogrel after PCI of greater complexity in patients with stable CAD. A total of 1,910 patients were randomized, and no difference was found in the primary outcome, defined as the composite of type 4a or 4b MI related to PCI or major myocardial injury. In the ticagrelor group, a higher rate of minor bleeding was observed at 30 days.^704^

The optimal duration of DAPT in stable CAD with drug-eluting stents has been progressively shortened over the years. Several studies have shown that 6 months of DAPT, when compared with 12 months, is effective and safe.^1,657,700–714^ However, a shorter duration of DAPT – 3 months – may be considered in patients with high bleeding risk.^1,657,700,701,710–714^ Some studies have assessed the use of P2Y12 monotherapy after a short period of DAPT.^715^

The Japanese STOP DAPT-2 study evaluated the use of 1-month DAPT followed by clopidogrel monotherapy in patients receiving cobalt-chromium everolimus-eluting stents, showing a reduction in bleeding events without a significant increase in ischemic events.^658^ TWILIGHT randomized 7,119 patients who underwent PCI with stenting. After 3 months of DAPT (35% in the CCS setting), ticagrelor alone resulted in a lower incidence of clinically relevant bleeding compared to ticagrelor plus ASA, with no increased risk of death, MI, or stroke.^208^

The use of DAPT for more than 6 months, extending up to 30 months, may be considered in patients with high ischemic risk and low bleeding risk.^716–718^ The DAPT score, which can support this decision, was derived from a cohort in the DAPT Study (Dual Antiplatelet Therapy Study). Patients with a high-risk score (≥2) showed a reduction in ischemic outcomes at the cost of a slight increase in bleeding risk when treated with DAPT for 30 months. In contrast, patients with a low score (<2) did not demonstrate a reduction in ischemic events.^414^

The COMPASS trial (Cardiovascular Outcomes for People Using Anticoagulation Strategies)^204,719^ demonstrated that dual pathway inhibition (DPI) using rivaroxaban 2.5 mg twice daily combined with ASA 100 mg once daily, compared with ASA 100 mg alone, reduced the primary outcome of MACE, which included CV death, MI, or stroke, as well as overall mortality in patients with CCS and/or PAD. In a subgroup analysis of COMPASS-PCI, outcomes were assessed in patients with CCS with or without prior PCI, treated with DPI versus ASA alone. Among the 27,395 patients in COMPASS, 16,560 had CCS, of which 9,862 (59.6%) had undergone prior PCI. The mean time from PCI to randomization was 5.4 years, with a mean follow-up of 1.98 years. In patients with prior PCI, DPI compared with ASA alone consistently reduced MACE regardless of time since PCI, though at the cost of increased bleeding (Figure 23).^720^

**Figure 23:** Antiplatelet therapy after PCI in chronic coronary syndrome. AAS: acetylsalicylic acid; DAPT: dual antiplatelet therapy; PCI: percutaneous coronary intervention.

### 21.2. Antiplatelet Therapy after Percutaneous Coronary Intervention with Stent in Patients with Indication for Anticoagulation

Many patients undergoing PCI with stenting in the context of CCS have AF with an indication for long-term OAC. The combination of OAC and DAPT significantly increases the risk of bleeding.^721–723^ This scenario has been addressed in several clinical trials aiming to define the best antithrombotic combination and its optimal duration. Randomized trials have demonstrated that DOACs are safer than VKAs in the setting of PCI and therefore are the preferred anticoagulants (Figure 24).^724–726^

**Figure 24:** Antiplatelet therapy after PCI in patients on OAC. DAPT: dual antiplatelet therapy; OAC: oral anticoagulation; PCI: percutaneous coronary intervention.

The first randomized trial to assess the use of a DOAC after PCI with stent was the PIONEER AF-PCI. The use of low-dose rivaroxaban in combination with a P2Y12i for 12 months was associated with a lower rate of clinically significant bleeding compared to the standard therapy with a VKA, with no differences in ischemic outcomes.^227^ The RE-DUAL PCI trial also demonstrated the benefits of dabigatran in this setting.^728^

The AUGUSTUS trial was pivotal in clarifying the role of ASA after PCI in patients on OAC. It randomized 4,614 patients across 33 countries in a 2×2 factorial design. Patients with AF in the context of ACS and/or undergoing PCI with stent were included. They were randomized to receive apixaban or a VKA and again to receive ASA or placebo in combination with a P2Y12i. Apixaban was associated with lower bleeding risk compared to warfarin. The use of ASA, compared to placebo, was associated with increased bleeding without significant differences in ischemic events.^210^

The ENTRUST-AF PCI was a multicenter, randomized trial including patients with AF on OAC undergoing PCI for stable CAD or ACS. Patients were randomized to receive dual therapy with edoxaban and clopidogrel or traditional triple therapy with warfarin plus DAPT (ASA and clopidogrel). Edoxaban-based therapy was noninferior in terms of bleeding compared to VKA-based regimens.^728^

DAPT with ASA and a P2Y12i, preferably clopidogrel, should be administered to all patients during PCI and maintained until hospital discharge, potentially extended up to 1 month depending on thrombotic risk. Dual therapy with clopidogrel and a DOAC should then be continued for 6 to 12 months depending on the balance between ischemic and bleeding risks. After this period, patients should discontinue antiplatelet therapy and continue with OAC alone.^730–732^

## 22. Postoperative Care after Coronary Artery Bypass Grafting – Routine Monitoring and Management

CABG restores distal coronary blood flow by bypassing an obstructed segment of a coronary artery. However, it does not modify the underlying atherosclerotic disease. Therefore, both pharmacological and nonpharmacological secondary prevention measures remain necessary in the postoperative setting.

1. We recommend that secondary prevention measures, including pharmacological and nonpharmacological strategies, be resumed after CABG.
  Cardiac rehabilitation, for instance, has been associated with a reduction in CV outcomes in the postoperative period and should be recommended to reduce CV mortality, MI, and hospitalizations.^490^
2. We recommend that patients undergo a cardiac rehabilitation program after CABG to reduce the risk of subsequent MACE.
  A key aspect of pharmacological therapy is antithrombotic strategy. Early reintroduction of ASA has been consistently associated with greater venous graft patency and fewer MACE,^733^ and is recommended for all patients. In some clinical scenarios, DAPT after surgery has proven useful,^734^ such as in surgeries performed for ACS^734^ or off-pump procedures, to maintain venous graft patency.^736,737^
3. We recommend initiating ASA (100-325 mg) 6 hours after surgery and maintaining its use indefinitely to reduce venous graft occlusion and MACE.
4. In selected patients without high bleeding risk, DAPT with clopidogrel or ticagrelor may be considered for up to 1 year after surgery to reduce graft occlusion.
  Regarding statins and RAASi, there is no consistent evidence supporting their early reintroduction.^737,738^ However, their long-term benefits justify resumption before hospital discharge or as soon as tolerated.
  Regarding BBs, despite conflicting results on prognostic benefits of early initiation in elective surgeries, their potential to reduce the incidence of postoperative AF (POAF) supports their early use once tolerated.^739^
5. We recommend initiating BBs as soon as the patient can tolerate them to reduce the incidence of POAF.
  CCBs have also been shown to be useful not only in reducing occlusion of spastic arterial grafts, such as radial artery grafts, but also in decreasing MACE in patients receiving these types of grafts.^749^
6. We recommend early initiation of CCBs in patients undergoing CABG with use of radial artery grafts, and continuing for at least 1 year to reduce graft occlusion and MACE.
  The use of antiarrhythmic drugs and full anticoagulation may also be necessary in the context of POAF. POAF is the most frequent arrhythmia after CABG, occurring in about 20% of cases.^741^ Chemical or electrical cardioversion or rate control are all possible strategies, as over 90% of patients, regardless of strategy, return to sinus rhythm within 60 days postsurgery.^742^ Despite conflicting evidence regarding anticoagulation in all cases,^741^ it is indicated for POAF episodes that are sustained or revert after lasting longer than 24 hours, and should be continued for at least 4 weeks. Besides warfarin, DOACs appear to be safe and cost-effective in this population.^743^
7. We recommend full anticoagulation (with warfarin or DOAC) after CABG in cases of POAF that reverts or lasts for at least 24 hours, and it should be maintained for a minimum of 4 weeks.
  The long-term follow-up of patients undergoing CABG aims not only to monitor potential procedure-related complications, but also to ensure adherence to pharmacological therapy and to identify any MACE. There is no consensus in the literature regarding the routine use of CCS re-stratification in these cases. In patients with recurrent symptoms or reduced ventricular function during follow-up, functional imaging tests are generally recommended as the preferred method.^744^ CCTA has also proven useful in detecting graft occlusion.^745^ In asymptomatic patients, particularly those in higher-risk populations, functional or anatomical re-stratification may be considered 5 years after the procedure (Table 52).^746^
8. Imaging-based re-stratification is recommended for patients after CABG who experience recurrence of symptoms or have a loss of ventricular function.
9. In populations under high CV risk, imaging-based re-stratification may be considered in asymptomatic patients 5 years after surgery.

**Table 52:** condary prevention after coronary artery bypass grafting

Therapy/action	Recommendation	Recommendation grade	Level of evidence
General measures	Reintroduction of optimal medical therapy	**I**	**A**
	Referral to a rehabilitation program	**I**	**A**
Antithrombotic strategy	Reintroduction of ASA within 6 hours and continued indefinitely	**I**	**A**
	DAPT with ticagrelor or clopidogrel in selected populations with low bleeding risk	**IIb**	**B**
	Use of warfarin or DOAC for at least 4 weeks in cases of POAF (reverted or lasting at least 24 hours)	**IIa**	**B**
ACEi/ARB/ARNI	Reintroduction as soon as tolerated	**I**	**C**
Statins	Reintroduction as soon as tolerated	**I**	**C**
BBs	Early reintroduction to reduce incidence of POAF	**I**	**B**
CCBs	Early initiation in patients receiving radial artery grafts, to be maintained for at least 1 year	**I**	**B**
Follow-up re-stratification	Imaging exams (functional or anatomical) for patients with recurrent symptoms or loss of ventricular function	**I**	**B**
	Re-stratification after 5 years in asymptomatic patients under high risk of events	**IIb**	**C**

ACEi: angiotensin-converting enzyme inhibitor; ARB: angiotensin receptor blocker; ARNI: angiotensin receptor neprilysin inhibitor; ASA: acetylsalicylic acid; CCB: calcium channel blocker; DAPT: dual antiplatelet therapy; DOAC: direct oral anticoagulant; POAF: postoperative atrial fibrillation.
