# Supplementary material for: Errata: Diretriz de Síndrome Coronariana Crônica – 2025
Source: Arq Bras Cardiol. 2026 Jul 28;123(7):e20260565. [Article in Portuguese] doi: 10.36660/abc.20260565 (PMC13671145; doi:10.36660/abc.20260565)
Supplement: Translation

# Translation: Erratum: Guideline for Chronic Coronary Syndrome – 2025

**DOI:** 10.36660/abc.20260565i

In the article Cesar LAM, et al. Guideline for Chronic Coronary Syndrome – 2025. Arq Bras Cardiol. 2025;122(9). doi: https://doi.org/10.36660/abc.20250619i, two authors were added to the author list: Erika Maria Gonçalves Campana, affiliated with Hospital Universitário Pedro Ernesto, Rio de Janeiro, RJ – Brazil, included as affiliation 48; and Ricardo Stein, affiliated with Universidade Federal do Rio Grande do Sul, Porto Alegre, RS – Brazil, included as affiliation 49. In the Conflict of Interest Disclosures section (page 3), the following disclosures were added:

**Table:** 

Guideline for Chronic Coronary Syndrome – 2025	
The report below lists declarations of interest as reported to the SBC by the experts during the period of the development of these statement, 2024/2025.	
Expert	Type of relationship with industry
Erika Maria Gonçalves Campana	Financial declaration A - Economically relevant payments of any kind made to (i) you, (ii) your spouse/partner or any other person living with you, (iii) any legal person in which any of these is either a direct or indirect controlling owner, business partner, shareholder or participant; any payments received for lectures, lessons, training instruction, compensation, fees paid for participation in advisory boards, investigative boards or other committees, etc. from the brazilian or international pharmaceutical, orthosis, prosthesis, equipment and implants industry: - Lectures: Servier: Triplixam; Biolab: Press Pluss, Bhena; AstraZeneca: Xarelto; EMS Bramicar. Other relationships Funding of continuing medical education activities, including travel, accommodation and registration in conferences and courses, from the brazilian or international pharmaceutical, orthosis, prosthesis, equipment and implants industry: - Servier: European Society of Cardiology Congress.
Ricardo Stein	Nothing to be declared

**Editorial team**
